# Classification of Clinical Isolates of *Klebsiella pneumoniae* Based on Their *in vitro* Biofilm Forming Capabilities and Elucidation of the Biofilm Matrix Chemistry With Special Reference to the Protein Content

**DOI:** 10.3389/fmicb.2019.00669

**Published:** 2019-04-04

**Authors:** Ashish Kumar Singh, Shivangi Yadav, Brijesh Singh Chauhan, Nabarun Nandy, Rajan Singh, Kaushik Neogi, Jagat Kumar Roy, Saripella Srikrishna, Rakesh Kumar Singh, Pradyot Prakash

**Affiliations:** ^1^Bacterial Biofilm and Drug Resistance Research Laboratory, Department of Microbiology, Institute of Medical Sciences, Banaras Hindu University, Varanasi, India; ^2^Molecular Immunology Laboratory, Department of Biochemistry, Institute of Science, Banaras Hindu University, Varanasi, India; ^3^Cell and Neurobiology Laboratory, Department of Biochemistry, Institute of Science, Banaras Hindu University, Varanasi, India; ^4^Cytogenetics Laboratory, Department of Zoology, Institute of Science, Banaras Hindu University, Varanasi, India; ^5^Department of Pharmaceutics, Indian Institute of Technology, Banaras Hindu University, Varanasi, India

**Keywords:** biofilm, brain heart infusion broth, methionine, *S*-adenosyl methionine, mannose, *N*-acetyl glucosamine, matrix assisted laser desorption ionization tandem mass spectroscopy

## Abstract

*Klebsiella pneumoniae* is a human pathogen, capable of forming biofilms on abiotic and biotic surfaces. The limitations of the therapeutic options against *Klebsiella pneumoniae* is actually due to its innate capabilities to form biofilm and harboring determinants of multidrug resistance. We utilized a newer approach for classification of biofilm producing *Klebsiella pneumoniae* isolates and subsequently we evaluated the chemistry of its slime, more accurately its biofilm. We extracted and determined the amount of polysaccharides and proteins from representative bacterial biofilms. The spatial distribution of sugars and proteins were then investigated in the biofilm matrix using confocal laser scanning microscopy (CLSM). Thereafter, the extracted matrix components were subjected to sophisticated analysis incorporating Fourier transform infrared (FTIR) spectroscopy, nuclear magnetic resonance (NMR) spectroscopy, one-dimensional gel-based electrophoresis (SDS-PAGE), high performance liquid chromatography (HPLC), and MALDI MS/MS analysis. Besides, the quantification of its total proteins, total sugars, uronates, total acetyl content was also done. Results suggest sugars are not the only/major constituent of its biofilms. The proteins were harvested and subjected to SDS-PAGE which revealed various common and unique protein bands. The common band was excised and analyzed by HPLC. MALDI MS/MS results of this common protein band indicated the presence of different proteins within the biofilm. The 55 different proteins were identified including both cytosolic and membrane proteins. About 22 proteins were related to protein synthesis and processing while 15 proteins were identified related to virulence. Similarly, proteins related to energy and metabolism were 8 and those related to capsule and cell wall synthesis were 4. These results will improve our understanding of *Klebsiella* biofilm composition and will further help us design better strategies for controlling its biofilm such as techniques focused on weakening/targeting certain portions of the slime which is the most common building block of the biofilm matrix.

## Introduction

*Klebsiella pneumoniae*, a member of Enterobacteriaceae is a remarkable human pathogen. In recent past, the prevalence of *Klebsiella pneumoniae* infections has exceedingly raised in the clinical settings (Decré et al., 2011; Vuotto et al., 2014). Recently, it has emerged globally as a multidrug-resistant hospital pathogen for which there are few treatment options (Paczosa and Mecsas, 2016). The investigations about its virulence factors have added newer insights to its self-protective pathogenic strategies, which comprise mainly of fimbriae, capsule, and lipopolysaccharide responsible for attachment to host surface, protection against phagocytosis, desiccation, and complement evasion, respectively (Victor et al., 2007; El Fertas-Aissani et al., 2013; Piperaki et al., 2017). Besides, type 1 and type 3 fimbriae are revealed to arbitrate its colonization on passive/inert abiotic surfaces (Murphy and Clegg, 2012; Murphy et al., 2013). Recently, Ferry et al. have reported implant-associated ESBL producing *Klebsiella pneumonia* involved in bone and joint infection in a healthy 40-year-old man who underwent a bifocal fracture of the left leg (Ronde-Oustau et al., 2017).

Biofilms are the complex structural consortium of exopolysaccharide matrix, of microbial origin and shaped by several host factors, within which bacteria reside (Donlan and Costerton, 2002; Soto, 2013). Biofilms are extensively investigated over the past few decades mainly owing to the involvement in almost 80% of bacterial infections, particularly prevalent in device-related infections, infections on body surfaces and chronic infections. These biofilms are of serious concern as they impart protection from host defense mechanisms and to conventional antimicrobial therapy, which markedly influences the antimicrobial treatment outcomes (Römling and Balsalobre, 2012; Kostakioti et al., 2013; Vuotto et al., 2014; Singh et al., 2017a).

*Klebsiella pneumoniae* forms biofilm as an essential step in its pathogenesis (Wu et al., 2011). The biofilm can be conceived on the skin and soft tissues, lungs, urinary bladder, etc. (Piperaki et al., 2017). Implant associated infections by *Klebsiella* spp. are frequently reported. Presence of small colony variants were also reported (Ronde-Oustau et al., 2017). Unlike *Staphylococcus aureus/epidermidis*, the biofilms associated with *Klebsiella* mediated urinary tract infections (UTI) are not due to the indwelling devices rather due to the bacterial adherence to bladder mucosal tissue and on the mucosal surface of the acini of prostate tissue as signified in the rat model of bacterial prostatitis (Murphy et al., 2013).

The prerequisites for optimal biofilm formation vary considerably among bacteria. These factors include source and concentration of carbon and nitrogen, pH, ionic strength, incubation time, nature of the adherent material and temperature, etc. (Singh et al., 2017a). Although investigators have sought to optimize the conditions required for biofilm development by *Klebsiella* isolates, some of the parameters like optimum concentration and nature of carbon and nitrogen sources, salt, amino acids, and richness of medium have not been scrupulously explored (Seifi et al., 2016). Besides, no accord pertaining to the quantitative interpretation and categorization based on biofilm biomass was observed among investigators. Additionally, the method utilized till date for the quantification of biofilm cannot be reproduced in every laboratory settings (Maldonado et al., 2007). Therefore, a consensus guideline for *in vitro* biofilm synthesis by clinical isolates of *K. pneumoniae* and its categorization is direly needed.

To the best of our knowledge, the effect of growth medium, time period, fixation, and then supplementation with carbon source (sugars), nitrogen source [amino acid(s)] and salt levels on the characteristics of *K. pneumoniae* biofilm has received comparatively scant attention. As the majority of investigators have simply repeated, the same method by exposing *K. pneumoniae* to the conditions as it was utilized in the cases of *Staphylococcus aureus* and *Staphylococcus epidermidis* (Maldonado et al., 2007; Seifi et al., 2016). In our preceding work on *Staphylococcus aureus* biofilm standardization, we observed supplementation dependent bacterial proliferation, i.e., when supplement mix was added to it, bacterial counts also increased linearly. This indicated active division of bacteria during biofilm biogenesis (Singh et al., 2017a). Similarly, it would be interesting to investigate the growth kinetics of Gram-negative slime producing bacterial isolate, *Klebsiella pneumoniae*.

Although the *Klebsiella* biofilm has been considered to be exclusively sugar, variations in the colony characteristics of the clinical isolates has been observed with regard to its external smoothness (texture) and consistency indicating differences in the composition of slime produced (Boddicker et al., 2006; Li et al., 2014; Piperaki et al., 2017). The matrix content of a biofilm may differ in quantity and nature of its constituents depending on the environmental factors. Various environmental factors have been reported to promote slime production including high levels of oxygen, limited availability of nitrogen, desiccation, and nutrient deprivation (Li et al., 2014). Despite research on *Klebsiella* biofilms, in particular, the slime/matrix composition in its adherence, the precise role of matrix composition in biofilm architecture is not completely understood. So far, very limited studies regarding the biochemical and biophysical parameters to ascertain the composition of biofilm matrix have been performed (Magana et al., 2018).

Further, the limitations of the therapeutic options against *Klebsiella pneumoniae* due to its innate capabilities to form biofilm and harboring determinants of multidrug resistance, the bacterium demands new measures for the management of the infections produced (Shon et al., 2013; Yao et al., 2015). Thus, in addition to assessment of biofilm forming capacity of *Klebsiella*, there is a need to explicate the chemistry of its biofilm matrix to ascertain any divergence from the previously accepted fact of sugar richness in its slime.

The bane of biofilm research is always been false positivity or negativity in the quantitative and qualitative estimation of biofilm (Magana et al., 2018). Therefore, the intent of the present study was twofold: first, to standardize a simple spectrophotometric method for the development of a consensus protocol for achieving maximum *in vitro* biofilm formation by the clinical isolates of *Klebsiella pneumoniae* utilizing a defined supplementation and the second was to thoroughly elucidate the biofilm matrix chemistry. In the present work, we extracted and determined the amount of polysaccharide and protein from representative biofilms of the bacteria isolated from various clinical specimens. The distribution of these two components was investigated across the biofilm matrix using confocal laser scanning microscopy (CLSM). Thereafter, the extracted matrix was subjected to sophisticated analysis incorporating Fourier transform infrared (FTIR) spectroscopy, nuclear magnetic resonance (NMR) spectroscopy, one-dimensional gel-based electrophoresis (SDS-PAGE), high performance liquid chromatography (HPLC), and matrix-assisted laser desorption/ionization tandem mass spectrometry (MALDI MS/MS) analysis. Besides, the quantification of various constituents like proteins, total sugars, uronates, total acetyl content was also done.

## Materials and Methods

### Bacterial Isolates

Two hundred fifty-seven non-repetitive, consecutive isolates of *K. pneumoniae* isolated from the clinical samples namely stool, blood, pus, and urine, collected from various outpatients (outpatient departments [OPDs]) and inpatients wards of University Hospital, Banaras Hindu University over a span of 8 months (March 2017–October 2017), were included in the present study. The study protocol was approved by the Institutional Ethical Committee of Institute of Medical Sciences, Banaras Hindu University (Dean/2015-16/EC/1707). The bacterial identification was performed using conventional bacteriological techniques, such as colony morphology, Gram-staining, and various biochemical tests (Gerald et al., 1996).

### Determination of Antimicrobial Resistance

Antibiotic susceptibility testing of the isolates was performed by modified Kirby-Bauer method in conformity with the Clinical and Laboratory Standards Institute guidelines 2016 using 14 antibiotic disks including ampicillin (10 μg), amoxicillin/clavulanate (20/10 μg), cephalexin (30 μg), cefuroxime (30 μg), gentamicin (120 μg), ciprofloxacin (5 μg), levofloxacin (5 μg), co-trimoxazole (23.75/1.25 μg), amikacin (30 μg), piperacillin and tazobactam (100/10 μg), cefepime (30 μg), ertapenem (10 μg), meropenem (10 μg), and imipenem (10 μg) (Clinical and Laboratory Standards Institute, 2016).

All the materials required for the culture isolation, biochemical identification and susceptibility testing were procured from HiMedia Laboratories, Mumbai unless specified otherwise.

### Standardization of *in vitro* Synthesis of Biofilm in Tissue Culture Grade Microtiter Plates

In the present study, the effect of various parameters on *in vitro* biofilm formation was evaluated for all 257 *K. pneumoniae* clinical isolates using 96 well flat bottom tissue culture plates (TCP) (Nunc, Denmark) employing protocol as described earlier with minor modifications (Singh et al., 2017b, 2018).

Briefly, *Klebsiella* isolates were grown on BHI agar overnight at 37°C. One or two colonies from the growth over BHI agar plate were inoculated into 5 ml of BHI and LB broths and were incubated statically for approximately 8 h at 37°C to generate fresh late-exponential-phase cultures. Four microliters of each late-exponential phase culture (absorbance at 600 nm [OD: λ_max_600] ∼ 0.2) was diluted 1:50 with the respective broths.

Each well of TCP plate was filled alternately with 190 μl aliquots of either LB or BHI broths and then 10 μl of a diluted bacterial suspension was added to it. The four different plates were incubated statically at 37°C and were scanned after 6, 12, 18, and 24 h of incubation individually after subjecting to crystal violet (CV) assay. The procedure was repeated thrice. The procedure outlined previously for staining, washing, drying, destaining, and plate reading was followed. Briefly, after respective incubations, the plates were upturned and gently tapped to remove residual broth. The wells were rinsed twice with 200 μl of phosphate buffer saline (PBS) (pH 7.4) to remove planktonic bacteria before fixation. The plates were incubated for chemical fixation by 175 μl sodium acetate (2% w/v). The biofilms were stained with 175 μl of 0.5% (w/v) CV (Sigma, St. Louis, MO, United States) for 5 min. The excess CV was drained off, and the plates were rinsed with running tap water until runoff was clear. For elution, we used 200 μl ethanol–acetone mixture (80:20) and left at room temperature for 30 min. Elute was then placed in wells of other TCP to take absorbance readings at λ_max_ 570 nm, the wavelength at which the CV used had the absorbance maxima in a 96-well microplate reader (Synergy H1 Hybrid Multi-Mode Reader version 3.02.1, BioTek Instruments, Inc., Winooski, VT, United States). The mean absorbance from control wells containing medium alone was deducted from the mean absorbance of the other wells.

### Supplementation of Growth Medium With Sugars, Amino Acids, and Salts

The effect on biofilm formation after supplementation with glucose and sodium chloride in various molar concentrations was observed on all the isolates employing the procedure as reported previously for *S. aureus* (Singh et al., 2017a). In addition, the effect of two amino acids viz. methionine (in concentrations 2, 4, 8, 10 mM) and *S*-adenosyl methionine (in concentrations 5, 10, 15, 20 mM) were also investigated.

Based on the observations of average maximum biofilm formed by supplementation of the individual ingredient, a solution consisting of optimum concentrations of sugars, salts and amino acids (supplement mix) was made and applied as growth medium for *in vitro* synthesis of biofilm by all the isolates.

### Categorization of Isolates Based on Biofilm Forming Capacity

In this study, *S. epidermidis* ATCC 12228 and sterile broth served as the negative control. While *S. epidermidis* ATCC 35984 (high biofilm producer) and *S. epidermidis* ATCC 35983 (moderate biofilm producer) were employed as positive controls. All experiments with clinical isolates were performed in quadruplet, i.e., each isolate was inoculated in four wells simultaneously and repeated thrice (on different days), and then, absorbance values were averaged and standard deviation (SD) was calculated.

We attempted to classify the biofilm forming isolates of *Klebsiella* into four groups namely non-biofilm former (NBF), weak biofilm-former (WBF), moderate biofilm-former (MBF), and high biofilm-former (HBF) as reported previously (Stepanovic et al., 2000; Singh et al., 2017a).

### Growth Rate Analysis

The growth rates of four representative high biofilm producing isolate each recovered from urine, blood, stool, and pus samples were analyzed spectrophotometrically (employing Synergy H1 Hybrid Multi-Mode Reader). Briefly, the bacterial cells at late-exponential phase were inoculated into TCP such that its absorbance at λ_max_ 600 nm was approximately 0.01. The optical density of each well was then scanned at λ_max_ 600 nm by periodic measurements after every 15 min for 4.5 h. To determine the maximum growth rate of each isolate, the slope of the linear part of the growth curve (*R*^2^, ≥0.98) was determined for at least six data points of the semi-logarithmic plot of absorbance (ln[OD_600_]) v/s incubation time (in hours).

### Elucidation of the Chemistry of Biofilm Matrix

To empirically predict the chemistry of biofilm matrix with regard to its sugar, protein, *e*DNA produced by clinical isolates of *K. pneumoniae*, we subjected the representative isolates (four isolates) of each category to form biofilm for 72 h and then subjected them for digestion by 10 μl proteinase K (20 μg/ml), 15 μl sodium metaperiodate (NaIO_4_) (25 μg/ml) and 10 μl DNase I (10 μg/ml) individually for 30 min. Then, we made four sets of the mocktail by incorporating these reagents as follows: (**1**) Proteinase K, NaIO_4_ (**2**) NaIO_4_, DNase I (**3**) Proteinase K, DNase I (**4**) Proteinase K, NaIO_4_, DNase I. We used 25 μl of each mocktail for 30 min in the current study.

To corroborate the results obtained using various chemicals and mocktails over 72 h old biofilms; we undertook quantitation of its diverse contents like protein, sugars and extra polymeric nucleic acids (*e*DNA). Briefly, the representative high slime producing isolates of *K. pneumoniae* isolated from blood, pus, urine, and stool samples were subjected to biofilm formation. The cells were grown for 72 h in 50 ml BHI broth as described earlier. Biofilm matrix was extracted by the method described by Singh et al. (2017b) with minor modifications. Briefly, the 72 h old biofilm was agitated to extricate the matrix. To 10 ml of the dislodged biofilm sludge, 40 ml of 36.5% formaldehyde (Merck, Germany) was added to fix the cells. Cell suspensions were then centrifuged 9,250 × *g* for 1 h. at 4°C. To extract components, the residual pellets were suspended in 10 ml of 1.5M NaCl and forthwith centrifuged at 5,000 × *g* for 10 min at 25°C, followed by the transfer of supernatant to a fresh test tube as exopolysaccharide fractions.

#### Protein Extraction and Estimation

To thus obtained supernatant, 2.5 ml of 10 mM Tris-Cl (Sigma-Aldrich, United States) pH 7.8 was added and vortexed with subsequent addition of 20 mM dithiothreitol (DTT) (Sigma, St. Louis, MO, United States) and 1 mM phenyl methyl sulfonyl fluoride (PMSF) (Sigma, St. Louis, MO, United States). The cell suspension was again vortexed well and was centrifuged at 12,000 × *g* for 30 min at 4°C. The supernatant was transferred to a fresh centrifuge tube where 100% trichloroacetic acid (TCA) (w/v) (Merck, Germany) was added in 4:1 ratio. The supernatant was placed at -20°C for 60 min for precipitation of the protein. After precipitation, the solution was centrifuged at 12,000 × *g* for 90 min at 4°C. The protein pellet was washed twice with 200 μl of chilled acetone by centrifuging at 14,000 × *g* for 10 min and was air dried thereafter. To the dried pellet, 500 μl of rehydration buffer [8M urea, 2M thiourea, 2% (w/v) cholamidopropyldimethylammoniopropane sulfonate (CHAPS), and 0.3% (w/v) DTT (Merck, Germany)] was added and the protein pellet was placed at 4°C for 8–10 h with intermittent vortexing to solubilize the protein pellet. The extracted matrix was subsequently analyzed for total protein content using Bradford’s method with BSA as a standard (Bradford and Williams, 1976). Briefly, BSA solutions were made in MilliQ water in different dilutions for the calibration curve, and 10 μl of the sample was dispensed in TCP wells. To those wells, 200 μl of the diluted Bradford assay solution (1:5 [v/v] in MilliQ water and filtered through Wattman filter paper) was added and the samples were incubated for 30 min at room temperature. The absorbances were measured at 595 nm using Synergy H1 Hybrid Multi-Mode Reader. The procedure was performed in triplicate for each sample.

#### Sugar Extraction and Estimation

The supernatant obtained after treatment of 1.5M NaCl was then subjected to total sugar estimation by phenol–sulfuric acid method (Chiba et al., 2015). Briefly, 20 μl of the isolated exopolysaccharide was mixed with 20 μl of 5% phenol in the 96 wells plate. Then, 100 μl of sulfuric acid (98%) was added and incubated for 10 min at 25°C. The concentration was measured at 492 nm with Synergy H1 Hybrid Multi-Mode Reader. Glucose was used as a standard.

#### Uronic Acid Estimation

To different wells of 96 well microtiter plate, 50 μl of extracted matrix sample and standard (galacturonic acid, concentration range of 200–1.562 μg/well) were placed in dilutions. Then, 20 μl of sulfamic acid reagent (4M sulfamic acid in water and pH maintained at 1.6 using saturated KOH solution) was added and mixed thoroughly. To this, 200 μl of concentrated sulfuric acid (98%) containing 75 mM disodium tetraborate was carefully added. The mixture was vortexed well and kept in boiling water bath for 15 min followed by cooling at ambient temperature for 20 min. Then, 50 μl of 0.125% carbazole in absolute ethanol was added. After heating in boiling water bath for 10 min and cooling at room temperature for 15 min, the plate was read in Synergy H1 Hybrid Multi-Mode Reader at a wavelength of 550 nm (Wingender et al., 2001). Finally, the uronic acid content of each extracted samples were interpreted with respect to the standard using GraphPad Prism version 5.01.

#### Acetyl Group in Sugars

Acetyl content of sugars is determined by the method proposed by Hestrin (1949) with modifications. Briefly, hydroxylamine reagent was prepared by mixing equal volumes of 2M hydroxylamine and 3.5M NaOH. 250 μl of the test samples and controls were mixed with 500 μl of hydroxylamine reagent. After 1 min of incubation at room temperature, 250 μl of 4.2M HCl is added and mixed thoroughly. Subsequently, 250 μl of ferric chloride solution (0.37M FeCl_3_ in 0.1M HCl) is added and immediately the absorbances were read at 540 nm. Calibration plots were made using glucose pentaacetate with stock of 500 μg/ml and the amount of acetyl groups were interpreted using GraphPad Prism version 5.01.

#### Estimation of *e*DNA

To quantify DNA in the extracted biofilm matrix fractions, we purified the DNA using conventional method utilizing phenol, chloroform, and isoamyl alcohol followed by ethanol precipitation (Chiba et al., 2015). Briefly, the 200 μl supernatant was treated first with 200 μl of phenol–chloroform–isoamyl alcohol (25:24:1) followed by 150 μl of chloroform–isoamyl alcohol (24:1). Then, the aqueous phase was collected. To 100 μl of the collected aqueous phase, 10 μl of 3M potassium acetate (pH 5.0) was added and mixed followed by the addition of 300 μl of chilled absolute ethanol and then stored at -20°C overnight. The following day, the precipitated DNA was collected by centrifugation (14,000 × *g*) for 15 min at 4°C, washed with ice-cold 70% (v/v) ethanol, air dried at room temperature, and dissolved in 50 μl of Tris-EDTA (TE) buffer (pH 8). The concentration of the purified DNA was then measured with NanoDrop 2000 (Thermo Fisher Scientific, Waltham, MA, United States). After the quantification, we ran the 1% agarose gel to check the quality and size of the *e*DNA.

### Biophysical Analysis of Biofilm Matrix Components

#### Fourier Transform Infrared (FTIR) and Nuclear Magnetic Resonance Spectrum (NMR) Spectroscopy

The FTIR analysis of the purified exopolysaccharide (sugar) was carried out by KBr pellet technique using Varian Excalibur 3000, (Palo Alto, CA, United States) and the spectrum was measured in the frequency range of 400–4,000 cm^-1^. However, the nuclear magnetic resonance spectrum (^1^H NMR and ^13^C NMR) analyses were carried out using Bruker spectrometer at 300 K at 500.13 MHz equipped with 5 mm broadband probe, using the dialyzed exopolysaccharide dissolved in 600 μl D_2_O as described previously (Lal et al., 2010). ^1^H NMR measurements were obtained at 300 K, and the chemical shifts (ppm) were referred indirectly to acetone. The spectral width was 10,330.578 Hz, and the digital resolution was 0.157 Hz, with an acquisition time of 3.17 s. The spectrum was obtained with 16 scans. ^13^C NMR spectra were also obtained at 300 K at 500.13 MHz, and the chemical shifts (ppm) were referred indirectly to tetramethylsilane. The spectral width was 26,455.02 Hz with the digital resolution of 1.61 Hz and acquisition time of 0.30 s. The spectrum was obtained with 1,024 scans.

#### Sodium Dodecyl Sulfate Polyacrylamide Gel Electrophoresis (SDS-PAGE) Analysis

SDS-PAGE was performed on proteins extracted from the biofilm matrix as an aqueous solution using a 12% (w/v) polyacrylamide gel, following the method previously described (Singh et al., 2017b). The equal amount of isolated matrix proteins (65 μg/ml) of representative isolates was dissolved in deionized water and then electrophoresed at constant voltage (120 V) until the bromophenol blue tracking dye front reached the bottom of the gel. Low-molecular weight protein markers (Prism Ultra Protein Ladder, ab116028, Abcam, India) were used as protein standards, and the protein bands were stained with Bio-Safe Coomassie Blue Stain (Bio-Rad, United Kingdom).

#### High Performance Liquid Chromatography (HPLC) Analysis

To look up the constitution and relative polarity of the common protein bands obtained from the biofilms of representative isolates of *K. pneumoniae*, the bands were first excised and then eluted out followed by subjection to analytical HPLC. Briefly, the excised gel bands were diced into small pieces and placed in fresh micro centrifuge tubes. The gel pieces were destained four times, each with 10 min interval using destaining solution (1:1 ratio of acetonitrile and 25 mM ammonium bicarbonate) until the gel pieces became translucent white. The destained gel pieces were then dehydrated using 100 μl acetonitrile (ACN) followed by thermo-mixing at 600 rpm for 10 min. Subsequently, the gel pieces were rehydrated with 100 μl of 10 mM dithiothreitol (DTT) by incubating them for 60 min. The DTT was removed with subsequent incubation for 45 min with 100 μl of 55 mM iodoacetamide. The residual iodoacetamide was discarded and the gel pieces were re-incubated with 100 μl of ammonium bicarbonate solution for 10 min. Again, the supernatant was discarded and gels were dehydrated with 100 μl of acetonitrile and thermo-mixed with at 600 rpm for 10 min till the gels were dehydrated. Finally, the gel pieces were extracted thrice with extraction buffer [1:1 ratio of acetonitrile and 0.1% trifluoroacetic acid (TFA)] and the supernatant was collected each time into the new micro-centrifuge tube and then freeze-dried overnight in a SpeedVac concentrator (SVC100H, Savant, Thermo Fisher Scientific, Inc., Waltham, MA, United States) equipped with a refrigerated condensation trap. The dried protein mixture was suspended in TA buffer (0.1% TFA and ACN in the ratio of 2:1).

The data were collected on an automatic HPLC system (Shimadzu) with an analytical reversed-phase column using the UV detector operating at 280 nm. A Phenomenex Gemini C18 (110 A°, 5 μm, 250 × 4.6 mm) column was used in this analysis. For this purpose, the elution was carried out with binary solvent systems with a flow rate of 0.8 ml/min at ambient temperature. The mobile phase consisted of methanol (A) and 0.1% TFA in acetonitrile (B). The sample was determined using the above solvents programmed linearly 62% eluent B for 20 min with a constant 38% A. The data were analyzed using HP ChemStation software. Independent peaks were identified and were reported with retention time.

#### MALDI MS/MS Analysis

We looked for the tentative sequence of the proteins/peptides from the common protein bands obtained from the biofilms of representative isolates of *K. pneumoniae* as described earlier for *Enterococcus faecalis* with modifications (Aydin et al., 2012). Briefly, the common protein bands of interest were excised from SDS-PAGE gel and destained thrice in 50% acetonitrile (ACN)/40 mM ammonium bicarbonate (pH 7.4), until the gel pieces became translucent white prior to digestion. The destained gel plugs were then dehydrated using 100 μl 100% ACN (thermo-mixed at 600 rpm for 10 min) and rehydrated with 100 μl of 10 mM DTT (incubated for 60 min). The rehydration was subsequently followed by incubation for 45 min in 100 μl of 55 mM iodoacetamide. Eight microliters of 10 ng/μl trypsin (Promega, Madison, WI, United States) in 150 μl of 40 mM ammonium bicarbonate/20% ACN was added to the diced gel plugs and incubated overnight 37°C. The digested solution was transferred to fresh micro-centrifuge tube. Peptides were then extracted in four volumes of 0.1% TFA in 50% ACN for 2 h at room temperature. The supernatant was collected each time into the fresh micro centrifuge tube and then freeze-dried overnight in a SpeedVac concentrator. The dried protein mixture was suspended in TA buffer (0.1% TFA and ACN in the ratio of 2:1) prior to MALDI MS/MS-analysis.

Protein database excavation was performed using commercial software MASCOT V 2.2.04 (Matrix Sciences). All monoisotopic MS/MS data were searched after conversion to MASCOT-compatible format. The entire SwissProt databases were searched using MASCOT without any fixed modification selected (since no chemical modification was expected during digestion) in the search criteria and the taxonomy selected was bacteria (Eubacteria). Methionine oxidation was the only variable modification considered. Maximum missed cleavages for trypsin was set at 1, peptide charge at 2+ and 3+, peptide mass tolerance at ±0.1%, and MS/MS tolerance at ±0.8 Da.

### Confocal Laser Scanning Microscopic (CLSM) Validation of Chemical Contents of Biofilm Matrix

Biochemical findings and mocktail assay results were further verified by CLSM. For confocal analysis, biofilm was grown in chambered slides as described previously (Wingender et al., 2001; Singh et al., 2017b). Briefly, overnight cultures of *K. pneumoniae* were grown in BHI broth and was diluted 1:100 and grown in Luria Bertani broth to OD∼0.2 at λ_max_ 600 nm. Ten-microliters of diluted suspension of representative bacterial isolates were dispensed into flat-bottom chambered slide containing 490 μl of BHI broth (Nunc, Denmark). The biofilm was statically grown for 72 h. Prior to staining, residual broth was removed by gentle tapping and was washed thrice by phosphate buffer (pH 7.5). Biofilm was fixed using 3% (v/v) formaldehyde for 30 min. After fixation, Concanavalin A (ConA), labeled with tetramethyl-rhodamine isothiocyanate (TRITC) (Life Technologies, United States) was used to access the sugar distribution in biofilm. Besides, we used phalloidin green for the detection of fibrillar amyloid-like proteins in the biofilm matrix. The conjugated lectin was reconstituted with 10 mM phosphate buffer (pH 7.5) and stock solutions of 1 mg/ml was prepared and stored frozen in aliquots of 100 μl. For use, stock solutions were diluted with phosphate buffer to a lectin concentration of 10 μg/ml. Ten-microliters samples of these staining solutions were applied directly to the top of the biofilms. However, 2 μl of 5μM green-fluorescent phalloidin in 10 mM phosphate buffer (pH 7.5) was used.

The Zeiss LSM 510 inverted confocal laser scanning microscope (Carl Zeiss, Jena, Germany) was used to detect the green and red fluorescence from the stains. Optical sections were gathered in 0.2/0.5μm steps perpendicular to the *z*-axis (microscope optical axis) via a Plan-Neofluar 40×/1.3 oil objective or 20× objective using a dual argon-ion (488 nm; green fluorescence) and green helium/neon (555 nm; red fluorescence) laser system.

### Statistical Analysis

The statistical analyses were made on non-repetitive isolates of *K. pneumoniae*. For grouped analysis, two-way analysis of variance (ANOVA) utilizing Bonferroni post-test was used to compute and analyze the differences in absorbance values obtained with different experimental variables. However, as per need differences in various parameters studied herein within the biofilms produced by different isolates with and without supplementation were analyzed by performing a one-way analysis of variance (ANOVA) test with a Tukey’s and Dunn’s multiple comparison posttests using GraphPad Prism version 5.01 (GraphPad Software, La Jolla, CA, United States). *P*-values of ≤0.05 were considered as statistically significant.

## Results

### Categorization of Isolates Based on Biofilm Forming Capacity

In this study, while attempting to elucidate the various conditions for maximum biofilm formation, we observed the effects of growth medium, incubation period, effects of sugar, salt and amino acid supplementation on the biofilm forming capacity of *K. pneumoniae* isolates. Initially the screening was done on the control strains (ATCC 35984, 35983, and 12228) which was later on applied and tested over all the 271 clinical isolates of *K. pneumoniae*. Insignificant difference was observed in absorbance indices in both the broths (BHI and LB, *P* = 0.6825, *P* < 0.05) (Supplementary Figure S1A and Supplementary Data S1). Similarly, 18 h of incubation was found sufficient for all the isolates to produce biofilms (Supplementary Figure S1B and Supplementary Data S2). Increasing sodium chloride supplementation from 171 mM to 684 mM at 37°C, enhanced biofilm formation among majority of isolates (Supplementary Figures S2A,B and Supplementary Data S3). Similarly, as the concentration of glucose was escalated from 111 mM to 444 mM, all the clinical isolates formed thicker biofilms at 37°C (Supplementary Figures S3A,B and Supplementary Data S4). The biofilm formation of all the clinical isolates was notably reinforced by methionine and *S*-adenosyl methionine at the concentrations below 10 mM and 20 mM, respectively (Supplementary Figures S4A–C and Supplementary Data S5). Finally, the synergistic effect of optimized sugar, salt and amino acids supplementations on the biofilm forming capacity was observed with the final combinatorial supplementation of 444 mM glucose, 684 mM NaCl and 8 mM methionine and 15 mM *S*-adenosyl methionine (supplement mix). The mean absorbance for the biofilms formed by these isolates increased almost 38% after supplementation (1.323 ± 0.05814) compared to the unsupplemented broth (0.9589 ± 0.04124) at 37°C (Supplementary Figure S5).

Based on our presumption of four different classes of biofilm formers, we attempted to categorize the 257 *Klebsiella pneumoniae* isolates [Blood (*n* = 68), Pus (*n* = 72), Stool (*n* = 22), Urine (*n* = 95)] with or without supplementation. Out of 257 isolates, 32 isolates were non-former (12.45%), 71 were weak-formers (27.62%), and 136 were medium formers (52.91%), while 18 isolates were high formers (∼7%). However, we noticed that some of the isolates have shown an unanticipated increase in the absorbance after supplementation with additives like sugar, salt and amino acids. Of note, control strains were also shifted from their respective categories after the aforesaid supplementation. Therefore, we felt the need to modify the classification criterion so that the isolates showing exaggerated biofilm response may be accommodated in their pertinent categories.

We, therefore, defined the negative control index as the square root of the cumulated summation of squares of absorbances (OD) of ATCC 12228 in supplemented and unsupplemented BHI and uninoculated supplemented and unsupplemented BHI as follow:

Negative control index

$$\begin{array}{c} =\;\sqrt{\left(\{{\left(\mathrm{OD}\mathrm{of}\mathrm{ATCC}12228\mathrm{in}BHI``Unsuppl''\right)}^{\wedge }(2)+{\left(\mathrm{OD}\mathrm{of}\mathrm{ATCC}12228\mathrm{in}BHI``Suppl''\right)}^{\wedge }(2)+{\left(\mathrm{OD}\mathrm{of}BHI``Unsuppl''\right)}^{\wedge }(2)+{\left(\mathrm{OD}\mathrm{of}BHI``Suppl''\right)}^{\wedge }(2)\}\right)} \end{array}$$

And then, a minimum cut off OD was defined as OD_cut_ = OD_avg_ of negative control index + 3 × SD of ODs of negative control index.

We applied the aforesaid criteria for an ordinal classification of *K. pneumoniae* isolates for their biofilm forming capabilities for the ease of interpretation and to accommodate the isolates with exaggerated biofilm formation in their respective classes (Table 1 and Supplementary Table S1). Bacterial isolates were thus categorized into six classes namely non-, weak, moderate, potentiated moderate, high and super biofilm formers based on the adherence indices of the isolates. Post supplementation, 30 (93.75%) of the total non-formers shifted to the weak former category, 28 (39.42%) of the total weak formers shifted to the moderate former category. Similarly, 67 (49.26%) of the total medium former isolates shifted to potentiated medium former category, 16 (11.76%) to the high former category and 8 (5.88%) to the super former category. Out of 18 high former isolates, 10 (55.55%) isolates were shifted to super former category. The major shifting is summed up in Table 1.

**Table 1:** Classification criteria of *Klebsiella* isolates based on their biofilm forming capacity.

OD_cut_ = OD_avg_ of negative control index + 3 × SD of ODs of negative control index (Average negative control index = 0.303 ± 0.0071)			
	Formula for calculating	Range of OD obtained	% occurrence
Classes	resultant OD	in this study	before/after
Non-biofilm-former (NBF)	OD ≤ OD_cut_	≤0.324	12.45/0.78
Weak biofilm-former (WBF)	OD_cut_ < OD ≤ 2 × OD_cut_	0.325–0.648	27.62/16.73
Moderate biofilm-former (MBF)	2 × OD_cut_ < OD ≤ 4 × OD_cut_	0.649–1.296	50/28.40
Potentiated moderate biofilm-former (pMBF)	4 × OD_cut_ < OD ≤ 6 × OD_cut_	1.297–1.944	–/26.07
High biofilm-former (HBF)	6 × OD_cut_ < OD ≤ 8 × OD_cut_	1.945–2.592	0.38/10.12
Super biofilm-former (SBF)	OD ≥ 8 × OD_cut_	≥2.593	–/10.12

### Growth Rates in BHI Broth Before and After Supplementation

The mean growth rates of the four high slime producing representative *Klebsiella* isolates from urine, pus, blood, and stool were significantly different and were found to be 0.2807 ± 0.006047 h^-1^, 0.4642 ± 0.01208 h^-1^, 0.4371 ± 0.007423 h^-1^, and 0.2955 ± 0.02255 h^-1^, respectively, in BHI broth at 37°C (*P* ≤ 0.001) (Supplementary Figures S6A,A′,B,B′ and S7). The supplementation of BHI broth with glucose, NaCl, methionine, and *S*-adenosyl methionine stimulated the growth significantly as evident from the shorter lag phase and higher growth rate (*P* ≤ 0.001) (Supplementary Table S2). Upon supplementation, the highest increase in the growth rate was observed in case of stool isolates (17.194%) followed by urine (11.878%), blood (6.28%), and pus isolates (4.405%) (Supplementary Figure S8).

### Matrix Chemistry Assay

Digestion of biofilm matrix by different chemicals revealed the differences in matrix chemistry of isolates coming from different sites of infections with respect to its protein, sugar and *e*DNA contents. We found proteinase K to be exceptionally efficient in the degradation of biofilms formed by blood and pus isolates (Supplementary Figure S9). Upon administration of proteinase K over biofilm(s) of blood isolates, 68.567% drop in biofilm absorbance was seen while comparatively lesser 58.279% degradation was observed with NaIO_4_. However, DNase I slashed the absorbance down to 28.058% compared to the control. The similar trend was noticed with the biofilms formed by stool isolates for proteinase K digestion while NaIO_4_ had barely any effect. Interestingly, sodium metaperiodate (NaIO_4_) mediated digestion was significantly marked in urine isolates (approximately same as blood isolate) (Supplementary Figure S9). In case of Pus isolates, we found almost equal reduction by proteinase K and NaIO_4_. However, NaIO_4_ had more obvious effect. Reduction up to 56% was observed when NaIO_4_ was used for degradation of biofilms formed by urine isolates. It was interesting to note the relative inertness of these biofilms for proteinase K degradation (only 6.976% reduction in absorbance). However, in case of the biofilms formed by the blood isolates, both proteinase K (68.567%) and NaIO_4_ (58.279%) reduced the absorbance to comparable extents. Mocktail containing proteinase K and DNase I was found highly significant in biofilm matrix disintegration in case of blood, pus and stool isolates. However, for urine isolates, mocktail of NaIO_4_ and DNase I was found to be exceptionally effective (Supplementary Table S3). Of note, the degradation mediated by the mocktail containing Proteinase K, NaIO_4_ and DNase I was found to degrade the biofilms of all the isolates irrespective of their sites of infection/isolation.

### Biophysical Analysis of Biofilm Matrix Contents

#### (a) Fourier Transform Infrared Spectroscopy

**FIGURE 1:**
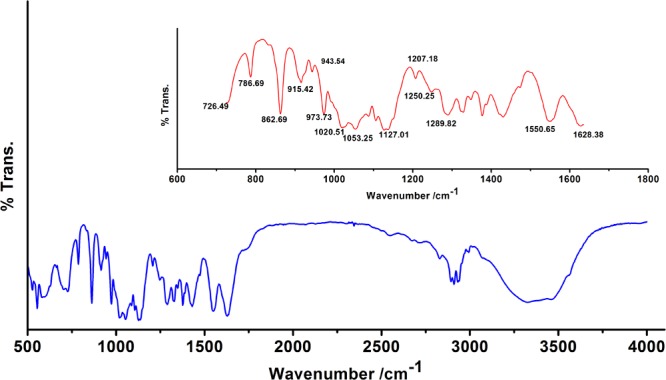
Fourier transform infrared (FTIR) absorption spectrum of the sugar fraction of *Klebsiella* biofilm matrix. Inset: Zoomed overview in the range 650–1,650 cm^-1^. Absorption peaks in mid and far-infrared regions (500–1,600 cm^-1^) reflected the absorption of sugars. The spectrum depicts the presence of β-glucans, mannans, sugar acids, and amines. Peaks at 1,207.18 and 1,250.25 cm^-1^ indicate the presence of 2′-acetylation indicating the presence of acetylated uronates. Apart from the sugar peaks, we also noted a broadband peak indicative of proteins (amide II) at 1,550.65 cm^-1^.

We observed band maxima in the mid and far-infrared regions with absorption at 500–1,600 cm^-1^ and was found this region to be highly useful for the identification of *K. pneumoniae* exopolysaccharide (isolate no. 1739). The peaks showed the presence of hydrogen bonded compound(s), possibly sugar acid(s) or sugar amine(s). Presence of band at 700–1,500 cm^-1^ indicates the presence of glucans (Table 2 and Supplementary Data S6). Polysaccharides showed absorption bands at 726.49, 786.69, 862.69, 915.42, 973.73, 1,020.51 and 1,053.25 cm^-1^. The absorption around 1,000 cm^-1^ (973.73, 1,020.51, and 1,053.25 cm^-1^) was typical for the glucose in pyranose form. However, the band at 786.69 and 862.69 cm^-1^ was of mannose. The FTIR spectra exhibited sharp specific absorbance of O–H stretching at 3,326.72 cm^-1^, while weak C–H stretchings were seen at 2,931.69, 2,310.13, and 2,893.25 cm^-1^. We further observed sharp asymmetric C=O stretching of carboxylate at 1,628.38 cm^-1^, C–C ring stretching at 1,430.38 cm^-1^ and C–N stretching of a primary amine at 1,289.82 cm^-1^. Besides, we also observed the sharp peaks for acetyl groups H_3_C–C = 0 at 1,207.18, 1,250.25 cm^-1^ (Figure 1). Amidst the sugar bands, we also observed the broad absorption of amide II protein bands at 1,550.65 cm^-1^. The absorption peaks in mid-infra-red region (726.49, 786.69, 862.69, 915.42, 943.54, 973.73, 1,020.51, 1,053.25, and 1,127.01 cm^-1^) clearly indicated the presence of β-glucans, mannans, and sugar acids and amines in *K. pneumoniae* biofilm matrix (Table 2).

**Table 2:** Annotated peaks for Fourier transform infrared (FTIR) spectroscopic analysis.

Peaks	Probable moiety	Peaks	Probable moiety
786.69 cm^-1^	Mannans (β1 → 6)	1,250.25 cm^-1^	Acetyl group
862.69 cm^-1^	Mannans (β1 → 4)	1,289.82 cm^-1^	C–N stretching (1° amine)
726.49 cm^-1^	Glucans	1,430.38 cm^-1^	C–C ring stretching
786.69 cm^-1^	Glucans	1,450.6 cm^-1^	Bending of (δCH_2_, δCH_3_) from proteins (amide III)
862.69 cm^-1^	Glucans	1,550.62 cm^-1^	Proteins (amide II)
915.42 cm^-1^	Mannans	1,628.38 cm^-1^	C=O stretching of carboxylate
943.54 cm^-1^	Glucans	2,310.13 cm^-1^	C-H stretchings
973.73 cm^-1^	Glucose (Pyranose)	2,893.25 cm^-1^	C-H stretchings
1,020.51 cm^-1^	Glucose (β1 → 3)	2,931.69 cm^-1^	C-H stretchings
1,053.25 cm^-1^	Glucose (β1 → 3)	3,326.72 cm^-1^	O–H stretching
1,207.18 cm^-1^	Acetyl group		

#### (b) Nuclear Magnetic Resonance Spectroscopic Analysis of Sugar Content

**FIGURE 2:**
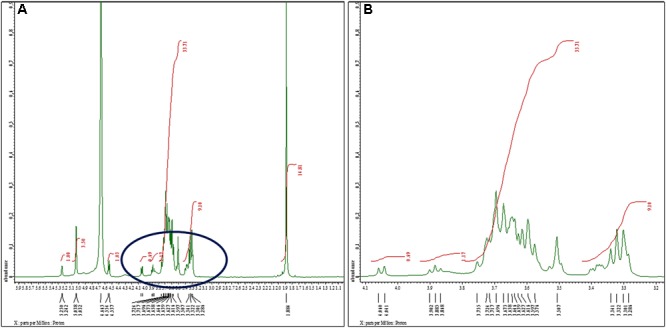
^1^H nuclear magnetic resonance spectra of the sugar content. **(A)** ^1^H NMR (500 MHz, D_2_O): *δ* 5.25 (d, *J* = 4.0 Hz, 1H), 5.03 (d, *J* = 3.0 Hz, 3H), 4.55 (d, *J* = 8.5 Hz, 1H), 4.05 (d, *J* = 9.5 Hz, 1H), 3.88 (t, *J* = 8.5 Hz, 1H), 3.75–3.50 (m, 34H), 3.34–3.38 (m, 9H), 1.88 (s, 15H). We noted signals for *N*-acetyl-glucosamine (GlcNAc) and glucuronates at 1.884 and 3.341 ppm, respectively. Glucose exhibited singlet for α-anomer at chemical shift 5.038 ppm. We observed another singlet peak at 5.032 ppm because of H1 proton of α-anomer of galactose. The peaks at 4.944, 4.995, 5.017, 5.041, and 5.196 ppm were assigned to α- and β-D-glucose, α-D-mannose, and α-l-rhamnose sugars, respectively. **(B)** Extended spectrum of encircled region of **(A)**. Most of the signals stretch out in a narrow region (non-anomeric) ranging between 3.286 and 3.724 ppm.

The structural analysis of *K. pneumoniae* (isolate no. 1739) exopolysaccharide was performed using ^1^H and ^13^C NMR spectroscopy. One of the complications associated with the modeling of carbohydrate ^1^H spectra is that most signals stretch out in a narrow region (non-anomeric); the region between 3.286 and 3.724 ppm features all signals except H1 (most deshielded). The proton spectrum of *K. pneumoniae* exopolysaccharide in D_2_O consists of three distinct regions as shown in Figure 2. The anomeric region, from c. 4.7 to 5.038 ppm contains the anomeric proton signals (H1) from all the sugar rings because these protons are shifted downfield from the other ring protons because of the close proximity of two adjacent oxygen atoms. The five anomeric signals, at shifts of 4.663, 5.032, 5.038, 5.242, and 5.250 ppm, were assigned easily. All of the other ring protons were found overlapping in a narrow region (non-anomeric protons) to form broad hump-like signals from c. 3.286 to 3.724 ppm (Figure 2B). The signal for residual D_2_O in the sample occurred at the edge of the anomeric region in the spectrum at 4.663 ppm, while peak for reference standard (tetramethylsilane) was detected at 0 ppm. The signal for *N*-acetyl-glucosamine (GlcNAc) and glucuronates were observed at 1.884 and 3.341 ppm, respectively (Figure 2A).

As the ‘hump region’ in the spectrum was too complex to interpret, the initial analysis was focused on the anomeric region. Analysis of anomeric region showed the presence of pyranose ring form in α-anomeric configuration. Glucose exhibited singlet for α-anomer at chemical shift 5.038 ppm, assigned at H1. Another singlet was observed at 5.032 ppm as a result of H1 proton of α-anomer of galactose (Figure 2A).

**FIGURE 3:**
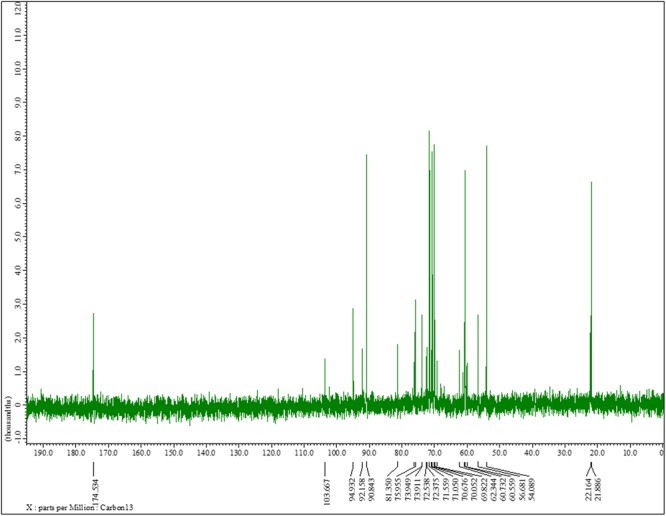
^13^C nuclear magnetic resonance spectra of the sugar content. ^13^C NMR (125 MHz, D_2_O): δ174.5, 103.6, 94.9, 92.1, 90.8, 81.3, 75.9, 73.9, 73.9, 72.5, 72.3, 71.5, 71.0, 70.6, 70.0, 69.8, 62.3, 60.7, 60.5, 56.6, 54.0, 22.1, 21.8. One can observe the chemical shift for α-l-rhamnose at 174.5 ppm in the spectrum. The carbon signals at 73.911 and 70.676 ppm were assigned to α-D-glucose, while carbon signal for β-D-glucose was detected at 60.732 ppm. The two carbon signals for β-pyranose can also be seen in the ^13^C spectrum at chemical shift 70.676 and 60.732, respectively.

The ^13^C NMR spectrum of *K. pneumoniae* exopolysaccharide as shown in Figure 3 demonstrated the presence of carbon signals of the sugars and derivatives at δ174.5, 103.6, 94.9, 92.1, 90.8, 81.3, 75.9, 73.9, 73.9, 72.5, 72.3, 71.5, 71.0, 70.6, 70.0, 69.8, 62.3, 60.7, 60.5, 56.6, 54.0, 22.1, and 21.8. ppm, corresponding to the different ring carbons in the exopolysaccharide chain. The chemical shift for α-l-rhamnose was observed at 174.5 ppm in the spectrum. The carbon signals at 73.911 and 70.676 ppm were assigned to α-D-glucose, while carbon signal for β-D-glucose was detected at 60.732 ppm. Two carbon signals for β-pyranose were also seen in the ^13^C spectrum at chemical shift 70.676 and 60.732, respectively (Figure 3). Overall data indicated the presence of glucose, galacturonate and *N*-acetyl glucosamine moieties in *K. pneumoniae* exopolysaccharide.

#### (c) SDS-PAGE Analysis

**FIGURE 4:**
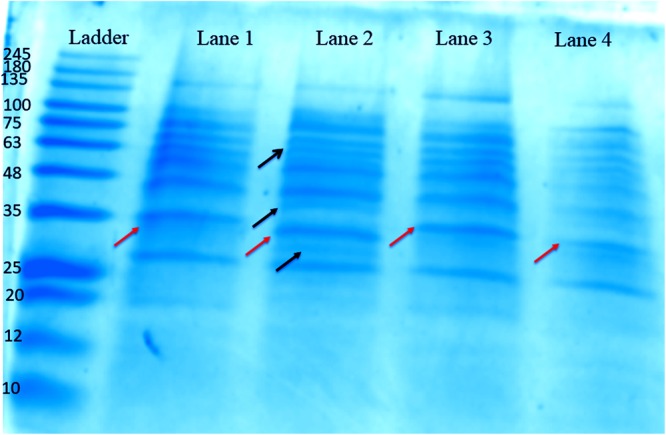
One-dimensional SDS-PAGE gel of proteins extracted from the biofilm matrices of representative high slime producing *K. pneumoniae* isolates from various samples grown in brain heart infusion (BHI) broth. Lane 1: Proteins from the matrix extract of stool isolate 197, harvested at 72 h. Lane 2: Proteins from the matrix extract of blood isolate 1739, harvested at 72 h. Lane 3: Proteins from the matrix extract of pus isolate 2884, harvested at 72 h. Lane 4: Proteins from the matrix extract of urine isolate 10894, harvested at 72 h. Ladder: MW protein marker ranging from 10 to 245 kDa. The orange arrows denote the common protein band (∼35 kDa), which we utilized for HPLC and MALDI analysis. However, the bands shown by black arrows are distinguished bands present in blood isolate 1739.

The concentration of protein obtained from the biofilms of different isolates, i.e., blood, pus, stool, and urine was found to be around 525, 265, 309, and 246 μg/ml, respectively, as estimated by Bradford’s method. The SDS-PAGE gel reveals many different protein bands present in these representative bacterial isolates, which seems to be consistent with the fact that different isolates were isolated from different sites of infections and hence proteins expressed therein is environment specific. However, a few bands were found to be common as well, indicating the basic commonality in structuring motif of the biofilm matrix architecture (Figure 4).

#### (d) HPLC Analysis

We then, sought for the nature of common protein(s) bands which was analyzed and confirmed by performing analytical HPLC (Supplementary Figure S10). We observed, two sharp peaks of the peptides with almost similar retention time (Rt) but varying peak area from different representative isolates in analytical HPLC chromatogram (Supplementary Table S4).

#### (e) MALDI MS/MS Analysis

**FIGURE 5:**
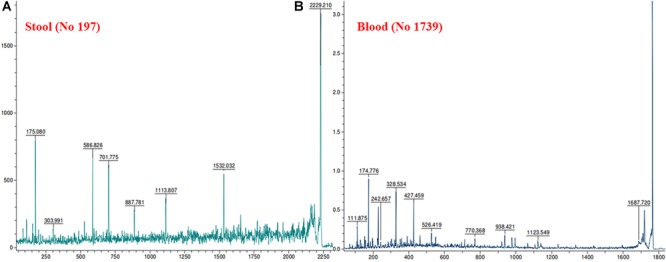
MALDI MS/MS data for the trypsin digested common protein bands. After subsequent digestion and extraction, 1 μl of the peptide mixture was mixed with 1 μl of α-cyano-hydroxycinnamic acid matrix solution (Sigma) together with a lock mass peptide and from this mixture; 1 μl was spotted on the target plate and analyzed by MALDI MS/MS. The protein plugs upon subjection to MALDI MS/MS analysis were evaluated for m/z ranging from 65 to 2,800. **(A)** Data obtained from stool isolates while **(B)** was representative of blood isolate.

MALDI MS/MS (tandem MS) experiments for *K. pneumoniae* specific common bands were performed. Figure 5A,B, 6A,B show representative MALDI MS/MS data from m/z 69.812 to 2229.210 (for stool isolate), 69.915 to 2717.322 (for blood isolate), 70.056 to 3284.688 (for pus isolate), and 84.068 to 2869.542 (for urine isolate) for proteins identified using MASCOT analysis. All the peaks were observed with high signal to noise ratio (S/N > 3) and were labeled based on the most intense peak observed in their isotopic distribution. No peaks were observed at higher masses. Twenty positive hits were observed when the MS/MS data were searched using MASCOT analysis in SwissProt database (2018_06 with 557713 sequences; 200130199 residues) where protein scores greater than 68 were considered significant (*p* < 0.05). Protein score is -10^∗^log (*P*), where *P* is the probability that the observed match is a random event. This search resulted in various proteins in isolate dependent manner. For instance, in stool isolate, the search revealed four proteins: EFTU1_ECO24, EFTU2_ECO24, EFTU1_SHIF8, and EFTU2_SHIF8 corresponding to Elongation factor Tu 1 and Tu 2 of *E. coli* and *Shigella flexneri*. The MS/MS data from other peaks did not yield a sufficient score to generate any successful matches in the protein database. Table 3 summarizes the results, revealing the identity of different proteins found in the common band of the biofilm matrix protein using the bottom up proteomic approach. Peptide sequences were only assigned when they displayed a MASCOT score above 68 which was taken to indicate identity or extensive homology (*p* < 0.05). Multiple peaks between m/z 69.812 and 3284.688 were observed which might be associated with trypsin digested peptides. However, majority of these peaks displayed low MASCOT scores and sequence were not assigned due to lacked correlation with any protein in the database (Supplementary Data S9, S10). Among the several proteins identified by MALDI MS/MS were elongation factor Tu 1, elongation factor Tu 2 (in stool isolate); dihydrolipoyl dehydrogenase, 3-phosphoshikimate 1-carboxyvinyltransferase, phosphoglucosamine mutase, conjugal transfer protein TraA, DNA-directed RNA polymerase subunit beta, putrescine aminotransferase, 50S ribosomal protein L9, 50S ribosomal protein L10, elongation factor P-(R)-beta-lysine ligase, translation initiation factor IF-2 (in blood isolate); elongation factor Tu, citrate synthase, chaperone protein DnaK, NADP-dependent isopropanol dehydrogenase, DNA topoisomerase 1, chaperone protein HscA homolog, 2-isopropylmalate synthase, protein translocase subunit SecA, DNA-directed RNA polymerase subunit alpha, gamma-glutamyl phosphate reductase (in pus isolate); transketolase 1, 30S ribosomal protein S1, ATP synthase gamma chain, 1-deoxy-D-xylulose 5-phosphate reductoisomerase, GTPase Era, argininosuccinate synthase, inositol 2-dehydrogenase, urease accessory protein UreG, catabolite control protein A, acetate kinase (in urine isolate). Besides, a huge number of peaks remained unannotated signifying the presence of undocumented proteins/peptides in its biofilm which needs further investigation.

**FIGURE 6:**
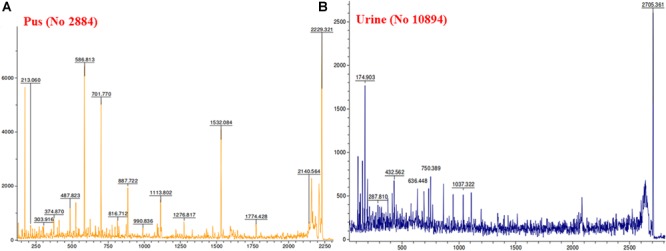
MALDI MS/MS data for the trypsin digested common protein bands. After subsequent digestion and extraction, 1 μl of the peptide mixture was mixed with 1 μl of α-cyano-hydroxycinnamic acid matrix solution together with a lock mass peptide and from this mixture, 1 μl was spotted on the target plate and analyzed by MALDI MS/MS for m/z ranging from 65 to 2,800. **(A)** Data obtained from pus isolate while **(B)** was representative of urine isolate.

**Table 3:** Functional proteins detected from the single common protein band isolated through 1D SDS-PAGE of biofilm matrix of *Klebsiella pneumoniae.*

Group and function	Protein	Accession no.	Mass	Score
**Protein synthesis and processing**	Elongation factor Tu 1	EFTU1_ECO24	43427	138
		EFTU1_ECOHS	43427	138
		EFTU1_SHIF8	43426	138
		EFTU1_SHISS	43427	138
	Elongation factor Tu 2	EFTU2_ECO24	43456	138
		EFTU2_ECOHS	43457	138
		EFTU2_SHIF8	43457	138
		EFTU2_SHISS	43457	138
	Elongation factor Tu	EFTU_ECO57	43457	138
	50S ribosomal protein L9	RL9_PARXL	16075	57
	50S ribosomal protein L10	RL10_THIDA	18640	56
	Elongation factor P–(R)-beta-lysine ligase	EPMA_SALAR	37284	55
	Translation initiation factor IF-2	IF2_BACFN	112675	55
	Elongation factor Tu	EFTU_KLEP7	43390	123
	Elongation factor Tu 1	EFTU1_HAEI8	43384	112
	Elongation factor Tu	EFTU_AERHH	43525	94
	Elongation factor Tu	EFTU_COXBN	43613	88
	Elongation factor Tu	EFTU_MARHV	44034	87
	Chaperone protein DnaK	DNAK_BRADU	68364	76
	Chaperone protein HscA homolog	HSCA_AROAE	66854	74
	Protein translocase subunit SecA	SECA_PROM4	107505	71
	Elongation factor Tu	EFTU_HERAU	43768	69
	Elongation factor Tu 1	EFTU1_HALHL	43283	69
	Elongation factor Tu 2	EFTU2_HALHL	43269	69
	30S ribosomal protein S1	RS1_DICD3	61334	63
	30S ribosomal protein S1	RS1_ECO57	61235	63
	30S ribosomal protein S1	RS1_SHIFL	61235	63
**Energy metabolism**	ATP synthase gamma chain	ATPG_BACCA	32323	61
	Transketolase 1	TKT1_ECOLI	72451	75
	Dihydrolipoyl dehydrogenase	DLDH_ECO57	50942	191
	3-Phosphoshikimate 1-carboxyvinyltransferase	AROA_BACLD	45722	78
	Citrate synthase	CISY_SALTY	48474	96
	NADP-dependent isopropanol dehydrogenase	ADH_THEBR	37851	75
	2-Isopropylmalate synthase (Fragment)	LEU1_BUCUN	56434	72
	Gamma-glutamyl phosphate reductase	PROA_BRUSU	44300	69
**Capsule production and cell wall**	Phosphoglucosamine mutase	GLMM_COXBN	48301	69
	Phosphoglucosamine mutase	GLMM_COXB1	48347	63
	Putrescine aminotransferase	PAT_SALAR	50192	61
	Putrescine aminotransferase	PAT_SALPB	50078	61
**Virulence factors**	1-Deoxy-D-xylulose 5-phosphate reductoisomerase	DXR_CLOBM	43041	57
	1-Deoxy-D-xylulose 5-phosphate reductoisomerase	DXR_CLOB6	43168	56
	1-Deoxy-D-xylulose 5-phosphate reductoisomerase	DXR_CLOBJ	43182	56
	1-Deoxy-D-xylulose 5-phosphate reductoisomerase	DXR_CLOBL	43212	56
	1-Deoxy-D-xylulose 5-phosphate reductoisomerase	DXR_CLOBK	43169	55
	GTPase Era	ERA_LACH4	34027	54
	Argininosuccinate synthase	ASSY_SOLUE	50458	54
	Inositol 2-dehydrogenase	IOLG_RUBXD	37446	54
	Urease accessory protein UreG	UREG_RHOPB	22253	53
	Acetate kinase	ACKA_PORG3	43586	52
	Conjugal transfer protein TraA	TRAA_RHIRD	123705	61
	Putrescine aminotransferase	PAT_SALAR	50192	61
	Putrescine aminotransferase	PAT_SALPB	50078	61
	Gamma-glutamyl phosphate reductase	PROA_BRUSU	44300	69
**Regulation and DNA binding**	DNA topoisomerase 1	TOP1_STAS1	79764	75
**Transcription**	DNA-directed RNA polymerase subunit beta	RPOC2_SYNSC	148515	61
	DNA-directed RNA polymerase subunit beta	RPOC2_PROMS	150243	59
	DNA-directed RNA polymerase subunit beta	RPOC2_PROM0	150258	57
	DNA-directed RNA polymerase subunit alpha	RPOA_PROM1	34244	71
	Catabolite control protein A	CCPA_STAEQ	36500	53
	DNA-directed RNA polymerase subunit beta	RPOC_PSYCK	155806	52

### Quantification of Biofilm Matrix Contents

#### Protein Estimation

The average amount of protein obtained from the biofilm of high slime producing representative isolates as estimated by Bradford method was found to be 525.2, 309.1, and 265.6 μg/ml for blood (isolate no. 1739), stool (isolate no. 197), and pus (isolate no. 2884) isolates, respectively. However, the protein content in representative urine isolate (isolate no. 10894) was comparatively lower than other isolates (246.1 μg/ml). The means and standard deviations of triplicate determinations can be seen in Table 4, Supplementary Table S5 and Supplementary Data S7.

**Table 4:** Sugar (phenol–sulfuric acid method), uronic acid (sulfamic acid–carbazole method), protein (Bradford’s assay) and acetyl content estimation of biofilm matrix.

Isolate		Sugar content	Protein content	Uronic acid content	Acetyl content	Acetyl as percent of	Acetyl as percent of
number	Source	(μg/ml)	(μg/ml)	(μg/ml)	(μg/ml)	total uronate	total sugar
2884	Pus	1445.995 ± 15.583	265.6 ± 3.786	307.6 ± 6.849	28.42 ± 4.214	9.24%	1.96%
10894	Urine	1616.088 ± 11.28	246.1 ± 1.651	316.9 ± 3.687	21.675 ± 9.165	6.84%	1.34%
197	Stool	717.0753 ± 12.65	309.1 ± 3.055	325.3 ± 33.58	20.16 ± 16.582	6.2%	2.51%
1739	Blood	2257.207 ± 14.791	525.2 ± 2.082	251.5 ± 4.347	28.36 ± 7.327	11.28%	1.26%

#### Sugar Estimation

Total sugar content in the extracted biofilm matrix was quantified by the phenol–sulfuric acid method. All the representative isolates showed the presence of sugars. However, the isolate no. 1739 showed the highest amount of sugar (2,257.207 μg/ml) in its biofilm matrix. The means and standard deviations of triplicate determinations can be seen in Table 4 and Supplementary Data S8.

#### Uronic Acid Estimation

**FIGURE 7:**
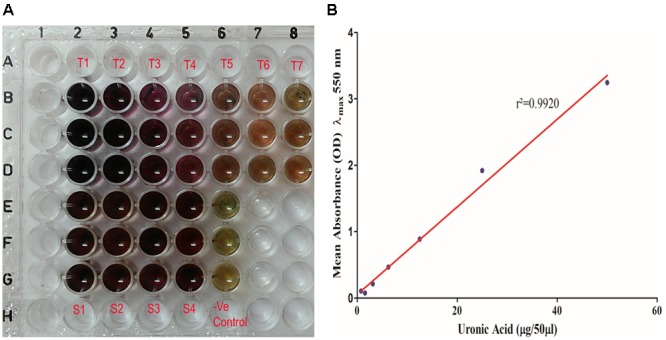
**(A)** Uronic acid estimation by modified sulfamic acid–carbazole method. (T1–T7) Galacturonic acid as the standard in concentration range of 200–1.562 μg/well in triplicate in parallel lanes from B to D. (S1–S4) 50 μl of extracted sugar fractions of the biofilm matrices of different isolates in triplicate in parallel lanes E–H. **(B)** Standard plot of galacturonic acid showing linear correlation between absorbance at λ_max_ 550 nm and amount of uronic acid with correlation coefficient *r*^2^= 0.9920.

Total uronic acid content in the extracted biofilm matrix was quantified by the modified sulfamic acid–carbazole method (Figure 7). All the representative isolates showed the presence of acidic sugars. However, the isolate no. 197 showed the highest amount of uronic acid content (325.3 ± 33.58 μg/ml) in its biofilm matrix. Strikingly, the pus isolate has more uronic acid content (307.6 ± 6.849 μg/ml) compared to the blood isolate (251.5 ± 4.347 μg/ml). The means and standard deviations of triplicate determinations can be seen in Table 4.

#### Acetyl Group Estimation

**FIGURE 8:**
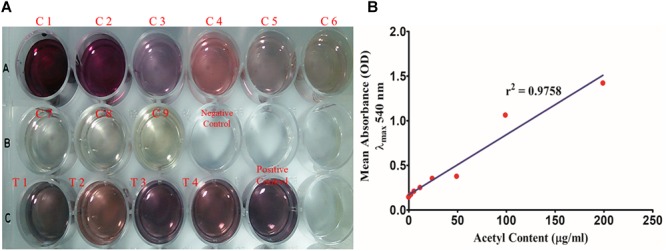
**(A)** Total acetyl content of the biofilm matrix. (C1–C9) Glucose pentaacetate as the standard in concentration range of 200–1.562 μg/well. (T1–T4) 250 μl of the test samples of the biofilm matrices of different isolates were utilized for the study. **(B)** Standard plot of glucose pentaacetate showing linear correlation between absorbance at λ_max_ 540 nm and total acetyl content with correlation coefficient *r*^2^= 0.9758.

Total acetyl content in the extracted sugar part of biofilm matrix was quantified by the method proposed by Hestrin with minor modification (Figure 8). All the representative isolates showed the presence of acetyl group. However, the isolate no. 2884 (pus isolate) showed the highest amount of acetyl content (28.42 ± 4.214 μg/ml) in its biofilm matrix. Strikingly, the blood isolate 1739 had more acetyl content corresponding to the percent molar content of the total uronates present (11.28%) compared to the blood isolate (251.5 ± 4.347 μg/ml). The molar ratio of acetyl: uronates was found to vary in the range of 0.092–0.113. The means and standard deviations of triplicate determinations can be seen in Table 4.

#### *e*DNA Estimation

Extracellular DNAs in the extracted ECM were purified and quantified using NanoDrop 2000 and then 1.5% agarose gel was run to check its tentative molecular weight and band width (Supplementary Figure S11). The means and standard deviations of triplicate determinations are summed up in Supplementary Table S6.

### Confocal Laser Scanning Microscopic (CLSM) Evaluation of Chemical Contents of Biofilm Matrix

**FIGURE 9:**
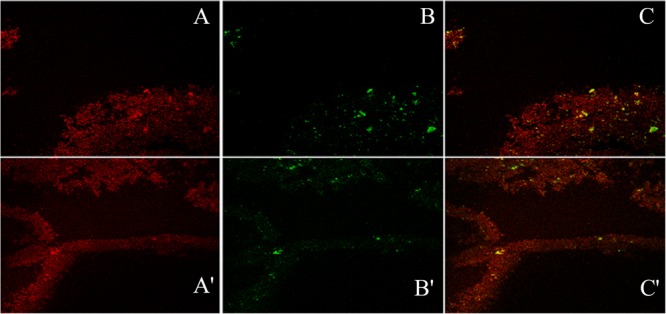
The confocal micrographs of the tetramethyl-rhodamine isothiocyanate (TRITC) labeled mannose-specific Concanavalin A lectin used to stain biofilm exopolysaccharide (red) and phalloidin green staining the matrix amyloid proteins (green). The square panels depict planar view looking down on the biofilm. **(A,A′)** Extracellular red staining of the exopolysaccharide by ConA-TRITC can be seen on 72 h old biofilms of *K. pneumoniae*. Application of the TRITC labeled ConA resulted in the cloudy appearance as seen in **(A,A′)** regions. **(B,B′)** Confocal image of extracellular green staining of the exopolysaccharide by phalloidin green of 72 h old biofilms of *K. pneumoniae*. **(C,C′)** The merged view of the panels **(A,B)** and **(A′,B′)**. The yellow signals are due to the exact overlap of ConA-TRITC with the phalloidin green. The thickness of the biofilm is 48 μm. Note the exact overlap of green fluorescence with the red one indicating the presence of proteins/amyloids amidst the biofilm matrix entwined with the sugar.

Confocal microscopic examination of mature biofilm formed after 72 h of growth of *K. pneumoniae* isolates no. 1739 (representative high slime producing blood isolate) was performed using the combination of fluorescent dyes ConA-TRITC (Concanavalin A labeled with tetramethyl-rhodamine isothiocyanate) and phalloidin green to assess the differential distribution of sugars and proteins in its biofilms. In agreement with the data obtained by various biochemical and spectrophotometric analysis, the biofilm of the isolates no. 1739 showed an intense ConA staining, indicating the presence of a markedly high amount of the sugars. However, the phalloidin staining was not as intense as ConA. Representative confocal micrographs of biofilms are shown in Figure 9. Of note, the phalloidin stain was found to retrace the same path as that of ConA, indicating the distribution of proteins in close proximity of the sugars as evident from the panels A, A′ and B, B′ of Figure 9. In panels from C, C′ of Figure 9, one can note the widely dispersed yellow signals coming from the matrix, showing the superimposition of red and green signals indicating juxtaposition/co-localization of sugars and proteins in the biofilm matrix. Images reveal high order of heterogeneity in *K. pneumoniae* mature biofilm architecture in terms of the distribution of sugars (indicated by red color) and proteins (green color).

**FIGURE 10:**
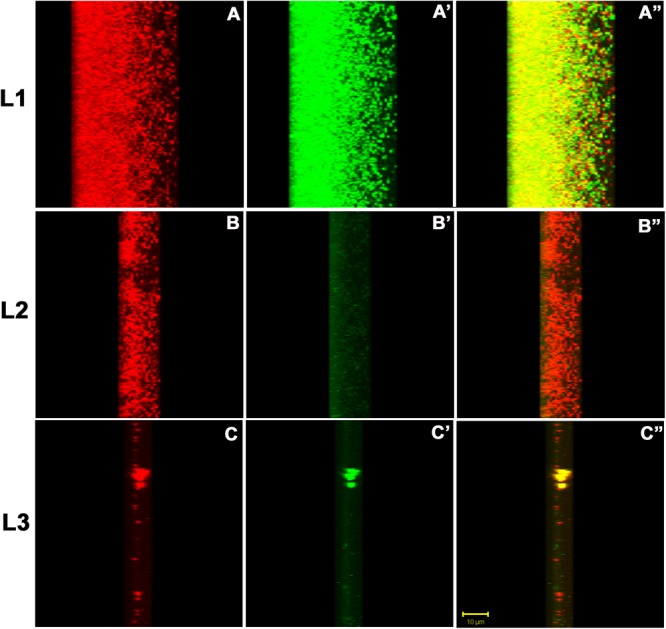
The confocal micrographs of the transverse sections of TRITC-labeled Concanavalin A and phalloidin green staining of the biofilm matrix. The square panels shown here is the view of the thickness (*Z*-axis) of the biofilm. The sections are rotated 22° to get a thorough transverse cross sectional overview. **(A–C)** One can see extracellular red staining of the exopolysaccharide by ConA-TRITC on 72 h old biofilms of *K. pneumoniae*. **(A′–C′)** Confocal image of extracellular green staining of the exopolysaccharide by Phalloidin green of 72 h old biofilms of *K. pneumoniae*. **(A″–C″**) The merged view of the panels **(A,A′), (B,B′)**, and **(C,C′)**. The yellow signals represent the juxtapositioning of ConA-TRITC and phalloidin green that indicates the presence of sugars and proteins in close proximity. The thickness of the biofilm is 48 μm. Note the green fluorescence amidst the red ones indicating the role of proteins/amyloids as the inevitable building block of biofilm matrix. The results shown here is in agreement with the matrix dissolution assay. Unstained regions or darker voids of the biofilms may be due to water-channels or non-binding of the ConA and/or phalloidin.

**FIGURE 11:**
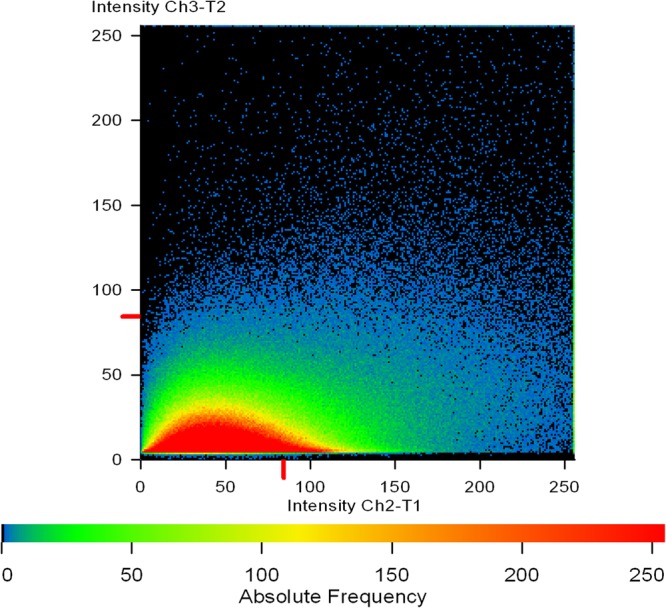
A co-localization map of TRITC-labeled Concanavalin A (red) and phalloidin (green). Extracellular mannose/glucose is stained red by ConA-TRITC while extracellular green staining is of proteins/amyloids present in the matrix. The intermediary yellow signals represent the co-localization of ConA-TRITC and phalloidin green, indicating the entangling of sugars and proteins. One can note the linearity of the intensity on *X*-axis. The intensity of green signal is increasing with the increase in red signal.

For finer details, we further investigated the transverse sections of biofilm matrix accompanied by analyzing co-localization maps which attested the impregnation of proteins in the sugar rich slime. The confocal transverse sectioning of the biofilm matrix in panels A″–C″ of Figure 10 clearly illustrates the entwined nature of sugars and proteins. As we changed the angle of analysis from panels L1 to L3, we can see the prominence of red signals indicating the preponderance of sugars in the biofilm. However, the green signals are also coming, although faint, are the suggestive of existence of proteins. In co-localization map shown in Figure 11, one can see the exact retrace of red signal by the green one. The overlap of signals have emerged in yellow appearance in the mid-way region. This map illustrates the linearity in the signals across the biofilm matrix.

## Discussion

Biofilm matrices vary in their composition and chemistry from bacteria to bacteria. For instance, *S. aureus/S. epidermidis* biofilm consists mainly of poly-*N*-acetyl glucosamine (PNAG) whereas in methicillin-resistant *S. aureus* (MRSA) PNAG-independent proteinaceous biofilms are also reported (Cramton et al., 2001; Fitzpatrick et al., 2005; O’Gara, 2007; Speziale et al., 2014). *P. aeruginosa* mainly consists of alginate whereas *E. coli* mainly contains curli proteins (Vu et al., 2009; Donot et al., 2012; Lembre et al., 2012; Andersson et al., 2013). The reports suggest *K. pneumoniae* biofilm consisting largely of polysaccharides (Sutherland, 2001; Vu et al., 2009; Donot et al., 2012; Lembre et al., 2012; Cescutti et al., 2016; Bellich et al., 2018).

One of objectives of the present work was to ascertain whether there is any variation in the biofilm forming ability/pattern among clinical isolates of *K. pneumoniae* obtained from different clinical specimens under varying concentrations of salt, sugar, and amino acids. Another intent of this study was to extricate the chemistry of the *Klebsiella pneumoniae* biofilm matrix in detail. We tried to investigate any possible relation between infectivity archetypes of *Klebsiella* and the underlying chemistry of the biofilm matrix. Besides, in the present work, we also provided the experimental evidence that sugar is not the only/major constituent of the extracellular matrix of *Klebsiella* biofilms.

Two hundred fifty-seven clinical isolates from various sources were reconnoitered for their adeptness to form biofilm. Nutrient richness influences biofilm formation. No significant difference was observed in biofilm forming abilities of the isolates while using BHI broth or LB broth, however, the average absorbance in BHI was found to be comparatively higher. Fredriksson and Nyström (2006) had reported that in less nutrient medium bacteria enters “conditional senescence” state whereby stationary phase cells are more likely to accumulate damaged proteins and hence either die or exhibit reduced fitness. BHI broth teems with essential amino acids, prerequisite for the formation of fimbriae and pili which are indispensable for primary adherence (Singh et al., 2017a). Therefore, in the present study, BHI broth was accepted as the medium of choice.

Chemical fixation by sodium acetate (2% w/v) was found to be more useful and efficient compared to heat fixation at 60°C for 20 min in our present study, unlike *S. aureus* isolates where heat fixation was a method of choice (Singh et al., 2017a). Heat disrupts hydrogen bonds, and dries out the sugar content and even chars some, leading to the flawful biomass estimation of *K. pneumoniae* biofilms. Therefore, we preferred chemical fixation.

When 444 mM glucose, 684 mM NaCl, 8 mM methionine, 15 mM *S*-adenosyl methionine were added as supplement mix, the upsurge in absorbance was highly significant (*P* ≤ 0.001). Of note, supplement mix has significantly escalated the absorbance regardless of the sources whether it is urine, pus, blood, or stool isolates (*P* ≤ 0.001). Therefore, the proposed method for *in vitro* biofilm quantitation of *K. pneumoniae* can be employed for biofilm study of *Klebsiella* isolates. The supplementation seems to stimulate the bacterial cells to produce more slime. Particularly, blood-isolates formed the highest-density biofilms after the addition of supplement mix at 37°C. Conversely, some pus isolates were found to be innately high slime producer at 37°C even without supplementation. The similar trend was observed with some of the blood and urine-isolates. These contrasting observations signal about the involvement of different mechanisms in the augmentation of biofilm biogenesis.

In the present study, all the *K. pneumoniae* isolates had shown considerably greater adherence in supplemented BHI broth. However, a few isolates retained their absorbance indices even after supplementation same as in unsupplemented BHI broth. This stifled any possibility of generalization of universal optimal condition for *in vitro* biofilm formation by *K. pneumoniae* clinical isolates. In the present study, we identified 30 isolates, which can be considered adherent only after supplementation. The situation became more complex in case of two isolates which were found to be non-adherent in either of the conditions. Majority of isolates (*n* = 224) were found adherent even without supplementation although, the more pronounced adherence was after supplementation. Almost one-fourth of the total isolates (*n* = 72) were found to be weakly adherent even after supplementation. This tally is inclusive of 30 non-former (earlier described) isolates. Not all frank pathogenic isolates of *K. pneumoniae*, which were obtained from blood, pus, urine, had high adherence indices. Conversely, some stool isolates (*n* = 22), where these are considered as commensal, were found to be extremely adherent. When we average out the absorbances of these stool isolates with other isolates, it may bias or skew the data, ultimately resulting in a false positive impression that all the isolates collected from different sources as a whole were highly adherent. Furthermore, it will obfuscate the interpretation of any criteria for exact discrimination between adherent and non-adherent isolates. To preclude this altered pattern and observation of expansion of the limit of absorbance in previously reported non-, weak, moderate, and HBF category; we perceived a need of the introduction of a new cut-off/classification criteria. It may be possible that these highly adherent stool isolates may prove to pathogenic; which once get access to the favorable susceptible site, invade the host and cause disease.

However, till date, there is no accord among investigators regarding biofilm quantitation and categorization of *K. pneumoniae* clinical isolates. Therefore, the rationale of deciding strong, medium, weak, and non-biofilm producers varies tremendously among the studies. Maldonado et al. (2007) have classified the isolates as high slime producers when the absorbance was greater than 0.5, moderate producers when the absorbance ranged between 0.5 and 0.1 or poor producers when absorbance was less than 0.1. While other investigators have not accurately defined the rationale of employing this criterion (Maldonado et al., 2007). It is therefore prudent to elucidate the consensus guidelines regarding quantitation and categorization.

We settled these problems by incorporating all the data of absorbances in both supplemented and unsupplemented conditions in BHI into a single scalar value (the value not influenced by any variable independently) to define a negative control index which was the measure of adherence for the negative control and then, a cut off OD was defined as OD_cut_ = OD_avg_ of negative control index + 3 × SD of ODs of negative control index. This indexing and definition of cutoff value were found to be more accurate in determining the precise cutoff rather than the broth or ATCC 12228 alone. Broth can be utilized to ensure the sterility during the execution of the experiment (Singh et al., 2017a). By adopting the proposed method and criteria, it was noted that reference strains ATCC 35984, 35983, and 12228 remained in their respective classes as high, medium, and non-formers both with and without supplementation. However, interestingly, when the new criterion was tested on all the other clinical isolates of *K. pneumoniae*, all the previously declared non-former/weak former isolates were either relocated to the moderate/potentiated moderate former or to the high-former category. Similarly, some of the moderate and high formers were turned to super former category. Therefore, instead of using either uninoculated broth or ATCC 12228 as a negative control, a cut-off was determined by specifying negative control index for error-free and concordant results.

The second noteworthy result of par medical implication pertaining to this work is the elucidation of the remarkably heterogeneous nature of the *K. pneumoniae* biofilm matrix in terms of its chemistry. This was initially checked by chemical disintegration and enzymatic dissolution of the slime. However, for delicate details, we used FTIR spectroscopy, NMR spectroscopy, CLSM, SDS-PAGE, HPLC, and MALDI MS/MS analysis. The results obtained by these analyses demonstrate the role of different biomolecules in constructing biofilm matrix architecture.

The initial screening of the biofilm matrix composition was made using various chemicals and enzyme. The results suggest the presence of protein, sugar and *e*DNA in the biofilm matrices of all the *K. pneumoniae* isolates. However, the percentage abundance was found to vary among isolates affecting different sites of infections. Proteinase K mediated degradation was more pronounced in the biofilms of blood and pus isolates which indicates the presence of considerable amount of proteins. Similarly, sodium metaperiodate (NaIO_4_) mediated digestion was significantly marked in urine isolates which indicates more of sugars in the matrix compared to proteins. Of note, urine isolates were relatively inert toward proteinase K degradation. However, the blood and pus isolates were found to be comparable in terms of degradation mediated by NaIO_4_ and proteinase K.

This initial screening was followed by extraction of biofilm matrix to characterize and quantitate its diverse constituents like protein, sugars and extra cellular nucleic acids. From the extracted biofilm matrix, we separated both the protein and sugar contents. The sugar content was then evaluated by FTIR, NMR. However, our primary focus was on protein extract which was further evaluated by SDS-PAGE, HPLC, and MALDI MS/MS analysis.

FTIR spectroscopy is a rapid and non-destructive technique which is based on the principle that atoms in molecules are loosely held and when subjected to infrared radiation (between 300 and 4,000 cm^-1^), the molecule absorbs energy and the bond undergoes a number of different vibrations (Schmitt and Flemming, 1998). Therefore, the FTIR spectrum contains information pertaining to the molecular structure of the sample. Recently it has been used for biofilm investigations (Tugarova et al., 2017). The FTIR spectral profile obtained in mid and far-infrared regions (500–1,600 cm^-1^) revealed the absorption of sugars present in the *K. pneumoniae* biofilm matrix. The spectral bands obtained are mainly associated with glucans (973.73 cm^-1^), β (1 → 4) linkage (862.69 cm^-1^), β (1 → 3) linkage (1,053.25 cm^-1^, 1,020.51 cm^-1^) and mannans at 862.69 and 915.42 cm^-1^. Asymmetric carboxylate stretching at 1,628.38 cm^-1^ points out the presence of sugar acids. Presence of acetyl group bands at 1,207.18 and 1,250.25 cm^-1^ specifies the existence of 2′ acetylation which ultimately reveals the presence of acetylated uronic acids. Besides, C–N stretchings at 1,289.82 cm^-1^ is the suggestive of primary amine which implicates the presence of amino sugar(s) as well. Amidst all the sugar bands, we noticed a broadband of proteins (amide II) at 1,550.62 cm^-1^ (Lal et al., 2010). The presence of carboxyl groups may confer some adaptive advantages with regard to the sequestration of divalent cations for their need. The carboxyl group may also serve as functional moieties to generate new or modified polymers which may promote bacterial pathogenesis or virulence or both (Kives et al., 2006; Baum et al., 2009).

The 1D ^1^H-NMR spectra of *K. pneumoniae* biofilm matrix in D_2_O showed two distinguishable groups of signals (non-anomeric protons and anomeric protons). Investigation of anomeric proton (H1) signals in this region indicated the presence of pyranose ring in α-or β-configuration. Based on NMR spectra of carbohydrates, these peaks (4.944, 4.995, 5.017, 5.041, and 5.196 ppm) were assigned to α- and β-D-glucose, α-D-mannose and α-l-rhamnose sugars. The presence of *N*-acetyl glucosamine (β-D-GlcNAc) was also detected at 1.884 ppm in the ^1^H spectrum. The ^13^C-NMR data of *K. pneumoniae* biofilm matrix showed 23 carbon signals (Mayer et al., 2001). The chemical shifts indicated the presence of α-l-rhamnose, α- and β-D-glucose in the exopolysaccharide chain. The exopolysaccharide structure of *K. pneumoniae* was found to be somewhat similar to the exopolysaccharide formed by *Staphylococcus aureus*. Both the micro-organisms are important biofilm colonizers on medically implanted devices and their exopolysaccharides contain glucose and hexosamine as major sugar units. Although, investigators have utilized ^1^H and ^13^C NMR spectroscopy for structural determination of cell wall components in *C. albicans*, however, in the case of chemical analysis of *K. pneumoniae* biofilm matrix components this has not been utilized earlier (Lowman et al., 2003).

Only a few attempts have been made to separate and characterize the exopolysaccharide associated with *Klebsiella* biofilm. Recent report proposed that *Klebsiella* biofilm contained almost 60% mannose, 20% galactose, and 17% galacturonic acid (Verhoef et al., 2005). Some investigators have reported the presence of colonic acid as well (Rättö et al., 2006). A marked disagreement among sugar composition of four types of *K. pneumoniae* isolates was observed in our present study. While all four isolates had high mannose content, the next most abundant carbohydrates present in blood and urine isolates (1739 and 10894) were rhamnose, galactose, and glucosamine. However, for pus isolate 2884, the abundance of glucose, rhamnose, and galactosamine were observed in the order of importance. However, we cannot confirm whether these variations are owing to the differences in extraction/purification procedures, or simply it represents natural diversity and selection depending upon the site of infection, ending up in heterogeneity in composition of the biofilm matrices within a species as noted among the four *K. pneumoniae* isolates in the present study. Therefore, the present study of the *K. pneumoniae* exopolysaccharide has revealed the unique composition consisting of glucans, mannans, uronate(s) and sugar amine(s) along with substantial amount of proteins and *e*DNA (Cescutti et al., 2016).

Besides biophysical qualitative characterizations of biofilm matrix sugars, we also quantitated the sugars in the present study. Like Knutson and Jeanes (1968), we also noted that at lower temperature, interference by the brown color produced by neutral sugars are minimized. Therefore, in the current study, we chose the temperature as low as 80°C. Another modification was the addition of the sulfamic acid reagent and the use of 75 mM disodium tetraborate reagent along with carbazole in estimation of uronic acids. Addition of tetraborate ions to the carbazole reagent increased the sensitivity for the detection of certain uronic acids. The presence of acetyl content corresponding to the percentage of total uronates was found to be around 11% and 9% in case of blood and pus isolates, respectively. These quantitative results of the biochemical investigations are in agreement with FTIR data. The richness of 2′ acetylated uronates in the biofilm matrix of the blood and pus isolates may bestow them some adaptive advantages with regard to the sequestration of cations, maintenance of biofilm polarity and hence hydrophilicity as per their need. These acetylated uronates may act as precursors of molecules needed to acclimatize in diverse environments as well as to show its virulence (Wilkinson, 1958; Sutherland, 2001; Mahmoud and George, 2004; Kives et al., 2006; Bowen et al., 2017). Therefore, in this study, a lectin-based approach was adopted to recognize specific sugars present in the biofilm matrices of different isolates. ConA is one of the most widely used lectins in biofilm research because of its ability to bind with alpha-linked mannose residues. Similarly, green phalloidin binds to the matrix amyloid proteins (Neu et al., 2001, 2010; Strathmann et al., 2002). We explored the binding of the ConA-TITC and green phalloidin to the *Klebsiella* biofilms. Figure 7, 8 show intense red fluorescence emanating from ConA-TITC, the green fluorescence owing to phalloidin, while yellow signal is the result of overlapping of red and green domains presumably due to the juxtaposition of sugars and proteins. The amyloids are proteins with a capacity to fold into a cross-beta structure and to polymerize into insoluble fibers (Larsen et al., 2007). We looked for amyloids in *K. pneumoniae* biofilms due to difficulties in complete degradation of the *Klebsiella* biofilm by proteinase K digestion and the mocktails containing proteinase K (although the stool isolate 197 was having minimal sugar and predominantly protein). Reports indicate that bacteria purposely produce amyloids as they are resilient to degradation by proteases and they contribute to the structural integrity of biofilms (Larsen et al., 2007). Unstained regions of the biofilms may be due to water-channels or non-binding of the lectin and phalloidin. Uneven distribution of ConA-TITC and green phalloidin seems to be a typical feature of *Klebsiella* aggregates/biofilms. However, CLSM and other techniques have suggested that *K. pneumoniae* extracellular matrix is not predominantly comprised of sugars like mannose, glucose, their amines and acetylated counterparts; rather it also consists of the proteins in considerable amounts. This indicates the indispensable role of proteins in matrix structuring in *Klebsiella pneumoniae* isolates. The earlier notion of “sugar only” biofilm of *Klebsiella pneumoniae* was a gross oversimplification.

The presence of appreciable amount of proteins in the biofilm matrix made us inquisitive about its quantification. The highest and lowest amount of proteins were detected in the biofilms of blood and urine isolates, respectively. The extracted proteins from the biofilm matrices of the four isolates were then subjected to SDS-PAGE analysis which revealed various common as well as unique protein bands. Common protein bands may indicate the main structuring protein motif of the biofilm which is probably present in all *Klebsiella* biofilms irrespective of their sites of isolation, however; the unique bands may be indicative of the environment/site specific expression of the proteins. The evaluation of common protein bands can form a lead/basis of the diagnosis/drug/vaccine development against biofilm associated *K. pneumoniae* infections. Taking this into account, we excised the common high molecular weight protein band (35 kDa) and looked for the polarity profile by analytical HPLC. We identified two different independent peaks for different representative isolates (based on their site of infection and isolation) with different height/area but almost the same retention time. The set experimental conditions and the observed results show the presence of some amphipathic nature of the proteins/peptides which was later on confirmed by MALDI MS/MS analysis. MALDI MS/MS results of the single common protein band indicated the differential expression of different proteins within the biofilm due to heterogeneous environments. The 55 different proteins were identified among various *Klebsiella* isolates which include both cytosolic and membrane proteins. About 22 proteins were identified with varied similarities related to protein synthesis and processing while 15 proteins were identified to play important role in virulence. Similarly, proteins related to energy and metabolism were 8 and those related to capsule and cell wall synthesis were 4. Several of these identified proteins have ‘moonlighting’ or diverse functional associations. For instance, the enzymes like phosphoglucosamine mutase and putrescine aminotransferase are reported for their role partly in capsule and cell wall synthesis and partly in interactions with the host which includes adaptive responses to environmental changes, adherence, and even host immune evasion. Phosphoglucomutase is involved in UDP-GlcNAc turnover cascade which is one of the main cytoplasmic precursors of bacterial cell-wall peptidoglycan. Besides, in Gram-negative bacteria, it is the precursor of outer membrane lipopolysaccharide, and triggers the synthesis of the enterobacterial common antigen (Rabin et al., 2015). In bacteria, polyamine turnover is maintained by putrescine aminotransferases. Putrescine and spermidine are reported to be involved in bacterial differentiation and signaling which in turn, coordinate multicellular actions (Shah and Swiatlo, 2008). Similarly, urease is multifunctional. In case of *H. pylori*, urease functions as an environmental modulator, while in *S. aureus* and *K. pneumoniae*; it is reported to hydrolyze urea to carbonic acid/bicarbonate and ammonia/ammonium, which buffers any acidic pH in the proximity of the bacterium. Several urease accessory proteins (like UreG) are found essential for incorporation of nickel into the active center of urease (Li et al., 2014). Besides, role of urease in colonization is also much advocated (Li et al., 2014). Like urease, acetate kinase also plays key role in the virulence behavior of several bacteria. Acetate kinase in conjunction with phosphate acetyltransferase controls acetyl phosphate levels in bacteria. It converts acetate to acetyl phosphate which is subsequently converted to acetyl-CoA. Acetyl-CoA is then utilized by the mevalonate pathway for peptidoglycan biosynthesis (Ingram-Smith et al., 2006). Another enzyme is 1-deoxy-D-xylulose 5-phosphate (DXP) reductoisomerase which is involved in the biosynthesis of isoprenoids through the mevalonate-independent DXP pathway which is present in many eubacteria, algae, and malarial parasites. In many bacteria including *P. aeruginosa*, the DXP pathway represents the only source of isopentenyl pyrophosphate (IPP), the universal precursor of isoprenoids (King et al., 2017). Transketolase links glycolysis and the pentose phosphate pathway (involved in the catabolism of pentose sugars, the formation of D-ribose-5-phosphate, D-erythrose 4-phosphate). D-erythrose-4-phosphate is a precursor of aromatic amino acids and pyridoxal phosphate (Diaz-Moralli et al., 2016). Thus, MALDI MS/MS analysis revealed the high probability of these multifunctional proteins in *Klebsiella* indicating their possible role as virulence factors. This is consistent with prior reports where investigators have noted the therapeutic failure against *Klebsiella* chronic infections (Singh et al., 2017b). A detailed study of these identified proteins in response to the effect of antimicrobial or other culturing perturbations could give a greater insight and add to the future applications of proteomics profiling of the bacterial biofilms.

The distinctive texture and composition of biofilm matrix seems to help *K. pneumoniae* to regulate biofilm density as per its need which may be modulated by the differential switching of sugar or protein or by the expression of both. The role of sugars in *K. pneumoniae* biofilms is undeniably important. However, one must consider that the onset of biofilm formation in the clinical settings starts with initial colonization which likely precludes the switch later on, to a more viscous phenotype with site-specific expression of sugars or proteins or both. Understanding the dynamic nature of *K. pneumoniae* biofilm production and regulation will shed new light on the understanding of chronic biofilms in *K. pneumoniae* pathogenesis. The most compelling complication of *Klebsiella* biofilm is its resistance toward biofilm degradation agents (Vuotto et al., 2014). A recent report of the use of curcumin quantum dots (CurQDs) as antibiofilm agent also stated the resistance of *Klebsiella* biofilms for CurQDs (Singh et al., 2017b).

The complexity of the structural motifs as revealed by CLSM and various spectrophotometric techniques suggest a highly organized assembly of the biofilm matrix and negates any *ad hoc* associations. The results obtained after thorough investigation of the biofilm matrix may help us in the control of “slimy business” of *K. pneumoniae*. In addition, specifically targeting the common structural protein motifs may also lead to vaccine development or can be utilized as a diagnostic marker. The result of the present study described the presence of an array of proteins, which may help the future investigators to identify protective epitopes and/or immunogenic determinants in order to develop a vaccine or diagnostic marker against *Klebsiella* infections, respectively.

The function of the given structure is entwined with its physical and chemical nature. The chemical composition of bacterial biofilms seems to be the function of the niche conditions wherein bacteria inhabit (Wilkinson, 1958; Sutherland, 2001; Donot et al., 2012). Therefore, bacteria growing at different sites of infection exhibit a high order of variability in their matrix compositions.

## Conclusion

*Klebsiella pneumoniae* is a human pathogen and its role in various diseases is an established fact. However, very little is known about the distribution of proteins and its association with sugars in biofilms of this organism.

The present study successfully puts forth a possible consensus protocol based on spectrophotometry for *in vitro* biofilm formation by clinical isolates of *Klebsiella pneumoniae*. The relative abundance and distribution of sugars and proteins its biofilm matrix show its diverse armament for its survival in different niches. The commonality of major protein bands is the strong evidence in favor of its pivotal role as structural building block of its biofilm. This may be investigated as the therapeutic target in the management of biofilm associated *Klebsiella pneumoniae* infections by designing better strategies for controlling its biofilm employing drugs/techniques targeting certain portions of the slime which could be the most important building block of the biofilm matrix. The methods used herein can be used to examine the spatial distribution of antibiotics and/or metabolites in the biofilms as well. Besides, we can fine tune our investigations by colocalization studies with cellular/biofilm associated proteins, providing a more complete picture of drug-biofilm interactions. These approaches may stimulate more focused research on methodologies for biofilm assembly study. In short, this study is the beginning to explore the biochemical underpinnings especially the protein(s) of the biofilm matrix in this organism.

## Author Contributions

AS and PP conceived, designed, directed, and supervised the complete study. AS, SY, BC, RS, NN, and KN performed the experiments and helped for analyzing the data obtained. JR, SS, and RKS did critical suggestions in study design, statistical analysis, and manuscript writing. AS wrote the entire manuscript which was approved by all the co-authors.

## Conflict of Interest Statement

The authors declare that the research was conducted in the absence of any commercial or financial relationships that could be construed as a potential conflict of interest.
