# Supplementary material for: Classification of Clinical Isolates of Klebsiella pneumoniae Based on Their in vitro Biofilm Forming Capabilities and Elucidation of the Biofilm Matrix Chemistry With Special Reference to the Protein Content
Source: Front Microbiol. 2019 Apr 4;10:669. doi: 10.3389/fmicb.2019.00669 (PMC6458294; doi:10.3389/fmicb.2019.00669)
Supplement: Supplementary file 1 [file Data_Sheet_1.pdf]

Classification of Clinical Isolates of *Klebsiella pneumoniae* based on its *in vitro* Biofilm forming ability and Chemistry of its Biofilm Matrix with reference to its Protein content

Ashish Kumar Singh, Shivangi Yadav, Brijesh Singh Chauhan, Nabarun Nandy, Rajan Singh, Kaushik Neogi, Jagat Kumar Roy, Saripella Srikrishna, Rakesh Kumar Singh, Pradyot Prakash

Supplementary Data Sheet

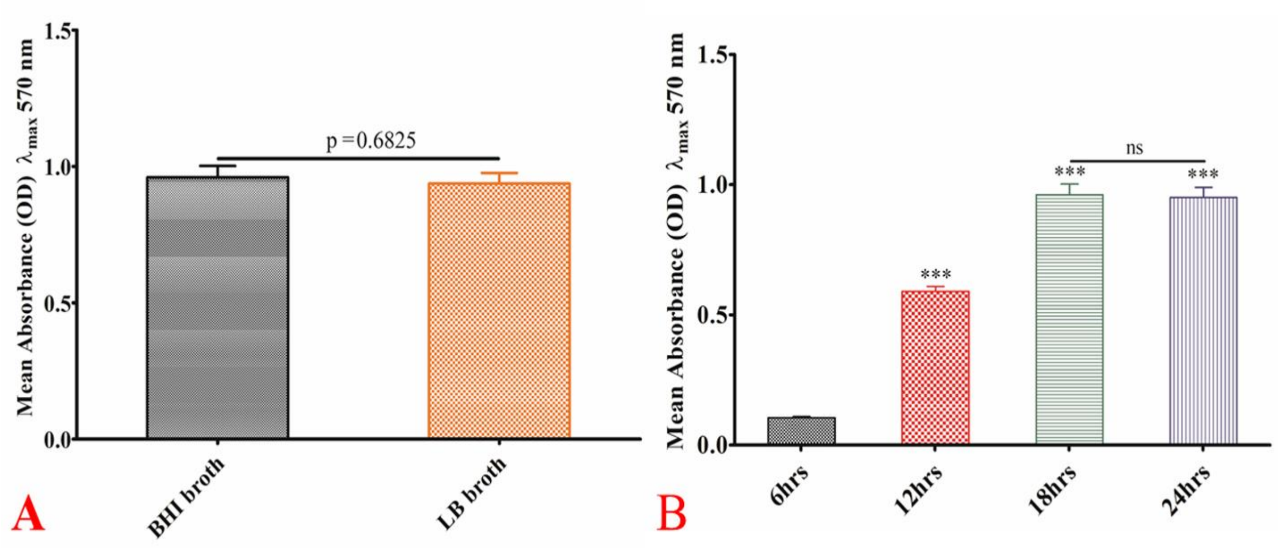

Figure S1: A. Comparison of mean absorbances of isolates grown in BHI and LB broths showing insignificant difference (P=0.6825). Data shown in Figure 1A are representative results of three independent experiments. No significant difference was observed in absorbance values when BHI broth was used as the growth medium as compared to LB (P=0.6825, P<0.05) (Supplementary data 1). However, the average absorbance values of all the clinical isolates were higher in BHI broth. For instance, the average absorbance for *S. epidermidis* ATCC 35984 was 1.957±0.028 {absorbance (OD)±SD} in BHI broth, which was 1.737% higher when compared with average absorbance in LB broth (1.923±0.019). Similarly, for *S. epidermidis* ATCC 35983, average absorbance was 0.579±0.023 in BHI broth, which was 4.3% higher when compared with average absorbance in LB broth (0.554±0.019). The results are shown as the mean ± SD of a quadruplicate assay. **B.** Comparative evaluation of the effect of incubation periods on the biofilm formation. Around 85% of the *K. pneumoniae* isolates showed detectable biofilm after 6 hrs of incubation. Bacterial adherence to the microtiter plate emerged to be a function of time till 18 hrs (with increased linearity). The increase in absorbance after 24 hrs was not significant in comparison to incubation for 18 hrs. Each strain was grown in BHI broth under static conditions at 37°C for 24 hours. Absorbance at λ_{max} 600 was measured at different time points during culture. The reference strains were found to form maximum biofilm after 18 hrs with resultant average absorbance 0.991±0.021 for ATCC 35984 and 0.102±0.017 for ATCC 12228. It was observed that after 6 hrs of incubation, the majority of the *K. pneumoniae* isolates displayed smaller absorbances with average value ranging from 0.01±0.0011 to 0.274±0.0410. After 18 hrs of incubation, all isolates were found to produce biofilms. For instance, the average absorbance for one of the isolates of K. pneumoniae (Isolate no. 2895) was 1.24±0.04, 2.254±0.02 and 2.17± 0.04 at 12 hrs, 18 hrs and 24 hrs, respectively. The identical pattern was also observed for other isolates. No significant (P≥0.05) difference in absorbance was observed after 24 hrs of incubation compared to 18 hrs of incubation (Supplementary data 2). The results are shown as the mean ± SD of a quadruplicate assay.

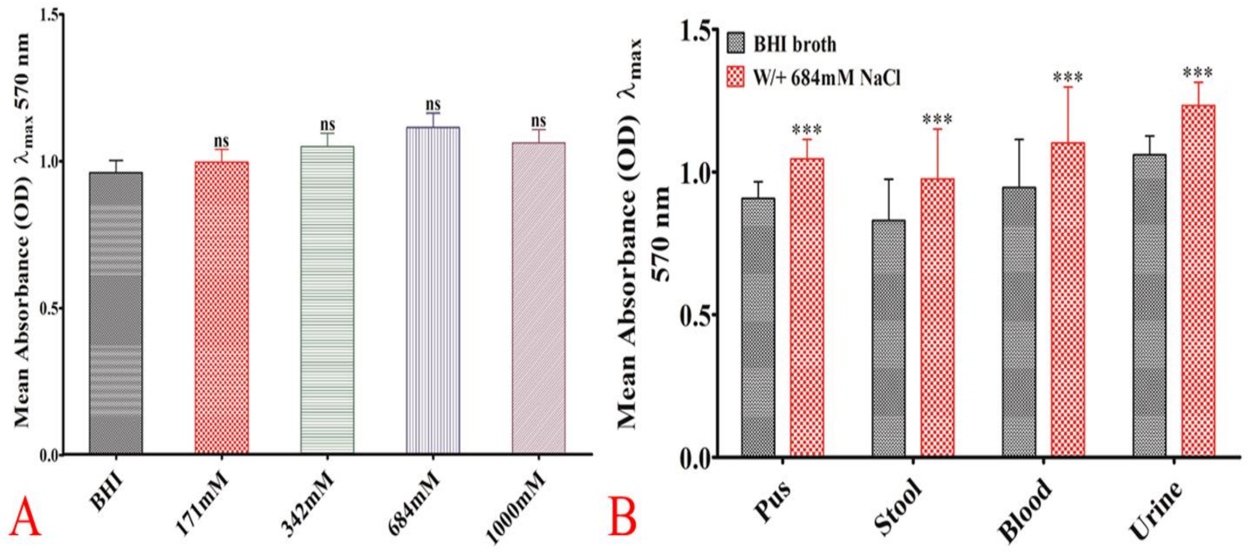

Figure S2: A. Comparative evaluation of the effect of salt concentration (sodium chloride) on biofilm formation. The increase in salt concentration positively enhanced the biofilm formation however; after the supplementation of 684mM NaCl, we noticed maximum biofilm production. Of note, the 2-fold increase in NaCl concentrations although enhanced the biofilm formation but the increase was insignificant. Almost all isolates showed enhanced biofilm formation when the salt concentration was increased from 171mM to 684mM at 37°C. Although the increase in absorbance was not significant, the optimal salt concentration for biofilm formation was found to be 684mM at 37°C. The results are shown as the mean ± SD of a quadruplicate assay. **B.** Comparative evaluation of the effect of NaCl concentration on biofilm formation among different isolates. The results are shown as the mean ± SD of a quadruplicate assay. The stool and blood isolates have shown more augmented biofilm formation (17.58% and 16.46% increase respectively) compared to urine (16.349%) and pus (15.33%) isolates when supplemented with 684mM NaCl ($P \leq 0.05$) (Supplementary data 3).

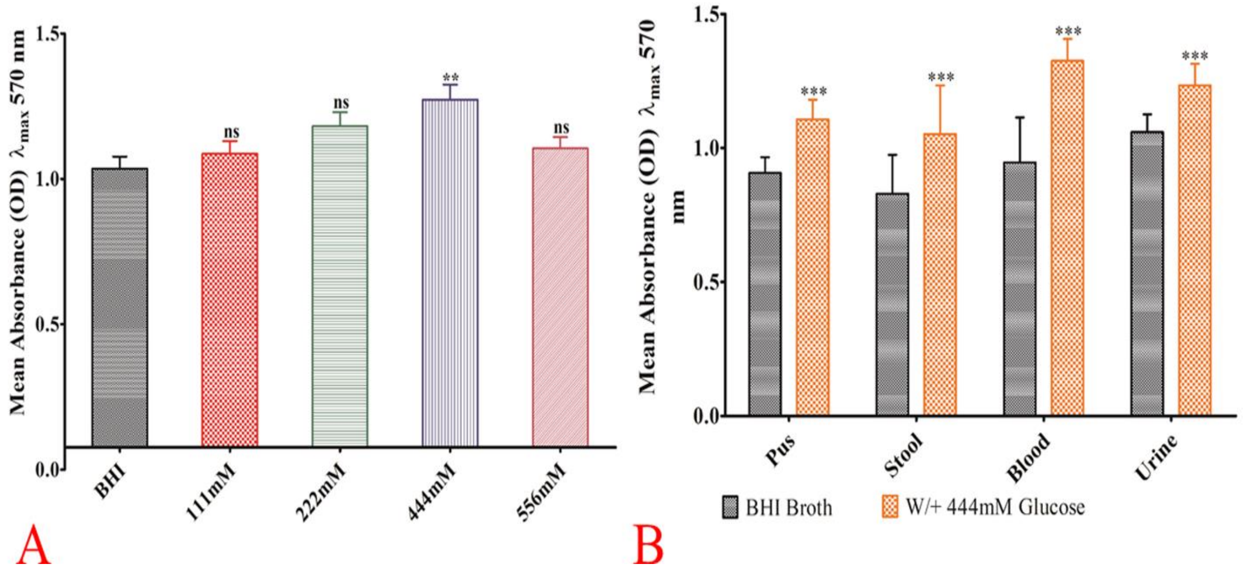

Figure S3: A. Sample wise comparative evaluation of the effect of glucose concentration on biofilm formation. Unlike salt supplementation, most of the clinical isolates displayed enhanced biofilm formation when fortified with glucose. As the concentration of glucose was increased from 111mM to 444mM, all the clinical isolates formed thicker biofilms at 37°C compared to when grown in BHI broth alone. Strikingly, at the supplemented concentration of 556mM glucose, almost all the isolates showed substantial reduction in biofilm formation at 37°C. The results are shown as the mean ± SD of a quadruplicate assay. **B.** Sample wise comparative evaluation of the effect of glucose concentration on biofilm formation. The average absorbance values were found to be 1.1070±0.07344, 1.0520±0.1818, 1.3250±0.08199, and 1.2333±0.08093 for the biofilms of the pus, stool, blood, and urine isolates grown in BHI with 444mM glucose supplementation (Supplementary Figure S3b, Supplementary data 4). Unlike salt supplementation, after the supplementation of 444mM glucose, blood isolates have shown maximum increment ($P \leq 0.01$) in their biofilm formation while urine isolates have shown the least. The results are shown as the mean ± SD of a quadruplicate assay.

.

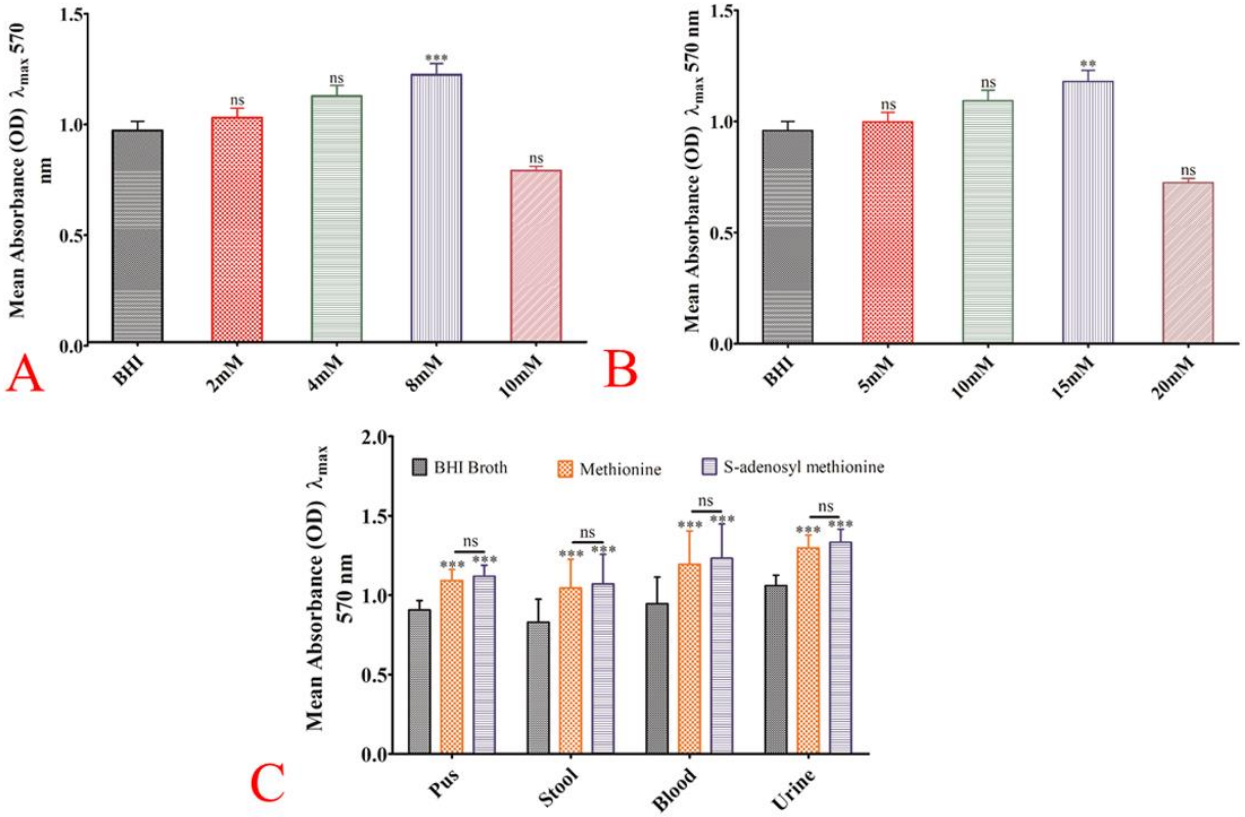

Figure S4: A. Comparative evaluation of the effect of methionine on biofilm formation. The biofilm formation of all the clinical isolates was notably reinforced by methionine and S-adenosyl methionine at the concentrations below 10mM and 20mM respectively. Methionine supplementation resulted in the densest biofilm formation by *Klebsiella* isolates at the concentration of 8mM (Supplementary data 5). The results are shown as the mean ± SD of a quadruplicate assay. **B.** Comparative evaluation of the effect of S-adenosyl methionine on biofilm formation. S-adenosyl methionine supplementation resulted in the densest biofilm formation by *Klebsiella* isolates at the concentration of 15mM (Supplementary data 5). The results are shown as the mean ± SD of a quadruplicate assay. **C.** Sample wise comparative evaluation of the effect of methionine and S-adenosyl methionine on biofilm formation. The highest percent fold surge in mean absorbance of the biofilm was observed in blood isolates (26.202% for Methionine and 23.206% for S-adenosyl methionine) accompanied by stool (25.346%, 22.53%), urine (22.358%, 20.48%) and pus (13.463%, 18.954%) isolates respectively when enriched with 8mM methionine and 15mM S-adenosyl methionine. The results are shown as the mean ± SD of a quadruplicate assay.

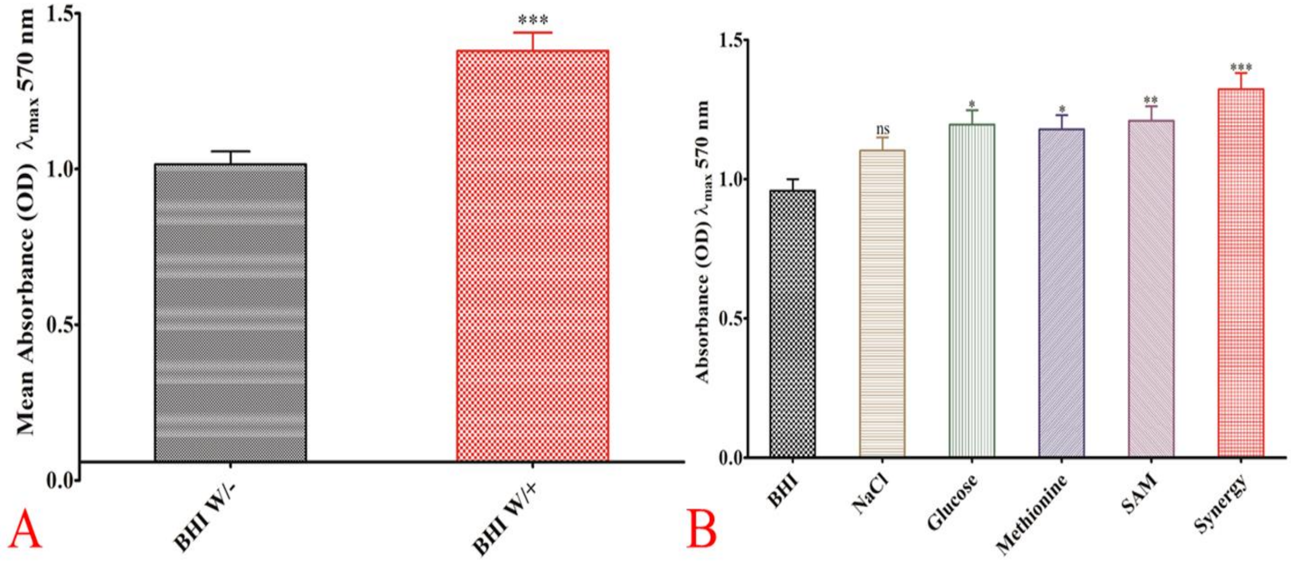

Figure S5: A. Comparative evaluation of the synergistic effect of supplementations on the biofilm forming capacity of *Klebsiella* isolates. The mean absorbance for the biofilms formed by these isolates increased almost 38% after supplementation (1.323±0.05814) compared to the unsupplemented broth (0.9589±0.04124) at 37°C (P≤0.001). The results are shown as the mean ± SD of a quadruplicate assay. **B.** Sample wise comparison of the effect of supplementations (444mM Glucose, 684mM NaCl and 20mM methionine and 10mM S-adenosyl methionine) on biofilm formation. Individual supplementation with 10mM SAM enhanced the biofilm absorbance maximally by 26.3% followed by glucose (24.725%), methionine (23.057%). However, individual supplementation by NaCl enhanced biofilm absorbance by 15.027%. Among all the isolates, the most pronounced effect of supplement mix was observed on blood isolates (46.284% increase in absorbance) while the least was observed in case of pus isolates (32.098%). The results are shown as the mean ± SD of a quadruplicate assay.

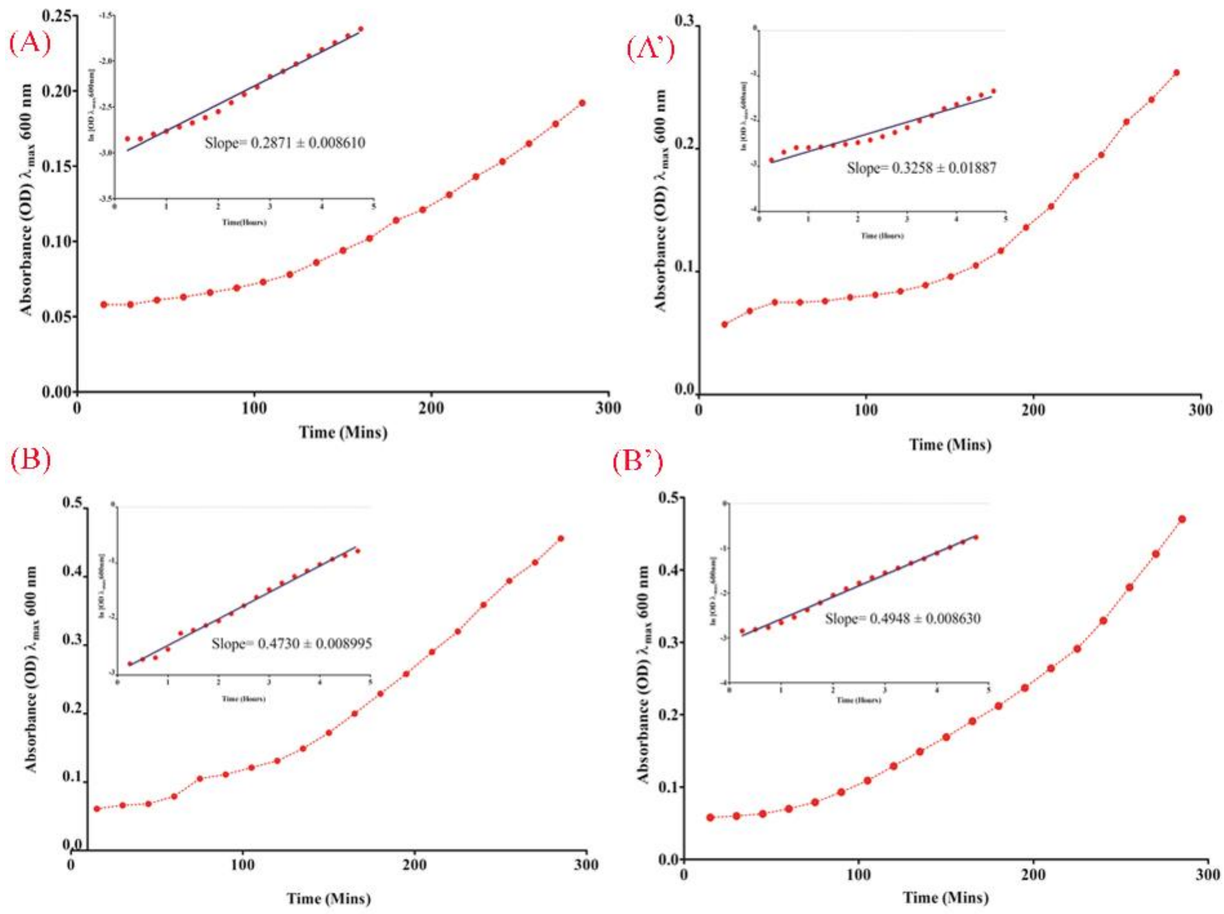

Figure S6: Graphical representation of observed growth rates of urine isolate 10894 before (A) and after (A') supplementation and of pus isolate 2884 before (B) and after (B') supplementation.

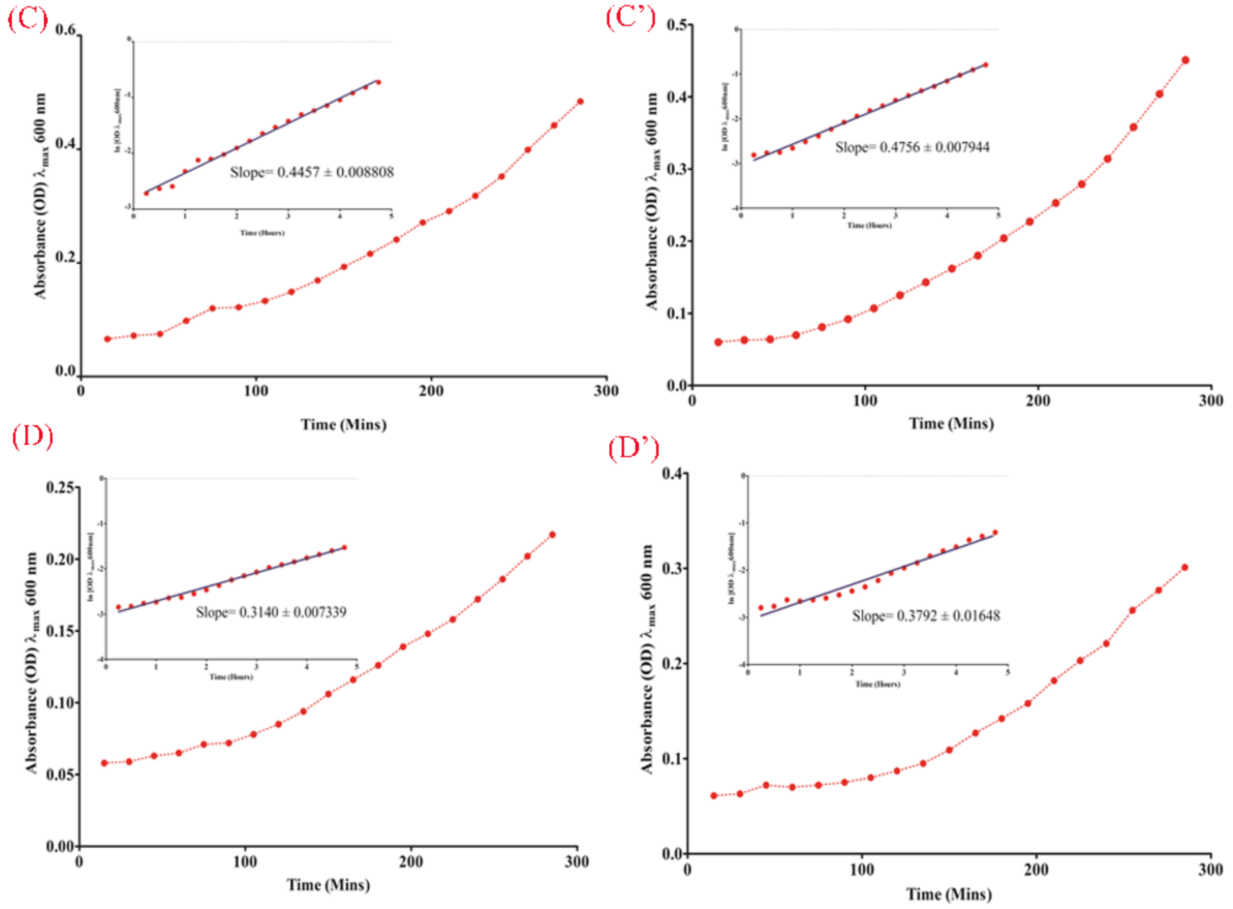

Figure S7: Graphical representation of observed growth rates of blood isolate 1739 before (C) and after (C') supplementation and of stool isolate 197 before (D) and after (D') supplementation.

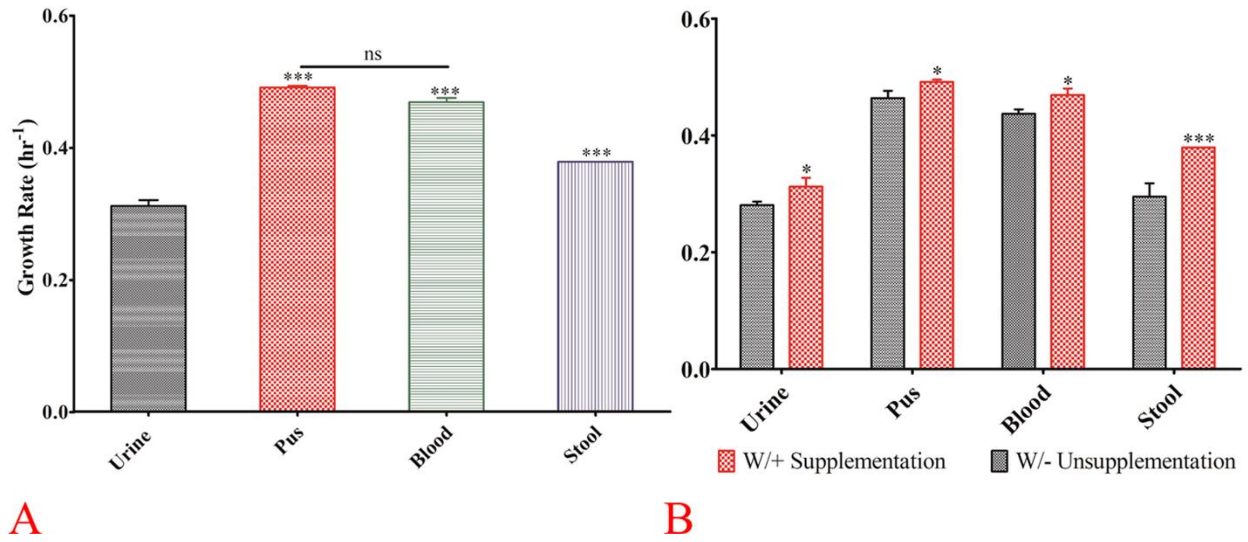

Figure S8: A. Sample wise comparative evaluation of the growth rates before supplementations. Isolates from different sample types had significantly different growth rates in BHI broth at 37°C ($P \leq 0.001$). In unsupplemented state, we found pus isolates to be the fastest growing while the urine isolates the slowest. The results are shown as the mean ± SD of a quadruplicate assay. **B.** Sample wise comparative evaluation of the growth rates after supplementations. Upon supplementation, astonishingly, we observed the highest increase in the growth rate of stool isolates (17.194%) followed by urine (11.878%) while the least effect on the growth of pus isolates (4.405%) was noted. The results are shown as the mean ± SD of a quadruplicate assay.

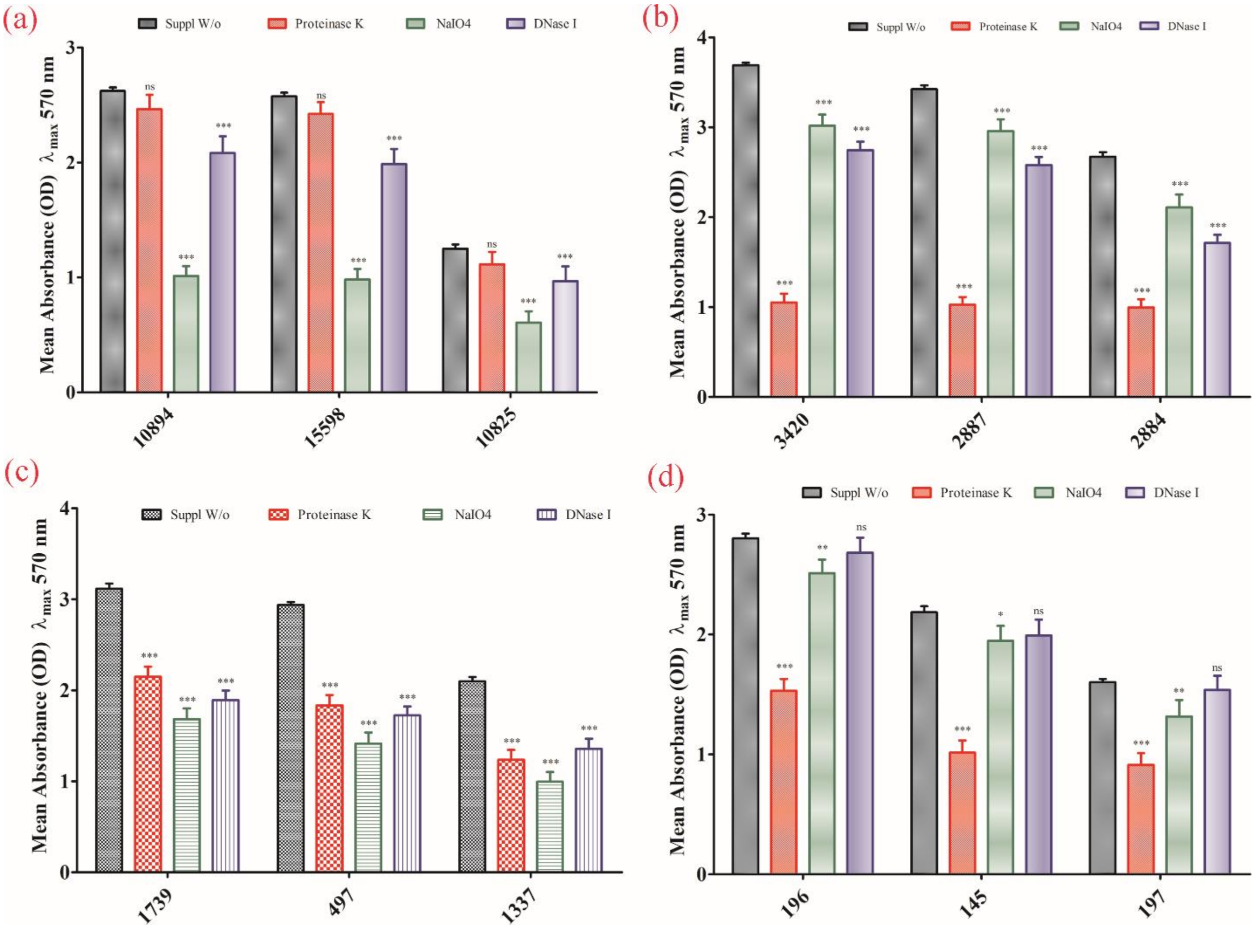

Figure S9: a. Biofilm matrix digestion of Urine isolates by various chemicals. Digestion by sodium metaperiodate ($NaIO_4$) was highly pronounced among urine isolates showing the sugar richness. **b.** Biofilm matrix digestion of pus isolates by various chemicals. Digestion by Proteinase K was highly pronounced among these isolates showing the predominance of proteins in their biofilms. **c.** Biofilm matrix digestion of Blood isolates by various chemicals. The extent of digestion by Proteinase K and $NaIO_4$ both were comparable and highly pronounced among blood isolates showing the co-dominance of proteins and sugars both in their biofilms. **d.** Biofilm matrix digestion of stool isolates by various chemicals. The extent of digestion by Proteinase K was significant compared to that of $NaIO_4$ showing the predominance of proteins. Of note, we found the digestion by $NaIO_4$ to be least effective, indicating the sparse distribution of sugars in their biofilm matrix. The results are shown as the mean ± SD of a quadruplicate assay.

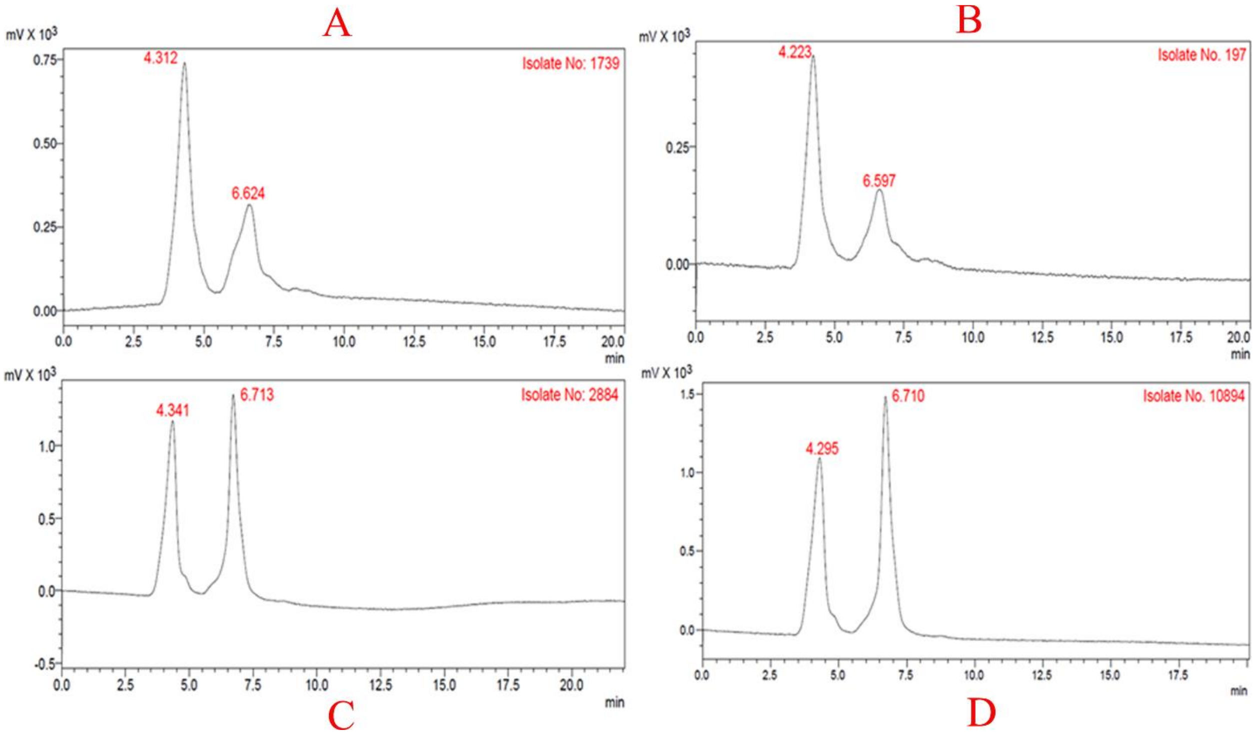

Figure S10: Analytical HPLC chromatogram of common protein(s) bands (~35kDa) as observed in SDS-PAGE. Panels A to D represent the chromatogram of the representative *K. pneumoniae* isolates obtained from blood, stool, pus, and urine samples respectively. Note the two sharp peaks of the peptides with almost similar retention times (Rt) throughout the panels A to D.

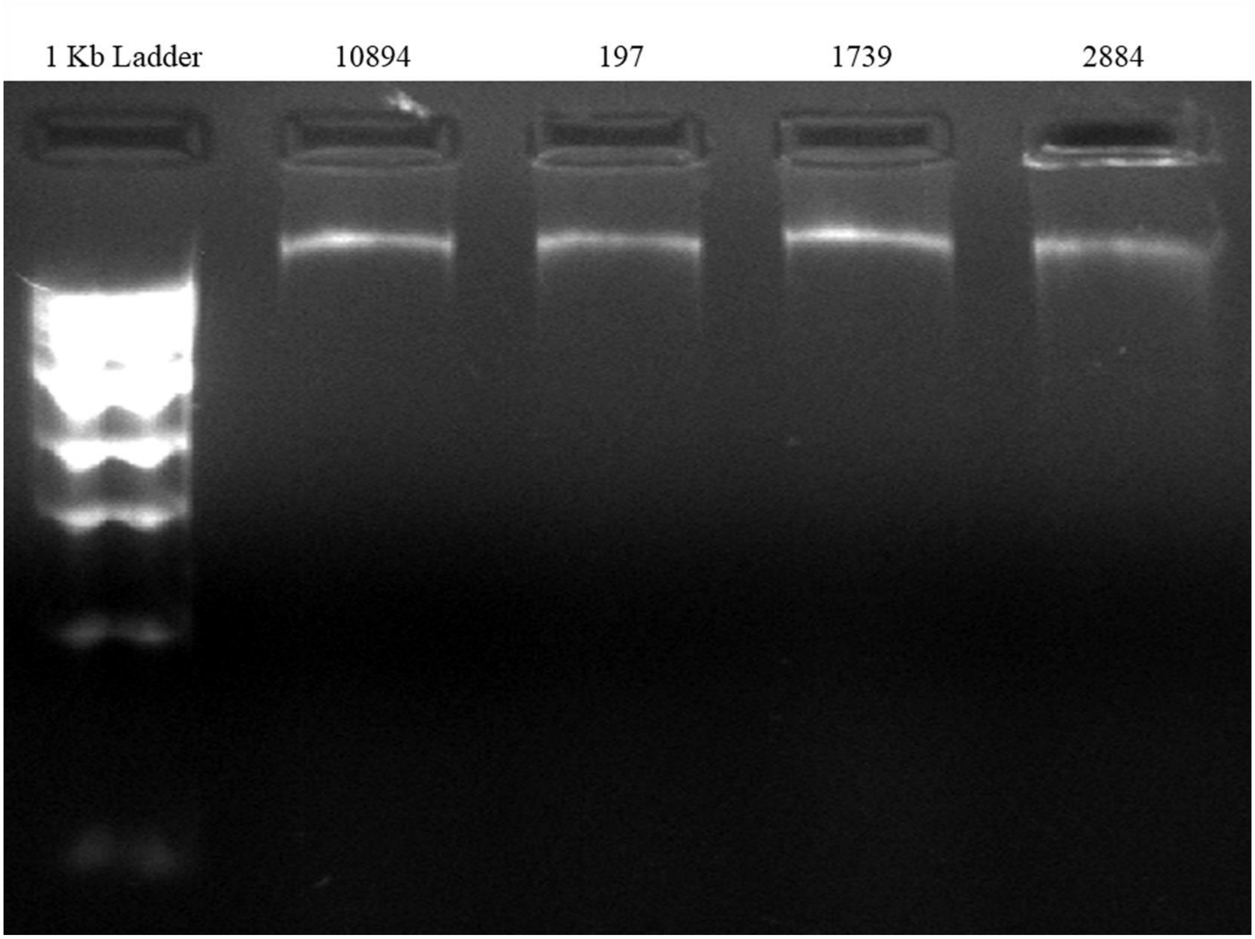

Figure S11: 1.0% agarose gel image of the purified *e*DNA of the biofilm matrices of the four representative isolates. The gel electrophoresis was executed for *e*DNA to check its tentative molecular weight and band width. The gel image shows the same molecular weight and the comparable bandwidth indicating the presence of common structural organization of matrix utilizing *e*DNA.

Supplementary table 1: Details of the number of *Klebsiella* isolates showing exaggerated biofilm response leading to major shift in biofilm category

	Potentiated moderate biofilm-former (pMBF) (n=47)		Super biofilm-former (SBF) (n=12)	
	**From**	**To**	**From**	**To**
Shifting after supplementation	Weak biofilm formers (LBF)	Potentiated moderate biofilm formers (pMBF)	Moderate biofilm formers (MBF)	Super biofilm-former (SBF)
	**(n=0)**		**(n=4)**	
	Moderate biofilm formers (MBF)	Potentiated moderate biofilm formers (pMBF)	High biofilm formers (HBF)	Super biofilm-former (SBF)
	**(n=45)**		**(n=8)**	

Supplementary table 2: The mean growth rates of high slime producing Klebsiella pneumoniae isolates from different sources of isolation in BHI broth before and after supplementation

Isolate number	Source	BHI (Biofilm OD)	Supplemented BHI (Biofilm OD)	Growth rate (BHI)	Growth rate (Supplemented BHI)
10894	Urine	2.623	4.059	0.2871±0.008610	0.3258±0.01887
15598	Urine	2.576	3.986	0.2798±0.008801	0.3151±0.01500
10825	Urine	1.25	1.689	0.2751±0.006068	0.2959±0.01481
3420	Pus	3.689	4.958	0.4730±0.008995	0.4948±0.008630
2887	Pus	3.423	3.991	0.4691±0.01116	0.4927±0.00824
2884	Pus	2.672	3.684	0.4504±0.008579	0.4869±0.008071
1739	Blood	3.118	4.125	0.4457±0.008808	0.4756±0.07944
497	Blood	2.939	3.938	0.4331±0.007093	0.4758±0.01006
1337	Blood	2.099	3.019	0.4326±0.01279	0.4564±0.008271
197	Stool	2.804	3.943	0.3140±0.007339	0.3792±0.01648
145	Stool	2.186	3.115	0.3022±0.007583	0.3795±0.01648
196	Stool	1.602	1.935	0.2704±0.01131	0.3792±0.01648

Supplementary table 3: Effect of different chemicals on the biofilm matrix of isolates from different sources in terms of biofilm reduction

Sl. No.	Isolates	Reagents	Absorbance without treatment	Absorbance upon treatment	Percent Reduction
**1.**	Pus	Proteinase K	3.261±0.3045	1.432±0.0162	56.06%
		$NaIO_4$		1.2936±0.2935	60.33%
		DNase I		2.360±0.3194	27.6%
**2.**	Urine	Proteinase K	2.15±0.45	2.0±0.44	6.976%
		$NaIO_4$		0.9322±0.1301	56.641%
		DNase I		1.679±0.3567	21.906%
**3.**	Blood	Proteinase K	2.719±0.3141	0.8547±0.2669	68.567%
		$NaIO_4$		1.1343±0.199	58.279%
		DNase I		1.9561±0.1588	28.058%
**4.**	Stool	Proteinase K	2.197±0.3470	0.7909±0.1916	64.004%
		$NaIO_4$		2.0161±0.3457	8.23%
		DNase I		1.646±0.3332	25.07%

Supplementary table 4: Annotated high performance liquid chromatogram peak(s) and its characteristic parameters of the representative isolates

Isolates	Retention time (R_t 1) in Mins	Retention time (R_t 2) in Mins	Peak Area (A 1)	Peak Area (A 2)	Height (H 1)	Height (H 2)
**1739**	4.312	6.624	25585	9770	701	222
**197**	4.223	6.597	13477	2680	425	96
**2884**	4.341	6.713	38363	42507	1195	1389
**10894**	4.295	6.710	36967	43848	1107	1495

Supplementary table 5: Protein estimation by Bradford's method

Isolate Number	Source	Protein content (µg/ml)
2884	Pus	**265.6±3.786**
10894	Urine	**246.1±1.651**
197	Stool	**309.1±3.055**
1739	Blood	**525.2±2.082**

Supplementary table 6: eDNA estimation of the biofilm matrix

Isolate Number	Source	*e*DNA content (ng/µl)
2884	Pus	**660.7±24.26**
10894	Urine	**679.6±31.24**
197	Stool	**657.6±19.49**
1739	Blood	**894.2±24.57**

MALDI MS MS Data Sheet for Stool Isolates

Supplementary Data 10

C:\Users\madhurekha\Desktop\MALDI_Data\16th batch data 070718\BHU\1\0_B1\1\1233.6652.LIFT\1SRef

Comment 1

Comment 2

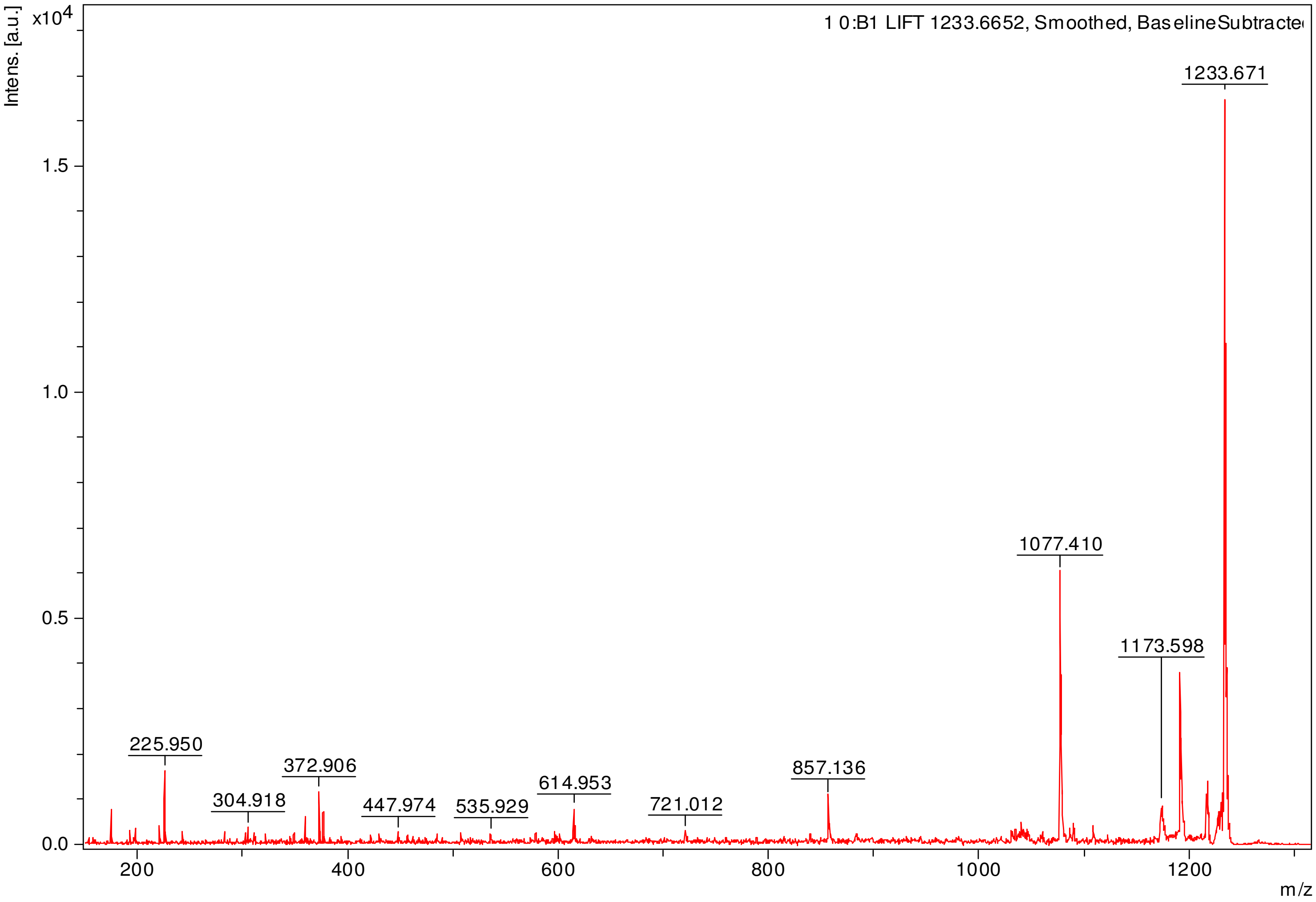

m/z	S/N	Quality Fac.	Res.	Intens.	Area
70.068	10.2	5409	459	273.67	46
112.055	11.8	688	885	328.70	47
115.045	14.0	17461	952	390.30	53
120.018	6.8	1115	887	187.48	29
129.031	4.1	58	1056	112.20	16
135.991	9.3	2618	986	253.14	40
146.970	3.1	140	1190	84.62	12
153.988	5.4	629	1041	149.57	25
157.983	5.5	43	1143	149.20	24
159.949	4.0	318	1108	109.10	18
175.006	28.0	7364	1248	794.30	130
189.934	3.3	32	1374	94.83	15
192.985	11.2	8348	1243	323.52	59
197.995	12.3	2628	1305	357.11	64
220.945	14.0	2660	1506	422.01	74
225.950	54.5	11333	1424	1658.66	317
242.947	9.7	1619	1547	305.40	58
282.937	7.9	6826	1418	275.22	68
285.921	3.3	75	1337	111.12	30
287.990	3.3	106	867	114.44	47
294.934	3.2	118	1090	113.33	38
302.905	7.6	564	1569	273.15	66
304.918	10.9	2169	1608	390.38	93
310.918	7.4	93	1368	269.50	77
321.936	6.0	133	1464	228.02	64
344.907	4.8	68	1386	192.02	62
348.915	5.7	159	1260	236.46	85
359.893	15.0	4574	1647	641.84	182
372.906	26.8	27723	1726	1187.54	336
376.913	16.5	14134	1579	734.08	230
382.880	3.9	45	1792	176.32	50
393.969	3.7	42	1550	174.58	59
421.867	3.9	164	1906	196.75	59
429.936	4.6	38	1588	235.44	86
447.974	5.3	113	1859	289.02	95
456.888	3.7	50	1704	206.05	76
473.867	3.1	39	1900	182.81	63
484.957	3.9	117	1457	240.48	111

m/z	S/N	Quality Fac.	Res.	Intens.	Area
507.882	4.2	123	1649	270.76	118
535.929	3.7	119	1386	250.47	139
578.911	3.3	65	1723	243.83	120
614.953	10.6	1106	2101	824.38	364
721.012	3.5	96	1109	308.74	322
857.136	10.4	1623	1588	1062.27	992
1040.603	4.1	34	1867	493.92	528
1046.058	3.2	44	1835	378.85	416
1077.410	50.0	65736	2136	6039.84	6002
1086.565	3.0	126	2038	364.30	385
1090.141	4.0	340	2333	490.49	455
1173.598	9.1	632	1893	938.28	1213
1175.832	3.7	80	1866	374.74	493
1191.591	37.2	35486	2438	3638.72	3744
1216.686	15.4	1519	2388	1361.16	1482
1218.946	3.3	337	3683	284.46	201
1226.771	6.7	54	1589	568.78	944
1229.053	8.4	51	2121	717.19	896
1231.344	10.4	57	2733	883.30	858
1233.671	197.2	100212	3463	16603.22	12762

C:\Users\madhurekha\Desktop\MALDI_Data\16th batch data 070718\BHU\1\0_B1\1\1965.1137.LIFT\1SRef

Comment 1

Comment 2

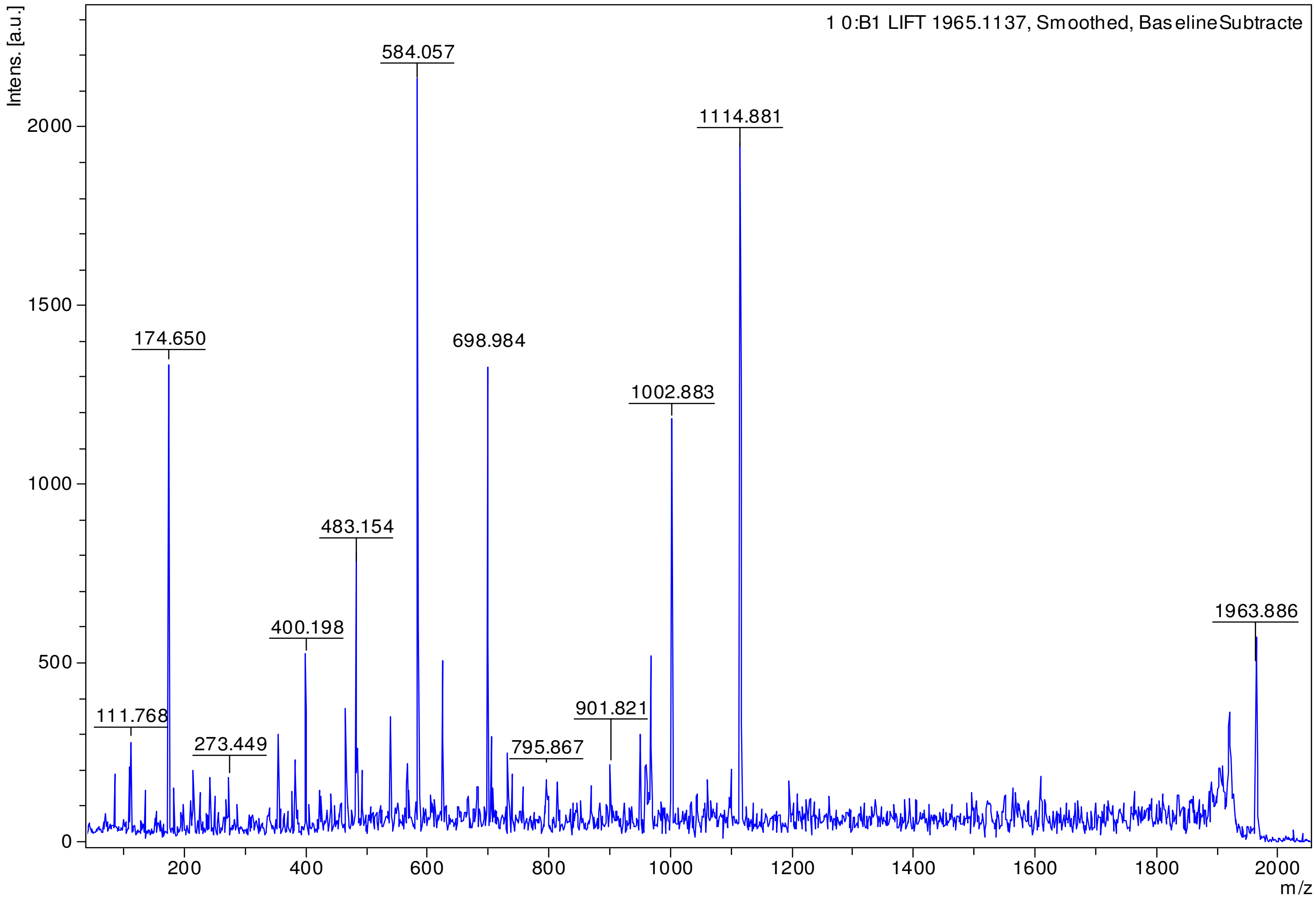

m/z	S/N	Quality Fac.	Res.	Intens.	Area
69.812	3.4	91	491	81.22	13
85.829	7.4	873	734	176.72	23
109.753	8.5	1786	1117	204.67	23
111.768	11.8	20258	1297	283.03	27
135.712	6.1	2760	1402	142.73	16
174.650	58.6	19275	1377	1338.50	198
182.645	6.0	1004	1096	138.10	27
194.575	4.1	34	1452	94.97	15
198.663	4.2	86	1175	96.10	19
210.607	4.9	347	1152	115.11	25
215.523	8.5	427	1223	196.45	41
226.543	6.0	172	1140	139.21	33
237.485	3.3	33	1934	78.07	12
242.501	7.3	349	1271	170.98	40
250.487	5.4	329	1792	128.14	22
256.470	3.9	37	1004	91.68	29
269.397	5.7	97	1609	136.37	28
273.449	7.4	956	1587	179.61	38
287.460	4.1	97	1674	103.33	22
355.275	10.0	2255	1715	292.44	79
370.232	3.0	35	2459	92.78	18
378.233	4.3	283	1848	135.55	36
383.231	7.7	87	1424	244.44	87
400.198	15.7	5048	1484	521.68	187
422.173	4.3	47	1620	149.96	53
426.226	3.4	114	1492	120.38	46
466.171	9.1	378	1786	355.04	128
483.154	19.2	1635	1882	770.71	275
493.075	4.8	297	1551	195.07	87
540.121	7.7	848	1639	343.65	162
567.046	4.6	141	2222	213.35	79
569.995	3.3	53	2366	154.20	54
584.057	44.6	59222	1862	2125.95	980
606.030	3.0	64	2386	145.43	55
625.959	10.2	3914	1804	504.02	265
698.984	26.1	66753	2287	1352.72	654
704.989	5.3	244	1984	277.50	156
732.967	5.1	187	1922	265.96	163

C:\Users\madhurekha\Desktop\MALDI_Data\16th batch data 070718\BHU\1\0_B1\1\1965.1137.LIFT\1SRef

m/z	S/N	Quality Fac.	Res.	Intens.	Area
738.935	3.8	333	1967	195.71	119
795.867	3.9	34	2324	208.26	119
901.821	3.8	233	2442	216.03	142
950.822	4.5	219	2767	256.62	161
967.863	9.0	2026	2556	521.32	364
1002.883	20.0	85180	2284	1179.20	973
1114.881	31.5	33218	2430	1930.88	1784
1903.946	4.1	71	3911	193.11	294
1908.591	3.4	78	2569	161.65	376
1919.807	7.2	490	2272	328.32	869
1963.886	12.8	958	4893	494.66	624

C:\Users\madhurekha\Desktop\MALDI_Data\16th batch data 070718\BHU\1\0_B1\1\2229.2398.LIFT\1SRef

Comment 1

Comment 2

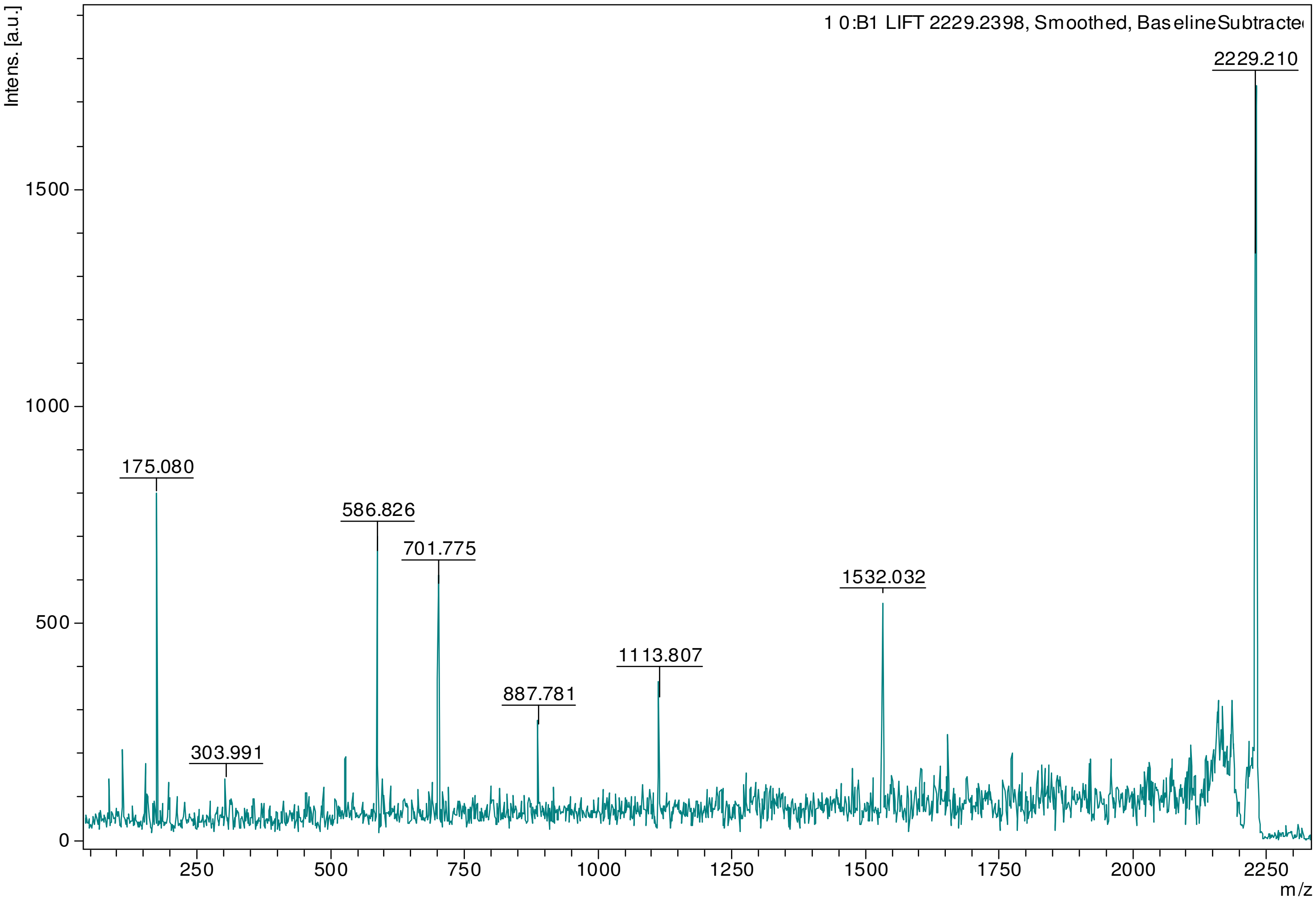

m/z	S/N	Quality Fac.	Res.	Intens.	Area
86.095	3.7	318	888	132.47	14
112.099	5.8	4262	1131	209.83	23
154.076	5.0	708	1240	171.83	24
157.520	3.0	296	1602	103.83	12
175.080	23.2	13888	1205	793.82	135
197.082	4.3	260	1048	144.46	32
213.030	3.2	33	865	107.68	32
303.991	3.8	227	1228	135.23	42
527.795	4.1	108	1297	201.81	117
586.826	12.4	9724	1771	656.38	320
701.775	9.9	12059	1907	582.11	339
887.781	3.9	226	1916	257.58	210
1113.807	4.2	205	2752	319.62	260
1532.032	6.0	1540	2508	559.64	876
2150.750	3.1	31	4027	196.21	335
2158.627	4.1	64	2397	257.36	742
2165.298	3.3	89	3326	202.64	423
2168.224	3.8	119	3072	231.81	525
2213.218	3.1	104	2741	166.76	436
2229.210	25.3	14503	4266	1341.53	2274

C:\Users\madhurekha\Desktop\MALDI_Data\16th batch data 070718\BHU\1\0_B1\1\1SRef

Comment 1

Comment 2

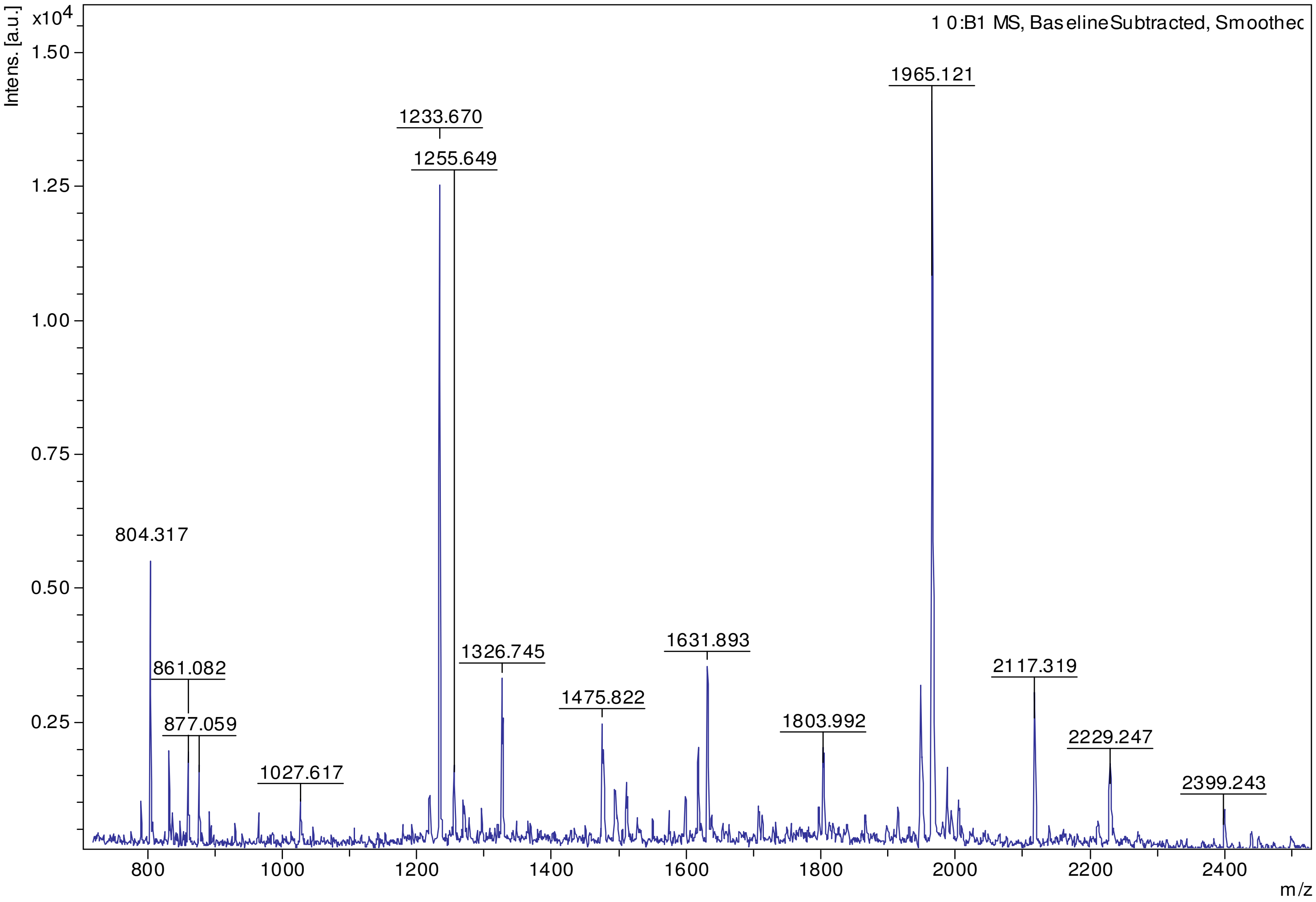

m/z	S/N	Quality Fac.	Res.	Intens.	Area
790.462	6.9	843	6070	942.72	216
804.317	42.4	6172	5917	5779.46	1387
832.350	13.4	1914	5114	1826.25	526
861.082	12.1	1600	5837	1666.02	429
877.059	10.7	569	5818	1483.75	399
1027.617	6.5	535	5784	931.21	323
1233.670	88.8	31051	8131	13319.41	4639
1255.649	9.8	1852	6814	1486.09	616
1269.671	6.1	208	6093	924.11	434
1326.745	22.1	11217	8403	3353.71	1277
1475.822	16.4	15650	9197	2514.78	1068
1477.780	7.0	1493	8072	1078.91	502
1494.883	7.0	572	6100	1064.15	667
1511.904	8.1	3724	8026	1233.09	609
1617.881	12.7	2817	8784	1924.65	988
1631.893	23.8	34192	9043	3577.56	1825
1803.992	11.7	3302	7377	1659.11	1252
1947.078	7.5	619	7733	940.75	756
1949.066	19.8	6629	8417	2495.57	1853
1965.121	86.9	12181	8794	10760.25	7834
1967.186	22.5	891	6835	2781.92	2554
1988.112	9.4	2140	7469	1132.43	969
2117.319	24.3	48010	9033	2501.34	1953
2229.247	14.0	1350	7998	1272.66	1172
2399.243	6.9	2736	5618	504.27	744

MATRIX SCIENCE Mascot Search Results

**User**	: sanovar
**Email**	: sanovar@sandor.co.in
**Search title**	:
**MS data file**	: DATA.TXT
**Database**	: SwissProt 2018_06 (557713 sequences; 200130199 residues)
**Taxonomy**	: Bacteria (Eubacteria) (333552 sequences)
**Timestamp**	: 11 Jul 2018 at 11:29:47 GMT
**Warning**	: A Peptide summary report will usually give a much clearer picture of MS/MS search results.
**Top Score**	: 138 for EFTU1_ECO24, Elongation factor Tu 1 OS=Escherichia coli O139:H28 (strain E24377A / ETEC) OX=331111 GN=tuf1 PI

Mascot Score Histogram

Protein score is -10*Log(P), where P is the probability that the observed match is a random event.
Protein scores greater than 68 are significant (p<0.05).
Protein scores are derived from ions scores as a non-probabilistic basis for ranking protein hits.

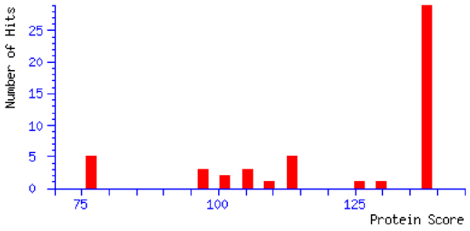

Protein Summary Report

Format As | Protein Summary (deprecated) | Help

Significance threshold p< 0.05 Max. number of hits 20

Preferred taxonomy All entries

Re-Search All | Search Unmatched

Index

	Accession	Mass	Score	Description
1.	EFTU1_ECO24	43427	138	Elongation factor Tu 1 OS=Escherichia coli O139:H28 (strain E24377A / ETEC) OX=331111 GN=tuf1 PE=3 SV=1
2.	EFTU1_ECOHS	43427	138	Elongation factor Tu 1 OS=Escherichia coli O9:H4 (strain HS) OX=331112 GN=tuf1 PE=3 SV=1
3.	EFTU1_ECOK1	43427	138	Elongation factor Tu 1 OS=Escherichia coli O1:K1 / APEC OX=405955 GN=tuf1 PE=3 SV=2
4.	EFTU1_ECOL5	43427	138	Elongation factor Tu 1 OS=Escherichia coli O6:K15:H31 (strain 536 / UPEC) OX=362663 GN=tuf1 PE=3 SV=1
5.	EFTU1_ECOLC	43427	138	Elongation factor Tu 1 OS=Escherichia coli (strain ATCC 8739 / DSM 1576 / Crooks) OX=481805 GN=tuf1 PE=
6.	EFTU1_ECOLI	43427	138	Elongation factor Tu 1 OS=Escherichia coli (strain K12) OX=83333 GN=tufA PE=1 SV=1
7.	EFTU1_ECOUT	43427	138	Elongation factor Tu 1 OS=Escherichia coli (strain UTI89 / UPEC) OX=364106 GN=tuf1 PE=1 SV=1
8.	EFTU1_SHIF8	43426	138	Elongation factor Tu 1 OS=Shigella flexneri serotype 5b (strain 8401) OX=373384 GN=tuf1 PE=3 SV=1
9.	EFTU1_SHISS	43427	138	Elongation factor Tu 1 OS=Shigella sonnei (strain Ss046) OX=300269 GN=tuf1 PE=3 SV=1
10.	EFTU2_ECO24	43456	138	Elongation factor Tu 2 OS=Escherichia coli O139:H28 (strain E24377A / ETEC) OX=331111 GN=tuf2 PE=3 SV=1
11.	EFTU2_ECOHS	43457	138	Elongation factor Tu 2 OS=Escherichia coli O9:H4 (strain HS) OX=331112 GN=tuf2 PE=3 SV=1
12.	EFTU2_ECOK1	43457	138	Elongation factor Tu 2 OS=Escherichia coli O1:K1 / APEC OX=405955 GN=tuf2 PE=3 SV=1
13.	EFTU2_ECOL5	43457	138	Elongation factor Tu 2 OS=Escherichia coli O6:K15:H31 (strain 536 / UPEC) OX=362663 GN=tuf2 PE=3 SV=1
14.	EFTU2_ECOLC	43457	138	Elongation factor Tu 2 OS=Escherichia coli (strain ATCC 8739 / DSM 1576 / Crooks) OX=481805 GN=tuf2 PE=
15.	EFTU2_ECOLI	43457	138	Elongation factor Tu 2 OS=Escherichia coli (strain K12) OX=83333 GN=tufB PE=1 SV=1
16.	EFTU2_ECOUT	43457	138	Elongation factor Tu 2 OS=Escherichia coli (strain UTI89 / UPEC) OX=364106 GN=tuf2 PE=3 SV=2
17.	EFTU2_SHIF8	43457	138	Elongation factor Tu 2 OS=Shigella flexneri serotype 5b (strain 8401) OX=373384 GN=tuf2 PE=3 SV=1
18.	EFTU2_SHISS	43457	138	Elongation factor Tu 2 OS=Shigella sonnei (strain Ss046) OX=300269 GN=tuf2 PE=3 SV=1
19.	EFTU_ECO57	43457	138	Elongation factor Tu OS=Escherichia coli O157:H7 OX=83334 GN=tufA PE=3 SV=2
20.	EFTU_ECOL6	43457	138	Elongation factor Tu OS=Escherichia coli O6:H1 (strain CFT073 / ATCC 700928 / UPEC) OX=199310 GN=tufA PI

Results List

1. EFTU1_ECO24 **Mass:** 43427 **Score:** 138 **Expect:** 5.3e-09 **Matches:** 11
Elongation factor Tu 1 OS=Escherichia coli O139:H28 (strain E24377A / ETEC) OX=331111 GN=tuf1 PE=3 SV=1

Observed	Mr(expt)	Mr(calc)	ppm	Start	End	Miss	Ions	Peptide
837.5072	836.4999	836.4868	15.7	118 -	124	0	---	R.EHILLGR.Q
1027.6166	1026.6093	1026.5822	26.4	271 -	280	0	---	R.AGENVGVLLR.G
1214.6581	1213.6508	1213.6230	22.9	305 -	314	0	---	K.FESEVYILSK.D
1233.6701	1232.6629	1232.6091	43.6	326 -	334	0	15	K.GYRPQFYFR.T
1315.7305	1314.7232	1314.6060	89.1	254 -	264	1	---	K.STCTGVEMFRK.L
1574.9723	1573.9650	1573.9304	22.0	290 -	304	0	---	R.GQVLAKPGTIKPHTK.F
1728.9325	1727.9253	1727.8366	51.3	305 -	319	1	---	K.FESEVYILSKDEGGR.H
1797.0350	1796.0277	1795.9325	53.1	359 -	374	0	---	K.MVVTLIHPIAMDDGLR.F + Oxidation (M)
1803.9918	1802.9845	1802.8799	58.0	60 -	75	0	---	R.GITINTSHVEYDTPTR.H
1965.1206	1964.1134	1963.9527	81.8	156 -	172	0	44	R.ELLSQYDFPGDDTPIVR.G
2117.3190	2116.3117	2116.1568	73.2	206 -	224	0	28	R.AIDKPFLLPIEDVFSISGR.G

No match to: 723.3202, 725.3349, 790.4618, 804.3175, 832.3504, 848.3077, 855.0634, 861.0816, 877.0593, 879.0642, 893.0384, 897.0395, 930.4819, 941.5245, 965.5432, 985.5503, 1001.5439, 1046.5846, 1179.6348, 1193.6685, 1219.5995, 1255.6486, 1269.6710, 1271.6285, 1277.6571, 1296.7322, 1320.6617, 1326.7448, 1348.7190, 1368.7612, 1450.7841, 1472.8880, 1475.8216, 1477.7796, 1494.8828, 1497.7996, 1509.8403, 1511.9038, 1527.8609, 1550.8947, 1598.9195, 1617.8806, 1631.8934, 1654.9031, 1707.9342, 1712.9042, 1733.0067, 1749.9856, 1755.9948, 1818.0212, 1866.0850, 1898.0605, 1915.0945, 1931.0593, 1947.0781, 1949.0662, 1967.1856, 1981.0932, 1988.1120, 1994.1406, 2005.1346, 2010.0916, 2139.2472, 2211.2249, 2229.2467, 2233.2426, 2271.2633, 2384.1999, 2399.2428, 2439.4144, 2451.4028, 2498.4046, 2615.4719, 2705.3847, 2808.6002, 3284.5981

2. EFTU1_ECOHS **Mass:** 43427 **Score:** 138 **Expect:** 5.3e-09 **Matches:** 11
Elongation factor Tu 1 OS=Escherichia coli O9:H4 (strain HS) OX=331112 GN=tuf1 PE=3 SV=1

Observed	Mr(expt)	Mr(calc)	ppm	Start	End	Miss	Ions	Peptide
837.5072	836.4999	836.4868	15.7	118 -	124	0	---	R.EHILLGR.Q

1027.6166	1026.6093	1026.5822	26.4	271 - 280	0	---	R.AGENVGVLLR.G
1214.6581	1213.6508	1213.6230	22.9	305 - 314	0	---	K.FESEVYILSK.D
1233.6701	1232.6629	1232.6091	43.6	326 - 334	0	15	K.GYRPQFYFR.T
1315.7305	1314.7232	1314.6060	89.1	254 - 264	1	---	K.STCTGVEMFRK.L
1574.9723	1573.9650	1573.9304	22.0	290 - 304	0	---	R.GQVLAKPGTIKPHTK.F
1728.9325	1727.9253	1727.8366	51.3	305 - 319	1	---	K.FESEVYILSKDEGGR.H
1797.0350	1796.0277	1795.9325	53.1	359 - 374	0	---	K.MVVTLIHPIAMDDGLR.F + Oxidation (M)
1803.9918	1802.9845	1802.8799	58.0	60 - 75	0	---	R.GITINTSHVEYDTPTR.H
1965.1206	1964.1134	1963.9527	81.8	156 - 172	0	44	R.ELLSQYDFPGDDTPIVR.G
2117.3190	2116.3117	2116.1568	73.2	206 - 224	0	28	R.AIDKPFLLPIEDVFSISGR.G

No match to: 723.3202, 725.3349, 790.4618, 804.3175, 832.3504, 848.3077, 855.0634, 861.0816, 877.0593, 879.0642, 893.0384, 897.0395, 930.4819, 941.5245, 965.5432, 985.5503, 1001.5439, 1046.5846, 1179.6348, 1193.6685, 1219.5995, 1255.6486, 1269.6710, 1271.6285, 1277.6571, 1296.7322, 1320.6617, 1326.7448, 1348.7190, 1368.7612, 1450.7841, 1472.8880, 1475.8216, 1477.7796, 1494.8828, 1497.7996, 1509.8403, 1511.9038, 1527.8609, 1550.8947, 1598.9195, 1617.8806, 1631.8934, 1654.9031, 1707.9342, 1712.9042, 1733.0067, 1749.9856, 1755.9948, 1818.0212, 1866.0850, 1898.0605, 1915.0945, 1931.0593, 1947.0781, 1949.0662, 1967.1856, 1981.0932, 1988.1120, 1994.1406, 2005.1346, 2010.0916, 2139.2472, 2211.2249, 2229.2467, 2233.2426, 2271.2633, 2384.1999, 2399.2428, 2439.4144, 2451.4028, 2498.4046, 2615.4719, 2705.3847, 2808.6002, 3284.5981

3. EFTU1_ECOK1 **Mass:** 43427 **Score:** 138 **Expect:** 5.3e-09 **Matches:** 11

Elongation factor Tu 1 OS=Escherichia coli O1:K1 / APEC OX=405955 GN=tuf1 PE=3 SV=2

Observed	Mr(expt)	Mr(calc)	ppm	Start - End	Miss	Ions	Peptide
837.5072	836.4999	836.4868	15.7	118 - 124	0	---	R.EHILLGR.Q
1027.6166	1026.6093	1026.5822	26.4	271 - 280	0	---	R.AGENVGVLLR.G
1214.6581	1213.6508	1213.6230	22.9	305 - 314	0	---	K.FESEVYILSK.D
1233.6701	1232.6629	1232.6091	43.6	326 - 334	0	15	K.GYRPQFYFR.T
1315.7305	1314.7232	1314.6060	89.1	254 - 264	1	---	K.STCTGVEMFRK.L
1574.9723	1573.9650	1573.9304	22.0	290 - 304	0	---	R.GQVLAKPGTIKPHTK.F
1728.9325	1727.9253	1727.8366	51.3	305 - 319	1	---	K.FESEVYILSKDEGGR.H
1797.0350	1796.0277	1795.9325	53.1	359 - 374	0	---	K.MVVTLIHPIAMDDGLR.F + Oxidation (M)
1803.9918	1802.9845	1802.8799	58.0	60 - 75	0	---	R.GITINTSHVEYDTPTR.H
1965.1206	1964.1134	1963.9527	81.8	156 - 172	0	44	R.ELLSQYDFPGDDTPIVR.G
2117.3190	2116.3117	2116.1568	73.2	206 - 224	0	28	R.AIDKPFLLPIEDVFSISGR.G

No match to: 723.3202, 725.3349, 790.4618, 804.3175, 832.3504, 848.3077, 855.0634, 861.0816, 877.0593, 879.0642, 893.0384, 897.0395, 930.4819, 941.5245, 965.5432, 985.5503, 1001.5439, 1046.5846, 1179.6348, 1193.6685, 1219.5995, 1255.6486, 1269.6710, 1271.6285, 1277.6571, 1296.7322, 1320.6617, 1326.7448, 1348.7190, 1368.7612, 1450.7841, 1472.8880, 1475.8216, 1477.7796, 1494.8828, 1497.7996, 1509.8403, 1511.9038, 1527.8609, 1550.8947, 1598.9195, 1617.8806, 1631.8934, 1654.9031, 1707.9342, 1712.9042, 1733.0067, 1749.9856, 1755.9948, 1818.0212, 1866.0850, 1898.0605, 1915.0945, 1931.0593, 1947.0781, 1949.0662, 1967.1856, 1981.0932, 1988.1120, 1994.1406, 2005.1346, 2010.0916, 2139.2472, 2211.2249, 2229.2467, 2233.2426, 2271.2633, 2384.1999, 2399.2428, 2439.4144, 2451.4028, 2498.4046, 2615.4719, 2705.3847, 2808.6002, 3284.5981

4. EFTU1_ECOL5 **Mass:** 43427 **Score:** 138 **Expect:** 5.3e-09 **Matches:** 11

Elongation factor Tu 1 OS=Escherichia coli O6:K15:H31 (strain 536 / UPEC) OX=362663 GN=tuf1 PE=3 SV=1

Observed	Mr(expt)	Mr(calc)	ppm	Start - End	Miss	Ions	Peptide
837.5072	836.4999	836.4868	15.7	118 - 124	0	---	R.EHILLGR.Q
1027.6166	1026.6093	1026.5822	26.4	271 - 280	0	---	R.AGENVGVLLR.G
1214.6581	1213.6508	1213.6230	22.9	305 - 314	0	---	K.FESEVYILSK.D
1233.6701	1232.6629	1232.6091	43.6	326 - 334	0	15	K.GYRPQFYFR.T
1315.7305	1314.7232	1314.6060	89.1	254 - 264	1	---	K.STCTGVEMFRK.L
1574.9723	1573.9650	1573.9304	22.0	290 - 304	0	---	R.GQVLAKPGTIKPHTK.F
1728.9325	1727.9253	1727.8366	51.3	305 - 319	1	---	K.FESEVYILSKDEGGR.H
1797.0350	1796.0277	1795.9325	53.1	359 - 374	0	---	K.MVVTLIHPIAMDDGLR.F + Oxidation (M)
1803.9918	1802.9845	1802.8799	58.0	60 - 75	0	---	R.GITINTSHVEYDTPTR.H
1965.1206	1964.1134	1963.9527	81.8	156 - 172	0	44	R.ELLSQYDFPGDDTPIVR.G
2117.3190	2116.3117	2116.1568	73.2	206 - 224	0	28	R.AIDKPFLLPIEDVFSISGR.G

No match to: 723.3202, 725.3349, 790.4618, 804.3175, 832.3504, 848.3077, 855.0634, 861.0816, 877.0593, 879.0642, 893.0384, 897.0395, 930.4819, 941.5245, 965.5432, 985.5503, 1001.5439, 1046.5846, 1179.6348, 1193.6685, 1219.5995, 1255.6486, 1269.6710, 1271.6285, 1277.6571, 1296.7322, 1320.6617, 1326.7448, 1348.7190, 1368.7612, 1450.7841, 1472.8880, 1475.8216, 1477.7796, 1494.8828, 1497.7996, 1509.8403, 1511.9038, 1527.8609, 1550.8947, 1598.9195, 1617.8806, 1631.8934, 1654.9031, 1707.9342, 1712.9042, 1733.0067, 1749.9856, 1755.9948, 1818.0212, 1866.0850, 1898.0605, 1915.0945, 1931.0593, 1947.0781, 1949.0662, 1967.1856, 1981.0932, 1988.1120, 1994.1406, 2005.1346, 2010.0916, 2139.2472, 2211.2249, 2229.2467, 2233.2426, 2271.2633, 2384.1999, 2399.2428, 2439.4144, 2451.4028, 2498.4046, 2615.4719, 2705.3847, 2808.6002, 3284.5981

5. EFTU1_ECOLC **Mass:** 43427 **Score:** 138 **Expect:** 5.3e-09 **Matches:** 11

Elongation factor Tu 1 OS=Escherichia coli (strain ATCC 8739 / DSM 1576 / Crooks) OX=481805 GN=tuf1 PE=3 SV=1

Observed	Mr(expt)	Mr(calc)	ppm	Start - End	Miss	Ions	Peptide
837.5072	836.4999	836.4868	15.7	118 - 124	0	---	R.EHILLGR.Q
1027.6166	1026.6093	1026.5822	26.4	271 - 280	0	---	R.AGENVGVLLR.G
1214.6581	1213.6508	1213.6230	22.9	305 - 314	0	---	K.FESEVYILSK.D
1233.6701	1232.6629	1232.6091	43.6	326 - 334	0	15	K.GYRPQFYFR.T
1315.7305	1314.7232	1314.6060	89.1	254 - 264	1	---	K.STCTGVEMFRK.L
1574.9723	1573.9650	1573.9304	22.0	290 - 304	0	---	R.GQVLAKPGTIKPHTK.F
1728.9325	1727.9253	1727.8366	51.3	305 - 319	1	---	K.FESEVYILSKDEGGR.H
1797.0350	1796.0277	1795.9325	53.1	359 - 374	0	---	K.MVVTLIHPIAMDDGLR.F + Oxidation (M)
1803.9918	1802.9845	1802.8799	58.0	60 - 75	0	---	R.GITINTSHVEYDTPTR.H
1965.1206	1964.1134	1963.9527	81.8	156 - 172	0	44	R.ELLSQYDFPGDDTPIVR.G
2117.3190	2116.3117	2116.1568	73.2	206 - 224	0	28	R.AIDKPFLLPIEDVFSISGR.G

No match to: 723.3202, 725.3349, 790.4618, 804.3175, 832.3504, 848.3077, 855.0634, 861.0816, 877.0593, 879.0642, 893.0384, 897.0395, 930.4819, 941.5245, 965.5432, 985.5503, 1001.5439, 1046.5846, 1179.6348, 1193.6685, 1219.5995, 1255.6486, 1269.6710, 1271.6285, 1277.6571, 1296.7322, 1320.6617, 1326.7448, 1348.7190, 1368.7612, 1450.7841, 1472.8880, 1475.8216, 1477.7796, 1494.8828, 1497.7996, 1509.8403, 1511.9038, 1527.8609, 1550.8947, 1598.9195, 1617.8806, 1631.8934, 1654.9031, 1707.9342, 1712.9042, 1733.0067, 1749.9856, 1755.9948, 1818.0212, 1866.0850, 1898.0605, 1915.0945, 1931.0593, 1947.0781, 1949.0662, 1967.1856, 1981.0932, 1988.1120, 1994.1406, 2005.1346, 2010.0916, 2139.2472, 2211.2249, 2229.2467, 2233.2426, 2271.2633, 2384.1999, 2399.2428, 2439.4144, 2451.4028, 2498.4046, 2615.4719, 2705.3847, 2808.6002, 3284.5981

6. EFTU1_ECOLI **Mass:** 43427 **Score:** 138 **Expect:** 5.3e-09 **Matches:** 11

Elongation factor Tu 1 OS=Escherichia coli (strain K12) OX=83333 GN=tufA PE=1 SV=1

Observed	Mr(expt)	Mr(calc)	ppm	Start - End	Miss	Ions	Peptide
837.5072	836.4999	836.4868	15.7	118 - 124	0	---	R.EHILLGR.Q
1027.6166	1026.6093	1026.5822	26.4	271 - 280	0	---	R.AGENVGVLLR.G
1214.6581	1213.6508	1213.6230	22.9	305 - 314	0	---	K.FESEVYILSK.D
1233.6701	1232.6629	1232.6091	43.6	326 - 334	0	15	K.GYRPQFYFR.T
1315.7305	1314.7232	1314.6060	89.1	254 - 264	1	---	K.STCTGVEMFRK.L
1574.9723	1573.9650	1573.9304	22.0	290 - 304	0	---	R.GQVLAKPGTIKPHTK.F

1728.9325	1727.9253	1727.8366	51.3	305	319	1	---	K.FESEVYILSKDEGGR.H
1797.0350	1796.0277	1795.9325	53.1	359	374	0	---	K.MVVTLIHPIAMDDGLR.F + Oxidation (M)
1803.9918	1802.9845	1802.8799	58.0	60	75	0	---	R.GITINTSHVEYDTPTR.H
1965.1206	1964.1134	1963.9527	81.8	156	172	0	44	R.ELLSQYDFPGDDTPIVR.G
2117.3190	2116.3117	2116.1568	73.2	206	224	0	28	R.AIDKPFLLPIEDVFSISGR.G

No match to: 723.3202, 725.3349, 790.4618, 804.3175, 832.3504, 848.3077, 855.0634, 861.0816, 877.0593, 879.0642, 893.0384, 897.0395, 930.4819, 941.5245, 965.5432, 985.5503, 1001.5439, 1046.5846, 1179.6348, 1193.6685, 1219.5995, 1255.6486, 1269.6710, 1271.6285, 1277.6571, 1296.7322, 1320.6617, 1326.7448, 1348.7190, 1368.7612, 1450.7841, 1472.8880, 1475.8216, 1477.7796, 1494.8828, 1497.7996, 1509.8403, 1511.9038, 1527.8609, 1550.8947, 1598.9195, 1617.8806, 1631.8934, 1654.9031, 1707.9342, 1712.9042, 1733.0067, 1749.9856, 1755.9948, 1818.0212, 1866.0850, 1898.0605, 1915.0945, 1931.0593, 1947.0781, 1949.0662, 1967.1856, 1981.0932, 1988.1120, 1994.1406, 2005.1346, 2010.0916, 2139.2472, 2211.2249, 2229.2467, 2233.2426, 2271.2633, 2384.1999, 2399.2428, 2439.4144, 2451.4028, 2498.4046, 2615.4719, 2705.3847, 2808.6002, 3284.5981

7. EFTU1_ECOUT **Mass:** 43427 **Score:** 138 **Expect:** 5.3e-09 **Matches:** 11

Elongation factor Tu 1 OS=Escherichia coli (strain UTI89 / UPEC) OX=364106 GN=tuf1 PE=1 SV=1

Observed	Mr(expt)	Mr(calc)	ppm	Start	End	Miss	Ions	Peptide
837.5072	836.4999	836.4868	15.7	118	124	0	---	R.EHILLGR.Q
1027.6166	1026.6093	1026.5822	26.4	271	280	0	---	R.AGENVGVLLR.G
1214.6581	1213.6508	1213.6230	22.9	305	314	0	---	K.FESEVYILSK.D
1233.6701	1232.6629	1232.6091	43.6	326	334	0	15	K.GYRPQFYFR.T
1315.7305	1314.7232	1314.6060	89.1	254	264	1	---	K.STCTGVEMFRK.L
1574.9723	1573.9650	1573.9304	22.0	290	304	0	---	R.GQVLAKPGTIKPHTK.F
1728.9325	1727.9253	1727.8366	51.3	305	319	1	---	K.FESEVYILSKDEGGR.H
1797.0350	1796.0277	1795.9325	53.1	359	374	0	---	K.MVVTLIHPIAMDDGLR.F + Oxidation (M)
1803.9918	1802.9845	1802.8799	58.0	60	75	0	---	R.GITINTSHVEYDTPTR.H
1965.1206	1964.1134	1963.9527	81.8	156	172	0	44	R.ELLSQYDFPGDDTPIVR.G
2117.3190	2116.3117	2116.1568	73.2	206	224	0	28	R.AIDKPFLLPIEDVFSISGR.G

No match to: 723.3202, 725.3349, 790.4618, 804.3175, 832.3504, 848.3077, 855.0634, 861.0816, 877.0593, 879.0642, 893.0384, 897.0395, 930.4819, 941.5245, 965.5432, 985.5503, 1001.5439, 1046.5846, 1179.6348, 1193.6685, 1219.5995, 1255.6486, 1269.6710, 1271.6285, 1277.6571, 1296.7322, 1320.6617, 1326.7448, 1348.7190, 1368.7612, 1450.7841, 1472.8880, 1475.8216, 1477.7796, 1494.8828, 1497.7996, 1509.8403, 1511.9038, 1527.8609, 1550.8947, 1598.9195, 1617.8806, 1631.8934, 1654.9031, 1707.9342, 1712.9042, 1733.0067, 1749.9856, 1755.9948, 1818.0212, 1866.0850, 1898.0605, 1915.0945, 1931.0593, 1947.0781, 1949.0662, 1967.1856, 1981.0932, 1988.1120, 1994.1406, 2005.1346, 2010.0916, 2139.2472, 2211.2249, 2229.2467, 2233.2426, 2271.2633, 2384.1999, 2399.2428, 2439.4144, 2451.4028, 2498.4046, 2615.4719, 2705.3847, 2808.6002, 3284.5981

8. EFTU1_SHIF8 **Mass:** 43426 **Score:** 138 **Expect:** 5.3e-09 **Matches:** 11

Elongation factor Tu 1 OS=Shigella flexneri serotype 5b (strain 8401) OX=373384 GN=tuf1 PE=3 SV=1

Observed	Mr(expt)	Mr(calc)	ppm	Start	End	Miss	Ions	Peptide
837.5072	836.4999	836.4868	15.7	118	124	0	---	R.EHILLGR.Q
1027.6166	1026.6093	1026.5822	26.4	271	280	0	---	R.AGENVGVLLR.G
1214.6581	1213.6508	1213.6230	22.9	305	314	0	---	K.FESEVYILSK.D
1233.6701	1232.6629	1232.6091	43.6	326	334	0	15	K.GYRPQFYFR.T
1315.7305	1314.7232	1314.6060	89.1	254	264	1	---	K.STCTGVEMFRK.L
1574.9723	1573.9650	1573.9304	22.0	290	304	0	---	R.GQVLAKPGTIKPHTK.F
1728.9325	1727.9253	1727.8366	51.3	305	319	1	---	K.FESEVYILSKDEGGR.H
1797.0350	1796.0277	1795.9325	53.1	359	374	0	---	K.MVVTLIHPIAMDDGLR.F + Oxidation (M)
1803.9918	1802.9845	1802.8799	58.0	60	75	0	---	R.GITINTSHVEYDTPTR.H
1965.1206	1964.1134	1963.9527	81.8	156	172	0	44	R.ELLSQYDFPGDDTPIVR.G
2117.3190	2116.3117	2116.1568	73.2	206	224	0	28	R.AIDKPFLLPIEDVFSISGR.G

No match to: 723.3202, 725.3349, 790.4618, 804.3175, 832.3504, 848.3077, 855.0634, 861.0816, 877.0593, 879.0642, 893.0384, 897.0395, 930.4819, 941.5245, 965.5432, 985.5503, 1001.5439, 1046.5846, 1179.6348, 1193.6685, 1219.5995, 1255.6486, 1269.6710, 1271.6285, 1277.6571, 1296.7322, 1320.6617, 1326.7448, 1348.7190, 1368.7612, 1450.7841, 1472.8880, 1475.8216, 1477.7796, 1494.8828, 1497.7996, 1509.8403, 1511.9038, 1527.8609, 1550.8947, 1598.9195, 1617.8806, 1631.8934, 1654.9031, 1707.9342, 1712.9042, 1733.0067, 1749.9856, 1755.9948, 1818.0212, 1866.0850, 1898.0605, 1915.0945, 1931.0593, 1947.0781, 1949.0662, 1967.1856, 1981.0932, 1988.1120, 1994.1406, 2005.1346, 2010.0916, 2139.2472, 2211.2249, 2229.2467, 2233.2426, 2271.2633, 2384.1999, 2399.2428, 2439.4144, 2451.4028, 2498.4046, 2615.4719, 2705.3847, 2808.6002, 3284.5981

9. EFTU1_SHISS **Mass:** 43427 **Score:** 138 **Expect:** 5.3e-09 **Matches:** 11

Elongation factor Tu 1 OS=Shigella sonnei (strain Ss046) OX=300269 GN=tuf1 PE=3 SV=1

Observed	Mr(expt)	Mr(calc)	ppm	Start	End	Miss	Ions	Peptide
837.5072	836.4999	836.4868	15.7	118	124	0	---	R.EHILLGR.Q
1027.6166	1026.6093	1026.5822	26.4	271	280	0	---	R.AGENVGVLLR.G
1214.6581	1213.6508	1213.6230	22.9	305	314	0	---	K.FESEVYILSK.D
1233.6701	1232.6629	1232.6091	43.6	326	334	0	15	K.GYRPQFYFR.T
1315.7305	1314.7232	1314.6060	89.1	254	264	1	---	K.STCTGVEMFRK.L
1574.9723	1573.9650	1573.9304	22.0	290	304	0	---	R.GQVLAKPGTIKPHTK.F
1728.9325	1727.9253	1727.8366	51.3	305	319	1	---	K.FESEVYILSKDEGGR.H
1797.0350	1796.0277	1795.9325	53.1	359	374	0	---	K.MVVTLIHPIAMDDGLR.F + Oxidation (M)
1803.9918	1802.9845	1802.8799	58.0	60	75	0	---	R.GITINTSHVEYDTPTR.H
1965.1206	1964.1134	1963.9527	81.8	156	172	0	44	R.ELLSQYDFPGDDTPIVR.G
2117.3190	2116.3117	2116.1568	73.2	206	224	0	28	R.AIDKPFLLPIEDVFSISGR.G

No match to: 723.3202, 725.3349, 790.4618, 804.3175, 832.3504, 848.3077, 855.0634, 861.0816, 877.0593, 879.0642, 893.0384, 897.0395, 930.4819, 941.5245, 965.5432, 985.5503, 1001.5439, 1046.5846, 1179.6348, 1193.6685, 1219.5995, 1255.6486, 1269.6710, 1271.6285, 1277.6571, 1296.7322, 1320.6617, 1326.7448, 1348.7190, 1368.7612, 1450.7841, 1472.8880, 1475.8216, 1477.7796, 1494.8828, 1497.7996, 1509.8403, 1511.9038, 1527.8609, 1550.8947, 1598.9195, 1617.8806, 1631.8934, 1654.9031, 1707.9342, 1712.9042, 1733.0067, 1749.9856, 1755.9948, 1818.0212, 1866.0850, 1898.0605, 1915.0945, 1931.0593, 1947.0781, 1949.0662, 1967.1856, 1981.0932, 1988.1120, 1994.1406, 2005.1346, 2010.0916, 2139.2472, 2211.2249, 2229.2467, 2233.2426, 2271.2633, 2384.1999, 2399.2428, 2439.4144, 2451.4028, 2498.4046, 2615.4719, 2705.3847, 2808.6002, 3284.5981

10. EFTU2_ECO24 **Mass:** 43456 **Score:** 138 **Expect:** 5.3e-09 **Matches:** 11

Elongation factor Tu 2 OS=Escherichia coli O139:H28 (strain E24377A / ETEC) OX=331111 GN=tuf2 PE=3 SV=1

Observed	Mr(expt)	Mr(calc)	ppm	Start	End	Miss	Ions	Peptide
837.5072	836.4999	836.4868	15.7	118	124	0	---	R.EHILLGR.Q
1027.6166	1026.6093	1026.5822	26.4	271	280	0	---	R.AGENVGVLLR.G
1214.6581	1213.6508	1213.6230	22.9	305	314	0	---	K.FESEVYILSK.D
1233.6701	1232.6629	1232.6091	43.6	326	334	0	15	K.GYRPQFYFR.T
1315.7305	1314.7232	1314.6060	89.1	254	264	1	---	K.STCTGVEMFRK.L
1574.9723	1573.9650	1573.9304	22.0	290	304	0	---	R.GQVLAKPGTIKPHTK.F
1728.9325	1727.9253	1727.8366	51.3	305	319	1	---	K.FESEVYILSKDEGGR.H
1797.0350	1796.0277	1795.9325	53.1	359	374	0	---	K.MVVTLIHPIAMDDGLR.F + Oxidation (M)
1803.9918	1802.9845	1802.8799	58.0	60	75	0	---	R.GITINTSHVEYDTPTR.H
1965.1206	1964.1134	1963.9527	81.8	156	172	0	44	R.ELLSQYDFPGDDTPIVR.G
2117.3190	2116.3117	2116.1568	73.2	206	224	0	28	R.AIDKPFLLPIEDVFSISGR.G

No match to: 723.3202, 725.3349, 790.4618, 804.3175, 832.3504, 848.3077, 855.0634, 861.0816, 877.0593, 879.0642, 893.0384, 897.0395, 930.4819, 941.5245, 965.5432, 985.5503, 1001.5439, 1046.5846, 1179.6348, 1193.6685, 1219.5995, 1255.6486, 1269.6710, 1271.6285, 1277.6571, 1296.7322, 1320.6617, 1326.7448, 1348.7190, 1368.7612, 1450.7841, 1472.8880, 1475.8216, 1477.7796, 1494.8828, 1497.7996, 1509.8403, 1511.9038, 1527.8609, 1550.8947, 1598.9195, 1617.8806, 1631.8934, 1654.9031, 1707.9342, 1712.9042, 1733.0067, 1749.9856, 1755.9948, 1818.0212, 1866.0850, 1898.0605, 1915.0945, 1931.0593, 1947.0781, 1949.0662, 1967.1856, 1981.0932, 1988.1120, 1994.1406, 2005.1346, 2010.0916, 2139.2472, 2211.2249, 2229.2467, 2233.2426, 2271.2633, 2384.1999, 2399.2428, 2439.4144, 2451.4028, 2498.4046, 2615.4719, 2705.3847, 2808.6002, 3284.5981

11. EFTU2_ECOHS **Mass:** 43457 **Score:** 138 **Expect:** 5.3e-09 **Matches:** 11
Elongation factor Tu 2 OS=Escherichia coli O9:H4 (strain HS) OX=331112 GN=tuf2 PE=3 SV=1

Observed	Mr(expt)	Mr(calc)	ppm	Start	End	Miss	Ions	Peptide
837.5072	836.4999	836.4868	15.7	118 -	124	0	---	R.EHILLGR.Q
1027.6166	1026.6093	1026.5822	26.4	271 -	280	0	---	R.AGENVGVLLR.G
1214.6581	1213.6508	1213.6230	22.9	305 -	314	0	---	K.FESEVYILSK.D
1233.6701	1232.6629	1232.6091	43.6	326 -	334	0	15	K.GYRPQFYFR.T
1315.7305	1314.7232	1314.6060	89.1	254 -	264	1	---	K.STCTGVEMFRK.L
1574.9723	1573.9650	1573.9304	22.0	290 -	304	0	---	R.GQVLAKPGTIKPHTK.F
1728.9325	1727.9253	1727.8366	51.3	305 -	319	1	---	K.FESEVYILSKDEGGR.H
1797.0350	1796.0277	1795.9325	53.1	359 -	374	0	---	K.MVVTLIHPIAMDDGLR.F + Oxidation (M)
1803.9918	1802.9845	1802.8799	58.0	60 -	75	0	---	R.GITINTSHVEYDTPTR.H
1965.1206	1964.1134	1963.9527	81.8	156 -	172	0	44	R.ELLSQYDFPGDDTPIVR.G
2117.3190	2116.3117	2116.1568	73.2	206 -	224	0	28	R.AIDKPFLLPIEDVFSISGR.G

No match to: 723.3202, 725.3349, 790.4618, 804.3175, 832.3504, 848.3077, 855.0634, 861.0816, 877.0593, 879.0642, 893.0384, 897.0395, 930.4819, 941.5245, 965.5432, 985.5503, 1001.5439, 1046.5846, 1179.6348, 1193.6685, 1219.5995, 1255.6486, 1269.6710, 1271.6285, 1277.6571, 1296.7322, 1320.6617, 1326.7448, 1348.7190, 1368.7612, 1450.7841, 1472.8880, 1475.8216, 1477.7796, 1494.8828, 1497.7996, 1509.8403, 1511.9038, 1527.8609, 1550.8947, 1598.9195, 1617.8806, 1631.8934, 1654.9031, 1707.9342, 1712.9042, 1733.0067, 1749.9856, 1755.9948, 1818.0212, 1866.0850, 1898.0605, 1915.0945, 1931.0593, 1947.0781, 1949.0662, 1967.1856, 1981.0932, 1988.1120, 1994.1406, 2005.1346, 2010.0916, 2139.2472, 2211.2249, 2229.2467, 2233.2426, 2271.2633, 2384.1999, 2399.2428, 2439.4144, 2451.4028, 2498.4046, 2615.4719, 2705.3847, 2808.6002, 3284.5981

12. EFTU2_ECOK1 **Mass:** 43457 **Score:** 138 **Expect:** 5.3e-09 **Matches:** 11
Elongation factor Tu 2 OS=Escherichia coli O1:K1 / APEC OX=405955 GN=tuf2 PE=3 SV=1

Observed	Mr(expt)	Mr(calc)	ppm	Start	End	Miss	Ions	Peptide
837.5072	836.4999	836.4868	15.7	118 -	124	0	---	R.EHILLGR.Q
1027.6166	1026.6093	1026.5822	26.4	271 -	280	0	---	R.AGENVGVLLR.G
1214.6581	1213.6508	1213.6230	22.9	305 -	314	0	---	K.FESEVYILSK.D
1233.6701	1232.6629	1232.6091	43.6	326 -	334	0	15	K.GYRPQFYFR.T
1315.7305	1314.7232	1314.6060	89.1	254 -	264	1	---	K.STCTGVEMFRK.L
1574.9723	1573.9650	1573.9304	22.0	290 -	304	0	---	R.GQVLAKPGTIKPHTK.F
1728.9325	1727.9253	1727.8366	51.3	305 -	319	1	---	K.FESEVYILSKDEGGR.H
1797.0350	1796.0277	1795.9325	53.1	359 -	374	0	---	K.MVVTLIHPIAMDDGLR.F + Oxidation (M)
1803.9918	1802.9845	1802.8799	58.0	60 -	75	0	---	R.GITINTSHVEYDTPTR.H
1965.1206	1964.1134	1963.9527	81.8	156 -	172	0	44	R.ELLSQYDFPGDDTPIVR.G
2117.3190	2116.3117	2116.1568	73.2	206 -	224	0	28	R.AIDKPFLLPIEDVFSISGR.G

No match to: 723.3202, 725.3349, 790.4618, 804.3175, 832.3504, 848.3077, 855.0634, 861.0816, 877.0593, 879.0642, 893.0384, 897.0395, 930.4819, 941.5245, 965.5432, 985.5503, 1001.5439, 1046.5846, 1179.6348, 1193.6685, 1219.5995, 1255.6486, 1269.6710, 1271.6285, 1277.6571, 1296.7322, 1320.6617, 1326.7448, 1348.7190, 1368.7612, 1450.7841, 1472.8880, 1475.8216, 1477.7796, 1494.8828, 1497.7996, 1509.8403, 1511.9038, 1527.8609, 1550.8947, 1598.9195, 1617.8806, 1631.8934, 1654.9031, 1707.9342, 1712.9042, 1733.0067, 1749.9856, 1755.9948, 1818.0212, 1866.0850, 1898.0605, 1915.0945, 1931.0593, 1947.0781, 1949.0662, 1967.1856, 1981.0932, 1988.1120, 1994.1406, 2005.1346, 2010.0916, 2139.2472, 2211.2249, 2229.2467, 2233.2426, 2271.2633, 2384.1999, 2399.2428, 2439.4144, 2451.4028, 2498.4046, 2615.4719, 2705.3847, 2808.6002, 3284.5981

13. EFTU2_ECOL5 **Mass:** 43457 **Score:** 138 **Expect:** 5.3e-09 **Matches:** 11
Elongation factor Tu 2 OS=Escherichia coli O6:K15:H31 (strain 536 / UPEC) OX=362663 GN=tuf2 PE=3 SV=1

Observed	Mr(expt)	Mr(calc)	ppm	Start	End	Miss	Ions	Peptide
837.5072	836.4999	836.4868	15.7	118 -	124	0	---	R.EHILLGR.Q
1027.6166	1026.6093	1026.5822	26.4	271 -	280	0	---	R.AGENVGVLLR.G
1214.6581	1213.6508	1213.6230	22.9	305 -	314	0	---	K.FESEVYILSK.D
1233.6701	1232.6629	1232.6091	43.6	326 -	334	0	15	K.GYRPQFYFR.T
1315.7305	1314.7232	1314.6060	89.1	254 -	264	1	---	K.STCTGVEMFRK.L
1574.9723	1573.9650	1573.9304	22.0	290 -	304	0	---	R.GQVLAKPGTIKPHTK.F
1728.9325	1727.9253	1727.8366	51.3	305 -	319	1	---	K.FESEVYILSKDEGGR.H
1797.0350	1796.0277	1795.9325	53.1	359 -	374	0	---	K.MVVTLIHPIAMDDGLR.F + Oxidation (M)
1803.9918	1802.9845	1802.8799	58.0	60 -	75	0	---	R.GITINTSHVEYDTPTR.H
1965.1206	1964.1134	1963.9527	81.8	156 -	172	0	44	R.ELLSQYDFPGDDTPIVR.G
2117.3190	2116.3117	2116.1568	73.2	206 -	224	0	28	R.AIDKPFLLPIEDVFSISGR.G

No match to: 723.3202, 725.3349, 790.4618, 804.3175, 832.3504, 848.3077, 855.0634, 861.0816, 877.0593, 879.0642, 893.0384, 897.0395, 930.4819, 941.5245, 965.5432, 985.5503, 1001.5439, 1046.5846, 1179.6348, 1193.6685, 1219.5995, 1255.6486, 1269.6710, 1271.6285, 1277.6571, 1296.7322, 1320.6617, 1326.7448, 1348.7190, 1368.7612, 1450.7841, 1472.8880, 1475.8216, 1477.7796, 1494.8828, 1497.7996, 1509.8403, 1511.9038, 1527.8609, 1550.8947, 1598.9195, 1617.8806, 1631.8934, 1654.9031, 1707.9342, 1712.9042, 1733.0067, 1749.9856, 1755.9948, 1818.0212, 1866.0850, 1898.0605, 1915.0945, 1931.0593, 1947.0781, 1949.0662, 1967.1856, 1981.0932, 1988.1120, 1994.1406, 2005.1346, 2010.0916, 2139.2472, 2211.2249, 2229.2467, 2233.2426, 2271.2633, 2384.1999, 2399.2428, 2439.4144, 2451.4028, 2498.4046, 2615.4719, 2705.3847, 2808.6002, 3284.5981

14. EFTU2_ECOLC **Mass:** 43457 **Score:** 138 **Expect:** 5.3e-09 **Matches:** 11
Elongation factor Tu 2 OS=Escherichia coli (strain ATCC 8739 / DSM 1576 / Crooks) OX=481805 GN=tuf2 PE=3 SV=1

Observed	Mr(expt)	Mr(calc)	ppm	Start	End	Miss	Ions	Peptide
837.5072	836.4999	836.4868	15.7	118 -	124	0	---	R.EHILLGR.Q
1027.6166	1026.6093	1026.5822	26.4	271 -	280	0	---	R.AGENVGVLLR.G
1214.6581	1213.6508	1213.6230	22.9	305 -	314	0	---	K.FESEVYILSK.D
1233.6701	1232.6629	1232.6091	43.6	326 -	334	0	15	K.GYRPQFYFR.T
1315.7305	1314.7232	1314.6060	89.1	254 -	264	1	---	K.STCTGVEMFRK.L
1574.9723	1573.9650	1573.9304	22.0	290 -	304	0	---	R.GQVLAKPGTIKPHTK.F
1728.9325	1727.9253	1727.8366	51.3	305 -	319	1	---	K.FESEVYILSKDEGGR.H
1797.0350	1796.0277	1795.9325	53.1	359 -	374	0	---	K.MVVTLIHPIAMDDGLR.F + Oxidation (M)
1803.9918	1802.9845	1802.8799	58.0	60 -	75	0	---	R.GITINTSHVEYDTPTR.H
1965.1206	1964.1134	1963.9527	81.8	156 -	172	0	44	R.ELLSQYDFPGDDTPIVR.G
2117.3190	2116.3117	2116.1568	73.2	206 -	224	0	28	R.AIDKPFLLPIEDVFSISGR.G

No match to: 723.3202, 725.3349, 790.4618, 804.3175, 832.3504, 848.3077, 855.0634, 861.0816, 877.0593, 879.0642, 893.0384, 897.0395, 930.4819, 941.5245, 965.5432, 985.5503, 1001.5439, 1046.5846, 1179.6348, 1193.6685, 1219.5995, 1255.6486, 1269.6710, 1271.6285, 1277.6571, 1296.7322, 1320.6617, 1326.7448, 1348.7190, 1368.7612, 1450.7841, 1472.8880, 1475.8216, 1477.7796, 1494.8828, 1497.7996, 1509.8403, 1511.9038, 1527.8609, 1550.8947, 1598.9195, 1617.8806, 1631.8934, 1654.9031, 1707.9342, 1712.9042, 1733.0067, 1749.9856, 1755.9948, 1818.0212, 1866.0850, 1898.0605, 1915.0945, 1931.0593, 1947.0781, 1949.0662, 1967.1856, 1981.0932, 1988.1120, 1994.1406, 2005.1346, 2010.0916, 2139.2472, 2211.2249, 2229.2467, 2233.2426, 2271.2633, 2384.1999, 2399.2428, 2439.4144, 2451.4028, 2498.4046, 2615.4719, 2705.3847, 2808.6002, 3284.5981

15. EFTU2_ECOLI **Mass:** 43457 **Score:** 138 **Expect:** 5.3e-09 **Matches:** 11

Elongation factor Tu 2 OS=Escherichia coli (strain K12) OX=83333 GN=tufB PE=1 SV=1

Observed	Mr(expt)	Mr(calc)	ppm	Start - End	Miss	Ions	Peptide
837.5072	836.4999	836.4868	15.7	118 - 124	0	---	R.EHILLGR.Q
1027.6166	1026.6093	1026.5822	26.4	271 - 280	0	---	R.AGENVGVLLR.G
1214.6581	1213.6508	1213.6230	22.9	305 - 314	0	---	K.FESEVYILSK.D
1233.6701	1232.6629	1232.6091	43.6	326 - 334	0	15	K.GYRPQFYFR.T
1315.7305	1314.7232	1314.6060	89.1	254 - 264	1	---	K.STCTGVEMFRK.L
1574.9723	1573.9650	1573.9304	22.0	290 - 304	0	---	R.GQVLAKPGTIKPHTK.F
1728.9325	1727.9253	1727.8366	51.3	305 - 319	1	---	K.FESEVYILSKDEGGR.H
1797.0350	1796.0277	1795.9325	53.1	359 - 374	0	---	K.MVVTLIHPIAMDDGLR.F + Oxidation (M)
1803.9918	1802.9845	1802.8799	58.0	60 - 75	0	---	R.GITINTSHVEYDTPTR.H
1965.1206	1964.1134	1963.9527	81.8	156 - 172	0	44	R.ELLSQYDFPGDDTPIVR.G
2117.3190	2116.3117	2116.1568	73.2	206 - 224	0	28	R.AIDKPFLLPIEDVFSISGR.G

No match to: 723.3202, 725.3349, 790.4618, 804.3175, 832.3504, 848.3077, 855.0634, 861.0816, 877.0593, 879.0642, 893.0384, 897.0395, 930.4819, 941.5245, 965.5432, 985.5503, 1001.5439, 1046.5846, 1179.6348, 1193.6685, 1219.5995, 1255.6486, 1269.6710, 1271.6285, 1277.6571, 1296.7322, 1320.6617, 1326.7448, 1348.7190, 1368.7612, 1450.7841, 1472.8880, 1475.8216, 1477.7796, 1494.8828, 1497.7996, 1509.8403, 1511.9038, 1527.8609, 1550.8947, 1598.9195, 1617.8806, 1631.8934, 1654.9031, 1707.9342, 1712.9042, 1733.0067, 1749.9856, 1755.9948, 1818.0212, 1866.0850, 1898.0605, 1915.0945, 1931.0593, 1947.0781, 1949.0662, 1967.1856, 1981.0932, 1988.1120, 1994.1406, 2005.1346, 2010.0916, 2139.2472, 2211.2249, 2229.2467, 2233.2426, 2271.2633, 2384.1999, 2399.2428, 2439.4144, 2451.4028, 2498.4046, 2615.4719, 2705.3847, 2808.6002, 3284.5981

16. EFTU2_ECOUT **Mass:** 43457 **Score:** 138 **Expect:** 5.3e-09 **Matches:** 11

Elongation factor Tu 2 OS=Escherichia coli (strain UTI89 / UPEC) OX=364106 GN=tuf2 PE=3 SV=2

Observed	Mr(expt)	Mr(calc)	ppm	Start - End	Miss	Ions	Peptide
837.5072	836.4999	836.4868	15.7	118 - 124	0	---	R.EHILLGR.Q
1027.6166	1026.6093	1026.5822	26.4	271 - 280	0	---	R.AGENVGVLLR.G
1214.6581	1213.6508	1213.6230	22.9	305 - 314	0	---	K.FESEVYILSK.D
1233.6701	1232.6629	1232.6091	43.6	326 - 334	0	15	K.GYRPQFYFR.T
1315.7305	1314.7232	1314.6060	89.1	254 - 264	1	---	K.STCTGVEMFRK.L
1574.9723	1573.9650	1573.9304	22.0	290 - 304	0	---	R.GQVLAKPGTIKPHTK.F
1728.9325	1727.9253	1727.8366	51.3	305 - 319	1	---	K.FESEVYILSKDEGGR.H
1797.0350	1796.0277	1795.9325	53.1	359 - 374	0	---	K.MVVTLIHPIAMDDGLR.F + Oxidation (M)
1803.9918	1802.9845	1802.8799	58.0	60 - 75	0	---	R.GITINTSHVEYDTPTR.H
1965.1206	1964.1134	1963.9527	81.8	156 - 172	0	44	R.ELLSQYDFPGDDTPIVR.G
2117.3190	2116.3117	2116.1568	73.2	206 - 224	0	28	R.AIDKPFLLPIEDVFSISGR.G

No match to: 723.3202, 725.3349, 790.4618, 804.3175, 832.3504, 848.3077, 855.0634, 861.0816, 877.0593, 879.0642, 893.0384, 897.0395, 930.4819, 941.5245, 965.5432, 985.5503, 1001.5439, 1046.5846, 1179.6348, 1193.6685, 1219.5995, 1255.6486, 1269.6710, 1271.6285, 1277.6571, 1296.7322, 1320.6617, 1326.7448, 1348.7190, 1368.7612, 1450.7841, 1472.8880, 1475.8216, 1477.7796, 1494.8828, 1497.7996, 1509.8403, 1511.9038, 1527.8609, 1550.8947, 1598.9195, 1617.8806, 1631.8934, 1654.9031, 1707.9342, 1712.9042, 1733.0067, 1749.9856, 1755.9948, 1818.0212, 1866.0850, 1898.0605, 1915.0945, 1931.0593, 1947.0781, 1949.0662, 1967.1856, 1981.0932, 1988.1120, 1994.1406, 2005.1346, 2010.0916, 2139.2472, 2211.2249, 2229.2467, 2233.2426, 2271.2633, 2384.1999, 2399.2428, 2439.4144, 2451.4028, 2498.4046, 2615.4719, 2705.3847, 2808.6002, 3284.5981

17. EFTU2_SHIF8 **Mass:** 43457 **Score:** 138 **Expect:** 5.3e-09 **Matches:** 11

Elongation factor Tu 2 OS=Shigella flexneri serotype 5b (strain 8401) OX=373384 GN=tuf2 PE=3 SV=1

Observed	Mr(expt)	Mr(calc)	ppm	Start - End	Miss	Ions	Peptide
837.5072	836.4999	836.4868	15.7	118 - 124	0	---	R.EHILLGR.Q
1027.6166	1026.6093	1026.5822	26.4	271 - 280	0	---	R.AGENVGVLLR.G
1214.6581	1213.6508	1213.6230	22.9	305 - 314	0	---	K.FESEVYILSK.D
1233.6701	1232.6629	1232.6091	43.6	326 - 334	0	15	K.GYRPQFYFR.T
1315.7305	1314.7232	1314.6060	89.1	254 - 264	1	---	K.STCTGVEMFRK.L
1574.9723	1573.9650	1573.9304	22.0	290 - 304	0	---	R.GQVLAKPGTIKPHTK.F
1728.9325	1727.9253	1727.8366	51.3	305 - 319	1	---	K.FESEVYILSKDEGGR.H
1797.0350	1796.0277	1795.9325	53.1	359 - 374	0	---	K.MVVTLIHPIAMDDGLR.F + Oxidation (M)
1803.9918	1802.9845	1802.8799	58.0	60 - 75	0	---	R.GITINTSHVEYDTPTR.H
1965.1206	1964.1134	1963.9527	81.8	156 - 172	0	44	R.ELLSQYDFPGDDTPIVR.G
2117.3190	2116.3117	2116.1568	73.2	206 - 224	0	28	R.AIDKPFLLPIEDVFSISGR.G

No match to: 723.3202, 725.3349, 790.4618, 804.3175, 832.3504, 848.3077, 855.0634, 861.0816, 877.0593, 879.0642, 893.0384, 897.0395, 930.4819, 941.5245, 965.5432, 985.5503, 1001.5439, 1046.5846, 1179.6348, 1193.6685, 1219.5995, 1255.6486, 1269.6710, 1271.6285, 1277.6571, 1296.7322, 1320.6617, 1326.7448, 1348.7190, 1368.7612, 1450.7841, 1472.8880, 1475.8216, 1477.7796, 1494.8828, 1497.7996, 1509.8403, 1511.9038, 1527.8609, 1550.8947, 1598.9195, 1617.8806, 1631.8934, 1654.9031, 1707.9342, 1712.9042, 1733.0067, 1749.9856, 1755.9948, 1818.0212, 1866.0850, 1898.0605, 1915.0945, 1931.0593, 1947.0781, 1949.0662, 1967.1856, 1981.0932, 1988.1120, 1994.1406, 2005.1346, 2010.0916, 2139.2472, 2211.2249, 2229.2467, 2233.2426, 2271.2633, 2384.1999, 2399.2428, 2439.4144, 2451.4028, 2498.4046, 2615.4719, 2705.3847, 2808.6002, 3284.5981

18. EFTU2_SHISS **Mass:** 43457 **Score:** 138 **Expect:** 5.3e-09 **Matches:** 11

Elongation factor Tu 2 OS=Shigella sonnei (strain Ss046) OX=300269 GN=tuf2 PE=3 SV=1

Observed	Mr(expt)	Mr(calc)	ppm	Start - End	Miss	Ions	Peptide
837.5072	836.4999	836.4868	15.7	118 - 124	0	---	R.EHILLGR.Q
1027.6166	1026.6093	1026.5822	26.4	271 - 280	0	---	R.AGENVGVLLR.G
1214.6581	1213.6508	1213.6230	22.9	305 - 314	0	---	K.FESEVYILSK.D
1233.6701	1232.6629	1232.6091	43.6	326 - 334	0	15	K.GYRPQFYFR.T
1315.7305	1314.7232	1314.6060	89.1	254 - 264	1	---	K.STCTGVEMFRK.L
1574.9723	1573.9650	1573.9304	22.0	290 - 304	0	---	R.GQVLAKPGTIKPHTK.F
1728.9325	1727.9253	1727.8366	51.3	305 - 319	1	---	K.FESEVYILSKDEGGR.H
1797.0350	1796.0277	1795.9325	53.1	359 - 374	0	---	K.MVVTLIHPIAMDDGLR.F + Oxidation (M)
1803.9918	1802.9845	1802.8799	58.0	60 - 75	0	---	R.GITINTSHVEYDTPTR.H
1965.1206	1964.1134	1963.9527	81.8	156 - 172	0	44	R.ELLSQYDFPGDDTPIVR.G
2117.3190	2116.3117	2116.1568	73.2	206 - 224	0	28	R.AIDKPFLLPIEDVFSISGR.G

No match to: 723.3202, 725.3349, 790.4618, 804.3175, 832.3504, 848.3077, 855.0634, 861.0816, 877.0593, 879.0642, 893.0384, 897.0395, 930.4819, 941.5245, 965.5432, 985.5503, 1001.5439, 1046.5846, 1179.6348, 1193.6685, 1219.5995, 1255.6486, 1269.6710, 1271.6285, 1277.6571, 1296.7322, 1320.6617, 1326.7448, 1348.7190, 1368.7612, 1450.7841, 1472.8880, 1475.8216, 1477.7796, 1494.8828, 1497.7996, 1509.8403, 1511.9038, 1527.8609, 1550.8947, 1598.9195, 1617.8806, 1631.8934, 1654.9031, 1707.9342, 1712.9042, 1733.0067, 1749.9856, 1755.9948, 1818.0212, 1866.0850, 1898.0605, 1915.0945, 1931.0593, 1947.0781, 1949.0662, 1967.1856, 1981.0932, 1988.1120, 1994.1406, 2005.1346, 2010.0916, 2139.2472, 2211.2249, 2229.2467, 2233.2426, 2271.2633, 2384.1999, 2399.2428, 2439.4144, 2451.4028, 2498.4046, 2615.4719, 2705.3847, 2808.6002, 3284.5981

19. EFTU_ECO57 **Mass:** 43457 **Score:** 138 **Expect:** 5.3e-09 **Matches:** 11

Elongation factor Tu OS=Escherichia coli O157:H7 OX=83334 GN=tufA PE=3 SV=2

Observed	Mr(expt)	Mr(calc)	ppm	Start - End	Miss	Ions	Peptide
837.5072	836.4999	836.4868	15.7	118 - 124	0	---	R.EHILLGR.Q
1027.6166	1026.6093	1026.5822	26.4	271 - 280	0	---	R.AGENVGVLLR.G

1214.6581	1213.6508	1213.6230	22.9	305	314	0	---	K.FESEVYILSK.D
1233.6701	1232.6629	1232.6091	43.6	326	334	0	15	K.GYRPQFYFR.T
1315.7305	1314.7232	1314.6060	89.1	254	264	1	---	K.STCTGVEMFRK.L
1574.9723	1573.9650	1573.9304	22.0	290	304	0	---	R.GQVLAKPGTIKPHTK.F
1728.9325	1727.9253	1727.8366	51.3	305	319	1	---	K.FESEVYILSKDEGGR.H
1797.0350	1796.0277	1795.9325	53.1	359	374	0	---	K.MVVTLIHPIAMDDGLR.F + Oxidation (M)
1803.9918	1802.9845	1802.8799	58.0	60	75	0	---	R.GITINTSHVEYDTPTR.H
1965.1206	1964.1134	1963.9527	81.8	156	172	0	44	R.ELLSQYDFPGDDTPIVR.G
2117.3190	2116.3117	2116.1568	73.2	206	224	0	28	R.AIDKPFLLPIEDVFSISGR.G

No match to: 723.3202, 725.3349, 790.4618, 804.3175, 832.3504, 848.3077, 855.0634, 861.0816, 877.0593, 879.0642, 893.0384, 897.0395, 930.4819, 941.5245, 965.5432, 985.5503, 1001.5439, 1046.5846, 1179.6348, 1193.6685, 1219.5995, 1255.6486, 1269.6710, 1271.6285, 1277.6571, 1296.7322, 1320.6617, 1326.7448, 1348.7190, 1368.7612, 1450.7841, 1472.8880, 1475.8216, 1477.7796, 1494.8828, 1497.7996, 1509.8403, 1511.9038, 1527.8609, 1550.8947, 1598.9195, 1617.8806, 1631.8934, 1654.9031, 1707.9342, 1712.9042, 1733.0067, 1749.9856, 1755.9948, 1818.0212, 1866.0850, 1898.0605, 1915.0945, 1931.0593, 1947.0781, 1949.0662, 1967.1856, 1981.0932, 1988.1120, 1994.1406, 2005.1346, 2010.0916, 2139.2472, 2211.2249, 2229.2467, 2233.2426, 2271.2633, 2384.1999, 2399.2428, 2439.4144, 2451.4028, 2498.4046, 2615.4719, 2705.3847, 2808.6002, 3284.5981

20. EFTU_ECOL6 **Mass:** 43457 **Score:** 138 **Expect:** 5.3e-09 **Matches:** 11

Elongation factor Tu OS=Escherichia coli O6:H1 (strain CFT073 / ATCC 700928 / UPEC) OX=199310 GN=tufA PE=3 SV=2

Observed	Mr(expt)	Mr(calc)	ppm	Start	End	Miss	Ions	Peptide
837.5072	836.4999	836.4868	15.7	118	124	0	---	R.EHILLGR.Q
1027.6166	1026.6093	1026.5822	26.4	271	280	0	---	R.AGENVGVLLR.G
1214.6581	1213.6508	1213.6230	22.9	305	314	0	---	K.FESEVYILSK.D
1233.6701	1232.6629	1232.6091	43.6	326	334	0	15	K.GYRPQFYFR.T
1315.7305	1314.7232	1314.6060	89.1	254	264	1	---	K.STCTGVEMFRK.L
1574.9723	1573.9650	1573.9304	22.0	290	304	0	---	R.GQVLAKPGTIKPHTK.F
1728.9325	1727.9253	1727.8366	51.3	305	319	1	---	K.FESEVYILSKDEGGR.H
1797.0350	1796.0277	1795.9325	53.1	359	374	0	---	K.MVVTLIHPIAMDDGLR.F + Oxidation (M)
1803.9918	1802.9845	1802.8799	58.0	60	75	0	---	R.GITINTSHVEYDTPTR.H
1965.1206	1964.1134	1963.9527	81.8	156	172	0	44	R.ELLSQYDFPGDDTPIVR.G
2117.3190	2116.3117	2116.1568	73.2	206	224	0	28	R.AIDKPFLLPIEDVFSISGR.G

No match to: 723.3202, 725.3349, 790.4618, 804.3175, 832.3504, 848.3077, 855.0634, 861.0816, 877.0593, 879.0642, 893.0384, 897.0395, 930.4819, 941.5245, 965.5432, 985.5503, 1001.5439, 1046.5846, 1179.6348, 1193.6685, 1219.5995, 1255.6486, 1269.6710, 1271.6285, 1277.6571, 1296.7322, 1320.6617, 1326.7448, 1348.7190, 1368.7612, 1450.7841, 1472.8880, 1475.8216, 1477.7796, 1494.8828, 1497.7996, 1509.8403, 1511.9038, 1527.8609, 1550.8947, 1598.9195, 1617.8806, 1631.8934, 1654.9031, 1707.9342, 1712.9042, 1733.0067, 1749.9856, 1755.9948, 1818.0212, 1866.0850, 1898.0605, 1915.0945, 1931.0593, 1947.0781, 1949.0662, 1967.1856, 1981.0932, 1988.1120, 1994.1406, 2005.1346, 2010.0916, 2139.2472, 2211.2249, 2229.2467, 2233.2426, 2271.2633, 2384.1999, 2399.2428, 2439.4144, 2451.4028, 2498.4046, 2615.4719, 2705.3847, 2808.6002, 3284.5981

Search Parameters

Type of search : MS/MS Ion Search
Enzyme : Trypsin
Fixed modifications : Carbamidomethyl (C)
Variable modifications : Oxidation (M)
Mass values : Monoisotopic
Protein Mass : Unrestricted
Peptide Mass Tolerance : ± 150 ppm
Fragment Mass Tolerance: ± 2 Da
Max Missed Cleavages : 1
Instrument type : MALDI-TOF-TOF
Query1 (723.3202,1+): <no title>
Query2 (725.3349,1+): <no title>
Query3 (790.4618,1+): <no title>
Query4 (804.3175,1+): <no title>
Query5 (832.3504,1+): <no title>
Query6 (837.5072,1+): <no title>
Query7 (848.3077,1+): <no title>
Query8 (855.0634,1+): <no title>
Query9 (861.0816,1+): <no title>
Query10 (877.0593,1+): <no title>
Query11 (879.0642,1+): <no title>
Query12 (893.0384,1+): <no title>
Query13 (897.0395,1+): <no title>
Query14 (930.4819,1+): <no title>
Query15 (941.5245,1+): <no title>
Query16 (965.5432,1+): <no title>
Query17 (985.5503,1+): <no title>
Query18 (1001.5439,1+): <no title>
Query19 (1027.6166,1+): <no title>
Query20 (1046.5846,1+): <no title>
Query21 (1179.6348,1+): <no title>
Query22 (1193.6685,1+): <no title>
Query23 (1214.6581,1+): <no title>
Query24 (1219.5995,1+): <no title>
Query25 (1233.6701,1+): <no title>
Query26 (1255.6486,1+): <no title>
Query27 (1269.6710,1+): <no title>
Query28 (1271.6285,1+): <no title>
Query29 (1277.6571,1+): <no title>
Query30 (1296.7322,1+): <no title>
Query31 (1315.7305,1+): <no title>
Query32 (1320.6617,1+): <no title>
Query33 (1326.7448,1+): <no title>
Query34 (1348.7190,1+): <no title>
Query35 (1368.7612,1+): <no title>
Query36 (1450.7841,1+): <no title>
Query37 (1472.8880,1+): <no title>
Query38 (1475.8216,1+): <no title>
Query39 (1477.7796,1+): <no title>
Query40 (1494.8828,1+): <no title>
Query41 (1497.7996,1+): <no title>
Query42 (1509.8403,1+): <no title>
Query43 (1511.9038,1+): <no title>
Query44 (1527.8609,1+): <no title>
Query45 (1550.8947,1+): <no title>
Query46 (1574.9723,1+): <no title>
Query47 (1598.9195,1+): <no title>
Query48 (1617.8806,1+): <no title>

```
Query49 (1631.8934,1+): <no title>
Query50 (1654.9031,1+): <no title>
Query51 (1707.9342,1+): <no title>
Query52 (1712.9042,1+): <no title>
Query53 (1728.9325,1+): <no title>
Query54 (1733.0067,1+): <no title>
Query55 (1749.9856,1+): <no title>
Query56 (1755.9948,1+): <no title>
Query57 (1797.0350,1+): <no title>
Query58 (1803.9918,1+): <no title>
Query59 (1818.0212,1+): <no title>
Query60 (1866.0850,1+): <no title>
Query61 (1898.0605,1+): <no title>
Query62 (1915.0945,1+): <no title>
Query63 (1931.0593,1+): <no title>
Query64 (1947.0781,1+): <no title>
Query65 (1949.0662,1+): <no title>
Query66 (1965.1206,1+): <no title>
Query67 (1967.1856,1+): <no title>
Query68 (1981.0932,1+): <no title>
Query69 (1988.1120,1+): <no title>
Query70 (1994.1406,1+): <no title>
Query71 (2005.1346,1+): <no title>
Query72 (2010.0916,1+): <no title>
Query73 (2117.3190,1+): <no title>
Query74 (2139.2472,1+): <no title>
Query75 (2211.2249,1+): <no title>
Query76 (2229.2467,1+): <no title>
Query77 (2233.2426,1+): <no title>
Query78 (2271.2633,1+): <no title>
Query79 (2384.1999,1+): <no title>
Query80 (2399.2428,1+): <no title>
Query81 (2439.4144,1+): <no title>
Query82 (2451.4028,1+): <no title>
Query83 (2498.4046,1+): <no title>
Query84 (2615.4719,1+): <no title>
Query85 (2705.3847,1+): <no title>
Query86 (2808.6002,1+): <no title>
Query87 (3284.5981,1+): <no title>
```

Mascot: http://www.matrixscience.com/

MALDI MS MS Data Sheet For Blood Isolate

C:\Users\madhurekha\Desktop\MALDI_Data\16th batch data 070718\BHU\2\0_B2\1\1075.6476.LIFT\1SRef

Comment 1

Comment 2

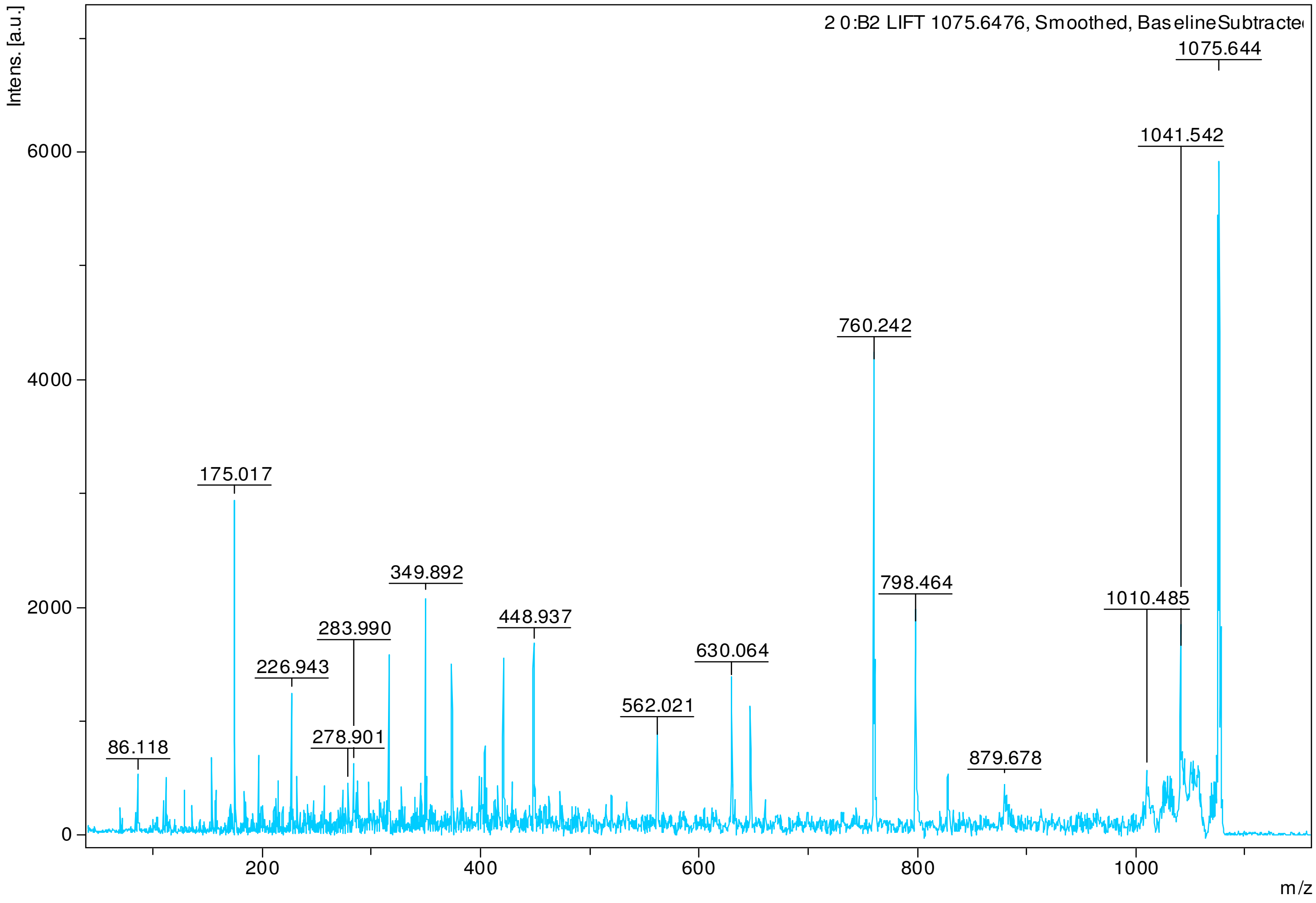

m/z	S/N	Quality Fac.	Res.	Intens.	Area
70.067	6.5	315	354	271.28	59
72.109	3.7	166	282	154.67	43
86.118	12.0	3410	385	539.50	133
104.032	3.3	133	629	153.14	28
110.020	6.5	1427	597	303.61	63
112.061	10.5	1726	547	490.46	113
115.079	3.1	51	763	147.28	25
129.058	8.3	1795	701	392.29	82
136.008	5.3	587	754	259.11	53
153.994	14.1	3801	936	694.28	131
158.014	8.1	1113	940	404.06	78
169.011	3.0	74	770	156.49	40
170.998	5.2	61	827	270.92	65
175.017	55.8	31800	944	2960.91	642
183.957	7.2	123	939	395.64	91
191.965	3.5	31	1349	199.70	34
196.973	12.3	1438	1052	715.12	159
200.967	4.3	69	912	254.96	67
210.968	4.3	131	1058	264.94	63
213.003	5.2	177	962	327.58	87
214.965	7.6	673	1095	480.64	113
218.978	4.0	256	1178	255.81	57
226.943	19.0	6603	1194	1267.92	290
231.985	7.7	3446	1075	517.95	135
246.963	4.3	47	1236	317.11	77
256.977	5.9	391	933	445.94	151
273.970	5.0	99	1146	418.39	124
278.901	5.6	319	1204	474.85	137
283.990	7.4	1635	1028	645.45	222
285.916	4.1	54	1081	363.36	120
287.938	5.5	317	1044	492.20	169
297.936	5.1	87	1245	482.50	145
315.925	15.6	15405	1271	1584.39	499
327.968	4.2	55	1195	449.94	158
344.925	4.2	35	1326	477.57	160
349.892	18.3	16791	1293	2121.79	742
373.912	12.3	5576	1278	1539.24	590
398.949	3.9	184	1049	530.41	268

m/z	S/N	Quality Fac.	Res.	Intens.	Area
400.918	3.6	83	1114	488.35	234
403.932	6.0	287	1319	817.26	333
415.998	3.1	97	958	436.01	254
420.974	11.0	15256	1258	1544.85	695
428.909	3.4	83	1402	485.81	201
448.937	11.6	14331	1255	1691.94	826
562.021	5.7	467	1227	846.85	563
630.064	9.5	4485	1351	1368.90	970
647.094	8.3	2333	1402	1189.74	843
760.242	30.3	76102	1462	4140.41	3525
798.464	13.6	5889	1278	1845.77	1931
828.324	4.0	582	1439	533.65	523
879.678	3.6	33	1478	505.27	527
1010.485	5.3	189	1264	601.90	908
1029.275	4.4	197	1766	465.09	517
1031.448	5.1	98	1482	537.17	714
1041.542	16.1	5257	1476	1625.95	2204
1044.635	5.3	173	1208	528.01	879
1050.224	6.1	104	1239	583.09	955
1052.621	5.9	109	1170	554.77	965
1055.007	5.7	95	1172	545.89	952
1057.468	6.0	188	1175	575.27	1003
1068.607	5.4	88	1890	485.64	539
1075.644	75.9	4039	2819	6682.54	5023
1077.875	6.1	99	2124	534.53	534

C:\Users\madhurekha\Desktop\MALDI_Data\16th batch data 070718\BHU\2\0_B2\1\1545.9178.LIFT\1SRef

Comment 1

Comment 2

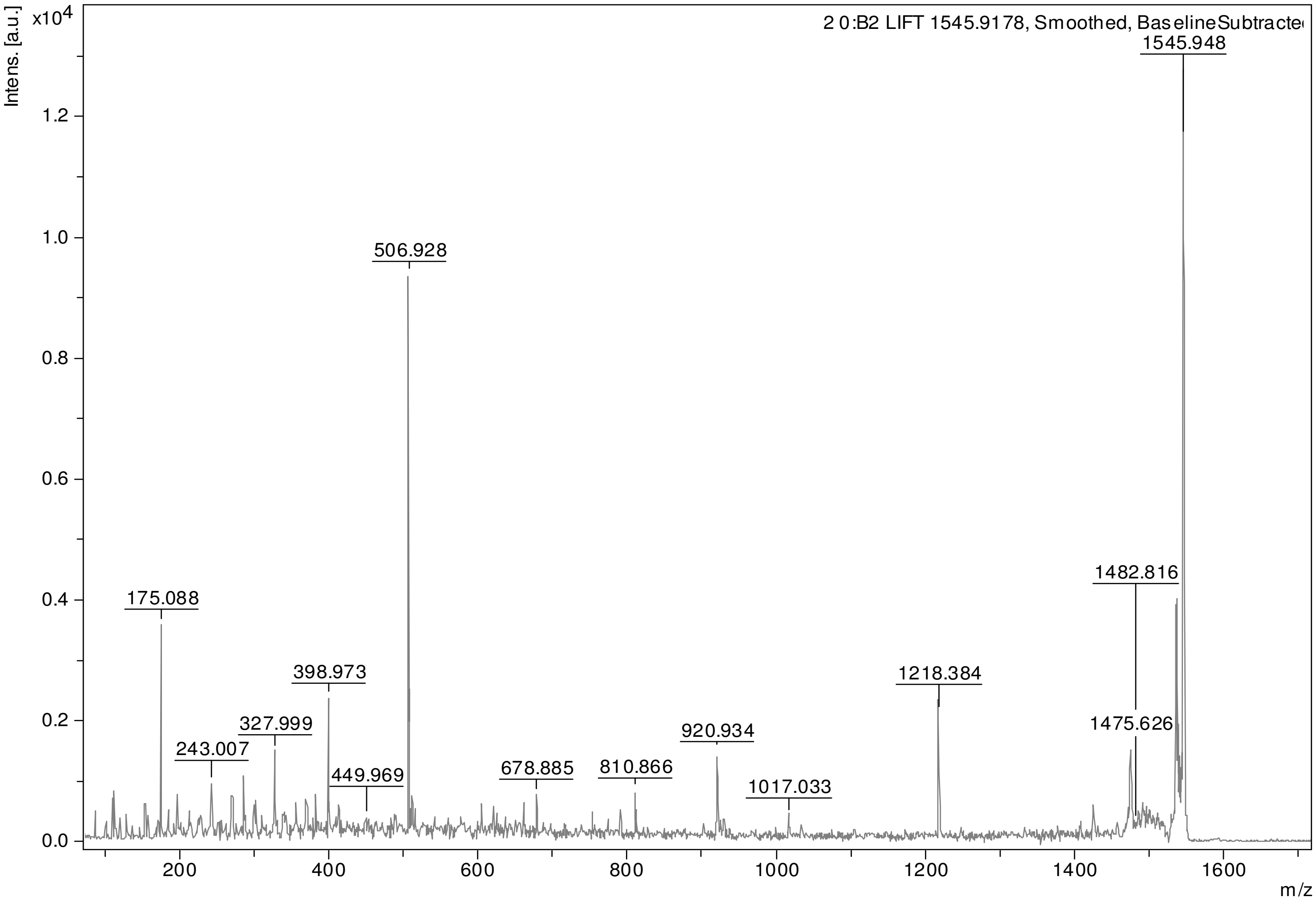

m/z	S/N	Quality Fac.	Res.	Intens.	Area
70.067	5.9	167	613	319.40	40
86.149	8.5	621	501	465.31	89
101.112	5.8	273	849	324.21	43
110.093	13.0	12421	972	725.72	92
112.104	14.3	1623	858	802.01	118
120.097	6.9	284	1029	382.17	50
129.106	8.1	639	1027	455.92	65
136.086	4.5	358	1084	250.78	36
147.081	4.2	292	1079	230.69	36
154.074	11.2	679	1040	626.85	106
158.083	7.6	1365	1091	422.57	70
169.065	4.2	95	1381	236.18	34
171.084	6.5	63	1345	362.39	54
175.088	64.3	28130	1324	3601.47	557
185.081	9.6	884	1196	531.95	97
192.054	5.3	124	1218	294.86	55
197.071	14.6	991	1344	817.70	142
201.057	5.4	48	1204	305.23	60
205.059	4.1	38	1230	235.58	47
213.069	9.2	353	1355	523.77	98
215.051	5.4	53	1239	308.59	64
226.038	7.4	62	1425	427.16	82
228.046	7.9	34	1469	457.40	86
230.020	5.6	169	1374	325.56	66
235.035	3.6	32	1437	207.40	41
243.007	16.9	757	1504	994.61	195
251.051	5.2	49	1312	311.77	73
256.034	6.2	74	1511	377.19	78
268.023	7.2	53	1410	448.63	105
270.023	12.3	1623	1455	768.81	176
272.022	11.5	617	1474	719.30	164
276.012	5.0	48	1559	320.88	70
285.993	16.8	651	1514	1128.50	266
299.996	9.1	100	1556	643.96	156
302.995	9.9	469	1559	700.60	171
310.978	6.4	40	1627	469.24	113
314.021	3.5	46	1332	259.99	78
315.992	3.8	57	1412	287.48	82

C:\Users\madhurekha\Desktop\MALDI_Data\16th batch data 070718\BHU\2\0_B2\1\1545.9178.LIFT\1SRef

m/z	S/N	Quality Fac.	Res.	Intens.	Area
327.999	19.0	3652	1617	1519.11	393
330.971	4.5	57	1725	359.10	88
338.985	5.8	67	1463	485.48	144
341.016	6.2	95	1625	520.47	140
342.993	5.1	42	1641	439.11	118
355.964	7.6	101	1633	681.51	193
368.999	7.8	254	1636	739.76	218
370.994	6.6	122	1536	625.51	198
379.011	4.3	51	1438	422.99	146
382.004	8.2	393	1621	805.12	250
398.973	23.2	8300	1732	2413.01	738
449.969	3.6	51	1764	430.69	150
487.945	3.5	58	1737	474.68	186
489.912	3.1	67	1883	425.50	155
506.928	66.6	443857	2062	9417.07	3263
511.983	5.9	94	1872	836.98	323
515.957	4.1	70	2012	590.26	214
604.888	4.0	134	2084	638.77	278
621.861	3.8	84	2038	606.28	280
625.936	3.1	47	2003	508.79	241
661.869	4.0	107	2013	667.46	340
678.885	4.8	560	1995	798.68	425
791.929	3.5	234	1836	547.68	394
810.866	5.6	310	1802	877.76	666
920.934	10.9	681	1966	1539.28	1294
1017.033	3.4	157	1775	453.53	492
1218.384	15.8	5533	1976	2148.04	2832
1425.686	4.5	664	1894	591.76	1074
1475.626	15.7	97	2855	1721.10	2208
1482.816	3.3	55	1961	357.08	674
1486.532	4.0	47	1651	421.05	947
1491.087	6.6	130	1811	698.68	1441
1494.193	4.1	245	2299	434.63	708
1497.274	5.1	126	2415	525.35	818
1500.585	5.9	90	2417	611.75	954
1505.295	3.8	141	1671	376.75	857
1510.995	4.3	129	1680	426.87	973
1519.840	3.0	110	2067	297.46	557
1528.985	3.9	50	1809	363.71	787
1536.794	45.8	26574	2951	4215.63	5646
1539.087	12.9	454	3906	1179.65	1197

C:\Users\madhurekha\Desktop\MALDI_Data\16th batch data 070718\BHU\2\0_B2\1\1545.9178.LIFT\1SRef

m/z	S/N	Quality Fac.	Res.	Intens.	Area
1543.640	12.0	110	3186	1091.06	1365
1545.948	129.7	55975	3356	11671.00	13889

C:\Users\madhurekha\Desktop\MALDI_Data\16th batch data 070718\BHU\2\0_B2\1\1764.0908.LIFT\1SRef

Comment 1

Comment 2

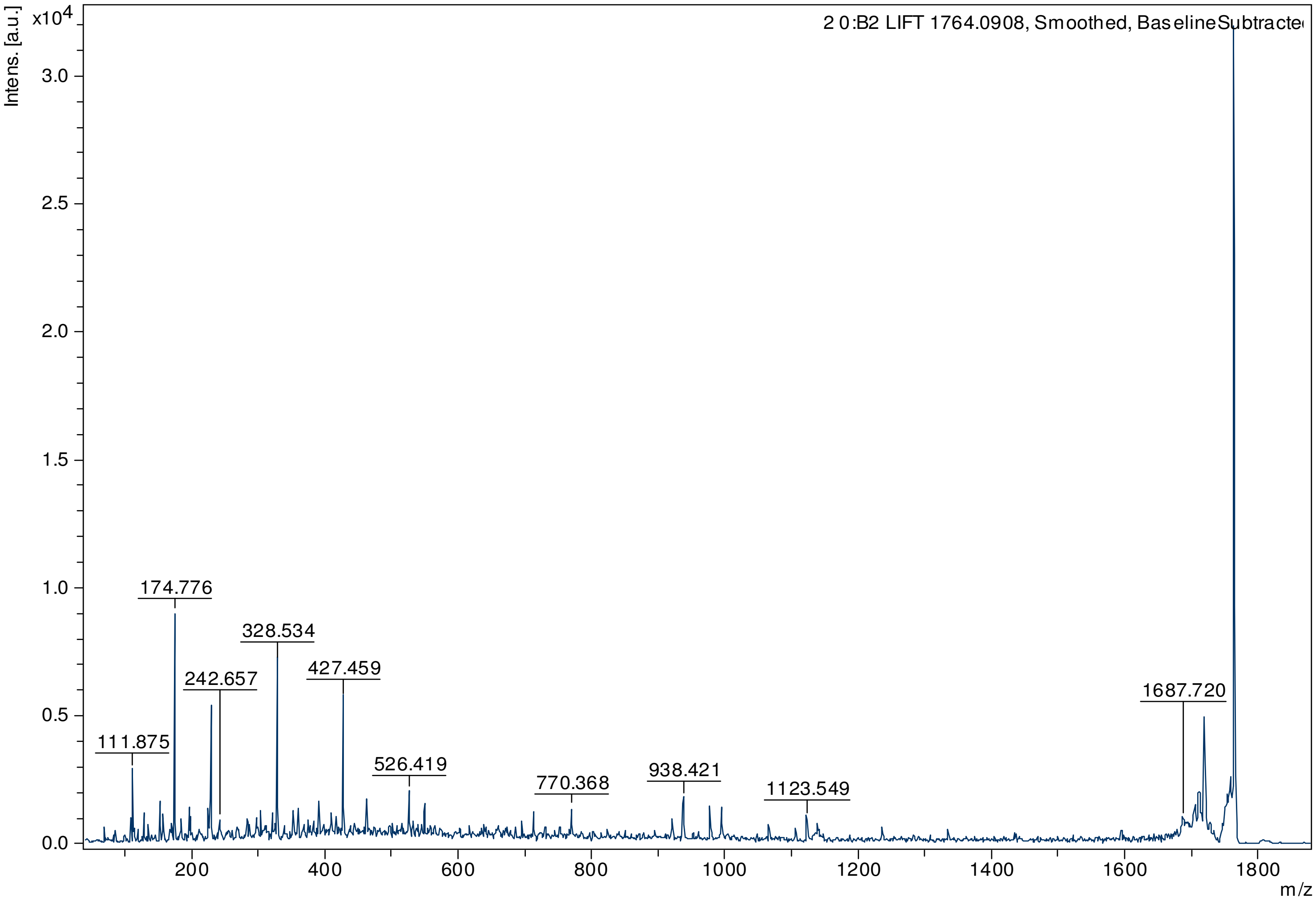

m/z	S/N	Quality Fac.	Res.	Intens.	Area
69.915	7.0	1092	413	683.16	127
83.901	3.6	1395	684	350.30	48
85.930	5.4	590	545	528.28	92
100.885	3.4	152	679	329.83	55
111.875	29.7	10731	1003	2919.78	365
114.865	6.3	1415	1099	622.00	73
119.860	6.0	1647	933	589.23	85
128.867	12.6	3926	1125	1246.13	161
132.849	3.1	628	1216	307.18	38
135.824	7.8	2696	1114	769.87	106
140.813	3.1	72	887	310.47	56
146.822	3.3	260	1162	330.77	48
153.801	16.5	3036	1230	1675.38	240
157.788	11.7	2830	1324	1200.51	164
168.780	5.5	841	1144	577.94	99
170.797	8.0	189	1250	839.72	134
174.776	85.6	23237	1241	9022.79	1486
180.758	3.1	44	1297	333.12	54
185.745	9.0	1749	1159	980.33	185
191.754	3.7	69	1120	405.09	82
196.762	13.4	2807	1195	1508.71	294
198.754	9.5	991	1237	1077.69	205
201.720	3.7	145	1181	427.47	87
211.710	5.5	59	1230	671.73	138
216.737	3.1	54	1251	384.25	80
225.679	11.3	521	1195	1478.78	336
229.653	40.7	14995	1305	5414.73	1150
242.657	6.8	118	1141	960.04	248
261.627	3.5	70	1169	555.96	153
271.651	3.3	38	1164	567.74	164
282.583	4.5	88	1163	823.62	249
284.592	5.5	132	1321	1029.73	276
287.644	4.2	117	1077	807.28	269
297.599	5.3	189	1348	1058.09	293
304.561	6.1	705	1281	1267.75	379
321.535	5.6	466	1455	1271.77	357
328.534	30.3	10341	1375	7100.37	2166
353.503	5.0	94	1170	1306.25	511

C:\Users\madhurekha\Desktop\MALDI_Data\16th batch data 070718\BHU\2\0_B2\1\1764.0908.LIFT\1SRef

m/z	S/N	Quality Fac.	Res.	Intens.	Area
360.483	5.2	548	1401	1379.37	461
375.492	3.5	59	1377	991.13	354
392.466	5.7	481	1439	1706.70	616
410.458	4.3	37	1530	1346.58	483
417.438	3.4	61	1345	1074.88	448
427.459	17.5	11923	1500	5695.62	2190
463.428	5.2	470	1474	1818.29	787
526.419	5.7	612	1569	2099.23	1003
550.406	4.3	486	1632	1595.58	776
713.359	3.8	186	2042	1292.23	720
770.368	4.3	213	1977	1417.87	910
921.362	3.6	344	2078	1027.60	818
938.421	6.4	3509	2315	1812.04	1331
978.384	5.0	685	2056	1382.18	1219
995.451	5.4	2464	2092	1453.67	1294
1066.498	3.4	316	2048	853.84	866
1123.549	4.4	542	1685	1024.24	1382
1139.398	3.7	48	2780	855.65	716
1683.994	3.8	40	2218	684.99	1464
1687.720	5.8	215	2221	1032.58	2211
1690.911	3.7	120	1938	647.38	1595
1694.164	3.4	75	1880	592.83	1512
1698.333	4.4	86	2298	775.06	1624
1702.668	4.4	60	2300	761.09	1601
1704.734	6.6	372	1894	1127.98	2902
1707.440	5.4	120	1897	912.58	2345
1710.956	11.3	1881	1899	1899.44	4891
1713.458	4.4	88	1904	749.33	1935
1715.767	6.0	112	1906	1007.12	2602
1719.875	23.6	14604	1911	3915.19	10117
1726.462	3.7	195	1917	611.75	1590
1728.640	3.4	112	1921	551.73	1433
1746.077	3.3	80	1940	509.64	1339
1748.306	3.5	58	2769	555.87	1025
1750.529	8.6	245	3210	1351.89	2166
1753.901	11.7	1974	4162	1812.12	2238
1757.257	11.9	1510	4209	1835.70	2252
1759.503	9.2	74	3891	1408.83	1876
1761.873	13.3	46	3288	2034.04	3209
1764.091	163.1	28936	3073	24768.05	41892

C:\Users\madhurekha\Desktop\MALDI_Data\16th batch data 070718\BHU\2\0_B2\1\1SRef

Comment 1

Comment 2

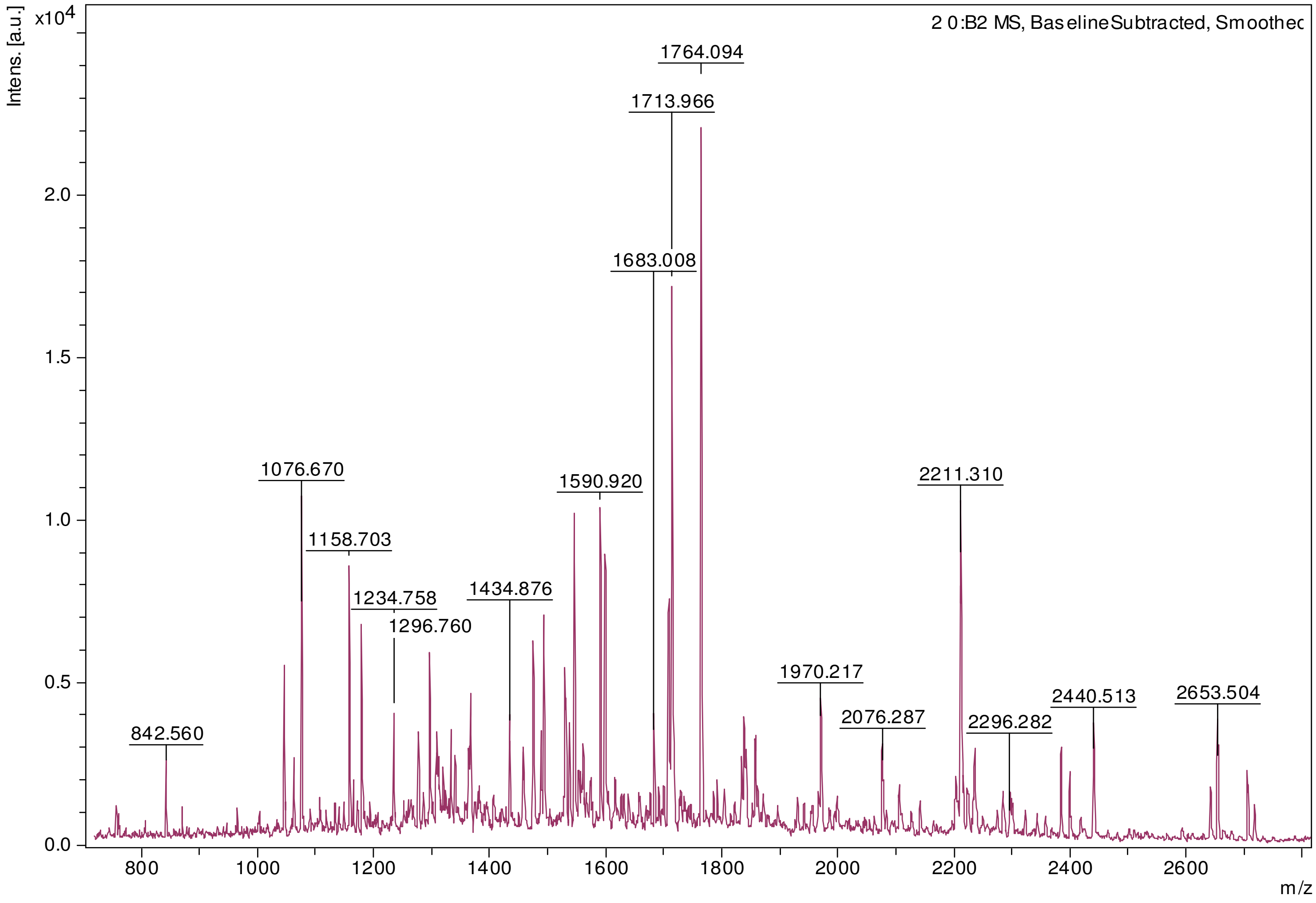

m/z	S/N	Quality Fac.	Res.	Intens.	Area
757.440	6.2	1281	4689	1071.90	289
842.560	13.3	5348	5800	2467.88	651
1046.610	26.2	22394	7181	5702.12	1708
1062.641	12.3	964	6979	2750.84	866
1075.652	28.8	2608	7268	6516.14	1958
1076.670	32.5	16406	7386	7376.42	2257
1158.703	35.0	30465	7510	8756.48	2974
1165.662	6.3	2394	6119	1589.24	642
1179.684	27.7	9489	8076	7108.40	2322
1234.758	15.5	1849	7418	4222.06	1567
1277.754	11.3	8645	7544	3164.86	1259
1296.760	22.4	3235	8322	6373.01	2377
1308.763	11.0	306	6027	3177.87	1597
1333.787	10.5	4325	7345	3059.88	1291
1340.788	8.0	1271	6751	2334.15	1090
1363.827	7.0	143	6318	2062.15	1044
1367.824	14.4	9842	8529	4253.29	1666
1434.876	12.2	9522	8209	3681.02	1599
1457.838	9.5	646	7039	2886.62	1502
1475.871	19.2	27590	8005	5846.14	2782
1488.886	10.0	2930	7452	3042.31	1536
1493.853	23.1	24804	8480	7033.30	3227
1530.804	15.3	4575	6300	4685.82	3001
1537.868	11.2	3498	7515	3419.09	1855
1545.921	34.1	61332	8692	10433.77	5017
1561.939	6.0	718	6277	1834.17	1211
1590.920	34.5	38807	9415	10486.76	5009
1598.956	31.5	29813	9310	9530.01	4628
1683.008	11.4	2049	7457	3392.36	2198
1707.930	22.2	3084	7667	6524.07	4277
1713.966	59.3	47902	9445	17390.34	9518
1764.094	81.6	42836	9967	23614.95	13159
1834.114	7.7	975	6792	2154.01	1819
1839.070	11.3	2043	7612	3164.21	2409
1858.146	8.8	851	6078	2413.12	2335
1970.217	16.0	1323	7218	3844.72	3373
2076.287	12.2	3183	8298	2486.28	2036
2203.333	8.4	470	6498	1397.54	1550

C:\Users\madhurekha\Desktop\MALDI_Data\16th batch data 070718\BHU\2\0_B2\1\1SRef

m/z	S/N	Quality Fac.	Res.	Intens.	Area
2211.310	54.0	30739	11032	8877.64	6117
2222.281	6.7	434	5200	1089.65	1533
2235.350	9.8	527	5350	1561.60	2136
2284.363	6.7	1047	6973	989.46	1075
2296.282	6.2	365	6296	904.80	1103
2384.175	15.9	2328	8113	2013.83	2011
2399.245	11.8	2928	8185	1453.17	1460
2440.513	24.6	19331	10473	2835.74	2307
2641.562	13.3	3177	8219	1100.17	1264
2653.504	32.5	35189	11398	2620.37	2224
2705.380	19.5	6338	9579	1424.56	1473
2717.322	9.7	3543	8203	700.19	854

MATRIX SCIENCE Mascot Search Results

```
User          : sanovar
Email         : sanovar@sandor.co.in
Search title  :
MS data file  : DATA.TXT
Database      : SwissProt 2018_06 (557713 sequences; 200130199 residues)
Taxonomy      : Bacteria (Eubacteria) (333552 sequences)
Timestamp     : 11 Jul 2018 at 11:37:18 GMT
Warning       : A Peptide summary report will usually give a much clearer picture of MS/MS search results.
Top Score     : 191 for DLDH_ECO57, Dihydrolipoyl dehydrogenase OS=Escherichia coli O157:H7 OX=83334 GN=lpdA PE=3 SV=2
```

Mascot Score Histogram

Protein score is -10*Log(P), where P is the probability that the observed match is a random event.
Protein scores greater than 68 are significant (p<0.05).
Protein scores are derived from ions scores as a non-probabilistic basis for ranking protein hits.

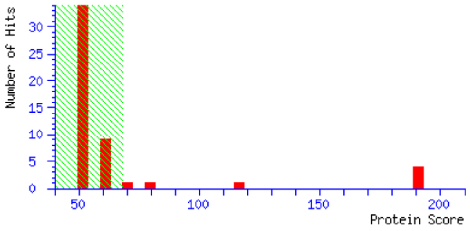

Protein Summary Report

Format As | Protein Summary (deprecated) | Help

Significance threshold p< 0.05 Max. number of hits 20

Preferred taxonomy All entries

Re-Search All | Search Unmatched

Index

	Accession	Mass	Score	Description
1.	DLDH_ECO57	50942	191	Dihydrolipoyl dehydrogenase OS=Escherichia coli O157:H7 OX=83334 GN=lpdA PE=3 SV=2
2.	DLDH_ECOL6	50942	191	Dihydrolipoyl dehydrogenase OS=Escherichia coli O6:H1 (strain CFT073 / ATCC 700928 / UPEC) OX=199310 GI
3.	DLDH_ECOLI	50942	191	Dihydrolipoyl dehydrogenase OS=Escherichia coli (strain K12) OX=83333 GN=lpdA PE=1 SV=2
4.	DLDH_SHIFL	50942	191	Dihydrolipoyl dehydrogenase OS=Shigella flexneri OX=623 GN=lpdA PE=3 SV=2
5.	DLDH_HAEIN	51521	119	Dihydrolipoyl dehydrogenase OS=Haemophilus influenzae (strain ATCC 51907 / DSM 11121 / KW20 / Rd) OX=7
6.	AROA_BACLD	45722	78	3-phosphoshikimate 1-carboxyvinyltransferase OS=Bacillus licheniformis (strain ATCC 14580 / DSM 13 / J
7.	GLMM_COXBN	48301	69	Phosphoglucosamine mutase OS=Coxiella burnetii (strain Dugway 5J108-111) OX=434922 GN=glmM PE=3 SV=1
8.	GLMM_COXB1	48347	63	Phosphoglucosamine mutase OS=Coxiella burnetii (strain CbuK_Q154) OX=434924 GN=glmM PE=3 SV=1
9.	GLMM_COXB2	48347	63	Phosphoglucosamine mutase OS=Coxiella burnetii (strain CbuG_Q212) OX=434923 GN=glmM PE=3 SV=1
10.	TRAA_RHIRD	123705	61	Conjugal transfer protein TraA OS=Rhizobium radiobacter OX=358 GN=traA PE=3 SV=1
11.	RPOC2_SYNSC	148515	61	DNA-directed RNA polymerase subunit beta' OS=Synechococcus sp. (strain CC9605) OX=110662 GN=rpoC2 PE=3
12.	PAT_SALAR	50192	61	Putrescine aminotransferase OS=Salmonella arizonae (strain ATCC BAA-731 / CDC346-86 / RSK2980) OX=4151
13.	PAT_SALPB	50078	61	Putrescine aminotransferase OS=Salmonella paratyphi B (strain ATCC BAA-1250 / SPB7) OX=1016998 GN=patA
14.	RPOC2_PROMS	150243	59	DNA-directed RNA polymerase subunit beta' OS=Prochlorococcus marinus (strain AS9601) OX=146891 GN=rpoC
15.	RPOC2_PROM0	150258	57	DNA-directed RNA polymerase subunit beta' OS=Prochlorococcus marinus (strain MIT 9301) OX=167546 GN=rp
16.	RL9_PARXL	16075	57	50S ribosomal protein L9 OS=Paraburkholderia xenovorans (strain LB400) OX=266265 GN=rplI PE=3 SV=1
17.	RL10_THIDA	18640	56	50S ribosomal protein L10 OS=Thiobacillus denitrificans (strain ATCC 25259) OX=292415 GN=rplJ PE=3 SV=
18.	EPMA_SALAR	37284	55	Elongation factor P--(R)-beta-lysine ligase OS=Salmonella arizonae (strain ATCC BAA-731 / CDC346-86 /
19.	IF2_BACFN	112675	55	Translation initiation factor IF-2 OS=Bacteroides fragilis (strain ATCC 25285 / DSM 2151 / JCM 11019 /
20.	IF2_BACFR	112675	55	Translation initiation factor IF-2 OS=Bacteroides fragilis (strain YCH46) OX=295405 GN=infB PE=3 SV=1

Results List

1. DLDH_ECO57 **Mass:** 50942 **Score:** 191 **Expect:** 2.6e-14 **Matches:** 8
Dihydrolipoyl dehydrogenase OS=Escherichia coli O157:H7 OX=83334 GN=lpdA PE=3 SV=2

Observed	Mr(expt)	Mr(calc)	ppm	Start - End	Miss	Ions	Peptide
758.4449	757.4377	757.4446	-9.19	297 - 302	1	---	R.VDKQLR.T
1076.6699	1075.6627	1075.6026	55.8	264 - 273	0	---	R.YDAVLVAIGR.V
1333.7866	1332.7793	1332.6786	75.5	285 - 296	1	---	K.AGVEVDDRGFIR.V
1367.8244	1366.8172	1366.7245	67.8	68 - 80	0	---	K.ALAEHGIVFGEPK.T
1713.9658	1712.9585	1712.8158	83.3	371 - 386	0	59	K.GISYETATFPWAASGR.A
1764.0939	1763.0866	1762.9366	85.1	7 - 24	0	103	K.TQVVVLGAGPAGYSAAFR.C
2274.3577	2273.3504	2273.1991	66.6	303 - 323	0	---	R.TNVPHIFAIGDIVGQPMLAHK.G + Oxidation (M)
2322.3983	2321.3910	2321.2379	66.0	2 - 24	1	---	M.STEIKTQVVVLGAGPAGYSAAFR.C

No match to: 761.4539, 773.4587, 807.4419, 842.5599, 870.5871, 931.5710, 965.5635, 1003.6035, 1046.6100, 1062.6412, 1064.6582, 1075.6517, 1118.6055, 1133.6818, 1140.6556, 1149.6929, 1158.7030, 1165.6616, 1172.7483, 1179.6837, 1234.7577, 1259.7439, 1277.7544, 1285.7582, 1296.7598, 1308.7634, 1320.6892, 1340.7881, 1373.7740, 1390.7958, 1434.8763, 1457.8382, 1475.8714, 1488.8863, 1493.8532, 1530.8044, 1537.8684, 1545.9206, 1553.9584, 1561.9388, 1590.9204, 1598.9555, 1615.9930, 1638.9781, 1657.9450, 1683.0081, 1690.9261, 1699.9587, 1707.9303, 1729.9719, 1786.0298, 1791.8986, 1804.0504, 1822.0336, 1834.1144, 1839.0705, 1842.1348, 1858.1460, 1862.0788, 1930.0964, 1941.1169, 1955.1238, 1970.2165, 1972.1537, 1985.1441, 1998.1688, 2076.2873, 2083.1868, 2105.2985, 2126.2672, 2141.2573, 2164.2353, 2203.3329, 2211.3104, 2215.3655, 2222.2808, 2235.3499, 2284.3628, 2296.2822, 2343.2148, 2357.2678, 2384.1747, 2399.2446, 2418.3632, 2440.5128, 2592.4853, 2641.5621, 2653.5041, 2705.3800, 2717.3219, 2888.5748, 3264.6235

2. DLDH_ECOL6 **Mass:** 50942 **Score:** 191 **Expect:** 2.6e-14 **Matches:** 8
Dihydrolipoyl dehydrogenase OS=Escherichia coli O6:H1 (strain CFT073 / ATCC 700928 / UPEC) OX=199310 GN=lpdA PE=3 SV=2

Observed	Mr(expt)	Mr(calc)	ppm	Start - End	Miss	Ions	Peptide
758.4449	757.4377	757.4446	-9.19	297 - 302	1	---	R.VDKQLR.T
1076.6699	1075.6627	1075.6026	55.8	264 - 273	0	---	R.YDAVLVAIGR.V

1333.7866	1332.7793	1332.6786	75.5	285	296	1	---	K.AGVEVDDRGFIR.V
1367.8244	1366.8172	1366.7245	67.8	68	80	0	---	K.ALAEHGIVFGEPK.T
1713.9658	1712.9585	1712.8158	83.3	371	386	0	59	K.GISYETATFPWAASGR.A
1764.0939	1763.0866	1762.9366	85.1	7	24	0	103	K.TQVVVLGAGPAGYSAAFR.C
2274.3577	2273.3504	2273.1991	66.6	303	323	0	---	R.TNVPHIFAIGDIVGQPMLAHK.G + Oxidation (M)
2322.3983	2321.3910	2321.2379	66.0	2	24	1	---	M.STEIKTQVVVLGAGPAGYSAAFR.C

No match to: 761.4539, 773.4587, 807.4419, 842.5599, 870.5871, 931.5710, 965.5635, 1003.6035, 1046.6100, 1062.6412, 1064.6582, 1075.6517, 1118.6055, 1133.6818, 1140.6556, 1149.6929, 1158.7030, 1165.6616, 1172.7483, 1179.6837, 1234.7577, 1259.7439, 1277.7544, 1285.7582, 1296.7598, 1308.7634, 1320.6892, 1340.7881, 1373.7740, 1390.7958, 1434.8763, 1457.8382, 1475.8714, 1488.8863, 1493.8532, 1530.8044, 1537.8684, 1545.9206, 1553.9584, 1561.9388, 1590.9204, 1598.9555, 1615.9930, 1638.9781, 1657.9450, 1683.0081, 1690.9261, 1699.9587, 1707.9303, 1729.9719, 1786.0298, 1791.8986, 1804.0504, 1822.0336, 1834.1144, 1839.0705, 1842.1348, 1858.1460, 1862.0788, 1930.0964, 1941.1169, 1955.1238, 1970.2165, 1972.1537, 1985.1441, 1998.1688, 2076.2873, 2083.1868, 2105.2985, 2126.2672, 2141.2573, 2164.2353, 2203.3329, 2211.3104, 2215.3655, 2222.2808, 2235.3499, 2284.3628, 2296.2822, 2343.2148, 2357.2678, 2384.1747, 2399.2446, 2418.3632, 2440.5128, 2592.4853, 2641.5621, 2653.5041, 2705.3800, 2717.3219, 2888.5748, 3264.6235

3. DLDH_ECOLI **Mass:** 50942 **Score:** 191 **Expect:** 2.6e-14 **Matches:** 8

Dihydrolipoyl dehydrogenase OS=Escherichia coli (strain K12) OX=83333 GN=lpdA PE=1 SV=2

Observed	Mr(expt)	Mr(calc)	ppm	Start	End	Miss	Ions	Peptide
758.4449	757.4377	757.4446	-9.19	297	302	1	---	R.VDKQLR.T
1076.6699	1075.6627	1075.6026	55.8	264	273	0	---	R.YDAVLVAIGR.V
1333.7866	1332.7793	1332.6786	75.5	285	296	1	---	K.AGVEVDDRGFIR.V
1367.8244	1366.8172	1366.7245	67.8	68	80	0	---	K.ALAEHGIVFGEPK.T
1713.9658	1712.9585	1712.8158	83.3	371	386	0	59	K.GISYETATFPWAASGR.A
1764.0939	1763.0866	1762.9366	85.1	7	24	0	103	K.TQVVVLGAGPAGYSAAFR.C
2274.3577	2273.3504	2273.1991	66.6	303	323	0	---	R.TNVPHIFAIGDIVGQPMLAHK.G + Oxidation (M)
2322.3983	2321.3910	2321.2379	66.0	2	24	1	---	M.STEIKTQVVVLGAGPAGYSAAFR.C

No match to: 761.4539, 773.4587, 807.4419, 842.5599, 870.5871, 931.5710, 965.5635, 1003.6035, 1046.6100, 1062.6412, 1064.6582, 1075.6517, 1118.6055, 1133.6818, 1140.6556, 1149.6929, 1158.7030, 1165.6616, 1172.7483, 1179.6837, 1234.7577, 1259.7439, 1277.7544, 1285.7582, 1296.7598, 1308.7634, 1320.6892, 1340.7881, 1373.7740, 1390.7958, 1434.8763, 1457.8382, 1475.8714, 1488.8863, 1493.8532, 1530.8044, 1537.8684, 1545.9206, 1553.9584, 1561.9388, 1590.9204, 1598.9555, 1615.9930, 1638.9781, 1657.9450, 1683.0081, 1690.9261, 1699.9587, 1707.9303, 1729.9719, 1786.0298, 1791.8986, 1804.0504, 1822.0336, 1834.1144, 1839.0705, 1842.1348, 1858.1460, 1862.0788, 1930.0964, 1941.1169, 1955.1238, 1970.2165, 1972.1537, 1985.1441, 1998.1688, 2076.2873, 2083.1868, 2105.2985, 2126.2672, 2141.2573, 2164.2353, 2203.3329, 2211.3104, 2215.3655, 2222.2808, 2235.3499, 2284.3628, 2296.2822, 2343.2148, 2357.2678, 2384.1747, 2399.2446, 2418.3632, 2440.5128, 2592.4853, 2641.5621, 2653.5041, 2705.3800, 2717.3219, 2888.5748, 3264.6235

4. DLDH_SHIFL **Mass:** 50942 **Score:** 191 **Expect:** 2.6e-14 **Matches:** 8

Dihydrolipoyl dehydrogenase OS=Shigella flexneri OX=623 GN=lpdA PE=3 SV=2

Observed	Mr(expt)	Mr(calc)	ppm	Start	End	Miss	Ions	Peptide
758.4449	757.4377	757.4446	-9.19	297	302	1	---	R.VDKQLR.T
1076.6699	1075.6627	1075.6026	55.8	264	273	0	---	R.YDAVLVAIGR.V
1333.7866	1332.7793	1332.6786	75.5	285	296	1	---	K.AGVEVDDRGFIR.V
1367.8244	1366.8172	1366.7245	67.8	68	80	0	---	K.ALAEHGIVFGEPK.T
1713.9658	1712.9585	1712.8158	83.3	371	386	0	59	K.GISYETATFPWAASGR.A
1764.0939	1763.0866	1762.9366	85.1	7	24	0	103	K.TQVVVLGAGPAGYSAAFR.C
2274.3577	2273.3504	2273.1991	66.6	303	323	0	---	R.TNVPHIFAIGDIVGQPMLAHK.G + Oxidation (M)
2322.3983	2321.3910	2321.2379	66.0	2	24	1	---	M.STEIKTQVVVLGAGPAGYSAAFR.C

No match to: 761.4539, 773.4587, 807.4419, 842.5599, 870.5871, 931.5710, 965.5635, 1003.6035, 1046.6100, 1062.6412, 1064.6582, 1075.6517, 1118.6055, 1133.6818, 1140.6556, 1149.6929, 1158.7030, 1165.6616, 1172.7483, 1179.6837, 1234.7577, 1259.7439, 1277.7544, 1285.7582, 1296.7598, 1308.7634, 1320.6892, 1340.7881, 1373.7740, 1390.7958, 1434.8763, 1457.8382, 1475.8714, 1488.8863, 1493.8532, 1530.8044, 1537.8684, 1545.9206, 1553.9584, 1561.9388, 1590.9204, 1598.9555, 1615.9930, 1638.9781, 1657.9450, 1683.0081, 1690.9261, 1699.9587, 1707.9303, 1729.9719, 1786.0298, 1791.8986, 1804.0504, 1822.0336, 1834.1144, 1839.0705, 1842.1348, 1858.1460, 1862.0788, 1930.0964, 1941.1169, 1955.1238, 1970.2165, 1972.1537, 1985.1441, 1998.1688, 2076.2873, 2083.1868, 2105.2985, 2126.2672, 2141.2573, 2164.2353, 2203.3329, 2211.3104, 2215.3655, 2222.2808, 2235.3499, 2284.3628, 2296.2822, 2343.2148, 2357.2678, 2384.1747, 2399.2446, 2418.3632, 2440.5128, 2592.4853, 2641.5621, 2653.5041, 2705.3800, 2717.3219, 2888.5748, 3264.6235

5. DLDH_HAEIN **Mass:** 51521 **Score:** 119 **Expect:** 4.2e-07 **Matches:** 5

Dihydrolipoyl dehydrogenase OS=Haemophilus influenzae (strain ATCC 51907 / DSM 11121 / KW20 / Rd) OX=71421 GN=lpdA PE=3 SV=2

Observed	Mr(expt)	Mr(calc)	ppm	Start	End	Miss	Ions	Peptide
1076.6699	1075.6627	1075.6026	55.8	264	273	0	---	R.YDAVLVAIGR.V
1457.8382	1456.8309	1456.7522	54.1	279	292	1	---	K.LIDAGKAGVEVDDR.G
1764.0939	1763.0866	1762.9366	85.1	7	24	0	103	K.TQVVVLGAGPAGYSAAFR.C
2274.3577	2273.3504	2273.1991	66.6	303	323	0	---	R.TNVPHIYAIGDIVGQPMLAHK.G
2705.3800	2704.3728	2704.3941	-7.90	300	323	1	---	K.QMRTNVPHIYAIGDIVGQPMLAHK.G + Oxidation (M)

No match to: 758.4449, 761.4539, 773.4587, 807.4419, 842.5599, 870.5871, 931.5710, 965.5635, 1003.6035, 1046.6100, 1062.6412, 1064.6582, 1075.6517, 1118.6055, 1133.6818, 1140.6556, 1149.6929, 1158.7030, 1165.6616, 1172.7483, 1179.6837, 1234.7577, 1259.7439, 1277.7544, 1285.7582, 1296.7598, 1308.7634, 1320.6892, 1333.7866, 1340.7881, 1367.8244, 1373.7740, 1390.7958, 1434.8763, 1475.8714, 1488.8863, 1493.8532, 1530.8044, 1537.8684, 1545.9206, 1553.9584, 1561.9388, 1590.9204, 1598.9555, 1615.9930, 1638.9781, 1657.9450, 1683.0081, 1690.9261, 1699.9587, 1707.9303, 1713.9658, 1729.9719, 1786.0298, 1791.8986, 1804.0504, 1822.0336, 1834.1144, 1839.0705, 1842.1348, 1858.1460, 1862.0788, 1930.0964, 1941.1169, 1955.1238, 1970.2165, 1972.1537, 1985.1441, 1998.1688, 2076.2873, 2083.1868, 2105.2985, 2126.2672, 2141.2573, 2164.2353, 2203.3329, 2211.3104, 2215.3655, 2222.2808, 2235.3499, 2284.3628, 2296.2822, 2322.3983, 2343.2148, 2357.2678, 2384.1747, 2399.2446, 2418.3632, 2440.5128, 2592.4853, 2641.5621, 2653.5041, 2717.3219, 2888.5748, 3264.6235

6. AROA_BACLD **Mass:** 45722 **Score:** 78 **Expect:** 0.0058 **Matches:** 14

3-phosphoshikimate 1-carboxyvinyltransferase OS=Bacillus licheniformis (strain ATCC 14580 / DSM 13 / JCM 2505 / NBRC 12200 / NCI

Observed	Mr(expt)	Mr(calc)	ppm	Start	End	Miss	Ions	Peptide
842.5599	841.5527	841.5021	60.0	124	130	1	---	R.VTEPLRK.M
870.5871	869.5798	869.5083	82.2	123	129	1	---	K.RVTEPLR.K
965.5635	964.5563	964.4978	60.6	11	19	0	---	K.GELHIPGDK.S
1140.6556	1139.6483	1139.5831	57.2	197	206	0	---	R.MLSMFGVSLR.E
1149.6929	1148.6856	1148.5826	89.7	139	149	0	---	R.AGGEYTPLSVR.G
1179.6837	1178.6765	1178.6772	-0.58	367	378	1	---	K.IHGKTPLTGGAK.V
1475.8714	1474.8641	1474.7627	68.8	340	352	1	---	K.ETNRIDTVASELK.K
1493.8532	1492.8459	1492.8249	14.1	6	19	1	---	K.ISSLKGELHIPGDK.S
1545.9206	1544.9134	1544.8059	69.5	11	24	1	7	K.GELHIPGDKSISHR.S
1590.9204	1589.9131	1589.8162	61.0	135	149	1	---	K.IDGRAGGEYTPLSVR.G
1657.9450	1656.9377	1656.9046	20.0	294	309	1	---	K.TSSLKAAEISGDLIPR.L
1707.9303	1706.9230	1706.8232	58.5	20	35	1	---	K.SISHRSVMFGAMAEGK.T
2322.3983	2321.3910	2321.3457	19.5	310	331	0	---	R.LIDEIPIIALLATQAEGTTVIK.D
2399.2446	2398.2373	2398.2679	-12.74	96	118	0	---	R.LMLGILAGRPFHSTVAGDESIAK.R + Oxidation (M)

No match to: 758.4449, 761.4539, 773.4587, 807.4419, 931.5710, 1003.6035, 1046.6100, 1062.6412, 1064.6582, 1075.6517, 1076.6699, 1118.6055, 1133.6818, 1158.7030, 1165.6616, 1172.7483, 1234.7577, 1259.7439, 1277.7544, 1285.7582, 1296.7598,

1308.7634, 1320.6892, 1333.7866, 1340.7881, 1367.8244, 1373.7740, 1390.7958, 1434.8763, 1457.8382, 1488.8863, 1530.8044, 1537.8684, 1553.9584, 1561.9388, 1598.9555, 1615.9930, 1638.9781, 1683.0081, 1690.9261, 1699.9587, 1713.9658, 1729.9719, 1764.0939, 1786.0298, 1791.8986, 1804.0504, 1822.0336, 1834.1144, 1839.0705, 1842.1348, 1858.1460, 1862.0788, 1930.0964, 1941.1169, 1955.1238, 1970.2165, 1972.1537, 1985.1441, 1998.1688, 2076.2873, 2083.1868, 2105.2985, 2126.2672, 2141.2573, 2164.2353, 2203.3329, 2211.3104, 2215.3655, 2222.2808, 2235.3499, 2274.3577, 2284.3628, 2296.2822, 2343.2148, 2357.2678, 2384.1747, 2418.3632, 2440.5128, 2592.4853, 2641.5621, 2653.5041, 2705.3800, 2717.3219, 2888.5748, 3264.6235

7. GLMM_COXBN **Mass:** 48301 **Score:** 69 **Expect:** 0.046 **Matches:** 9

Phosphoglucosamine mutase OS=Coxiella burnetii (strain Dugway 5J108-111) OX=434922 GN=glmM PE=3 SV=1

Observed	Mr(expt)	Mr(calc)	ppm	Start	End	Miss	Ions	Peptide
758.4449	757.4377	757.4235	18.7	28 -	34	0	---	K.LGWAVGR.V
761.4539	760.4466	760.3967	65.7	393 -	399	0	---	K.AITEAEK.Q
1234.7577	1233.7504	1233.7445	4.81	369 -	379	0	---	K.NPQVLINVPIK.G
1259.7439	1258.7367	1258.6656	56.5	120 -	130	0	---	K.LSDELELAIEK.Q
1457.8382	1456.8309	1456.7634	46.4	393 -	406	1	---	K.AITEAEKQLNGAGR.I
1561.9388	1560.9315	1560.7817	96.0	432 -	446	0	31	R.QTAEMLAAAVQQSTL.-
1707.9303	1706.9230	1707.0117	-51.96	365 -	379	1	---	K.VMVKNPQVLINVPIK.G + Oxidation (M)
2284.3628	2283.3555	2283.2144	61.8	274 -	295	0	---	K.ENAPLGVVGTIMSNLGLEQTLK.R
2440.5128	2439.5056	2439.3155	77.9	274 -	296	1	---	K.ENAPLGVVGTIMSNLGLEQTLKR.H

No match to: 773.4587, 807.4419, 842.5599, 870.5871, 931.5710, 965.5635, 1003.6035, 1046.6100, 1062.6412, 1064.6582, 1075.6517, 1076.6699, 1118.6055, 1133.6818, 1140.6556, 1149.6929, 1158.7030, 1165.6616, 1172.7483, 1179.6837, 1277.7544, 1285.7582, 1296.7598, 1308.7634, 1320.6892, 1333.7866, 1340.7881, 1367.8244, 1373.7740, 1390.7958, 1434.8763, 1475.8714, 1488.8863, 1493.8532, 1530.8044, 1537.8684, 1545.9206, 1553.9584, 1590.9204, 1598.9555, 1615.9930, 1638.9781, 1657.9450, 1683.0081, 1690.9261, 1699.9587, 1713.9658, 1729.9719, 1764.0939, 1786.0298, 1791.8986, 1804.0504, 1822.0336, 1834.1144, 1839.0705, 1842.1348, 1858.1460, 1862.0788, 1930.0964, 1941.1169, 1955.1238, 1970.2165, 1972.1537, 1985.1441, 1998.1688, 2076.2873, 2083.1868, 2105.2985, 2126.2672, 2141.2573, 2164.2353, 2203.3329, 2211.3104, 2215.3655, 2222.2808, 2235.3499, 2274.3577, 2296.2822, 2322.3983, 2343.2148, 2357.2678, 2384.1747, 2399.2446, 2418.3632, 2592.4853, 2641.5621, 2653.5041, 2705.3800, 2717.3219, 2888.5748, 3264.6235

8. GLMM_COXB1 **Mass:** 48347 **Score:** 63 **Expect:** 0.18 **Matches:** 8

Phosphoglucosamine mutase OS=Coxiella burnetii (strain CbuK_Q154) OX=434924 GN=glmM PE=3 SV=1

Observed	Mr(expt)	Mr(calc)	ppm	Start	End	Miss	Ions	Peptide
758.4449	757.4377	757.4235	18.7	28 -	34	0	---	K.LGWAVGR.V
761.4539	760.4466	760.3967	65.7	393 -	399	0	---	K.AITEAEK.Q
1259.7439	1258.7367	1258.6656	56.5	120 -	130	0	---	K.LSDELELAIEK.Q
1457.8382	1456.8309	1456.7634	46.4	393 -	406	1	---	K.AITEAEKQLNGAGR.I
1561.9388	1560.9315	1560.7817	96.0	432 -	446	0	31	R.QTAEMLAAAVQQSTL.-
1941.1169	1940.1096	1940.0190	46.7	74 -	91	0	---	K.LTGPMPTPAIAYLTHSVR.A + Oxidation (M)
2284.3628	2283.3555	2283.2144	61.8	274 -	295	0	---	K.ENAPLGVVGTIMSNLGLEQTLK.R
2440.5128	2439.5056	2439.3155	77.9	274 -	296	1	---	K.ENAPLGVVGTIMSNLGLEQTLKR.H

No match to: 773.4587, 807.4419, 842.5599, 870.5871, 931.5710, 965.5635, 1003.6035, 1046.6100, 1062.6412, 1064.6582, 1075.6517, 1076.6699, 1118.6055, 1133.6818, 1140.6556, 1149.6929, 1158.7030, 1165.6616, 1172.7483, 1179.6837, 1234.7577, 1277.7544, 1285.7582, 1296.7598, 1308.7634, 1320.6892, 1333.7866, 1340.7881, 1367.8244, 1373.7740, 1390.7958, 1434.8763, 1475.8714, 1488.8863, 1493.8532, 1530.8044, 1537.8684, 1545.9206, 1553.9584, 1590.9204, 1598.9555, 1615.9930, 1638.9781, 1657.9450, 1683.0081, 1690.9261, 1699.9587, 1707.9303, 1713.9658, 1729.9719, 1764.0939, 1786.0298, 1791.8986, 1804.0504, 1822.0336, 1834.1144, 1839.0705, 1842.1348, 1858.1460, 1862.0788, 1930.0964, 1955.1238, 1970.2165, 1972.1537, 1985.1441, 1998.1688, 2076.2873, 2083.1868, 2105.2985, 2126.2672, 2141.2573, 2164.2353, 2203.3329, 2211.3104, 2215.3655, 2222.2808, 2235.3499, 2274.3577, 2296.2822, 2322.3983, 2343.2148, 2357.2678, 2384.1747, 2399.2446, 2418.3632, 2592.4853, 2641.5621, 2653.5041, 2705.3800, 2717.3219, 2888.5748, 3264.6235

9. GLMM_COXB2 **Mass:** 48347 **Score:** 63 **Expect:** 0.18 **Matches:** 8

Phosphoglucosamine mutase OS=Coxiella burnetii (strain CbuG_Q212) OX=434923 GN=glmM PE=3 SV=1

Observed	Mr(expt)	Mr(calc)	ppm	Start	End	Miss	Ions	Peptide
758.4449	757.4377	757.4235	18.7	28 -	34	0	---	K.LGWAVGR.V
761.4539	760.4466	760.3967	65.7	393 -	399	0	---	K.AITEAEK.Q
1259.7439	1258.7367	1258.6656	56.5	120 -	130	0	---	K.LSDELELAIEK.Q
1457.8382	1456.8309	1456.7634	46.4	393 -	406	1	---	K.AITEAEKQLNGAGR.I
1561.9388	1560.9315	1560.7817	96.0	432 -	446	0	31	R.QTAEMLAAAVQQSTL.-
1941.1169	1940.1096	1940.0190	46.7	74 -	91	0	---	K.LTGPMPTPAIAYLTHSVR.A + Oxidation (M)
2284.3628	2283.3555	2283.2144	61.8	274 -	295	0	---	K.ENAPLGVVGTIMSNLGLEQTLK.R
2440.5128	2439.5056	2439.3155	77.9	274 -	296	1	---	K.ENAPLGVVGTIMSNLGLEQTLKR.H

No match to: 773.4587, 807.4419, 842.5599, 870.5871, 931.5710, 965.5635, 1003.6035, 1046.6100, 1062.6412, 1064.6582, 1075.6517, 1076.6699, 1118.6055, 1133.6818, 1140.6556, 1149.6929, 1158.7030, 1165.6616, 1172.7483, 1179.6837, 1234.7577, 1277.7544, 1285.7582, 1296.7598, 1308.7634, 1320.6892, 1333.7866, 1340.7881, 1367.8244, 1373.7740, 1390.7958, 1434.8763, 1475.8714, 1488.8863, 1493.8532, 1530.8044, 1537.8684, 1545.9206, 1553.9584, 1590.9204, 1598.9555, 1615.9930, 1638.9781, 1657.9450, 1683.0081, 1690.9261, 1699.9587, 1707.9303, 1713.9658, 1729.9719, 1764.0939, 1786.0298, 1791.8986, 1804.0504, 1822.0336, 1834.1144, 1839.0705, 1842.1348, 1858.1460, 1862.0788, 1930.0964, 1955.1238, 1970.2165, 1972.1537, 1985.1441, 1998.1688, 2076.2873, 2083.1868, 2105.2985, 2126.2672, 2141.2573, 2164.2353, 2203.3329, 2211.3104, 2215.3655, 2222.2808, 2235.3499, 2274.3577, 2296.2822, 2322.3983, 2343.2148, 2357.2678, 2384.1747, 2399.2446, 2418.3632, 2592.4853, 2641.5621, 2653.5041, 2705.3800, 2717.3219, 2888.5748, 3264.6235

10. TRAA_RHIRD **Mass:** 123705 **Score:** 61 **Expect:** 0.25 **Matches:** 20

Conjugal transfer protein TraA OS=Rhizobium radiobacter OX=358 GN=traA PE=3 SV=1

Observed	Mr(expt)	Mr(calc)	ppm	Start	End	Miss	Ions	Peptide
773.4587	772.4514	772.4443	9.23	611 -	616	1	---	R.EKLVER.G
807.4419	806.4346	806.3956	48.3	348 -	354	0	---	R.LEATMAR.Q + Oxidation (M)
931.5710	930.5638	930.4771	93.2	462 -	469	1	---	R.GRDLLDDK.T
965.5635	964.5563	964.5342	22.9	18 -	26	0	---	R.SVVLSAAYR.H
1118.6055	1117.5982	1117.5339	57.6	617 -	626	0	---	R.GMVGEGHLFR.T + Oxidation (M)
1165.6616	1164.6543	1164.5775	66.0	286 -	295	1	---	K.SVFDERDVAK.V
1234.7577	1233.7504	1233.6354	93.3	366 -	377	0	---	R.GVSPTALDATFR.R
1367.8244	1366.8172	1366.7569	44.1	161 -	173	1	---	K.KVAVIGEDGQPVR.T
1373.7740	1372.7668	1372.7059	44.4	960 -	970	1	---	R.LQTDEQSLRQR.V
1390.7958	1389.7885	1389.7365	37.5	366 -	378	1	---	R.GVSPTALDATFRR.H
1488.8863	1487.8790	1487.7732	71.1	996 -	1009	0	---	R.NDLPAAIAYALSNR.E
1537.8684	1536.8611	1536.7719	58.1	484 -	498	1	---	K.QMAGFVDTAVRAGAK.I + Oxidation (M)
1615.9930	1614.9858	1614.8590	78.5	2 -	17	1	---	M.AIAHFSASIVSRGSGR.S
1764.0939	1763.0866	1762.9254	91.5	46 -	61	0	14	K.QGLLHEEFVLPADAPK.W
1791.8986	1790.8913	1790.9275	-20.19	382 -	398	1	---	R.LSDEQKAAIEHVAGPAR.I
1804.0504	1803.0431	1802.9122	72.6	954 -	968	1	---	R.ETVTQRLQTDEQSLR.Q
1822.0336	1821.0263	1821.0723	-25.28	270 -	285	1	---	R.ILNNPAIVLDLITREK.S
1842.1348	1841.1275	1841.0271	54.5	202 -	218	1	---	R.LNHHLALGGIDLKIDGR.S
2126.2672	2125.2599	2125.1068	72.0	416 -	436	1	---	K.AAREAWELAGYHVVGGALAGK.A
2284.3628	2283.3555	2283.1535	88.5	176 -	195	1	---	K.SGKILYELWAGSTDDFNVLR.D

No match to: 758.4449, 761.4539, 842.5599, 870.5871, 1003.6035, 1046.6100, 1062.6412, 1064.6582, 1075.6517, 1076.6699, 1133.6818, 1140.6556, 1149.6929, 1158.7030, 1172.7483, 1179.6837, 1259.7439, 1277.7544, 1285.7582, 1296.7598, 1308.7634, 1320.6892, 1333.7866, 1340.7881, 1434.8763, 1457.8382, 1475.8714, 1493.8532, 1530.8044, 1545.9206, 1553.9584, 1561.9388, 1590.9204, 1598.9555, 1638.9781, 1657.9450, 1683.0081, 1690.9261, 1699.9587, 1707.9303, 1713.9658, 1729.9719, 1786.0298, 1834.1144, 1839.0705, 1858.1460, 1862.0788, 1930.0964, 1941.1169, 1955.1238, 1970.2165, 1972.1537, 1985.1441, 1998.1688, 2076.2873, 2083.1868, 2105.2985, 2141.2573, 2164.2353, 2203.3329, 2211.3104, 2215.3655, 2222.2808, 2235.3499, 2274.3577, 2296.2822, 2322.3983, 2343.2148, 2357.2678, 2384.1747, 2399.2446, 2418.3632, 2440.5128, 2592.4853, 2641.5621, 2653.5041, 2705.3800, 2717.3219, 2888.5748, 3264.6235

11. RPOC2_SYNSC **Mass:** 148515 **Score:** 61 **Expect:** 0.25 **Matches:** 18

DNA-directed RNA polymerase subunit beta' OS=Synechococcus sp. (strain CC9605) OX=110662 GN=rpoC2 PE=3 SV=1

Observed	Mr(expt)	Mr(calc)	ppm	Start	End	Miss	Ions	Peptide
773.4587	772.4514	772.4443	9.21	488	494	0	---	R.QGNITLK.A
807.4419	806.4346	806.3705	79.5	166	172	0	---	R.GNMSQVR.Q + Oxidation (M)
1165.6616	1164.6543	1164.5887	56.3	538	547	1	---	R.SEYGGEVRLR.D
1259.7439	1258.7367	1258.6816	43.8	1024	1033	1	---	R.RPRESTILCK.K
1320.6892	1319.6820	1319.7197	-28.63	298	310	1	---	K.SFEAAGVKAVSVR.S
1333.7866	1332.7793	1332.7323	35.3	555	566	0	---	R.EVQIVTTAMTLK.D
1373.7740	1372.7668	1372.7310	26.0	483	494	1	---	R.EVTDRQGNITLK.A
1475.8714	1474.8641	1474.7497	77.6	166	178	1	---	R.GNMSQVRQLVGMR.G
1545.9206	1544.9134	1544.7981	74.6	461	474	1	13	K.ATKDVICDLAGQVR.Y
1729.9719	1728.9646	1728.8431	70.3	125	138	1	---	R.HTKVIDTWTETNER.L
1834.1144	1833.1072	1832.9381	92.2	361	378	1	---	R.TFHTGGVSTAETGVVRSK.V
1842.1348	1841.1275	1840.9618	90.0	970	985	0	---	R.LGRPYMVSPDSVLHVR.D + Oxidation (M)
1941.1169	1940.1096	1940.0102	51.2	1157	1174	0	---	R.VEDAGDTTLLPGELIELR.Q
1972.1537	1971.1464	1971.0538	47.0	986	1003	1	---	R.DGNLVQRGDGLALLVFER.Q
2105.2985	2104.2913	2104.1052	88.4	77	96	1	---	K.YATQAAVSISVDDLKVPEAK.K
2141.2573	2140.2500	2140.1052	67.7	72	91	1	---	K.DLGFKYATQAAVSISVDDLK.V
2384.1747	2383.1674	2383.0685	41.5	145	165	1	---	K.KNFDENAPLNSVWMMANSGAR.G + 2 Oxidation (M)
2888.5748	2887.5675	2887.4637	35.9	1175	1202	0	---	R.QVEDTNQAMAITGGAPAEFTPVLLGITK.A + Oxidation (M)

No match to: 758.4449, 761.4539, 842.5599, 870.5871, 931.5710, 965.5635, 1003.6035, 1046.6100, 1062.6412, 1064.6582, 1075.6517, 1076.6699, 1118.6055, 1133.6818, 1140.6556, 1149.6929, 1158.7030, 1172.7483, 1179.6837, 1234.7577, 1277.7544, 1285.7582, 1296.7598, 1308.7634, 1340.7881, 1367.8244, 1390.7958, 1434.8763, 1457.8382, 1488.8863, 1493.8532, 1530.8044, 1537.8684, 1553.9584, 1561.9388, 1590.9204, 1598.9555, 1615.9930, 1638.9781, 1657.9450, 1683.0081, 1690.9261, 1699.9587, 1707.9303, 1713.9658, 1764.0939, 1786.0298, 1791.8986, 1804.0504, 1822.0336, 1839.0705, 1858.1460, 1862.0788, 1930.0964, 1955.1238, 1970.2165, 1985.1441, 1998.1688, 2076.2873, 2083.1868, 2126.2672, 2164.2353, 2203.3329, 2211.3104, 2215.3655, 2222.2808, 2235.3499, 2274.3577, 2284.3628, 2296.2822, 2322.3983, 2343.2148, 2357.2678, 2399.2446, 2418.3632, 2440.5128, 2592.4853, 2641.5621, 2653.5041, 2705.3800, 2717.3219, 3264.6235

12. PAT_SALAR **Mass:** 50192 **Score:** 61 **Expect:** 0.28 **Matches:** 9

Putrescine aminotransferase OS=Salmonella arizonae (strain ATCC BAA-731 / CDC346-86 / RSK2980) OX=41514 GN=patA PE=3 SV=2

Observed	Mr(expt)	Mr(calc)	ppm	Start	End	Miss	Ions	Peptide
1259.7439	1258.7367	1258.6241	89.5	209	219	0	---	R.HVTFGNINAMR.M
1367.8244	1366.8172	1366.8548	-27.51	127	140	1	---	R.AILAKTLAALTPGK.L
1488.8863	1487.8790	1487.7521	85.3	44	55	1	---	K.EHVNPGFLEYRK.S
1545.9206	1544.9134	1544.8311	53.3	114	126	0	23	K.QPLHSQELLDPLR.A
1683.0081	1682.0008	1681.8536	87.5	372	385	1	---	R.QLAREYPDLVHDAR.G
1690.9261	1689.9189	1689.8356	49.3	444	459	1	---	K.SARNALAAMQVSVEEV.- + Oxidation (M)
2126.2672	2125.2599	2125.0659	91.3	4	23	0	---	R.LPSSASALACCAHALNLIEK.R
2222.2808	2221.2736	2221.0725	90.5	143	162	1	---	K.YSFFCNSGTESVEAALKLAK.A
2399.2446	2398.2373	2398.1331	43.5	259	279	1	---	R.KLCDEFGALMILDEVQTGMGR.T + Oxidation (M)

No match to: 758.4449, 761.4539, 773.4587, 807.4419, 842.5599, 870.5871, 931.5710, 965.5635, 1003.6035, 1046.6100, 1062.6412, 1064.6582, 1075.6517, 1076.6699, 1118.6055, 1133.6818, 1140.6556, 1149.6929, 1158.7030, 1165.6616, 1172.7483, 1179.6837, 1234.7577, 1277.7544, 1285.7582, 1296.7598, 1308.7634, 1320.6892, 1333.7866, 1340.7881, 1373.7740, 1390.7958, 1434.8763, 1457.8382, 1475.8714, 1493.8532, 1530.8044, 1537.8684, 1553.9584, 1561.9388, 1590.9204, 1598.9555, 1615.9930, 1638.9781, 1657.9450, 1699.9587, 1707.9303, 1713.9658, 1729.9719, 1764.0939, 1786.0298, 1791.8986, 1804.0504, 1822.0336, 1834.1144, 1839.0705, 1842.1348, 1858.1460, 1862.0788, 1930.0964, 1941.1169, 1955.1238, 1970.2165, 1972.1537, 1985.1441, 1998.1688, 2076.2873, 2083.1868, 2105.2985, 2141.2573, 2164.2353, 2203.3329, 2211.3104, 2215.3655, 2235.3499, 2274.3577, 2284.3628, 2296.2822, 2322.3983, 2343.2148, 2357.2678, 2384.1747, 2418.3632, 2440.5128, 2592.4853, 2641.5621, 2653.5041, 2705.3800, 2717.3219, 2888.5748, 3264.6235

13. PAT_SALPB **Mass:** 50078 **Score:** 61 **Expect:** 0.29 **Matches:** 9

Putrescine aminotransferase OS=Salmonella paratyphi B (strain ATCC BAA-1250 / SPB7) OX=1016998 GN=patA PE=3 SV=1

Observed	Mr(expt)	Mr(calc)	ppm	Start	End	Miss	Ions	Peptide
1367.8244	1366.8172	1366.7245	67.8	33	43	1	---	K.ALNREVIDYFK.E
1457.8382	1456.8309	1456.8362	-3.61	413	426	1	---	R.VLVAGTLNNAKTTR.I
1488.8863	1487.8790	1487.7521	85.3	44	55	1	---	K.EHVNPGFLEYRK.S
1545.9206	1544.9134	1544.8311	53.3	114	126	0	23	K.QPLHSQELLDPLR.A
1690.9261	1689.9189	1689.8356	49.3	444	459	1	---	K.SARNALAAMQVSVEEV.- + Oxidation (M)
1822.0336	1821.0263	1820.9872	21.5	194	208	1	---	K.STFRRPFMPLLPGFR.H
2076.2873	2075.2800	2075.1197	77.3	114	131	1	---	K.QPLHSQELLDPLRAMLAK.T + Oxidation (M)
2222.2808	2221.2736	2221.0725	90.5	143	162	1	---	K.YSFFCNSGTESVEAALKLAK.A
2399.2446	2398.2373	2398.1331	43.5	259	279	1	---	R.KLCDEFGALMILDEVQTGMGR.T + Oxidation (M)

No match to: 758.4449, 761.4539, 773.4587, 807.4419, 842.5599, 870.5871, 931.5710, 965.5635, 1003.6035, 1046.6100, 1062.6412, 1064.6582, 1075.6517, 1076.6699, 1118.6055, 1133.6818, 1140.6556, 1149.6929, 1158.7030, 1165.6616, 1172.7483, 1179.6837, 1234.7577, 1259.7439, 1277.7544, 1285.7582, 1296.7598, 1308.7634, 1320.6892, 1333.7866, 1340.7881, 1373.7740, 1390.7958, 1434.8763, 1475.8714, 1493.8532, 1530.8044, 1537.8684, 1553.9584, 1561.9388, 1590.9204, 1598.9555, 1615.9930, 1638.9781, 1657.9450, 1683.0081, 1699.9587, 1707.9303, 1713.9658, 1729.9719, 1764.0939, 1786.0298, 1791.8986, 1804.0504, 1834.1144, 1839.0705, 1842.1348, 1858.1460, 1862.0788, 1930.0964, 1941.1169, 1955.1238, 1970.2165, 1972.1537, 1985.1441, 1998.1688, 2083.1868, 2105.2985, 2126.2672, 2141.2573, 2164.2353, 2203.3329, 2211.3104, 2215.3655, 2235.3499, 2274.3577, 2284.3628, 2296.2822, 2322.3983, 2343.2148, 2357.2678, 2384.1747, 2418.3632, 2440.5128, 2592.4853, 2641.5621, 2653.5041, 2705.3800, 2717.3219, 2888.5748, 3264.6235

14. RPOC2_PROMS **Mass:** 150243 **Score:** 59 **Expect:** 0.43 **Matches:** 18

DNA-directed RNA polymerase subunit beta' OS=Prochlorococcus marinus (strain AS9601) OX=146891 GN=rpoC2 PE=3 SV=1

Observed	Mr(expt)	Mr(calc)	ppm	Start	End	Miss	Ions	Peptide
773.4587	772.4514	772.4443	9.21	490	496	0	---	R.QGNITLK.A
807.4419	806.4346	806.3705	79.5	166	172	0	---	R.GNMSQVR.Q + Oxidation (M)
1046.6100	1045.6027	1045.5291	70.4	253	262	0	---	R.SIVVEAEDGK.F
1062.6412	1061.6339	1061.5757	54.8	404	412	0	---	K.QAEVDFILK.I
1179.6837	1178.6765	1178.6547	18.5	799	808	1	---	R.LTYKDGELIK.S
1340.7881	1339.7808	1339.7208	44.8	413	425	1	---	K.IVPQGNNSGKAQK.I
1373.7740	1372.7668	1372.7310	26.0	485	496	1	---	K.EVTDRQGNITLK.A
1475.8714	1474.8641	1474.7497	77.6	166	178	1	---	R.GNMSQVRQLVGMR.G
1530.8044	1529.7971	1529.7508	30.3	57	71	0	---	K.THGTAITAAMADNLK.D + Oxidation (M)

1537.8684	1536.8611	1536.9715	-71.83	849	861	1	---	R.LNLVILESILVRR.D
1545.9206	1544.9134	1544.7981	74.6	463	476	1	13	K.ATKDVICDLAGQVR.Y
1713.9658	1712.9585	1712.8655	54.3	557	571	0	---	R.EVQIVTTSMSLTNFK.L + Oxidation (M)
1729.9719	1728.9646	1728.8431	70.3	125	138	1	---	R.HTKVIDTWTETNER.L
1955.1238	1954.1166	1954.0259	46.4	1159	1176	0	---	R.IEDAGDTTLLPGELIELR.Q
2141.2573	2140.2500	2140.1052	67.7	72	91	1	---	K.DLGFKYATQAAVSISVDDLK.V
2235.3499	2234.3426	2234.1907	68.0	685	704	1	---	K.DIFSQTSGIVTVTQKNDILR.E
2343.2148	2342.2075	2342.1424	27.8	951	971	1	---	K.SIKSTFCGEIEEVSNSSVTLR.Y
2888.5748	2887.5675	2887.4637	35.9	1177	1204	0	---	R.QVEDTNQAMAITGGAPAEFTPVLLGITK.A + Oxidation (M)

No match to: 758.4449, 761.4539, 842.5599, 870.5871, 931.5710, 965.5635, 1003.6035, 1064.6582, 1075.6517, 1076.6699, 1118.6055, 1133.6818, 1140.6556, 1149.6929, 1158.7030, 1165.6616, 1172.7483, 1234.7577, 1259.7439, 1277.7544, 1285.7582, 1296.7598, 1308.7634, 1320.6892, 1333.7866, 1367.8244, 1390.7958, 1434.8763, 1457.8382, 1488.8863, 1493.8532, 1553.9584, 1561.9388, 1590.9204, 1598.9555, 1615.9930, 1638.9781, 1657.9450, 1683.0081, 1690.9261, 1699.9587, 1707.9303, 1764.0939, 1786.0298, 1791.8986, 1804.0504, 1822.0336, 1834.1144, 1839.0705, 1858.1460, 1862.0788, 1930.0964, 1941.1169, 1970.2165, 1972.1537, 1985.1441, 1998.1688, 2076.2873, 2083.1868, 2105.2985, 2126.2672, 2164.2353, 2203.3329, 2211.3104, 2215.3655, 2222.2808, 2274.3577, 2284.3628, 2296.2822, 2322.3983, 2357.2678, 2384.1747, 2399.2446, 2418.3632, 2440.5128, 2592.4853, 2641.5621, 2653.5041, 2705.3800, 2717.3219, 3264.6235

15. RPOC2_PROM0 **Mass:** 150258 **Score:** 57 **Expect:** 0.67 **Matches:** 18

DNA-directed RNA polymerase subunit beta' OS=Prochlorococcus marinus (strain MIT 9301) OX=167546 GN=rpoC2 PE=3 SV=1

Observed	Mr(expt)	Mr(calc)	ppm	Start	End	Miss	Ions	Peptide
761.4539	760.4466	760.3967	65.7	922	927	1	---	R.EEDKIK.I
773.4587	772.4514	772.4443	9.21	490	496	0	---	R.QGNITLK.A
807.4419	806.4346	806.3705	79.5	166	172	0	---	R.GNMSQVR.Q + Oxidation (M)
1046.6100	1045.6027	1045.5291	70.4	253	262	0	---	R.SIVVEAEDGK.F
1062.6412	1061.6339	1061.5757	54.8	404	412	0	---	K.QAEVDFILK.I
1140.6556	1139.6483	1139.6298	16.2	1107	1116	0	---	K.DQKPLIEAAR.E
1179.6837	1178.6765	1178.6547	18.5	799	808	1	---	R.LTYKDGELIK.S
1373.7740	1372.7668	1372.7310	26.0	485	496	1	---	K.EVTDRQGNITLK.A
1475.8714	1474.8641	1474.7497	77.6	166	178	1	---	R.GNMSQVRQLVGMR.G
1537.8684	1536.8611	1536.9715	-71.83	849	861	1	---	R.LNLVILESILVRR.D
1545.9206	1544.9134	1544.7981	74.6	463	476	1	13	K.ATKDVICDLAGQVR.Y
1713.9658	1712.9585	1712.8655	54.3	557	571	0	---	R.EVQIVTTSMSLTNFK.L + Oxidation (M)
1729.9719	1728.9646	1728.8431	70.3	125	138	1	---	R.HTKVIDTWTETNER.L
1955.1238	1954.1166	1954.0259	46.4	1159	1176	0	---	R.IEDAGDTTLLPGELIELR.Q
2105.2985	2104.2913	2104.1052	88.4	77	96	1	---	K.YATQAAVSISVDDLKVPEAK.Q
2141.2573	2140.2500	2140.1052	67.7	72	91	1	---	K.DLGFKYATQAAVSISVDDLK.V
2235.3499	2234.3426	2234.1907	68.0	685	704	1	---	K.DIFSQTSGIVTVTQKNDILR.E
2888.5748	2887.5675	2887.4637	35.9	1177	1204	0	---	R.QVEDTNQAMAITGGAPAEFTPVLLGITK.A + Oxidation (M)

No match to: 758.4449, 842.5599, 870.5871, 931.5710, 965.5635, 1003.6035, 1064.6582, 1075.6517, 1076.6699, 1118.6055, 1133.6818, 1149.6929, 1158.7030, 1165.6616, 1172.7483, 1234.7577, 1259.7439, 1277.7544, 1285.7582, 1296.7598, 1308.7634, 1320.6892, 1333.7866, 1340.7881, 1367.8244, 1390.7958, 1434.8763, 1457.8382, 1488.8863, 1493.8532, 1530.8044, 1553.9584, 1561.9388, 1590.9204, 1598.9555, 1615.9930, 1638.9781, 1657.9450, 1683.0081, 1690.9261, 1699.9587, 1707.9303, 1764.0939, 1786.0298, 1791.8986, 1804.0504, 1822.0336, 1834.1144, 1839.0705, 1842.1348, 1858.1460, 1862.0788, 1930.0964, 1941.1169, 1970.2165, 1972.1537, 1985.1441, 1998.1688, 2076.2873, 2083.1868, 2126.2672, 2164.2353, 2203.3329, 2211.3104, 2215.3655, 2222.2808, 2274.3577, 2284.3628, 2296.2822, 2322.3983, 2343.2148, 2357.2678, 2384.1747, 2399.2446, 2418.3632, 2440.5128, 2592.4853, 2641.5621, 2653.5041, 2705.3800, 2717.3219, 3264.6235

16. RL9_PARXL **Mass:** 16075 **Score:** 57 **Expect:** 0.68 **Matches:** 5

50S ribosomal protein L9 OS=Paraburkholderia xenovorans (strain LB400) OX=266265 GN=rplI PE=3 SV=1

Observed	Mr(expt)	Mr(calc)	ppm	Start	End	Miss	Ions	Peptide
1003.6035	1002.5962	1002.5783	17.9	1	8	0	---	-.MQIILLEK.V + Oxidation (M)
1158.7030	1157.6958	1157.6404	47.8	72	82	0	---	K.LAGSTVQINQK.A
1713.9658	1712.9585	1712.9169	24.3	72	88	1	24	K.LAGSTVQINQKAGVDGR.L
2126.2672	2125.2599	2125.1491	52.2	62	82	1	---	K.LAAAQAQGEKLAGSTVQINQK.A
2203.3329	2202.3256	2202.1644	73.2	83	104	1	---	K.AGVDGRLFGSVTNADIAEALVK.Q

No match to: 758.4449, 761.4539, 773.4587, 807.4419, 842.5599, 870.5871, 931.5710, 965.5635, 1046.6100, 1062.6412, 1064.6582, 1075.6517, 1076.6699, 1118.6055, 1133.6818, 1140.6556, 1149.6929, 1165.6616, 1172.7483, 1179.6837, 1234.7577, 1259.7439, 1277.7544, 1285.7582, 1296.7598, 1308.7634, 1320.6892, 1333.7866, 1340.7881, 1367.8244, 1373.7740, 1390.7958, 1434.8763, 1457.8382, 1475.8714, 1488.8863, 1493.8532, 1530.8044, 1537.8684, 1545.9206, 1553.9584, 1561.9388, 1590.9204, 1598.9555, 1615.9930, 1638.9781, 1657.9450, 1683.0081, 1690.9261, 1699.9587, 1707.9303, 1729.9719, 1764.0939, 1786.0298, 1791.8986, 1804.0504, 1822.0336, 1834.1144, 1839.0705, 1842.1348, 1858.1460, 1862.0788, 1930.0964, 1941.1169, 1955.1238, 1970.2165, 1972.1537, 1985.1441, 1998.1688, 2076.2873, 2083.1868, 2105.2985, 2141.2573, 2164.2353, 2211.3104, 2215.3655, 2222.2808, 2235.3499, 2274.3577, 2284.3628, 2296.2822, 2322.3983, 2343.2148, 2357.2678, 2384.1747, 2399.2446, 2418.3632, 2440.5128, 2592.4853, 2641.5621, 2653.5041, 2705.3800, 2717.3219, 2888.5748, 3264.6235

17. RL10_THIDA **Mass:** 18640 **Score:** 56 **Expect:** 0.92 **Matches:** 7

50S ribosomal protein L10 OS=Thiobacillus denitrificans (strain ATCC 25259) OX=292415 GN=rplJ PE=3 SV=1

Observed	Mr(expt)	Mr(calc)	ppm	Start	End	Miss	Ions	Peptide
758.4449	757.4377	757.4559	-24.01	57	62	1	---	K.NTLVRR.A
1076.6699	1075.6627	1075.6390	22.0	48	56	1	---	K.EGVYLRVLK.N
1475.8714	1474.8641	1474.7814	56.1	32	44	1	---	R.GIAVEDMTQLRVK.A + Oxidation (M)
1545.9206	1544.9134	1544.7657	95.6	87	100	1	14	K.DPVAAAKVMHEFSK.T + Oxidation (M)
1862.0788	1861.0715	1860.9251	78.7	121	137	1	---	K.DVGNLASMPSREELLSK.L + Oxidation (M)
2215.3655	2214.3582	2214.2008	71.1	10	31	0	---	K.AVVAEVAAQVAAAQTVVVAEYR.G
2343.2148	2342.2075	2342.2958	-37.68	9	31	1	---	K.KAVVAEVAAQVAAAQTVVVAEYR.G

No match to: 761.4539, 773.4587, 807.4419, 842.5599, 870.5871, 931.5710, 965.5635, 1003.6035, 1046.6100, 1062.6412, 1064.6582, 1075.6517, 1118.6055, 1133.6818, 1140.6556, 1149.6929, 1158.7030, 1165.6616, 1172.7483, 1179.6837, 1234.7577, 1259.7439, 1277.7544, 1285.7582, 1296.7598, 1308.7634, 1320.6892, 1333.7866, 1340.7881, 1367.8244, 1373.7740, 1390.7958, 1434.8763, 1457.8382, 1488.8863, 1493.8532, 1530.8044, 1537.8684, 1553.9584, 1561.9388, 1590.9204, 1598.9555, 1615.9930, 1638.9781, 1657.9450, 1683.0081, 1690.9261, 1699.9587, 1707.9303, 1713.9658, 1729.9719, 1764.0939, 1786.0298, 1791.8986, 1804.0504, 1822.0336, 1834.1144, 1839.0705, 1842.1348, 1858.1460, 1930.0964, 1941.1169, 1955.1238, 1970.2165, 1972.1537, 1985.1441, 1998.1688, 2076.2873, 2083.1868, 2105.2985, 2126.2672, 2141.2573, 2164.2353, 2203.3329, 2211.3104, 2222.2808, 2235.3499, 2274.3577, 2284.3628, 2296.2822, 2322.3983, 2357.2678, 2384.1747, 2399.2446, 2418.3632, 2440.5128, 2592.4853, 2641.5621, 2653.5041, 2705.3800, 2717.3219, 2888.5748, 3264.6235

18. EPMA_SALAR **Mass:** 37284 **Score:** 55 **Expect:** 0.96 **Matches:** 3

Elongation factor P--(R)-beta-lysine ligase OS=Salmonella arizonae (strain ATCC BAA-731 / CDC346-86 / RSK2980) OX=41514 GN=epmA

Observed	Mr(expt)	Mr(calc)	ppm	Start	End	Miss	Ions	Peptide
1076.6699	1075.6627	1075.5808	76.1	20	28	1	---	R.AAIMTEIRR.F + Oxidation (M)
1561.9388	1560.9315	1560.7905	90.4	84	97	0	47	R.LLAAGCGPVFQLCR.S
1985.1441	1984.1368	1984.0378	49.9	2	19	1	---	M.SETATWQPSASVPNLLKR.A

No match to: 758.4449, 761.4539, 773.4587, 807.4419, 842.5599, 870.5871, 931.5710, 965.5635, 1003.6035, 1046.6100, 1062.6412, 1064.6582, 1075.6517, 1118.6055, 1133.6818, 1140.6556, 1149.6929, 1158.7030, 1165.6616, 1172.7483, 1179.6837, 1234.7577,

1259.7439, 1277.7544, 1285.7582, 1296.7598, 1308.7634, 1320.6892, 1333.7866, 1340.7881, 1367.8244, 1373.7740, 1390.7958, 1434.8763, 1457.8382, 1475.8714, 1488.8863, 1493.8532, 1530.8044, 1537.8684, 1545.9206, 1553.9584, 1590.9204, 1598.9555, 1615.9930, 1638.9781, 1657.9450, 1683.0081, 1690.9261, 1699.9587, 1707.9303, 1713.9658, 1729.9719, 1764.0939, 1786.0298, 1791.8986, 1804.0504, 1822.0336, 1834.1144, 1839.0705, 1842.1348, 1858.1460, 1862.0788, 1930.0964, 1941.1169, 1955.1238, 1970.2165, 1972.1537, 1998.1688, 2076.2873, 2083.1868, 2105.2985, 2126.2672, 2141.2573, 2164.2353, 2203.3329, 2211.3104, 2215.3655, 2222.2808, 2235.3499, 2274.3577, 2284.3628, 2296.2822, 2322.3983, 2343.2148, 2357.2678, 2384.1747, 2399.2446, 2418.3632, 2440.5128, 2592.4853, 2641.5621, 2653.5041, 2705.3800, 2717.3219, 2888.5748, 3264.6235

19. IF2_BACFN **Mass:** 112675 **Score:** 55 **Expect:** 1.2 **Matches:** 13
Translation initiation factor IF-2 OS=Bacteroides fragilis (strain ATCC 25285 / DSM 2151 / JCM 11019 / NCTC 9343) OX=272559 GN=i

Observed	Mr(expt)	Mr(calc)	ppm	Start	End	Miss	Ions	Peptide
1062.6412	1061.6339	1061.5618	67.9	63 -	70	1	---	R.FIQERQNK.D
1076.6699	1075.6627	1075.6026	55.8	235 -	243	0	---	K.IRPTEFVSK.I
1165.6616	1164.6543	1164.5458	93.2	734 -	742	1	---	K.AMFNERNQR.V
1259.7439	1258.7367	1258.7285	6.47	108 -	118	1	---	K.FKPVGKIDLDK.L
1296.7598	1295.7525	1295.6357	90.1	73 -	84	1	---	R.NKASVSIDGYDK.K
1340.7881	1339.7808	1339.6693	83.2	38 -	48	0	---	K.ITEEQYAMLVK.E + Oxidation (M)
1488.8863	1487.8790	1487.7467	88.9	381 -	393	1	---	K.QEVSEEDVAKQVK.E
1561.9388	1560.9315	1560.8107	77.4	690 -	704	1	20	R.NATGSIIESTLDKGR.G
1713.9658	1712.9585	1712.9937	-20.56	806 -	820	1	8	R.RIALGNFQELNVIVK.G
1842.1348	1841.1275	1840.9716	84.7	674 -	689	1	---	K.VLLEAEMLDLKANPNR.N + Oxidation (M)
2343.2148	2342.2075	2342.2477	-17.14	664 -	684	1	---	K.GLGVPELMEKVLLEAEMLDLK.A + Oxidation (M)
2384.1747	2383.1674	2383.2669	-41.73	584 -	606	1	---	R.GAKVTDIAIIIVAADDDVMPQTK.E
2888.5748	2887.5675	2887.3698	68.5	638 -	662	1	---	K.ETLAQMNYLVEEWGGKYQSQDISAK.K

No match to: 758.4449, 761.4539, 773.4587, 807.4419, 842.5599, 870.5871, 931.5710, 965.5635, 1003.6035, 1046.6100, 1064.6582, 1075.6517, 1118.6055, 1133.6818, 1140.6556, 1149.6929, 1158.7030, 1172.7483, 1179.6837, 1234.7577, 1277.7544, 1285.7582, 1308.7634, 1320.6892, 1333.7866, 1367.8244, 1373.7740, 1390.7958, 1434.8763, 1457.8382, 1475.8714, 1493.8532, 1530.8044, 1537.8684, 1545.9206, 1553.9584, 1590.9204, 1598.9555, 1615.9930, 1638.9781, 1657.9450, 1683.0081, 1690.9261, 1699.9587, 1707.9303, 1729.9719, 1764.0939, 1786.0298, 1791.8986, 1804.0504, 1822.0336, 1834.1144, 1839.0705, 1858.1460, 1862.0788, 1930.0964, 1941.1169, 1955.1238, 1970.2165, 1972.1537, 1985.1441, 1998.1688, 2076.2873, 2083.1868, 2105.2985, 2126.2672, 2141.2573, 2164.2353, 2203.3329, 2211.3104, 2215.3655, 2222.2808, 2235.3499, 2274.3577, 2284.3628, 2296.2822, 2322.3983, 2357.2678, 2399.2446, 2418.3632, 2440.5128, 2592.4853, 2641.5621, 2653.5041, 2705.3800, 2717.3219, 3264.6235

20. IF2_BACFR **Mass:** 112675 **Score:** 55 **Expect:** 1.2 **Matches:** 13
Translation initiation factor IF-2 OS=Bacteroides fragilis (strain YCH46) OX=295405 GN=infB PE=3 SV=1

Observed	Mr(expt)	Mr(calc)	ppm	Start	End	Miss	Ions	Peptide
1062.6412	1061.6339	1061.5618	67.9	63 -	70	1	---	R.FIQERQNK.D
1076.6699	1075.6627	1075.6026	55.8	235 -	243	0	---	K.IRPTEFVSK.I
1165.6616	1164.6543	1164.5458	93.2	734 -	742	1	---	K.AMFNERNQR.V
1259.7439	1258.7367	1258.7285	6.47	108 -	118	1	---	K.FKPVGKIDLDK.L
1296.7598	1295.7525	1295.6357	90.1	73 -	84	1	---	R.NKASVSIDGYDK.K
1340.7881	1339.7808	1339.6693	83.2	38 -	48	0	---	K.ITEEQYAMLVK.E + Oxidation (M)
1488.8863	1487.8790	1487.7467	88.9	381 -	393	1	---	K.QEVSEEDVAKQVK.E
1561.9388	1560.9315	1560.8107	77.4	690 -	704	1	20	R.NATGSIIESTLDKGR.G
1713.9658	1712.9585	1712.9937	-20.56	806 -	820	1	8	R.RIALGNFQELNVIVK.G
1842.1348	1841.1275	1840.9716	84.7	674 -	689	1	---	K.VLLEAEMLDLKANPNR.N + Oxidation (M)
2343.2148	2342.2075	2342.2477	-17.14	664 -	684	1	---	K.GLGVPELMEKVLLEAEMLDLK.A + Oxidation (M)
2384.1747	2383.1674	2383.2669	-41.73	584 -	606	1	---	R.GAKVTDIAIIIVAADDDVMPQTK.E
2888.5748	2887.5675	2887.3698	68.5	638 -	662	1	---	K.ETLAQMNYLVEEWGGKYQSQDISAK.K

No match to: 758.4449, 761.4539, 773.4587, 807.4419, 842.5599, 870.5871, 931.5710, 965.5635, 1003.6035, 1046.6100, 1064.6582, 1075.6517, 1118.6055, 1133.6818, 1140.6556, 1149.6929, 1158.7030, 1172.7483, 1179.6837, 1234.7577, 1277.7544, 1285.7582, 1308.7634, 1320.6892, 1333.7866, 1367.8244, 1373.7740, 1390.7958, 1434.8763, 1457.8382, 1475.8714, 1493.8532, 1530.8044, 1537.8684, 1545.9206, 1553.9584, 1590.9204, 1598.9555, 1615.9930, 1638.9781, 1657.9450, 1683.0081, 1690.9261, 1699.9587, 1707.9303, 1729.9719, 1764.0939, 1786.0298, 1791.8986, 1804.0504, 1822.0336, 1834.1144, 1839.0705, 1858.1460, 1862.0788, 1930.0964, 1941.1169, 1955.1238, 1970.2165, 1972.1537, 1985.1441, 1998.1688, 2076.2873, 2083.1868, 2105.2985, 2126.2672, 2141.2573, 2164.2353, 2203.3329, 2211.3104, 2215.3655, 2222.2808, 2235.3499, 2274.3577, 2284.3628, 2296.2822, 2322.3983, 2357.2678, 2399.2446, 2418.3632, 2440.5128, 2592.4853, 2641.5621, 2653.5041, 2705.3800, 2717.3219, 3264.6235

Search Parameters

Type of search : MS/MS Ion Search
Enzyme : Trypsin
Fixed modifications : Carbamidomethyl (C)
Variable modifications : Oxidation (M)
Mass values : Monoisotopic
Protein Mass : Unrestricted
Peptide Mass Tolerance : ± 100 ppm
Fragment Mass Tolerance: ± 2 Da
Max Missed Cleavages : 1
Instrument type : MALDI-TOF-TOF
Query1 (758.4449,1+): <no title>
Query2 (761.4539,1+): <no title>
Query3 (773.4587,1+): <no title>
Query4 (807.4419,1+): <no title>
Query5 (842.5599,1+): <no title>
Query6 (870.5871,1+): <no title>
Query7 (931.5710,1+): <no title>
Query8 (965.5635,1+): <no title>
Query9 (1003.6035,1+): <no title>
Query10 (1046.6100,1+): <no title>
Query11 (1062.6412,1+): <no title>
Query12 (1064.6582,1+): <no title>
Query13 (1075.6517,1+): <no title>
Query14 (1076.6699,1+): <no title>
Query15 (1118.6055,1+): <no title>
Query16 (1133.6818,1+): <no title>
Query17 (1140.6556,1+): <no title>
Query18 (1149.6929,1+): <no title>
Query19 (1158.7030,1+): <no title>
Query20 (1165.6616,1+): <no title>
Query21 (1172.7483,1+): <no title>
Query22 (1179.6837,1+): <no title>
Query23 (1234.7577,1+): <no title>
Query24 (1259.7439,1+): <no title>
Query25 (1277.7544,1+): <no title>
Query26 (1285.7582,1+): <no title>
Query27 (1296.7598,1+): <no title>
Query28 (1308.7634,1+): <no title>

```
 Query29 (1320.6892,1+): <no title>
 Query30 (1333.7866,1+): <no title>
 Query31 (1340.7881,1+): <no title>
 Query32 (1367.8244,1+): <no title>
 Query33 (1373.7740,1+): <no title>
 Query34 (1390.7958,1+): <no title>
 Query35 (1434.8763,1+): <no title>
 Query36 (1457.8382,1+): <no title>
 Query37 (1475.8714,1+): <no title>
 Query38 (1488.8863,1+): <no title>
 Query39 (1493.8532,1+): <no title>
 Query40 (1530.8044,1+): <no title>
 Query41 (1537.8684,1+): <no title>
 Query42 (1545.9206,1+): <no title>
 Query43 (1553.9584,1+): <no title>
 Query44 (1561.9388,1+): <no title>
 Query45 (1590.9204,1+): <no title>
 Query46 (1598.9555,1+): <no title>
 Query47 (1615.9930,1+): <no title>
 Query48 (1638.9781,1+): <no title>
 Query49 (1657.9450,1+): <no title>
 Query50 (1683.0081,1+): <no title>
 Query51 (1690.9261,1+): <no title>
 Query52 (1699.9587,1+): <no title>
 Query53 (1707.9303,1+): <no title>
 Query54 (1713.9658,1+): <no title>
 Query55 (1729.9719,1+): <no title>
 Query56 (1764.0939,1+): <no title>
 Query57 (1786.0298,1+): <no title>
 Query58 (1791.8986,1+): <no title>
 Query59 (1804.0504,1+): <no title>
 Query60 (1822.0336,1+): <no title>
 Query61 (1834.1144,1+): <no title>
 Query62 (1839.0705,1+): <no title>
 Query63 (1842.1348,1+): <no title>
 Query64 (1858.1460,1+): <no title>
 Query65 (1862.0788,1+): <no title>
 Query66 (1930.0964,1+): <no title>
 Query67 (1941.1169,1+): <no title>
 Query68 (1955.1238,1+): <no title>
 Query69 (1970.2165,1+): <no title>
 Query70 (1972.1537,1+): <no title>
 Query71 (1985.1441,1+): <no title>
 Query72 (1998.1688,1+): <no title>
 Query73 (2076.2873,1+): <no title>
 Query74 (2083.1868,1+): <no title>
 Query75 (2105.2985,1+): <no title>
 Query76 (2126.2672,1+): <no title>
 Query77 (2141.2573,1+): <no title>
 Query78 (2164.2353,1+): <no title>
 Query79 (2203.3329,1+): <no title>
 Query80 (2211.3104,1+): <no title>
 Query81 (2215.3655,1+): <no title>
 Query82 (2222.2808,1+): <no title>
 Query83 (2235.3499,1+): <no title>
 Query84 (2274.3577,1+): <no title>
 Query85 (2284.3628,1+): <no title>
 Query86 (2296.2822,1+): <no title>
 Query87 (2322.3983,1+): <no title>
 Query88 (2343.2148,1+): <no title>
 Query89 (2357.2678,1+): <no title>
 Query90 (2384.1747,1+): <no title>
 Query91 (2399.2446,1+): <no title>
 Query92 (2418.3632,1+): <no title>
 Query93 (2440.5128,1+): <no title>
 Query94 (2592.4853,1+): <no title>
 Query95 (2641.5621,1+): <no title>
 Query96 (2653.5041,1+): <no title>
 Query97 (2705.3800,1+): <no title>
 Query98 (2717.3219,1+): <no title>
 Query99 (2888.5748,1+): <no title>
Query100 (3264.6235,1+): <no title>
```

Mascot: http://www.matrixscience.com/

MALDI MS MS Data Sheet of Pus Isolate

C:\Users\madhurekha\Desktop\MALDI_Data\16th batch data 070718\BHU\3\0_B3\1\1233.7044.LIFT\1SRef

Comment 1

Comment 2

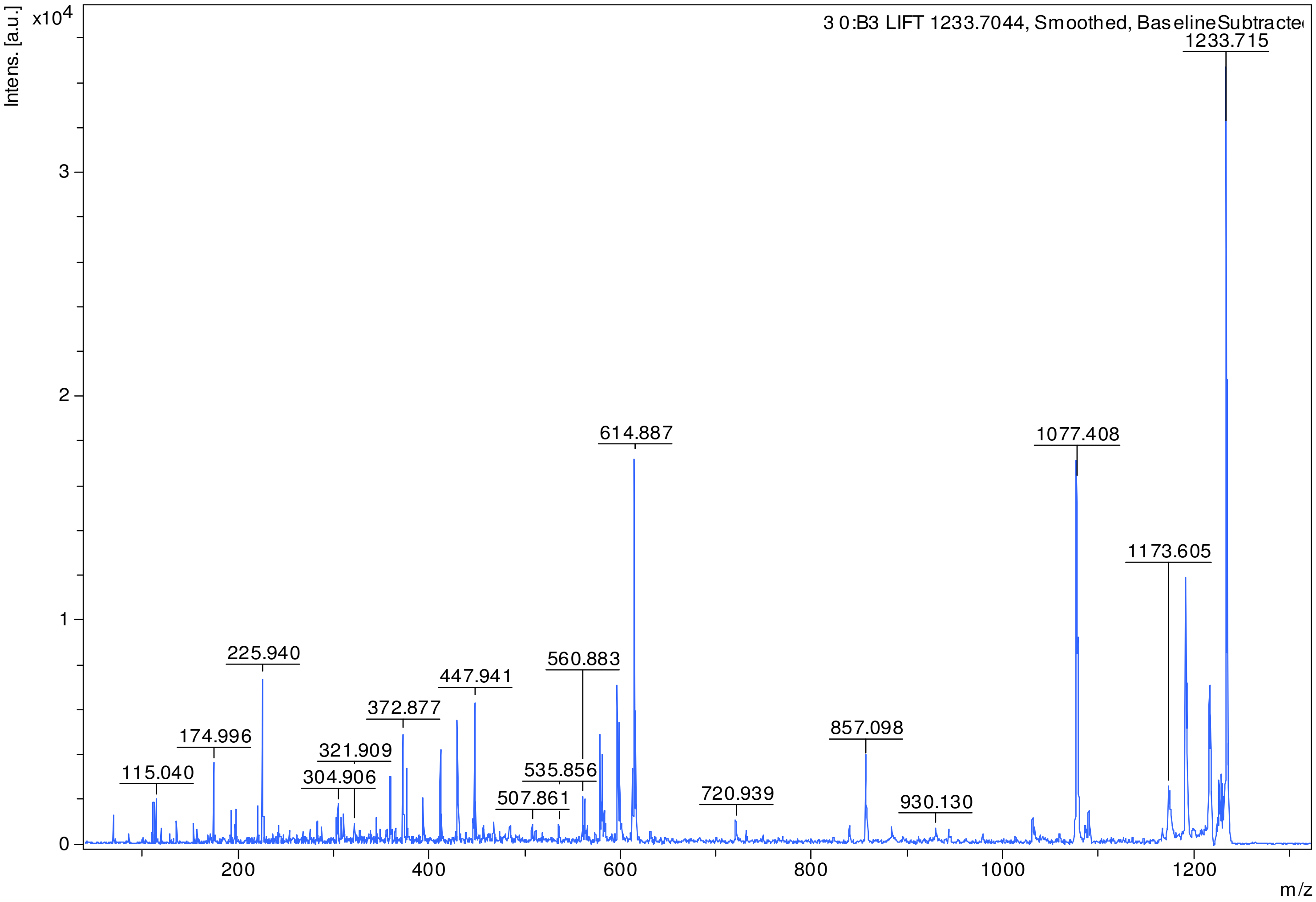

m/z	S/N	Quality Fac.	Res.	Intens.	Area
70.056	19.8	7453	449	1272.74	218
86.056	7.1	901	668	462.80	66
101.034	4.5	65	737	291.90	45
112.041	29.2	7109	874	1860.72	268
115.040	31.6	18831	946	2023.37	276
120.025	10.9	7789	957	688.41	97
129.030	6.7	381	892	421.87	69
133.024	3.8	180	939	240.42	39
136.010	15.9	11725	1013	1015.46	155
153.978	14.4	4200	1181	931.78	139
157.985	10.5	762	1158	679.85	106
168.994	6.0	539	1253	395.17	62
170.990	7.3	110	1193	480.45	80
174.996	52.3	17925	1202	3667.60	625
192.955	20.6	5685	1246	1519.87	278
197.968	21.5	3525	1337	1586.60	278
214.944	4.0	79	1237	313.07	65
216.960	3.6	44	1323	284.35	56
220.920	21.7	7880	1316	1726.37	348
225.940	92.2	28352	1432	7501.79	1425
229.922	4.8	74	1467	385.38	73
236.917	5.6	54	1209	471.35	112
242.939	10.0	137	1334	864.16	191
247.940	4.8	51	1552	420.63	82
253.970	6.7	109	1308	617.59	147
261.938	4.7	55	1219	445.65	118
267.929	5.7	212	1357	567.92	138
275.908	6.8	105	1349	719.93	182
282.919	9.7	557	1423	1057.84	262
287.931	6.7	632	1182	725.40	220
302.893	10.3	842	1597	1233.34	294
304.906	15.2	2442	1523	1837.05	463
308.026	9.6	792	1496	1189.94	309
310.883	11.2	1388	1435	1393.36	381
321.909	7.2	230	1397	965.82	283
336.906	3.2	34	1282	464.89	157
344.905	8.1	313	1588	1245.16	348
348.896	4.3	86	1256	669.86	240

m/z	S/N	Quality Fac.	Res.	Intens.	Area
359.861	18.9	9758	1560	3078.07	922
364.889	4.4	86	1376	730.03	252
372.877	28.9	21197	1578	4994.09	1545
376.878	19.4	12806	1517	3385.31	1103
393.963	12.0	1331	1769	2176.36	642
411.945	22.9	7575	1846	4351.83	1299
429.943	28.6	4831	1775	5669.09	1855
447.941	31.3	26338	1922	6483.52	2062
456.856	4.3	43	1574	901.11	359
467.843	4.5	116	1405	978.95	450
507.861	4.2	176	1296	935.40	517
535.856	3.9	274	1419	905.12	489
560.883	9.2	2664	1895	2159.73	928
563.196	8.1	609	1767	1875.93	869
565.578	3.1	57	1561	717.20	378
578.895	21.1	9556	1833	4883.03	2261
581.148	16.3	2571	1687	3754.75	1898
583.480	6.0	81	1348	1378.67	877
596.907	31.0	11345	1749	7044.03	3584
599.107	22.6	4224	1738	5131.18	2641
601.925	3.4	74	1306	771.74	532
614.887	77.9	89722	2004	17470.26	8075
720.939	5.6	490	1311	1093.32	963
731.945	3.3	328	1592	643.56	477
840.092	4.2	535	1634	779.30	687
857.098	20.7	16675	1573	3844.99	3624
884.075	4.0	131	1054	737.39	1087
930.130	3.9	221	1557	743.49	801
943.897	3.1	156	1370	600.96	753
1031.346	5.9	135	1528	1192.27	1537
1077.408	79.6	100182	1757	16226.36	19610
1086.595	4.1	195	1208	831.37	1482
1090.075	7.0	1485	2070	1450.67	1517
1167.312	4.0	159	1947	640.91	798
1173.605	16.9	1212	1363	2638.34	4734
1176.616	7.5	233	1307	1141.23	2145
1182.920	3.4	75	1312	510.06	968
1191.633	75.5	25712	1988	10926.44	13790
1198.910	5.9	58	1398	825.32	1509
1201.833	3.3	88	1335	458.06	879
1204.542	3.0	33	1769	417.86	604

m/z	S/N	Quality Fac.	Res.	Intens.	Area
1206.553	3.7	42	1770	507.85	738
1208.612	4.6	37	1520	630.43	1066
1216.643	48.1	5324	1352	6397.60	12299
1224.541	8.7	227	1805	1138.60	1659
1226.837	19.6	1831	2467	2560.14	2736
1229.158	21.1	775	2791	2731.50	2588
1231.384	17.6	43	2885	2267.64	2087
1233.715	251.6	39482	2795	32094.42	30569

C:\Users\madhurekha\Desktop\MALDI_Data\16th batch data 070718\BHU\3\0_B3\1\1631.9377.LIFT\1SRef

Comment 1

Comment 2

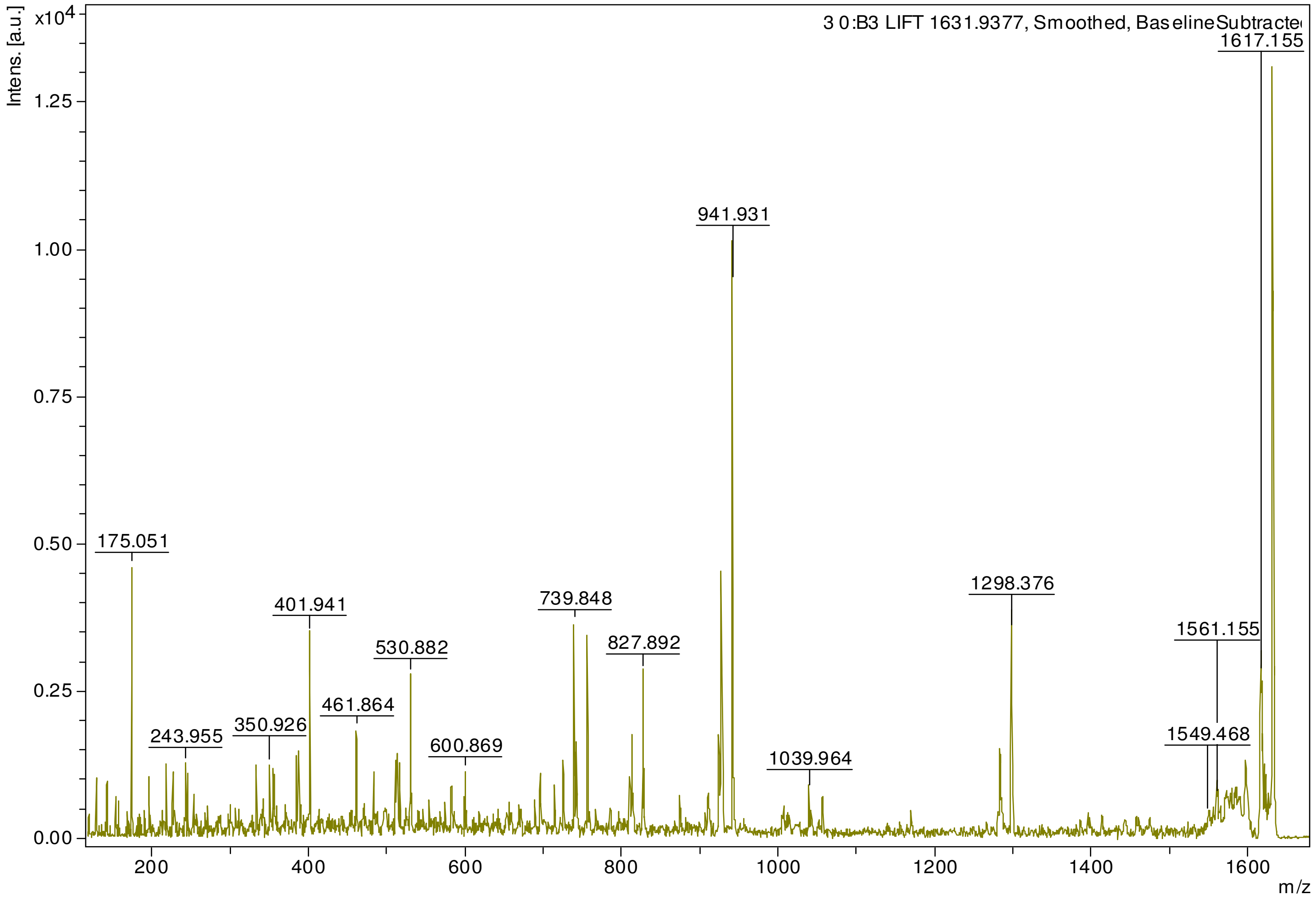

m/z	S/N	Quality Fac.	Res.	Intens.	Area
70.081	5.5	306	292	391.20	103
84.096	4.5	395	684	322.61	44
86.111	6.8	401	558	496.74	85
98.106	8.2	962	658	601.58	100
101.075	5.4	153	741	397.03	60
112.074	16.0	3352	1023	1146.22	141
120.075	5.8	1987	918	409.81	60
129.096	14.6	8466	1081	1035.14	140
136.038	3.4	110	1131	238.72	33
143.095	13.9	18129	1244	977.68	128
154.050	10.5	1035	1228	726.87	104
158.041	9.2	2380	1281	646.56	92
169.044	6.5	4186	1177	451.00	76
171.058	4.5	55	1203	312.25	52
175.051	66.9	26654	1286	4636.53	738
181.015	3.1	39	1361	216.42	34
183.039	5.1	93	1195	362.59	65
185.043	6.4	68	1215	453.24	81
191.030	6.9	152	1339	487.11	82
197.036	14.8	8338	1390	1050.60	176
201.024	3.3	34	1360	237.46	42
211.009	4.1	41	1188	299.77	64
214.044	6.3	149	1166	460.22	101
218.994	16.9	12832	1280	1275.68	262
226.983	15.4	1351	1283	1196.77	255
229.963	6.0	396	1200	473.76	110
232.030	3.7	39	1336	290.51	61
243.955	15.6	1768	1261	1281.53	302
246.009	13.4	3268	1194	1110.05	279
254.008	9.2	288	1290	792.01	191
257.993	4.8	92	1299	416.42	101
262.943	3.4	54	1331	305.85	74
271.988	6.2	169	1113	571.80	173
285.976	4.1	62	1254	394.64	112
288.017	4.3	50	1022	411.87	145
300.927	5.6	78	1310	576.95	167
308.040	4.7	86	1260	501.06	155
311.947	4.7	103	1395	504.63	143

C:\Users\madhurekha\Desktop\MALDI_Data\16th batch data 070718\BHU\3\0_B3\1\1631.9377.LIFT\1SRef

m/z	S/N	Quality Fac.	Res.	Intens.	Area
333.897	10.7	2453	1354	1267.78	400
342.964	5.9	88	1354	732.17	239
350.926	9.8	1369	1400	1263.72	409
355.925	10.4	242	1253	1367.98	504
359.958	4.2	76	1266	562.06	208
370.983	4.3	87	1103	606.03	267
384.937	9.7	2415	1587	1423.28	455
388.048	10.2	1945	1265	1498.54	607
390.883	4.1	127	1268	609.85	249
401.941	22.7	133935	1667	3485.68	1118
461.864	10.5	4257	1499	1886.50	799
471.892	3.6	30	1348	653.72	316
483.910	6.2	221	1687	1150.25	459
511.898	7.2	593	1724	1400.40	588
513.896	7.2	843	1789	1409.68	573
516.998	6.6	844	1639	1300.45	581
530.882	14.0	71048	1945	2798.35	1090
554.882	3.3	64	1588	677.93	343
574.874	3.1	101	1564	647.74	348
582.854	4.7	145	1696	979.99	495
600.869	5.5	458	2103	1164.30	497
689.830	3.2	97	1916	687.82	389
696.865	5.4	357	1979	1162.53	646
714.862	4.5	300	2019	958.37	542
725.923	6.4	675	1780	1353.33	886
739.848	17.6	23048	2165	3690.41	2041
742.941	7.8	1610	2130	1635.81	925
756.858	16.2	16238	2176	3361.28	1911
810.848	5.8	240	1938	1168.31	824
813.883	8.8	2795	2244	1768.32	1083
827.892	14.6	28802	2205	2848.40	1820
874.808	4.2	228	1990	789.58	607
910.860	4.6	95	2060	855.68	675
924.891	10.3	862	2115	1890.84	1487
927.849	24.7	7827	1926	4556.99	3955
941.931	52.1	39009	2097	9462.53	7716
1007.816	3.0	43	2471	511.91	393
1039.964	5.0	727	2153	838.13	777
1057.026	4.1	1120	1669	687.27	843
1283.774	9.8	1104	1495	1547.23	2950
1298.376	21.9	37476	2020	3549.82	5111

m/z	S/N	Quality Fac.	Res.	Intens.	Area
1549.468	3.6	49	1722	438.38	1023
1558.739	3.3	57	1733	393.17	921
1561.155	6.1	207	2025	725.33	1458
1564.765	4.4	190	1736	507.72	1192
1571.892	5.6	88	1744	637.08	1508
1574.110	5.8	255	1982	671.54	1403
1578.020	5.8	369	2052	656.94	1342
1580.233	4.0	184	1974	451.43	958
1582.181	4.8	126	2378	547.18	965
1584.441	6.6	350	1977	735.45	1565
1586.796	4.5	112	1761	505.56	1208
1589.317	5.9	206	1765	631.46	1513
1597.663	12.7	663	1774	1385.64	3336
1600.560	3.4	72	2065	374.23	775
1617.155	27.8	3399	1796	2815.07	6851
1620.658	6.9	220	3013	700.10	1018
1622.919	8.0	1073	3950	808.21	903
1631.919	110.6	23519	2965	10795.23	16204

C:\Users\madhurekha\Desktop\MALDI_Data\16th batch data 070718\BHU\3\0_B3\1\2229.3184.LIFT\1SRef

Comment 1

Comment 2

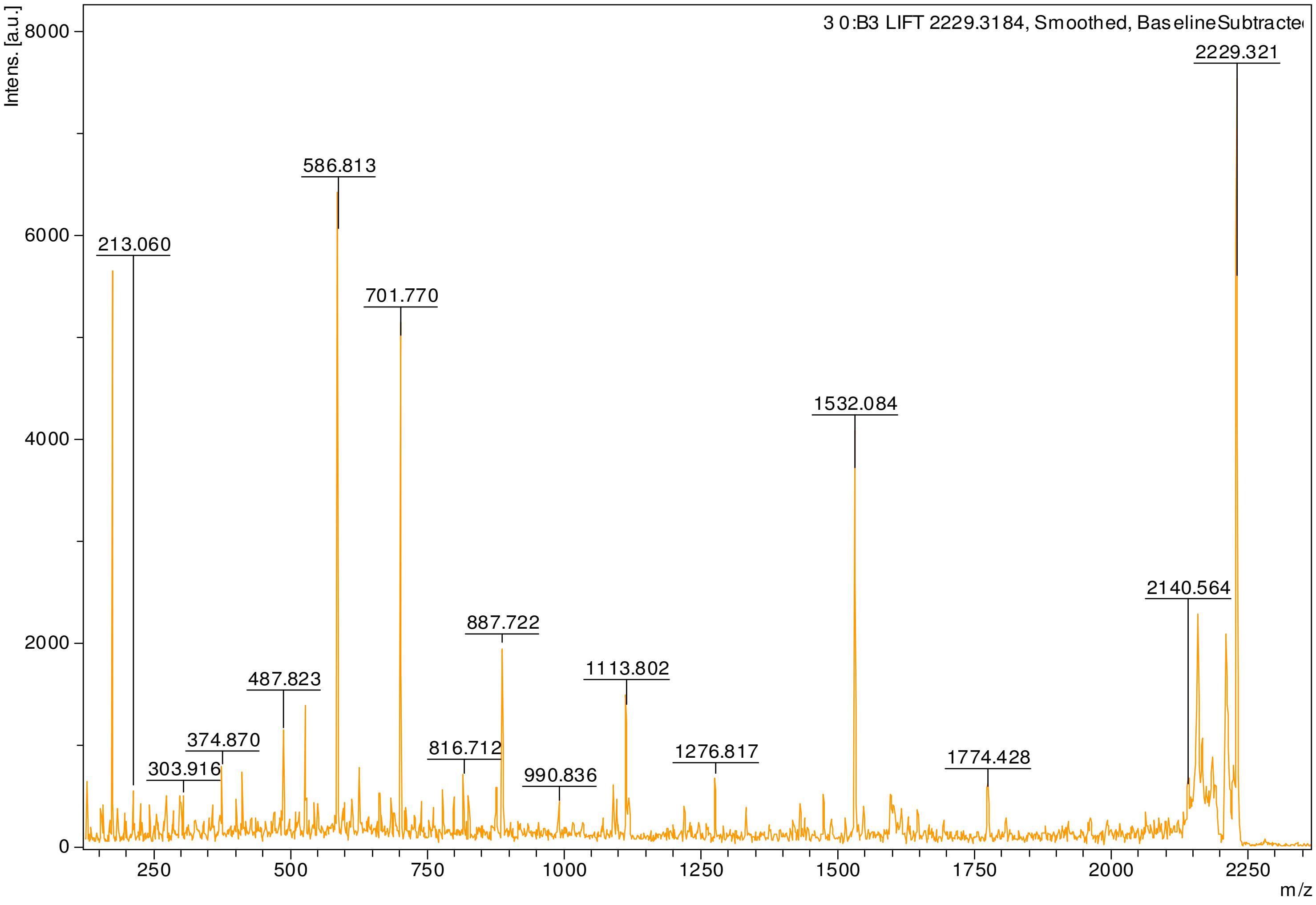

m/z	S/N	Quality Fac.	Res.	Intens.	Area
70.042	4.9	460	378	325.90	66
86.091	7.5	2602	724	493.45	65
102.071	3.6	520	1202	231.45	22
112.086	22.7	23550	1211	1500.50	156
115.092	3.5	33	1122	231.62	27
120.069	3.1	519	1351	206.46	21
129.103	9.6	12552	1234	631.99	75
154.065	5.7	220	1169	376.91	57
158.065	6.3	733	1282	415.02	59
171.064	4.2	86	1200	276.81	46
175.073	83.8	14652	1164	5622.16	989
185.084	5.6	408	1138	384.29	74
197.069	5.0	809	1059	349.62	77
199.071	3.8	155	930	262.82	67
201.018	3.4	214	867	242.80	67
213.060	7.7	1300	1078	563.13	133
227.004	5.1	367	1036	385.51	102
243.010	4.9	134	1181	394.79	99
255.971	3.9	254	1201	324.30	85
271.988	3.3	52	1035	281.95	92
274.048	6.2	1532	1042	544.22	177
286.969	4.0	71	945	367.00	139
297.922	5.0	241	1330	478.42	134
299.950	4.4	44	1282	419.88	123
303.916	5.0	732	1281	487.37	146
374.870	6.4	1250	1259	769.30	300
400.847	3.7	32	1221	468.29	204
412.832	5.3	386	1212	693.92	316
487.823	7.6	1367	1644	1118.86	463
527.729	8.9	1460	1522	1358.75	671
586.813	37.9	17113	1731	6017.36	3003
612.840	3.5	80	1360	548.39	372
626.721	4.7	368	1734	745.21	408
663.886	3.2	111	1361	512.00	387
684.763	3.3	37	2251	521.89	249
701.770	31.6	38146	1884	4976.98	2936
778.892	3.9	149	1739	586.07	434
799.739	3.2	243	1805	473.54	351

C:\Users\madhurekha\Desktop\MALDI_Data\16th batch data 070718\BHU\3\0_B3\1\2229.3184.LIFT\1SRef

m/z	S/N	Quality Fac.	Res.	Intens.	Area
816.712	4.5	454	1913	672.37	485
825.875	3.6	68	2084	541.57	365
877.840	4.1	244	1565	593.96	583
887.722	13.7	24233	2335	1969.24	1318
990.836	3.1	117	1971	416.33	391
1090.832	5.2	142	2467	693.49	609
1096.795	3.5	138	2435	474.00	426
1113.802	10.2	4859	2298	1357.47	1324
1219.743	3.1	113	2856	416.05	380
1276.817	5.1	1395	2289	674.79	832
1332.776	3.1	194	2497	409.74	500
1431.684	3.2	123	2909	444.57	529
1474.992	3.7	476	2224	517.13	851
1532.084	26.5	20840	2108	3673.87	6844
1546.821	3.2	38	2620	441.91	675
1597.726	3.8	80	2558	539.44	901
1774.428	4.1	478	1972	553.84	1478
2140.564	6.1	209	2379	568.07	1637
2145.668	3.6	198	2382	331.28	956
2148.640	3.4	72	2613	318.59	838
2151.806	4.9	99	2464	451.37	1263
2155.250	8.2	1082	3706	772.99	1439
2158.361	20.3	15675	2871	1889.69	4551
2165.553	6.7	216	2405	582.54	1682
2168.392	5.7	433	2688	508.40	1315
2170.956	3.1	92	2820	282.84	699
2174.102	4.6	181	2415	403.23	1165
2178.610	4.7	197	2421	411.88	1190
2182.088	3.9	105	2424	335.89	971
2185.266	8.8	364	2425	748.40	2167
2190.292	6.0	170	2432	520.19	1506
2207.882	6.1	130	2454	492.14	1432
2210.288	19.4	8137	2726	1619.31	4246
2213.753	10.2	1696	3812	860.39	1616
2218.143	4.4	157	6434	363.98	406
2222.442	6.4	644	4927	527.90	772
2224.719	5.4	35	4640	447.36	695
2229.321	68.0	50838	3615	5564.42	11129

C:\Users\madhurekha\Desktop\MALDI_Data\16th batch data 070718\BHU\3\0_B3\1\1SRef

Comment 1

Comment 2

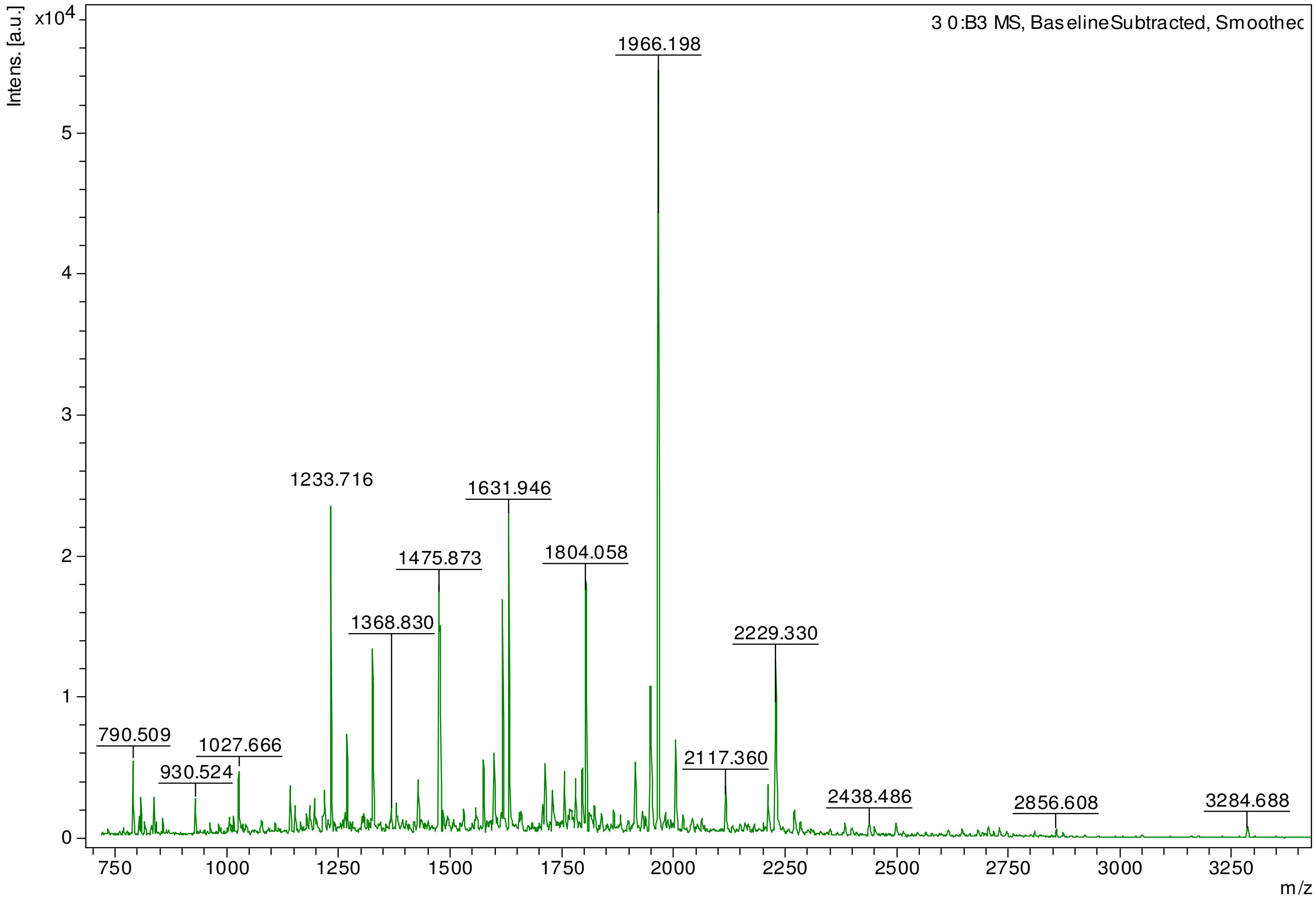

m/z	S/N	Quality Fac.	Res.	Intens.	Area
790.509	31.7	63576	5626	5388.92	1331
804.332	7.3	628	4936	1246.93	349
808.445	16.0	11428	5560	2753.22	699
837.559	15.6	14589	6098	2755.55	681
857.505	6.6	1667	5225	1179.25	345
930.524	13.7	6221	5839	2665.18	797
1006.645	6.1	488	4904	1261.25	502
1015.633	6.9	2270	7095	1451.76	418
1027.666	22.7	7210	7008	4778.47	1424
1142.674	14.3	6028	6169	3465.96	1354
1153.655	8.0	1498	5782	1960.22	830
1186.730	7.5	732	6058	1897.16	805
1197.658	8.0	365	4650	2052.49	1141
1219.644	10.2	1037	4037	2680.12	1782
1233.716	91.1	47471	6626	24270.98	10072
1269.724	25.1	15834	6787	6950.25	2966
1326.788	46.9	20428	6885	13464.13	6106
1368.830	6.5	1074	6642	1927.21	949
1380.867	6.6	544	5868	1965.26	1112
1428.921	11.7	3694	6251	3575.18	2029
1475.873	55.4	26434	7013	17148.72	9244
1477.849	24.6	10362	6550	7606.92	4367
1575.015	16.3	13125	7540	5133.41	2871
1598.984	19.1	10641	7281	5995.02	3642
1617.928	52.3	45964	7184	16354.19	10267
1631.946	72.9	62393	7498	22703.40	13937
1712.968	16.1	15133	7709	4863.80	3209
1729.003	10.2	5440	8430	3078.01	1867
1756.087	14.0	4531	7242	4146.67	3028
1781.094	9.6	810	5375	2838.58	2853
1797.115	11.7	7277	7642	3420.05	2497
1804.058	59.3	69867	7902	17300.11	12317
1915.149	16.0	7863	7463	4232.01	3479
1947.142	12.1	581	6974	3069.99	2721
1949.132	32.9	16438	7609	8314.25	6919
1952.152	8.2	901	4978	2071.61	2588
1966.198	177.6	30339	7372	44013.54	38141
2005.247	23.9	21912	7703	5639.58	4778

C:\Users\madhurekha\Desktop\MALDI_Data\16th batch data 070718\BHU\3\0_B3\1\1SRef

m/z	S/N	Quality Fac.	Res.	Intens.	Area
2117.360	14.3	4351	7444	2809.44	2628
2211.316	16.0	1493	7705	2721.93	2600
2229.330	56.5	7475	7739	9375.13	9076
2271.365	8.9	3328	7604	1386.29	1393
2438.486	6.1	925	6541	699.07	896
2856.608	6.5	4103	8169	361.51	476
3284.688	16.3	4338	8332	411.68	680

Mascot Search Results

```
User          : Madhurarekha
Email         : sanovar@sandor.co.in
Search title  :
MS data file  : DATA.TXT
Database      : SwissProt 2018_06 (557713 sequences; 200130199 residues)
Taxonomy      : Bacteria (Eubacteria) (333552 sequences)
Timestamp     : 11 Jul 2018 at 11:45:45 GMT
Warning       : A Peptide summary report will usually give a much clearer picture of MS/MS search results.
Top Score     : 123 for EFTU_KLEP7, Elongation factor Tu OS=Klebsiella pneumoniae subsp. pneumoniae (strain ATCC 700721 / MGH 78578)
```

Mascot Score Histogram

Protein score is -10*Log(P), where P is the probability that the observed match is a random event.
Protein scores greater than 68 are significant (p<0.05).
Protein scores are derived from ions scores as a non-probabilistic basis for ranking protein hits.

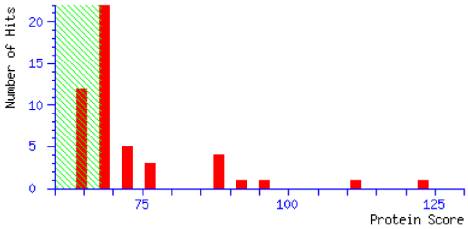

Protein Summary Report

Format As | Protein Summary (deprecated) | Help

Significance threshold p< 0.05 Max. number of hits 20

Preferred taxonomy All entries

Overview Table

Click on column header to jump to entry in results list.
Move mouse over any indicator to highlight identical peptides.
Click on an indicator to see details of individual match.
Use check boxes to select sub-set of queries for new search.

Mouse over: -Query-
-Accession- -Sequence-

Hit:	1	2	3	4	5	6	7	8	9	10	11	12	13	14	15	16	17	18	19	20
☑ 790.5086 (1+)																				
☑ 804.3324 (1+)																				
☑ 808.4445 (1+)																				
☑ 817.4935 (1+)																				
☑ 832.4057 (1+)																				
☑ 837.5595 (1+)																				
☑ 842.5625 (1+)																				
☑ 857.5055 (1+)																				
☑ 930.5245 (1+)																				
☑ 964.5936 (1+)																				
☑ 982.6070 (1+)																				
☑ 986.6133 (1+)																				
☑ 1006.6445 (1+)																				
☑ 1015.6326 (1+)																				
☑ 1027.6664 (1+)																				
☑ 1042.6300 (1+)																				
☑ 1078.6445 (1+)																				
☑ 1107.8344 (1+)																				
☑ 1142.6742 (1+)																				
☑ 1153.6546 (1+)																				
☑ 1165.6966 (1+)																				
☑ 1179.7073 (1+)																				
☑ 1186.7303 (1+)																				
☑ 1197.6582 (1+)																				
☑ 1214.7097 (1+)																				
☑ 1219.6443 (1+)																				
☑ 1233.7159 (1+)	●	●		●				●												
☑ 1240.6944 (1+)																				
☑ 1265.7392 (1+)																				
☑ 1269.7243 (1+)																				

☑ 1326.7883 (1+)			●																	
☑ 1368.8298 (1+)																				
☑ 1380.8667 (1+)																				
☑ 1428.9212 (1+)																				
☑ 1450.8531 (1+)																				
☑ 1475.8727 (1+)					●	●	●				●		●		●	●	●			
☑ 1477.8489 (1+)																				
☑ 1484.8980 (1+)																				
☑ 1530.8700 (1+)																				
☑ 1557.9283 (1+)																				
☑ 1575.0154 (1+)																				
☑ 1598.9839 (1+)																				
☑ 1617.9280 (1+)																				
☑ 1631.9464 (1+)					●	●	●	●		●	●			●	●	●		●	●	●
☑ 1654.9735 (1+)																				
☑ 1658.9825 (1+)																				
☑ 1708.0185 (1+)																				
☑ 1712.9684 (1+)																				
☑ 1724.0543 (1+)																				
☑ 1729.0035 (1+)																				
☑ 1733.0874 (1+)																				
☑ 1756.0868 (1+)																				
☑ 1781.0936 (1+)																				
☑ 1797.1145 (1+)																				
☑ 1804.0575 (1+)																				
☑ 1813.1035 (1+)																				
☑ 1824.0974 (1+)																				
☑ 1839.0932 (1+)																				
☑ 1850.0717 (1+)																				
☑ 1882.1314 (1+)																				
☑ 1900.1334 (1+)																				
☑ 1915.1486 (1+)																				
☑ 1931.1352 (1+)																				
☑ 1938.1476 (1+)																				
☑ 1947.1417 (1+)																				
☑ 1949.1320 (1+)																				
☑ 1952.1522 (1+)																				
☑ 1966.1980 (1+)	●	●		●					●				●	●			●			
☑ 1981.1803 (1+)																				
☑ 1994.1847 (1+)																				
☑ 2005.2465 (1+)																				
☑ 2021.1854 (1+)																				
☑ 2042.2539 (1+)																				
☑ 2044.2373 (1+)																				
☑ 2064.3065 (1+)																				
☑ 2117.3601 (1+)																				
☑ 2149.2508 (1+)																				
☑ 2181.2406 (1+)																				
☑ 2211.3162 (1+)																				
☑ 2215.2391 (1+)																				
☑ 2229.3298 (1+)									●			●								
☑ 2271.3649 (1+)																				
☑ 2284.3753 (1+)																				
☑ 2384.2549 (1+)																				
☑ 2399.3191 (1+)																				
☑ 2438.4857 (1+)																				
☑ 2450.5041 (1+)																				
☑ 2498.5023 (1+)																				
☑ 2615.5335 (1+)																				
☑ 2645.5873 (1+)																				
☑ 2681.5950 (1+)																				
☑ 2705.4362 (1+)																				
☑ 2717.3922 (1+)																				
☑ 2729.6061 (1+)																				
☑ 2745.6012 (1+)																				
☑ 2808.6023 (1+)																				
☑ 2856.6076 (1+)																				
☑ 2872.6380 (1+)																				
☑ 3048.6449 (1+)																				
☑ 3284.6878 (1+)																				

Select All | Select None | Search Selected

Index

	Accession	Mass	Score	Description
1.	EFTU_KLEP7	43390	123	Elongation factor Tu OS=Klebsiella pneumoniae subsp. pneumoniae (strain ATCC 700721 / MGH 78578) OX=27
2.	EFTU1_HAEI8	43384	112	Elongation factor Tu 1 OS=Haemophilus influenzae (strain 86-028NP) OX=281310 GN=tuf1 PE=3 SV=1
3.	CISY_SALTY	48474	96	Citrate synthase OS=Salmonella typhimurium (strain LT2 / SGSC1412 / ATCC 700720) OX=99287 GN=gltA PE=3
4.	EFTU_AERHH	43525	94	Elongation factor Tu OS=Aeromonas hydrophila subsp. hydrophila (strain ATCC 7966 / DSM 30187 / JCM 102
5.	EFTU_COXBN	43613	88	Elongation factor Tu OS=Coxiella burnetii (strain Dugway 5J108-111) OX=434922 GN=tuf1 PE=3 SV=1
6.	EFTU_COXBR	43613	88	Elongation factor Tu OS=Coxiella burnetii (strain RSA 331 / Henzerling II) OX=360115 GN=tuf1 PE=3 SV=1
7.	EFTU_COXBU	43613	88	Elongation factor Tu OS=Coxiella burnetii (strain RSA 493 / Nine Mile phase I) OX=227377 GN=tufA PE=1
8.	EFTU_MARHV	44034	87	Elongation factor Tu OS=Marinobacter hydrocarbonoclasticus (strain ATCC 700491 / DSM 11845 / VT8) OX=3
9.	DNAK_BRADU	68364	76	Chaperone protein DnaK OS=Bradyrhizobium diazoefficiens (strain JCM 10833 / IAM 13628 / NBRC 14792 / U
10.	ADH_THEBR	37851	75	NADP-dependent isopropanol dehydrogenase OS=Thermoanaerobacter brockii OX=29323 GN=adh PE=1 SV=1
11.	TOP1_STAS1	79764	75	DNA topoisomerase 1 OS=Staphylococcus saprophyticus subsp. saprophyticus (strain ATCC 15305 / DSM 2022
12.	HSCA_AROAE	66854	74	Chaperone protein HscA homolog OS=Aromatoleum aromaticum (strain EbN1) OX=76114 GN=hscA PE=3 SV=1
13.	LEU1_BUCUN	56434	72	2-isopropylmalate synthase (Fragment) OS=Buchnera aphidicola subsp. Uroleucon sonchi OX=118118 GN=leuA
14.	SECA_PROM4	107505	71	Protein translocase subunit SecA OS=Prochlorococcus marinus (strain MIT 9211) OX=93059 GN=secA PE=3 SV
15.	RPOA_PROM1	34244	71	DNA-directed RNA polymerase subunit alpha OS=Prochlorococcus marinus (strain NATL1A) OX=167555 GN=rpoA
16.	RPOA_PROMT	34244	71	DNA-directed RNA polymerase subunit alpha OS=Prochlorococcus marinus (strain NATL2A) OX=59920 GN=rpoA
17.	PROA_BRUSU	44300	69	Gamma-glutamyl phosphate reductase OS=Brucella suis biovar 1 (strain 1330) OX=204722 GN=proA PE=3 SV=1
18.	EFTU_HERAU	43768	69	Elongation factor Tu OS=Herpetosiphon aurantiacus OX=65 GN=tuf PE=3 SV=1
19.	EFTU1_HALHL	43283	69	Elongation factor Tu 1 OS=Halorhodospira halophila (strain DSM 244 / SL1) OX=349124 GN=tuf1 PE=3 SV=1
20.	EFTU2_HALHL	43269	69	Elongation factor Tu 2 OS=Halorhodospira halophila (strain DSM 244 / SL1) OX=349124 GN=tuf2 PE=3 SV=1

Results List

1. EFTU_KLEP7 **Mass:** 43390 **Score:** 123 **Expect:** 1.7e-07 **Matches:** 14

Elongation factor Tu OS=Klebsiella pneumoniae subsp. pneumoniae (strain ATCC 700721 / MGH 78578) OX=272620 GN=tufA PE=3 SV=1

Observed	Mr(expt)	Mr(calc)	ppm	Start - End	Miss	Ions	Peptide
837.5595	836.5522	836.4868	78.2	118 - 124	0	---	R.EHILLGR.Q
1027.6664	1026.6591	1026.5822	74.9	271 - 280	0	---	R.AGENVGVLLR.G
1214.7097	1213.7024	1213.6230	65.4	305 - 314	0	---	K.FESEVYILSK.D
1233.7159	1232.7086	1232.6091	80.8	326 - 334	0	10	K.GYRPQFYFR.T
1729.0035	1727.9962	1727.8366	92.4	305 - 319	1	---	K.FESEVYILSKDEGGR.H
1781.0936	1780.0863	1779.9375	83.6	359 - 374	0	---	K.MVVTLIHPIAMDDGLR.F
1797.1145	1796.1072	1795.9325	97.3	359 - 374	0	---	K.MVVTLIHPIAMDDGLR.F + Oxidation (M)
1804.0575	1803.0502	1802.8799	94.5	60 - 75	0	---	R.GITINTSHVEYDTPTR.H
1813.1035	1812.0963	1811.9274	93.2	359 - 374	0	---	K.MVVTLIHPIAMDDGLR.F + 2 Oxidation (M)
1966.1980	1965.1907	1965.0207	86.5	189 - 205	0	64	K.IIELAGHLDTYIPEPER.A
2117.3601	2116.3529	2116.1568	92.6	206 - 224	0	---	R.AIDKPFLLPIEDVFSISGR.G
2284.3753	2283.3680	2283.2232	63.4	359 - 378	1	---	K.MVVTLIHPIAMDDGLRFAIR.E + Oxidation (M)
2729.6061	2728.5988	2728.3346	96.8	91 - 117	0	---	K.NMITGAAQMDGAILVVAATDGPMPQTR.E
2745.6012	2744.5939	2744.3296	96.3	91 - 117	0	---	K.NMITGAAQMDGAILVVAATDGPMPQTR.E + Oxidation (M)

No match to: 790.5086, 804.3324, 808.4445, 817.4935, 832.4057, 842.5625, 857.5055, 930.5245, 964.5936, 982.6070, 986.6133, 1006.6445, 1015.6326, 1042.6300, 1078.6445, 1107.8344, 1142.6742, 1153.6546, 1165.6966, 1179.7073, 1186.7303, 1197.6582, 1219.6443, 1240.6944, 1265.7392, 1269.7243, 1326.7883, 1368.8298, 1380.8667, 1428.9212, 1450.8531, 1475.8727, 1477.8489, 1484.8980, 1530.8700, 1557.9283, 1575.0154, 1598.9839, 1617.9280, 1631.9464, 1654.9735, 1658.9825, 1708.0185, 1712.9684, 1724.0543, 1733.0874, 1756.0868, 1824.0974, 1839.0932, 1850.0717, 1882.1314, 1900.1334, 1915.1486, 1931.1352, 1938.1476, 1947.1417, 1949.1320, 1952.1522, 1981.1803, 1994.1847, 2005.2465, 2021.1854, 2042.2539, 2044.2373, 2064.3065, 2149.2508, 2181.2406, 2211.3162, 2215.2391, 2229.3298, 2271.3649, 2384.2549, 2399.3191, 2438.4857, 2450.5041, 2498.5023, 2615.5335, 2645.5873, 2681.5950, 2705.4362, 2717.3922, 2808.6023, 2856.6076, 2872.6380, 3048.6449, 3284.6878

2. EFTU1_HAEI8 **Mass:** 43384 **Score:** 112 **Expect:** 2.1e-06 **Matches:** 10

Elongation factor Tu 1 OS=Haemophilus influenzae (strain 86-028NP) OX=281310 GN=tuf1 PE=3 SV=1

Observed	Mr(expt)	Mr(calc)	ppm	Start - End	Miss	Ions	Peptide
837.5595	836.5522	836.4868	78.2	118 - 124	0	---	R.EHILLGR.Q
842.5625	841.5553	841.5134	49.8	232 - 238	1	---	R.VERGIIR.T
1233.7159	1232.7086	1232.6091	80.8	326 - 334	0	10	K.GYRPQFYFR.T
1575.0154	1574.0081	1573.8199	120	239 - 253	1	---	R.TGDEVEIVGIKDTAK.T
1598.9839	1597.9766	1597.9039	45.5	235 - 249	1	---	R.GIIRTGDEVEIVGIK.D
1804.0575	1803.0502	1802.8799	94.5	60 - 75	0	---	R.GITINTSHVEYDTPTR.H
1966.1980	1965.1907	1965.0207	86.5	189 - 205	0	64	K.ILELAGHLDTYIPEPER.A
2117.3601	2116.3529	2116.1205	110	206 - 224	0	---	R.AIDQPFLLPIEDVFSISGR.G
2729.6061	2728.5988	2728.3346	96.8	91 - 117	0	---	K.NMITGAAQMDGAILVVAATDGPMPQTR.E
2745.6012	2744.5939	2744.3296	96.3	91 - 117	0	---	K.NMITGAAQMDGAILVVAATDGPMPQTR.E + Oxidation (M)

No match to: 790.5086, 804.3324, 808.4445, 817.4935, 832.4057, 857.5055, 930.5245, 964.5936, 982.6070, 986.6133, 1006.6445, 1015.6326, 1027.6664, 1042.6300, 1078.6445, 1107.8344, 1142.6742, 1153.6546, 1165.6966, 1179.7073, 1186.7303, 1197.6582, 1214.7097, 1219.6443, 1240.6944, 1265.7392, 1269.7243, 1326.7883, 1368.8298, 1380.8667, 1428.9212, 1450.8531, 1475.8727, 1477.8489, 1484.8980, 1530.8700, 1557.9283, 1617.9280, 1631.9464, 1654.9735, 1658.9825, 1708.0185, 1712.9684, 1724.0543, 1729.0035, 1733.0874, 1756.0868, 1781.0936, 1797.1145, 1813.1035, 1824.0974, 1839.0932, 1850.0717, 1882.1314, 1900.1334, 1915.1486, 1931.1352, 1938.1476, 1947.1417, 1949.1320, 1952.1522, 1981.1803, 1994.1847, 2005.2465, 2021.1854, 2042.2539, 2044.2373, 2064.3065, 2149.2508, 2181.2406, 2211.3162, 2215.2391, 2229.3298, 2271.3649, 2284.3753, 2384.2549, 2399.3191, 2438.4857, 2450.5041, 2498.5023, 2615.5335, 2645.5873, 2681.5950, 2705.4362, 2717.3922, 2808.6023, 2856.6076, 2872.6380, 3048.6449, 3284.6878

3. CISY_SALTY **Mass:** 48474 **Score:** 96 **Expect:** 9.2e-05 **Matches:** 15

Citrate synthase OS=Salmonella typhimurium (strain LT2 / SGSC1412 / ATCC 700720) OX=99287 GN=gltA PE=3 SV=1

Observed	Mr(expt)	Mr(calc)	ppm	Start - End	Miss	Ions	Peptide
790.5086	789.5013	789.4286	92.1	121 - 126	0	---	R.LFHAFR.R
808.4445	807.4373	807.4490	-14.59	420 - 426	1	---	R.DFKSALK.R
817.4935	816.4862	816.4065	97.6	301 - 307	0	---	R.LMGFGHR.V
1015.6326	1014.6253	1014.4804	143	321 - 328	0	---	R.ETCHEVLK.E
1186.7303	1185.7230	1185.6354	73.9	23 - 33	0	---	K.GTLGQDVIDIR.S
1219.6443	1218.6371	1218.4905	120	169 - 178	0	---	K.MPTMAAMCYK.Y + Oxidation (M)
1265.7392	1264.7319	1264.6346	76.9	111 - 120	0	---	R.HTMIHEQITR.L
1326.7883	1325.7810	1325.6768	78.6	179 - 189	0	18	K.YSIGQPFVYPR.N
1598.9839	1597.9766	1597.8464	81.5	57 - 70	0	---	K.ITFIDGDEGILLHR.G
1631.9464	1630.9392	1630.9029	22.2	8 - 22	0	---	K.ITLTGDTTIELDVLK.G
1658.9825	1657.9752	1657.8999	45.4	23 - 38	1	---	K.GTLGQDVIDIRSLGSK.G
1824.0974	1823.0901	1822.9472	78.4	106 - 120	1	---	R.TTVTRHTMIHEQITR.L
1938.1476	1937.1403	1936.8513	149	39 - 56	0	---	K.GVFTFDPGFTSTASCESK.I
2005.2465	2004.2393	2004.0388	100	223 - 240	0	---	R.ILILHADHEQNASTSTVR.T

2717.3922	2716.3849	2716.3305	20.0	334 - 356	0	---	K.DDLLEVAMELEHIALNDPYFIEK.K

No match to: 804.3324, 832.4057, 837.5595, 842.5625, 857.5055, 930.5245, 964.5936, 982.6070, 986.6133, 1006.6445, 1027.6664, 1042.6300, 1078.6445, 1107.8344, 1142.6742, 1153.6546, 1165.6966, 1179.7073, 1197.6582, 1214.7097, 1233.7159, 1240.6944, 1269.7243, 1368.8298, 1380.8667, 1428.9212, 1450.8531, 1475.8727, 1477.8489, 1484.8980, 1530.8700, 1557.9283, 1575.0154, 1617.9280, 1654.9735, 1708.0185, 1712.9684, 1724.0543, 1729.0035, 1733.0874, 1756.0868, 1781.0936, 1797.1145, 1804.0575, 1813.1035, 1839.0932, 1850.0717, 1882.1314, 1900.1334, 1915.1486, 1931.1352, 1947.1417, 1949.1320, 1952.1522, 1966.1980, 1981.1803, 1994.1847, 2021.1854, 2042.2539, 2044.2373, 2064.3065, 2117.3601, 2149.2508, 2181.2406, 2211.3162, 2215.2391, 2229.3298, 2271.3649, 2284.3753, 2384.2549, 2399.3191, 2438.4857, 2450.5041, 2498.5023, 2615.5335, 2645.5873, 2681.5950, 2705.4362, 2729.6061, 2745.6012, 2808.6023, 2856.6076, 2872.6380, 3048.6449, 3284.6878

4. EFTU_AERHH **Mass:** 43525 **Score:** 94 **Expect:** 0.00015 **Matches:** 7
Elongation factor Tu OS=Aeromonas hydrophila subsp. hydrophila (strain ATCC 7966 / DSM 30187 / JCM 1027 / KCTC 2358 / NCIMB 9240

Observed	Mr(expt)	Mr(calc)	ppm	Start - End	Miss	Ions	Peptide
837.5595	836.5522	836.4868	78.2	118 - 124	0	---	R.EHILLGR.Q
1233.7159	1232.7086	1232.6091	80.8	326 - 334	0	10	K.GYRPQFYFR.T
1575.0154	1574.0081	1573.9304	49.4	290 - 304	0	---	R.GQVLAKPGTIKPHTK.F
1729.0035	1727.9962	1727.8366	92.4	305 - 319	1	---	K.FESEVYVLSKEEGGR.H
1966.1980	1965.1907	1965.0207	86.5	189 - 205	0	64	K.ILELAGHLDTYIPEPER.A
2729.6061	2728.5988	2728.3346	96.8	91 - 117	0	---	K.NMITGAAQMDGAILVVAATDGPMPQTR.E
2745.6012	2744.5939	2744.3296	96.3	91 - 117	0	---	K.NMITGAAQMDGAILVVAATDGPMPQTR.E + Oxidation (M)

No match to: 790.5086, 804.3324, 808.4445, 817.4935, 832.4057, 842.5625, 857.5055, 930.5245, 964.5936, 982.6070, 986.6133, 1006.6445, 1015.6326, 1027.6664, 1042.6300, 1078.6445, 1107.8344, 1142.6742, 1153.6546, 1165.6966, 1179.7073, 1186.7303, 1197.6582, 1214.7097, 1219.6443, 1240.6944, 1265.7392, 1269.7243, 1326.7883, 1368.8298, 1380.8667, 1428.9212, 1450.8531, 1475.8727, 1477.8489, 1484.8980, 1530.8700, 1557.9283, 1598.9839, 1617.9280, 1631.9464, 1654.9735, 1658.9825, 1708.0185, 1712.9684, 1724.0543, 1733.0874, 1756.0868, 1781.0936, 1797.1145, 1804.0575, 1813.1035, 1824.0974, 1839.0932, 1850.0717, 1882.1314, 1900.1334, 1915.1486, 1931.1352, 1938.1476, 1947.1417, 1949.1320, 1952.1522, 1981.1803, 1994.1847, 2005.2465, 2021.1854, 2042.2539, 2044.2373, 2064.3065, 2117.3601, 2149.2508, 2181.2406, 2211.3162, 2215.2391, 2229.3298, 2271.3649, 2284.3753, 2384.2549, 2399.3191, 2438.4857, 2450.5041, 2498.5023, 2615.5335, 2645.5873, 2681.5950, 2705.4362, 2717.3922, 2808.6023, 2856.6076, 2872.6380, 3048.6449, 3284.6878

5. EFTU_COXBN **Mass:** 43613 **Score:** 88 **Expect:** 0.00058 **Matches:** 8
Elongation factor Tu OS=Coxiella burnetii (strain Dugway 5J108-111) OX=434922 GN=tuf1 PE=3 SV=1

Observed	Mr(expt)	Mr(calc)	ppm	Start - End	Miss	Ions	Peptide
817.4935	816.4862	816.4090	94.6	286 - 291	1	---	K.REEVER.G
1186.7303	1185.7230	1185.6969	22.0	386 - 397	1	---	R.TVGAGVVTKIIE.-
1326.7883	1325.7810	1325.7231	43.7	306 - 316	1	---	K.KFEAEIYVLSK.E
1475.8727	1474.8655	1474.8606	3.26	26 - 39	1	5	K.TTLTAALTKVLSEK.Y
1631.9464	1630.9392	1630.7699	104	46 - 59	1	63	K.AFDQIDNAPEERAR.G
1712.9684	1711.9611	1711.9178	25.3	362 - 377	0	---	K.VTVELIAPVAMDEGLR.F
1729.0035	1727.9962	1727.9128	48.3	362 - 377	0	---	K.VTVELIAPVAMDEGLR.F + Oxidation (M)
1824.0974	1823.0901	1822.9438	80.2	9 - 25	0	---	R.EKPHVNVGTIGHVDHGK.T

No match to: 790.5086, 804.3324, 808.4445, 832.4057, 837.5595, 842.5625, 857.5055, 930.5245, 964.5936, 982.6070, 986.6133, 1006.6445, 1015.6326, 1027.6664, 1042.6300, 1078.6445, 1107.8344, 1142.6742, 1153.6546, 1165.6966, 1179.7073, 1197.6582, 1214.7097, 1219.6443, 1233.7159, 1240.6944, 1265.7392, 1269.7243, 1368.8298, 1380.8667, 1428.9212, 1450.8531, 1477.8489, 1484.8980, 1530.8700, 1557.9283, 1575.0154, 1598.9839, 1617.9280, 1654.9735, 1658.9825, 1708.0185, 1724.0543, 1733.0874, 1756.0868, 1781.0936, 1797.1145, 1804.0575, 1813.1035, 1839.0932, 1850.0717, 1882.1314, 1900.1334, 1915.1486, 1931.1352, 1938.1476, 1947.1417, 1949.1320, 1952.1522, 1966.1980, 1981.1803, 1994.1847, 2005.2465, 2021.1854, 2042.2539, 2044.2373, 2064.3065, 2117.3601, 2149.2508, 2181.2406, 2211.3162, 2215.2391, 2229.3298, 2271.3649, 2284.3753, 2384.2549, 2399.3191, 2438.4857, 2450.5041, 2498.5023, 2615.5335, 2645.5873, 2681.5950, 2705.4362, 2717.3922, 2729.6061, 2745.6012, 2808.6023, 2856.6076, 2872.6380, 3048.6449, 3284.6878

6. EFTU_COXBR **Mass:** 43613 **Score:** 88 **Expect:** 0.00058 **Matches:** 8
Elongation factor Tu OS=Coxiella burnetii (strain RSA 331 / Henzerling II) OX=360115 GN=tuf1 PE=3 SV=1

Observed	Mr(expt)	Mr(calc)	ppm	Start - End	Miss	Ions	Peptide
817.4935	816.4862	816.4090	94.6	286 - 291	1	---	K.REEVER.G
1186.7303	1185.7230	1185.6969	22.0	386 - 397	1	---	R.TVGAGVVTKIIE.-
1326.7883	1325.7810	1325.7231	43.7	306 - 316	1	---	K.KFEAEIYVLSK.E
1475.8727	1474.8655	1474.8606	3.26	26 - 39	1	5	K.TTLTAALTKVLSEK.Y
1631.9464	1630.9392	1630.7699	104	46 - 59	1	63	K.AFDQIDNAPEERAR.G
1712.9684	1711.9611	1711.9178	25.3	362 - 377	0	---	K.VTVELIAPVAMDEGLR.F
1729.0035	1727.9962	1727.9128	48.3	362 - 377	0	---	K.VTVELIAPVAMDEGLR.F + Oxidation (M)
1824.0974	1823.0901	1822.9438	80.2	9 - 25	0	---	R.EKPHVNVGTIGHVDHGK.T

No match to: 790.5086, 804.3324, 808.4445, 832.4057, 837.5595, 842.5625, 857.5055, 930.5245, 964.5936, 982.6070, 986.6133, 1006.6445, 1015.6326, 1027.6664, 1042.6300, 1078.6445, 1107.8344, 1142.6742, 1153.6546, 1165.6966, 1179.7073, 1197.6582, 1214.7097, 1219.6443, 1233.7159, 1240.6944, 1265.7392, 1269.7243, 1368.8298, 1380.8667, 1428.9212, 1450.8531, 1477.8489, 1484.8980, 1530.8700, 1557.9283, 1575.0154, 1598.9839, 1617.9280, 1654.9735, 1658.9825, 1708.0185, 1724.0543, 1733.0874, 1756.0868, 1781.0936, 1797.1145, 1804.0575, 1813.1035, 1839.0932, 1850.0717, 1882.1314, 1900.1334, 1915.1486, 1931.1352, 1938.1476, 1947.1417, 1949.1320, 1952.1522, 1966.1980, 1981.1803, 1994.1847, 2005.2465, 2021.1854, 2042.2539, 2044.2373, 2064.3065, 2117.3601, 2149.2508, 2181.2406, 2211.3162, 2215.2391, 2229.3298, 2271.3649, 2284.3753, 2384.2549, 2399.3191, 2438.4857, 2450.5041, 2498.5023, 2615.5335, 2645.5873, 2681.5950, 2705.4362, 2717.3922, 2729.6061, 2745.6012, 2808.6023, 2856.6076, 2872.6380, 3048.6449, 3284.6878

7. EFTU_COXBU **Mass:** 43613 **Score:** 88 **Expect:** 0.00058 **Matches:** 8
Elongation factor Tu OS=Coxiella burnetii (strain RSA 493 / Nine Mile phase I) OX=227377 GN=tufA PE=1 SV=1

Observed	Mr(expt)	Mr(calc)	ppm	Start - End	Miss	Ions	Peptide
817.4935	816.4862	816.4090	94.6	286 - 291	1	---	K.REEVER.G
1186.7303	1185.7230	1185.6969	22.0	386 - 397	1	---	R.TVGAGVVTKIIE.-
1326.7883	1325.7810	1325.7231	43.7	306 - 316	1	---	K.KFEAEIYVLSK.E
1475.8727	1474.8655	1474.8606	3.26	26 - 39	1	5	K.TTLTAALTKVLSEK.Y
1631.9464	1630.9392	1630.7699	104	46 - 59	1	63	K.AFDQIDNAPEERAR.G
1712.9684	1711.9611	1711.9178	25.3	362 - 377	0	---	K.VTVELIAPVAMDEGLR.F
1729.0035	1727.9962	1727.9128	48.3	362 - 377	0	---	K.VTVELIAPVAMDEGLR.F + Oxidation (M)
1824.0974	1823.0901	1822.9438	80.2	9 - 25	0	---	R.EKPHVNVGTIGHVDHGK.T

No match to: 790.5086, 804.3324, 808.4445, 832.4057, 837.5595, 842.5625, 857.5055, 930.5245, 964.5936, 982.6070, 986.6133, 1006.6445, 1015.6326, 1027.6664, 1042.6300, 1078.6445, 1107.8344, 1142.6742, 1153.6546, 1165.6966, 1179.7073, 1197.6582, 1214.7097, 1219.6443, 1233.7159, 1240.6944, 1265.7392, 1269.7243, 1368.8298, 1380.8667, 1428.9212, 1450.8531, 1477.8489, 1484.8980, 1530.8700, 1557.9283, 1575.0154, 1598.9839, 1617.9280, 1654.9735, 1658.9825, 1708.0185, 1724.0543, 1733.0874, 1756.0868, 1781.0936, 1797.1145, 1804.0575, 1813.1035, 1839.0932, 1850.0717, 1882.1314, 1900.1334, 1915.1486, 1931.1352, 1938.1476, 1947.1417, 1949.1320, 1952.1522, 1966.1980, 1981.1803, 1994.1847, 2005.2465, 2021.1854, 2042.2539, 2044.2373, 2064.3065, 2117.3601, 2149.2508, 2181.2406, 2211.3162, 2215.2391, 2229.3298, 2271.3649, 2284.3753, 2384.2549, 2399.3191, 2438.4857, 2450.5041, 2498.5023, 2615.5335, 2645.5873, 2681.5950, 2705.4362, 2717.3922, 2729.6061, 2745.6012, 2808.6023, 2856.6076, 2872.6380, 3048.6449, 3284.6878

8. EFTU_MARHV **Mass:** 44034 **Score:** 87 **Expect:** 0.00071 **Matches:** 5
Elongation factor Tu OS=Marinobacter hydrocarbonoclasticus (strain ATCC 700491 / DSM 11845 / VT8) OX=351348 GN=tuf PE=3 SV=1

Observed	Mr(expt)	Mr(calc)	ppm	Start - End	Miss	Ions	Peptide

1027.6664	1026.6591	1026.5822	74.9	275	284	0	---	R.AGENVGVLLR.G
1233.7159	1232.7086	1232.6091	80.8	330	338	0	10	K.GYRPQFYFR.T
1631.9464	1630.9392	1630.7699	104	47	60	1	63	R.AFDQIDNAPEERAR.G
1658.9825	1657.9752	1657.8199	93.7	196	209	0	---	K.LVEALDDYIPEPER.A
2808.6023	2807.5950	2807.2711	115	92	118	0	---	K.NMITGAAQMDGAILVCSAADGPMPQTR.E + 2 Oxidation (M)

No match to: 790.5086, 804.3324, 808.4445, 817.4935, 832.4057, 837.5595, 842.5625, 857.5055, 930.5245, 964.5936, 982.6070, 986.6133, 1006.6445, 1015.6326, 1042.6300, 1078.6445, 1107.8344, 1142.6742, 1153.6546, 1165.6966, 1179.7073, 1186.7303, 1197.6582, 1214.7097, 1219.6443, 1240.6944, 1265.7392, 1269.7243, 1326.7883, 1368.8298, 1380.8667, 1428.9212, 1450.8531, 1475.8727, 1477.8489, 1484.8980, 1530.8700, 1557.9283, 1575.0154, 1598.9839, 1617.9280, 1654.9735, 1708.0185, 1712.9684, 1724.0543, 1729.0035, 1733.0874, 1756.0868, 1781.0936, 1797.1145, 1804.0575, 1813.1035, 1824.0974, 1839.0932, 1850.0717, 1882.1314, 1900.1334, 1915.1486, 1931.1352, 1938.1476, 1947.1417, 1949.1320, 1952.1522, 1966.1980, 1981.1803, 1994.1847, 2005.2465, 2021.1854, 2042.2539, 2044.2373, 2064.3065, 2117.3601, 2149.2508, 2181.2406, 2211.3162, 2215.2391, 2229.3298, 2271.3649, 2284.3753, 2384.2549, 2399.3191, 2438.4857, 2450.5041, 2498.5023, 2615.5335, 2645.5873, 2681.5950, 2705.4362, 2717.3922, 2729.6061, 2745.6012, 2856.6076, 2872.6380, 3048.6449, 3284.6878

9. DNAK_BRADU **Mass:** 68364 **Score:** 76 **Expect:** 0.0076 **Matches:** 7

Chaperone protein DnaK OS=Bradyrhizobium diazoefficiens (strain JCM 10833 / IAM 13628 / NBRC 14792 / USDA 110) OX=224911 GN=dnaK

Observed	Mr(expt)	Mr(calc)	ppm	Start	End	Miss	Ions	Peptide
1015.6326	1014.6253	1014.5644	60.0	290	297	1	---	K.HLTMKLTR.A + Oxidation (M)
1078.6445	1077.6372	1077.5488	82.0	583	592	0	---	K.TQTLAQASMK.L
1186.7303	1185.7230	1185.6102	95.2	242	251	1	---	K.EQGINLRNDK.L
1658.9825	1657.9752	1657.8320	86.4	448	462	0	---	K.MLGQFDLMGIPPAPR.G + Oxidation (M)
1966.1980	1965.1907	1964.9547	120	4	22	0	36	K.VIGIDLGTTNSCVAVMDGK.N + Oxidation (M)
2229.3298	2228.3225	2228.1835	62.4	318	340	1	25	K.ALKDAGVTAGEIGEVVLVGGMSR.M
2450.5041	2449.4968	2449.1440	144	441	462	1	---	R.EMAADNKMLGQFDLMGIPPAPR.G + 3 Oxidation (M)

No match to: 790.5086, 804.3324, 808.4445, 817.4935, 832.4057, 837.5595, 842.5625, 857.5055, 930.5245, 964.5936, 982.6070, 986.6133, 1006.6445, 1027.6664, 1042.6300, 1107.8344, 1142.6742, 1153.6546, 1165.6966, 1179.7073, 1197.6582, 1214.7097, 1219.6443, 1233.7159, 1240.6944, 1265.7392, 1269.7243, 1326.7883, 1368.8298, 1380.8667, 1428.9212, 1450.8531, 1475.8727, 1477.8489, 1484.8980, 1530.8700, 1557.9283, 1575.0154, 1598.9839, 1617.9280, 1631.9464, 1654.9735, 1708.0185, 1712.9684, 1724.0543, 1729.0035, 1733.0874, 1756.0868, 1781.0936, 1797.1145, 1804.0575, 1813.1035, 1824.0974, 1839.0932, 1850.0717, 1882.1314, 1900.1334, 1915.1486, 1931.1352, 1938.1476, 1947.1417, 1949.1320, 1952.1522, 1981.1803, 1994.1847, 2005.2465, 2021.1854, 2042.2539, 2044.2373, 2064.3065, 2117.3601, 2149.2508, 2181.2406, 2211.3162, 2215.2391, 2271.3649, 2284.3753, 2384.2549, 2399.3191, 2438.4857, 2498.5023, 2615.5335, 2645.5873, 2681.5950, 2705.4362, 2717.3922, 2729.6061, 2745.6012, 2808.6023, 2856.6076, 2872.6380, 3048.6449, 3284.6878

10. ADH_THEBR **Mass:** 37851 **Score:** 75 **Expect:** 0.0096 **Matches:** 7

NADP-dependent isopropanol dehydrogenase OS=Thermoanaerobacter brockii OX=29323 GN=adh PE=1 SV=1

Observed	Mr(expt)	Mr(calc)	ppm	Start	End	Miss	Ions	Peptide
1042.6300	1041.6227	1041.5502	69.7	292	301	1	---	K.GGLCPGGRLR.M
1214.7097	1213.7024	1213.6199	68.0	1	11	1	---	-.MKGFAMLSIGK.V + 2 Oxidation (M)
1240.6944	1239.6871	1239.6355	41.6	333	342	1	---	K.AFMLMKDKPK.D + 2 Oxidation (M)
1530.8700	1529.8628	1529.7483	74.8	279	291	1	---	R.LEWGCGMAHKTIK.G
1631.9464	1630.9392	1630.7872	93.2	220	234	0	48	K.DGPIESQIMNLTEGK.G
2229.3298	2228.3225	2228.0545	120	92	111	1	---	R.TSEVQRGYHQHSGGMLAGWK.F
3048.6449	3047.6376	3047.5135	40.7	53	80	1	---	R.HNMILGHEAVGEVVEVGSEVKDFKPGDR.V

No match to: 790.5086, 804.3324, 808.4445, 817.4935, 832.4057, 837.5595, 842.5625, 857.5055, 930.5245, 964.5936, 982.6070, 986.6133, 1006.6445, 1015.6326, 1027.6664, 1078.6445, 1107.8344, 1142.6742, 1153.6546, 1165.6966, 1179.7073, 1186.7303, 1197.6582, 1219.6443, 1233.7159, 1265.7392, 1269.7243, 1326.7883, 1368.8298, 1380.8667, 1428.9212, 1450.8531, 1475.8727, 1477.8489, 1484.8980, 1557.9283, 1575.0154, 1598.9839, 1617.9280, 1654.9735, 1658.9825, 1708.0185, 1712.9684, 1724.0543, 1729.0035, 1733.0874, 1756.0868, 1781.0936, 1797.1145, 1804.0575, 1813.1035, 1824.0974, 1839.0932, 1850.0717, 1882.1314, 1900.1334, 1915.1486, 1931.1352, 1938.1476, 1947.1417, 1949.1320, 1952.1522, 1966.1980, 1981.1803, 1994.1847, 2005.2465, 2021.1854, 2042.2539, 2044.2373, 2064.3065, 2117.3601, 2149.2508, 2181.2406, 2211.3162, 2215.2391, 2271.3649, 2284.3753, 2384.2549, 2399.3191, 2438.4857, 2450.5041, 2498.5023, 2615.5335, 2645.5873, 2681.5950, 2705.4362, 2717.3922, 2729.6061, 2745.6012, 2808.6023, 2856.6076, 2872.6380, 3284.6878

11. TOP1_STAS1 **Mass:** 79764 **Score:** 75 **Expect:** 0.01 **Matches:** 12

DNA topoisomerase 1 OS=Staphylococcus saprophyticus subsp. saprophyticus (strain ATCC 15305 / DSM 20229) OX=342451 GN=topA PE=3

Observed	Mr(expt)	Mr(calc)	ppm	Start	End	Miss	Ions	Peptide
1006.6445	1005.6372	1005.5607	76.1	401	409	1	---	R.ANGQTIKFK.G
1186.7303	1185.7230	1185.6003	104	358	366	1	---	K.NFLTRDQHR.L
1240.6944	1239.6871	1239.5924	76.4	184	192	0	---	R.NFKPEEYWK.I
1269.7243	1268.7171	1268.6976	15.4	2	13	0	---	M.AENLVIVESPAK.A
1368.8298	1367.8225	1367.8249	-1.72	167	178	1	---	R.VQSVALRLVIDR.E
1450.8531	1449.8459	1449.7874	40.3	27	39	1	---	K.VIASMGHVRDLPR.S
1475.8727	1474.8655	1474.7086	106	127	139	0	26	R.GIEMELVDAQQAR.R + Oxidation (M)
1631.9464	1630.9392	1630.8097	79.4	127	140	1	29	R.GIEMELVDAQQARR.I + Oxidation (M)
1850.0717	1849.0644	1848.9264	74.6	124	139	1	---	K.HPRGIEMELVDAQQAR.R
1882.1314	1881.1241	1880.9740	79.8	274	289	1	---	K.TMMLAQQLYEGIDLKK.Q
1938.1476	1937.1403	1936.9351	106	334	351	0	---	K.GQGDQDAHEAIRPSSTLR.T
2229.3298	2228.3225	2228.0419	126	40	58	1	---	R.SQMGVDAENDYEPKYITIR.G

No match to: 790.5086, 804.3324, 808.4445, 817.4935, 832.4057, 837.5595, 842.5625, 857.5055, 930.5245, 964.5936, 982.6070, 986.6133, 1015.6326, 1027.6664, 1042.6300, 1078.6445, 1107.8344, 1142.6742, 1153.6546, 1165.6966, 1179.7073, 1197.6582, 1214.7097, 1219.6443, 1233.7159, 1265.7392, 1326.7883, 1380.8667, 1428.9212, 1477.8489, 1484.8980, 1530.8700, 1557.9283, 1575.0154, 1598.9839, 1617.9280, 1654.9735, 1658.9825, 1708.0185, 1712.9684, 1724.0543, 1729.0035, 1733.0874, 1756.0868, 1781.0936, 1797.1145, 1804.0575, 1813.1035, 1824.0974, 1839.0932, 1900.1334, 1915.1486, 1931.1352, 1947.1417, 1949.1320, 1952.1522, 1966.1980, 1981.1803, 1994.1847, 2005.2465, 2021.1854, 2042.2539, 2044.2373, 2064.3065, 2117.3601, 2149.2508, 2181.2406, 2211.3162, 2215.2391, 2271.3649, 2284.3753, 2384.2549, 2399.3191, 2438.4857, 2450.5041, 2498.5023, 2615.5335, 2645.5873, 2681.5950, 2705.4362, 2717.3922, 2729.6061, 2745.6012, 2808.6023, 2856.6076, 2872.6380, 3048.6449, 3284.6878

12. HSCA_AROAE **Mass:** 66854 **Score:** 74 **Expect:** 0.014 **Matches:** 12

Chaperone protein HscA homolog OS=Aromatoleum aromaticum (strain EbN1) OX=76114 GN=hscA PE=3 SV=1

Observed	Mr(expt)	Mr(calc)	ppm	Start	End	Miss	Ions	Peptide
808.4445	807.4373	807.4425	-6.52	89	95	1	---	R.FMGRGLK.D
832.4057	831.3984	831.4450	-56.00	165	172	1	---	R.QATKDAAK.L
857.5055	856.4982	856.5130	-17.28	587	594	1	---	K.KGIEALAR.A
964.5936	963.5863	963.5171	71.8	81	88	1	---	K.NTIMSVKR.F + Oxidation (M)
1240.6944	1239.6871	1239.7299	-34.52	169	180	1	---	K.DAAKLAGLNVLR.L
1368.8298	1367.8225	1367.7409	59.7	321	333	1	---	K.VLRDAGLGPEDVK.G
1617.9280	1616.9207	1616.8093	68.9	51	64	1	---	R.SMLPSIVRYHADGR.I + Oxidation (M)
1824.0974	1823.0901	1822.9577	72.6	416	431	1	---	R.NSTLPIARAQEFTTFK.D
2149.2508	2148.2435	2148.0092	109	432	450	1	---	K.DGQTAMAFHVVQGEREMVK.D + Oxidation (M)
2211.3162	2210.3090	2209.9950	142	96	115	0	---	K.DVSHVESTPYDFIDAGGMVR.L + Oxidation (M)
2229.3298	2228.3225	2228.1405	81.7	38	58	1	33	R.NGIAVCLADEAGRSMLPSIVR.Y
2399.3191	2398.3118	2398.2162	39.9	65	87	1	---	R.IEVGQTAAAAHTTDPKNTIMSVK.R + Oxidation (M)

No match to: 790.5086, 804.3324, 817.4935, 837.5595, 842.5625, 930.5245, 982.6070, 986.6133, 1006.6445, 1015.6326, 1027.6664, 1042.6300, 1078.6445, 1107.8344, 1142.6742, 1153.6546, 1165.6966, 1179.7073, 1186.7303, 1197.6582, 1214.7097, 1219.6443, 1233.7159, 1265.7392, 1269.7243, 1326.7883, 1380.8667, 1428.9212, 1450.8531, 1475.8727, 1477.8489, 1484.8980, 1530.8700, 1557.9283, 1575.0154, 1598.9839, 1631.9464, 1654.9735, 1658.9825, 1708.0185, 1712.9684, 1724.0543, 1729.0035, 1733.0874, 1756.0868, 1781.0936, 1797.1145, 1804.0575, 1813.1035, 1839.0932, 1850.0717, 1882.1314, 1900.1334, 1915.1486, 1931.1352, 1938.1476, 1947.1417, 1949.1320, 1952.1522, 1966.1980, 1981.1803, 1994.1847, 2005.2465, 2021.1854, 2042.2539, 2044.2373, 2064.3065, 2117.3601, 2181.2406, 2215.2391, 2271.3649, 2284.3753, 2384.2549, 2438.4857, 2450.5041, 2498.5023, 2615.5335, 2645.5873, 2681.5950, 2705.4362, 2717.3922, 2729.6061, 2745.6012, 2808.6023, 2856.6076, 2872.6380, 3048.6449, 3284.6878

13. LEU1_BUCUN **Mass:** 56434 **Score:** 72 **Expect:** 0.022 **Matches:** 6

2-isopropylmalate synthase (Fragment) OS=Buchnera aphidicola subsp. Uroleucon sonchi OX=118118 GN=leuA PE=3 SV=1

Observed	Mr(expt)	Mr(calc)	ppm	Start	End	Miss	Ions	Peptide
790.5086	789.5013	789.4345	84.7	256	262	0	---	R.TSQVISR.I
930.5245	929.5172	929.5658	-52.29	311	318	1	---	K.VKLNLTSR.S
1475.8727	1474.8655	1474.7701	64.7	223	236	0	39	R.AGNTALEEVIMAIK.V + Oxidation (M)
1724.0543	1723.0470	1722.8498	114	296	310	1	---	R.ENYEIMDPSSIGLKK.V
1882.1314	1881.1241	1880.8938	122	294	309	1	---	K.NRENYEIMDPSSIGLK.K + Oxidation (M)
1966.1980	1965.1907	1964.9554	120	25	43	0	19	K.AGVDIMEVGFPISSPGDFK.S

No match to: 804.3324, 808.4445, 817.4935, 832.4057, 837.5595, 842.5625, 857.5055, 964.5936, 982.6070, 986.6133, 1006.6445, 1015.6326, 1027.6664, 1042.6300, 1078.6445, 1107.8344, 1142.6742, 1153.6546, 1165.6966, 1179.7073, 1186.7303, 1197.6582, 1214.7097, 1219.6443, 1233.7159, 1240.6944, 1265.7392, 1269.7243, 1326.7883, 1368.8298, 1380.8667, 1428.9212, 1450.8531, 1477.8489, 1484.8980, 1530.8700, 1557.9283, 1575.0154, 1598.9839, 1617.9280, 1631.9464, 1654.9735, 1658.9825, 1708.0185, 1712.9684, 1729.0035, 1733.0874, 1756.0868, 1781.0936, 1797.1145, 1804.0575, 1813.1035, 1824.0974, 1839.0932, 1850.0717, 1900.1334, 1915.1486, 1931.1352, 1938.1476, 1947.1417, 1949.1320, 1952.1522, 1981.1803, 1994.1847, 2005.2465, 2021.1854, 2042.2539, 2044.2373, 2064.3065, 2117.3601, 2149.2508, 2181.2406, 2211.3162, 2215.2391, 2229.3298, 2271.3649, 2284.3753, 2384.2549, 2399.3191, 2438.4857, 2450.5041, 2498.5023, 2615.5335, 2645.5873, 2681.5950, 2705.4362, 2717.3922, 2729.6061, 2745.6012, 2808.6023, 2856.6076, 2872.6380, 3048.6449, 3284.6878

14. SECA_PROM4 **Mass:** 107505 **Score:** 71 **Expect:** 0.025 **Matches:** 12

Protein translocase subunit SecA OS=Prochlorococcus marinus (strain MIT 9211) OX=93059 GN=secA PE=3 SV=1

Observed	Mr(expt)	Mr(calc)	ppm	Start	End	Miss	Ions	Peptide
808.4445	807.4373	807.4239	16.6	41	47	1	---	R.AKTADFR.E
857.5055	856.4982	856.5018	-4.18	589	595	0	---	K.VLIDLER.K
1428.9212	1427.9139	1427.7197	136	299	310	0	---	K.DPWAHYITNALK.A
1598.9839	1597.9766	1597.8689	67.4	128	141	1	---	R.GVHVVTVNDYLARR.D
1631.9464	1630.9392	1630.8712	41.7	242	257	1	17	K.AAEVVASLIRAAEMGK.D + Oxidation (M)
1824.0974	1823.0901	1822.9247	90.7	507	523	1	---	R.GTDIILGGNSDYMARLK.L
1947.1417	1946.1345	1945.9680	85.5	325	342	1	---	R.NGEAVIVDEFTGRVMPGR.R
1966.1980	1965.1907	1965.1272	32.3	532	548	1	31	K.LVKPEDGHKPPVPLQRR.S
2042.2539	2041.2467	2041.1208	61.7	59	75	1	---	K.QRELLDEILPEVFSVVR.E
2149.2508	2148.2435	2147.9544	135	928	945	1	---	R.NVIYSMFMFQPAPESDKE.- + Oxidation (M)
2384.2549	2383.2476	2383.1664	34.1	86	106	0	---	R.HFDVQLIGGMVLHEGQIAEMK.T + 2 Oxidation (M)
2856.6076	2855.6003	2855.3946	72.0	86	111	1	---	R.HFDVQLIGGMVLHEGQIAEMKTGEGK.T + 2 Oxidation (M)

No match to: 790.5086, 804.3324, 817.4935, 832.4057, 837.5595, 842.5625, 930.5245, 964.5936, 982.6070, 986.6133, 1006.6445, 1015.6326, 1027.6664, 1042.6300, 1078.6445, 1107.8344, 1142.6742, 1153.6546, 1165.6966, 1179.7073, 1186.7303, 1197.6582, 1214.7097, 1219.6443, 1233.7159, 1240.6944, 1265.7392, 1269.7243, 1326.7883, 1368.8298, 1380.8667, 1450.8531, 1475.8727, 1477.8489, 1484.8980, 1530.8700, 1557.9283, 1575.0154, 1617.9280, 1654.9735, 1658.9825, 1708.0185, 1712.9684, 1724.0543, 1729.0035, 1733.0874, 1756.0868, 1781.0936, 1797.1145, 1804.0575, 1813.1035, 1839.0932, 1850.0717, 1882.1314, 1900.1334, 1915.1486, 1931.1352, 1938.1476, 1949.1320, 1952.1522, 1981.1803, 1994.1847, 2005.2465, 2021.1854, 2044.2373, 2064.3065, 2117.3601, 2181.2406, 2211.3162, 2215.2391, 2229.3298, 2271.3649, 2284.3753, 2399.3191, 2438.4857, 2450.5041, 2498.5023, 2615.5335, 2645.5873, 2681.5950, 2705.4362, 2717.3922, 2729.6061, 2745.6012, 2808.6023, 2872.6380, 3048.6449, 3284.6878

15. RPOA_PROM1 **Mass:** 34244 **Score:** 71 **Expect:** 0.026 **Matches:** 5

DNA-directed RNA polymerase subunit alpha OS=Prochlorococcus marinus (strain NATL1A) OX=167555 GN=rpoA PE=3 SV=1

Observed	Mr(expt)	Mr(calc)	ppm	Start	End	Miss	Ions	Peptide
804.3324	803.3251	803.4137	-110.29	84	90	0	---	K.QISVDSR.S
1197.6582	1196.6509	1196.7129	-51.78	99	110	1	---	R.LVVTGPADVKAK.D
1475.8727	1474.8655	1474.7814	57.0	43	57	0	28	R.VLMGGLEGSAVTAVR.I + Oxidation (M)
1631.9464	1630.9392	1630.8825	34.8	42	57	1	27	R.RVLMGGLEGSAVTAVR.I + Oxidation (M)
2117.3601	2116.3529	2116.0834	127	73	90	1	---	R.EDVLDILLNCKQISVDSR.S

No match to: 790.5086, 808.4445, 817.4935, 832.4057, 837.5595, 842.5625, 857.5055, 930.5245, 964.5936, 982.6070, 986.6133, 1006.6445, 1015.6326, 1027.6664, 1042.6300, 1078.6445, 1107.8344, 1142.6742, 1153.6546, 1165.6966, 1179.7073, 1186.7303, 1214.7097, 1219.6443, 1233.7159, 1240.6944, 1265.7392, 1269.7243, 1326.7883, 1368.8298, 1380.8667, 1428.9212, 1450.8531, 1477.8489, 1484.8980, 1530.8700, 1557.9283, 1575.0154, 1598.9839, 1617.9280, 1654.9735, 1658.9825, 1708.0185, 1712.9684, 1724.0543, 1729.0035, 1733.0874, 1756.0868, 1781.0936, 1797.1145, 1804.0575, 1813.1035, 1824.0974, 1839.0932, 1850.0717, 1882.1314, 1900.1334, 1915.1486, 1931.1352, 1938.1476, 1947.1417, 1949.1320, 1952.1522, 1966.1980, 1981.1803, 1994.1847, 2005.2465, 2021.1854, 2042.2539, 2044.2373, 2064.3065, 2149.2508, 2181.2406, 2211.3162, 2215.2391, 2229.3298, 2271.3649, 2284.3753, 2384.2549, 2399.3191, 2438.4857, 2450.5041, 2498.5023, 2615.5335, 2645.5873, 2681.5950, 2705.4362, 2717.3922, 2729.6061, 2745.6012, 2808.6023, 2856.6076, 2872.6380, 3048.6449, 3284.6878

16. RPOA_PROMT **Mass:** 34244 **Score:** 71 **Expect:** 0.026 **Matches:** 5

DNA-directed RNA polymerase subunit alpha OS=Prochlorococcus marinus (strain NATL2A) OX=59920 GN=rpoA PE=3 SV=1

Observed	Mr(expt)	Mr(calc)	ppm	Start	End	Miss	Ions	Peptide
804.3324	803.3251	803.4137	-110.29	84	90	0	---	K.QISVDSR.S
1197.6582	1196.6509	1196.7129	-51.78	99	110	1	---	R.LVVTGPADVKAK.D
1475.8727	1474.8655	1474.7814	57.0	43	57	0	28	R.VLMGGLEGSAVTAVR.I + Oxidation (M)
1631.9464	1630.9392	1630.8825	34.8	42	57	1	27	R.RVLMGGLEGSAVTAVR.I + Oxidation (M)
2117.3601	2116.3529	2116.0834	127	73	90	1	---	R.EDVLDILLNCKQISVDSR.S

No match to: 790.5086, 808.4445, 817.4935, 832.4057, 837.5595, 842.5625, 857.5055, 930.5245, 964.5936, 982.6070, 986.6133, 1006.6445, 1015.6326, 1027.6664, 1042.6300, 1078.6445, 1107.8344, 1142.6742, 1153.6546, 1165.6966, 1179.7073, 1186.7303, 1214.7097, 1219.6443, 1233.7159, 1240.6944, 1265.7392, 1269.7243, 1326.7883, 1368.8298, 1380.8667, 1428.9212, 1450.8531, 1477.8489, 1484.8980, 1530.8700, 1557.9283, 1575.0154, 1598.9839, 1617.9280, 1654.9735, 1658.9825, 1708.0185, 1712.9684, 1724.0543, 1729.0035, 1733.0874, 1756.0868, 1781.0936, 1797.1145, 1804.0575, 1813.1035, 1824.0974, 1839.0932, 1850.0717, 1882.1314, 1900.1334, 1915.1486, 1931.1352, 1938.1476, 1947.1417, 1949.1320, 1952.1522, 1966.1980, 1981.1803, 1994.1847, 2005.2465, 2021.1854, 2042.2539, 2044.2373, 2064.3065, 2149.2508, 2181.2406, 2211.3162, 2215.2391, 2229.3298, 2271.3649, 2284.3753, 2384.2549, 2399.3191, 2438.4857, 2450.5041, 2498.5023, 2615.5335, 2645.5873, 2681.5950, 2705.4362, 2717.3922, 2729.6061, 2745.6012, 2808.6023, 2856.6076, 2872.6380, 3048.6449, 3284.6878

17. PROA_BRUSU **Mass:** 44300 **Score:** 69 **Expect:** 0.039 **Matches:** 9

Gamma-glutamyl phosphate reductase OS=Brucella suis biovar 1 (strain 1330) OX=204722 GN=proA PE=3 SV=1

Observed	Mr(expt)	Mr(calc)	ppm	Start	End	Miss	Ions	Peptide
1015.6326	1014.6253	1014.5345	89.5	80	88	0	---	R.IDAIAEDIR.A
1475.8727	1474.8655	1474.7450	81.7	258	271	0	21	R.TGICGAAETLLVDR.A
1631.9464	1630.9392	1630.8461	57.1	257	271	1	---	R.RTGICGAAETLLVDR.A
1756.0868	1755.0795	1754.9162	93.0	47	62	1	---	R.ADILEANRLDLANAEK.N
1813.1035	1812.0963	1811.9741	67.4	73	88	1	---	R.LTLNEARIDAIAEDIR.A

```
1938.1476  1937.1403  1936.9312    108    55 -  72   1  ---   R.LDLANAEKNGMAASFVDR.L + Oxidation (M)
1966.1980  1965.1907  1965.1081   42.0   189 - 208   1   12   R.AAVGEMLKGLGGAIDVIVPR.G
2181.2406  2180.2333  2180.1549   36.0    34 -  54   1  ---   K.ALNAAADAILEARADILEANR.L
2681.5950  2680.5877  2680.3795   77.7   217 - 239   1  ---   R.VQSEARVPVFAHLEGICHLYIDK.S
```

No match to: 790.5086, 804.3324, 808.4445, 817.4935, 832.4057, 837.5595, 842.5625, 857.5055, 930.5245, 964.5936, 982.6070, 986.6133, 1006.6445, 1027.6664, 1042.6300, 1078.6445, 1107.8344, 1142.6742, 1153.6546, 1165.6966, 1179.7073, 1186.7303, 1197.6582, 1214.7097, 1219.6443, 1233.7159, 1240.6944, 1265.7392, 1269.7243, 1326.7883, 1368.8298, 1380.8667, 1428.9212, 1450.8531, 1477.8489, 1484.8980, 1530.8700, 1557.9283, 1575.0154, 1598.9839, 1617.9280, 1654.9735, 1658.9825, 1708.0185, 1712.9684, 1724.0543, 1729.0035, 1733.0874, 1781.0936, 1797.1145, 1804.0575, 1824.0974, 1839.0932, 1850.0717, 1882.1314, 1900.1334, 1915.1486, 1931.1352, 1947.1417, 1949.1320, 1952.1522, 1981.1803, 1994.1847, 2005.2465, 2021.1854, 2042.2539, 2044.2373, 2064.3065, 2117.3601, 2149.2508, 2211.3162, 2215.2391, 2229.3298, 2271.3649, 2284.3753, 2384.2549, 2399.3191, 2438.4857, 2450.5041, 2498.5023, 2615.5335, 2645.5873, 2705.4362, 2717.3922, 2729.6061, 2745.6012, 2808.6023, 2856.6076, 2872.6380, 3048.6449, 3284.6878

18. EFTU_HERAU **Mass:** 43768 **Score:** 69 **Expect:** 0.04 **Matches:** 3

Elongation factor Tu OS=Herpetosiphon aurantiacus OX=65 GN=tuf PE=3 SV=1

Observed	Mr(expt)	Mr(calc)	ppm	Start	End	Miss	Ions	Peptide
804.3324	803.3251	803.4436	-147.44	35 -	41	1	---	K.TMALRGR.A
1631.9464	1630.9392	1630.7699	104	46 -	59	1	63	R.AFDQIDNAPEERAR.G
1947.1417	1946.1345	1946.0513	42.7	156 -	172	1	---	R.ELLTKYGFPGDEIPIVR.G

No match to: 790.5086, 808.4445, 817.4935, 832.4057, 837.5595, 842.5625, 857.5055, 930.5245, 964.5936, 982.6070, 986.6133, 1006.6445, 1015.6326, 1027.6664, 1042.6300, 1078.6445, 1107.8344, 1142.6742, 1153.6546, 1165.6966, 1179.7073, 1186.7303, 1197.6582, 1214.7097, 1219.6443, 1233.7159, 1240.6944, 1265.7392, 1269.7243, 1326.7883, 1368.8298, 1380.8667, 1428.9212, 1450.8531, 1475.8727, 1477.8489, 1484.8980, 1530.8700, 1557.9283, 1575.0154, 1598.9839, 1617.9280, 1654.9735, 1658.9825, 1708.0185, 1712.9684, 1724.0543, 1729.0035, 1733.0874, 1756.0868, 1781.0936, 1797.1145, 1804.0575, 1813.1035, 1824.0974, 1839.0932, 1850.0717, 1882.1314, 1900.1334, 1915.1486, 1931.1352, 1938.1476, 1949.1320, 1952.1522, 1966.1980, 1981.1803, 1994.1847, 2005.2465, 2021.1854, 2042.2539, 2044.2373, 2064.3065, 2117.3601, 2149.2508, 2181.2406, 2211.3162, 2215.2391, 2229.3298, 2271.3649, 2284.3753, 2384.2549, 2399.3191, 2438.4857, 2450.5041, 2498.5023, 2615.5335, 2645.5873, 2681.5950, 2705.4362, 2717.3922, 2729.6061, 2745.6012, 2808.6023, 2856.6076, 2872.6380, 3048.6449, 3284.6878

19. EFTU1_HALHL **Mass:** 43283 **Score:** 69 **Expect:** 0.04 **Matches:** 3

Elongation factor Tu 1 OS=Halorhodospira halophila (strain DSM 244 / SL1) OX=349124 GN=tuf1 PE=3 SV=1

Observed	Mr(expt)	Mr(calc)	ppm	Start	End	Miss	Ions	Peptide
817.4935	816.4862	816.4705	19.2	385 -	393	0	---	R.TVGAGVVSK.I
1631.9464	1630.9392	1630.7699	104	46 -	59	1	63	R.AFDQIDNAPEERAR.G
1654.9735	1653.9662	1653.8686	59.0	267 -	282	0	---	K.LLDQGEAGDNIGALLR.G

No match to: 790.5086, 804.3324, 808.4445, 832.4057, 837.5595, 842.5625, 857.5055, 930.5245, 964.5936, 982.6070, 986.6133, 1006.6445, 1015.6326, 1027.6664, 1042.6300, 1078.6445, 1107.8344, 1142.6742, 1153.6546, 1165.6966, 1179.7073, 1186.7303, 1197.6582, 1214.7097, 1219.6443, 1233.7159, 1240.6944, 1265.7392, 1269.7243, 1326.7883, 1368.8298, 1380.8667, 1428.9212, 1450.8531, 1475.8727, 1477.8489, 1484.8980, 1530.8700, 1557.9283, 1575.0154, 1598.9839, 1617.9280, 1658.9825, 1708.0185, 1712.9684, 1724.0543, 1729.0035, 1733.0874, 1756.0868, 1781.0936, 1797.1145, 1804.0575, 1813.1035, 1824.0974, 1839.0932, 1850.0717, 1882.1314, 1900.1334, 1915.1486, 1931.1352, 1938.1476, 1947.1417, 1949.1320, 1952.1522, 1966.1980, 1981.1803, 1994.1847, 2005.2465, 2021.1854, 2042.2539, 2044.2373, 2064.3065, 2117.3601, 2149.2508, 2181.2406, 2211.3162, 2215.2391, 2229.3298, 2271.3649, 2284.3753, 2384.2549, 2399.3191, 2438.4857, 2450.5041, 2498.5023, 2615.5335, 2645.5873, 2681.5950, 2705.4362, 2717.3922, 2729.6061, 2745.6012, 2808.6023, 2856.6076, 2872.6380, 3048.6449, 3284.6878

20. EFTU2_HALHL **Mass:** 43269 **Score:** 69 **Expect:** 0.04 **Matches:** 3

Elongation factor Tu 2 OS=Halorhodospira halophila (strain DSM 244 / SL1) OX=349124 GN=tuf2 PE=3 SV=1

Observed	Mr(expt)	Mr(calc)	ppm	Start	End	Miss	Ions	Peptide
817.4935	816.4862	816.4705	19.2	385 -	393	0	---	R.TVGAGVVSK.I
1631.9464	1630.9392	1630.7699	104	46 -	59	1	63	R.AFDQIDNAPEERAR.G
1654.9735	1653.9662	1653.8686	59.0	267 -	282	0	---	K.LLDQGEAGDNIGALLR.G

No match to: 790.5086, 804.3324, 808.4445, 832.4057, 837.5595, 842.5625, 857.5055, 930.5245, 964.5936, 982.6070, 986.6133, 1006.6445, 1015.6326, 1027.6664, 1042.6300, 1078.6445, 1107.8344, 1142.6742, 1153.6546, 1165.6966, 1179.7073, 1186.7303, 1197.6582, 1214.7097, 1219.6443, 1233.7159, 1240.6944, 1265.7392, 1269.7243, 1326.7883, 1368.8298, 1380.8667, 1428.9212, 1450.8531, 1475.8727, 1477.8489, 1484.8980, 1530.8700, 1557.9283, 1575.0154, 1598.9839, 1617.9280, 1658.9825, 1708.0185, 1712.9684, 1724.0543, 1729.0035, 1733.0874, 1756.0868, 1781.0936, 1797.1145, 1804.0575, 1813.1035, 1824.0974, 1839.0932, 1850.0717, 1882.1314, 1900.1334, 1915.1486, 1931.1352, 1938.1476, 1947.1417, 1949.1320, 1952.1522, 1966.1980, 1981.1803, 1994.1847, 2005.2465, 2021.1854, 2042.2539, 2044.2373, 2064.3065, 2117.3601, 2149.2508, 2181.2406, 2211.3162, 2215.2391, 2229.3298, 2271.3649, 2284.3753, 2384.2549, 2399.3191, 2438.4857, 2450.5041, 2498.5023, 2615.5335, 2645.5873, 2681.5950, 2705.4362, 2717.3922, 2729.6061, 2745.6012, 2808.6023, 2856.6076, 2872.6380, 3048.6449, 3284.6878

Search Parameters

```
Type of search         : MS/MS Ion Search
Enzyme                 : Trypsin
Fixed modifications    : Carbamidomethyl (C)
Variable modifications : Oxidation (M)
Mass values            : Monoisotopic
Protein Mass           : Unrestricted
Peptide Mass Tolerance : ± 150 ppm
Fragment Mass Tolerance: ± 2 Da
Max Missed Cleavages   : 1
Instrument type        : MALDI-TOF-TOF
   Query1  (790.5086,1+): <no title>
   Query2  (804.3324,1+): <no title>
   Query3  (808.4445,1+): <no title>
   Query4  (817.4935,1+): <no title>
   Query5  (832.4057,1+): <no title>
   Query6  (837.5595,1+): <no title>
   Query7  (842.5625,1+): <no title>
   Query8  (857.5055,1+): <no title>
   Query9  (930.5245,1+): <no title>
  Query10  (964.5936,1+): <no title>
  Query11  (982.6070,1+): <no title>
  Query12  (986.6133,1+): <no title>
 Query13  (1006.6445,1+): <no title>
 Query14  (1015.6326,1+): <no title>
 Query15  (1027.6664,1+): <no title>
 Query16  (1042.6300,1+): <no title>
 Query17  (1078.6445,1+): <no title>
 Query18  (1107.8344,1+): <no title>
 Query19  (1142.6742,1+): <no title>
 Query20  (1153.6546,1+): <no title>
 Query21  (1165.6966,1+): <no title>
 Query22  (1179.7073,1+): <no title>
 Query23  (1186.7303,1+): <no title>
 Query24  (1197.6582,1+): <no title>
 Query25  (1214.7097,1+): <no title>
 Query26  (1219.6443,1+): <no title>
```

```
 Query27 (1233.7159,1+): <no title>
 Query28 (1240.6944,1+): <no title>
 Query29 (1265.7392,1+): <no title>
 Query30 (1269.7243,1+): <no title>
 Query31 (1326.7883,1+): <no title>
 Query32 (1368.8298,1+): <no title>
 Query33 (1380.8667,1+): <no title>
 Query34 (1428.9212,1+): <no title>
 Query35 (1450.8531,1+): <no title>
 Query36 (1475.8727,1+): <no title>
 Query37 (1477.8489,1+): <no title>
 Query38 (1484.8980,1+): <no title>
 Query39 (1530.8700,1+): <no title>
 Query40 (1557.9283,1+): <no title>
 Query41 (1575.0154,1+): <no title>
 Query42 (1598.9839,1+): <no title>
 Query43 (1617.9280,1+): <no title>
 Query44 (1631.9464,1+): <no title>
 Query45 (1654.9735,1+): <no title>
 Query46 (1658.9825,1+): <no title>
 Query47 (1708.0185,1+): <no title>
 Query48 (1712.9684,1+): <no title>
 Query49 (1724.0543,1+): <no title>
 Query50 (1729.0035,1+): <no title>
 Query51 (1733.0874,1+): <no title>
 Query52 (1756.0868,1+): <no title>
 Query53 (1781.0936,1+): <no title>
 Query54 (1797.1145,1+): <no title>
 Query55 (1804.0575,1+): <no title>
 Query56 (1813.1035,1+): <no title>
 Query57 (1824.0974,1+): <no title>
 Query58 (1839.0932,1+): <no title>
 Query59 (1850.0717,1+): <no title>
 Query60 (1882.1314,1+): <no title>
 Query61 (1900.1334,1+): <no title>
 Query62 (1915.1486,1+): <no title>
 Query63 (1931.1352,1+): <no title>
 Query64 (1938.1476,1+): <no title>
 Query65 (1947.1417,1+): <no title>
 Query66 (1949.1320,1+): <no title>
 Query67 (1952.1522,1+): <no title>
 Query68 (1966.1980,1+): <no title>
 Query69 (1981.1803,1+): <no title>
 Query70 (1994.1847,1+): <no title>
 Query71 (2005.2465,1+): <no title>
 Query72 (2021.1854,1+): <no title>
 Query73 (2042.2539,1+): <no title>
 Query74 (2044.2373,1+): <no title>
 Query75 (2064.3065,1+): <no title>
 Query76 (2117.3601,1+): <no title>
 Query77 (2149.2508,1+): <no title>
 Query78 (2181.2406,1+): <no title>
 Query79 (2211.3162,1+): <no title>
 Query80 (2215.2391,1+): <no title>
 Query81 (2229.3298,1+): <no title>
 Query82 (2271.3649,1+): <no title>
 Query83 (2284.3753,1+): <no title>
 Query84 (2384.2549,1+): <no title>
 Query85 (2399.3191,1+): <no title>
 Query86 (2438.4857,1+): <no title>
 Query87 (2450.5041,1+): <no title>
 Query88 (2498.5023,1+): <no title>
 Query89 (2615.5335,1+): <no title>
 Query90 (2645.5873,1+): <no title>
 Query91 (2681.5950,1+): <no title>
 Query92 (2705.4362,1+): <no title>
 Query93 (2717.3922,1+): <no title>
 Query94 (2729.6061,1+): <no title>
 Query95 (2745.6012,1+): <no title>
 Query96 (2808.6023,1+): <no title>
 Query97 (2856.6076,1+): <no title>
 Query98 (2872.6380,1+): <no title>
 Query99 (3048.6449,1+): <no title>
Query100 (3284.6878,1+): <no title>
```

Mascot: http://www.matrixscience.com/

MALDI MS MS Data Sheet of Urine Isolate

C:\Users\madhurekha\Desktop\MALDI_Data\16th batch data 070718\BHU\4\0_B4\1\1549.8852.LIFT\1SRef

Comment 1

Comment 2

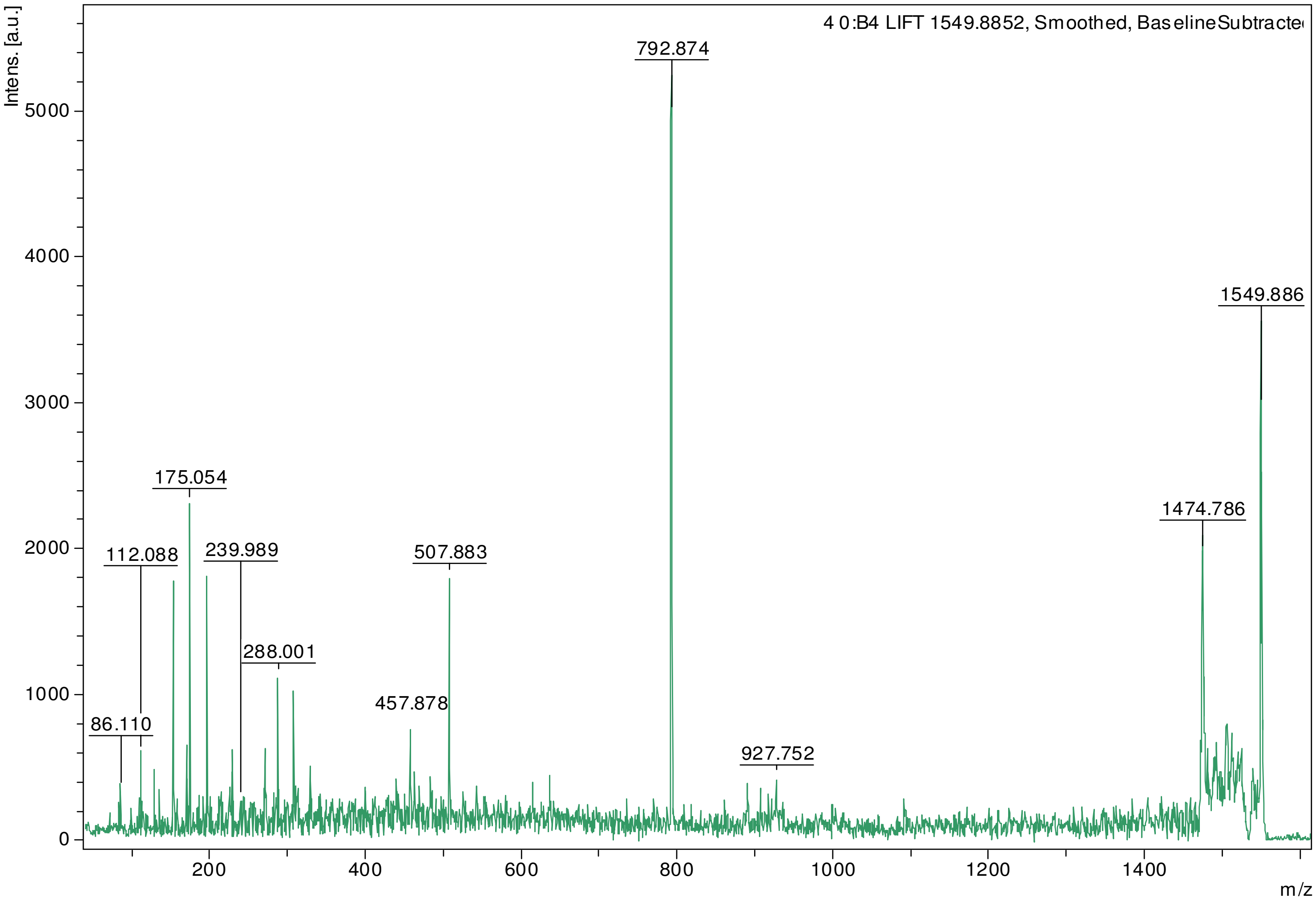

m/z	S/N	Quality Fac.	Res.	Intens.	Area
84.068	3.3	50	756	242.18	30
86.110	5.0	159	584	362.70	59
100.086	3.3	47	969	237.91	27
112.088	8.4	902	927	611.99	83
129.090	6.9	553	1113	498.01	65
136.056	5.0	375	1236	354.06	44
154.042	25.7	7002	1274	1802.78	250
171.046	9.8	232	1235	685.35	111
175.054	33.4	134020	1263	2326.66	377
187.055	4.6	63	1413	317.18	49
192.051	4.6	150	1230	317.69	59
197.043	26.2	14133	1353	1832.65	316
215.022	4.8	54	1311	342.32	67
217.033	3.6	63	1178	255.60	56
239.989	4.1	34	1363	299.93	64
254.003	3.8	34	1181	285.44	75
288.001	14.0	3247	1392	1140.48	294
308.098	11.9	1759	1506	1026.27	265
313.985	4.3	36	1287	374.91	116
328.951	6.3	35	1530	582.30	160
342.941	3.6	36	1204	340.02	125
457.878	6.3	173	1864	822.18	277
463.897	3.7	42	1734	482.20	178
507.883	12.8	14729	1942	1822.50	672
792.874	31.5	26310	2026	4995.12	3261
927.752	3.1	48	2494	450.85	302
1472.429	5.1	73	1637	557.19	1242
1474.786	18.0	4905	2006	1989.73	3628
1477.907	3.6	70	1640	389.04	869
1481.154	4.7	235	1938	514.35	979
1491.241	7.0	48	2756	737.59	1000
1494.643	4.0	159	1661	413.17	939
1497.937	3.5	100	2729	363.21	501
1500.212	3.9	36	2628	402.09	579
1504.691	8.6	138	2632	875.96	1264
1506.579	5.6	88	2728	572.35	799
1510.589	4.7	78	2731	468.12	656
1512.524	6.1	156	1811	604.22	1288

C:\Users\madhurekha\Desktop\MALDI_Data\16th batch data 070718\BHU\4\0_B4\1\1549.8852.LIFT\1SRef

m/z	S/N	Quality Fac.	Res.	Intens.	Area
1517.668	4.9	115	1684	473.46	1089
1520.891	5.5	189	1689	527.18	1212
1524.039	5.9	275	1693	563.19	1294
1538.552	5.7	95	2245	538.33	950
1543.084	3.7	235	3365	346.10	410
1549.886	32.7	18955	3045	2986.80	3937

C:\Users\madhurekha\Desktop\MALDI_Data\16th batch data 070718\BHU\4\0_B4\1\1989.1187.LIFT\1SRef

Comment 1

Comment 2

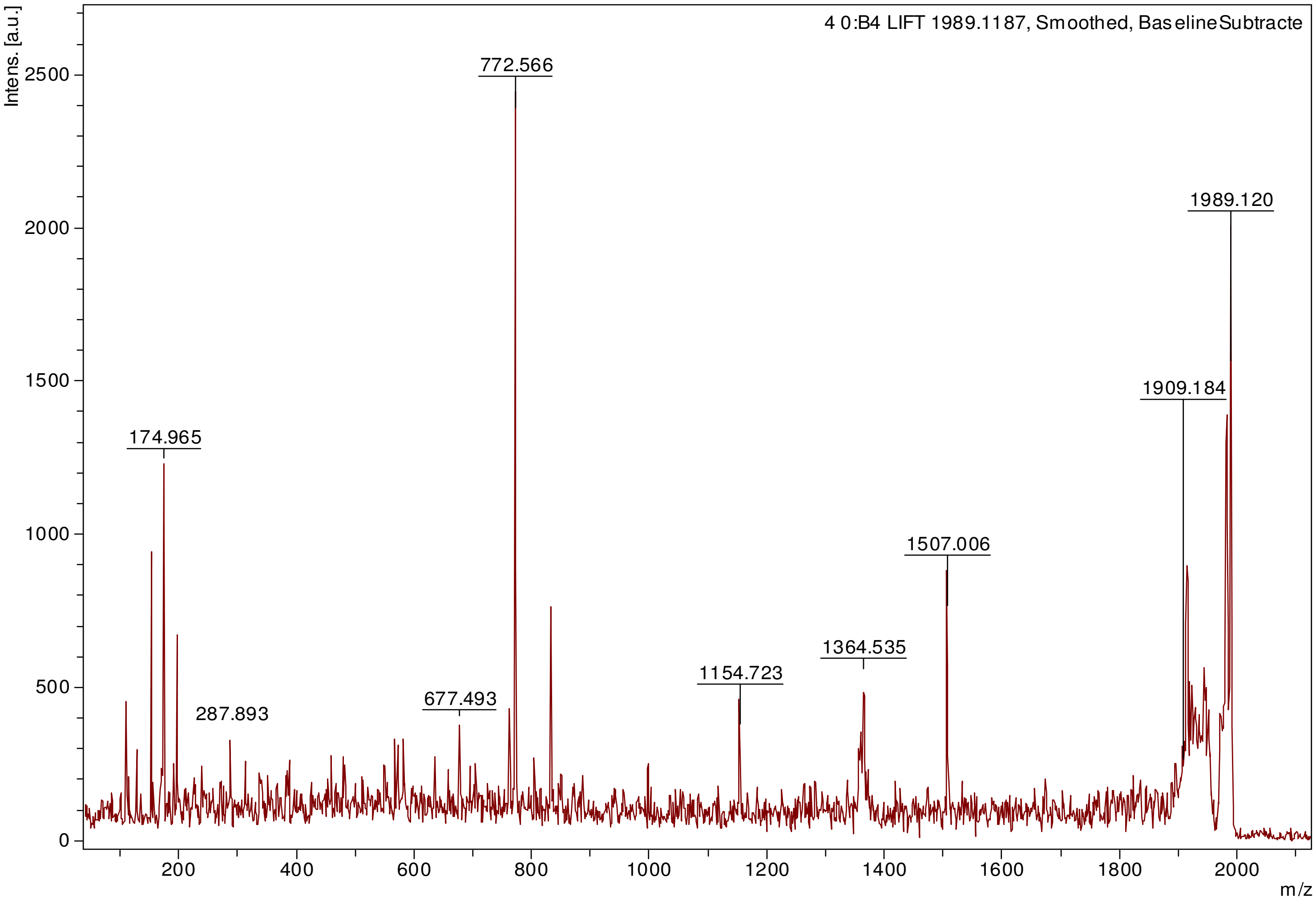

m/z	S/N	Quality Fac.	Res.	Intens.	Area
110.008	6.9	11791	1096	452.00	51
112.017	6.6	746	1105	432.46	49
115.006	3.2	569	1208	209.33	22
129.017	4.8	356	1267	303.09	35
153.970	15.4	23766	1430	959.92	118
171.951	3.9	40	1150	244.45	43
174.965	20.0	29380	1367	1232.30	184
191.939	4.3	153	1068	264.17	56
196.941	11.3	4058	1240	695.09	131
239.885	3.9	42	1370	247.64	53
287.893	5.3	190	1040	365.12	126
313.817	3.6	31	1222	255.23	83
388.741	3.1	121	1571	261.04	85
582.549	3.1	51	1742	331.58	163
677.493	3.5	94	2172	392.44	191
762.515	4.1	279	2409	448.94	233
772.566	21.9	45367	2257	2374.99	1341
833.469	6.5	1019	2339	696.85	424
1154.723	3.5	171	2826	364.71	307
1364.535	4.9	175	2763	544.67	626
1507.006	6.6	1520	1891	753.36	1517
1909.184	3.0	31	4067	250.10	368
1914.977	10.0	720	2747	813.11	1775
1920.499	4.4	128	2206	336.87	919
1923.700	5.6	190	2489	446.19	1081
1928.734	4.9	120	2492	389.35	944
1935.686	4.3	109	2150	315.11	890
1939.152	4.2	65	2154	319.44	902
1944.389	7.4	244	2157	527.89	1493
1947.635	3.8	168	2389	283.20	724
1950.867	4.5	154	2166	334.99	947
1971.236	5.2	222	2401	361.47	933
1974.294	4.2	191	2192	290.77	824
1977.810	4.7	210	3621	334.64	575
1980.041	4.6	118	4427	320.73	451
1982.323	17.2	5856	4284	1198.11	1745
1985.696	5.7	86	3259	393.13	754
1989.120	22.5	18551	3049	1549.41	3182

C:\Users\madhurekha\Desktop\MALDI_Data\16th batch data 070718\BHU\4\0_B4\1\2705.3299.LIFT\1SRef

Comment 1

Comment 2

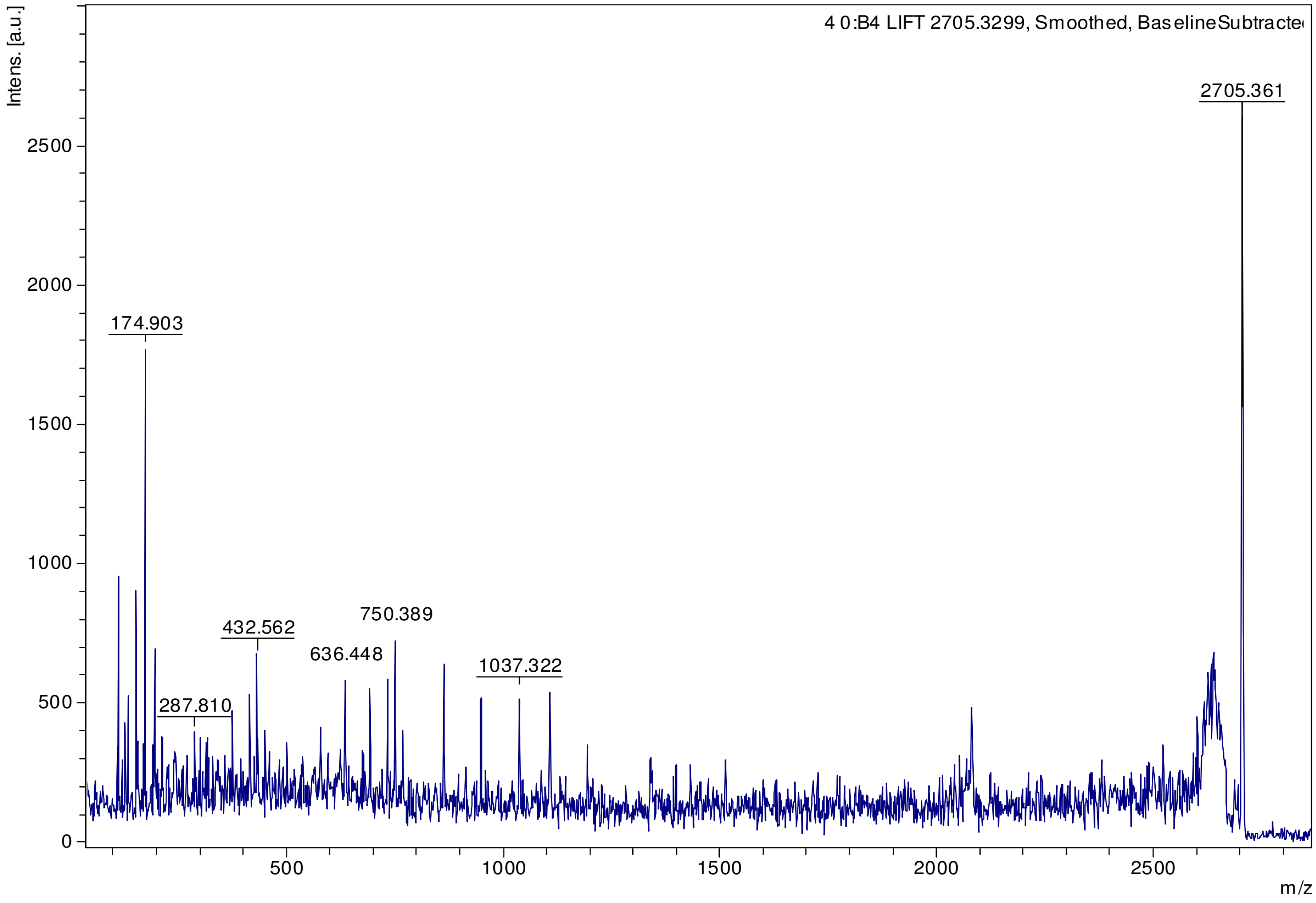

m/z	S/N	Quality Fac.	Res.	Intens.	Area
111.949	7.7	3570	701	879.65	158
128.947	3.5	300	686	395.68	84
135.910	4.6	1225	1452	518.75	55
153.901	7.9	7500	1378	884.01	113
157.907	3.5	47	1112	391.59	64
170.892	3.5	52	1386	386.79	56
174.903	16.2	22161	1567	1780.25	232
191.865	3.0	380	1306	332.14	58
196.880	6.4	1273	1517	696.09	107
213.864	3.8	109	1495	422.51	72
246.813	3.0	72	1460	337.90	70
271.797	3.1	41	1586	360.25	76
287.810	3.4	113	1152	401.88	125
301.711	3.2	95	2092	385.45	70
318.707	3.0	37	1465	377.66	104
375.642	3.2	94	1262	442.48	173
415.600	4.0	116	1447	578.26	223
432.562	4.6	134	1687	672.08	233
636.448	3.8	143	1605	612.30	370
693.370	3.6	175	2165	569.49	287
733.351	3.8	114	2028	594.00	346
750.389	5.0	777	1955	773.36	483
863.339	4.5	825	2222	665.55	449
950.391	3.7	368	2107	518.67	427
1037.322	4.0	266	2701	553.28	407
1108.270	3.2	84	2122	447.44	469
2624.440	5.5	178	3925	489.81	1138
2629.634	4.1	58	2923	356.91	1117
2634.529	3.6	63	2925	313.11	981
2638.928	5.2	103	2933	416.48	1305
2643.993	4.5	95	2936	365.00	1145
2649.931	3.9	66	2943	316.70	996
2705.361	19.7	14471	4251	1547.18	3477

C:\Users\madhurekha\Desktop\MALDI_Data\16th batch data 070718\BHU\4\0_B4\1\1SRef

Comment 1

Comment 2

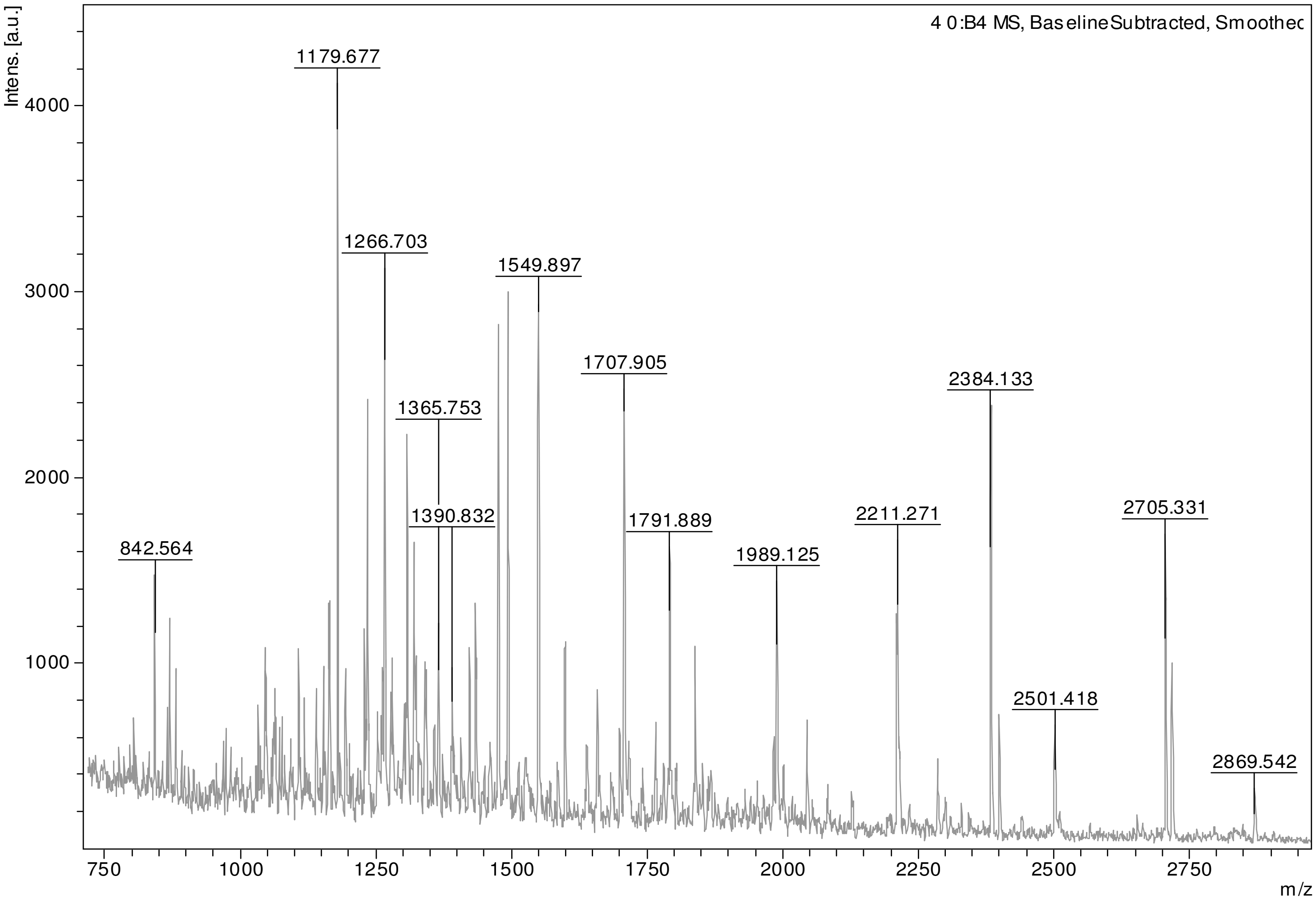

m/z	S/N	Quality Fac.	Res.	Intens.	Area
842.564	8.5	1479	4124	1141.12	406
870.594	6.3	810	3626	843.69	360
1107.621	7.0	368	4361	920.00	480
1153.695	6.6	435	7420	864.70	296
1162.697	6.4	79	5812	840.57	356
1164.675	7.2	202	5022	945.43	466
1179.677	29.4	13665	5948	3848.40	1651
1193.698	6.3	216	6222	828.88	347
1229.897	8.1	861	6044	1057.70	470
1234.747	19.4	1998	5959	2515.46	1162
1266.703	20.2	7660	5763	2607.41	1294
1280.752	6.7	596	5593	860.29	449
1308.753	11.9	2233	5063	1524.58	903
1320.680	8.4	366	4093	1070.68	798
1340.777	6.2	847	5061	785.04	486
1365.753	7.5	1696	5083	940.11	599
1390.832	6.2	571	4547	770.56	566
1422.803	7.2	1515	5520	886.07	563
1434.864	9.2	2839	6133	1119.94	654
1475.856	22.1	8771	6540	2640.49	1515
1493.845	22.8	12552	6061	2706.52	1720
1549.897	25.0	18485	6858	2870.36	1737
1598.938	9.2	6046	6343	1023.43	712
1657.932	6.1	633	4757	649.91	642
1707.905	22.7	25644	6707	2328.44	1744
1791.889	13.1	2963	5653	1260.03	1223
1839.063	8.8	5216	6673	816.14	712
1989.125	14.0	8035	6866	1079.87	1006
2045.190	6.4	1553	5619	454.15	531
2211.271	22.9	11216	9070	1293.53	1038
2384.133	35.3	15290	8029	1597.91	1625
2399.201	10.1	6130	8689	449.30	425
2501.418	10.3	1439	6624	403.84	528
2705.331	37.2	60552	9753	1110.08	1115
2717.253	19.7	3369	7836	580.75	734
2869.542	6.6	1077	6568	160.65	262

MATRIX SCIENCE Mascot Search Results

```
User           : sanovar
Email          : sanovar@sandor.co.in
Search title   :
MS data file   : DATA.TXT
Database       : SwissProt 2018_06 (557713 sequences; 200130199 residues)
Taxonomy       : Bacteria (Eubacteria) (333552 sequences)
Timestamp      : 11 Jul 2018 at 12:08:07 GMT
Warning        : A Peptide summary report will usually give a much clearer picture of MS/MS search results.
Top Score      : 75 for TKT1_ECOLI, Transketolase 1 OS=Escherichia coli (strain K12) OX=83333 GN=tktA PE=1 SV=5
```

Mascot Score Histogram

Protein score is -10*Log(P), where P is the probability that the observed match is a random event.
Protein scores greater than 68 are significant (p<0.05).
Protein scores are derived from ions scores as a non-probabilistic basis for ranking protein hits.

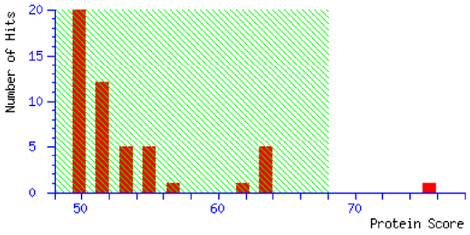

Protein Summary Report

Format As | Protein Summary (deprecated) | Help

Significance threshold p< 0.05 Max. number of hits 20

Preferred taxonomy All entries

Re-Search All | Search Unmatched

Index

	Accession	Mass	Score	Description
1.	TKT1_ECOLI	72451	75	Transketolase 1 OS=Escherichia coli (strain K12) OX=83333 GN=tktA PE=1 SV=5
2.	RS1_DICD3	61334	63	30S ribosomal protein S1 OS=Dickeya dadantii (strain 3937) OX=198628 GN=rpsA PE=3 SV=2
3.	RS1_ECO57	61235	63	30S ribosomal protein S1 OS=Escherichia coli O157:H7 OX=83334 GN=rpsA PE=3 SV=1
4.	RS1_ECOL6	61235	63	30S ribosomal protein S1 OS=Escherichia coli O6:H1 (strain CFT073 / ATCC 700928 / UPEC) OX=199310 GN=rp
5.	RS1_ECOLI	61235	63	30S ribosomal protein S1 OS=Escherichia coli (strain K12) OX=83333 GN=rpsA PE=1 SV=1
6.	RS1_SHIFL	61235	63	30S ribosomal protein S1 OS=Shigella flexneri OX=623 GN=rpsA PE=3 SV=1
7.	ATPG_BACCA	32323	61	ATP synthase gamma chain OS=Bacillus caldotenax OX=1395 GN=atpG PE=3 SV=1
8.	DXR_CLOBM	43041	57	1-deoxy-D-xylulose 5-phosphate reductoisomerase OS=Clostridium botulinum (strain Loch Maree / Type A3) (
9.	DXR_CLOB6	43168	56	1-deoxy-D-xylulose 5-phosphate reductoisomerase OS=Clostridium botulinum (strain 657 / Type Ba4) OX=515
10.	DXR_CLOBJ	43182	56	1-deoxy-D-xylulose 5-phosphate reductoisomerase OS=Clostridium botulinum (strain Kyoto / Type A2) OX=53
11.	DXR_CLOBL	43212	56	1-deoxy-D-xylulose 5-phosphate reductoisomerase OS=Clostridium botulinum (strain Langeland / NCTC 10281
12.	DXR_CLOBK	43169	55	1-deoxy-D-xylulose 5-phosphate reductoisomerase OS=Clostridium botulinum (strain Okra / Type B1) OX=498
13.	ERA_LACH4	34027	54	GTPase Era OS=Lactobacillus helveticus (strain DPC 4571) OX=405566 GN=era PE=3 SV=1
14.	ASSY_SOLUE	50458	54	Argininosuccinate synthase OS=Solibacter usitatus (strain Ellin6076) OX=234267 GN=argG PE=3 SV=1
15.	IOLG_RUBXD	37446	54	Inositol 2-dehydrogenase OS=Rubrobacter xylanophilus (strain DSM 9941 / NBRC 16129) OX=266117 GN=iolG P
16.	UREG_RHOPB	22253	53	Urease accessory protein UreG OS=Rhodopseudomonas palustris (strain BisB18) OX=316056 GN=ureG PE=3 SV=1
17.	CCPA_STAEQ	36500	53	Catabolite control protein A OS=Staphylococcus epidermidis (strain ATCC 35984 / RP62A) OX=176279 GN=ccp
18.	CCPA_STAES	36500	53	Catabolite control protein A OS=Staphylococcus epidermidis (strain ATCC 12228) OX=176280 GN=ccpA PE=3 S
19.	ACKA_PORG3	43586	52	Acetate kinase OS=Porphyromonas gingivalis (strain ATCC 33277 / DSM 20709 / CIP 103683 / JCM 12257 / NC
20.	RPOC_PSYCK	155806	52	DNA-directed RNA polymerase subunit beta' OS=Psychrobacter cryohalolentis (strain K5) OX=335284 GN=rpoC

Results List

1. TKT1_ECOLI **Mass:** 72451 **Score:** 75 **Expect:** 0.0096 **Matches:** 6
Transketolase 1 OS=Escherichia coli (strain K12) OX=83333 GN=tktA PE=1 SV=5

Observed	Mr(expt)	Mr(calc)	ppm	Start - End	Miss	Ions	Peptide
1033.5958	1032.5885	1032.5604	27.2	245 - 254	0	---	K.TIIGFGSPNK.A
1277.7589	1276.7516	1276.6353	91.1	205 - 214	0	---	R.FEAYGWHVIR.D
1329.7326	1328.7254	1328.7452	-14.93	343 - 354	1	---	K.EFIAKLQANPAK.I
1983.1414	1982.1341	1982.0261	54.5	604 - 621	1	---	K.AVTARVAVEAGIADYWYK.Y
1989.1250	1988.1177	1987.9712	73.7	255 - 274	0	63	K.AGTHDSHGAPLGDAEIALTR.E
2001.0988	2000.0915	1999.9309	80.3	580 - 597	1	---	R.VVSMPSTDAFDKQDAAYR.E

No match to: 720.3201, 721.3315, 724.3214, 804.3108, 842.5642, 865.5456, 870.5940, 881.3242, 969.5548, 973.5948, 982.5138, 1037.6072, 1045.6282, 1047.6804, 1064.6488, 1072.6447, 1077.2496, 1082.6362, 1093.6181, 1107.6212, 1109.5910, 1118.5838, 1140.6515, 1153.6950, 1163.6855, 1164.6754, 1165.6684, 1179.6771, 1184.6858, 1193.6977, 1195.6893, 1201.7343, 1229.8974, 1232.6901, 1253.7005, 1259.2801, 1259.7540, 1263.2247, 1263.7024, 1266.7035, 1280.7521, 1283.7721, 1302.8009, 1306.7653, 1308.7528, 1310.3027, 1320.6798, 1323.2817, 1323.7637, 1324.7890, 1326.3207, 1332.7618, 1340.7775, 1343.7777, 1357.7890, 1365.7525, 1373.7532, 1390.8324, 1393.8069, 1407.7954, 1422.8033, 1434.8643, 1460.8550, 1475.8557, 1490.8367, 1493.8451, 1549.8969, 1598.9377, 1638.9642, 1657.9317, 1684.0126, 1699.9639, 1707.9051, 1716.9715, 1740.9356, 1781.0033, 1789.0003, 1791.8887, 1804.0297, 1839.0630, 1847.0491, 1852.0436, 2083.1533, 2128.1603, 2211.2706, 2215.3315, 2286.2807, 2299.3189, 2329.2726, 2343.2471, 2384.1325, 2399.2011, 2441.2881, 2501.4182, 2510.3224, 2705.3310, 2717.2530, 2848.5165, 2869.5420, 3223.3745, 3264.5742, 3312.3559

2. RS1_DICD3 **Mass:** 61334 **Score:** 63 **Expect:** 0.18 **Matches:** 2
30S ribosomal protein S1 OS=Dickeya dadantii (strain 3937) OX=198628 GN=rpsA PE=3 SV=2

Observed	Mr(expt)	Mr(calc)	ppm	Start - End	Miss	Ions	Peptide
1329.7326	1328.7254	1328.7664	-30.87	451 - 464	1	---	K.GAIVTGKVTAVDAK.G
1549.8969	1548.8896	1548.7784	71.8	465 - 479	0	63	K.GATVELADGVEGYLR.A

No match to: 720.3201, 721.3315, 724.3214, 804.3108, 842.5642, 865.5456, 870.5940, 881.3242, 969.5548, 973.5948, 982.5138, 1033.5958, 1037.6072, 1045.6282, 1047.6804, 1064.6488, 1072.6447, 1077.2496, 1082.6362, 1093.6181, 1107.6212, 1109.5910,

1118.5838, 1140.6515, 1153.6950, 1163.6855, 1164.6754, 1165.6684, 1179.6771, 1184.6858, 1193.6977, 1195.6893, 1201.7343, 1229.8974, 1232.6901, 1253.7005, 1259.2801, 1259.7540, 1263.2247, 1263.7024, 1266.7035, 1277.7589, 1280.7521, 1283.7721, 1302.8009, 1306.7653, 1308.7528, 1310.3027, 1320.6798, 1323.2817, 1323.7637, 1324.7890, 1326.3207, 1332.7618, 1340.7775, 1343.7777, 1357.7890, 1365.7525, 1373.7532, 1390.8324, 1393.8069, 1407.7954, 1422.8033, 1434.8643, 1460.8550, 1475.8557, 1490.8367, 1493.8451, 1598.9377, 1638.9642, 1657.9317, 1684.0126, 1699.9639, 1707.9051, 1716.9715, 1740.9356, 1781.0033, 1789.0003, 1791.8887, 1804.0297, 1839.0630, 1847.0491, 1852.0436, 1983.1414, 1989.1250, 2001.0988, 2083.1533, 2128.1603, 2211.2706, 2215.3315, 2286.2807, 2299.3189, 2329.2726, 2343.2471, 2384.1325, 2399.2011, 2441.2881, 2501.4182, 2510.3224, 2705.3310, 2717.2530, 2848.5165, 2869.5420, 3223.3745, 3264.5742, 3312.3559

3. RS1_ECO57 **Mass:** 61235 **Score:** 63 **Expect:** 0.18 **Matches:** 2

30S ribosomal protein S1 OS=Escherichia coli O157:H7 OX=83334 GN=rpsA PE=3 SV=1

Observed	Mr(expt)	Mr(calc)	ppm	Start	End	Miss	Ions	Peptide
1329.7326	1328.7254	1328.7664	-30.87	451 -	464	1	---	K.GAIVTGKVTAVDAK.G
1549.8969	1548.8896	1548.7784	71.8	465 -	479	0	63	K.GATVELADGVEGYLR.A

No match to: 720.3201, 721.3315, 724.3214, 804.3108, 842.5642, 865.5456, 870.5940, 881.3242, 969.5548, 973.5948, 982.5138, 1033.5958, 1037.6072, 1045.6282, 1047.6804, 1064.6488, 1072.6447, 1077.2496, 1082.6362, 1093.6181, 1107.6212, 1109.5910, 1118.5838, 1140.6515, 1153.6950, 1163.6855, 1164.6754, 1165.6684, 1179.6771, 1184.6858, 1193.6977, 1195.6893, 1201.7343, 1229.8974, 1232.6901, 1253.7005, 1259.2801, 1259.7540, 1263.2247, 1263.7024, 1266.7035, 1277.7589, 1280.7521, 1283.7721, 1302.8009, 1306.7653, 1308.7528, 1310.3027, 1320.6798, 1323.2817, 1323.7637, 1324.7890, 1326.3207, 1332.7618, 1340.7775, 1343.7777, 1357.7890, 1365.7525, 1373.7532, 1390.8324, 1393.8069, 1407.7954, 1422.8033, 1434.8643, 1460.8550, 1475.8557, 1490.8367, 1493.8451, 1598.9377, 1638.9642, 1657.9317, 1684.0126, 1699.9639, 1707.9051, 1716.9715, 1740.9356, 1781.0033, 1789.0003, 1791.8887, 1804.0297, 1839.0630, 1847.0491, 1852.0436, 1983.1414, 1989.1250, 2001.0988, 2083.1533, 2128.1603, 2211.2706, 2215.3315, 2286.2807, 2299.3189, 2329.2726, 2343.2471, 2384.1325, 2399.2011, 2441.2881, 2501.4182, 2510.3224, 2705.3310, 2717.2530, 2848.5165, 2869.5420, 3223.3745, 3264.5742, 3312.3559

4. RS1_ECOL6 **Mass:** 61235 **Score:** 63 **Expect:** 0.18 **Matches:** 2

30S ribosomal protein S1 OS=Escherichia coli O6:H1 (strain CFT073 / ATCC 700928 / UPEC) OX=199310 GN=rpsA PE=3 SV=1

Observed	Mr(expt)	Mr(calc)	ppm	Start	End	Miss	Ions	Peptide
1329.7326	1328.7254	1328.7664	-30.87	451 -	464	1	---	K.GAIVTGKVTAVDAK.G
1549.8969	1548.8896	1548.7784	71.8	465 -	479	0	63	K.GATVELADGVEGYLR.A

No match to: 720.3201, 721.3315, 724.3214, 804.3108, 842.5642, 865.5456, 870.5940, 881.3242, 969.5548, 973.5948, 982.5138, 1033.5958, 1037.6072, 1045.6282, 1047.6804, 1064.6488, 1072.6447, 1077.2496, 1082.6362, 1093.6181, 1107.6212, 1109.5910, 1118.5838, 1140.6515, 1153.6950, 1163.6855, 1164.6754, 1165.6684, 1179.6771, 1184.6858, 1193.6977, 1195.6893, 1201.7343, 1229.8974, 1232.6901, 1253.7005, 1259.2801, 1259.7540, 1263.2247, 1263.7024, 1266.7035, 1277.7589, 1280.7521, 1283.7721, 1302.8009, 1306.7653, 1308.7528, 1310.3027, 1320.6798, 1323.2817, 1323.7637, 1324.7890, 1326.3207, 1332.7618, 1340.7775, 1343.7777, 1357.7890, 1365.7525, 1373.7532, 1390.8324, 1393.8069, 1407.7954, 1422.8033, 1434.8643, 1460.8550, 1475.8557, 1490.8367, 1493.8451, 1598.9377, 1638.9642, 1657.9317, 1684.0126, 1699.9639, 1707.9051, 1716.9715, 1740.9356, 1781.0033, 1789.0003, 1791.8887, 1804.0297, 1839.0630, 1847.0491, 1852.0436, 1983.1414, 1989.1250, 2001.0988, 2083.1533, 2128.1603, 2211.2706, 2215.3315, 2286.2807, 2299.3189, 2329.2726, 2343.2471, 2384.1325, 2399.2011, 2441.2881, 2501.4182, 2510.3224, 2705.3310, 2717.2530, 2848.5165, 2869.5420, 3223.3745, 3264.5742, 3312.3559

5. RS1_ECOLI **Mass:** 61235 **Score:** 63 **Expect:** 0.18 **Matches:** 2

30S ribosomal protein S1 OS=Escherichia coli (strain K12) OX=83333 GN=rpsA PE=1 SV=1

Observed	Mr(expt)	Mr(calc)	ppm	Start	End	Miss	Ions	Peptide
1329.7326	1328.7254	1328.7664	-30.87	451 -	464	1	---	K.GAIVTGKVTAVDAK.G
1549.8969	1548.8896	1548.7784	71.8	465 -	479	0	63	K.GATVELADGVEGYLR.A

No match to: 720.3201, 721.3315, 724.3214, 804.3108, 842.5642, 865.5456, 870.5940, 881.3242, 969.5548, 973.5948, 982.5138, 1033.5958, 1037.6072, 1045.6282, 1047.6804, 1064.6488, 1072.6447, 1077.2496, 1082.6362, 1093.6181, 1107.6212, 1109.5910, 1118.5838, 1140.6515, 1153.6950, 1163.6855, 1164.6754, 1165.6684, 1179.6771, 1184.6858, 1193.6977, 1195.6893, 1201.7343, 1229.8974, 1232.6901, 1253.7005, 1259.2801, 1259.7540, 1263.2247, 1263.7024, 1266.7035, 1277.7589, 1280.7521, 1283.7721, 1302.8009, 1306.7653, 1308.7528, 1310.3027, 1320.6798, 1323.2817, 1323.7637, 1324.7890, 1326.3207, 1332.7618, 1340.7775, 1343.7777, 1357.7890, 1365.7525, 1373.7532, 1390.8324, 1393.8069, 1407.7954, 1422.8033, 1434.8643, 1460.8550, 1475.8557, 1490.8367, 1493.8451, 1598.9377, 1638.9642, 1657.9317, 1684.0126, 1699.9639, 1707.9051, 1716.9715, 1740.9356, 1781.0033, 1789.0003, 1791.8887, 1804.0297, 1839.0630, 1847.0491, 1852.0436, 1983.1414, 1989.1250, 2001.0988, 2083.1533, 2128.1603, 2211.2706, 2215.3315, 2286.2807, 2299.3189, 2329.2726, 2343.2471, 2384.1325, 2399.2011, 2441.2881, 2501.4182, 2510.3224, 2705.3310, 2717.2530, 2848.5165, 2869.5420, 3223.3745, 3264.5742, 3312.3559

6. RS1_SHIFL **Mass:** 61235 **Score:** 63 **Expect:** 0.18 **Matches:** 2

30S ribosomal protein S1 OS=Shigella flexneri OX=623 GN=rpsA PE=3 SV=1

Observed	Mr(expt)	Mr(calc)	ppm	Start	End	Miss	Ions	Peptide
1329.7326	1328.7254	1328.7664	-30.87	451 -	464	1	---	K.GAIVTGKVTAVDAK.G
1549.8969	1548.8896	1548.7784	71.8	465 -	479	0	63	K.GATVELADGVEGYLR.A

No match to: 720.3201, 721.3315, 724.3214, 804.3108, 842.5642, 865.5456, 870.5940, 881.3242, 969.5548, 973.5948, 982.5138, 1033.5958, 1037.6072, 1045.6282, 1047.6804, 1064.6488, 1072.6447, 1077.2496, 1082.6362, 1093.6181, 1107.6212, 1109.5910, 1118.5838, 1140.6515, 1153.6950, 1163.6855, 1164.6754, 1165.6684, 1179.6771, 1184.6858, 1193.6977, 1195.6893, 1201.7343, 1229.8974, 1232.6901, 1253.7005, 1259.2801, 1259.7540, 1263.2247, 1263.7024, 1266.7035, 1277.7589, 1280.7521, 1283.7721, 1302.8009, 1306.7653, 1308.7528, 1310.3027, 1320.6798, 1323.2817, 1323.7637, 1324.7890, 1326.3207, 1332.7618, 1340.7775, 1343.7777, 1357.7890, 1365.7525, 1373.7532, 1390.8324, 1393.8069, 1407.7954, 1422.8033, 1434.8643, 1460.8550, 1475.8557, 1490.8367, 1493.8451, 1598.9377, 1638.9642, 1657.9317, 1684.0126, 1699.9639, 1707.9051, 1716.9715, 1740.9356, 1781.0033, 1789.0003, 1791.8887, 1804.0297, 1839.0630, 1847.0491, 1852.0436, 1983.1414, 1989.1250, 2001.0988, 2083.1533, 2128.1603, 2211.2706, 2215.3315, 2286.2807, 2299.3189, 2329.2726, 2343.2471, 2384.1325, 2399.2011, 2441.2881, 2501.4182, 2510.3224, 2705.3310, 2717.2530, 2848.5165, 2869.5420, 3223.3745, 3264.5742, 3312.3559

7. ATPG_BACCA **Mass:** 32323 **Score:** 61 **Expect:** 0.27 **Matches:** 12

ATP synthase gamma chain OS=Bacillus caldotenax OX=1395 GN=atpG PE=3 SV=1

Observed	Mr(expt)	Mr(calc)	ppm	Start	End	Miss	Ions	Peptide
1140.6515	1139.6442	1139.7026	-51.22	2 -	11	1	---	M.KPLASLRDIK.T
1164.6754	1163.6681	1163.6008	57.8	42 -	50	0	---	R.EIVRPYMEK.I
1266.7035	1265.6962	1265.6914	3.77	64 -	74	0	---	R.SHPMLVSRPVK.K + Oxidation (M)
1320.6798	1319.6725	1319.7019	-22.31	41 -	50	1	---	K.REIVRPYMEK.I
1332.7618	1331.7545	1331.6867	50.9	51 -	63	0	---	K.IQEVVAMSASAAR.S
1343.7777	1342.7704	1342.7391	23.3	130 -	140	1	---	K.RNMPVILDITR.L + Oxidation (M)
1373.7532	1372.7459	1372.7602	-10.43	189 -	200	0	---	K.LLPLTDFLAENK.Q
1657.9317	1656.9244	1656.9199	2.75	189 -	202	1	---	K.LLPLTDFLAENKQR.T
1699.9639	1698.9566	1698.8941	36.8	141 -	155	1	---	R.LPDQPSFADIKEIAR.K
1781.0033	1779.9960	1779.8243	96.5	243 -	258	1	---	R.MTAMKNATDNANDVIR.T + Oxidation (M)
2399.2011	2398.1938	2398.2566	-26.20	131 -	151	1	---	R.NMPVILDITRLPDQPSFADIK.E + Oxidation (M)
2510.3224	2509.3151	2509.2668	19.2	42 -	63	1	---	R.EIVRPYMEKIQEVVAMSASAAR.S + 2 Oxidation (M)

No match to: 720.3201, 721.3315, 724.3214, 804.3108, 842.5642, 865.5456, 870.5940, 881.3242, 969.5548, 973.5948, 982.5138, 1033.5958, 1037.6072, 1045.6282, 1047.6804, 1064.6488, 1072.6447, 1077.2496, 1082.6362, 1093.6181, 1107.6212, 1109.5910, 1118.5838, 1153.6950, 1163.6855, 1165.6684, 1179.6771, 1184.6858, 1193.6977, 1195.6893, 1201.7343, 1229.8974, 1232.6901, 1253.7005, 1259.2801, 1259.7540, 1263.2247, 1263.7024, 1277.7589, 1280.7521, 1283.7721, 1302.8009, 1306.7653, 1308.7528, 1310.3027, 1323.2817, 1323.7637, 1324.7890, 1326.3207, 1329.7326, 1340.7775, 1357.7890, 1365.7525, 1390.8324, 1393.8069, 1407.7954, 1422.8033, 1434.8643, 1460.8550, 1475.8557, 1490.8367, 1493.8451, 1549.8969, 1598.9377, 1638.9642, 1684.0126, 1707.9051, 1716.9715, 1740.9356, 1789.0003, 1791.8887, 1804.0297, 1839.0630, 1847.0491, 1852.0436, 1983.1414, 1989.1250, 2001.0988, 2083.1533, 2128.1603, 2211.2706, 2215.3315, 2286.2807, 2299.3189, 2329.2726, 2343.2471, 2384.1325, 2441.2881, 2501.4182, 2705.3310, 2717.2530, 2848.5165, 2869.5420, 3223.3745, 3264.5742, 3312.3559

8. DXR_CLOBM **Mass:** 43041 **Score:** 57 **Expect:** 0.73 **Matches:** 12

1-deoxy-D-xylulose 5-phosphate reductoisomerase OS=Clostridium botulinum (strain Loch Maree / Type A3) OX=498214 GN=dxr PE=3 SV=

Observed	Mr(expt)	Mr(calc)	ppm	Start	End	Miss	Ions	Peptide
842.5642	841.5569	841.5637	-8.05	41	47	1	---	K.KVIEIIK.E
1193.6977	1192.6904	1192.6815	7.46	30	40	1	---	K.LVAISANKSYK.K
1320.6798	1319.6725	1319.7119	-29.82	207	218	1	---	R.KISIDSATLMNK.G
1329.7326	1328.7254	1328.7299	-3.44	113	125	1	---	K.AIESGKDIALANK.E
1343.7777	1342.7704	1342.8224	-38.72	42	52	1	---	K.VIEIIKEFKPK.Y
1357.7890	1356.7817	1356.7864	-3.46	126	138	0	---	K.ETLVVAGELVISK.A
1716.9715	1715.9642	1715.9206	25.4	166	181	1	---	K.EEVKNIIVTASGGPFR.G
1852.0436	1851.0364	1850.9713	35.2	238	253	0	---	K.VVVHPQSIVHSMVEYK.D
2083.1533	2082.1461	2082.1936	-22.83	119	138	1	---	K.DIALANKETLVVAGELVISK.A
2128.1603	2127.1531	2127.1398	6.23	318	338	0	---	K.GGVMPAILNGANEVAVDLFLK.G
2329.2726	2328.2653	2328.2511	6.10	318	340	1	---	K.GGVMPAILNGANEVAVDLFLKGK.I + Oxidation (M)
3312.3559	3311.3486	3311.6353	-86.54	238	267	1	---	K.VVVHPQSIVHSMVEYKDGSVIAQMATPDMK.L + Oxidation (M)

No match to: 720.3201, 721.3315, 724.3214, 804.3108, 865.5456, 870.5940, 881.3242, 969.5548, 973.5948, 982.5138, 1033.5958, 1037.6072, 1045.6282, 1047.6804, 1064.6488, 1072.6447, 1077.2496, 1082.6362, 1093.6181, 1107.6212, 1109.5910, 1118.5838, 1140.6515, 1153.6950, 1163.6855, 1164.6754, 1165.6684, 1179.6771, 1184.6858, 1195.6893, 1201.7343, 1229.8974, 1232.6901, 1253.7005, 1259.2801, 1259.7540, 1263.2247, 1263.7024, 1266.7035, 1277.7589, 1280.7521, 1283.7721, 1302.8009, 1306.7653, 1308.7528, 1310.3027, 1323.2817, 1323.7637, 1324.7890, 1326.3207, 1332.7618, 1340.7775, 1365.7525, 1373.7532, 1390.8324, 1393.8069, 1407.7954, 1422.8033, 1434.8643, 1460.8550, 1475.8557, 1490.8367, 1493.8451, 1549.8969, 1598.9377, 1638.9642, 1657.9317, 1684.0126, 1699.9639, 1707.9051, 1740.9356, 1781.0033, 1789.0003, 1791.8887, 1804.0297, 1839.0630, 1847.0491, 1983.1414, 1989.1250, 2001.0988, 2211.2706, 2215.3315, 2286.2807, 2299.3189, 2343.2471, 2384.1325, 2399.2011, 2441.2881, 2501.4182, 2510.3224, 2705.3310, 2717.2530, 2848.5165, 2869.5420, 3223.3745, 3264.5742

9. DXR_CLOB6 **Mass:** 43168 **Score:** 56 **Expect:** 0.94 **Matches:** 12

1-deoxy-D-xylulose 5-phosphate reductoisomerase OS=Clostridium botulinum (strain 657 / Type Ba4) OX=515621 GN=dxr PE=3 SV=1

Observed	Mr(expt)	Mr(calc)	ppm	Start	End	Miss	Ions	Peptide
842.5642	841.5569	841.5637	-8.05	41	47	1	---	K.KVIEIIK.E
1193.6977	1192.6904	1192.6815	7.46	30	40	1	---	K.LVAISANKSYK.K
1320.6798	1319.6725	1319.7119	-29.82	207	218	1	---	R.KISIDSATLMNK.G
1329.7326	1328.7254	1328.7299	-3.44	113	125	1	---	K.AIESGKDIALANK.E
1343.7777	1342.7704	1342.8224	-38.72	42	52	1	---	K.VIEIIKEFKPK.Y
1357.7890	1356.7817	1356.7864	-3.46	126	138	0	---	K.ETLVVAGELVISK.A
1716.9715	1715.9642	1715.9206	25.4	166	181	1	---	K.EEVKNIIVTASGGPFR.G
1852.0436	1851.0364	1850.9713	35.2	238	253	0	---	K.VVVHPQSIVHSMVEYK.D
2083.1533	2082.1461	2082.1936	-22.83	119	138	1	---	K.DIALANKETLVVAGELVISK.A
2128.1603	2127.1531	2127.1398	6.23	318	338	0	---	K.GGVMPAILNGANEVAVDLFLK.G
2329.2726	2328.2653	2328.2511	6.10	318	340	1	---	K.GGVMPAILNGANEVAVDLFLKGK.I + Oxidation (M)
3312.3559	3311.3486	3311.6353	-86.54	238	267	1	---	K.VVVHPQSIVHSMVEYKDGSVIAQMATPDMK.L + Oxidation (M)

No match to: 720.3201, 721.3315, 724.3214, 804.3108, 865.5456, 870.5940, 881.3242, 969.5548, 973.5948, 982.5138, 1033.5958, 1037.6072, 1045.6282, 1047.6804, 1064.6488, 1072.6447, 1077.2496, 1082.6362, 1093.6181, 1107.6212, 1109.5910, 1118.5838, 1140.6515, 1153.6950, 1163.6855, 1164.6754, 1165.6684, 1179.6771, 1184.6858, 1195.6893, 1201.7343, 1229.8974, 1232.6901, 1253.7005, 1259.2801, 1259.7540, 1263.2247, 1263.7024, 1266.7035, 1277.7589, 1280.7521, 1283.7721, 1302.8009, 1306.7653, 1308.7528, 1310.3027, 1323.2817, 1323.7637, 1324.7890, 1326.3207, 1332.7618, 1340.7775, 1365.7525, 1373.7532, 1390.8324, 1393.8069, 1407.7954, 1422.8033, 1434.8643, 1460.8550, 1475.8557, 1490.8367, 1493.8451, 1549.8969, 1598.9377, 1638.9642, 1657.9317, 1684.0126, 1699.9639, 1707.9051, 1740.9356, 1781.0033, 1789.0003, 1791.8887, 1804.0297, 1839.0630, 1847.0491, 1983.1414, 1989.1250, 2001.0988, 2211.2706, 2215.3315, 2286.2807, 2299.3189, 2343.2471, 2384.1325, 2399.2011, 2441.2881, 2501.4182, 2510.3224, 2705.3310, 2717.2530, 2848.5165, 2869.5420, 3223.3745, 3264.5742

10. DXR_CLOBJ **Mass:** 43182 **Score:** 56 **Expect:** 0.94 **Matches:** 12

1-deoxy-D-xylulose 5-phosphate reductoisomerase OS=Clostridium botulinum (strain Kyoto / Type A2) OX=536232 GN=dxr PE=3 SV=1

Observed	Mr(expt)	Mr(calc)	ppm	Start	End	Miss	Ions	Peptide
842.5642	841.5569	841.5637	-8.05	41	47	1	---	K.KVIEIIK.E
1193.6977	1192.6904	1192.6815	7.46	30	40	1	---	K.LVAISANKSYK.K
1320.6798	1319.6725	1319.7119	-29.82	207	218	1	---	R.KISIDSATLMNK.G
1329.7326	1328.7254	1328.7299	-3.44	113	125	1	---	K.AIESGKDIALANK.E
1343.7777	1342.7704	1342.8224	-38.72	42	52	1	---	K.VIEIIKEFKPK.Y
1357.7890	1356.7817	1356.7864	-3.46	126	138	0	---	K.ETLVVAGELVISK.A
1716.9715	1715.9642	1715.9206	25.4	166	181	1	---	K.EEVKNIIVTASGGPFR.G
1852.0436	1851.0364	1850.9713	35.2	238	253	0	---	K.VVVHPQSIVHSMVEYK.D
2083.1533	2082.1461	2082.1936	-22.83	119	138	1	---	K.DIALANKETLVVAGELVISK.A
2128.1603	2127.1531	2127.1398	6.23	318	338	0	---	K.GGVMPAILNGANEVAVDLFLK.G
2329.2726	2328.2653	2328.2511	6.10	318	340	1	---	K.GGVMPAILNGANEVAVDLFLKGK.I + Oxidation (M)
3312.3559	3311.3486	3311.6353	-86.54	238	267	1	---	K.VVVHPQSIVHSMVEYKDGSVIAQMATPDMK.L + Oxidation (M)

No match to: 720.3201, 721.3315, 724.3214, 804.3108, 865.5456, 870.5940, 881.3242, 969.5548, 973.5948, 982.5138, 1033.5958, 1037.6072, 1045.6282, 1047.6804, 1064.6488, 1072.6447, 1077.2496, 1082.6362, 1093.6181, 1107.6212, 1109.5910, 1118.5838, 1140.6515, 1153.6950, 1163.6855, 1164.6754, 1165.6684, 1179.6771, 1184.6858, 1195.6893, 1201.7343, 1229.8974, 1232.6901, 1253.7005, 1259.2801, 1259.7540, 1263.2247, 1263.7024, 1266.7035, 1277.7589, 1280.7521, 1283.7721, 1302.8009, 1306.7653, 1308.7528, 1310.3027, 1323.2817, 1323.7637, 1324.7890, 1326.3207, 1332.7618, 1340.7775, 1365.7525, 1373.7532, 1390.8324, 1393.8069, 1407.7954, 1422.8033, 1434.8643, 1460.8550, 1475.8557, 1490.8367, 1493.8451, 1549.8969, 1598.9377, 1638.9642, 1657.9317, 1684.0126, 1699.9639, 1707.9051, 1740.9356, 1781.0033, 1789.0003, 1791.8887, 1804.0297, 1839.0630, 1847.0491, 1983.1414, 1989.1250, 2001.0988, 2211.2706, 2215.3315, 2286.2807, 2299.3189, 2343.2471, 2384.1325, 2399.2011, 2441.2881, 2501.4182, 2510.3224, 2705.3310, 2717.2530, 2848.5165, 2869.5420, 3223.3745, 3264.5742

11. DXR_CLOBL **Mass:** 43212 **Score:** 56 **Expect:** 0.94 **Matches:** 12

1-deoxy-D-xylulose 5-phosphate reductoisomerase OS=Clostridium botulinum (strain Langeland / NCTC 10281 / Type F) OX=441772 GN=d

Observed	Mr(expt)	Mr(calc)	ppm	Start	End	Miss	Ions	Peptide
842.5642	841.5569	841.5637	-8.05	41	47	1	---	K.KVIEIIK.E
1193.6977	1192.6904	1192.6815	7.46	30	40	1	---	K.LVAISANKSYK.K
1320.6798	1319.6725	1319.7119	-29.82	207	218	1	---	R.KISIDSATLMNK.G
1329.7326	1328.7254	1328.7299	-3.44	113	125	1	---	K.AIESGKDIALANK.E
1343.7777	1342.7704	1342.8224	-38.72	42	52	1	---	K.VIEIIKEFKPK.Y
1357.7890	1356.7817	1356.7864	-3.46	126	138	0	---	K.ETLVVAGELVISK.A
1716.9715	1715.9642	1715.9206	25.4	166	181	1	---	K.EEVKNIIVTASGGPFR.G
1852.0436	1851.0364	1850.9713	35.2	238	253	0	---	K.VVVHPQSIVHSMVEYK.D
2083.1533	2082.1461	2082.1936	-22.83	119	138	1	---	K.DIALANKETLVVAGELVISK.A
2128.1603	2127.1531	2127.1398	6.23	318	338	0	---	K.GGVMPAILNGANEVAVDLFLK.G
2329.2726	2328.2653	2328.2511	6.10	318	340	1	---	K.GGVMPAILNGANEVAVDLFLKGK.I + Oxidation (M)
3312.3559	3311.3486	3311.6353	-86.54	238	267	1	---	K.VVVHPQSIVHSMVEYKDGSVIAQMATPDMK.L + Oxidation (M)

No match to: 720.3201, 721.3315, 724.3214, 804.3108, 865.5456, 870.5940, 881.3242, 969.5548, 973.5948, 982.5138, 1033.5958, 1037.6072, 1045.6282, 1047.6804, 1064.6488, 1072.6447, 1077.2496, 1082.6362, 1093.6181, 1107.6212, 1109.5910, 1118.5838, 1140.6515, 1153.6950, 1163.6855, 1164.6754, 1165.6684, 1179.6771, 1184.6858, 1195.6893, 1201.7343, 1229.8974, 1232.6901, 1253.7005, 1259.2801, 1259.7540, 1263.2247, 1263.7024, 1266.7035, 1277.7589, 1280.7521, 1283.7721, 1302.8009, 1306.7653, 1308.7528, 1310.3027, 1323.2817, 1323.7637, 1324.7890, 1326.3207, 1332.7618, 1340.7775, 1365.7525, 1373.7532, 1390.8324,

1393.8069, 1407.7954, 1422.8033, 1434.8643, 1460.8550, 1475.8557, 1490.8367, 1493.8451, 1549.8969, 1598.9377, 1638.9642, 1657.9317, 1684.0126, 1699.9639, 1707.9051, 1740.9356, 1781.0033, 1789.0003, 1791.8887, 1804.0297, 1839.0630, 1847.0491, 1983.1414, 1989.1250, 2001.0988, 2211.2706, 2215.3315, 2286.2807, 2299.3189, 2343.2471, 2384.1325, 2399.2011, 2441.2881, 2501.4182, 2510.3224, 2705.3310, 2717.2530, 2848.5165, 2869.5420, 3223.3745, 3264.5742

12. DXR_CLOBK **Mass:** 43169 **Score:** 55 **Expect:** 1 **Matches:** 12

1-deoxy-D-xylulose 5-phosphate reductoisomerase OS=Clostridium botulinum (strain Okra / Type B1) OX=498213 GN=dxr PE=3 SV=1

Observed	Mr(expt)	Mr(calc)	ppm	Start	End	Miss	Ions	Peptide
842.5642	841.5569	841.5637	-8.05	41	47	1	---	K.KVIEIIK.E
1193.6977	1192.6904	1192.6815	7.46	30	40	1	---	K.LVAISANKSYK.K
1320.6798	1319.6725	1319.7119	-29.82	207	218	1	---	R.KISIDSATLMNK.G
1329.7326	1328.7254	1328.7299	-3.44	113	125	1	---	K.AIESGKDIALANK.E
1343.7777	1342.7704	1342.8224	-38.72	42	52	1	---	K.VIEIIKEFKPK.Y
1357.7890	1356.7817	1356.7864	-3.46	126	138	0	---	K.ETLVVAGELVISK.A
1716.9715	1715.9642	1715.9206	25.4	166	181	1	---	K.EEVKNIIVTASGGPFR.G
1852.0436	1851.0364	1850.9713	35.2	238	253	0	---	K.VVVHPQSIVHSMVEYK.D
2083.1533	2082.1461	2082.1936	-22.83	119	138	1	---	K.DIALANKETLVVAGELVISK.A
2128.1603	2127.1531	2127.1398	6.23	318	338	0	---	K.GGVMPAILNGANEVAVDLFLK.G
2329.2726	2328.2653	2328.2511	6.10	318	340	1	---	K.GGVMPAILNGANEVAVDLFLKGK.I + Oxidation (M)
3312.3559	3311.3486	3311.6353	-86.54	238	267	1	---	K.VVVHPQSIVHSMVEYKDGSVIAQMATPDMK.L + Oxidation (M)

No match to: 720.3201, 721.3315, 724.3214, 804.3108, 865.5456, 870.5940, 881.3242, 969.5548, 973.5948, 982.5138, 1033.5958, 1037.6072, 1045.6282, 1047.6804, 1064.6488, 1072.6447, 1077.2496, 1082.6362, 1093.6181, 1107.6212, 1109.5910, 1118.5838, 1140.6515, 1153.6950, 1163.6855, 1164.6754, 1165.6684, 1179.6771, 1184.6858, 1195.6893, 1201.7343, 1229.8974, 1232.6901, 1253.7005, 1259.2801, 1259.7540, 1263.2247, 1263.7024, 1266.7035, 1277.7589, 1280.7521, 1283.7721, 1302.8009, 1306.7653, 1308.7528, 1310.3027, 1323.2817, 1323.7637, 1324.7890, 1326.3207, 1332.7618, 1340.7775, 1365.7525, 1373.7532, 1390.8324, 1393.8069, 1407.7954, 1422.8033, 1434.8643, 1460.8550, 1475.8557, 1490.8367, 1493.8451, 1549.8969, 1598.9377, 1638.9642, 1657.9317, 1684.0126, 1699.9639, 1707.9051, 1740.9356, 1781.0033, 1789.0003, 1791.8887, 1804.0297, 1839.0630, 1847.0491, 1983.1414, 1989.1250, 2001.0988, 2211.2706, 2215.3315, 2286.2807, 2299.3189, 2343.2471, 2384.1325, 2399.2011, 2441.2881, 2501.4182, 2510.3224, 2705.3310, 2717.2530, 2848.5165, 2869.5420, 3223.3745, 3264.5742

13. ERA_LACH4 **Mass:** 34027 **Score:** 54 **Expect:** 1.2 **Matches:** 7

GTPase Era OS=Lactobacillus helveticus (strain DPC 4571) OX=405566 GN=era PE=3 SV=1

Observed	Mr(expt)	Mr(calc)	ppm	Start	End	Miss	Ions	Peptide
842.5642	841.5569	841.5385	21.8	242	250	1	---	K.GIIIGKGGK.M
1195.6893	1194.6821	1194.6608	17.8	261	270	1	---	R.KEIEHLLGEK.V
1232.6901	1231.6828	1231.6635	15.7	133	142	0	---	K.LLLIMDSYHK.L
1253.7005	1252.6932	1252.6485	35.7	22	32	0	---	K.STLMNYLVGQK.V
1365.7525	1364.7453	1364.7466	-0.95	275	284	1	---	R.LWVKVQHNWR.S
1490.8367	1489.8294	1489.8140	10.3	103	115	1	---	K.GDQYIANLLKEVK.V
1549.8969	1548.8896	1548.8624	17.6	262	274	1	28	K.EIEHLLGEKVNLR.L

No match to: 720.3201, 721.3315, 724.3214, 804.3108, 865.5456, 870.5940, 881.3242, 969.5548, 973.5948, 982.5138, 1033.5958, 1037.6072, 1045.6282, 1047.6804, 1064.6488, 1072.6447, 1077.2496, 1082.6362, 1093.6181, 1107.6212, 1109.5910, 1118.5838, 1140.6515, 1153.6950, 1163.6855, 1164.6754, 1165.6684, 1179.6771, 1184.6858, 1193.6977, 1201.7343, 1229.8974, 1259.2801, 1259.7540, 1263.2247, 1263.7024, 1266.7035, 1277.7589, 1280.7521, 1283.7721, 1302.8009, 1306.7653, 1308.7528, 1310.3027, 1320.6798, 1323.2817, 1323.7637, 1324.7890, 1326.3207, 1329.7326, 1332.7618, 1340.7775, 1343.7777, 1357.7890, 1373.7532, 1390.8324, 1393.8069, 1407.7954, 1422.8033, 1434.8643, 1460.8550, 1475.8557, 1493.8451, 1598.9377, 1638.9642, 1657.9317, 1684.0126, 1699.9639, 1707.9051, 1716.9715, 1740.9356, 1781.0033, 1789.0003, 1791.8887, 1804.0297, 1839.0630, 1847.0491, 1852.0436, 1983.1414, 1989.1250, 2001.0988, 2083.1533, 2128.1603, 2211.2706, 2215.3315, 2286.2807, 2299.3189, 2329.2726, 2343.2471, 2384.1325, 2399.2011, 2441.2881, 2501.4182, 2510.3224, 2705.3310, 2717.2530, 2848.5165, 2869.5420, 3223.3745, 3264.5742, 3312.3559

14. ASSY_SOLUE **Mass:** 50458 **Score:** 54 **Expect:** 1.4 **Matches:** 11

Argininosuccinate synthase OS=Solibacter usitatus (strain Ellin6076) OX=234267 GN=argG PE=3 SV=1

Observed	Mr(expt)	Mr(calc)	ppm	Start	End	Miss	Ions	Peptide
1153.6950	1152.6877	1152.6139	64.0	206	215	1	---	K.DLEHLDKGVK.I
1253.7005	1252.6932	1252.6775	12.5	2	13	0	---	M.GNILQNLPAGEK.V
1266.7035	1265.6962	1265.6723	18.9	108	119	0	---	R.AVTGTMLVIAMK.E + 2 Oxidation (M)
1324.7890	1323.7817	1323.6531	97.2	271	282	0	---	R.HGLGVSDQIENR.I
1343.7777	1342.7704	1342.7569	10.1	355	366	1	---	R.AITGEVTVELRR.G
1357.7890	1356.7817	1356.7224	43.7	216	227	0	---	K.IVEPIMGVASWR.E
1373.7532	1372.7459	1372.7173	20.8	216	227	0	---	K.IVEPIMGVASWR.E + Oxidation (M)
1657.9317	1656.9244	1656.9021	13.4	213	227	1	---	K.GVKIVEPIMGVASWR.E + Oxidation (M)
1699.9639	1698.9566	1698.9417	8.76	351	365	1	---	R.WVARAITGEVTVELR.R
1989.1250	1988.1177	1987.9938	62.3	14	32	0	10	K.VGLAFSGGLDTSAAIHWMR.A
3223.3745	3222.3672	3222.6608	-91.10	2	32	1	---	M.GNILQNLPAGEKVGLAFSGGLDTSAAIHWMR.A

No match to: 720.3201, 721.3315, 724.3214, 804.3108, 842.5642, 865.5456, 870.5940, 881.3242, 969.5548, 973.5948, 982.5138, 1033.5958, 1037.6072, 1045.6282, 1047.6804, 1064.6488, 1072.6447, 1077.2496, 1082.6362, 1093.6181, 1107.6212, 1109.5910, 1118.5838, 1140.6515, 1163.6855, 1164.6754, 1165.6684, 1179.6771, 1184.6858, 1193.6977, 1195.6893, 1201.7343, 1229.8974, 1232.6901, 1259.2801, 1259.7540, 1263.2247, 1263.7024, 1277.7589, 1280.7521, 1283.7721, 1302.8009, 1306.7653, 1308.7528, 1310.3027, 1320.6798, 1323.2817, 1323.7637, 1326.3207, 1329.7326, 1332.7618, 1340.7775, 1365.7525, 1390.8324, 1393.8069, 1407.7954, 1422.8033, 1434.8643, 1460.8550, 1475.8557, 1490.8367, 1493.8451, 1549.8969, 1598.9377, 1638.9642, 1684.0126, 1707.9051, 1716.9715, 1740.9356, 1781.0033, 1789.0003, 1791.8887, 1804.0297, 1839.0630, 1847.0491, 1852.0436, 1983.1414, 2001.0988, 2083.1533, 2128.1603, 2211.2706, 2215.3315, 2286.2807, 2299.3189, 2329.2726, 2343.2471, 2384.1325, 2399.2011, 2441.2881, 2501.4182, 2510.3224, 2705.3310, 2717.2530, 2848.5165, 2869.5420, 3264.5742, 3312.3559

15. IOLG_RUBXD **Mass:** 37446 **Score:** 54 **Expect:** 1.5 **Matches:** 5

Inositol 2-dehydrogenase OS=Rubrobacter xylanophilus (strain DSM 9941 / NBRC 16129) OX=266117 GN=iolG PE=3 SV=1

Observed	Mr(expt)	Mr(calc)	ppm	Start	End	Miss	Ions	Peptide
1072.6447	1071.6374	1071.5924	42.0	35	44	0	---	R.LVAVADLDTR.R
1093.6181	1092.6108	1092.6114	-0.50	122	130	1	---	R.KLVQVGFMR.R + Oxidation (M)
1393.8069	1392.7996	1392.8089	-6.69	249	262	0	---	R.GTVQVPPLSGAIVR.R
1549.8969	1548.8896	1548.9100	-13.16	249	263	1	38	R.GTVQVPPLSGAIVRR.G
1740.9356	1739.9283	1739.8406	50.4	186	198	0	---	R.WFIEEEIEEVYVR.G

No match to: 720.3201, 721.3315, 724.3214, 804.3108, 842.5642, 865.5456, 870.5940, 881.3242, 969.5548, 973.5948, 982.5138, 1033.5958, 1037.6072, 1045.6282, 1047.6804, 1064.6488, 1077.2496, 1082.6362, 1107.6212, 1109.5910, 1118.5838, 1140.6515, 1153.6950, 1163.6855, 1164.6754, 1165.6684, 1179.6771, 1184.6858, 1193.6977, 1195.6893, 1201.7343, 1229.8974, 1232.6901, 1253.7005, 1259.2801, 1259.7540, 1263.2247, 1263.7024, 1266.7035, 1277.7589, 1280.7521, 1283.7721, 1302.8009, 1306.7653, 1308.7528, 1310.3027, 1320.6798, 1323.2817, 1323.7637, 1324.7890, 1326.3207, 1329.7326, 1332.7618, 1340.7775, 1343.7777, 1357.7890, 1365.7525, 1373.7532, 1390.8324, 1407.7954, 1422.8033, 1434.8643, 1460.8550, 1475.8557, 1490.8367, 1493.8451, 1598.9377, 1638.9642, 1657.9317, 1684.0126, 1699.9639, 1707.9051, 1716.9715, 1781.0033, 1789.0003, 1791.8887, 1804.0297, 1839.0630, 1847.0491, 1852.0436, 1983.1414, 1989.1250, 2001.0988, 2083.1533, 2128.1603, 2211.2706, 2215.3315, 2286.2807, 2299.3189, 2329.2726, 2343.2471, 2384.1325, 2399.2011, 2441.2881, 2501.4182, 2510.3224, 2705.3310, 2717.2530, 2848.5165, 2869.5420, 3223.3745, 3264.5742, 3312.3559

16. UREG_RHOPB **Mass:** 22253 **Score:** 53 **Expect:** 1.5 **Matches:** 6

Urease accessory protein UreG OS=Rhodopseudomonas palustris (strain BisB18) OX=316056 GN=ureG PE=3 SV=1

Observed	Mr(expt)	Mr(calc)	ppm	Start	End	Miss	Ions	Peptide
721.3315	720.3242	720.3476	-32.50	166 -	171	0	---	K.MQIDAK.R + Oxidation (M)
1064.6488	1063.6415	1063.5406	94.9	21 -	29	0	---	K.TALMDLLCK.T
1082.6362	1081.6289	1081.6244	4.19	134 -	144	1	---	K.IPSKGGPGITR.S
1259.7540	1258.7467	1258.7397	5.52	193 -	204	1	---	R.IVGFIEAKGGLR.A
1434.8643	1433.8570	1433.7734	58.3	21 -	32	1	---	K.TALMDLLCKTLR.D
1989.1250	1988.1177	1988.0435	37.3	10 -	29	1	23	R.VGIGGPVGSGKTALMDLLCK.T + Oxidation (M)

No match to: 720.3201, 724.3214, 804.3108, 842.5642, 865.5456, 870.5940, 881.3242, 969.5548, 973.5948, 982.5138, 1033.5958, 1037.6072, 1045.6282, 1047.6804, 1072.6447, 1077.2496, 1093.6181, 1107.6212, 1109.5910, 1118.5838, 1140.6515, 1153.6950, 1163.6855, 1164.6754, 1165.6684, 1179.6771, 1184.6858, 1193.6977, 1195.6893, 1201.7343, 1229.8974, 1232.6901, 1253.7005, 1259.2801, 1263.2247, 1263.7024, 1266.7035, 1277.7589, 1280.7521, 1283.7721, 1302.8009, 1306.7653, 1308.7528, 1310.3027, 1320.6798, 1323.2817, 1323.7637, 1324.7890, 1326.3207, 1329.7326, 1332.7618, 1340.7775, 1343.7777, 1357.7890, 1365.7525, 1373.7532, 1390.8324, 1393.8069, 1407.7954, 1422.8033, 1460.8550, 1475.8557, 1490.8367, 1493.8451, 1549.8969, 1598.9377, 1638.9642, 1657.9317, 1684.0126, 1699.9639, 1707.9051, 1716.9715, 1740.9356, 1781.0033, 1789.0003, 1791.8887, 1804.0297, 1839.0630, 1847.0491, 1852.0436, 1983.1414, 2001.0988, 2083.1533, 2128.1603, 2211.2706, 2215.3315, 2286.2807, 2299.3189, 2329.2726, 2343.2471, 2384.1325, 2399.2011, 2441.2881, 2501.4182, 2510.3224, 2705.3310, 2717.2530, 2848.5165, 2869.5420, 3223.3745, 3264.5742, 3312.3559

17. CCPA_STAEQ **Mass:** 36500 **Score:** 53 **Expect:** 1.8 **Matches:** 10
Catabolite control protein A OS=Staphylococcus epidermidis (strain ATCC 35984 / RP62A) OX=176279 GN=ccpA PE=3 SV=1

Observed	Mr(expt)	Mr(calc)	ppm	Start	End	Miss	Ions	Peptide
1037.6072	1036.5999	1036.5553	43.0	2 -	10	0	---	M.TVTIYDVAR.E
1140.6515	1139.6442	1139.5532	79.9	81 -	90	0	---	R.GLEDIATMYK.Y
1184.6858	1183.6785	1183.5907	74.2	1 -	10	0	---	-.MTVTIYDVAR.E + Oxidation (M)
1329.7326	1328.7254	1328.7425	-12.91	43 -	53	1	---	K.RLNYRPNAVAR.G
1373.7532	1372.7459	1372.6623	60.9	151 -	162	0	---	K.DDHIASVNIDFK.Q
1393.8069	1392.7996	1392.7361	45.6	2 -	13	1	---	M.TVTIYDVAREAR.V
1493.8451	1492.8378	1492.7409	64.9	163 -	175	0	---	K.QAAEEATQYLIEK.G
2083.1533	2082.1461	2081.9840	77.8	305 -	321	0	---	K.YMNDEEIENPNVILPHR.I
2286.2807	2285.2734	2285.1910	36.1	14 -	34	1	---	R.VSMATVSRVVNGNQNVKPETR.N
2848.5165	2847.5092	2847.3926	40.9	151 -	175	1	---	K.DDHIASVNIDFKQAAEEATQYLIEK.G

No match to: 720.3201, 721.3315, 724.3214, 804.3108, 842.5642, 865.5456, 870.5940, 881.3242, 969.5548, 973.5948, 982.5138, 1033.5958, 1045.6282, 1047.6804, 1064.6488, 1072.6447, 1077.2496, 1082.6362, 1093.6181, 1107.6212, 1109.5910, 1118.5838, 1153.6950, 1163.6855, 1164.6754, 1165.6684, 1179.6771, 1193.6977, 1195.6893, 1201.7343, 1229.8974, 1232.6901, 1253.7005, 1259.2801, 1259.7540, 1263.2247, 1263.7024, 1266.7035, 1277.7589, 1280.7521, 1283.7721, 1302.8009, 1306.7653, 1308.7528, 1310.3027, 1320.6798, 1323.2817, 1323.7637, 1324.7890, 1326.3207, 1332.7618, 1340.7775, 1343.7777, 1357.7890, 1365.7525, 1390.8324, 1407.7954, 1422.8033, 1434.8643, 1460.8550, 1475.8557, 1490.8367, 1549.8969, 1598.9377, 1638.9642, 1657.9317, 1684.0126, 1699.9639, 1707.9051, 1716.9715, 1740.9356, 1781.0033, 1789.0003, 1791.8887, 1804.0297, 1839.0630, 1847.0491, 1852.0436, 1983.1414, 1989.1250, 2001.0988, 2128.1603, 2211.2706, 2215.3315, 2299.3189, 2329.2726, 2343.2471, 2384.1325, 2399.2011, 2441.2881, 2501.4182, 2510.3224, 2705.3310, 2717.2530, 2869.5420, 3223.3745, 3264.5742, 3312.3559

18. CCPA_STAES **Mass:** 36500 **Score:** 53 **Expect:** 1.8 **Matches:** 10
Catabolite control protein A OS=Staphylococcus epidermidis (strain ATCC 12228) OX=176280 GN=ccpA PE=3 SV=2

Observed	Mr(expt)	Mr(calc)	ppm	Start	End	Miss	Ions	Peptide
1037.6072	1036.5999	1036.5553	43.0	2 -	10	0	---	M.TVTIYDVAR.E
1140.6515	1139.6442	1139.5532	79.9	81 -	90	0	---	R.GLEDIATMYK.Y
1184.6858	1183.6785	1183.5907	74.2	1 -	10	0	---	-.MTVTIYDVAR.E + Oxidation (M)
1329.7326	1328.7254	1328.7425	-12.91	43 -	53	1	---	K.RLNYRPNAVAR.G
1373.7532	1372.7459	1372.6623	60.9	151 -	162	0	---	K.DDHIASVNIDFK.Q
1393.8069	1392.7996	1392.7361	45.6	2 -	13	1	---	M.TVTIYDVAREAR.V
1493.8451	1492.8378	1492.7409	64.9	163 -	175	0	---	K.QAAEEATQYLIEK.G
2083.1533	2082.1461	2081.9840	77.8	305 -	321	0	---	K.YMNDEEIENPNVILPHR.I
2286.2807	2285.2734	2285.1910	36.1	14 -	34	1	---	R.VSMATVSRVVNGNQNVKPETR.N
2848.5165	2847.5092	2847.3926	40.9	151 -	175	1	---	K.DDHIASVNIDFKQAAEEATQYLIEK.G

No match to: 720.3201, 721.3315, 724.3214, 804.3108, 842.5642, 865.5456, 870.5940, 881.3242, 969.5548, 973.5948, 982.5138, 1033.5958, 1045.6282, 1047.6804, 1064.6488, 1072.6447, 1077.2496, 1082.6362, 1093.6181, 1107.6212, 1109.5910, 1118.5838, 1153.6950, 1163.6855, 1164.6754, 1165.6684, 1179.6771, 1193.6977, 1195.6893, 1201.7343, 1229.8974, 1232.6901, 1253.7005, 1259.2801, 1259.7540, 1263.2247, 1263.7024, 1266.7035, 1277.7589, 1280.7521, 1283.7721, 1302.8009, 1306.7653, 1308.7528, 1310.3027, 1320.6798, 1323.2817, 1323.7637, 1324.7890, 1326.3207, 1332.7618, 1340.7775, 1343.7777, 1357.7890, 1365.7525, 1390.8324, 1407.7954, 1422.8033, 1434.8643, 1460.8550, 1475.8557, 1490.8367, 1549.8969, 1598.9377, 1638.9642, 1657.9317, 1684.0126, 1699.9639, 1707.9051, 1716.9715, 1740.9356, 1781.0033, 1789.0003, 1791.8887, 1804.0297, 1839.0630, 1847.0491, 1852.0436, 1983.1414, 1989.1250, 2001.0988, 2128.1603, 2211.2706, 2215.3315, 2299.3189, 2329.2726, 2343.2471, 2384.1325, 2399.2011, 2441.2881, 2501.4182, 2510.3224, 2705.3310, 2717.2530, 2869.5420, 3223.3745, 3264.5742, 3312.3559

19. ACKA_PORG3 **Mass:** 43586 **Score:** 52 **Expect:** 2 **Matches:** 10
Acetate kinase OS=Porphyromonas gingivalis (strain ATCC 33277 / DSM 20709 / CIP 103683 / JCM 12257 / NCTC 11834 / 2561) OX=43194

Observed	Mr(expt)	Mr(calc)	ppm	Start	End	Miss	Ions	Peptide
721.3315	720.3242	720.3225	2.41	358 -	363	0	---	K.VNEGMR.G + Oxidation (M)
1037.6072	1036.5999	1036.5627	35.9	15 -	22	1	---	K.YKLLEMPK.G + Oxidation (M)
1072.6447	1071.6374	1071.5560	75.9	23 -	33	0	---	K.GDVLAQGGVEK.L
1163.6855	1162.6783	1162.5903	75.6	366 -	375	0	---	K.EMVISKPESK.V + Oxidation (M)
1332.7618	1331.7545	1331.7119	32.0	364 -	375	1	---	R.GKEMVISKPESK.V
1390.8324	1389.8251	1389.7650	43.3	2 -	14	1	---	M.KVLVLNCGSSSVK.Y
1460.8550	1459.8477	1459.7995	33.0	271 -	285	1	---	K.KSGVLGVSGVSSDLR.E
1549.8969	1548.8896	1548.8987	-5.88	204 -	219	0	14	R.IITAHIGNGASIAAIK.N
2510.3224	2509.3151	2509.3866	-28.49	51 -	72	1	---	K.VVLEKDMPEHTIAVEFILSVLK.D
3223.3745	3222.3672	3222.5656	-61.56	312 -	342	1	---	K.YVGAYAAAMGGVDVLVFTGGVGENQYTTREK.V

No match to: 720.3201, 724.3214, 804.3108, 842.5642, 865.5456, 870.5940, 881.3242, 969.5548, 973.5948, 982.5138, 1033.5958, 1045.6282, 1047.6804, 1064.6488, 1077.2496, 1082.6362, 1093.6181, 1107.6212, 1109.5910, 1118.5838, 1140.6515, 1153.6950, 1164.6754, 1165.6684, 1179.6771, 1184.6858, 1193.6977, 1195.6893, 1201.7343, 1229.8974, 1232.6901, 1253.7005, 1259.2801, 1259.7540, 1263.2247, 1263.7024, 1266.7035, 1277.7589, 1280.7521, 1283.7721, 1302.8009, 1306.7653, 1308.7528, 1310.3027, 1320.6798, 1323.2817, 1323.7637, 1324.7890, 1326.3207, 1329.7326, 1340.7775, 1343.7777, 1357.7890, 1365.7525, 1373.7532, 1393.8069, 1407.7954, 1422.8033, 1434.8643, 1475.8557, 1490.8367, 1493.8451, 1598.9377, 1638.9642, 1657.9317, 1684.0126, 1699.9639, 1707.9051, 1716.9715, 1740.9356, 1781.0033, 1789.0003, 1791.8887, 1804.0297, 1839.0630, 1847.0491, 1852.0436, 1983.1414, 1989.1250, 2001.0988, 2083.1533, 2128.1603, 2211.2706, 2215.3315, 2286.2807, 2299.3189, 2329.2726, 2343.2471, 2384.1325, 2399.2011, 2441.2881, 2501.4182, 2705.3310, 2717.2530, 2848.5165, 2869.5420, 3264.5742, 3312.3559

20. RPOC_PSYCK **Mass:** 155806 **Score:** 52 **Expect:** 2.1 **Matches:** 15
DNA-directed RNA polymerase subunit beta' OS=Psychrobacter cryohalolentis (strain K5) OX=335284 GN=rpoC PE=3 SV=1

Observed	Mr(expt)	Mr(calc)	ppm	Start	End	Miss	Ions	Peptide
982.5138	981.5065	981.4879	18.9	785 -	793	0	---	K.TANSGYLTR.R
1109.5910	1108.5837	1108.4893	85.2	691 -	700	0	---	K.AMMDNLATDK.I
1153.6950	1152.6877	1152.6866	0.89	380 -	390	0	---	K.LLSHGLATTIK.A
1184.6858	1183.6785	1183.7037	-21.28	310 -	320	1	---	R.AITGSNKRPLK.S
1201.7343	1200.7270	1200.6350	76.7	1273 -	1283	1	---	K.ALNAKLEAEDK.F

1283.7721	1282.7648	1282.7721	-5.71	830 - 841	1	---	K.LGELVLGRVTAR.D
1332.7618	1331.7545	1331.6834	53.4	677 - 687	1	---	K.VVDIWSRTNDK.V
1407.7954	1406.7882	1406.8180	-21.21	1238 - 1248	1	---	K.HIEVIIRQMLR.K
1549.8969	1548.8896	1548.8552	22.3	1083 - 1095	0	16	K.EVVYFLPAETIIR.V
1657.9317	1656.9244	1656.9232	0.71	119 - 132	1	---	R.IGLLLDMTLRDIER.V
1684.0126	1683.0054	1682.8952	65.5	1111 - 1127	1	---	R.VPQASSGTKDITGGLPR.V
1716.9715	1715.9642	1715.8624	59.3	293 - 307	1	---	R.MLQESVDALLDNGRR.G
1791.8887	1790.8814	1790.9315	-28.00	760 - 775	0	15	R.EGLTVLQYFISTHGAR.K
2083.1533	2082.1461	2081.9762	81.6	1139 - 1157	1	---	K.DHAIMAEMTGVVSFGKETK.G + 2 Oxidation (M)
2705.3310	2704.3238	2704.1893	49.7	572 - 593	0	---	R.LLIWNIMPVGMSFDECNEEMTK.K + 3 Oxidation (M)

No match to: 720.3201, 721.3315, 724.3214, 804.3108, 842.5642, 865.5456, 870.5940, 881.3242, 969.5548, 973.5948, 1033.5958, 1037.6072, 1045.6282, 1047.6804, 1064.6488, 1072.6447, 1077.2496, 1082.6362, 1093.6181, 1107.6212, 1118.5838, 1140.6515, 1163.6855, 1164.6754, 1165.6684, 1179.6771, 1193.6977, 1195.6893, 1229.8974, 1232.6901, 1253.7005, 1259.2801, 1259.7540, 1263.2247, 1263.7024, 1266.7035, 1277.7589, 1280.7521, 1302.8009, 1306.7653, 1308.7528, 1310.3027, 1320.6798, 1323.2817, 1323.7637, 1324.7890, 1326.3207, 1329.7326, 1340.7775, 1343.7777, 1357.7890, 1365.7525, 1373.7532, 1390.8324, 1393.8069, 1422.8033, 1434.8643, 1460.8550, 1475.8557, 1490.8367, 1493.8451, 1598.9377, 1638.9642, 1699.9639, 1707.9051, 1740.9356, 1781.0033, 1789.0003, 1804.0297, 1839.0630, 1847.0491, 1852.0436, 1983.1414, 1989.1250, 2001.0988, 2128.1603, 2211.2706, 2215.3315, 2286.2807, 2299.3189, 2329.2726, 2343.2471, 2384.1325, 2399.2011, 2441.2881, 2501.4182, 2510.3224, 2717.2530, 2848.5165, 2869.5420, 3223.3745, 3264.5742, 3312.3559

Search Parameters

Type of search : MS/MS Ion Search
Enzyme : Trypsin
Fixed modifications : Carbamidomethyl (C)
Variable modifications : Oxidation (M)
Mass values : Monoisotopic
Protein Mass : Unrestricted
Peptide Mass Tolerance : ± 100 ppm
Fragment Mass Tolerance: ± 2 Da
Max Missed Cleavages : 1
Instrument type : MALDI-TOF-TOF
Query1 (720.3201,1+): <no title>
Query2 (721.3315,1+): <no title>
Query3 (724.3214,1+): <no title>
Query4 (804.3108,1+): <no title>
Query5 (842.5642,1+): <no title>
Query6 (865.5456,1+): <no title>
Query7 (870.5940,1+): <no title>
Query8 (881.3242,1+): <no title>
Query9 (969.5548,1+): <no title>
Query10 (973.5948,1+): <no title>
Query11 (982.5138,1+): <no title>
Query12 (1033.5958,1+): <no title>
Query13 (1037.6072,1+): <no title>
Query14 (1045.6282,1+): <no title>
Query15 (1047.6804,1+): <no title>
Query16 (1064.6488,1+): <no title>
Query17 (1072.6447,1+): <no title>
Query18 (1077.2496,1+): <no title>
Query19 (1082.6362,1+): <no title>
Query20 (1093.6181,1+): <no title>
Query21 (1107.6212,1+): <no title>
Query22 (1109.5910,1+): <no title>
Query23 (1118.5838,1+): <no title>
Query24 (1140.6515,1+): <no title>
Query25 (1153.6950,1+): <no title>
Query26 (1163.6855,1+): <no title>
Query27 (1164.6754,1+): <no title>
Query28 (1165.6684,1+): <no title>
Query29 (1179.6771,1+): <no title>
Query30 (1184.6858,1+): <no title>
Query31 (1193.6977,1+): <no title>
Query32 (1195.6893,1+): <no title>
Query33 (1201.7343,1+): <no title>
Query34 (1229.8974,1+): <no title>
Query35 (1232.6901,1+): <no title>
Query36 (1253.7005,1+): <no title>
Query37 (1259.2801,1+): <no title>
Query38 (1259.7540,1+): <no title>
Query39 (1263.2247,1+): <no title>
Query40 (1263.7024,1+): <no title>
Query41 (1266.7035,1+): <no title>
Query42 (1277.7589,1+): <no title>
Query43 (1280.7521,1+): <no title>
Query44 (1283.7721,1+): <no title>
Query45 (1302.8009,1+): <no title>
Query46 (1306.7653,1+): <no title>
Query47 (1308.7528,1+): <no title>
Query48 (1310.3027,1+): <no title>
Query49 (1320.6798,1+): <no title>
Query50 (1323.2817,1+): <no title>
Query51 (1323.7637,1+): <no title>
Query52 (1324.7890,1+): <no title>
Query53 (1326.3207,1+): <no title>
Query54 (1329.7326,1+): <no title>
Query55 (1332.7618,1+): <no title>
Query56 (1340.7775,1+): <no title>
Query57 (1343.7777,1+): <no title>
Query58 (1357.7890,1+): <no title>
Query59 (1365.7525,1+): <no title>
Query60 (1373.7532,1+): <no title>
Query61 (1390.8324,1+): <no title>
Query62 (1393.8069,1+): <no title>
Query63 (1407.7954,1+): <no title>
Query64 (1422.8033,1+): <no title>
Query65 (1434.8643,1+): <no title>
Query66 (1460.8550,1+): <no title>
Query67 (1475.8557,1+): <no title>
Query68 (1490.8367,1+): <no title>
Query69 (1493.8451,1+): <no title>
Query70 (1549.8969,1+): <no title>
Query71 (1598.9377,1+): <no title>

```
 Query72 (1638.9642,1+): <no title>
 Query73 (1657.9317,1+): <no title>
 Query74 (1684.0126,1+): <no title>
 Query75 (1699.9639,1+): <no title>
 Query76 (1707.9051,1+): <no title>
 Query77 (1716.9715,1+): <no title>
 Query78 (1740.9356,1+): <no title>
 Query79 (1781.0033,1+): <no title>
 Query80 (1789.0003,1+): <no title>
 Query81 (1791.8887,1+): <no title>
 Query82 (1804.0297,1+): <no title>
 Query83 (1839.0630,1+): <no title>
 Query84 (1847.0491,1+): <no title>
 Query85 (1852.0436,1+): <no title>
 Query86 (1983.1414,1+): <no title>
 Query87 (1989.1250,1+): <no title>
 Query88 (2001.0988,1+): <no title>
 Query89 (2083.1533,1+): <no title>
 Query90 (2128.1603,1+): <no title>
 Query91 (2211.2706,1+): <no title>
 Query92 (2215.3315,1+): <no title>
 Query93 (2286.2807,1+): <no title>
 Query94 (2299.3189,1+): <no title>
 Query95 (2329.2726,1+): <no title>
 Query96 (2343.2471,1+): <no title>
 Query97 (2384.1325,1+): <no title>
 Query98 (2399.2011,1+): <no title>
 Query99 (2441.2881,1+): <no title>
Query100 (2501.4182,1+): <no title>
Query101 (2510.3224,1+): <no title>
Query102 (2705.3310,1+): <no title>
Query103 (2717.2530,1+): <no title>
Query104 (2848.5165,1+): <no title>
Query105 (2869.5420,1+): <no title>
Query106 (3223.3745,1+): <no title>
Query107 (3264.5742,1+): <no title>
Query108 (3312.3559,1+): <no title>
```

Mascot: http://www.matrixscience.com/

Supplementary Data 1.pdf

Supplementary Data 2.pdf

Supplementary Data 3.pdf

Supplementary Data 4.pdf

Supplementary Data 5.pdf

Supplementary Data 6.pdf

Supplementary Data 7.pdf

Supplementary Data 8.pdf

Supplementary data 9.pdf

Supplementary Data 1: Effect of Growth Medium

Isolates	Absorbance in BHI broth	Absorbance in LB broth
222	0.145	0.141
989	0.164	0.165
1606	0.173	0.171
1249	0.186	0.188
997	0.198	0.195
1250	0.2	0.197
1929	0.201	0.201
3179	0.2168	0.217
1252	0.221	0.224
1749	0.224	0.218
15288	0.236	0.234
2067	0.236	0.239
1996	0.237	0.237
1220	0.243	0.241
215	0.2458	0.247
6160	0.249	0.248
216	0.2518	0.253
7834	0.259	0.255
2276	0.2698	0.271
4158	0.2715	0.268
8373	0.284	0.28
217	0.293	0.297
4148	0.2963	0.264
7772	0.299	0.3
214	0.2995	0.297
3169	0.3028	0.3
11569	0.3048	0.31
1194	0.306	0.298
2232	0.314	0.312
1607	0.318	0.316
1287	0.319	0.319
2001	0.338	0.341
6211	0.348	0.347
191	0.348	0.344
1074	0.352	0.35
1931	0.353	0.359
8678	0.357	0.354
8	0.367	0.361
213	0.3713	0.372
Isolates	Absorbance in BHI broth	Absorbance in LB broth
14122	0.3723	0.371
4079	0.3728	0.374
2989	0.373	0.371
5993	0.374	0.3728
15311	0.3818	0.38
4008	0.392	0.393
12	0.394	0.391
6122	0.399	0.398
4	0.4	0.41
4151	0.4105	0.387
11204	0.4193	0.396
210	0.4208	0.419
1876	0.424	0.426
2322	0.433	0.433
15255	0.4335	0.431
11023	0.4373	0.438
2310	0.4383	0.436
4073	0.4428	0.45
6617	0.445	0.441
8547	0.455	0.453
6	0.4565	0.457
7	0.4628	0.464
3990	0.47	0.463
2066	0.472	0.47
3303	0.472	0.478
10533	0.473	0.477
4272	0.4755	0.464
5	0.476	0.443
1886	0.477	0.475
3977	0.4843	0.486
2187	0.4865	0.486
10697	0.487	0.489
209	0.4873	0.473
4150	0.4913	0.483
3707	0.496	0.495
1214	0.499	0.52
3788	0.4998	0.53
192	0.501	0.493
7663	0.503	0.497
1008	0.505	0.5
Isolates	Absorbance in BHI broth	Absorbance in LB broth
13738	0.511	0.52

Supplementary Data 1: Effect of Growth Medium

4179	0.516	0.522
11111	0.5238	0.514
11095	0.539	0.527
13328	0.548	0.545
13409	0.5685	0.553
1922(F)	0.573	0.57
2296	0.5735	0.572
2263	0.574	0.575
4066	0.5765	0.583
6563	0.578	0.549
3906	0.58	0.574
3357	0.5855	0.58
2	0.594	0.591
13836	0.5985	0.605
9	0.6003	0.6
2359	0.6068	0.61
1609	0.624	0.62
51	0.625	0.62
190	0.628	0.631
11109	0.6333	0.627
1740	0.645	0.64
1972	0.653	0.658
3917	0.6563	0.659
1583	0.664	0.66
14514	0.6728	0.68
3869	0.6803	0.678
2965	0.6818	0.682
2972	0.686	0.681
1974	0.693	0.687
12027	0.6938	0.69
10	0.6968	0.692
11600	0.7	0.693
4268	0.7098	0.71
3294	0.712	0.688
200	0.714	0.692
2822	0.716	0.694
193	0.722	0.703
14310	0.7248	0.718
993	0.733	0.734
Isolates	Absorbance in BHI broth	Absorbance in LB broth
4182	0.738	0.736
1772	0.74	0.72

4067	0.7545	0.75
2204/2294	0.761	0.756
2517	0.764	0.761
2190	0.764	0.766
3811	0.7658	0.77
11040	0.766	0.771
1315	0.767	0.77
9607	0.773	0.778
7877	0.775	0.771
1	0.777	0.782
2551	0.779	0.781
14730	0.8078	0.798
3022	0.8125	0.82
7520	0.817	0.8
198	0.827	0.825
3807	0.8315	0.831
194	0.832	0.83
3293	0.8335	0.841
2070	0.8533	0.85
3	0.8568	0.849
1882	0.862	0.857
7327	0.862	0.86
12110	0.8693	0.872
8315	0.884	0.882
14842	0.8865	0.889
4840	0.888	0.89
8301	0.9	0.9
14696	0.9018	0.878
8342	0.905	0.9
2171	0.905	0.89
9032	0.908	0.91
2485	0.9193	0.93
14881	0.9263	0.931
3557	0.9313	0.937
4020	0.957	0.948
4289	0.9628	0.971
4238	0.963	0.967
12998	0.9638	0.965
Isolates	Absorbance in BHI broth	Absorbance in LB broth
3248	0.9693	0.97
11	0.97	0.97
10915	0.972	0.976

Supplementary Data 1: Effect of Growth Medium

3830	0.9758	0.975
9517	1.005	1.02
2717	1.012	1.06
9972	1.026	1.003
15724	1.042	1.009
8571	1.058	1.01
1318	1.085	1.049
2825	1.091	1.1
12084	1.119	1.06
10046	1.143	1.13
13423	1.147	1.136
1313	1.156	1.12
3160	1.156	1.2
4059	1.157	1.08
6752	1.163	1.03
4073	1.169	1.12
14944	1.185	1.06
14735	1.186	1.06
2164	1.187	1.11
2700	1.195	1.13
10723	1.216	1.16
3398	1.225	1.2
1210	1.226	1.21
212	1.24	1.22
10825	1.25	1.19
3724	1.269	1.25
3652	1.279	1.27
201	1.279	1.283
7884	1.282	1.26
15718	1.298	1.275
14513	1.311	1.25
6585	1.329	1.27
15521	1.33	1.31
2503	1.339	1.33
1309	1.341	1.31
2173	1.351	1.23
3709	1.371	1.36
Isolates	Absorbance in BHI broth	Absorbance in LB broth
2498	1.392	1.399
1834	1.398	1.39
10807	1.408	1.41
195	1.43	1.36

13657	1.454	1.42
4299	1.488	1.469
8436	1.51	1.5
10101	1.525	1.521
13797	1.525	1.5
13059	1.543	1.53
13603	1.553	1.526
14478	1.565	1.56
1338	1.571	1.56
3362	1.585	1.53
2588	1.59	1.6
12497	1.597	1.58
197	1.602	1.61
2056(PA)	1.632	1.6
13306	1.641	1.623
Sim	1.655	1.64
14068	1.674	1.66
1922 R	1.675	1.69
1240	1.682	1.67
12566	1.693	1.69
12617	1.752	1.67
2187	1.756	1.78
10872	1.757	1.71
3415	1.758	1.76
4227	1.762	1.73
14350	1.775	1.7
3593	1.802	1.8
3690	1.811	1.78
2010	1.842	1.8
14345	1.864	1.82
13962	1.864	1.84
10088	1.874	1.857
14203	1.886	1.83
6708	1.893	1.9
10012	1.903	1.9
2086	1.985	1.96
Isolates	Absorbance in BHI broth	Absorbance in LB broth
15603	2.011	2
2138	2.089	2.03
1337	2.099	2.06
145	2.186	2.2
2895	2.254	2.21

Supplementary Data 1: Effect of Growth Medium

12600	2.261	2.26	196	2.804	2.73
2196	2.333	2.3	497	2.939	2.84
2931	2.378	2.35	1739	3.118	2.97
15598	2.576	2.47	2887	3.423	3.08
10894	2.623	2.53	10869	3.667	3.21
2844	2.672	2.59	3420	3.689	3.29

Supplementary Data 2: Effect of Incubation Period

Isolates	6hrs (OD)	12hrs (OD)	18hrs (OD)	24hrs (OD)
222	0	0.02	0.145	0.147
989	0	0.038	0.164	0.16
1606	0	0.057	0.173	0.172
1249	0	0.071	0.186	0.183
997	0	0.102	0.198	0.195
1250	0	0.105	0.2	0.2
1929	0	0.109	0.201	0.2
3179	0	0.113	0.2168	0.2
1252	0	0.104	0.221	0.21
1749	0	0.143	0.224	0.22
15288	0	0.152	0.236	0.228
2067	0	0.137	0.236	0.23
1996	0	0.128	0.237	0.234
1220	0	0.203	0.243	0.24
215	0.03	0.208	0.2458	0.246
6160	0.02	0.211	0.249	0.245
216	0.01	0.206	0.2518	0.25
7834	0.05	0.214	0.259	0.261
2276	0	0.205	0.2698	0.27
4158	0	0.2	0.2715	0.272
8373	0	0.212	0.284	0.283
217	0.06	0.219	0.293	0.29
4148	0	0.24	0.2963	0.295
7772	0	0.247	0.299	0.3
214	0.02	0.251	0.2995	0.302
3169	0.05	0.254	0.3028	0.302
11569	0.01	0.236	0.3048	0.31
1194	0.03	0.251	0.306	0.3
2232	0	0.223	0.314	0.315
1607	0.07	0.27	0.318	0.315
1287	0.07	0.264	0.319	0.32
2001	0	0.136	0.338	0.34
6211	0	0.214	0.348	0.34
191	0	0.182	0.348	0.35
1074	0	0.203	0.352	0.35
1931	0	0.153	0.353	0.355
8678	0	0.218	0.357	0.36
8	0	0.213	0.367	0.361
213	0.02	0.221	0.3713	0.37
14122	0.09	0.227	0.3723	0.37
**Isolates**	**6hrs**	**12hrs**	**18hrs**	**24hrs**

	(OD)	(OD)	(OD)	(OD)
4079	0	0.281	0.3728	0.371
2989	0	0.238	0.373	0.372
5993	0	0.342	0.374	0.375
15311	0.07	0.277	0.3818	0.383
4008	0	0.252	0.392	0.39
12	0	0.286	0.394	0.392
6122	0	0.254	0.399	0.396
4	0	0.219	0.4	0.399
4151	0	0.214	0.4105	0.4
11204	0.03	0.268	0.4193	0.42
210	0	0.273	0.4208	0.42
1876	0.01	0.281	0.424	0.421
2322	0	0.235	0.433	0.43
15255	0.06	0.326	0.4335	0.43
11023	0.08	0.317	0.4373	0.435
2310	0	0.28	0.4383	0.435
4073	0	0.313	0.4428	0.44
6617	0	0.279	0.445	0.446
8547	0	0.324	0.455	0.45
6	0.07	0.319	0.4565	0.458
7	0.08	0.26	0.4628	0.46
3990	0.09	0.352	0.47	0.472
2066	0	0.361	0.472	0.479
3303	0	0.332	0.472	0.476
10533	0.102	0.378	0.473	0.475
4272	0.1	0.384	0.4755	0.476
5	0.009	0.404	0.476	0.477
1886	0.108	0.331	0.477	0.483
3977	0.103	0.417	0.4843	0.486
2187	0.105	0.43	0.4865	0.485
10697	0.108	0.423	0.487	0.487
209	0	0.313	0.4873	0.488
4150	0	0.419	0.4913	0.49
3707	0	0.45	0.496	0.495
1214	0	0.432	0.499	0.51
3788	0	0.347	0.4998	0.53
192	0	0.426	0.501	0.51
7663	0	0.433	0.503	0.51
1008	0	0.354	0.505	0.516
13738	0	0.472	0.511	0.523
**Isolates**	**6hrs (OD)**	**12hrs (OD)**	**18hrs (OD)**	**24hrs (OD)**

Supplementary Data 2: Effect of Incubation Period

4179	0	0.431	0.516	0.513
11111	0.103	0.425	0.5238	0.526
11095	0.121	0.319	0.539	0.542
13328	0.113	0.413	0.548	0.551
13409	0.105	0.396	0.5685	0.571
1922(F)	0	0.392	0.573	0.575
2296	0	0.36	0.5735	0.575
2263	0	0.411	0.574	0.581
4066	0	0.427	0.5765	0.582
6563	0.105	0.414	0.578	0.57
3906	0.1	0.423	0.58	0.586
3357	0.11	0.422	0.5855	0.583
2	0.12	0.437	0.594	0.59
13836	0.13	0.352	0.5985	0.597
9	0	0.418	0.6003	0.61
2359	0.12	0.404	0.6068	0.613
2359	0.108	0.441	0.6068	0.611
1609	0.103	0.511	0.624	0.62
51	0	0.439	0.625	0.628
190	0.12	0.519	0.628	0.631
11109	0.103	0.483	0.6333	0.637
1740	0.108	0.471	0.645	0.65
1972	0.101	0.478	0.653	0.651
3917	0.083	0.43	0.6563	0.65
1583	0.113	0.474	0.664	0.67
14514	0.121	0.512	0.6728	0.668
3869	0.095	0.487	0.6803	0.675
2965	0.11	0.499	0.6818	0.68
2972	0.089	0.464	0.686	0.682
1974	0.103	0.517	0.693	0.689
12027	0.091	0.481	0.6938	0.691
10	0.108	0.515	0.6968	0.697
11600	0.112	0.524	0.7	0.72
4268	0.115	0.539	0.7098	0.71
3294	0.093	0.528	0.712	0.7
200	0.104	0.564	0.714	0.719
2822	0.106	0.573	0.716	0.715
193	0.097	0.581	0.722	0.718
14310	0.086	0.552	0.7248	0.72
993	0.117	0.66	0.733	0.726
**Isolates**	**6hrs (OD)**	**12hrs (OD)**	**18hrs (OD)**	**24hrs (OD)**
4182	0.101	0.565	0.738	0.742

1772	0.12	0.627	0.74	0.751
4067	0.109	0.618	0.7545	0.752
2204/2294	0.085	0.521	0.761	0.768
2517	0.114	0.653	0.764	0.76
2190	0.11	0.552	0.764	0.767
3811	0.107	0.602	0.7658	0.77
11040	0.093	0.575	0.766	0.768
1315	0.114	0.57	0.767	0.772
9607	0.099	0.572	0.773	0.779
7877	0.127	0.622	0.775	0.8
1	0.108	0.614	0.777	0.793
2551	0.129	0.633	0.779	0.753
14730	0.13	0.658	0.8078	0.82
3022	0.136	0.72	0.8125	0.764
7520	0.132	0.691	0.817	0.804
198	0.106	0.684	0.827	0.81
3807	0.123	0.667	0.8315	0.824
194	0.139	0.7	0.832	0.838
3293	0.14	0.703	0.8335	0.834
2070	0.14	0.709	0.8533	0.85
3	0.132	0.663	0.8568	0.86
1882	0.138	0.721	0.862	0.865
7327	0.132	0.684	0.862	0.857
12110	0.135	0.711	0.8693	0.9
8315	0.134	0.723	0.884	0.847
14842	0.142	0.762	0.8865	0.89
4840	0.131	0.694	0.888	0.9
8301	0.132	0.699	0.9	0.886
14696	0.15	0.758	0.9018	0.92
8342	0.147	0.756	0.905	0.9
2171	0.143	0.762	0.905	0.92
9032	0.121	0.697	0.908	0.893
2485	0.138	0.726	0.9193	0.924
14881	0.142	0.74	0.9263	0.923
3557	0.129	0.738	0.9313	0.9
4020	0.146	0.742	0.957	0.942
4289	0.152	0.721	0.9628	0.959
4238	0.127	0.706	0.963	0.961
12998	0.153	0.71	0.9638	0.96
**Isolates**	**6hrs (OD)**	**12hrs (OD)**	**18hrs (OD)**	**24hrs (OD)**
3248	0.158	0.731	0.9693	0.968
11	0.153	0.74	0.97	0.975

Supplementary Data 2: Effect of Incubation Period

10915	0.143	0.675	0.972	0.968
3830	0.15	0.658	0.9758	0.95
9517	0.135	0.697	1.005	1.11
2717	0.156	0.743	1.012	0.988
9972	0.145	0.724	1.026	1.21
15724	0.157	0.752	1.042	1.2
8571	0.151	0.75	1.058	1.23
1318	0.136	0.597	1.085	1.1
2825	0.144	0.689	1.091	1.03
12084	0.14	0.662	1.119	1.1
10046	0.155	0.764	1.143	1.13
13423	0.151	0.743	1.147	1.15
1313	0.142	0.771	1.156	0.935
3160	0.133	0.698	1.156	0.914
4059	0.144	0.725	1.157	0.93
6752	0.159	0.764	1.163	0.926
4073	0.146	0.734	1.169	1.03
14944	0.152	0.755	1.185	1.2
14735	0.15	0.759	1.186	1.22
2164	0.147	0.713	1.187	1.2
2700	0.148	0.697	1.195	1.25
10723	0.143	0.678	1.216	1.29
3398	0.16	0.82	1.225	1.3
1210	0.16	0.792	1.226	1.23
212	0.157	0.786	1.24	1.28
10825	0.145	0.779	1.25	1.29
3724	0.162	0.783	1.269	1.272
3652	0.154	0.794	1.279	1.283
201	0.159	0.766	1.279	1.286
7884	0.142	0.749	1.282	1.24
15718	0.151	0.795	1.298	1.237
14513	0.164	0.8	1.311	1.3
6585	0.177	0.821	1.329	1.302
15521	0.17	0.814	1.33	1.31
2503	0.159	0.763	1.339	1.32
1309	0.163	0.794	1.341	1.327
2173	0.161	0.786	1.351	1.346
3709	0.154	0.775	1.371	1.35
**Isolates**	**6hrs (OD)**	**12hrs (OD)**	**18hrs (OD)**	**24hrs (OD)**
2498	0.151	0.773	1.392	1.42
1834	0.132	0.587	1.398	1.46
10807	0.128	0.693	1.408	1.44

195	0.155	0.735	1.43	1.417
13657	0.173	0.831	1.454	1.46
4299	0.167	0.786	1.488	1.49
8436	0.175	0.833	1.51	1.5
10101	0.178	0.864	1.525	1.53
13797	0.18	0.869	1.525	1.528
13059	0.182	0.873	1.543	1.6
13603	0.166	0.797	1.553	1.59
14478	0.185	0.882	1.565	1.54
1338	0.184	0.854	1.571	1.569
3362	0.189	0.931	1.585	1.583
2588	0.166	0.839	1.59	1.62
12497	0.173	0.85	1.597	1.624
197	0.19	0.92	1.602	1.66
2056(PA)	0.19	0.895	1.632	1.629
13306	0.188	0.891	1.641	1.638
Sim	0.193	0.922	1.655	1.65
14068	0.193	0.929	1.674	1.663
1922 R	0.196	0.934	1.675	1.67
1240	0.19	0.917	1.682	1.676
12566	0.178	0.864	1.693	1.709
12617	0.189	0.932	1.752	1.728
2187	0.197	0.951	1.756	1.75
10872	0.197	0.867	1.757	1.76
3415	0.195	0.924	1.758	1.76
4227	0.199	1.092	1.762	1.758
14350	0.196	0.976	1.775	1.773
3593	0.2	1.08	1.802	1.8
3690	0.191	0.955	1.811	1.82
2010	0.183	0.947	1.842	1.817
14345	0.163	0.894	1.864	1.854
13962	0.175	0.916	1.864	1.86
10088	0.182	0.922	1.874	1.872
14203	0.185	0.964	1.886	1.877
6708	0.179	0.991	1.893	1.853
10012	0.21	1.07	1.903	1.89
2086	0.2	0.996	1.985	1.92
**Isolates**	**6hrs (OD)**	**12hrs (OD)**	**18hrs (OD)**	**24hrs (OD)**
15603	0.23	1.19	2.011	2.1
2138	0.211	1.034	2.089	1.98
1337	0.213	1.06	2.099	1.91
145	0.191	0.963	2.186	2.21

Supplementary Data 2: Effect of Incubation Period

2895	0.24	1.24	2.254	2.17
12600	0.215	1.132	2.261	2.19
2196	0.221	1.29	2.333	2.24
2931	0.224	1.187	2.378	2.35
15598	0.229	1.46	2.576	2.41
10894	0.214	1.367	2.623	2.581
2844	0.211	1.375	2.672	2.593
196	0.197	1.14	2.804	2.657
497	0.206	1.259	2.939	2.734
1739	0.198	1.168	3.118	2.98
2887	0.192	1.322	3.423	3.076
10869	0.173	0.967	3.667	3.215
3420	0.155	0.966	3.689	3.361

Supplementary Data 3: Effect of salt concentration (sodium chloride) on biofilm production

Isolates	BHI W/-	NaCl 1%	NaCl 2%	NaCl 4%	NaCl 8%
222	0.145	0.157	0.169	0.276	0.227
989	0.164	0.178	0.191	0.289	0.235
1606	0.173	0.182	0.195	0.273	0.218
1249	0.186	0.197	0.218	0.291	0.211
997	0.198	0.212	0.224	0.314	0.252
1250	0.2	0.234	0.278	0.324	0.301
1929	0.201	0.214	0.267	0.322	0.286
3179	0.2168	0.223	0.269	0.319	0.274
1252	0.221	0.229	0.271	0.326	0.289
1749	0.224	0.232	0.276	0.325	0.273
15288	0.236	0.261	0.286	0.331	0.311
2067	0.236	0.243	0.267	0.319	0.253
1996	0.237	0.244	0.269	0.323	0.247
1220	0.243	0.261	0.287	0.319	0.255
215	0.2458	0.26	0.293	0.321	0.293
6160	0.249	0.254	0.279	0.311	0.262
216	0.2518	0.269	0.281	0.324	0.294
7834	0.259	0.273	0.291	0.324	0.254
2276	0.2698	0.281	0.312	0.363	0.337
4158	0.2715	0.283	0.311	0.354	0.318
8373	0.284	0.295	0.326	0.367	0.321
217	0.293	0.306	0.322	0.361	0.343
4148	0.2963	0.312	0.339	0.357	0.312
7772	0.299	0.31	0.324	0.362	0.327
214	0.2995	0.314	0.322	0.368	0.346
3169	0.3028	0.316	0.334	0.372	0.339
11569	0.3048	0.325	0.351	0.386	0.358
1194	0.306	0.32	0.346	0.377	0.33
2232	0.314	0.328	0.345	0.371	0.348
1607	0.318	0.322	0.337	0.364	0.329
1287	0.319	0.322	0.342	0.36	0.335
2001	0.338	0.344	0.36	0.382	0.354
6211	0.348	0.351	0.363	0.38	0.355
191	0.348	0.355	0.372	0.392	0.376
1074	0.352	0.35	0.377	0.391	0.382
1931	0.353	0.359	0.373	0.388	0.351
8678	0.357	0.36	0.375	0.39	0.367
8	0.367	0.37	0.379	0.392	0.381
213	0.3713	0.378	0.383	0.396	0.384
14122	0.3723	0.381	0.394	0.402	0.387
4079	0.3728	0.38	0.388	0.41	0.391

Supplementary Data 3: Effect of salt concentration (sodium chloride) on biofilm production

2989	0.373	0.384	0.391	0.413	0.401
5993	0.374	0.389	0.397	0.411	0.397
15311	0.3818	0.39	0.425	0.441	0.428
4008	0.392	0.403	0.428	0.452	0.439
12	0.394	0.407	0.431	0.455	0.442
6122	0.399	0.4	0.429	0.452	0.43
4	0.4	0.44	0.482	0.503	0.491
4151	0.4105	0.426	0.441	0.472	0.453
11204	0.4193	0.432	0.457	0.488	0.461
210	0.4208	0.443	0.479	0.511	0.495
1876	0.424	0.451	0.482	0.522	0.493
2322	0.433	0.45	0.476	0.513	0.491
15255	0.4335	0.452	0.473	0.507	0.478
11023	0.4373	0.461	0.48	0.524	0.486
2310	0.4383	0.464	0.492	0.537	0.512
4073	0.4428	0.47	0.497	0.542	0.523
6617	0.445	0.468	0.489	0.544	0.526
8547	0.455	0.47	0.494	0.541	0.528
6	0.4565	0.473	0.503	0.552	0.531
7	0.4628	0.478	0.51	0.558	0.544
3990	0.47	0.485	0.521	0.566	0.539
2066	0.472	0.482	0.517	0.561	0.533
3303	0.472	0.49	0.528	0.563	0.55
10533	0.473	0.483	0.519	0.551	0.534
4272	0.4755	0.486	0.524	0.56	0.541
5	0.476	0.491	0.533	0.572	0.566
1886	0.477	0.512	0.561	0.583	0.562
3977	0.4843	0.505	0.547	0.572	0.554
2187	0.4865	0.51	0.553	0.58	0.56
10697	0.487	0.513	0.549	0.584	0.547
209	0.4873	0.522	0.558	0.592	0.581
4150	0.4913	0.516	0.543	0.579	0.556
3707	0.496	0.515	0.538	0.566	0.542
1214	0.499	0.527	0.554	0.583	0.563
3788	0.4998	0.519	0.549	0.577	0.558
192	0.501	0.531	0.563	0.59	0.576
7663	0.503	0.535	0.568	0.597	0.582
1008	0.505	0.533	0.568	0.592	0.585
13738	0.511	0.538	0.573	0.596	0.588
4179	0.516	0.536	0.58	0.597	0.583
11111	0.5238	0.542	0.586	0.603	0.592
11095	0.539	0.548	0.591	0.614	0.596
13328	0.548	0.574	0.606	0.619	0.595

Supplementary Data 3: Effect of salt concentration (sodium chloride) on biofilm production

13409	0.5685	0.582	0.61	0.634	0.605
1922(F)	0.573	0.587	0.63	0.673	0.662
2296	0.5735	0.592	0.634	0.682	0.668
2263	0.574	0.597	0.638	0.688	0.672
4066	0.5765	0.594	0.625	0.671	0.653
6563	0.578	0.591	0.629	0.678	0.663
3906	0.58	0.602	0.635	0.684	0.671
3357	0.5855	0.611	0.639	0.682	0.668
2	0.594	0.622	0.647	0.685	0.673
13836	0.5985	0.623	0.641	0.684	0.662
9	0.6003	0.627	0.649	0.688	0.675
2359	0.6068	0.625	0.638	0.674	0.661
2359	0.6068	0.625	0.638	0.674	0.661
1609	0.624	0.644	0.67	0.695	0.682
51	0.625	0.641	0.664	0.698	0.687
190	0.628	0.644	0.669	0.75	0.693
11109	0.6333	0.649	0.672	0.783	0.681
1740	0.645	0.653	0.671	0.782	0.715
1972	0.653	0.66	0.679	0.788	0.733
3917	0.6563	0.662	0.681	0.784	0.725
1583	0.664	0.672	0.684	0.789	0.75
14514	0.6728	0.685	0.725	0.774	0.733
3869	0.6803	0.692	0.723	0.779	0.758
2965	0.6818	0.69	0.727	0.773	0.752
2972	0.686	0.695	0.724	0.781	0.748
1974	0.693	0.725	0.747	0.79	0.773
12027	0.6938	0.718	0.732	0.786	0.754
10	0.6968	0.722	0.743	0.788	0.776
11600	0.7	0.726	0.753	0.784	0.772
4268	0.7098	0.719	0.745	0.775	0.753
3294	0.712	0.733	0.759	0.786	0.766
200	0.714	0.724	0.767	0.792	0.781
2822	0.716	0.722	0.764	0.797	0.775
193	0.722	0.736	0.772	0.81	0.794
14310	0.7248	0.738	0.763	0.793	0.772
993	0.733	0.742	0.776	0.803	0.78
4182	0.738	0.745	0.771	0.804	0.786
1772	0.74	0.753	0.779	0.824	0.795
4067	0.7545	0.769	0.783	0.833	0.812
2204/2294	0.761	0.773	0.788	0.828	0.806
2517	0.764	0.764	0.773	0.816	0.798
2190	0.764	0.773	0.796	0.823	0.805
3811	0.7658	0.779	0.803	0.834	0.819

Supplementary Data 3: Effect of salt concentration (sodium chloride) on biofilm production

11040	0.766	0.782	0.814	0.846	0.817
1315	0.767	0.777	0.799	0.828	0.816
9607	0.773	0.794	0.817	0.833	0.821
7877	0.775	0.801	0.825	0.848	0.827
1	0.777	0.807	0.819	0.853	0.836
2551	0.779	0.81	0.828	0.851	0.832
14730	0.8078	0.815	0.831	0.866	0.836
3022	0.8125	0.83	0.863	0.891	0.883
7520	0.817	0.822	0.855	0.886	0.862
198	0.827	0.837	0.861	0.893	0.887
3807	0.8315	0.841	0.859	0.884	0.861
194	0.832	0.848	0.866	0.892	0.873
3293	0.8335	0.844	0.863	0.885	0.869
2070	0.8533	0.871	0.893	0.921	0.962
3	0.8568	0.875	0.897	0.931	0.918
1882	0.862	0.882	0.924	0.953	0.941
7327	0.862	0.887	0.932	0.961	0.948
12110	0.8693	0.883	0.924	0.955	0.927
8315	0.884	0.925	0.958	0.983	0.964
14842	0.8865	0.921	0.952	0.975	0.961
4840	0.888	0.928	0.956	0.986	0.966
8301	0.9	0.924	0.95	0.983	0.972
14696	0.9018	0.927	0.948	0.973	0.951
8342	0.905	0.925	0.955	0.984	0.974
2171	0.905	0.927	0.958	0.981	0.973
9032	0.908	0.923	0.951	0.976	0.965
2485	0.9193	0.928	0.966	0.993	0.98
14881	0.9263	0.935	0.962	0.985	0.968
3557	0.9313	0.946	0.977	0.993	0.984
4020	0.957	0.967	0.984	1.021	0.992
4289	0.9628	0.973	0.983	1.028	0.995
4238	0.963	0.968	0.988	1.025	0.985
12998	0.9638	0.977	0.991	1.056	0.998
3248	0.9693	0.976	1.019	1.103	1.03
11	0.97	0.985	1.119	1.137	1.11
10915	0.972	0.979	1.103	1.134	1.11
3830	0.9758	0.982	1.111	1.138	1.118
9517	1.005	1.109	1.139	1.165	1.153
2717	1.012	1.11	1.148	1.172	1.166
9972	1.026	1.172	1.208	1.286	1.221
15724	1.042	1.165	1.197	1.254	1.205
8571	1.058	1.177	1.213	1.279	1.243
1318	1.085	1.18	1.224	1.281	1.267

Supplementary Data 3: Effect of salt concentration (sodium chloride) on biofilm production

2825	1.091	1.169	1.217	1.273	1.252
12084	1.119	1.192	1.269	1.334	1.286
10046	1.143	1.187	1.254	1.331	1.273
13423	1.147	1.196	1.273	1.358	1.295
1313	1.156	1.191	1.264	1.347	1.304
3160	1.156	1.203	1.281	1.366	1.332
4059	1.157	1.2	1.258	1.361	1.342
6752	1.163	1.233	1.284	1.355	1.336
4073	1.169	1.231	1.281	1.37	1.346
14944	1.185	1.25	1.292	1.386	1.344
14735	1.186	1.258	1.287	1.392	1.357
2164	1.187	1.246	1.283	1.377	1.351
2700	1.195	1.263	1.295	1.395	1.376
10723	1.216	1.282	1.347	1.402	1.391
3398	1.225	1.276	1.354	1.389	1.371
1210	1.226	1.2803	1.351	1.391	1.367
212	1.24	1.291	1.373	1.407	1.39
10825	1.25	1.296	1.394	1.5	1.434
3724	1.269	1.305	1.41	1.544	1.496
3652	1.279	1.295	1.391	1.52	1.483
201	1.279	1.302	1.413	1.56	1.49
7884	1.282	1.307	1.425	1.554	1.502
15718	1.298	1.314	1.433	1.621	1.523
14513	1.311	1.341	1.427	1.614	1.566
6585	1.329	1.355	1.454	1.627	1.59
15521	1.33	1.39	1.51	1.66	1.578
2503	1.339	1.367	1.449	1.589	1.571
1309	1.341	1.382	1.468	1.582	1.566
2173	1.351	1.389	1.529	1.601	1.587
3709	1.371	1.403	1.554	1.634	1.613
2498	1.392	1.431	1.59	1.662	1.647
1834	1.398	1.442	1.62	1.668	1.638
10807	1.408	1.468	1.634	1.679	1.655
195	1.43	1.492	1.652	1.683	1.671
13657	1.454	1.502	1.648	1.688	1.659
4299	1.488	1.521	1.657	1.675	1.662
8436	1.51	1.564	1.678	1.693	1.677
10101	1.525	1.582	1.691	1.722	1.703
13797	1.525	1.578	1.685	1.711	1.694
13059	1.543	1.603	1.694	1.756	1.699
13603	1.553	1.612	1.683	1.759	1.704
14478	1.565	1.62	1.691	1.763	1.715
1338	1.571	1.608	1.657	1.725	1.693

Supplementary Data 3: Effect of salt concentration (sodium chloride) on biofilm production

3362	1.585	1.625	1.668	1.736	1.704
2588	1.59	1.617	1.673	1.755	1.712
12497	1.597	1.622	1.693	1.766	1.694
197	1.602	1.63	1.682	1.745	1.721
2056(PA)	1.632	1.652	1.698	1.783	1.716
13306	1.641	1.682	1.708	1.795	1.721
Sim	1.655	1.677	1.713	1.812	1.752
14068	1.674	1.691	1.724	1.832	1.764
1922 R	1.675	1.685	1.715	1.824	1.735
1240	1.682	1.697	1.722	1.817	1.783
12566	1.693	1.723	1.766	1.885	1.816
12617	1.752	1.812	1.873	1.921	1.892
2187	1.756	1.783	1.825	1.893	1.866
10872	1.757	1.82	1.884	1.915	1.892
3415	1.758	1.812	1.857	1.896	1.871
4227	1.762	1.828	1.866	1.902	1.885
14350	1.775	1.864	1.892	1.956	1.912
3593	1.802	1.841	1.877	1.912	1.893
3690	1.811	1.856	1.891	1.945	1.921
2010	1.842	1.891	1.934	1.966	1.939
14345	1.864	1.911	1.956	2.051	1.969
13962	1.864	1.907	1.949	2.132	1.915
10088	1.874	1.915	1.961	2.117	1.893
14203	1.886	1.921	1.958	2.122	1.976
6708	1.893	1.916	1.952	2.105	1.953
10012	1.903	2.12	2.358	2.547	2.054
2086	1.985	2.107	2.324	2.531	2.317
15603	2.011	2.268	2.422	2.685	2.154
2138	2.089	2.155	2.366	2.593	2.357
1337	2.099	2.213	2.391	2.584	2.402
145	2.186	2.266	2.413	2.677	2.535
2895	2.254	2.381	2.524	2.713	2.51
12600	2.261	2.424	2.635	2.754	2.427
2196	2.333	2.354	2.576	2.688	2.466
2931	2.378	2.411	2.537	2.693	2.521
15598	2.576	2.825	3.125	3.364	2.869
10894	2.623	2.867	3.268	3.533	2.983
2844	2.672	2.816	3.069	3.315	3.087
196	2.804	2.954	3.137	3.426	3.154
497	2.939	3.106	3.354	3.562	3.284
1739	3.118	3.152	3.258	3.577	3.259
2887	3.423	3.632	3.886	3.913	3.552
10869	3.667	3.89	3.956	4.567	3.983

Supplementary Data 3: Effect of salt concentration (sodium chloride) on biofilm production

3420	3.689	3.914	3.987	4.324	4.065

Supplementary Data 4: Effect of glucose concentration on biofilm formation

Isolates	BHI (W/-)	Glucose 2%	Glucose 4%	Glucose 8%	Glucose 10%
222	0.145	0.187	0.195	0.296	0.293
989	0.164	0.183	0.201	0.292	0.285
1606	0.173	0.197	0.265	0.315	0.298
1249	0.186	0.206	0.295	0.331	0.32
997	0.198	0.227	0.298	0.346	0.345
1250	0.2	0.254	0.308	0.354	0.355
1929	0.201	0.259	0.299	0.345	0.341
3179	0.2168	0.262	0.312	0.359	0.354
1252	0.221	0.269	0.331	0.366	0.365
1749	0.224	0.272	0.339	0.375	0.373
15288	0.236	0.279	0.346	0.381	0.379
2067	0.236	0.275	0.345	0.378	0.373
1996	0.237	0.284	0.368	0.387	0.385
1220	0.243	0.289	0.369	0.385	0.38
215	0.2458	0.286	0.372	0.388	0.383
6160	0.249	0.295	0.389	0.421	0.397
216	0.2518	0.302	0.395	0.437	0.432
7834	0.259	0.313	0.398	0.439	0.436
2276	0.2698	0.321	0.396	0.435	0.429
4158	0.2715	0.323	0.392	0.433	0.424
8373	0.284	0.326	0.399	0.442	0.431
217	0.293	0.332	0.411	0.45	0.45
4148	0.2963	0.33	0.415	0.453	0.45
7772	0.299	0.326	0.408	0.448	0.442
214	0.2995	0.335	0.418	0.457	0.455
3169	0.3028	0.341	0.422	0.452	0.448
11569	0.3048	0.339	0.437	0.455	0.452
1194	0.306	0.348	0.442	0.477	0.462
2232	0.314	0.343	0.345	0.481	0.473
1607	0.318	0.346	0.351	0.474	0.47
1287	0.319	0.345	0.357	0.486	0.482
2001	0.338	0.351	0.368	0.492	0.49
6211	0.348	0.357	0.369	0.489	0.488
191	0.348	0.355	0.372	0.487	0.458
1074	0.352	0.363	0.385	0.494	0.463
1931	0.353	0.359	0.392	0.498	0.483
8678	0.357	0.371	0.406	0.513	0.497
8	0.367	0.378	0.419	0.522	0.508
213	0.3713	0.388	0.423	0.535	0.524
**Isolates**	**BHI (W/-)**	**Glucose 2%**	**Glucose 4%**	**Glucose 8%**	**Glucose 10%**

Supplementary Data 4: Effect of glucose concentration on biofilm formation

14122	0.3723	0.385	0.434	0.542	0.521
4079	0.3728	0.38	0.428	0.539	0.53
2989	0.373	0.388	0.431	0.545	0.535
5993	0.374	0.386	0.435	0.541	0.537
15311	0.3818	0.394	0.442	0.549	0.545
4008	0.392	0.411	0.448	0.553	0.55
12	0.394	0.417	0.445	0.551	0.55
6122	0.399	0.422	0.449	0.556	0.553
4	0.4	0.443	0.463	0.554	0.551
4151	0.4105	0.446	0.458	0.557	0.554
11204	0.4193	0.445	0.457	0.556	0.553
210	0.4208	0.449	0.466	0.551	0.485
1876	0.424	0.456	0.481	0.558	0.493
2322	0.433	0.455	0.473	0.565	0.551
15255	0.4335	0.462	0.484	0.577	0.56
11023	0.4373	0.468	0.482	0.583	0.564
2310	0.4383	0.471	0.492	0.579	0.561
4073	0.4428	0.475	0.496	0.582	0.563
6617	0.445	0.478	0.509	0.587	0.571
8547	0.455	0.475	0.511	0.584	0.566
6	0.4565	0.474	0.513	0.572	0.553
7	0.4628	0.481	0.52	0.581	0.562
3990	0.47	0.489	0.528	0.586	0.573
2066	0.472	0.495	0.537	0.591	0.583
3303	0.472	0.497	0.533	0.587	0.58
10533	0.473	0.491	0.539	0.593	0.586
4272	0.4755	0.494	0.532	0.595	0.582
5	0.476	0.493	0.535	0.592	0.59
1886	0.477	0.511	0.542	0.591	0.581
3977	0.4843	0.52	0.553	0.597	0.588
2187	0.4865	0.518	0.551	0.588	0.576
10697	0.487	0.521	0.554	0.592	0.588
209	0.4873	0.522	0.558	0.591	0.589
4150	0.4913	0.533	0.569	0.595	0.591
3707	0.496	0.539	0.568	0.598	0.593
1214	0.499	0.543	0.565	0.596	0.592
3788	0.4998	0.545	0.569	0.597	0.593
192	0.501	0.549	0.576	0.605	0.59
7663	0.503	0.55	0.582	0.613	0.601
**Isolates**	**BHI (W/-)**	**Glucose 2%**	**Glucose 4%**	**Glucose 8%**	**Glucose 10%**
1008	0.505	0.543	0.588	0.608	0.6
13738	0.511	0.557	0.593	0.618	0.608

Supplementary Data 4: Effect of glucose concentration on biofilm formation

4179	0.516	0.554	0.591	0.614	0.603
11111	0.5238	0.568	0.607	0.623	0.606
11095	0.539	0.572	0.614	0.629	0.605
13328	0.548	0.574	0.626	0.637	0.613
13409	0.5685	0.593	0.638	0.654	0.618
1922(F)	0.573	0.598	0.642	0.671	0.63
2296	0.5735	0.595	0.644	0.682	0.638
2263	0.574	0.593	0.646	0.688	0.635
4066	0.5765	0.59	0.651	0.685	0.643
6563	0.578	0.592	0.649	0.679	0.631
3906	0.58	0.613	0.665	0.689	0.652
3357	0.5855	0.619	0.669	0.682	0.654
2	0.594	0.622	0.673	0.689	0.653
13836	0.5985	0.626	0.675	0.687	0.655
9	0.6003	0.635	0.682	0.692	0.657
2359	0.6068	0.642	0.688	0.696	0.663
2359	0.6068	0.653	0.685	0.695	0.661
1609	0.624	0.657	0.693	0.703	0.672
51	0.625	0.659	0.705	0.718	0.682
190	0.628	0.644	0.669	0.75	0.253
11109	0.6333	0.649	0.672	0.783	0.253
1740	0.645	0.653	0.671	0.782	0.253
1972	0.653	0.66	0.679	0.788	0.253
3917	0.6563	0.662	0.681	0.784	0.253
1583	0.664	0.672	0.684	0.789	0.253
14514	0.6728	0.685	0.725	0.774	0.253
3869	0.6803	0.692	0.723	0.779	0.253
2965	0.6818	0.69	0.727	0.773	0.253
2972	0.686	0.695	0.724	0.781	0.253
1974	0.693	0.725	0.747	0.79	0.253
12027	0.6938	0.718	0.732	0.786	0.253
10	0.6968	0.722	0.743	0.788	0.253
11600	0.7	0.726	0.753	0.784	0.253
4268	0.7098	0.719	0.745	0.775	0.253
3294	0.712	0.733	0.759	0.786	0.253
200	0.714	0.724	0.767	0.792	0.253
2822	0.716	0.722	0.764	0.797	0.253
**Isolates**	**BHI (W/-)**	**Glucose 2%**	**Glucose 4%**	**Glucose 8%**	**Glucose 10%**
193	0.722	0.736	0.772	0.81	0.253
14310	0.7248	0.738	0.763	0.793	0.253
993	0.733	0.742	0.776	0.803	0.253
4182	0.738	0.745	0.771	0.804	0.253

Supplementary Data 4: Effect of glucose concentration on biofilm formation

1772	0.74	0.753	0.779	0.824	0.253
4067	0.7545	0.769	0.783	0.833	0.253
2204/2294	0.761	0.773	0.788	0.828	0.253
2517	0.764	0.764	0.773	0.816	0.253
2190	0.764	0.773	0.796	0.823	0.253
3811	0.7658	0.779	0.803	0.834	0.253
11040	0.766	0.782	0.814	0.846	0.253
1315	0.767	0.777	0.799	0.828	0.253
9607	0.773	0.794	0.817	0.833	0.253
7877	0.775	0.801	0.825	0.848	0.253
1	0.777	0.807	0.819	0.853	0.253
2551	0.779	0.81	0.828	0.851	0.253
14730	0.8078	0.815	0.831	0.866	0.253
3022	0.8125	0.83	0.863	0.891	0.253
7520	0.817	0.822	0.855	0.886	0.253
198	0.827	0.837	0.861	0.893	0.253
3807	0.8315	0.841	0.859	0.884	0.253
194	0.832	0.848	0.866	0.892	0.253
3293	0.8335	0.844	0.863	0.885	0.253
2070	0.8533	0.871	0.893	0.921	0.253
3	0.8568	0.875	0.897	0.931	0.253
1882	0.862	0.882	0.924	0.953	0.253
7327	0.862	0.887	0.932	0.961	0.253
12110	0.8693	0.883	0.924	0.955	0.253
8315	0.884	0.925	0.958	0.983	0.253
14842	0.8865	0.921	0.952	0.975	0.253
4840	0.888	0.928	0.956	0.986	0.253
8301	0.9	0.924	0.95	0.983	0.253
14696	0.9018	0.927	0.948	0.973	0.253
8342	0.905	0.925	0.955	0.984	0.253
2171	0.905	0.927	0.958	0.981	0.253
9032	0.908	0.923	0.951	0.976	0.253
2485	0.9193	0.928	0.966	0.993	0.253
14881	0.9263	0.935	0.962	0.985	0.253
3557	0.9313	0.946	0.977	0.993	0.253
**Isolates**	**BHI (W/-)**	**Glucose 2%**	**Glucose 4%**	**Glucose 8%**	**Glucose 10%**
4020	0.957	0.967	0.984	1.021	0.253
4289	0.9628	0.973	0.983	1.028	0.253
4238	0.963	0.968	0.988	1.025	0.253
12998	0.9638	0.977	0.991	1.056	0.253
3248	0.9693	0.976	1.019	1.103	0.253
11	0.97	0.985	1.119	1.137	0.253

Supplementary Data 4: Effect of glucose concentration on biofilm formation

10915	0.972	0.979	1.103	1.134	0.253
3830	0.9758	0.982	1.111	1.138	0.253
9517	1.005	1.109	1.139	1.165	0.253
2717	1.012	1.11	1.148	1.172	0.253
9972	1.026	1.172	1.208	1.286	0.253
15724	1.042	1.165	1.197	1.254	0.253
8571	1.058	1.177	1.213	1.279	0.253
1318	1.085	1.18	1.224	1.281	0.253
2825	1.091	1.169	1.217	1.273	0.253
12084	1.119	1.192	1.269	1.334	0.253
10046	1.143	1.187	1.254	1.331	0.253
13423	1.147	1.196	1.273	1.358	0.253
1313	1.156	1.191	1.264	1.347	0.253
3160	1.156	1.203	1.281	1.366	0.253
4059	1.157	1.2	1.258	1.361	0.253
6752	1.163	1.233	1.284	1.355	0.253
4073	1.169	1.231	1.281	1.37	0.253
14944	1.185	1.25	1.292	1.386	0.253
14735	1.186	1.258	1.287	1.392	0.253
2164	1.187	1.246	1.283	1.377	0.253
2700	1.195	1.263	1.295	1.395	0.253
10723	1.216	1.282	1.347	1.402	0.253
3398	1.225	1.276	1.354	1.389	0.253
1210	1.226	1.2803	1.351	1.391	0.253
212	1.24	1.291	1.373	1.407	0.253
10825	1.25	1.296	1.394	1.5	0.253
3724	1.269	1.305	1.41	1.544	0.253
3652	1.279	1.295	1.391	1.52	0.253
201	1.279	1.302	1.413	1.56	0.253
7884	1.282	1.307	1.425	1.554	0.253
15718	1.298	1.314	1.433	1.621	0.253
14513	1.311	1.341	1.427	1.614	0.253
6585	1.329	1.355	1.454	1.627	0.253
**Isolates**	**BHI (W/-)**	**Glucose 2%**	**Glucose 4%**	**Glucose 8%**	**Glucose 10%**
15521	1.33	1.39	1.51	1.66	0.253
2503	1.339	1.367	1.449	1.589	0.253
1309	1.341	1.382	1.468	1.582	0.253
2173	1.351	1.389	1.529	1.601	0.253
3709	1.371	1.403	1.554	1.634	0.253
2498	1.392	1.431	1.59	1.662	0.253
1834	1.398	1.442	1.62	1.668	0.253
10807	1.408	1.468	1.634	1.679	0.253

Supplementary Data 4: Effect of glucose concentration on biofilm formation

195	1.43	1.492	1.652	1.683	0.253
13657	1.454	1.502	1.648	1.688	0.253
4299	1.488	1.521	1.657	1.675	0.253
8436	1.51	1.564	1.678	1.693	0.253
10101	1.525	1.582	1.691	1.722	0.253
13797	1.525	1.578	1.685	1.711	0.253
13059	1.543	1.603	1.694	1.756	0.253
13603	1.553	1.612	1.683	1.759	0.253
14478	1.565	1.62	1.691	1.763	0.253
1338	1.571	1.608	1.657	1.725	0.253
3362	1.585	1.625	1.668	1.736	0.253
2588	1.59	1.617	1.673	1.755	0.253
12497	1.597	1.622	1.693	1.766	0.253
197	1.602	1.63	1.682	1.745	0.253
2056(PA)	1.632	1.652	1.698	1.783	0.253
13306	1.641	1.682	1.708	1.795	0.253
Sim	1.655	1.677	1.713	1.812	0.253
14068	1.674	1.691	1.724	1.832	0.253
1922 R	1.675	1.685	1.715	1.824	0.253
1240	1.682	1.697	1.722	1.817	0.253
12566	1.693	1.723	1.766	1.885	0.253
12617	1.752	1.812	1.873	1.921	0.253
2187	1.756	1.783	1.825	1.893	0.253
10872	1.757	1.82	1.884	1.915	0.253
3415	1.758	1.812	1.857	1.896	0.253
4227	1.762	1.828	1.866	1.902	0.253
14350	1.775	1.864	1.892	1.956	0.253
3593	1.802	1.841	1.877	1.912	0.253
3690	1.811	1.856	1.891	1.945	0.253
2010	1.842	1.891	1.934	1.966	0.253
14345	1.864	1.911	1.956	2.051	0.253
**Isolates**	**BHI (W/-)**	**Glucose 2%**	**Glucose 4%**	**Glucose 8%**	**Glucose 10%**
13962	1.864	1.907	1.949	2.132	0.253
10088	1.874	1.915	1.961	2.117	0.253
14203	1.886	1.921	1.958	2.122	0.253
6708	1.893	1.916	1.952	2.105	0.253
10012	1.903	2.12	2.358	2.547	0.253
2086	1.985	2.107	2.324	2.531	0.253
15603	2.011	2.268	2.422	2.685	0.253
2138	2.089	2.155	2.366	2.593	0.253
1337	2.099	2.213	2.391	2.584	0.253
145	2.186	2.266	2.413	2.677	0.253

Supplementary Data 4: Effect of glucose concentration on biofilm formation

2895	2.254	2.381	2.524	2.713	0.253
12600	2.261	2.424	2.635	2.754	0.253
2196	2.333	2.354	2.576	2.688	0.253
2931	2.378	2.411	2.537	2.693	0.253
15598	2.576	2.825	3.125	3.364	0.253
10894	2.623	2.867	3.268	3.533	0.253
2844	2.672	2.816	3.069	3.315	0.253
196	2.804	2.954	3.137	3.426	0.253
497	2.939	3.106	3.354	3.562	0.253
1739	3.118	3.152	3.258	3.577	0.253
2887	3.423	3.632	3.886	3.913	0.253
10869	3.667	3.89	3.956	4.567	0.253
3420	3.689	3.914	3.987	4.324	0.253

Supplementary Data 5: Effect of amino acids on biofilm formation

Isolates	BHI (W/-)	Met 2 mM	Met 4 mM	Met 8 mM	Met 10 mM	SAM 5 mM	SAM 10 mM	SAM 15 mM	SAM 20 mM
222	0.145	0.159	0.184	0.266	0.272	0.168	0.195	0.277	0.298
989	0.164	0.175	0.208	0.273	0.281	0.189	0.216	0.295	0.315
1606	0.173	0.186	0.231	0.285	0.294	0.195	0.269	0.316	0.327
1249	0.186	0.196	0.267	0.313	0.319	0.216	0.297	0.323	0.335
997	0.198	0.216	0.285	0.335	0.343	0.227	0.298	0.346	0.357
1250	0.2	0.223	0.294	0.334	0.339	0.231	0.303	0.344	0.354
1929	0.201	0.238	0.292	0.347	0.352	0.243	0.296	0.351	0.359
3179	0.2168	0.245	0.316	0.363	0.364	0.257	0.315	0.375	0.379
1252	0.221	0.259	0.324	0.375	0.376	0.266	0.338	0.388	0.385
1749	0.224	0.274	0.335	0.379	0.377	0.279	0.359	0.399	0.397
15288	0.236	0.285	0.346	0.383	0.38	0.291	0.373	0.411	0.405
2067	0.236	0.292	0.345	0.385	0.383	0.295	0.385	0.415	0.403
1996	0.237	0.291	0.368	0.389	0.385	0.284	0.356	0.392	0.395
1220	0.243	0.292	0.375	0.386	0.38	0.289	0.382	0.416	0.39
215	0.2458	0.285	0.369	0.398	0.395	0.295	0.383	0.406	0.401
6160	0.249	0.297	0.375	0.414	0.402	0.305	0.395	0.427	0.408
216	0.2518	0.305	0.391	0.427	0.412	0.314	0.395	0.436	0.414
7834	0.259	0.314	0.399	0.435	0.417	0.319	0.398	0.431	0.416
2276	0.2698	0.327	0.406	0.443	0.44	0.335	0.414	0.452	0.42
4158	0.2715	0.331	0.392	0.436	0.425	0.339	0.405	0.441	0.425
8373	0.284	0.338	0.411	0.446	0.435	0.347	0.416	0.449	0.429
217	0.293	0.342	0.415	0.445	0.431	0.345	0.422	0.45	0.427
4148	0.2963	0.335	0.408	0.441	0.422	0.342	0.429	0.458	0.432
7772	0.299	0.332	0.403	0.437	0.418	0.347	0.425	0.453	0.425
214	0.2995	0.329	0.416	0.448	0.421	0.335	0.425	0.459	0.432
3169	0.3028	0.336	0.425	0.447	0.423	0.345	0.436	0.457	0.425
11569	0.3048	0.349	0.434	0.452	0.428	0.352	0.442	0.465	0.424
1194	0.306	0.352	0.447	0.479	0.433	0.363	0.456	0.489	0.435
2232	0.314	0.344	0.435	0.483	0.43	0.349	0.445	0.496	0.431
1607	0.318	0.342	0.439	0.489	0.437	0.355	0.357	0.498	0.448
1287	0.319	0.341	0.443	0.485	0.435	0.349	0.458	0.494	0.445
2001	0.338	0.348	0.453	0.494	0.439	0.357	0.468	0.504	0.453
6211	0.348	0.359	0.469	0.507	0.445	0.365	0.478	0.517	0.458
191	0.348	0.365	0.476	0.513	0.448	0.375	0.483	0.524	0.458
1074	0.352	0.369	0.484	0.522	0.453	0.376	0.495	0.537	0.461
1931	0.353	0.354	0.482	0.517	0.451	0.367	0.489	0.535	0.465
8678	0.357	0.367	0.429	0.497	0.425	0.381	0.456	0.529	0.466
8	0.367	0.371	0.433	0.512	0.438	0.398	0.468	0.532	0.468
213	0.3713	0.389	0.445	0.538	0.445	0.397	0.458	0.553	0.472
Isolates	BHI (W/-)	Met 2 mM	Met 4 mM	Met 8 mM	Met 10 mM	SAM 5 mM	SAM 10 mM	SAM 15 mM	SAM 20 mM

14122	0.3723	0.382	0.441	0.525	0.431	0.395	0.472	0.545	0.475
4079	0.3728	0.385	0.438	0.529	0.43	0.393	0.457	0.543	0.48
2989	0.373	0.389	0.437	0.537	0.435	0.395	0.454	0.547	0.485
5993	0.374	0.395	0.444	0.547	0.441	0.398	0.468	0.557	0.488
15311	0.3818	0.393	0.446	0.543	0.445	0.397	0.465	0.552	0.485
4008	0.392	0.412	0.445	0.538	0.452	0.419	0.468	0.558	0.485
12	0.394	0.416	0.443	0.548	0.455	0.425	0.463	0.564	0.489
6122	0.399	0.425	0.452	0.558	0.457	0.432	0.465	0.567	0.483
4	0.4	0.437	0.461	0.561	0.452	0.455	0.476	0.583	0.485
4151	0.4105	0.442	0.465	0.567	0.452	0.453	0.465	0.579	0.482
11204	0.4193	0.447	0.459	0.563	0.455	0.459	0.472	0.575	0.485
210	0.4208	0.451	0.468	0.562	0.461	0.465	0.479	0.577	0.493
1876	0.424	0.455	0.475	0.568	0.465	0.468	0.486	0.582	0.491
2322	0.433	0.453	0.471	0.558	0.455	0.464	0.481	0.577	0.486
15255	0.4335	0.459	0.478	0.573	0.463	0.467	0.489	0.586	0.49
11023	0.4373	0.465	0.481	0.582	0.465	0.478	0.495	0.593	0.495
2310	0.4383	0.472	0.493	0.578	0.468	0.483	0.511	0.586	0.492
4073	0.4428	0.475	0.498	0.585	0.471	0.485	0.518	0.596	0.495
6617	0.445	0.479	0.508	0.593	0.475	0.486	0.524	0.617	0.501
8547	0.455	0.483	0.518	0.596	0.478	0.493	0.529	0.617	0.505
6	0.4565	0.487	0.515	0.592	0.475	0.494	0.525	0.613	0.51
7	0.4628	0.485	0.522	0.588	0.482	0.492	0.527	0.598	0.515
3990	0.47	0.488	0.529	0.585	0.485	0.497	0.531	0.608	0.514
2066	0.472	0.492	0.535	0.593	0.487	0.498	0.545	0.613	0.517
3303	0.472	0.498	0.538	0.595	0.485	0.508	0.551	0.618	0.515
10533	0.473	0.495	0.537	0.588	0.491	0.498	0.549	0.597	0.506
4272	0.4755	0.497	0.539	0.592	0.485	0.505	0.545	0.608	0.511
5	0.476	0.492	0.533	0.585	0.488	0.508	0.549	0.615	0.515
1886	0.477	0.515	0.547	0.595	0.492	0.527	0.559	0.612	0.519
3977	0.4843	0.521	0.552	0.598	0.495	0.532	0.565	0.619	0.515
2187	0.4865	0.517	0.555	0.593	0.499	0.528	0.568	0.611	0.521
10697	0.487	0.523	0.559	0.598	0.502	0.532	0.577	0.623	0.525
209	0.4873	0.519	0.547	0.585	0.493	0.535	0.565	0.612	0.518
4150	0.4913	0.528	0.558	0.597	0.501	0.539	0.576	0.619	0.524
3707	0.496	0.535	0.565	0.595	0.505	0.542	0.585	0.625	0.529
1214	0.499	0.546	0.568	0.592	0.511	0.554	0.576	0.613	0.532
3788	0.4998	0.549	0.575	0.605	0.515	0.558	0.585	0.624	0.537
192	0.501	0.553	0.579	0.604	0.517	0.563	0.587	0.619	0.535
7663	0.503	0.559	0.588	0.615	0.521	0.567	0.596	0.628	0.542
**Isolates**	**BHI (W/-)**	**Met 2 mM**	**Met 4 mM**	**Met 8 mM**	**Met 10 mM**	**SAM 5 mM**	**SAM 10 mM**	**SAM 15 mM**	**SAM 20 mM**
1008	0.505	0.547	0.575	0.604	0.516	0.561	0.589	0.622	0.546
13738	0.511	0.552	0.582	0.613	0.511	0.569	0.598	0.625	0.551

Supplementary Data 5: Effect of amino acids on biofilm formation

4179	0.516	0.562	0.593	0.615	0.515	0.578	0.605	0.628	0.554
11111	0.5238	0.569	0.605	0.628	0.521	0.585	0.613	0.638	0.557
11095	0.539	0.575	0.619	0.637	0.528	0.588	0.628	0.648	0.56
13328	0.548	0.579	0.629	0.635	0.525	0.592	0.642	0.653	0.565
13409	0.5685	0.595	0.639	0.658	0.528	0.604	0.653	0.676	0.568
1922(F)	0.573	0.592	0.637	0.661	0.53	0.605	0.651	0.678	0.572
2296	0.5735	0.598	0.645	0.678	0.538	0.611	0.659	0.689	0.575
2263	0.574	0.585	0.627	0.669	0.526	0.599	0.645	0.683	0.569
4066	0.5765	0.591	0.641	0.673	0.532	0.607	0.664	0.697	0.585
6563	0.578	0.595	0.665	0.685	0.535	0.613	0.683	0.705	0.577
3906	0.58	0.615	0.665	0.681	0.538	0.626	0.689	0.709	0.579
3357	0.5855	0.607	0.654	0.673	0.531	0.617	0.672	0.685	0.585
2	0.594	0.621	0.675	0.693	0.536	0.632	0.685	0.712	0.584
13836	0.5985	0.625	0.672	0.696	0.539	0.637	0.689	0.706	0.587
9	0.6003	0.633	0.685	0.707	0.545	0.646	0.696	0.718	0.593
2359	0.6068	0.638	0.675	0.698	0.541	0.649	0.688	0.702	0.595
2359	0.6068	0.644	0.675	0.695	0.545	0.659	0.692	0.705	0.602
1609	0.624	0.652	0.682	0.693	0.548	0.665	0.693	0.712	0.605
51	0.625	0.658	0.697	0.716	0.552	0.673	0.712	0.729	0.612
190	0.628	0.661	0.715	0.738	0.564	0.674	0.727	0.755	0.619
11109	0.6333	0.667	0.726	0.747	0.573	0.676	0.737	0.762	0.626
1740	0.645	0.669	0.728	0.753	0.575	0.682	0.744	0.765	0.625
1972	0.653	0.675	0.731	0.758	0.575	0.687	0.748	0.772	0.628
3917	0.6563	0.671	0.725	0.744	0.578	0.688	0.739	0.779	0.632
1583	0.664	0.673	0.734	0.759	0.585	0.691	0.755	0.786	0.63
14514	0.6728	0.682	0.741	0.773	0.592	0.695	0.755	0.791	0.637
3869	0.6803	0.687	0.747	0.788	0.598	0.707	0.762	0.805	0.641
2965	0.6818	0.695	0.756	0.792	0.608	0.715	0.784	0.826	0.645
2972	0.686	0.708	0.779	0.826	0.605	0.718	0.786	0.835	0.642
1974	0.693	0.724	0.783	0.832	0.603	0.736	0.795	0.842	0.648
12027	0.6938	0.725	0.789	0.839	0.605	0.734	0.806	0.851	0.652
10	0.6968	0.722	0.785	0.845	0.607	0.736	0.809	0.847	0.658
11600	0.7	0.728	0.792	0.858	0.609	0.737	0.814	0.867	0.663
4268	0.7098	0.726	0.785	0.853	0.614	0.735	0.807	0.855	0.665
3294	0.712	0.733	0.792	0.874	0.612	0.744	0.796	0.888	0.668
200	0.714	0.737	0.789	0.877	0.615	0.748	0.795	0.893	0.673
2822	0.716	0.735	0.781	0.882	0.615	0.745	0.798	0.895	0.677
**Isolates**	**BHI (W/-)**	**Met 2%**	**Met 4%**	**Met 8%**	**Met 10%**	**SAM 2%**	**SAM 4%**	**SAM 8%**	**SAM 10%**
193	0.722	0.744	0.795	0.887	0.618	0.749	0.798	0.895	0.68
14310	0.7248	0.742	0.787	0.885	0.62	0.748	0.795	0.907	0.685
993	0.733	0.753	0.797	0.897	0.624	0.758	0.807	0.915	0.688
4182	0.738	0.755	0.794	0.895	0.625	0.762	0.811	0.924	0.686

Supplementary Data 5: Effect of amino acids on biofilm formation

1772	0.74	0.761	0.805	0.907	0.628	0.772	0.819	0.928	0.683
4067	0.7545	0.768	0.821	0.92	0.636	0.782	0.835	0.932	0.689
2204/2294	0.761	0.782	0.843	0.935	0.642	0.793	0.855	0.941	0.695
2517	0.764	0.786	0.841	0.933	0.645	0.795	0.853	0.938	0.697
2190	0.764	0.789	0.845	0.938	0.642	0.797	0.855	0.943	0.695
3811	0.7658	0.785	0.842	0.932	0.639	0.802	0.856	0.947	0.704
11040	0.766	0.789	0.848	0.937	0.646	0.798	0.859	0.955	0.702
1315	0.767	0.787	0.851	0.943	0.648	0.808	0.863	0.959	0.705
9607	0.773	0.793	0.855	0.945	0.645	0.813	0.865	0.963	0.705
7877	0.775	0.792	0.859	0.948	0.652	0.807	0.862	0.961	0.707
1	0.777	0.804	0.865	0.951	0.655	0.815	0.876	0.968	0.712
2551	0.779	0.811	0.872	0.958	0.658	0.824	0.881	0.967	0.715
14730	0.8078	0.815	0.875	0.965	0.662	0.827	0.885	0.978	0.718
3022	0.8125	0.822	0.873	0.962	0.661	0.834	0.886	0.974	0.714
7520	0.817	0.829	0.876	0.969	0.665	0.838	0.887	0.983	0.716
198	0.827	0.837	0.885	0.974	0.668	0.851	0.897	0.989	0.715
3807	0.8315	0.848	0.893	0.987	0.673	0.855	0.906	0.998	0.717
194	0.832	0.851	0.889	0.975	0.675	0.863	0.913	1.095	0.716
3293	0.8335	0.858	0.892	0.981	0.672	0.867	0.911	0.996	0.715
2070	0.8533	0.872	0.898	0.996	0.675	0.889	0.918	1.124	0.718
3	0.8568	0.879	0.907	0.993	0.67	0.893	0.925	1.12	0.72
1882	0.862	0.885	0.915	0.997	0.674	0.895	0.931	1.128	0.725
7327	0.862	0.881	0.913	0.995	0.677	0.893	0.929	1.115	0.728
12110	0.8693	0.887	0.919	0.997	0.681	0.898	0.927	1.119	0.732
8315	0.884	0.902	0.935	1.049	0.705	0.915	0.946	1.077	0.754
14842	0.8865	0.908	0.934	0.998	0.696	0.922	0.953	1.128	0.755
4840	0.888	0.918	0.939	1.037	0.702	0.926	0.955	1.125	0.746
8301	0.9	0.927	0.946	1.062	0.705	0.938	0.963	1.128	0.751
14696	0.9018	0.925	0.953	1.108	0.708	0.938	0.976	1.125	0.757
8342	0.905	0.931	0.965	1.115	0.713	0.947	0.978	1.129	0.761
2171	0.905	0.929	0.963	1.108	0.703	0.937	0.975	1.118	0.755
9032	0.908	0.933	0.968	1.119	0.706	0.941	0.981	1.127	0.757
2485	0.9193	0.939	0.973	1.127	0.712	0.948	0.985	1.136	0.766
14881	0.9263	0.945	0.975	1.136	0.718	0.955	0.983	1.146	0.768
3557	0.9313	0.951	0.979	1.141	0.725	0.961	0.985	1.153	0.772
**Isolates**	**BHI (W/-)**	**Met 2 mM**	**Met 4 mM**	**Met 8 mM**	**Met 10 mM**	**SAM 5 mM**	**SAM 10 mM**	**SAM 15 mM**	**SAM 20 mM**
4020	0.957	0.959	0.985	1.159	0.729	0.967	0.992	1.173	0.783
4289	0.9628	0.967	0.997	1.195	0.737	0.978	1.016	1.221	0.788
4238	0.963	0.976	1.027	1.229	0.746	0.987	1.048	1.238	0.795
12998	0.9638	0.985	1.086	1.255	0.755	0.995	1.127	1.363	0.807
3248	0.9693	0.983	1.093	1.226	0.748	0.994	1.118	1.267	0.787
11	0.97	0.987	1.123	1.248	0.755	0.996	1.135	1.377	0.813

10915	0.972	0.985	1.125	1.238	0.745	0.998	1.131	1.369	0.796
3830	0.9758	0.991	1.119	1.228	0.752	1.056	1.138	1.384	0.823
9517	1.005	1.107	1.129	1.241	0.758	1.119	1.146	1.378	0.829
2717	1.012	1.118	1.135	1.252	0.762	1.132	1.149	1.388	0.837
9972	1.026	1.129	1.149	1.263	0.775	1.143	1.158	1.381	0.835
15724	1.042	1.138	1.156	1.271	0.782	1.155	1.165	1.385	0.838
8571	1.058	1.145	1.166	1.282	0.785	1.167	1.179	1.394	0.842
1318	1.085	1.144	1.175	1.294	0.787	1.163	1.186	1.391	0.845
2825	1.091	1.149	1.175	1.298	0.791	1.172	1.189	1.395	0.843
12084	1.119	1.175	1.208	1.317	0.798	1.194	1.245	1.384	0.848
10046	1.143	1.189	1.218	1.326	0.805	1.217	1.265	1.396	0.849
13423	1.147	1.196	1.238	1.347	0.812	1.215	1.269	1.388	0.853
1313	1.156	1.208	1.247	1.358	0.821	1.227	1.282	1.395	0.864
3160	1.156	1.215	1.244	1.365	0.825	1.236	1.289	1.392	0.867
4059	1.157	1.213	1.245	1.365	0.829	1.243	1.285	1.387	0.865
6752	1.163	1.224	1.252	1.368	0.83	1.239	1.278	1.377	0.868
4073	1.169	1.233	1.255	1.371	0.832	1.245	1.274	1.389	0.874
14944	1.185	1.247	1.266	1.382	0.835	1.261	1.28	1.395	0.871
14735	1.186	1.251	1.265	1.378	0.833	1.265	1.288	1.389	0.879
2164	1.187	1.249	1.269	1.383	0.837	1.262	1.285	1.392	0.883
2700	1.195	1.257	1.285	1.396	0.844	1.274	1.304	1.411	0.887
10723	1.216	1.281	1.318	1.408	0.851	1.296	1.341	1.437	0.896
3398	1.225	1.289	1.331	1.425	0.863	1.308	1.353	1.449	0.908
1210	1.226	1.285	1.335	1.432	0.865	1.302	1.355	1.452	0.913
212	1.24	1.292	1.347	1.446	0.871	1.314	1.363	1.461	0.918
10825	1.25	1.304	1.355	1.452	0.875	1.311	1.372	1.468	0.915
3724	1.269	1.315	1.373	1.471	0.882	1.325	1.398	1.492	0.919
3652	1.279	1.322	1.385	1.487	0.887	1.338	1.413	1.515	0.925
201	1.279	1.318	1.377	1.481	0.883	1.331	1.405	1.507	0.928
7884	1.282	1.327	1.392	1.495	0.889	1.339	1.415	1.514	0.933
15718	1.298	1.335	1.404	1.508	0.893	1.347	1.421	1.523	0.939
14513	1.311	1.333	1.411	1.517	0.895	1.345	1.437	1.529	0.944
6585	1.329	1.342	1.417	1.525	0.904	1.353	1.444	1.538	0.948
**Isolates**	**BHI (W/-)**	**Met 2 mM**	**Met 4 mM**	**Met 8 mM**	**Met 10 mM**	**SAM 5 mM**	**SAM 10 mM**	**SAM 15 mM**	**SAM 20 mM**
15521	1.33	1.346	1.428	1.538	0.905	1.359	1.468	1.559	0.952
2503	1.339	1.349	1.433	1.559	0.902	1.365	1.475	1.576	0.957
1309	1.341	1.358	1.451	1.575	0.908	1.384	1.493	1.593	0.963
2173	1.351	1.372	1.473	1.588	0.912	1.398	1.524	1.615	0.975
3709	1.371	1.404	1.498	1.613	0.919	1.421	1.537	1.629	0.986
2498	1.392	1.428	1.538	1.633	0.922	1.443	1.568	1.648	0.995
1834	1.398	1.435	1.535	1.646	0.928	1.448	1.583	1.663	1.057
10807	1.408	1.443	1.574	1.655	0.934	1.466	1.612	1.703	1.115

Supplementary Data 5: Effect of amino acids on biofilm formation

195	1.43	1.475	1.644	1.687	0.939	1.488	1.677	1.716	1.119
13657	1.454	1.483	1.667	1.711	0.943	1.497	1.686	1.735	1.126
4299	1.488	1.516	1.683	1.735	0.948	1.529	1.699	1.754	1.133
8436	1.51	1.538	1.694	1.752	0.955	1.558	1.718	1.773	1.138
10101	1.525	1.554	1.718	1.759	0.961	1.575	1.726	1.788	1.145
13797	1.525	1.551	1.723	1.755	0.96	1.577	1.732	1.785	1.145
13059	1.543	1.584	1.757	1.789	0.976	1.598	1.776	1.803	1.152
13603	1.553	1.595	1.773	1.796	0.988	1.614	1.796	1.818	1.155
14478	1.565	1.614	1.788	1.815	0.992	1.632	1.805	1.827	1.162
1338	1.571	1.625	1.798	1.828	0.998	1.644	1.812	1.849	1.169
3362	1.585	1.633	1.815	1.843	1.051	1.65	1.826	1.857	1.176
2588	1.59	1.647	1.829	1.858	1.058	1.662	1.839	1.869	1.182
12497	1.597	1.653	1.831	1.867	1.061	1.668	1.843	1.875	1.187
197	1.602	1.675	1.846	1.878	1.068	1.685	1.855	1.887	1.189
13306	1.635	1.692	1.861	1.897	1.075	1.712	1.875	1.912	1.197
Sim	1.655	1.698	1.877	1.912	1.082	1.713	1.888	1.925	1.204
14068	1.674	1.708	1.875	1.908	1.085	1.719	1.897	1.924	1.205
1922 R	1.675	1.705	1.871	1.905	1.085	1.717	1.903	1.921	1.203
1240	1.689	1.723	1.894	1.924	1.104	1.738	1.923	1.942	1.213
12566	1.693	1.738	1.922	1.947	1.107	1.751	1.942	1.963	1.218
12617	1.752	1.829	1.957	1.975	1.116	1.839	1.973	1.995	1.254
2187	1.756	1.825	1.955	1.971	1.107	1.837	1.975	1.991	1.251
10872	1.757	1.837	1.967	1.988	1.115	1.848	1.987	2.063	1.255
3415	1.758	1.835	1.965	1.985	1.113	1.847	1.985	2.069	1.258
4227	1.762	1.843	1.983	1.987	1.115	1.855	1.992	2.105	1.263
14350	1.775	1.855	1.996	2.056	1.119	1.872	2.107	2.236	1.268
3593	1.802	1.916	2.165	2.238	1.215	1.928	2.182	2.255	1.315
3690	1.811	1.921	2.179	2.247	1.223	1.933	2.194	2.269	1.311
2010	1.842	1.941	2.232	2.353	1.238	1.955	2.258	2.374	1.324
14345	1.864	1.944	2.255	2.378	1.246	1.959	2.296	2.393	1.328
13962	1.864	1.945	2.261	2.375	1.248	1.957	2.311	2.398	1.325
**Isolates**	**BHI (W/-)**	**Met 2 mM**	**Met 4 mM**	**Met 8 mM**	**Met 10 mM**	**SAM 5 mM**	**SAM 10 mM**	**SAM 15 mM**	**SAM 20 mM**
10088	1.874	1.957	2.318	2.517	1.267	1.972	2.385	2.577	1.368
14203	1.886	1.965	2.327	2.534	1.265	1.983	2.394	2.594	1.375
6708	1.893	1.965	2.354	2.551	1.318	1.984	2.408	2.613	1.387
10012	1.903	1.974	2.355	2.568	1.329	1.995	2.428	2.645	1.391
2086	1.985	1.993	2.427	2.666	1.332	2.105	2.516	2.689	1.405
15603	2.011	2.115	2.494	2.687	1.335	2.227	2.611	2.746	1.416
2138	2.089	2.132	2.512	2.694	1.338	2.232	2.624	2.831	1.422
1337	2.099	2.176	2.562	2.724	1.355	2.264	2.658	2.847	1.431
145	2.186	2.234	2.642	2.873	1.358	2.377	2.751	2.95	1.438
2895	2.254	2.276	2.735	2.947	1.381	2.414	2.864	2.978	1.445

Supplementary Data 5: Effect of amino acids on biofilm formation

12600	2.261	2.284	2.796	2.977	1.385	2.428	2.877	2.983	1.465
2196	2.333	2.389	2.824	2.985	1.394	2.519	2.891	2.996	1.474
2931	2.378	2.443	2.965	3.138	1.412	2.531	2.983	3.224	1.475
15598	2.576	2.655	3.137	3.452	1.456	2.758	3.188	3.527	1.482
10894	2.623	2.755	3.184	3.514	1.472	2.866	3.242	3.551	1.488
2844	2.672	2.813	3.189	3.528	1.481	2.857	3.241	3.498	1.493
196	2.804	3.061	3.354	3.671	1.489	3.247	3.381	3.776	1.511
497	2.939	3.175	3.379	3.658	1.506	3.239	3.389	3.775	1.515
1739	3.118	3.241	3.458	3.818	1.511	3.328	3.535	3.942	1.519
2887	3.423	3.448	3.765	3.855	1.522	3.524	3.814	3.877	1.529
10869	3.667	3.695	3.922	4.324	1.53	3.754	3.961	4.571	1.538
3420	3.689	3.822	3.975	4.533	1.542	3.859	3.997	4.562	1.547

Supplementary Data 6: Fourier transform infrared spectroscopy

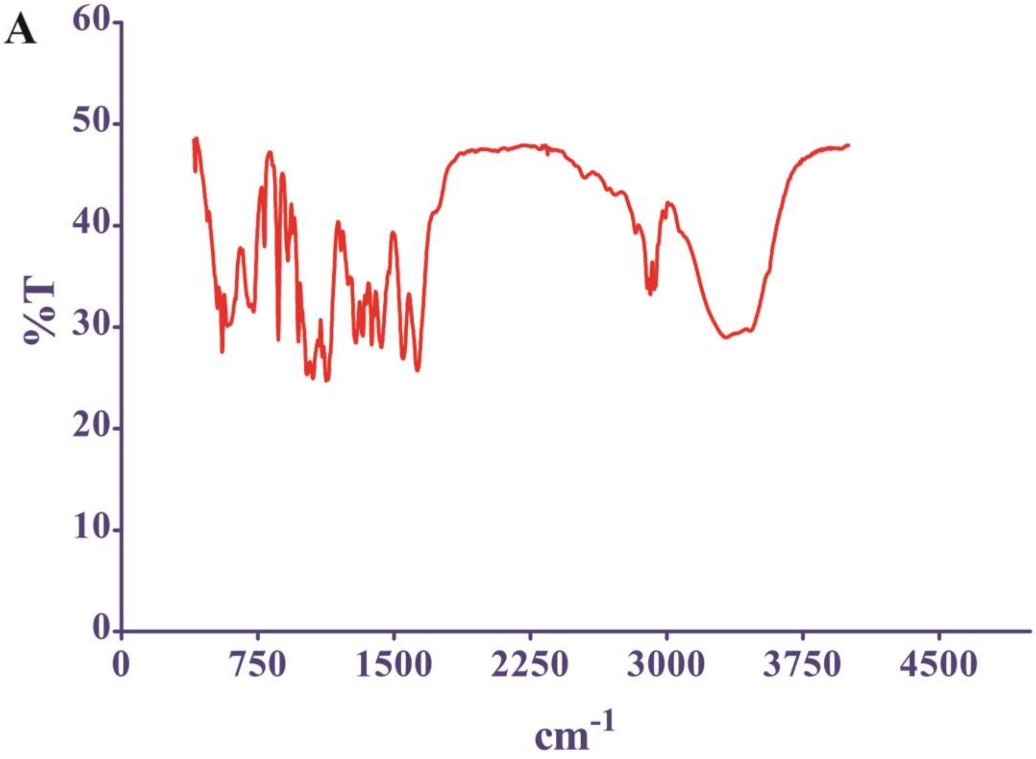

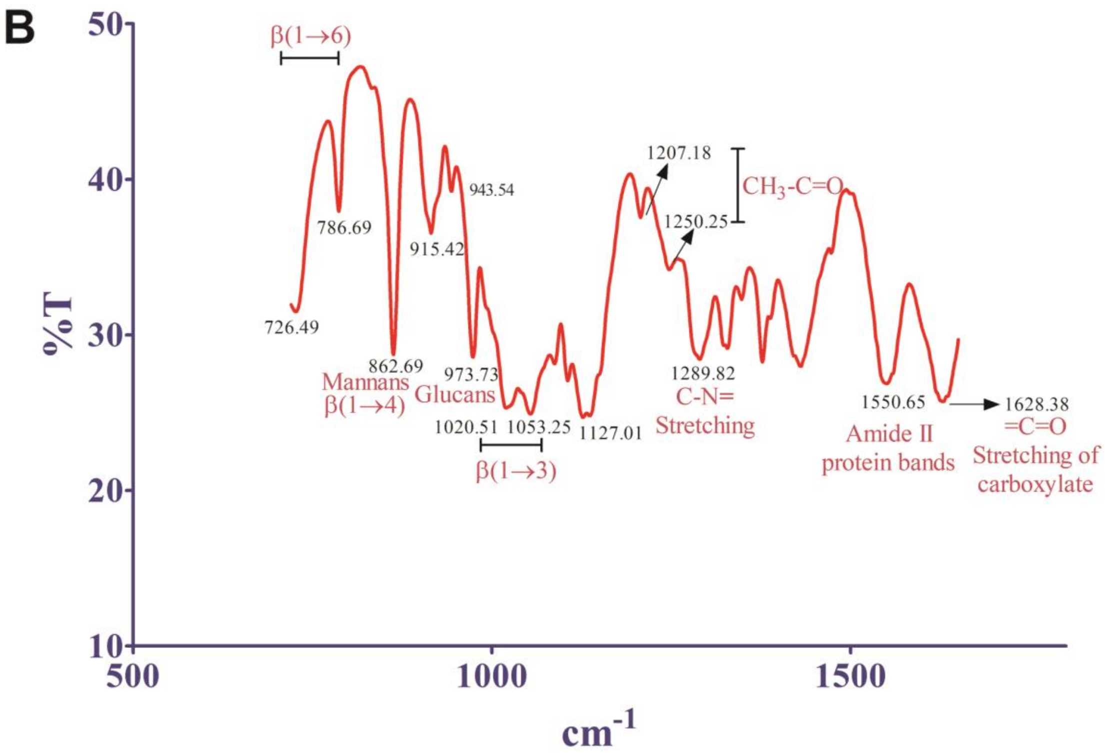

Supplementary Data 7: Protein Estimation (Bradford's Assay Standard Plot)

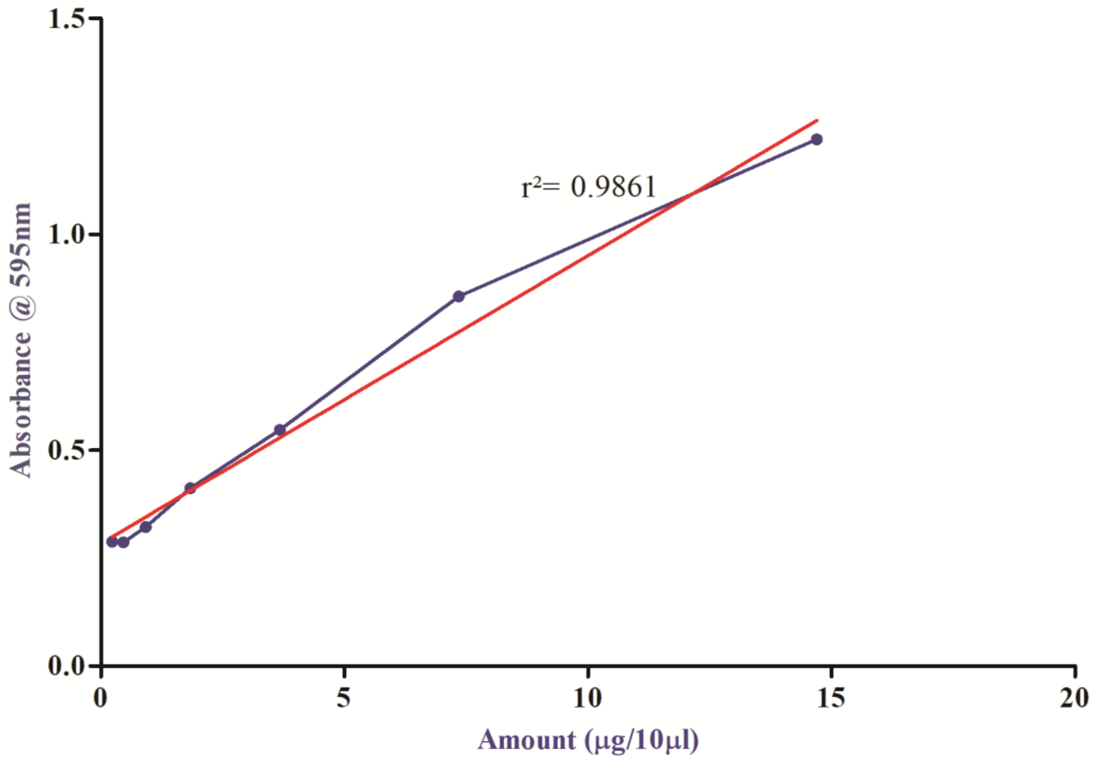

Supplementary Data 8: Estimation of Total Sugar-Standard Plot

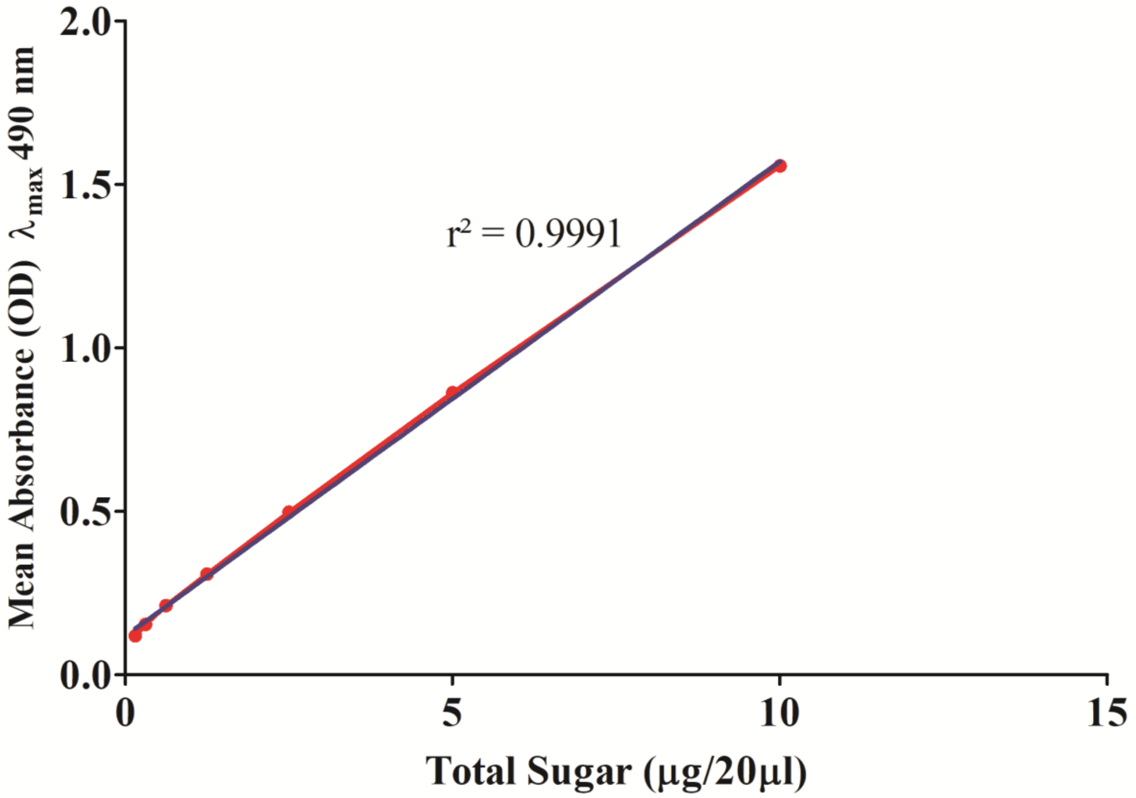

MALDI MS MS Data Sheet for Stool Isolates

C:\Users\madhurekha\Desktop\MALDI_Data\16th batch data 070718\BHU\1\0_B1\1\1233.6652.LIFT\1SRef

Comment 1

Comment 2

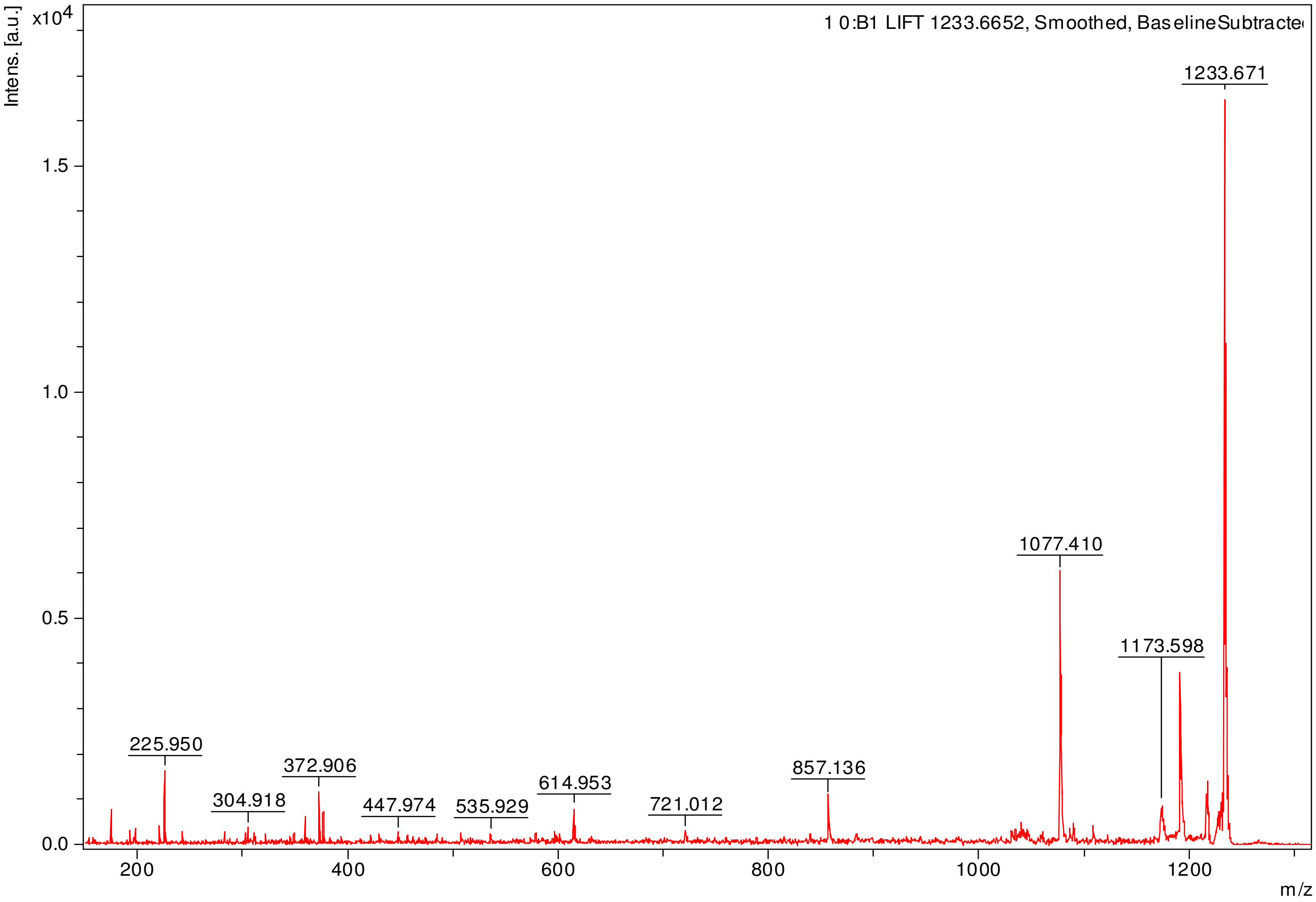

m/z	S/N	Quality Fac.	Res.	Intens.	Area
70.068	10.2	5409	459	273.67	46
112.055	11.8	688	885	328.70	47
115.045	14.0	17461	952	390.30	53
120.018	6.8	1115	887	187.48	29
129.031	4.1	58	1056	112.20	16
135.991	9.3	2618	986	253.14	40
146.970	3.1	140	1190	84.62	12
153.988	5.4	629	1041	149.57	25
157.983	5.5	43	1143	149.20	24
159.949	4.0	318	1108	109.10	18
175.006	28.0	7364	1248	794.30	130
189.934	3.3	32	1374	94.83	15
192.985	11.2	8348	1243	323.52	59
197.995	12.3	2628	1305	357.11	64
220.945	14.0	2660	1506	422.01	74
225.950	54.5	11333	1424	1658.66	317
242.947	9.7	1619	1547	305.40	58
282.937	7.9	6826	1418	275.22	68
285.921	3.3	75	1337	111.12	30
287.990	3.3	106	867	114.44	47
294.934	3.2	118	1090	113.33	38
302.905	7.6	564	1569	273.15	66
304.918	10.9	2169	1608	390.38	93
310.918	7.4	93	1368	269.50	77
321.936	6.0	133	1464	228.02	64
344.907	4.8	68	1386	192.02	62
348.915	5.7	159	1260	236.46	85
359.893	15.0	4574	1647	641.84	182
372.906	26.8	27723	1726	1187.54	336
376.913	16.5	14134	1579	734.08	230
382.880	3.9	45	1792	176.32	50
393.969	3.7	42	1550	174.58	59
421.867	3.9	164	1906	196.75	59
429.936	4.6	38	1588	235.44	86
447.974	5.3	113	1859	289.02	95
456.888	3.7	50	1704	206.05	76
473.867	3.1	39	1900	182.81	63
484.957	3.9	117	1457	240.48	111

C:\Users\madhurekha\Desktop\MALDI_Data\16th batch data 070718\BHU\1\0_B1\1\1233.6652.LIFT\1SRef

m/z	S/N	Quality Fac.	Res.	Intens.	Area
507.882	4.2	123	1649	270.76	118
535.929	3.7	119	1386	250.47	139
578.911	3.3	65	1723	243.83	120
614.953	10.6	1106	2101	824.38	364
721.012	3.5	96	1109	308.74	322
857.136	10.4	1623	1588	1062.27	992
1040.603	4.1	34	1867	493.92	528
1046.058	3.2	44	1835	378.85	416
1077.410	50.0	65736	2136	6039.84	6002
1086.565	3.0	126	2038	364.30	385
1090.141	4.0	340	2333	490.49	455
1173.598	9.1	632	1893	938.28	1213
1175.832	3.7	80	1866	374.74	493
1191.591	37.2	35486	2438	3638.72	3744
1216.686	15.4	1519	2388	1361.16	1482
1218.946	3.3	337	3683	284.46	201
1226.771	6.7	54	1589	568.78	944
1229.053	8.4	51	2121	717.19	896
1231.344	10.4	57	2733	883.30	858
1233.671	197.2	100212	3463	16603.22	12762

C:\Users\madhurekha\Desktop\MALDI_Data\16th batch data 070718\BHU\1\0_B1\1\1965.1137.LIFT\1SRef

Comment 1

Comment 2

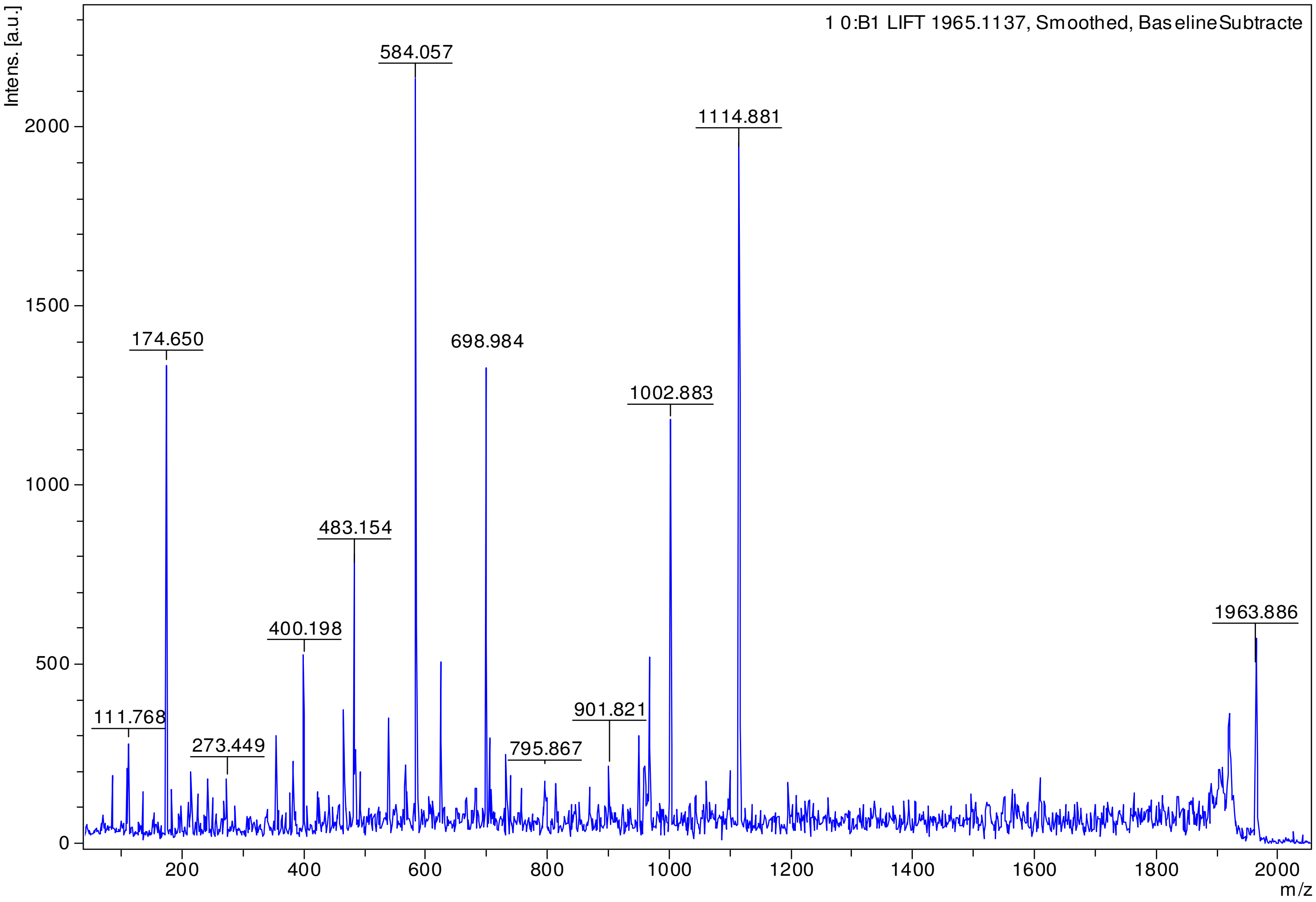

m/z	S/N	Quality Fac.	Res.	Intens.	Area
69.812	3.4	91	491	81.22	13
85.829	7.4	873	734	176.72	23
109.753	8.5	1786	1117	204.67	23
111.768	11.8	20258	1297	283.03	27
135.712	6.1	2760	1402	142.73	16
174.650	58.6	19275	1377	1338.50	198
182.645	6.0	1004	1096	138.10	27
194.575	4.1	34	1452	94.97	15
198.663	4.2	86	1175	96.10	19
210.607	4.9	347	1152	115.11	25
215.523	8.5	427	1223	196.45	41
226.543	6.0	172	1140	139.21	33
237.485	3.3	33	1934	78.07	12
242.501	7.3	349	1271	170.98	40
250.487	5.4	329	1792	128.14	22
256.470	3.9	37	1004	91.68	29
269.397	5.7	97	1609	136.37	28
273.449	7.4	956	1587	179.61	38
287.460	4.1	97	1674	103.33	22
355.275	10.0	2255	1715	292.44	79
370.232	3.0	35	2459	92.78	18
378.233	4.3	283	1848	135.55	36
383.231	7.7	87	1424	244.44	87
400.198	15.7	5048	1484	521.68	187
422.173	4.3	47	1620	149.96	53
426.226	3.4	114	1492	120.38	46
466.171	9.1	378	1786	355.04	128
483.154	19.2	1635	1882	770.71	275
493.075	4.8	297	1551	195.07	87
540.121	7.7	848	1639	343.65	162
567.046	4.6	141	2222	213.35	79
569.995	3.3	53	2366	154.20	54
584.057	44.6	59222	1862	2125.95	980
606.030	3.0	64	2386	145.43	55
625.959	10.2	3914	1804	504.02	265
698.984	26.1	66753	2287	1352.72	654
704.989	5.3	244	1984	277.50	156
732.967	5.1	187	1922	265.96	163

C:\Users\madhurekha\Desktop\MALDI_Data\16th batch data 070718\BHU\1\0_B1\1\1965.1137.LIFT\1SRef

m/z	S/N	Quality Fac.	Res.	Intens.	Area
738.935	3.8	333	1967	195.71	119
795.867	3.9	34	2324	208.26	119
901.821	3.8	233	2442	216.03	142
950.822	4.5	219	2767	256.62	161
967.863	9.0	2026	2556	521.32	364
1002.883	20.0	85180	2284	1179.20	973
1114.881	31.5	33218	2430	1930.88	1784
1903.946	4.1	71	3911	193.11	294
1908.591	3.4	78	2569	161.65	376
1919.807	7.2	490	2272	328.32	869
1963.886	12.8	958	4893	494.66	624

C:\Users\madhurekha\Desktop\MALDI_Data\16th batch data 070718\BHU\1\0_B1\1\2229.2398.LIFT\1SRef

Comment 1

Comment 2

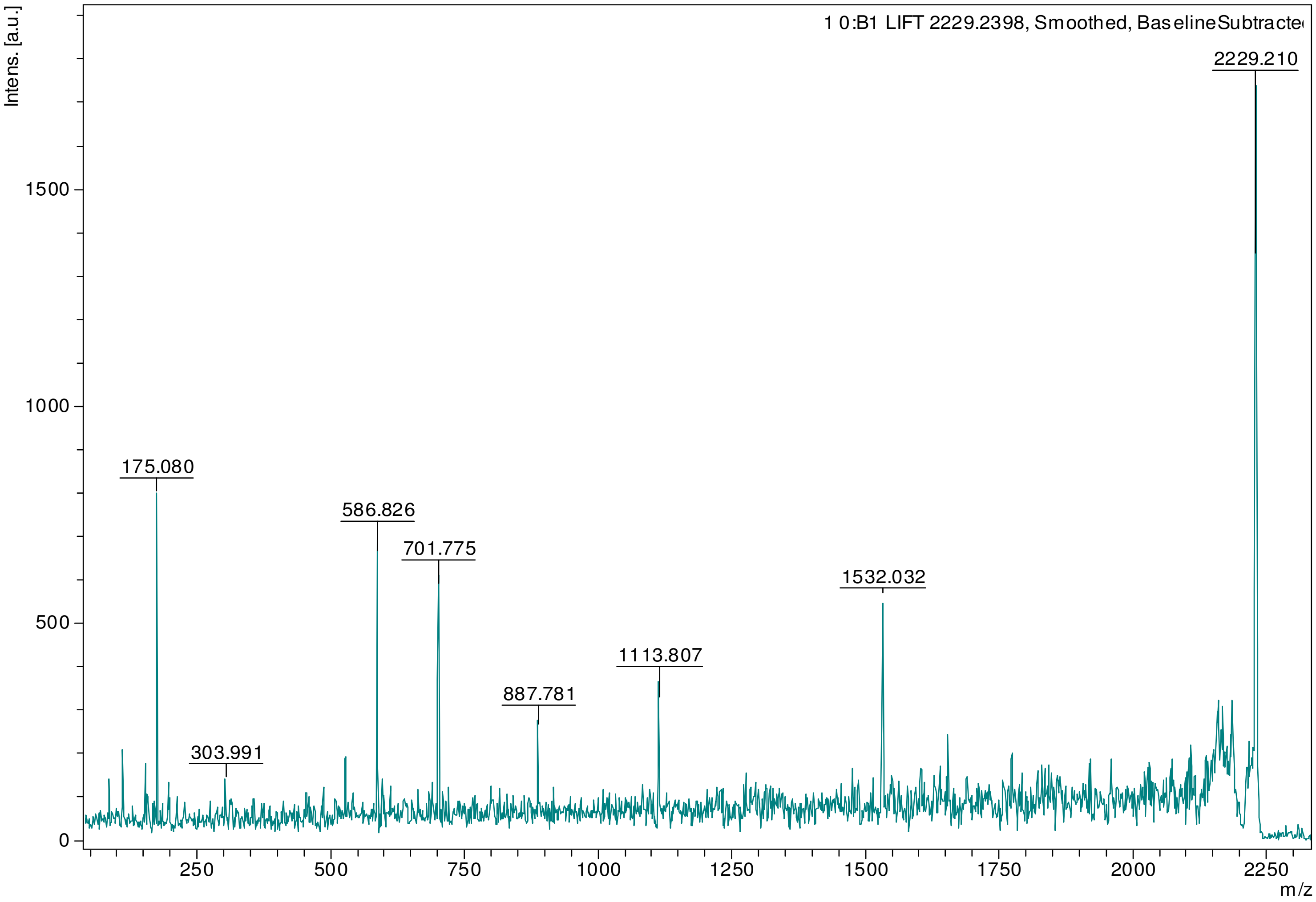

m/z	S/N	Quality Fac.	Res.	Intens.	Area
86.095	3.7	318	888	132.47	14
112.099	5.8	4262	1131	209.83	23
154.076	5.0	708	1240	171.83	24
157.520	3.0	296	1602	103.83	12
175.080	23.2	13888	1205	793.82	135
197.082	4.3	260	1048	144.46	32
213.030	3.2	33	865	107.68	32
303.991	3.8	227	1228	135.23	42
527.795	4.1	108	1297	201.81	117
586.826	12.4	9724	1771	656.38	320
701.775	9.9	12059	1907	582.11	339
887.781	3.9	226	1916	257.58	210
1113.807	4.2	205	2752	319.62	260
1532.032	6.0	1540	2508	559.64	876
2150.750	3.1	31	4027	196.21	335
2158.627	4.1	64	2397	257.36	742
2165.298	3.3	89	3326	202.64	423
2168.224	3.8	119	3072	231.81	525
2213.218	3.1	104	2741	166.76	436
2229.210	25.3	14503	4266	1341.53	2274

C:\Users\madhurekha\Desktop\MALDI_Data\16th batch data 070718\BHU\1\0_B1\1\1SRef

Comment 1

Comment 2

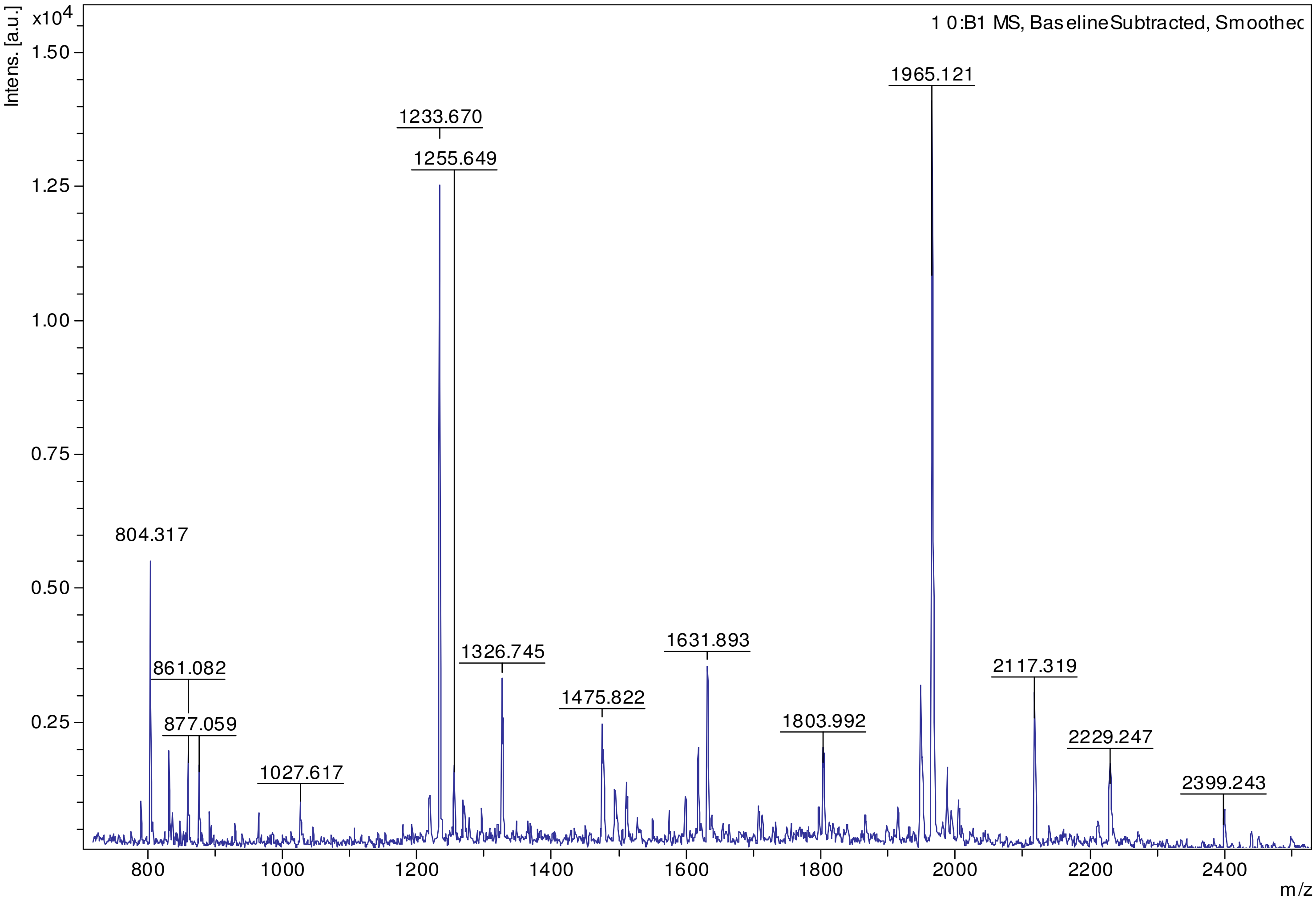

m/z	S/N	Quality Fac.	Res.	Intens.	Area
790.462	6.9	843	6070	942.72	216
804.317	42.4	6172	5917	5779.46	1387
832.350	13.4	1914	5114	1826.25	526
861.082	12.1	1600	5837	1666.02	429
877.059	10.7	569	5818	1483.75	399
1027.617	6.5	535	5784	931.21	323
1233.670	88.8	31051	8131	13319.41	4639
1255.649	9.8	1852	6814	1486.09	616
1269.671	6.1	208	6093	924.11	434
1326.745	22.1	11217	8403	3353.71	1277
1475.822	16.4	15650	9197	2514.78	1068
1477.780	7.0	1493	8072	1078.91	502
1494.883	7.0	572	6100	1064.15	667
1511.904	8.1	3724	8026	1233.09	609
1617.881	12.7	2817	8784	1924.65	988
1631.893	23.8	34192	9043	3577.56	1825
1803.992	11.7	3302	7377	1659.11	1252
1947.078	7.5	619	7733	940.75	756
1949.066	19.8	6629	8417	2495.57	1853
1965.121	86.9	12181	8794	10760.25	7834
1967.186	22.5	891	6835	2781.92	2554
1988.112	9.4	2140	7469	1132.43	969
2117.319	24.3	48010	9033	2501.34	1953
2229.247	14.0	1350	7998	1272.66	1172
2399.243	6.9	2736	5618	504.27	744

MATRIX SCIENCE Mascot Search Results

```
User          : sanovar
Email         : sanovar@sandor.co.in
Search title  :
MS data file  : DATA.TXT
Database      : SwissProt 2018_06 (557713 sequences; 200130199 residues)
Taxonomy      : Bacteria (Eubacteria) (333552 sequences)
Timestamp     : 11 Jul 2018 at 11:29:47 GMT
Warning       : A Peptide summary report will usually give a much clearer picture of MS/MS search results.
Top Score     : 138 for EFTU1_ECO24, Elongation factor Tu 1 OS=Escherichia coli O139:H28 (strain E24377A / ETEC) OX=331111 GN=tuf1 P
```

Mascot Score Histogram

Protein score is -10*Log(P), where P is the probability that the observed match is a random event.
Protein scores greater than 68 are significant (p<0.05).
Protein scores are derived from ions scores as a non-probabilistic basis for ranking protein hits.

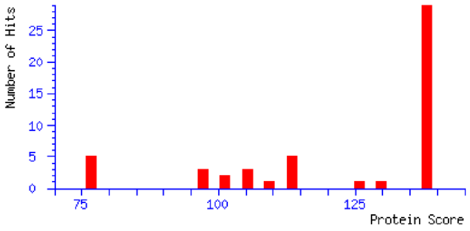

Protein Summary Report

Format As | Protein Summary (deprecated) | Help

Significance threshold p< 0.05 Max. number of hits 20

Preferred taxonomy All entries

Re-Search All | Search Unmatched

Index

	Accession	Mass	Score	Description
1.	EFTU1_ECO24	43427	138	Elongation factor Tu 1 OS=Escherichia coli O139:H28 (strain E24377A / ETEC) OX=331111 GN=tuf1 PE=3 SV=1
2.	EFTU1_ECOHS	43427	138	Elongation factor Tu 1 OS=Escherichia coli O9:H4 (strain HS) OX=331112 GN=tuf1 PE=3 SV=1
3.	EFTU1_ECOK1	43427	138	Elongation factor Tu 1 OS=Escherichia coli O1:K1 / APEC OX=405955 GN=tuf1 PE=3 SV=2
4.	EFTU1_ECOL5	43427	138	Elongation factor Tu 1 OS=Escherichia coli O6:K15:H31 (strain 536 / UPEC) OX=362663 GN=tuf1 PE=3 SV=1
5.	EFTU1_ECOLC	43427	138	Elongation factor Tu 1 OS=Escherichia coli (strain ATCC 8739 / DSM 1576 / Crooks) OX=481805 GN=tuf1 PE=
6.	EFTU1_ECOLI	43427	138	Elongation factor Tu 1 OS=Escherichia coli (strain K12) OX=83333 GN=tufA PE=1 SV=1
7.	EFTU1_ECOUT	43427	138	Elongation factor Tu 1 OS=Escherichia coli (strain UTI89 / UPEC) OX=364106 GN=tuf1 PE=1 SV=1
8.	EFTU1_SHIF8	43426	138	Elongation factor Tu 1 OS=Shigella flexneri serotype 5b (strain 8401) OX=373384 GN=tuf1 PE=3 SV=1
9.	EFTU1_SHISS	43427	138	Elongation factor Tu 1 OS=Shigella sonnei (strain Ss046) OX=300269 GN=tuf1 PE=3 SV=1
10.	EFTU2_ECO24	43456	138	Elongation factor Tu 2 OS=Escherichia coli O139:H28 (strain E24377A / ETEC) OX=331111 GN=tuf2 PE=3 SV=1
11.	EFTU2_ECOHS	43457	138	Elongation factor Tu 2 OS=Escherichia coli O9:H4 (strain HS) OX=331112 GN=tuf2 PE=3 SV=1
12.	EFTU2_ECOK1	43457	138	Elongation factor Tu 2 OS=Escherichia coli O1:K1 / APEC OX=405955 GN=tuf2 PE=3 SV=1
13.	EFTU2_ECOL5	43457	138	Elongation factor Tu 2 OS=Escherichia coli O6:K15:H31 (strain 536 / UPEC) OX=362663 GN=tuf2 PE=3 SV=1
14.	EFTU2_ECOLC	43457	138	Elongation factor Tu 2 OS=Escherichia coli (strain ATCC 8739 / DSM 1576 / Crooks) OX=481805 GN=tuf2 PE=
15.	EFTU2_ECOLI	43457	138	Elongation factor Tu 2 OS=Escherichia coli (strain K12) OX=83333 GN=tufB PE=1 SV=1
16.	EFTU2_ECOUT	43457	138	Elongation factor Tu 2 OS=Escherichia coli (strain UTI89 / UPEC) OX=364106 GN=tuf2 PE=3 SV=2
17.	EFTU2_SHIF8	43457	138	Elongation factor Tu 2 OS=Shigella flexneri serotype 5b (strain 8401) OX=373384 GN=tuf2 PE=3 SV=1
18.	EFTU2_SHISS	43457	138	Elongation factor Tu 2 OS=Shigella sonnei (strain Ss046) OX=300269 GN=tuf2 PE=3 SV=1
19.	EFTU_ECO57	43457	138	Elongation factor Tu OS=Escherichia coli O157:H7 OX=83334 GN=tufA PE=3 SV=2
20.	EFTU_ECOL6	43457	138	Elongation factor Tu OS=Escherichia coli O6:H1 (strain CFT073 / ATCC 700928 / UPEC) OX=199310 GN=tufA P

Results List

1. EFTU1_ECO24 **Mass:** 43427 **Score:** 138 **Expect:** 5.3e-09 **Matches:** 11

Elongation factor Tu 1 OS=Escherichia coli O139:H28 (strain E24377A / ETEC) OX=331111 GN=tuf1 PE=3 SV=1

Observed	Mr(expt)	Mr(calc)	ppm	Start	End	Miss	Ions	Peptide
837.5072	836.4999	836.4868	15.7	118 -	124	0	---	R.EHILLGR.Q
1027.6166	1026.6093	1026.5822	26.4	271 -	280	0	---	R.AGENVGVLLR.G
1214.6581	1213.6508	1213.6230	22.9	305 -	314	0	---	K.FESEVYILSK.D
1233.6701	1232.6629	1232.6091	43.6	326 -	334	0	15	K.GYRPQFYFR.T
1315.7305	1314.7232	1314.6060	89.1	254 -	264	1	---	K.STCTGVEMFRK.L
1574.9723	1573.9650	1573.9304	22.0	290 -	304	0	---	R.GQVLAKPGTIKPHTK.F
1728.9325	1727.9253	1727.8366	51.3	305 -	319	1	---	K.FESEVYILSKDEGGR.H
1797.0350	1796.0277	1795.9325	53.1	359 -	374	0	---	K.MVVTLIHPIAMDDGLR.F + Oxidation (M)
1803.9918	1802.9845	1802.8799	58.0	60 -	75	0	---	R.GITINTSHVEYDTPTR.H
1965.1206	1964.1134	1963.9527	81.8	156 -	172	0	44	R.ELLSQYDFPGDDTPIVR.G
2117.3190	2116.3117	2116.1568	73.2	206 -	224	0	28	R.AIDKPFLLPIEDVFSISGR.G

No match to: 723.3202, 725.3349, 790.4618, 804.3175, 832.3504, 848.3077, 855.0634, 861.0816, 877.0593, 879.0642, 893.0384, 897.0395, 930.4819, 941.5245, 965.5432, 985.5503, 1001.5439, 1046.5846, 1179.6348, 1193.6685, 1219.5995, 1255.6486, 1269.6710, 1271.6285, 1277.6571, 1296.7322, 1320.6617, 1326.7448, 1348.7190, 1368.7612, 1450.7841, 1472.8880, 1475.8216, 1477.7796, 1494.8828, 1497.7996, 1509.8403, 1511.9038, 1527.8609, 1550.8947, 1598.9195, 1617.8806, 1631.8934, 1654.9031, 1707.9342, 1712.9042, 1733.0067, 1749.9856, 1755.9948, 1818.0212, 1866.0850, 1898.0605, 1915.0945, 1931.0593, 1947.0781, 1949.0662, 1967.1856, 1981.0932, 1988.1120, 1994.1406, 2005.1346, 2010.0916, 2139.2472, 2211.2249, 2229.2467, 2233.2426, 2271.2633, 2384.1999, 2399.2428, 2439.4144, 2451.4028, 2498.4046, 2615.4719, 2705.3847, 2808.6002, 3284.5981

2. EFTU1_ECOHS **Mass:** 43427 **Score:** 138 **Expect:** 5.3e-09 **Matches:** 11

Elongation factor Tu 1 OS=Escherichia coli O9:H4 (strain HS) OX=331112 GN=tuf1 PE=3 SV=1

Observed	Mr(expt)	Mr(calc)	ppm	Start	End	Miss	Ions	Peptide
837.5072	836.4999	836.4868	15.7	118 -	124	0	---	R.EHILLGR.Q

1027.6166	1026.6093	1026.5822	26.4	271	280	0	---	R.AGENVGVLLR.G
1214.6581	1213.6508	1213.6230	22.9	305	314	0	---	K.FESEVYILSK.D
1233.6701	1232.6629	1232.6091	43.6	326	334	0	15	K.GYRPQFYFR.T
1315.7305	1314.7232	1314.6060	89.1	254	264	1	---	K.STCTGVEMFRK.L
1574.9723	1573.9650	1573.9304	22.0	290	304	0	---	R.GQVLAKPGTIKPHTK.F
1728.9325	1727.9253	1727.8366	51.3	305	319	1	---	K.FESEVYILSKDEGGR.H
1797.0350	1796.0277	1795.9325	53.1	359	374	0	---	K.MVVTLIHPIAMDDGLR.F + Oxidation (M)
1803.9918	1802.9845	1802.8799	58.0	60	75	0	---	R.GITINTSHVEYDTPTR.H
1965.1206	1964.1134	1963.9527	81.8	156	172	0	44	R.ELLSQYDFPGDDTPIVR.G
2117.3190	2116.3117	2116.1568	73.2	206	224	0	28	R.AIDKPFLLPIEDVFSISGR.G

No match to: 723.3202, 725.3349, 790.4618, 804.3175, 832.3504, 848.3077, 855.0634, 861.0816, 877.0593, 879.0642, 893.0384, 897.0395, 930.4819, 941.5245, 965.5432, 985.5503, 1001.5439, 1046.5846, 1179.6348, 1193.6685, 1219.5995, 1255.6486, 1269.6710, 1271.6285, 1277.6571, 1296.7322, 1320.6617, 1326.7448, 1348.7190, 1368.7612, 1450.7841, 1472.8880, 1475.8216, 1477.7796, 1494.8828, 1497.7996, 1509.8403, 1511.9038, 1527.8609, 1550.8947, 1598.9195, 1617.8806, 1631.8934, 1654.9031, 1707.9342, 1712.9042, 1733.0067, 1749.9856, 1755.9948, 1818.0212, 1866.0850, 1898.0605, 1915.0945, 1931.0593, 1947.0781, 1949.0662, 1967.1856, 1981.0932, 1988.1120, 1994.1406, 2005.1346, 2010.0916, 2139.2472, 2211.2249, 2229.2467, 2233.2426, 2271.2633, 2384.1999, 2399.2428, 2439.4144, 2451.4028, 2498.4046, 2615.4719, 2705.3847, 2808.6002, 3284.5981

3. EFTU1_ECOK1 **Mass:** 43427 **Score:** 138 **Expect:** 5.3e-09 **Matches:** 11

Elongation factor Tu 1 OS=Escherichia coli O1:K1 / APEC OX=405955 GN=tuf1 PE=3 SV=2

Observed	Mr(expt)	Mr(calc)	ppm	Start	End	Miss	Ions	Peptide
837.5072	836.4999	836.4868	15.7	118	124	0	---	R.EHILLGR.Q
1027.6166	1026.6093	1026.5822	26.4	271	280	0	---	R.AGENVGVLLR.G
1214.6581	1213.6508	1213.6230	22.9	305	314	0	---	K.FESEVYILSK.D
1233.6701	1232.6629	1232.6091	43.6	326	334	0	15	K.GYRPQFYFR.T
1315.7305	1314.7232	1314.6060	89.1	254	264	1	---	K.STCTGVEMFRK.L
1574.9723	1573.9650	1573.9304	22.0	290	304	0	---	R.GQVLAKPGTIKPHTK.F
1728.9325	1727.9253	1727.8366	51.3	305	319	1	---	K.FESEVYILSKDEGGR.H
1797.0350	1796.0277	1795.9325	53.1	359	374	0	---	K.MVVTLIHPIAMDDGLR.F + Oxidation (M)
1803.9918	1802.9845	1802.8799	58.0	60	75	0	---	R.GITINTSHVEYDTPTR.H
1965.1206	1964.1134	1963.9527	81.8	156	172	0	44	R.ELLSQYDFPGDDTPIVR.G
2117.3190	2116.3117	2116.1568	73.2	206	224	0	28	R.AIDKPFLLPIEDVFSISGR.G

No match to: 723.3202, 725.3349, 790.4618, 804.3175, 832.3504, 848.3077, 855.0634, 861.0816, 877.0593, 879.0642, 893.0384, 897.0395, 930.4819, 941.5245, 965.5432, 985.5503, 1001.5439, 1046.5846, 1179.6348, 1193.6685, 1219.5995, 1255.6486, 1269.6710, 1271.6285, 1277.6571, 1296.7322, 1320.6617, 1326.7448, 1348.7190, 1368.7612, 1450.7841, 1472.8880, 1475.8216, 1477.7796, 1494.8828, 1497.7996, 1509.8403, 1511.9038, 1527.8609, 1550.8947, 1598.9195, 1617.8806, 1631.8934, 1654.9031, 1707.9342, 1712.9042, 1733.0067, 1749.9856, 1755.9948, 1818.0212, 1866.0850, 1898.0605, 1915.0945, 1931.0593, 1947.0781, 1949.0662, 1967.1856, 1981.0932, 1988.1120, 1994.1406, 2005.1346, 2010.0916, 2139.2472, 2211.2249, 2229.2467, 2233.2426, 2271.2633, 2384.1999, 2399.2428, 2439.4144, 2451.4028, 2498.4046, 2615.4719, 2705.3847, 2808.6002, 3284.5981

4. EFTU1_ECOL5 **Mass:** 43427 **Score:** 138 **Expect:** 5.3e-09 **Matches:** 11

Elongation factor Tu 1 OS=Escherichia coli O6:K15:H31 (strain 536 / UPEC) OX=362663 GN=tuf1 PE=3 SV=1

Observed	Mr(expt)	Mr(calc)	ppm	Start	End	Miss	Ions	Peptide
837.5072	836.4999	836.4868	15.7	118	124	0	---	R.EHILLGR.Q
1027.6166	1026.6093	1026.5822	26.4	271	280	0	---	R.AGENVGVLLR.G
1214.6581	1213.6508	1213.6230	22.9	305	314	0	---	K.FESEVYILSK.D
1233.6701	1232.6629	1232.6091	43.6	326	334	0	15	K.GYRPQFYFR.T
1315.7305	1314.7232	1314.6060	89.1	254	264	1	---	K.STCTGVEMFRK.L
1574.9723	1573.9650	1573.9304	22.0	290	304	0	---	R.GQVLAKPGTIKPHTK.F
1728.9325	1727.9253	1727.8366	51.3	305	319	1	---	K.FESEVYILSKDEGGR.H
1797.0350	1796.0277	1795.9325	53.1	359	374	0	---	K.MVVTLIHPIAMDDGLR.F + Oxidation (M)
1803.9918	1802.9845	1802.8799	58.0	60	75	0	---	R.GITINTSHVEYDTPTR.H
1965.1206	1964.1134	1963.9527	81.8	156	172	0	44	R.ELLSQYDFPGDDTPIVR.G
2117.3190	2116.3117	2116.1568	73.2	206	224	0	28	R.AIDKPFLLPIEDVFSISGR.G

No match to: 723.3202, 725.3349, 790.4618, 804.3175, 832.3504, 848.3077, 855.0634, 861.0816, 877.0593, 879.0642, 893.0384, 897.0395, 930.4819, 941.5245, 965.5432, 985.5503, 1001.5439, 1046.5846, 1179.6348, 1193.6685, 1219.5995, 1255.6486, 1269.6710, 1271.6285, 1277.6571, 1296.7322, 1320.6617, 1326.7448, 1348.7190, 1368.7612, 1450.7841, 1472.8880, 1475.8216, 1477.7796, 1494.8828, 1497.7996, 1509.8403, 1511.9038, 1527.8609, 1550.8947, 1598.9195, 1617.8806, 1631.8934, 1654.9031, 1707.9342, 1712.9042, 1733.0067, 1749.9856, 1755.9948, 1818.0212, 1866.0850, 1898.0605, 1915.0945, 1931.0593, 1947.0781, 1949.0662, 1967.1856, 1981.0932, 1988.1120, 1994.1406, 2005.1346, 2010.0916, 2139.2472, 2211.2249, 2229.2467, 2233.2426, 2271.2633, 2384.1999, 2399.2428, 2439.4144, 2451.4028, 2498.4046, 2615.4719, 2705.3847, 2808.6002, 3284.5981

5. EFTU1_ECOLC **Mass:** 43427 **Score:** 138 **Expect:** 5.3e-09 **Matches:** 11

Elongation factor Tu 1 OS=Escherichia coli (strain ATCC 8739 / DSM 1576 / Crooks) OX=481805 GN=tuf1 PE=3 SV=1

Observed	Mr(expt)	Mr(calc)	ppm	Start	End	Miss	Ions	Peptide
837.5072	836.4999	836.4868	15.7	118	124	0	---	R.EHILLGR.Q
1027.6166	1026.6093	1026.5822	26.4	271	280	0	---	R.AGENVGVLLR.G
1214.6581	1213.6508	1213.6230	22.9	305	314	0	---	K.FESEVYILSK.D
1233.6701	1232.6629	1232.6091	43.6	326	334	0	15	K.GYRPQFYFR.T
1315.7305	1314.7232	1314.6060	89.1	254	264	1	---	K.STCTGVEMFRK.L
1574.9723	1573.9650	1573.9304	22.0	290	304	0	---	R.GQVLAKPGTIKPHTK.F
1728.9325	1727.9253	1727.8366	51.3	305	319	1	---	K.FESEVYILSKDEGGR.H
1797.0350	1796.0277	1795.9325	53.1	359	374	0	---	K.MVVTLIHPIAMDDGLR.F + Oxidation (M)
1803.9918	1802.9845	1802.8799	58.0	60	75	0	---	R.GITINTSHVEYDTPTR.H
1965.1206	1964.1134	1963.9527	81.8	156	172	0	44	R.ELLSQYDFPGDDTPIVR.G
2117.3190	2116.3117	2116.1568	73.2	206	224	0	28	R.AIDKPFLLPIEDVFSISGR.G

No match to: 723.3202, 725.3349, 790.4618, 804.3175, 832.3504, 848.3077, 855.0634, 861.0816, 877.0593, 879.0642, 893.0384, 897.0395, 930.4819, 941.5245, 965.5432, 985.5503, 1001.5439, 1046.5846, 1179.6348, 1193.6685, 1219.5995, 1255.6486, 1269.6710, 1271.6285, 1277.6571, 1296.7322, 1320.6617, 1326.7448, 1348.7190, 1368.7612, 1450.7841, 1472.8880, 1475.8216, 1477.7796, 1494.8828, 1497.7996, 1509.8403, 1511.9038, 1527.8609, 1550.8947, 1598.9195, 1617.8806, 1631.8934, 1654.9031, 1707.9342, 1712.9042, 1733.0067, 1749.9856, 1755.9948, 1818.0212, 1866.0850, 1898.0605, 1915.0945, 1931.0593, 1947.0781, 1949.0662, 1967.1856, 1981.0932, 1988.1120, 1994.1406, 2005.1346, 2010.0916, 2139.2472, 2211.2249, 2229.2467, 2233.2426, 2271.2633, 2384.1999, 2399.2428, 2439.4144, 2451.4028, 2498.4046, 2615.4719, 2705.3847, 2808.6002, 3284.5981

6. EFTU1_ECOLI **Mass:** 43427 **Score:** 138 **Expect:** 5.3e-09 **Matches:** 11

Elongation factor Tu 1 OS=Escherichia coli (strain K12) OX=83333 GN=tufA PE=1 SV=1

Observed	Mr(expt)	Mr(calc)	ppm	Start	End	Miss	Ions	Peptide
837.5072	836.4999	836.4868	15.7	118	124	0	---	R.EHILLGR.Q
1027.6166	1026.6093	1026.5822	26.4	271	280	0	---	R.AGENVGVLLR.G
1214.6581	1213.6508	1213.6230	22.9	305	314	0	---	K.FESEVYILSK.D
1233.6701	1232.6629	1232.6091	43.6	326	334	0	15	K.GYRPQFYFR.T
1315.7305	1314.7232	1314.6060	89.1	254	264	1	---	K.STCTGVEMFRK.L
1574.9723	1573.9650	1573.9304	22.0	290	304	0	---	R.GQVLAKPGTIKPHTK.F

1728.9325	1727.9253	1727.8366	51.3	305	319	1	---	K.FESEVYILSKDEGGR.H
1797.0350	1796.0277	1795.9325	53.1	359	374	0	---	K.MVVTLIHPIAMDDGLR.F + Oxidation (M)
1803.9918	1802.9845	1802.8799	58.0	60	75	0	---	R.GITINTSHVEYDTPTR.H
1965.1206	1964.1134	1963.9527	81.8	156	172	0	44	R.ELLSQYDFPGDDTPIVR.G
2117.3190	2116.3117	2116.1568	73.2	206	224	0	28	R.AIDKPFLLPIEDVFSISGR.G

No match to: 723.3202, 725.3349, 790.4618, 804.3175, 832.3504, 848.3077, 855.0634, 861.0816, 877.0593, 879.0642, 893.0384, 897.0395, 930.4819, 941.5245, 965.5432, 985.5503, 1001.5439, 1046.5846, 1179.6348, 1193.6685, 1219.5995, 1255.6486, 1269.6710, 1271.6285, 1277.6571, 1296.7322, 1320.6617, 1326.7448, 1348.7190, 1368.7612, 1450.7841, 1472.8880, 1475.8216, 1477.7796, 1494.8828, 1497.7996, 1509.8403, 1511.9038, 1527.8609, 1550.8947, 1598.9195, 1617.8806, 1631.8934, 1654.9031, 1707.9342, 1712.9042, 1733.0067, 1749.9856, 1755.9948, 1818.0212, 1866.0850, 1898.0605, 1915.0945, 1931.0593, 1947.0781, 1949.0662, 1967.1856, 1981.0932, 1988.1120, 1994.1406, 2005.1346, 2010.0916, 2139.2472, 2211.2249, 2229.2467, 2233.2426, 2271.2633, 2384.1999, 2399.2428, 2439.4144, 2451.4028, 2498.4046, 2615.4719, 2705.3847, 2808.6002, 3284.5981

7. EFTU1_ECOUT **Mass:** 43427 **Score:** 138 **Expect:** 5.3e-09 **Matches:** 11

Elongation factor Tu 1 OS=Escherichia coli (strain UTI89 / UPEC) OX=364106 GN=tuf1 PE=1 SV=1

Observed	Mr(expt)	Mr(calc)	ppm	Start	End	Miss	Ions	Peptide
837.5072	836.4999	836.4868	15.7	118	124	0	---	R.EHILLGR.Q
1027.6166	1026.6093	1026.5822	26.4	271	280	0	---	R.AGENVGVLLR.G
1214.6581	1213.6508	1213.6230	22.9	305	314	0	---	K.FESEVYILSK.D
1233.6701	1232.6629	1232.6091	43.6	326	334	0	15	K.GYRPQFYFR.T
1315.7305	1314.7232	1314.6060	89.1	254	264	1	---	K.STCTGVEMFRK.L
1574.9723	1573.9650	1573.9304	22.0	290	304	0	---	R.GQVLAKPGTIKPHTK.F
1728.9325	1727.9253	1727.8366	51.3	305	319	1	---	K.FESEVYILSKDEGGR.H
1797.0350	1796.0277	1795.9325	53.1	359	374	0	---	K.MVVTLIHPIAMDDGLR.F + Oxidation (M)
1803.9918	1802.9845	1802.8799	58.0	60	75	0	---	R.GITINTSHVEYDTPTR.H
1965.1206	1964.1134	1963.9527	81.8	156	172	0	44	R.ELLSQYDFPGDDTPIVR.G
2117.3190	2116.3117	2116.1568	73.2	206	224	0	28	R.AIDKPFLLPIEDVFSISGR.G

No match to: 723.3202, 725.3349, 790.4618, 804.3175, 832.3504, 848.3077, 855.0634, 861.0816, 877.0593, 879.0642, 893.0384, 897.0395, 930.4819, 941.5245, 965.5432, 985.5503, 1001.5439, 1046.5846, 1179.6348, 1193.6685, 1219.5995, 1255.6486, 1269.6710, 1271.6285, 1277.6571, 1296.7322, 1320.6617, 1326.7448, 1348.7190, 1368.7612, 1450.7841, 1472.8880, 1475.8216, 1477.7796, 1494.8828, 1497.7996, 1509.8403, 1511.9038, 1527.8609, 1550.8947, 1598.9195, 1617.8806, 1631.8934, 1654.9031, 1707.9342, 1712.9042, 1733.0067, 1749.9856, 1755.9948, 1818.0212, 1866.0850, 1898.0605, 1915.0945, 1931.0593, 1947.0781, 1949.0662, 1967.1856, 1981.0932, 1988.1120, 1994.1406, 2005.1346, 2010.0916, 2139.2472, 2211.2249, 2229.2467, 2233.2426, 2271.2633, 2384.1999, 2399.2428, 2439.4144, 2451.4028, 2498.4046, 2615.4719, 2705.3847, 2808.6002, 3284.5981

8. EFTU1_SHIF8 **Mass:** 43426 **Score:** 138 **Expect:** 5.3e-09 **Matches:** 11

Elongation factor Tu 1 OS=Shigella flexneri serotype 5b (strain 8401) OX=373384 GN=tuf1 PE=3 SV=1

Observed	Mr(expt)	Mr(calc)	ppm	Start	End	Miss	Ions	Peptide
837.5072	836.4999	836.4868	15.7	118	124	0	---	R.EHILLGR.Q
1027.6166	1026.6093	1026.5822	26.4	271	280	0	---	R.AGENVGVLLR.G
1214.6581	1213.6508	1213.6230	22.9	305	314	0	---	K.FESEVYILSK.D
1233.6701	1232.6629	1232.6091	43.6	326	334	0	15	K.GYRPQFYFR.T
1315.7305	1314.7232	1314.6060	89.1	254	264	1	---	K.STCTGVEMFRK.L
1574.9723	1573.9650	1573.9304	22.0	290	304	0	---	R.GQVLAKPGTIKPHTK.F
1728.9325	1727.9253	1727.8366	51.3	305	319	1	---	K.FESEVYILSKDEGGR.H
1797.0350	1796.0277	1795.9325	53.1	359	374	0	---	K.MVVTLIHPIAMDDGLR.F + Oxidation (M)
1803.9918	1802.9845	1802.8799	58.0	60	75	0	---	R.GITINTSHVEYDTPTR.H
1965.1206	1964.1134	1963.9527	81.8	156	172	0	44	R.ELLSQYDFPGDDTPIVR.G
2117.3190	2116.3117	2116.1568	73.2	206	224	0	28	R.AIDKPFLLPIEDVFSISGR.G

No match to: 723.3202, 725.3349, 790.4618, 804.3175, 832.3504, 848.3077, 855.0634, 861.0816, 877.0593, 879.0642, 893.0384, 897.0395, 930.4819, 941.5245, 965.5432, 985.5503, 1001.5439, 1046.5846, 1179.6348, 1193.6685, 1219.5995, 1255.6486, 1269.6710, 1271.6285, 1277.6571, 1296.7322, 1320.6617, 1326.7448, 1348.7190, 1368.7612, 1450.7841, 1472.8880, 1475.8216, 1477.7796, 1494.8828, 1497.7996, 1509.8403, 1511.9038, 1527.8609, 1550.8947, 1598.9195, 1617.8806, 1631.8934, 1654.9031, 1707.9342, 1712.9042, 1733.0067, 1749.9856, 1755.9948, 1818.0212, 1866.0850, 1898.0605, 1915.0945, 1931.0593, 1947.0781, 1949.0662, 1967.1856, 1981.0932, 1988.1120, 1994.1406, 2005.1346, 2010.0916, 2139.2472, 2211.2249, 2229.2467, 2233.2426, 2271.2633, 2384.1999, 2399.2428, 2439.4144, 2451.4028, 2498.4046, 2615.4719, 2705.3847, 2808.6002, 3284.5981

9. EFTU1_SHISS **Mass:** 43427 **Score:** 138 **Expect:** 5.3e-09 **Matches:** 11

Elongation factor Tu 1 OS=Shigella sonnei (strain Ss046) OX=300269 GN=tuf1 PE=3 SV=1

Observed	Mr(expt)	Mr(calc)	ppm	Start	End	Miss	Ions	Peptide
837.5072	836.4999	836.4868	15.7	118	124	0	---	R.EHILLGR.Q
1027.6166	1026.6093	1026.5822	26.4	271	280	0	---	R.AGENVGVLLR.G
1214.6581	1213.6508	1213.6230	22.9	305	314	0	---	K.FESEVYILSK.D
1233.6701	1232.6629	1232.6091	43.6	326	334	0	15	K.GYRPQFYFR.T
1315.7305	1314.7232	1314.6060	89.1	254	264	1	---	K.STCTGVEMFRK.L
1574.9723	1573.9650	1573.9304	22.0	290	304	0	---	R.GQVLAKPGTIKPHTK.F
1728.9325	1727.9253	1727.8366	51.3	305	319	1	---	K.FESEVYILSKDEGGR.H
1797.0350	1796.0277	1795.9325	53.1	359	374	0	---	K.MVVTLIHPIAMDDGLR.F + Oxidation (M)
1803.9918	1802.9845	1802.8799	58.0	60	75	0	---	R.GITINTSHVEYDTPTR.H
1965.1206	1964.1134	1963.9527	81.8	156	172	0	44	R.ELLSQYDFPGDDTPIVR.G
2117.3190	2116.3117	2116.1568	73.2	206	224	0	28	R.AIDKPFLLPIEDVFSISGR.G

No match to: 723.3202, 725.3349, 790.4618, 804.3175, 832.3504, 848.3077, 855.0634, 861.0816, 877.0593, 879.0642, 893.0384, 897.0395, 930.4819, 941.5245, 965.5432, 985.5503, 1001.5439, 1046.5846, 1179.6348, 1193.6685, 1219.5995, 1255.6486, 1269.6710, 1271.6285, 1277.6571, 1296.7322, 1320.6617, 1326.7448, 1348.7190, 1368.7612, 1450.7841, 1472.8880, 1475.8216, 1477.7796, 1494.8828, 1497.7996, 1509.8403, 1511.9038, 1527.8609, 1550.8947, 1598.9195, 1617.8806, 1631.8934, 1654.9031, 1707.9342, 1712.9042, 1733.0067, 1749.9856, 1755.9948, 1818.0212, 1866.0850, 1898.0605, 1915.0945, 1931.0593, 1947.0781, 1949.0662, 1967.1856, 1981.0932, 1988.1120, 1994.1406, 2005.1346, 2010.0916, 2139.2472, 2211.2249, 2229.2467, 2233.2426, 2271.2633, 2384.1999, 2399.2428, 2439.4144, 2451.4028, 2498.4046, 2615.4719, 2705.3847, 2808.6002, 3284.5981

10. EFTU2_ECO24 **Mass:** 43456 **Score:** 138 **Expect:** 5.3e-09 **Matches:** 11

Elongation factor Tu 2 OS=Escherichia coli O139:H28 (strain E24377A / ETEC) OX=331111 GN=tuf2 PE=3 SV=1

Observed	Mr(expt)	Mr(calc)	ppm	Start	End	Miss	Ions	Peptide
837.5072	836.4999	836.4868	15.7	118	124	0	---	R.EHILLGR.Q
1027.6166	1026.6093	1026.5822	26.4	271	280	0	---	R.AGENVGVLLR.G
1214.6581	1213.6508	1213.6230	22.9	305	314	0	---	K.FESEVYILSK.D
1233.6701	1232.6629	1232.6091	43.6	326	334	0	15	K.GYRPQFYFR.T
1315.7305	1314.7232	1314.6060	89.1	254	264	1	---	K.STCTGVEMFRK.L
1574.9723	1573.9650	1573.9304	22.0	290	304	0	---	R.GQVLAKPGTIKPHTK.F
1728.9325	1727.9253	1727.8366	51.3	305	319	1	---	K.FESEVYILSKDEGGR.H
1797.0350	1796.0277	1795.9325	53.1	359	374	0	---	K.MVVTLIHPIAMDDGLR.F + Oxidation (M)
1803.9918	1802.9845	1802.8799	58.0	60	75	0	---	R.GITINTSHVEYDTPTR.H
1965.1206	1964.1134	1963.9527	81.8	156	172	0	44	R.ELLSQYDFPGDDTPIVR.G
2117.3190	2116.3117	2116.1568	73.2	206	224	0	28	R.AIDKPFLLPIEDVFSISGR.G

No match to: 723.3202, 725.3349, 790.4618, 804.3175, 832.3504, 848.3077, 855.0634, 861.0816, 877.0593, 879.0642, 893.0384, 897.0395, 930.4819, 941.5245, 965.5432, 985.5503, 1001.5439, 1046.5846, 1179.6348, 1193.6685, 1219.5995, 1255.6486, 1269.6710, 1271.6285, 1277.6571, 1296.7322, 1320.6617, 1326.7448, 1348.7190, 1368.7612, 1450.7841, 1472.8880, 1475.8216, 1477.7796, 1494.8828, 1497.7996, 1509.8403, 1511.9038, 1527.8609, 1550.8947, 1598.9195, 1617.8806, 1631.8934, 1654.9031, 1707.9342, 1712.9042, 1733.0067, 1749.9856, 1755.9948, 1818.0212, 1866.0850, 1898.0605, 1915.0945, 1931.0593, 1947.0781, 1949.0662, 1967.1856, 1981.0932, 1988.1120, 1994.1406, 2005.1346, 2010.0916, 2139.2472, 2211.2249, 2229.2467, 2233.2426, 2271.2633, 2384.1999, 2399.2428, 2439.4144, 2451.4028, 2498.4046, 2615.4719, 2705.3847, 2808.6002, 3284.5981

11. EFTU2_ECOHS **Mass:** 43457 **Score:** 138 **Expect:** 5.3e-09 **Matches:** 11

Elongation factor Tu 2 OS=Escherichia coli O9:H4 (strain HS) OX=331112 GN=tuf2 PE=3 SV=1

Observed	Mr(expt)	Mr(calc)	ppm	Start	End	Miss	Ions	Peptide
837.5072	836.4999	836.4868	15.7	118	124	0	---	R.EHILLGR.Q
1027.6166	1026.6093	1026.5822	26.4	271	280	0	---	R.AGENVGVLLR.G
1214.6581	1213.6508	1213.6230	22.9	305	314	0	---	K.FESEVYILSK.D
1233.6701	1232.6629	1232.6091	43.6	326	334	0	15	K.GYRPQFYFR.T
1315.7305	1314.7232	1314.6060	89.1	254	264	1	---	K.STCTGVEMFRK.L
1574.9723	1573.9650	1573.9304	22.0	290	304	0	---	R.GQVLAKPGTIKPHTK.F
1728.9325	1727.9253	1727.8366	51.3	305	319	1	---	K.FESEVYILSKDEGGR.H
1797.0350	1796.0277	1795.9325	53.1	359	374	0	---	K.MVVTLIHPIAMDDGLR.F + Oxidation (M)
1803.9918	1802.9845	1802.8799	58.0	60	75	0	---	R.GITINTSHVEYDTPTR.H
1965.1206	1964.1134	1963.9527	81.8	156	172	0	44	R.ELLSQYDFPGDDTPIVR.G
2117.3190	2116.3117	2116.1568	73.2	206	224	0	28	R.AIDKPFLLPIEDVFSISGR.G

No match to: 723.3202, 725.3349, 790.4618, 804.3175, 832.3504, 848.3077, 855.0634, 861.0816, 877.0593, 879.0642, 893.0384, 897.0395, 930.4819, 941.5245, 965.5432, 985.5503, 1001.5439, 1046.5846, 1179.6348, 1193.6685, 1219.5995, 1255.6486, 1269.6710, 1271.6285, 1277.6571, 1296.7322, 1320.6617, 1326.7448, 1348.7190, 1368.7612, 1450.7841, 1472.8880, 1475.8216, 1477.7796, 1494.8828, 1497.7996, 1509.8403, 1511.9038, 1527.8609, 1550.8947, 1598.9195, 1617.8806, 1631.8934, 1654.9031, 1707.9342, 1712.9042, 1733.0067, 1749.9856, 1755.9948, 1818.0212, 1866.0850, 1898.0605, 1915.0945, 1931.0593, 1947.0781, 1949.0662, 1967.1856, 1981.0932, 1988.1120, 1994.1406, 2005.1346, 2010.0916, 2139.2472, 2211.2249, 2229.2467, 2233.2426, 2271.2633, 2384.1999, 2399.2428, 2439.4144, 2451.4028, 2498.4046, 2615.4719, 2705.3847, 2808.6002, 3284.5981

12. EFTU2_ECOK1 **Mass:** 43457 **Score:** 138 **Expect:** 5.3e-09 **Matches:** 11

Elongation factor Tu 2 OS=Escherichia coli O1:K1 / APEC OX=405955 GN=tuf2 PE=3 SV=1

Observed	Mr(expt)	Mr(calc)	ppm	Start	End	Miss	Ions	Peptide
837.5072	836.4999	836.4868	15.7	118	124	0	---	R.EHILLGR.Q
1027.6166	1026.6093	1026.5822	26.4	271	280	0	---	R.AGENVGVLLR.G
1214.6581	1213.6508	1213.6230	22.9	305	314	0	---	K.FESEVYILSK.D
1233.6701	1232.6629	1232.6091	43.6	326	334	0	15	K.GYRPQFYFR.T
1315.7305	1314.7232	1314.6060	89.1	254	264	1	---	K.STCTGVEMFRK.L
1574.9723	1573.9650	1573.9304	22.0	290	304	0	---	R.GQVLAKPGTIKPHTK.F
1728.9325	1727.9253	1727.8366	51.3	305	319	1	---	K.FESEVYILSKDEGGR.H
1797.0350	1796.0277	1795.9325	53.1	359	374	0	---	K.MVVTLIHPIAMDDGLR.F + Oxidation (M)
1803.9918	1802.9845	1802.8799	58.0	60	75	0	---	R.GITINTSHVEYDTPTR.H
1965.1206	1964.1134	1963.9527	81.8	156	172	0	44	R.ELLSQYDFPGDDTPIVR.G
2117.3190	2116.3117	2116.1568	73.2	206	224	0	28	R.AIDKPFLLPIEDVFSISGR.G

No match to: 723.3202, 725.3349, 790.4618, 804.3175, 832.3504, 848.3077, 855.0634, 861.0816, 877.0593, 879.0642, 893.0384, 897.0395, 930.4819, 941.5245, 965.5432, 985.5503, 1001.5439, 1046.5846, 1179.6348, 1193.6685, 1219.5995, 1255.6486, 1269.6710, 1271.6285, 1277.6571, 1296.7322, 1320.6617, 1326.7448, 1348.7190, 1368.7612, 1450.7841, 1472.8880, 1475.8216, 1477.7796, 1494.8828, 1497.7996, 1509.8403, 1511.9038, 1527.8609, 1550.8947, 1598.9195, 1617.8806, 1631.8934, 1654.9031, 1707.9342, 1712.9042, 1733.0067, 1749.9856, 1755.9948, 1818.0212, 1866.0850, 1898.0605, 1915.0945, 1931.0593, 1947.0781, 1949.0662, 1967.1856, 1981.0932, 1988.1120, 1994.1406, 2005.1346, 2010.0916, 2139.2472, 2211.2249, 2229.2467, 2233.2426, 2271.2633, 2384.1999, 2399.2428, 2439.4144, 2451.4028, 2498.4046, 2615.4719, 2705.3847, 2808.6002, 3284.5981

13. EFTU2_ECOL5 **Mass:** 43457 **Score:** 138 **Expect:** 5.3e-09 **Matches:** 11

Elongation factor Tu 2 OS=Escherichia coli O6:K15:H31 (strain 536 / UPEC) OX=362663 GN=tuf2 PE=3 SV=1

Observed	Mr(expt)	Mr(calc)	ppm	Start	End	Miss	Ions	Peptide
837.5072	836.4999	836.4868	15.7	118	124	0	---	R.EHILLGR.Q
1027.6166	1026.6093	1026.5822	26.4	271	280	0	---	R.AGENVGVLLR.G
1214.6581	1213.6508	1213.6230	22.9	305	314	0	---	K.FESEVYILSK.D
1233.6701	1232.6629	1232.6091	43.6	326	334	0	15	K.GYRPQFYFR.T
1315.7305	1314.7232	1314.6060	89.1	254	264	1	---	K.STCTGVEMFRK.L
1574.9723	1573.9650	1573.9304	22.0	290	304	0	---	R.GQVLAKPGTIKPHTK.F
1728.9325	1727.9253	1727.8366	51.3	305	319	1	---	K.FESEVYILSKDEGGR.H
1797.0350	1796.0277	1795.9325	53.1	359	374	0	---	K.MVVTLIHPIAMDDGLR.F + Oxidation (M)
1803.9918	1802.9845	1802.8799	58.0	60	75	0	---	R.GITINTSHVEYDTPTR.H
1965.1206	1964.1134	1963.9527	81.8	156	172	0	44	R.ELLSQYDFPGDDTPIVR.G
2117.3190	2116.3117	2116.1568	73.2	206	224	0	28	R.AIDKPFLLPIEDVFSISGR.G

No match to: 723.3202, 725.3349, 790.4618, 804.3175, 832.3504, 848.3077, 855.0634, 861.0816, 877.0593, 879.0642, 893.0384, 897.0395, 930.4819, 941.5245, 965.5432, 985.5503, 1001.5439, 1046.5846, 1179.6348, 1193.6685, 1219.5995, 1255.6486, 1269.6710, 1271.6285, 1277.6571, 1296.7322, 1320.6617, 1326.7448, 1348.7190, 1368.7612, 1450.7841, 1472.8880, 1475.8216, 1477.7796, 1494.8828, 1497.7996, 1509.8403, 1511.9038, 1527.8609, 1550.8947, 1598.9195, 1617.8806, 1631.8934, 1654.9031, 1707.9342, 1712.9042, 1733.0067, 1749.9856, 1755.9948, 1818.0212, 1866.0850, 1898.0605, 1915.0945, 1931.0593, 1947.0781, 1949.0662, 1967.1856, 1981.0932, 1988.1120, 1994.1406, 2005.1346, 2010.0916, 2139.2472, 2211.2249, 2229.2467, 2233.2426, 2271.2633, 2384.1999, 2399.2428, 2439.4144, 2451.4028, 2498.4046, 2615.4719, 2705.3847, 2808.6002, 3284.5981

14. EFTU2_ECOLC **Mass:** 43457 **Score:** 138 **Expect:** 5.3e-09 **Matches:** 11

Elongation factor Tu 2 OS=Escherichia coli (strain ATCC 8739 / DSM 1576 / Crooks) OX=481805 GN=tuf2 PE=3 SV=1

Observed	Mr(expt)	Mr(calc)	ppm	Start	End	Miss	Ions	Peptide
837.5072	836.4999	836.4868	15.7	118	124	0	---	R.EHILLGR.Q
1027.6166	1026.6093	1026.5822	26.4	271	280	0	---	R.AGENVGVLLR.G
1214.6581	1213.6508	1213.6230	22.9	305	314	0	---	K.FESEVYILSK.D
1233.6701	1232.6629	1232.6091	43.6	326	334	0	15	K.GYRPQFYFR.T
1315.7305	1314.7232	1314.6060	89.1	254	264	1	---	K.STCTGVEMFRK.L
1574.9723	1573.9650	1573.9304	22.0	290	304	0	---	R.GQVLAKPGTIKPHTK.F
1728.9325	1727.9253	1727.8366	51.3	305	319	1	---	K.FESEVYILSKDEGGR.H
1797.0350	1796.0277	1795.9325	53.1	359	374	0	---	K.MVVTLIHPIAMDDGLR.F + Oxidation (M)
1803.9918	1802.9845	1802.8799	58.0	60	75	0	---	R.GITINTSHVEYDTPTR.H
1965.1206	1964.1134	1963.9527	81.8	156	172	0	44	R.ELLSQYDFPGDDTPIVR.G
2117.3190	2116.3117	2116.1568	73.2	206	224	0	28	R.AIDKPFLLPIEDVFSISGR.G

No match to: 723.3202, 725.3349, 790.4618, 804.3175, 832.3504, 848.3077, 855.0634, 861.0816, 877.0593, 879.0642, 893.0384, 897.0395, 930.4819, 941.5245, 965.5432, 985.5503, 1001.5439, 1046.5846, 1179.6348, 1193.6685, 1219.5995, 1255.6486, 1269.6710, 1271.6285, 1277.6571, 1296.7322, 1320.6617, 1326.7448, 1348.7190, 1368.7612, 1450.7841, 1472.8880, 1475.8216, 1477.7796, 1494.8828, 1497.7996, 1509.8403, 1511.9038, 1527.8609, 1550.8947, 1598.9195, 1617.8806, 1631.8934, 1654.9031, 1707.9342, 1712.9042, 1733.0067, 1749.9856, 1755.9948, 1818.0212, 1866.0850, 1898.0605, 1915.0945, 1931.0593, 1947.0781, 1949.0662, 1967.1856, 1981.0932, 1988.1120, 1994.1406, 2005.1346, 2010.0916, 2139.2472, 2211.2249, 2229.2467, 2233.2426, 2271.2633, 2384.1999, 2399.2428, 2439.4144, 2451.4028, 2498.4046, 2615.4719, 2705.3847, 2808.6002, 3284.5981

15. EFTU2_ECOLI **Mass:** 43457 **Score:** 138 **Expect:** 5.3e-09 **Matches:** 11

Elongation factor Tu 2 OS=Escherichia coli (strain K12) OX=83333 GN=tufB PE=1 SV=1

Observed	Mr(expt)	Mr(calc)	ppm	Start	End	Miss	Ions	Peptide
837.5072	836.4999	836.4868	15.7	118	124	0	---	R.EHILLGR.Q
1027.6166	1026.6093	1026.5822	26.4	271	280	0	---	R.AGENVGVLLR.G
1214.6581	1213.6508	1213.6230	22.9	305	314	0	---	K.FESEVYILSK.D
1233.6701	1232.6629	1232.6091	43.6	326	334	0	15	K.GYRPQFYFR.T
1315.7305	1314.7232	1314.6060	89.1	254	264	1	---	K.STCTGVEMFRK.L
1574.9723	1573.9650	1573.9304	22.0	290	304	0	---	R.GQVLAKPGTIKPHTK.F
1728.9325	1727.9253	1727.8366	51.3	305	319	1	---	K.FESEVYILSKDEGGR.H
1797.0350	1796.0277	1795.9325	53.1	359	374	0	---	K.MVVTLIHPIAMDDGLR.F + Oxidation (M)
1803.9918	1802.9845	1802.8799	58.0	60	75	0	---	R.GITINTSHVEYDTPTR.H
1965.1206	1964.1134	1963.9527	81.8	156	172	0	44	R.ELLSQYDFPGDDTPIVR.G
2117.3190	2116.3117	2116.1568	73.2	206	224	0	28	R.AIDKPFLLPIEDVFSISGR.G

No match to: 723.3202, 725.3349, 790.4618, 804.3175, 832.3504, 848.3077, 855.0634, 861.0816, 877.0593, 879.0642, 893.0384, 897.0395, 930.4819, 941.5245, 965.5432, 985.5503, 1001.5439, 1046.5846, 1179.6348, 1193.6685, 1219.5995, 1255.6486, 1269.6710, 1271.6285, 1277.6571, 1296.7322, 1320.6617, 1326.7448, 1348.7190, 1368.7612, 1450.7841, 1472.8880, 1475.8216, 1477.7796, 1494.8828, 1497.7996, 1509.8403, 1511.9038, 1527.8609, 1550.8947, 1598.9195, 1617.8806, 1631.8934, 1654.9031, 1707.9342, 1712.9042, 1733.0067, 1749.9856, 1755.9948, 1818.0212, 1866.0850, 1898.0605, 1915.0945, 1931.0593, 1947.0781, 1949.0662, 1967.1856, 1981.0932, 1988.1120, 1994.1406, 2005.1346, 2010.0916, 2139.2472, 2211.2249, 2229.2467, 2233.2426, 2271.2633, 2384.1999, 2399.2428, 2439.4144, 2451.4028, 2498.4046, 2615.4719, 2705.3847, 2808.6002, 3284.5981

16. EFTU2_ECOUT **Mass:** 43457 **Score:** 138 **Expect:** 5.3e-09 **Matches:** 11

Elongation factor Tu 2 OS=Escherichia coli (strain UTI89 / UPEC) OX=364106 GN=tuf2 PE=3 SV=2

Observed	Mr(expt)	Mr(calc)	ppm	Start	End	Miss	Ions	Peptide
837.5072	836.4999	836.4868	15.7	118	124	0	---	R.EHILLGR.Q
1027.6166	1026.6093	1026.5822	26.4	271	280	0	---	R.AGENVGVLLR.G
1214.6581	1213.6508	1213.6230	22.9	305	314	0	---	K.FESEVYILSK.D
1233.6701	1232.6629	1232.6091	43.6	326	334	0	15	K.GYRPQFYFR.T
1315.7305	1314.7232	1314.6060	89.1	254	264	1	---	K.STCTGVEMFRK.L
1574.9723	1573.9650	1573.9304	22.0	290	304	0	---	R.GQVLAKPGTIKPHTK.F
1728.9325	1727.9253	1727.8366	51.3	305	319	1	---	K.FESEVYILSKDEGGR.H
1797.0350	1796.0277	1795.9325	53.1	359	374	0	---	K.MVVTLIHPIAMDDGLR.F + Oxidation (M)
1803.9918	1802.9845	1802.8799	58.0	60	75	0	---	R.GITINTSHVEYDTPTR.H
1965.1206	1964.1134	1963.9527	81.8	156	172	0	44	R.ELLSQYDFPGDDTPIVR.G
2117.3190	2116.3117	2116.1568	73.2	206	224	0	28	R.AIDKPFLLPIEDVFSISGR.G

No match to: 723.3202, 725.3349, 790.4618, 804.3175, 832.3504, 848.3077, 855.0634, 861.0816, 877.0593, 879.0642, 893.0384, 897.0395, 930.4819, 941.5245, 965.5432, 985.5503, 1001.5439, 1046.5846, 1179.6348, 1193.6685, 1219.5995, 1255.6486, 1269.6710, 1271.6285, 1277.6571, 1296.7322, 1320.6617, 1326.7448, 1348.7190, 1368.7612, 1450.7841, 1472.8880, 1475.8216, 1477.7796, 1494.8828, 1497.7996, 1509.8403, 1511.9038, 1527.8609, 1550.8947, 1598.9195, 1617.8806, 1631.8934, 1654.9031, 1707.9342, 1712.9042, 1733.0067, 1749.9856, 1755.9948, 1818.0212, 1866.0850, 1898.0605, 1915.0945, 1931.0593, 1947.0781, 1949.0662, 1967.1856, 1981.0932, 1988.1120, 1994.1406, 2005.1346, 2010.0916, 2139.2472, 2211.2249, 2229.2467, 2233.2426, 2271.2633, 2384.1999, 2399.2428, 2439.4144, 2451.4028, 2498.4046, 2615.4719, 2705.3847, 2808.6002, 3284.5981

17. EFTU2_SHIF8 **Mass:** 43457 **Score:** 138 **Expect:** 5.3e-09 **Matches:** 11

Elongation factor Tu 2 OS=Shigella flexneri serotype 5b (strain 8401) OX=373384 GN=tuf2 PE=3 SV=1

Observed	Mr(expt)	Mr(calc)	ppm	Start	End	Miss	Ions	Peptide
837.5072	836.4999	836.4868	15.7	118	124	0	---	R.EHILLGR.Q
1027.6166	1026.6093	1026.5822	26.4	271	280	0	---	R.AGENVGVLLR.G
1214.6581	1213.6508	1213.6230	22.9	305	314	0	---	K.FESEVYILSK.D
1233.6701	1232.6629	1232.6091	43.6	326	334	0	15	K.GYRPQFYFR.T
1315.7305	1314.7232	1314.6060	89.1	254	264	1	---	K.STCTGVEMFRK.L
1574.9723	1573.9650	1573.9304	22.0	290	304	0	---	R.GQVLAKPGTIKPHTK.F
1728.9325	1727.9253	1727.8366	51.3	305	319	1	---	K.FESEVYILSKDEGGR.H
1797.0350	1796.0277	1795.9325	53.1	359	374	0	---	K.MVVTLIHPIAMDDGLR.F + Oxidation (M)
1803.9918	1802.9845	1802.8799	58.0	60	75	0	---	R.GITINTSHVEYDTPTR.H
1965.1206	1964.1134	1963.9527	81.8	156	172	0	44	R.ELLSQYDFPGDDTPIVR.G
2117.3190	2116.3117	2116.1568	73.2	206	224	0	28	R.AIDKPFLLPIEDVFSISGR.G

No match to: 723.3202, 725.3349, 790.4618, 804.3175, 832.3504, 848.3077, 855.0634, 861.0816, 877.0593, 879.0642, 893.0384, 897.0395, 930.4819, 941.5245, 965.5432, 985.5503, 1001.5439, 1046.5846, 1179.6348, 1193.6685, 1219.5995, 1255.6486, 1269.6710, 1271.6285, 1277.6571, 1296.7322, 1320.6617, 1326.7448, 1348.7190, 1368.7612, 1450.7841, 1472.8880, 1475.8216, 1477.7796, 1494.8828, 1497.7996, 1509.8403, 1511.9038, 1527.8609, 1550.8947, 1598.9195, 1617.8806, 1631.8934, 1654.9031, 1707.9342, 1712.9042, 1733.0067, 1749.9856, 1755.9948, 1818.0212, 1866.0850, 1898.0605, 1915.0945, 1931.0593, 1947.0781, 1949.0662, 1967.1856, 1981.0932, 1988.1120, 1994.1406, 2005.1346, 2010.0916, 2139.2472, 2211.2249, 2229.2467, 2233.2426, 2271.2633, 2384.1999, 2399.2428, 2439.4144, 2451.4028, 2498.4046, 2615.4719, 2705.3847, 2808.6002, 3284.5981

18. EFTU2_SHISS **Mass:** 43457 **Score:** 138 **Expect:** 5.3e-09 **Matches:** 11

Elongation factor Tu 2 OS=Shigella sonnei (strain Ss046) OX=300269 GN=tuf2 PE=3 SV=1

Observed	Mr(expt)	Mr(calc)	ppm	Start	End	Miss	Ions	Peptide
837.5072	836.4999	836.4868	15.7	118	124	0	---	R.EHILLGR.Q
1027.6166	1026.6093	1026.5822	26.4	271	280	0	---	R.AGENVGVLLR.G
1214.6581	1213.6508	1213.6230	22.9	305	314	0	---	K.FESEVYILSK.D
1233.6701	1232.6629	1232.6091	43.6	326	334	0	15	K.GYRPQFYFR.T
1315.7305	1314.7232	1314.6060	89.1	254	264	1	---	K.STCTGVEMFRK.L
1574.9723	1573.9650	1573.9304	22.0	290	304	0	---	R.GQVLAKPGTIKPHTK.F
1728.9325	1727.9253	1727.8366	51.3	305	319	1	---	K.FESEVYILSKDEGGR.H
1797.0350	1796.0277	1795.9325	53.1	359	374	0	---	K.MVVTLIHPIAMDDGLR.F + Oxidation (M)
1803.9918	1802.9845	1802.8799	58.0	60	75	0	---	R.GITINTSHVEYDTPTR.H
1965.1206	1964.1134	1963.9527	81.8	156	172	0	44	R.ELLSQYDFPGDDTPIVR.G
2117.3190	2116.3117	2116.1568	73.2	206	224	0	28	R.AIDKPFLLPIEDVFSISGR.G

No match to: 723.3202, 725.3349, 790.4618, 804.3175, 832.3504, 848.3077, 855.0634, 861.0816, 877.0593, 879.0642, 893.0384, 897.0395, 930.4819, 941.5245, 965.5432, 985.5503, 1001.5439, 1046.5846, 1179.6348, 1193.6685, 1219.5995, 1255.6486, 1269.6710, 1271.6285, 1277.6571, 1296.7322, 1320.6617, 1326.7448, 1348.7190, 1368.7612, 1450.7841, 1472.8880, 1475.8216, 1477.7796, 1494.8828, 1497.7996, 1509.8403, 1511.9038, 1527.8609, 1550.8947, 1598.9195, 1617.8806, 1631.8934, 1654.9031, 1707.9342, 1712.9042, 1733.0067, 1749.9856, 1755.9948, 1818.0212, 1866.0850, 1898.0605, 1915.0945, 1931.0593, 1947.0781, 1949.0662, 1967.1856, 1981.0932, 1988.1120, 1994.1406, 2005.1346, 2010.0916, 2139.2472, 2211.2249, 2229.2467, 2233.2426, 2271.2633, 2384.1999, 2399.2428, 2439.4144, 2451.4028, 2498.4046, 2615.4719, 2705.3847, 2808.6002, 3284.5981

19. EFTU_ECO57 **Mass:** 43457 **Score:** 138 **Expect:** 5.3e-09 **Matches:** 11

Elongation factor Tu OS=Escherichia coli O157:H7 OX=83334 GN=tufA PE=3 SV=2

Observed	Mr(expt)	Mr(calc)	ppm	Start	End	Miss	Ions	Peptide
837.5072	836.4999	836.4868	15.7	118	124	0	---	R.EHILLGR.Q
1027.6166	1026.6093	1026.5822	26.4	271	280	0	---	R.AGENVGVLLR.G

1214.6581	1213.6508	1213.6230	22.9	305 - 314	0	---	K.FESEVYILSK.D
1233.6701	1232.6629	1232.6091	43.6	326 - 334	0	15	K.GYRPQFYFR.T
1315.7305	1314.7232	1314.6060	89.1	254 - 264	1	---	K.STCTGVEMFRK.L
1574.9723	1573.9650	1573.9304	22.0	290 - 304	0	---	R.GQVLAKPGTIKPHTK.F
1728.9325	1727.9253	1727.8366	51.3	305 - 319	1	---	K.FESEVYILSKDEGGR.H
1797.0350	1796.0277	1795.9325	53.1	359 - 374	0	---	K.MVVTLIHPIAMDDGLR.F + Oxidation (M)
1803.9918	1802.9845	1802.8799	58.0	60 - 75	0	---	R.GITINTSHVEYDTPTR.H
1965.1206	1964.1134	1963.9527	81.8	156 - 172	0	44	R.ELLSQYDFPGDDTPIVR.G
2117.3190	2116.3117	2116.1568	73.2	206 - 224	0	28	R.AIDKPFLLPIEDVFSISGR.G

No match to: 723.3202, 725.3349, 790.4618, 804.3175, 832.3504, 848.3077, 855.0634, 861.0816, 877.0593, 879.0642, 893.0384, 897.0395, 930.4819, 941.5245, 965.5432, 985.5503, 1001.5439, 1046.5846, 1179.6348, 1193.6685, 1219.5995, 1255.6486, 1269.6710, 1271.6285, 1277.6571, 1296.7322, 1320.6617, 1326.7448, 1348.7190, 1368.7612, 1450.7841, 1472.8880, 1475.8216, 1477.7796, 1494.8828, 1497.7996, 1509.8403, 1511.9038, 1527.8609, 1550.8947, 1598.9195, 1617.8806, 1631.8934, 1654.9031, 1707.9342, 1712.9042, 1733.0067, 1749.9856, 1755.9948, 1818.0212, 1866.0850, 1898.0605, 1915.0945, 1931.0593, 1947.0781, 1949.0662, 1967.1856, 1981.0932, 1988.1120, 1994.1406, 2005.1346, 2010.0916, 2139.2472, 2211.2249, 2229.2467, 2233.2426, 2271.2633, 2384.1999, 2399.2428, 2439.4144, 2451.4028, 2498.4046, 2615.4719, 2705.3847, 2808.6002, 3284.5981

20. EFTU_ECOL6 **Mass:** 43457 **Score:** 138 **Expect:** 5.3e-09 **Matches:** 11

Elongation factor Tu OS=Escherichia coli O6:H1 (strain CFT073 / ATCC 700928 / UPEC) OX=199310 GN=tufA PE=3 SV=2

Observed	Mr(expt)	Mr(calc)	ppm	Start - End	Miss	Ions	Peptide
837.5072	836.4999	836.4868	15.7	118 - 124	0	---	R.EHILLGR.Q
1027.6166	1026.6093	1026.5822	26.4	271 - 280	0	---	R.AGENVGVLLR.G
1214.6581	1213.6508	1213.6230	22.9	305 - 314	0	---	K.FESEVYILSK.D
1233.6701	1232.6629	1232.6091	43.6	326 - 334	0	15	K.GYRPQFYFR.T
1315.7305	1314.7232	1314.6060	89.1	254 - 264	1	---	K.STCTGVEMFRK.L
1574.9723	1573.9650	1573.9304	22.0	290 - 304	0	---	R.GQVLAKPGTIKPHTK.F
1728.9325	1727.9253	1727.8366	51.3	305 - 319	1	---	K.FESEVYILSKDEGGR.H
1797.0350	1796.0277	1795.9325	53.1	359 - 374	0	---	K.MVVTLIHPIAMDDGLR.F + Oxidation (M)
1803.9918	1802.9845	1802.8799	58.0	60 - 75	0	---	R.GITINTSHVEYDTPTR.H
1965.1206	1964.1134	1963.9527	81.8	156 - 172	0	44	R.ELLSQYDFPGDDTPIVR.G
2117.3190	2116.3117	2116.1568	73.2	206 - 224	0	28	R.AIDKPFLLPIEDVFSISGR.G

No match to: 723.3202, 725.3349, 790.4618, 804.3175, 832.3504, 848.3077, 855.0634, 861.0816, 877.0593, 879.0642, 893.0384, 897.0395, 930.4819, 941.5245, 965.5432, 985.5503, 1001.5439, 1046.5846, 1179.6348, 1193.6685, 1219.5995, 1255.6486, 1269.6710, 1271.6285, 1277.6571, 1296.7322, 1320.6617, 1326.7448, 1348.7190, 1368.7612, 1450.7841, 1472.8880, 1475.8216, 1477.7796, 1494.8828, 1497.7996, 1509.8403, 1511.9038, 1527.8609, 1550.8947, 1598.9195, 1617.8806, 1631.8934, 1654.9031, 1707.9342, 1712.9042, 1733.0067, 1749.9856, 1755.9948, 1818.0212, 1866.0850, 1898.0605, 1915.0945, 1931.0593, 1947.0781, 1949.0662, 1967.1856, 1981.0932, 1988.1120, 1994.1406, 2005.1346, 2010.0916, 2139.2472, 2211.2249, 2229.2467, 2233.2426, 2271.2633, 2384.1999, 2399.2428, 2439.4144, 2451.4028, 2498.4046, 2615.4719, 2705.3847, 2808.6002, 3284.5981

Search Parameters

Type of search : MS/MS Ion Search
Enzyme : Trypsin
Fixed modifications : Carbamidomethyl (C)
Variable modifications : Oxidation (M)
Mass values : Monoisotopic
Protein Mass : Unrestricted
Peptide Mass Tolerance : ± 150 ppm
Fragment Mass Tolerance: ± 2 Da
Max Missed Cleavages : 1
Instrument type : MALDI-TOF-TOF
Query1 (723.3202,1+): <no title>
Query2 (725.3349,1+): <no title>
Query3 (790.4618,1+): <no title>
Query4 (804.3175,1+): <no title>
Query5 (832.3504,1+): <no title>
Query6 (837.5072,1+): <no title>
Query7 (848.3077,1+): <no title>
Query8 (855.0634,1+): <no title>
Query9 (861.0816,1+): <no title>
Query10 (877.0593,1+): <no title>
Query11 (879.0642,1+): <no title>
Query12 (893.0384,1+): <no title>
Query13 (897.0395,1+): <no title>
Query14 (930.4819,1+): <no title>
Query15 (941.5245,1+): <no title>
Query16 (965.5432,1+): <no title>
Query17 (985.5503,1+): <no title>
Query18 (1001.5439,1+): <no title>
Query19 (1027.6166,1+): <no title>
Query20 (1046.5846,1+): <no title>
Query21 (1179.6348,1+): <no title>
Query22 (1193.6685,1+): <no title>
Query23 (1214.6581,1+): <no title>
Query24 (1219.5995,1+): <no title>
Query25 (1233.6701,1+): <no title>
Query26 (1255.6486,1+): <no title>
Query27 (1269.6710,1+): <no title>
Query28 (1271.6285,1+): <no title>
Query29 (1277.6571,1+): <no title>
Query30 (1296.7322,1+): <no title>
Query31 (1315.7305,1+): <no title>
Query32 (1320.6617,1+): <no title>
Query33 (1326.7448,1+): <no title>
Query34 (1348.7190,1+): <no title>
Query35 (1368.7612,1+): <no title>
Query36 (1450.7841,1+): <no title>
Query37 (1472.8880,1+): <no title>
Query38 (1475.8216,1+): <no title>
Query39 (1477.7796,1+): <no title>
Query40 (1494.8828,1+): <no title>
Query41 (1497.7996,1+): <no title>
Query42 (1509.8403,1+): <no title>
Query43 (1511.9038,1+): <no title>
Query44 (1527.8609,1+): <no title>
Query45 (1550.8947,1+): <no title>
Query46 (1574.9723,1+): <no title>
Query47 (1598.9195,1+): <no title>
Query48 (1617.8806,1+): <no title>

```
Query49 (1631.8934,1+): <no title>
Query50 (1654.9031,1+): <no title>
Query51 (1707.9342,1+): <no title>
Query52 (1712.9042,1+): <no title>
Query53 (1728.9325,1+): <no title>
Query54 (1733.0067,1+): <no title>
Query55 (1749.9856,1+): <no title>
Query56 (1755.9948,1+): <no title>
Query57 (1797.0350,1+): <no title>
Query58 (1803.9918,1+): <no title>
Query59 (1818.0212,1+): <no title>
Query60 (1866.0850,1+): <no title>
Query61 (1898.0605,1+): <no title>
Query62 (1915.0945,1+): <no title>
Query63 (1931.0593,1+): <no title>
Query64 (1947.0781,1+): <no title>
Query65 (1949.0662,1+): <no title>
Query66 (1965.1206,1+): <no title>
Query67 (1967.1856,1+): <no title>
Query68 (1981.0932,1+): <no title>
Query69 (1988.1120,1+): <no title>
Query70 (1994.1406,1+): <no title>
Query71 (2005.1346,1+): <no title>
Query72 (2010.0916,1+): <no title>
Query73 (2117.3190,1+): <no title>
Query74 (2139.2472,1+): <no title>
Query75 (2211.2249,1+): <no title>
Query76 (2229.2467,1+): <no title>
Query77 (2233.2426,1+): <no title>
Query78 (2271.2633,1+): <no title>
Query79 (2384.1999,1+): <no title>
Query80 (2399.2428,1+): <no title>
Query81 (2439.4144,1+): <no title>
Query82 (2451.4028,1+): <no title>
Query83 (2498.4046,1+): <no title>
Query84 (2615.4719,1+): <no title>
Query85 (2705.3847,1+): <no title>
Query86 (2808.6002,1+): <no title>
Query87 (3284.5981,1+): <no title>
```

**Mascot:** http://www.matrixscience.com/

MALDI MS MS Data Sheet For Blood Isolate

C:\Users\madhurekha\Desktop\MALDI_Data\16th batch data 070718\BHU\2\0_B2\1\1075.6476.LIFT\1SRef

Comment 1

Comment 2

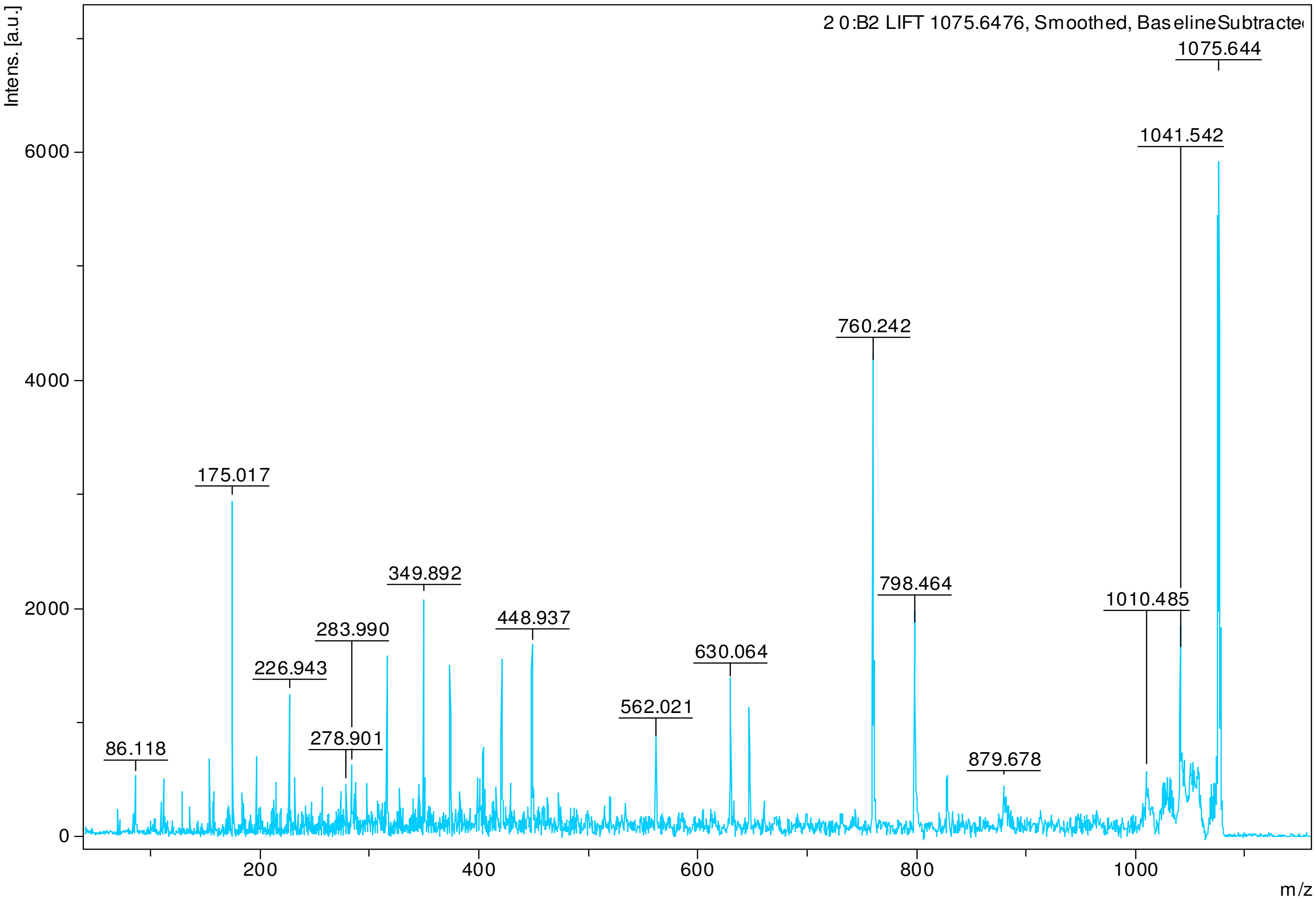

m/z	S/N	Quality Fac.	Res.	Intens.	Area
70.067	6.5	315	354	271.28	59
72.109	3.7	166	282	154.67	43
86.118	12.0	3410	385	539.50	133
104.032	3.3	133	629	153.14	28
110.020	6.5	1427	597	303.61	63
112.061	10.5	1726	547	490.46	113
115.079	3.1	51	763	147.28	25
129.058	8.3	1795	701	392.29	82
136.008	5.3	587	754	259.11	53
153.994	14.1	3801	936	694.28	131
158.014	8.1	1113	940	404.06	78
169.011	3.0	74	770	156.49	40
170.998	5.2	61	827	270.92	65
175.017	55.8	31800	944	2960.91	642
183.957	7.2	123	939	395.64	91
191.965	3.5	31	1349	199.70	34
196.973	12.3	1438	1052	715.12	159
200.967	4.3	69	912	254.96	67
210.968	4.3	131	1058	264.94	63
213.003	5.2	177	962	327.58	87
214.965	7.6	673	1095	480.64	113
218.978	4.0	256	1178	255.81	57
226.943	19.0	6603	1194	1267.92	290
231.985	7.7	3446	1075	517.95	135
246.963	4.3	47	1236	317.11	77
256.977	5.9	391	933	445.94	151
273.970	5.0	99	1146	418.39	124
278.901	5.6	319	1204	474.85	137
283.990	7.4	1635	1028	645.45	222
285.916	4.1	54	1081	363.36	120
287.938	5.5	317	1044	492.20	169
297.936	5.1	87	1245	482.50	145
315.925	15.6	15405	1271	1584.39	499
327.968	4.2	55	1195	449.94	158
344.925	4.2	35	1326	477.57	160
349.892	18.3	16791	1293	2121.79	742
373.912	12.3	5576	1278	1539.24	590
398.949	3.9	184	1049	530.41	268

C:\Users\madhurekha\Desktop\MALDI_Data\16th batch data 070718\BHU\2\0_B2\1\1075.6476.LIFT\1SRef

m/z	S/N	Quality Fac.	Res.	Intens.	Area
400.918	3.6	83	1114	488.35	234
403.932	6.0	287	1319	817.26	333
415.998	3.1	97	958	436.01	254
420.974	11.0	15256	1258	1544.85	695
428.909	3.4	83	1402	485.81	201
448.937	11.6	14331	1255	1691.94	826
562.021	5.7	467	1227	846.85	563
630.064	9.5	4485	1351	1368.90	970
647.094	8.3	2333	1402	1189.74	843
760.242	30.3	76102	1462	4140.41	3525
798.464	13.6	5889	1278	1845.77	1931
828.324	4.0	582	1439	533.65	523
879.678	3.6	33	1478	505.27	527
1010.485	5.3	189	1264	601.90	908
1029.275	4.4	197	1766	465.09	517
1031.448	5.1	98	1482	537.17	714
1041.542	16.1	5257	1476	1625.95	2204
1044.635	5.3	173	1208	528.01	879
1050.224	6.1	104	1239	583.09	955
1052.621	5.9	109	1170	554.77	965
1055.007	5.7	95	1172	545.89	952
1057.468	6.0	188	1175	575.27	1003
1068.607	5.4	88	1890	485.64	539
1075.644	75.9	4039	2819	6682.54	5023
1077.875	6.1	99	2124	534.53	534

C:\Users\madhurekha\Desktop\MALDI_Data\16th batch data 070718\BHU\2\0_B2\1\1545.9178.LIFT\1SRef

Comment 1

Comment 2

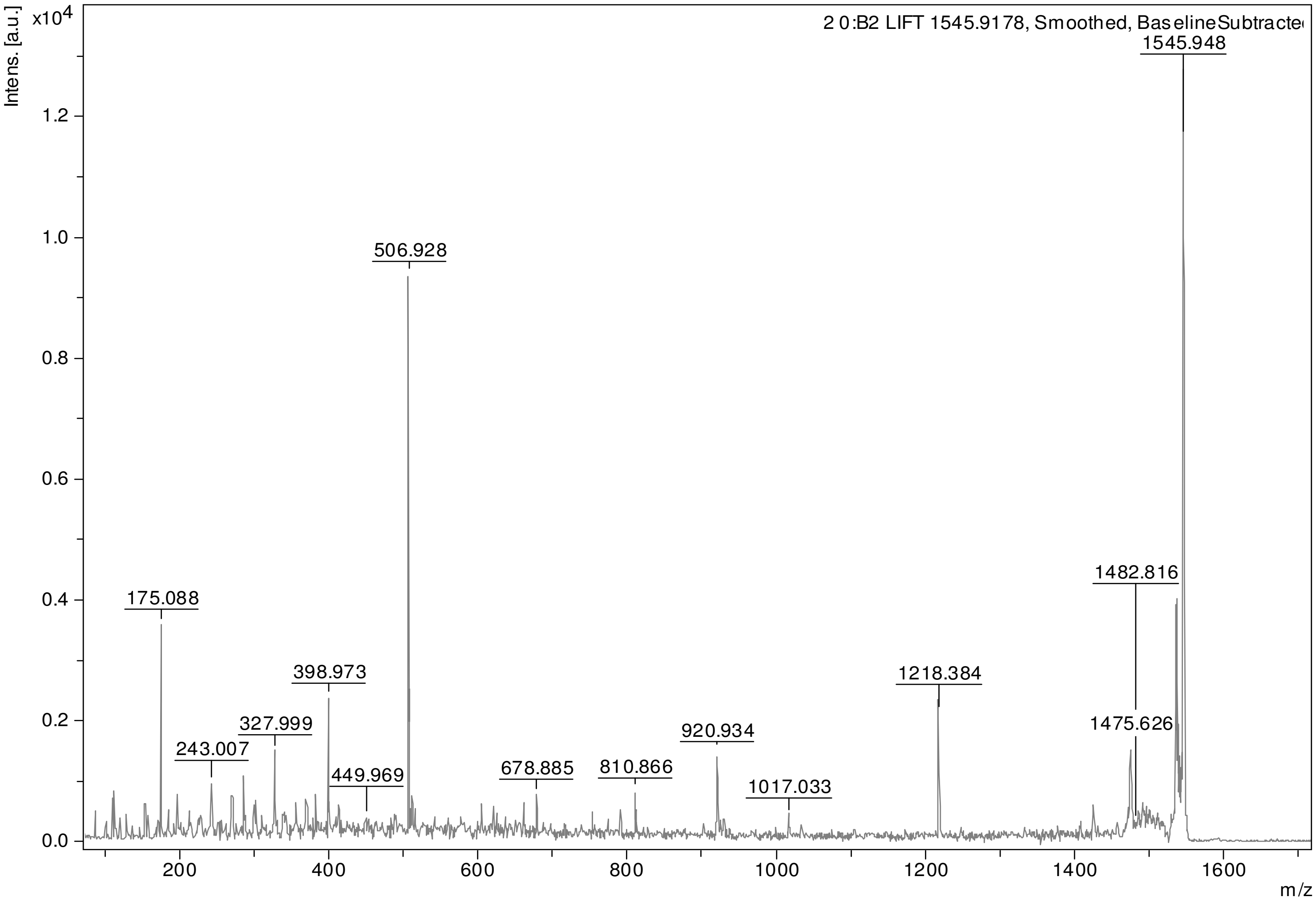

m/z	S/N	Quality Fac.	Res.	Intens.	Area
70.067	5.9	167	613	319.40	40
86.149	8.5	621	501	465.31	89
101.112	5.8	273	849	324.21	43
110.093	13.0	12421	972	725.72	92
112.104	14.3	1623	858	802.01	118
120.097	6.9	284	1029	382.17	50
129.106	8.1	639	1027	455.92	65
136.086	4.5	358	1084	250.78	36
147.081	4.2	292	1079	230.69	36
154.074	11.2	679	1040	626.85	106
158.083	7.6	1365	1091	422.57	70
169.065	4.2	95	1381	236.18	34
171.084	6.5	63	1345	362.39	54
175.088	64.3	28130	1324	3601.47	557
185.081	9.6	884	1196	531.95	97
192.054	5.3	124	1218	294.86	55
197.071	14.6	991	1344	817.70	142
201.057	5.4	48	1204	305.23	60
205.059	4.1	38	1230	235.58	47
213.069	9.2	353	1355	523.77	98
215.051	5.4	53	1239	308.59	64
226.038	7.4	62	1425	427.16	82
228.046	7.9	34	1469	457.40	86
230.020	5.6	169	1374	325.56	66
235.035	3.6	32	1437	207.40	41
243.007	16.9	757	1504	994.61	195
251.051	5.2	49	1312	311.77	73
256.034	6.2	74	1511	377.19	78
268.023	7.2	53	1410	448.63	105
270.023	12.3	1623	1455	768.81	176
272.022	11.5	617	1474	719.30	164
276.012	5.0	48	1559	320.88	70
285.993	16.8	651	1514	1128.50	266
299.996	9.1	100	1556	643.96	156
302.995	9.9	469	1559	700.60	171
310.978	6.4	40	1627	469.24	113
314.021	3.5	46	1332	259.99	78
315.992	3.8	57	1412	287.48	82

m/z	S/N	Quality Fac.	Res.	Intens.	Area
327.999	19.0	3652	1617	1519.11	393
330.971	4.5	57	1725	359.10	88
338.985	5.8	67	1463	485.48	144
341.016	6.2	95	1625	520.47	140
342.993	5.1	42	1641	439.11	118
355.964	7.6	101	1633	681.51	193
368.999	7.8	254	1636	739.76	218
370.994	6.6	122	1536	625.51	198
379.011	4.3	51	1438	422.99	146
382.004	8.2	393	1621	805.12	250
398.973	23.2	8300	1732	2413.01	738
449.969	3.6	51	1764	430.69	150
487.945	3.5	58	1737	474.68	186
489.912	3.1	67	1883	425.50	155
506.928	66.6	443857	2062	9417.07	3263
511.983	5.9	94	1872	836.98	323
515.957	4.1	70	2012	590.26	214
604.888	4.0	134	2084	638.77	278
621.861	3.8	84	2038	606.28	280
625.936	3.1	47	2003	508.79	241
661.869	4.0	107	2013	667.46	340
678.885	4.8	560	1995	798.68	425
791.929	3.5	234	1836	547.68	394
810.866	5.6	310	1802	877.76	666
920.934	10.9	681	1966	1539.28	1294
1017.033	3.4	157	1775	453.53	492
1218.384	15.8	5533	1976	2148.04	2832
1425.686	4.5	664	1894	591.76	1074
1475.626	15.7	97	2855	1721.10	2208
1482.816	3.3	55	1961	357.08	674
1486.532	4.0	47	1651	421.05	947
1491.087	6.6	130	1811	698.68	1441
1494.193	4.1	245	2299	434.63	708
1497.274	5.1	126	2415	525.35	818
1500.585	5.9	90	2417	611.75	954
1505.295	3.8	141	1671	376.75	857
1510.995	4.3	129	1680	426.87	973
1519.840	3.0	110	2067	297.46	557
1528.985	3.9	50	1809	363.71	787
1536.794	45.8	26574	2951	4215.63	5646
1539.087	12.9	454	3906	1179.65	1197

C:\Users\madhurekha\Desktop\MALDI_Data\16th batch data 070718\BHU\2\0_B2\1\1545.9178.LIFT\1SRef

m/z	S/N	Quality Fac.	Res.	Intens.	Area
1543.640	12.0	110	3186	1091.06	1365
1545.948	129.7	55975	3356	11671.00	13889

C:\Users\madhurekha\Desktop\MALDI_Data\16th batch data 070718\BHU\2\0_B2\1\1764.0908.LIFT\1SRef

Comment 1

Comment 2

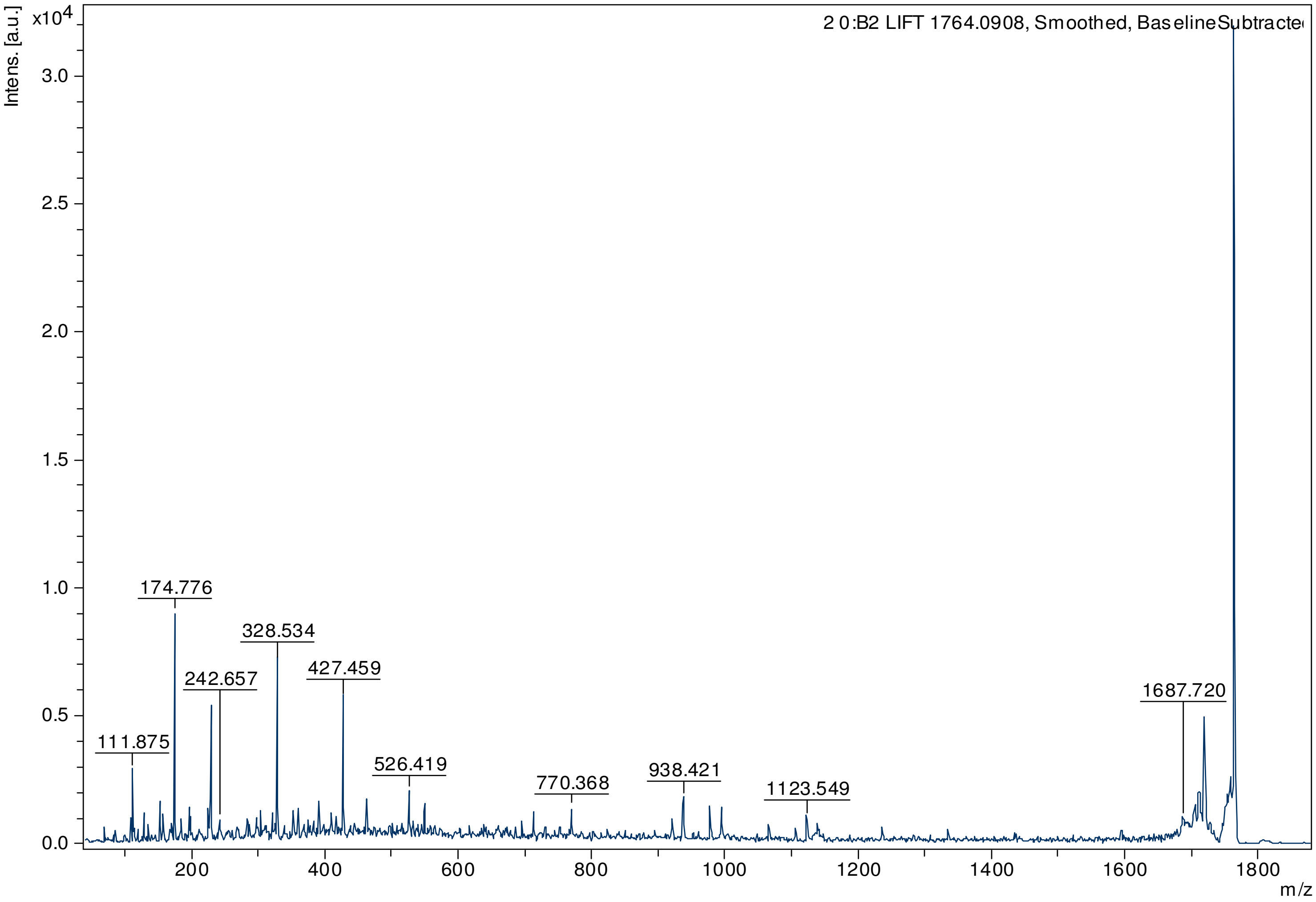

m/z	S/N	Quality Fac.	Res.	Intens.	Area
69.915	7.0	1092	413	683.16	127
83.901	3.6	1395	684	350.30	48
85.930	5.4	590	545	528.28	92
100.885	3.4	152	679	329.83	55
111.875	29.7	10731	1003	2919.78	365
114.865	6.3	1415	1099	622.00	73
119.860	6.0	1647	933	589.23	85
128.867	12.6	3926	1125	1246.13	161
132.849	3.1	628	1216	307.18	38
135.824	7.8	2696	1114	769.87	106
140.813	3.1	72	887	310.47	56
146.822	3.3	260	1162	330.77	48
153.801	16.5	3036	1230	1675.38	240
157.788	11.7	2830	1324	1200.51	164
168.780	5.5	841	1144	577.94	99
170.797	8.0	189	1250	839.72	134
174.776	85.6	23237	1241	9022.79	1486
180.758	3.1	44	1297	333.12	54
185.745	9.0	1749	1159	980.33	185
191.754	3.7	69	1120	405.09	82
196.762	13.4	2807	1195	1508.71	294
198.754	9.5	991	1237	1077.69	205
201.720	3.7	145	1181	427.47	87
211.710	5.5	59	1230	671.73	138
216.737	3.1	54	1251	384.25	80
225.679	11.3	521	1195	1478.78	336
229.653	40.7	14995	1305	5414.73	1150
242.657	6.8	118	1141	960.04	248
261.627	3.5	70	1169	555.96	153
271.651	3.3	38	1164	567.74	164
282.583	4.5	88	1163	823.62	249
284.592	5.5	132	1321	1029.73	276
287.644	4.2	117	1077	807.28	269
297.599	5.3	189	1348	1058.09	293
304.561	6.1	705	1281	1267.75	379
321.535	5.6	466	1455	1271.77	357
328.534	30.3	10341	1375	7100.37	2166
353.503	5.0	94	1170	1306.25	511

m/z	S/N	Quality Fac.	Res.	Intens.	Area
360.483	5.2	548	1401	1379.37	461
375.492	3.5	59	1377	991.13	354
392.466	5.7	481	1439	1706.70	616
410.458	4.3	37	1530	1346.58	483
417.438	3.4	61	1345	1074.88	448
427.459	17.5	11923	1500	5695.62	2190
463.428	5.2	470	1474	1818.29	787
526.419	5.7	612	1569	2099.23	1003
550.406	4.3	486	1632	1595.58	776
713.359	3.8	186	2042	1292.23	720
770.368	4.3	213	1977	1417.87	910
921.362	3.6	344	2078	1027.60	818
938.421	6.4	3509	2315	1812.04	1331
978.384	5.0	685	2056	1382.18	1219
995.451	5.4	2464	2092	1453.67	1294
1066.498	3.4	316	2048	853.84	866
1123.549	4.4	542	1685	1024.24	1382
1139.398	3.7	48	2780	855.65	716
1683.994	3.8	40	2218	684.99	1464
1687.720	5.8	215	2221	1032.58	2211
1690.911	3.7	120	1938	647.38	1595
1694.164	3.4	75	1880	592.83	1512
1698.333	4.4	86	2298	775.06	1624
1702.668	4.4	60	2300	761.09	1601
1704.734	6.6	372	1894	1127.98	2902
1707.440	5.4	120	1897	912.58	2345
1710.956	11.3	1881	1899	1899.44	4891
1713.458	4.4	88	1904	749.33	1935
1715.767	6.0	112	1906	1007.12	2602
1719.875	23.6	14604	1911	3915.19	10117
1726.462	3.7	195	1917	611.75	1590
1728.640	3.4	112	1921	551.73	1433
1746.077	3.3	80	1940	509.64	1339
1748.306	3.5	58	2769	555.87	1025
1750.529	8.6	245	3210	1351.89	2166
1753.901	11.7	1974	4162	1812.12	2238
1757.257	11.9	1510	4209	1835.70	2252
1759.503	9.2	74	3891	1408.83	1876
1761.873	13.3	46	3288	2034.04	3209
1764.091	163.1	28936	3073	24768.05	41892

C:\Users\madhurekha\Desktop\MALDI_Data\16th batch data 070718\BHU\2\0_B2\1\1SRef

Comment 1

Comment 2

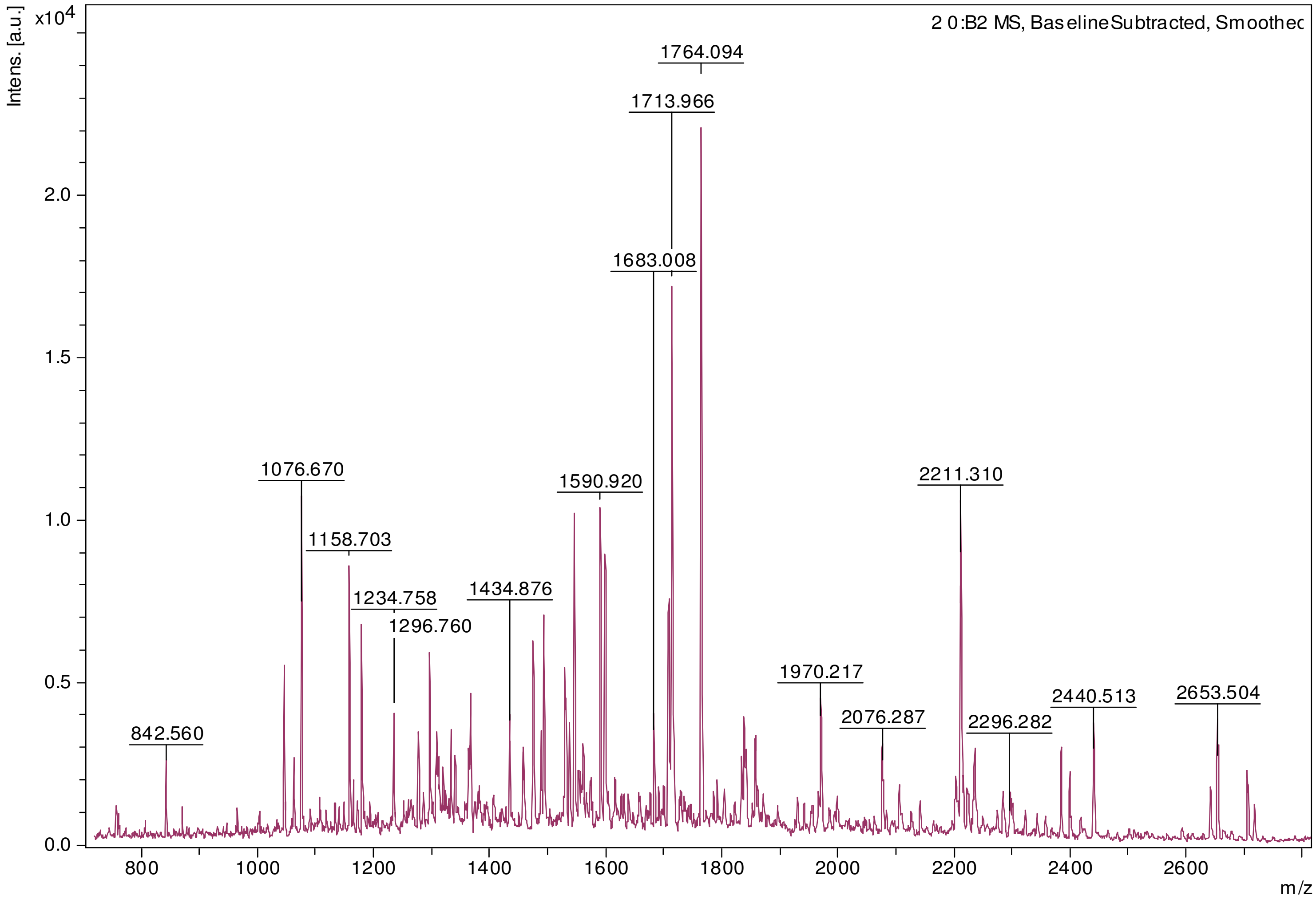

m/z	S/N	Quality Fac.	Res.	Intens.	Area
757.440	6.2	1281	4689	1071.90	289
842.560	13.3	5348	5800	2467.88	651
1046.610	26.2	22394	7181	5702.12	1708
1062.641	12.3	964	6979	2750.84	866
1075.652	28.8	2608	7268	6516.14	1958
1076.670	32.5	16406	7386	7376.42	2257
1158.703	35.0	30465	7510	8756.48	2974
1165.662	6.3	2394	6119	1589.24	642
1179.684	27.7	9489	8076	7108.40	2322
1234.758	15.5	1849	7418	4222.06	1567
1277.754	11.3	8645	7544	3164.86	1259
1296.760	22.4	3235	8322	6373.01	2377
1308.763	11.0	306	6027	3177.87	1597
1333.787	10.5	4325	7345	3059.88	1291
1340.788	8.0	1271	6751	2334.15	1090
1363.827	7.0	143	6318	2062.15	1044
1367.824	14.4	9842	8529	4253.29	1666
1434.876	12.2	9522	8209	3681.02	1599
1457.838	9.5	646	7039	2886.62	1502
1475.871	19.2	27590	8005	5846.14	2782
1488.886	10.0	2930	7452	3042.31	1536
1493.853	23.1	24804	8480	7033.30	3227
1530.804	15.3	4575	6300	4685.82	3001
1537.868	11.2	3498	7515	3419.09	1855
1545.921	34.1	61332	8692	10433.77	5017
1561.939	6.0	718	6277	1834.17	1211
1590.920	34.5	38807	9415	10486.76	5009
1598.956	31.5	29813	9310	9530.01	4628
1683.008	11.4	2049	7457	3392.36	2198
1707.930	22.2	3084	7667	6524.07	4277
1713.966	59.3	47902	9445	17390.34	9518
1764.094	81.6	42836	9967	23614.95	13159
1834.114	7.7	975	6792	2154.01	1819
1839.070	11.3	2043	7612	3164.21	2409
1858.146	8.8	851	6078	2413.12	2335
1970.217	16.0	1323	7218	3844.72	3373
2076.287	12.2	3183	8298	2486.28	2036
2203.333	8.4	470	6498	1397.54	1550

m/z	S/N	Quality Fac.	Res.	Intens.	Area
2211.310	54.0	30739	11032	8877.64	6117
2222.281	6.7	434	5200	1089.65	1533
2235.350	9.8	527	5350	1561.60	2136
2284.363	6.7	1047	6973	989.46	1075
2296.282	6.2	365	6296	904.80	1103
2384.175	15.9	2328	8113	2013.83	2011
2399.245	11.8	2928	8185	1453.17	1460
2440.513	24.6	19331	10473	2835.74	2307
2641.562	13.3	3177	8219	1100.17	1264
2653.504	32.5	35189	11398	2620.37	2224
2705.380	19.5	6338	9579	1424.56	1473
2717.322	9.7	3543	8203	700.19	854

MATRIX SCIENCE Mascot Search Results

User	: sanovar
Email	: sanovar@sandor.co.in
Search title	:
MS data file	: DATA.TXT
Database	: SwissProt 2018_06 (557713 sequences; 200130199 residues)
Taxonomy	: Bacteria (Eubacteria) (333552 sequences)
Timestamp	: 11 Jul 2018 at 11:37:18 GMT
Warning	: A Peptide summary report will usually give a much clearer picture of MS/MS search results.
Top Score	: 191 for DLDH_ECO57, Dihydrolipoyl dehydrogenase OS=Escherichia coli O157:H7 OX=83334 GN=lpdA PE=3 SV=2

Mascot Score Histogram

Protein score is -10*Log(P), where P is the probability that the observed match is a random event.
Protein scores greater than 68 are significant (p<0.05).
Protein scores are derived from ions scores as a non-probabilistic basis for ranking protein hits.

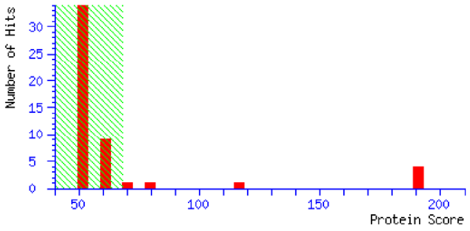

Protein Summary Report

Format As | Protein Summary (deprecated) | Help

Significance threshold p< 0.05 Max. number of hits 20

Preferred taxonomy All entries

Re-Search All | Search Unmatched

Index

	Accession	Mass	Score	Description
1.	DLDH_ECO57	50942	191	Dihydrolipoyl dehydrogenase OS=Escherichia coli O157:H7 OX=83334 GN=lpdA PE=3 SV=2
2.	DLDH_ECOL6	50942	191	Dihydrolipoyl dehydrogenase OS=Escherichia coli O6:H1 (strain CFT073 / ATCC 700928 / UPEC) OX=199310 GI
3.	DLDH_ECOLI	50942	191	Dihydrolipoyl dehydrogenase OS=Escherichia coli (strain K12) OX=83333 GN=lpdA PE=1 SV=2
4.	DLDH_SHIFL	50942	191	Dihydrolipoyl dehydrogenase OS=Shigella flexneri OX=623 GN=lpdA PE=3 SV=2
5.	DLDH_HAEIN	51521	119	Dihydrolipoyl dehydrogenase OS=Haemophilus influenzae (strain ATCC 51907 / DSM 11121 / KW20 / Rd) OX=7
6.	AROA_BACLD	45722	78	3-phosphoshikimate 1-carboxyvinyltransferase OS=Bacillus licheniformis (strain ATCC 14580 / DSM 13 / J
7.	GLMM_COXBN	48301	69	Phosphoglucosamine mutase OS=Coxiella burnetii (strain Dugway 5J108-111) OX=434922 GN=glmM PE=3 SV=1
8.	GLMM_COXB1	48347	63	Phosphoglucosamine mutase OS=Coxiella burnetii (strain CbuK_Q154) OX=434924 GN=glmM PE=3 SV=1
9.	GLMM_COXB2	48347	63	Phosphoglucosamine mutase OS=Coxiella burnetii (strain CbuG_Q212) OX=434923 GN=glmM PE=3 SV=1
10.	TRAA_RHIRD	123705	61	Conjugal transfer protein TraA OS=Rhizobium radiobacter OX=358 GN=traA PE=3 SV=1
11.	RPOC2_SYNSC	148515	61	DNA-directed RNA polymerase subunit beta' OS=Synechococcus sp. (strain CC9605) OX=110662 GN=rpoC2 PE=3
12.	PAT_SALAR	50192	61	Putrescine aminotransferase OS=Salmonella arizonae (strain ATCC BAA-731 / CDC346-86 / RSK2980) OX=4151
13.	PAT_SALPB	50078	61	Putrescine aminotransferase OS=Salmonella paratyphi B (strain ATCC BAA-1250 / SPB7) OX=1016998 GN=patA
14.	RPOC2_PROMS	150243	59	DNA-directed RNA polymerase subunit beta' OS=Prochlorococcus marinus (strain AS9601) OX=146891 GN=rpoC
15.	RPOC2_PROM0	150258	57	DNA-directed RNA polymerase subunit beta' OS=Prochlorococcus marinus (strain MIT 9301) OX=167546 GN=rp
16.	RL9_PARXL	16075	57	50S ribosomal protein L9 OS=Paraburkholderia xenovorans (strain LB400) OX=266265 GN=rplI PE=3 SV=1
17.	RL10_THIDA	18640	56	50S ribosomal protein L10 OS=Thiobacillus denitrificans (strain ATCC 25259) OX=292415 GN=rplJ PE=3 SV=
18.	EPMA_SALAR	37284	55	Elongation factor P--(R)-beta-lysine ligase OS=Salmonella arizonae (strain ATCC BAA-731 / CDC346-86 /
19.	IF2_BACFN	112675	55	Translation initiation factor IF-2 OS=Bacteroides fragilis (strain ATCC 25285 / DSM 2151 / JCM 11019 /
20.	IF2_BACFR	112675	55	Translation initiation factor IF-2 OS=Bacteroides fragilis (strain YCH46) OX=295405 GN=infB PE=3 SV=1

Results List

1. DLDH_ECO57 **Mass:** 50942 **Score:** 191 **Expect:** 2.6e-14 **Matches:** 8
Dihydrolipoyl dehydrogenase OS=Escherichia coli O157:H7 OX=83334 GN=lpdA PE=3 SV=2

Observed	Mr(expt)	Mr(calc)	ppm	Start	End	Miss	Ions	Peptide
758.4449	757.4377	757.4446	-9.19	297 -	302	1	---	R.VDKQLR.T
1076.6699	1075.6627	1075.6026	55.8	264 -	273	0	---	R.YDAVLVAIGR.V
1333.7866	1332.7793	1332.6786	75.5	285 -	296	1	---	K.AGVEVDDRGFIR.V
1367.8244	1366.8172	1366.7245	67.8	68 -	80	0	---	K.ALAEHGIVFGEPK.T
1713.9658	1712.9585	1712.8158	83.3	371 -	386	0	59	K.GISYETATFPWAASGR.A
1764.0939	1763.0866	1762.9366	85.1	7 -	24	0	103	K.TQVVVLGAGPAGYSAAFR.C
2274.3577	2273.3504	2273.1991	66.6	303 -	323	0	---	R.TNVPHIFAIGDIVGQPMLAHK.G + Oxidation (M)
2322.3983	2321.3910	2321.2379	66.0	2 -	24	1	---	M.STEIKTQVVVLGAGPAGYSAAFR.C

No match to: 761.4539, 773.4587, 807.4419, 842.5599, 870.5871, 931.5710, 965.5635, 1003.6035, 1046.6100, 1062.6412, 1064.6582, 1075.6517, 1118.6055, 1133.6818, 1140.6556, 1149.6929, 1158.7030, 1165.6616, 1172.7483, 1179.6837, 1234.7577, 1259.7439, 1277.7544, 1285.7582, 1296.7598, 1308.7634, 1320.6892, 1340.7881, 1373.7740, 1390.7958, 1434.8763, 1457.8382, 1475.8714, 1488.8863, 1493.8532, 1530.8044, 1537.8684, 1545.9206, 1553.9584, 1561.9388, 1590.9204, 1598.9555, 1615.9930, 1638.9781, 1657.9450, 1683.0081, 1690.9261, 1699.9587, 1707.9303, 1729.9719, 1786.0298, 1791.8986, 1804.0504, 1822.0336, 1834.1144, 1839.0705, 1842.1348, 1858.1460, 1862.0788, 1930.0964, 1941.1169, 1955.1238, 1970.2165, 1972.1537, 1985.1441, 1998.1688, 2076.2873, 2083.1868, 2105.2985, 2126.2672, 2141.2573, 2164.2353, 2203.3329, 2211.3104, 2215.3655, 2222.2808, 2235.3499, 2284.3628, 2296.2822, 2343.2148, 2357.2678, 2384.1747, 2399.2446, 2418.3632, 2440.5128, 2592.4853, 2641.5621, 2653.5041, 2705.3800, 2717.3219, 2888.5748, 3264.6235

2. DLDH_ECOL6 **Mass:** 50942 **Score:** 191 **Expect:** 2.6e-14 **Matches:** 8
Dihydrolipoyl dehydrogenase OS=Escherichia coli O6:H1 (strain CFT073 / ATCC 700928 / UPEC) OX=199310 GN=lpdA PE=3 SV=2

Observed	Mr(expt)	Mr(calc)	ppm	Start	End	Miss	Ions	Peptide
758.4449	757.4377	757.4446	-9.19	297 -	302	1	---	R.VDKQLR.T
1076.6699	1075.6627	1075.6026	55.8	264 -	273	0	---	R.YDAVLVAIGR.V

1333.7866	1332.7793	1332.6786	75.5	285	296	1	---	K.AGVEVDDRGFIR.V
1367.8244	1366.8172	1366.7245	67.8	68	80	0	---	K.ALAEHGIVFGEPK.T
1713.9658	1712.9585	1712.8158	83.3	371	386	0	59	K.GISYETATFPWAASGR.A
1764.0939	1763.0866	1762.9366	85.1	7	24	0	103	K.TQVVVLGAGPAGYSAAFR.C
2274.3577	2273.3504	2273.1991	66.6	303	323	0	---	R.TNVPHIFAIGDIVGQPMLAHK.G + Oxidation (M)
2322.3983	2321.3910	2321.2379	66.0	2	24	1	---	M.STEIKTQVVVLGAGPAGYSAAFR.C

No match to: 761.4539, 773.4587, 807.4419, 842.5599, 870.5871, 931.5710, 965.5635, 1003.6035, 1046.6100, 1062.6412, 1064.6582, 1075.6517, 1118.6055, 1133.6818, 1140.6556, 1149.6929, 1158.7030, 1165.6616, 1172.7483, 1179.6837, 1234.7577, 1259.7439, 1277.7544, 1285.7582, 1296.7598, 1308.7634, 1320.6892, 1340.7881, 1373.7740, 1390.7958, 1434.8763, 1457.8382, 1475.8714, 1488.8863, 1493.8532, 1530.8044, 1537.8684, 1545.9206, 1553.9584, 1561.9388, 1590.9204, 1598.9555, 1615.9930, 1638.9781, 1657.9450, 1683.0081, 1690.9261, 1699.9587, 1707.9303, 1729.9719, 1786.0298, 1791.8986, 1804.0504, 1822.0336, 1834.1144, 1839.0705, 1842.1348, 1858.1460, 1862.0788, 1930.0964, 1941.1169, 1955.1238, 1970.2165, 1972.1537, 1985.1441, 1998.1688, 2076.2873, 2083.1868, 2105.2985, 2126.2672, 2141.2573, 2164.2353, 2203.3329, 2211.3104, 2215.3655, 2222.2808, 2235.3499, 2284.3628, 2296.2822, 2343.2148, 2357.2678, 2384.1747, 2399.2446, 2418.3632, 2440.5128, 2592.4853, 2641.5621, 2653.5041, 2705.3800, 2717.3219, 2888.5748, 3264.6235

3. DLDH_ECOLI **Mass:** 50942 **Score:** 191 **Expect:** 2.6e-14 **Matches:** 8

Dihydrolipoyl dehydrogenase OS=Escherichia coli (strain K12) OX=83333 GN=lpdA PE=1 SV=2

Observed	Mr(expt)	Mr(calc)	ppm	Start	End	Miss	Ions	Peptide
758.4449	757.4377	757.4446	-9.19	297	302	1	---	R.VDKQLR.T
1076.6699	1075.6627	1075.6026	55.8	264	273	0	---	R.YDAVLVAIGR.V
1333.7866	1332.7793	1332.6786	75.5	285	296	1	---	K.AGVEVDDRGFIR.V
1367.8244	1366.8172	1366.7245	67.8	68	80	0	---	K.ALAEHGIVFGEPK.T
1713.9658	1712.9585	1712.8158	83.3	371	386	0	59	K.GISYETATFPWAASGR.A
1764.0939	1763.0866	1762.9366	85.1	7	24	0	103	K.TQVVVLGAGPAGYSAAFR.C
2274.3577	2273.3504	2273.1991	66.6	303	323	0	---	R.TNVPHIFAIGDIVGQPMLAHK.G + Oxidation (M)
2322.3983	2321.3910	2321.2379	66.0	2	24	1	---	M.STEIKTQVVVLGAGPAGYSAAFR.C

No match to: 761.4539, 773.4587, 807.4419, 842.5599, 870.5871, 931.5710, 965.5635, 1003.6035, 1046.6100, 1062.6412, 1064.6582, 1075.6517, 1118.6055, 1133.6818, 1140.6556, 1149.6929, 1158.7030, 1165.6616, 1172.7483, 1179.6837, 1234.7577, 1259.7439, 1277.7544, 1285.7582, 1296.7598, 1308.7634, 1320.6892, 1340.7881, 1373.7740, 1390.7958, 1434.8763, 1457.8382, 1475.8714, 1488.8863, 1493.8532, 1530.8044, 1537.8684, 1545.9206, 1553.9584, 1561.9388, 1590.9204, 1598.9555, 1615.9930, 1638.9781, 1657.9450, 1683.0081, 1690.9261, 1699.9587, 1707.9303, 1729.9719, 1786.0298, 1791.8986, 1804.0504, 1822.0336, 1834.1144, 1839.0705, 1842.1348, 1858.1460, 1862.0788, 1930.0964, 1941.1169, 1955.1238, 1970.2165, 1972.1537, 1985.1441, 1998.1688, 2076.2873, 2083.1868, 2105.2985, 2126.2672, 2141.2573, 2164.2353, 2203.3329, 2211.3104, 2215.3655, 2222.2808, 2235.3499, 2284.3628, 2296.2822, 2343.2148, 2357.2678, 2384.1747, 2399.2446, 2418.3632, 2440.5128, 2592.4853, 2641.5621, 2653.5041, 2705.3800, 2717.3219, 2888.5748, 3264.6235

4. DLDH_SHIFL **Mass:** 50942 **Score:** 191 **Expect:** 2.6e-14 **Matches:** 8

Dihydrolipoyl dehydrogenase OS=Shigella flexneri OX=623 GN=lpdA PE=3 SV=2

Observed	Mr(expt)	Mr(calc)	ppm	Start	End	Miss	Ions	Peptide
758.4449	757.4377	757.4446	-9.19	297	302	1	---	R.VDKQLR.T
1076.6699	1075.6627	1075.6026	55.8	264	273	0	---	R.YDAVLVAIGR.V
1333.7866	1332.7793	1332.6786	75.5	285	296	1	---	K.AGVEVDDRGFIR.V
1367.8244	1366.8172	1366.7245	67.8	68	80	0	---	K.ALAEHGIVFGEPK.T
1713.9658	1712.9585	1712.8158	83.3	371	386	0	59	K.GISYETATFPWAASGR.A
1764.0939	1763.0866	1762.9366	85.1	7	24	0	103	K.TQVVVLGAGPAGYSAAFR.C
2274.3577	2273.3504	2273.1991	66.6	303	323	0	---	R.TNVPHIFAIGDIVGQPMLAHK.G + Oxidation (M)
2322.3983	2321.3910	2321.2379	66.0	2	24	1	---	M.STEIKTQVVVLGAGPAGYSAAFR.C

No match to: 761.4539, 773.4587, 807.4419, 842.5599, 870.5871, 931.5710, 965.5635, 1003.6035, 1046.6100, 1062.6412, 1064.6582, 1075.6517, 1118.6055, 1133.6818, 1140.6556, 1149.6929, 1158.7030, 1165.6616, 1172.7483, 1179.6837, 1234.7577, 1259.7439, 1277.7544, 1285.7582, 1296.7598, 1308.7634, 1320.6892, 1340.7881, 1373.7740, 1390.7958, 1434.8763, 1457.8382, 1475.8714, 1488.8863, 1493.8532, 1530.8044, 1537.8684, 1545.9206, 1553.9584, 1561.9388, 1590.9204, 1598.9555, 1615.9930, 1638.9781, 1657.9450, 1683.0081, 1690.9261, 1699.9587, 1707.9303, 1729.9719, 1786.0298, 1791.8986, 1804.0504, 1822.0336, 1834.1144, 1839.0705, 1842.1348, 1858.1460, 1862.0788, 1930.0964, 1941.1169, 1955.1238, 1970.2165, 1972.1537, 1985.1441, 1998.1688, 2076.2873, 2083.1868, 2105.2985, 2126.2672, 2141.2573, 2164.2353, 2203.3329, 2211.3104, 2215.3655, 2222.2808, 2235.3499, 2284.3628, 2296.2822, 2343.2148, 2357.2678, 2384.1747, 2399.2446, 2418.3632, 2440.5128, 2592.4853, 2641.5621, 2653.5041, 2705.3800, 2717.3219, 2888.5748, 3264.6235

5. DLDH_HAEIN **Mass:** 51521 **Score:** 119 **Expect:** 4.2e-07 **Matches:** 5

Dihydrolipoyl dehydrogenase OS=Haemophilus influenzae (strain ATCC 51907 / DSM 11121 / KW20 / Rd) OX=71421 GN=lpdA PE=3 SV=2

Observed	Mr(expt)	Mr(calc)	ppm	Start	End	Miss	Ions	Peptide
1076.6699	1075.6627	1075.6026	55.8	264	273	0	---	R.YDAVLVAIGR.V
1457.8382	1456.8309	1456.7522	54.1	279	292	1	---	K.LIDAGKAGVEVDDR.G
1764.0939	1763.0866	1762.9366	85.1	7	24	0	103	K.TQVVVLGAGPAGYSAAFR.C
2274.3577	2273.3504	2273.1991	66.6	303	323	0	---	R.TNVPHIYAIGDIVGQPMLAHK.G
2705.3800	2704.3728	2704.3941	-7.90	300	323	1	---	K.QMRTNVPHIYAIGDIVGQPMLAHK.G + Oxidation (M)

No match to: 758.4449, 761.4539, 773.4587, 807.4419, 842.5599, 870.5871, 931.5710, 965.5635, 1003.6035, 1046.6100, 1062.6412, 1064.6582, 1075.6517, 1118.6055, 1133.6818, 1140.6556, 1149.6929, 1158.7030, 1165.6616, 1172.7483, 1179.6837, 1234.7577, 1259.7439, 1277.7544, 1285.7582, 1296.7598, 1308.7634, 1320.6892, 1333.7866, 1340.7881, 1367.8244, 1373.7740, 1390.7958, 1434.8763, 1475.8714, 1488.8863, 1493.8532, 1530.8044, 1537.8684, 1545.9206, 1553.9584, 1561.9388, 1590.9204, 1598.9555, 1615.9930, 1638.9781, 1657.9450, 1683.0081, 1690.9261, 1699.9587, 1707.9303, 1713.9658, 1729.9719, 1786.0298, 1791.8986, 1804.0504, 1822.0336, 1834.1144, 1839.0705, 1842.1348, 1858.1460, 1862.0788, 1930.0964, 1941.1169, 1955.1238, 1970.2165, 1972.1537, 1985.1441, 1998.1688, 2076.2873, 2083.1868, 2105.2985, 2126.2672, 2141.2573, 2164.2353, 2203.3329, 2211.3104, 2215.3655, 2222.2808, 2235.3499, 2284.3628, 2296.2822, 2322.3983, 2343.2148, 2357.2678, 2384.1747, 2399.2446, 2418.3632, 2440.5128, 2592.4853, 2641.5621, 2653.5041, 2717.3219, 2888.5748, 3264.6235

6. AROA_BACLD **Mass:** 45722 **Score:** 78 **Expect:** 0.0058 **Matches:** 14

3-phosphoshikimate 1-carboxyvinyltransferase OS=Bacillus licheniformis (strain ATCC 14580 / DSM 13 / JCM 2505 / NBRC 12200 / NCI

Observed	Mr(expt)	Mr(calc)	ppm	Start	End	Miss	Ions	Peptide
842.5599	841.5527	841.5021	60.0	124	130	1	---	R.VTEPLRK.M
870.5871	869.5798	869.5083	82.2	123	129	1	---	K.RVTEPLR.K
965.5635	964.5563	964.4978	60.6	11	19	0	---	K.GELHIPGDK.S
1140.6556	1139.6483	1139.5831	57.2	197	206	0	---	R.MLSMFGVSLR.E
1149.6929	1148.6856	1148.5826	89.7	139	149	0	---	R.AGGEYTPLSVR.G
1179.6837	1178.6765	1178.6772	-0.58	367	378	1	---	K.IHGKTPLTGGAK.V
1475.8714	1474.8641	1474.7627	68.8	340	352	1	---	K.ETNRIDTVASELK.K
1493.8532	1492.8459	1492.8249	14.1	6	19	1	---	K.ISSLKGELHIPGDK.S
1545.9206	1544.9134	1544.8059	69.5	11	24	1	7	K.GELHIPGDKSISHR.S
1590.9204	1589.9131	1589.8162	61.0	135	149	1	---	K.IDGRAGGEYTPLSVR.G
1657.9450	1656.9377	1656.9046	20.0	294	309	1	---	K.TSSLKAAEISGDLIPR.L
1707.9303	1706.9230	1706.8232	58.5	20	35	1	---	K.SISHRSVMFGAMAEGK.T
2322.3983	2321.3910	2321.3457	19.5	310	331	0	---	R.LIDEIPIIALLATQAEGTTVIK.D
2399.2446	2398.2373	2398.2679	-12.74	96	118	0	---	R.LMLGILAGRPFHSTVAGDESIAK.R + Oxidation (M)

No match to: 758.4449, 761.4539, 773.4587, 807.4419, 931.5710, 1003.6035, 1046.6100, 1062.6412, 1064.6582, 1075.6517, 1076.6699, 1118.6055, 1133.6818, 1158.7030, 1165.6616, 1172.7483, 1234.7577, 1259.7439, 1277.7544, 1285.7582, 1296.7598,

1308.7634, 1320.6892, 1333.7866, 1340.7881, 1367.8244, 1373.7740, 1390.7958, 1434.8763, 1457.8382, 1488.8863, 1530.8044, 1537.8684, 1553.9584, 1561.9388, 1598.9555, 1615.9930, 1638.9781, 1683.0081, 1690.9261, 1699.9587, 1713.9658, 1729.9719, 1764.0939, 1786.0298, 1791.8986, 1804.0504, 1822.0336, 1834.1144, 1839.0705, 1842.1348, 1858.1460, 1862.0788, 1930.0964, 1941.1169, 1955.1238, 1970.2165, 1972.1537, 1985.1441, 1998.1688, 2076.2873, 2083.1868, 2105.2985, 2126.2672, 2141.2573, 2164.2353, 2203.3329, 2211.3104, 2215.3655, 2222.2808, 2235.3499, 2274.3577, 2284.3628, 2296.2822, 2343.2148, 2357.2678, 2384.1747, 2418.3632, 2440.5128, 2592.4853, 2641.5621, 2653.5041, 2705.3800, 2717.3219, 2888.5748, 3264.6235

7. GLMM_COXBN **Mass:** 48301 **Score:** 69 **Expect:** 0.046 **Matches:** 9

Phosphoglucosamine mutase OS=Coxiella burnetii (strain Dugway 5J108-111) OX=434922 GN=glmM PE=3 SV=1

Observed	Mr(expt)	Mr(calc)	ppm	Start	End	Miss	Ions	Peptide
758.4449	757.4377	757.4235	18.7	28 -	34	0	---	K.LGWAVGR.V
761.4539	760.4466	760.3967	65.7	393 -	399	0	---	K.AITEAEK.Q
1234.7577	1233.7504	1233.7445	4.81	369 -	379	0	---	K.NPQVLINVPIK.G
1259.7439	1258.7367	1258.6656	56.5	120 -	130	0	---	K.LSDELELAIEK.Q
1457.8382	1456.8309	1456.7634	46.4	393 -	406	1	---	K.AITEAEKQLNGAGR.I
1561.9388	1560.9315	1560.7817	96.0	432 -	446	0	31	R.QTAEMLAAAVQQSTL.-
1707.9303	1706.9230	1707.0117	-51.96	365 -	379	1	---	K.VMVKNPQVLINVPIK.G + Oxidation (M)
2284.3628	2283.3555	2283.2144	61.8	274 -	295	0	---	K.ENAPLGVVGTIMSNLGLEQTLK.R
2440.5128	2439.5056	2439.3155	77.9	274 -	296	1	---	K.ENAPLGVVGTIMSNLGLEQTLKR.H

No match to: 773.4587, 807.4419, 842.5599, 870.5871, 931.5710, 965.5635, 1003.6035, 1046.6100, 1062.6412, 1064.6582, 1075.6517, 1076.6699, 1118.6055, 1133.6818, 1140.6556, 1149.6929, 1158.7030, 1165.6616, 1172.7483, 1179.6837, 1277.7544, 1285.7582, 1296.7598, 1308.7634, 1320.6892, 1333.7866, 1340.7881, 1367.8244, 1373.7740, 1390.7958, 1434.8763, 1475.8714, 1488.8863, 1493.8532, 1530.8044, 1537.8684, 1545.9206, 1553.9584, 1590.9204, 1598.9555, 1615.9930, 1638.9781, 1657.9450, 1683.0081, 1690.9261, 1699.9587, 1713.9658, 1729.9719, 1764.0939, 1786.0298, 1791.8986, 1804.0504, 1822.0336, 1834.1144, 1839.0705, 1842.1348, 1858.1460, 1862.0788, 1930.0964, 1941.1169, 1955.1238, 1970.2165, 1972.1537, 1985.1441, 1998.1688, 2076.2873, 2083.1868, 2105.2985, 2126.2672, 2141.2573, 2164.2353, 2203.3329, 2211.3104, 2215.3655, 2222.2808, 2235.3499, 2274.3577, 2296.2822, 2322.3983, 2343.2148, 2357.2678, 2384.1747, 2399.2446, 2418.3632, 2592.4853, 2641.5621, 2653.5041, 2705.3800, 2717.3219, 2888.5748, 3264.6235

8. GLMM_COXB1 **Mass:** 48347 **Score:** 63 **Expect:** 0.18 **Matches:** 8

Phosphoglucosamine mutase OS=Coxiella burnetii (strain CbuK_Q154) OX=434924 GN=glmM PE=3 SV=1

Observed	Mr(expt)	Mr(calc)	ppm	Start	End	Miss	Ions	Peptide
758.4449	757.4377	757.4235	18.7	28 -	34	0	---	K.LGWAVGR.V
761.4539	760.4466	760.3967	65.7	393 -	399	0	---	K.AITEAEK.Q
1259.7439	1258.7367	1258.6656	56.5	120 -	130	0	---	K.LSDELELAIEK.Q
1457.8382	1456.8309	1456.7634	46.4	393 -	406	1	---	K.AITEAEKQLNGAGR.I
1561.9388	1560.9315	1560.7817	96.0	432 -	446	0	31	R.QTAEMLAAAVQQSTL.-
1941.1169	1940.1096	1940.0190	46.7	74 -	91	0	---	K.LTGPMPTPAIAYLTHSVR.A + Oxidation (M)
2284.3628	2283.3555	2283.2144	61.8	274 -	295	0	---	K.ENAPLGVVGTIMSNLGLEQTLK.R
2440.5128	2439.5056	2439.3155	77.9	274 -	296	1	---	K.ENAPLGVVGTIMSNLGLEQTLKR.H

No match to: 773.4587, 807.4419, 842.5599, 870.5871, 931.5710, 965.5635, 1003.6035, 1046.6100, 1062.6412, 1064.6582, 1075.6517, 1076.6699, 1118.6055, 1133.6818, 1140.6556, 1149.6929, 1158.7030, 1165.6616, 1172.7483, 1179.6837, 1234.7577, 1277.7544, 1285.7582, 1296.7598, 1308.7634, 1320.6892, 1333.7866, 1340.7881, 1367.8244, 1373.7740, 1390.7958, 1434.8763, 1475.8714, 1488.8863, 1493.8532, 1530.8044, 1537.8684, 1545.9206, 1553.9584, 1590.9204, 1598.9555, 1615.9930, 1638.9781, 1657.9450, 1683.0081, 1690.9261, 1699.9587, 1707.9303, 1713.9658, 1729.9719, 1764.0939, 1786.0298, 1791.8986, 1804.0504, 1822.0336, 1834.1144, 1839.0705, 1842.1348, 1858.1460, 1862.0788, 1930.0964, 1955.1238, 1970.2165, 1972.1537, 1985.1441, 1998.1688, 2076.2873, 2083.1868, 2105.2985, 2126.2672, 2141.2573, 2164.2353, 2203.3329, 2211.3104, 2215.3655, 2222.2808, 2235.3499, 2274.3577, 2296.2822, 2322.3983, 2343.2148, 2357.2678, 2384.1747, 2399.2446, 2418.3632, 2592.4853, 2641.5621, 2653.5041, 2705.3800, 2717.3219, 2888.5748, 3264.6235

9. GLMM_COXB2 **Mass:** 48347 **Score:** 63 **Expect:** 0.18 **Matches:** 8

Phosphoglucosamine mutase OS=Coxiella burnetii (strain CbuG_Q212) OX=434923 GN=glmM PE=3 SV=1

Observed	Mr(expt)	Mr(calc)	ppm	Start	End	Miss	Ions	Peptide
758.4449	757.4377	757.4235	18.7	28 -	34	0	---	K.LGWAVGR.V
761.4539	760.4466	760.3967	65.7	393 -	399	0	---	K.AITEAEK.Q
1259.7439	1258.7367	1258.6656	56.5	120 -	130	0	---	K.LSDELELAIEK.Q
1457.8382	1456.8309	1456.7634	46.4	393 -	406	1	---	K.AITEAEKQLNGAGR.I
1561.9388	1560.9315	1560.7817	96.0	432 -	446	0	31	R.QTAEMLAAAVQQSTL.-
1941.1169	1940.1096	1940.0190	46.7	74 -	91	0	---	K.LTGPMPTPAIAYLTHSVR.A + Oxidation (M)
2284.3628	2283.3555	2283.2144	61.8	274 -	295	0	---	K.ENAPLGVVGTIMSNLGLEQTLK.R
2440.5128	2439.5056	2439.3155	77.9	274 -	296	1	---	K.ENAPLGVVGTIMSNLGLEQTLKR.H

No match to: 773.4587, 807.4419, 842.5599, 870.5871, 931.5710, 965.5635, 1003.6035, 1046.6100, 1062.6412, 1064.6582, 1075.6517, 1076.6699, 1118.6055, 1133.6818, 1140.6556, 1149.6929, 1158.7030, 1165.6616, 1172.7483, 1179.6837, 1234.7577, 1277.7544, 1285.7582, 1296.7598, 1308.7634, 1320.6892, 1333.7866, 1340.7881, 1367.8244, 1373.7740, 1390.7958, 1434.8763, 1475.8714, 1488.8863, 1493.8532, 1530.8044, 1537.8684, 1545.9206, 1553.9584, 1590.9204, 1598.9555, 1615.9930, 1638.9781, 1657.9450, 1683.0081, 1690.9261, 1699.9587, 1707.9303, 1713.9658, 1729.9719, 1764.0939, 1786.0298, 1791.8986, 1804.0504, 1822.0336, 1834.1144, 1839.0705, 1842.1348, 1858.1460, 1862.0788, 1930.0964, 1955.1238, 1970.2165, 1972.1537, 1985.1441, 1998.1688, 2076.2873, 2083.1868, 2105.2985, 2126.2672, 2141.2573, 2164.2353, 2203.3329, 2211.3104, 2215.3655, 2222.2808, 2235.3499, 2274.3577, 2296.2822, 2322.3983, 2343.2148, 2357.2678, 2384.1747, 2399.2446, 2418.3632, 2592.4853, 2641.5621, 2653.5041, 2705.3800, 2717.3219, 2888.5748, 3264.6235

10. TRAA_RHIRD **Mass:** 123705 **Score:** 61 **Expect:** 0.25 **Matches:** 20

Conjugal transfer protein TraA OS=Rhizobium radiobacter OX=358 GN=traA PE=3 SV=1

Observed	Mr(expt)	Mr(calc)	ppm	Start	End	Miss	Ions	Peptide
773.4587	772.4514	772.4443	9.23	611 -	616	1	---	R.EKLVER.G
807.4419	806.4346	806.3956	48.3	348 -	354	0	---	R.LEATMAR.Q + Oxidation (M)
931.5710	930.5638	930.4771	93.2	462 -	469	1	---	R.GRDLLDDK.T
965.5635	964.5563	964.5342	22.9	18 -	26	0	---	R.SVVLSAAYR.H
1118.6055	1117.5982	1117.5339	57.6	617 -	626	0	---	R.GMVGEGHLFR.T + Oxidation (M)
1165.6616	1164.6543	1164.5775	66.0	286 -	295	1	---	K.SVFDERDVAK.V
1234.7577	1233.7504	1233.6354	93.3	366 -	377	0	---	R.GVSPTALDATFR.R
1367.8244	1366.8172	1366.7569	44.1	161 -	173	1	---	K.KVAVIGEDGQPVR.T
1373.7740	1372.7668	1372.7059	44.4	960 -	970	1	---	R.LQTDEQSLRQR.V
1390.7958	1389.7885	1389.7365	37.5	366 -	378	1	---	R.GVSPTALDATFRR.H
1488.8863	1487.8790	1487.7732	71.1	996 -	1009	0	---	R.NDLPAAIAYALSNR.E
1537.8684	1536.8611	1536.7719	58.1	484 -	498	1	---	K.QMAGFVDTAVRAGAK.I + Oxidation (M)
1615.9930	1614.9858	1614.8590	78.5	2 -	17	1	---	M.AIAHFSASIVSRGSGR.S
1764.0939	1763.0866	1762.9254	91.5	46 -	61	0	14	K.QGLLHEEFVLPADAPK.W
1791.8986	1790.8913	1790.9275	-20.19	382 -	398	1	---	R.LSDEQKAAIEHVAGPAR.I
1804.0504	1803.0431	1802.9122	72.6	954 -	968	1	---	R.ETVTQRLQTDEQSLR.Q
1822.0336	1821.0263	1821.0723	-25.28	270 -	285	1	---	R.ILNNPAIVLDLITREK.S
1842.1348	1841.1275	1841.0271	54.5	202 -	218	1	---	R.LNHHLALGGIDLKIDGR.S
2126.2672	2125.2599	2125.1068	72.0	416 -	436	1	---	K.AAREAWELAGYHVVGGALAGK.A
2284.3628	2283.3555	2283.1535	88.5	176 -	195	1	---	K.SGKILYELWAGSTDDFNVLR.D

No match to: 758.4449, 761.4539, 842.5599, 870.5871, 1003.6035, 1046.6100, 1062.6412, 1064.6582, 1075.6517, 1076.6699, 1133.6818, 1140.6556, 1149.6929, 1158.7030, 1172.7483, 1179.6837, 1259.7439, 1277.7544, 1285.7582, 1296.7598, 1308.7634, 1320.6892, 1333.7866, 1340.7881, 1434.8763, 1457.8382, 1475.8714, 1493.8532, 1530.8044, 1545.9206, 1553.9584, 1561.9388, 1590.9204, 1598.9555, 1638.9781, 1657.9450, 1683.0081, 1690.9261, 1699.9587, 1707.9303, 1713.9658, 1729.9719, 1786.0298, 1834.1144, 1839.0705, 1858.1460, 1862.0788, 1930.0964, 1941.1169, 1955.1238, 1970.2165, 1972.1537, 1985.1441, 1998.1688, 2076.2873, 2083.1868, 2105.2985, 2141.2573, 2164.2353, 2203.3329, 2211.3104, 2215.3655, 2222.2808, 2235.3499, 2274.3577, 2296.2822, 2322.3983, 2343.2148, 2357.2678, 2384.1747, 2399.2446, 2418.3632, 2440.5128, 2592.4853, 2641.5621, 2653.5041, 2705.3800, 2717.3219, 2888.5748, 3264.6235

11. RPOC2_SYNSC **Mass:** 148515 **Score:** 61 **Expect:** 0.25 **Matches:** 18

DNA-directed RNA polymerase subunit beta' OS=Synechococcus sp. (strain CC9605) OX=110662 GN=rpoC2 PE=3 SV=1

Observed	Mr(expt)	Mr(calc)	ppm	Start	End	Miss	Ions	Peptide
773.4587	772.4514	772.4443	9.21	488	494	0	---	R.QGNITLK.A
807.4419	806.4346	806.3705	79.5	166	172	0	---	R.GNMSQVR.Q + Oxidation (M)
1165.6616	1164.6543	1164.5887	56.3	538	547	1	---	R.SEYGGEVRLR.D
1259.7439	1258.7367	1258.6816	43.8	1024	1033	1	---	R.RPRESTILCK.K
1320.6892	1319.6820	1319.7197	-28.63	298	310	1	---	K.SFEAAGVKAVSVR.S
1333.7866	1332.7793	1332.7323	35.3	555	566	0	---	R.EVQIVTTAMTLK.D
1373.7740	1372.7668	1372.7310	26.0	483	494	1	---	R.EVTDRQGNITLK.A
1475.8714	1474.8641	1474.7497	77.6	166	178	1	---	R.GNMSQVRQLVGMR.G
1545.9206	1544.9134	1544.7981	74.6	461	474	1	13	K.ATKDVICDLAGQVR.Y
1729.9719	1728.9646	1728.8431	70.3	125	138	1	---	R.HTKVIDTWTETNER.L
1834.1144	1833.1072	1832.9381	92.2	361	378	1	---	R.TFHTGGVSTAETGVVRSK.V
1842.1348	1841.1275	1840.9618	90.0	970	985	0	---	R.LGRPYMVSPDSVLHVR.D + Oxidation (M)
1941.1169	1940.1096	1940.0102	51.2	1157	1174	0	---	R.VEDAGDTTLLPGELIELR.Q
1972.1537	1971.1464	1971.0538	47.0	986	1003	1	---	R.DGNLVQRGDGLALLVFER.Q
2105.2985	2104.2913	2104.1052	88.4	77	96	1	---	K.YATQAAVSISVDDLKVPEAK.K
2141.2573	2140.2500	2140.1052	67.7	72	91	1	---	K.DLGFKYATQAAVSISVDDLK.V
2384.1747	2383.1674	2383.0685	41.5	145	165	1	---	K.KNFDENAPLNSVWMMANSGAR.G + 2 Oxidation (M)
2888.5748	2887.5675	2887.4637	35.9	1175	1202	0	---	R.QVEDTNQAMAITGGAPAEFTPVLLGITK.A + Oxidation (M)

No match to: 758.4449, 761.4539, 842.5599, 870.5871, 931.5710, 965.5635, 1003.6035, 1046.6100, 1062.6412, 1064.6582, 1075.6517, 1076.6699, 1118.6055, 1133.6818, 1140.6556, 1149.6929, 1158.7030, 1172.7483, 1179.6837, 1234.7577, 1277.7544, 1285.7582, 1296.7598, 1308.7634, 1340.7881, 1367.8244, 1390.7958, 1434.8763, 1457.8382, 1488.8863, 1493.8532, 1530.8044, 1537.8684, 1553.9584, 1561.9388, 1590.9204, 1598.9555, 1615.9930, 1638.9781, 1657.9450, 1683.0081, 1690.9261, 1699.9587, 1707.9303, 1713.9658, 1764.0939, 1786.0298, 1791.8986, 1804.0504, 1822.0336, 1839.0705, 1858.1460, 1862.0788, 1930.0964, 1955.1238, 1970.2165, 1985.1441, 1998.1688, 2076.2873, 2083.1868, 2126.2672, 2164.2353, 2203.3329, 2211.3104, 2215.3655, 2222.2808, 2235.3499, 2274.3577, 2284.3628, 2296.2822, 2322.3983, 2343.2148, 2357.2678, 2399.2446, 2418.3632, 2440.5128, 2592.4853, 2641.5621, 2653.5041, 2705.3800, 2717.3219, 3264.6235

12. PAT_SALAR **Mass:** 50192 **Score:** 61 **Expect:** 0.28 **Matches:** 9

Putrescine aminotransferase OS=Salmonella arizonae (strain ATCC BAA-731 / CDC346-86 / RSK2980) OX=41514 GN=patA PE=3 SV=2

Observed	Mr(expt)	Mr(calc)	ppm	Start	End	Miss	Ions	Peptide
1259.7439	1258.7367	1258.6241	89.5	209	219	0	---	R.HVTFGNINAMR.M
1367.8244	1366.8172	1366.8548	-27.51	127	140	1	---	R.AILAKTLAALTPGK.L
1488.8863	1487.8790	1487.7521	85.3	44	55	1	---	K.EHVNPGFLEYRK.S
1545.9206	1544.9134	1544.8311	53.3	114	126	0	23	K.QPLHSQELLDPLR.A
1683.0081	1682.0008	1681.8536	87.5	372	385	1	---	R.QLAREYPDLVHDAR.G
1690.9261	1689.9189	1689.8356	49.3	444	459	1	---	K.SARNALAAMQVSVEEV.- + Oxidation (M)
2126.2672	2125.2599	2125.0659	91.3	4	23	0	---	R.LPSSASALACCAHALNLIEK.R
2222.2808	2221.2736	2221.0725	90.5	143	162	1	---	K.YSFFCNSGTESVEAALKLAK.A
2399.2446	2398.2373	2398.1331	43.5	259	279	1	---	R.KLCDEFGALMILDEVQTGMGR.T + Oxidation (M)

No match to: 758.4449, 761.4539, 773.4587, 807.4419, 842.5599, 870.5871, 931.5710, 965.5635, 1003.6035, 1046.6100, 1062.6412, 1064.6582, 1075.6517, 1076.6699, 1118.6055, 1133.6818, 1140.6556, 1149.6929, 1158.7030, 1165.6616, 1172.7483, 1179.6837, 1234.7577, 1277.7544, 1285.7582, 1296.7598, 1308.7634, 1320.6892, 1333.7866, 1340.7881, 1373.7740, 1390.7958, 1434.8763, 1457.8382, 1475.8714, 1493.8532, 1530.8044, 1537.8684, 1553.9584, 1561.9388, 1590.9204, 1598.9555, 1615.9930, 1638.9781, 1657.9450, 1699.9587, 1707.9303, 1713.9658, 1729.9719, 1764.0939, 1786.0298, 1791.8986, 1804.0504, 1822.0336, 1834.1144, 1839.0705, 1842.1348, 1858.1460, 1862.0788, 1930.0964, 1941.1169, 1955.1238, 1970.2165, 1972.1537, 1985.1441, 1998.1688, 2076.2873, 2083.1868, 2105.2985, 2141.2573, 2164.2353, 2203.3329, 2211.3104, 2215.3655, 2235.3499, 2274.3577, 2284.3628, 2296.2822, 2322.3983, 2343.2148, 2357.2678, 2384.1747, 2418.3632, 2440.5128, 2592.4853, 2641.5621, 2653.5041, 2705.3800, 2717.3219, 2888.5748, 3264.6235

13. PAT_SALPB **Mass:** 50078 **Score:** 61 **Expect:** 0.29 **Matches:** 9

Putrescine aminotransferase OS=Salmonella paratyphi B (strain ATCC BAA-1250 / SPB7) OX=1016998 GN=patA PE=3 SV=1

Observed	Mr(expt)	Mr(calc)	ppm	Start	End	Miss	Ions	Peptide
1367.8244	1366.8172	1366.7245	67.8	33	43	1	---	K.ALNREVIDYFK.E
1457.8382	1456.8309	1456.8362	-3.61	413	426	1	---	R.VLVAGTLNNAKTTR.I
1488.8863	1487.8790	1487.7521	85.3	44	55	1	---	K.EHVNPGFLEYRK.S
1545.9206	1544.9134	1544.8311	53.3	114	126	0	23	K.QPLHSQELLDPLR.A
1690.9261	1689.9189	1689.8356	49.3	444	459	1	---	K.SARNALAAMQVSVEEV.- + Oxidation (M)
1822.0336	1821.0263	1820.9872	21.5	194	208	1	---	K.STFRRPFMPLLPGFR.H
2076.2873	2075.2800	2075.1197	77.3	114	131	1	---	K.QPLHSQELLDPLRAMLAK.T + Oxidation (M)
2222.2808	2221.2736	2221.0725	90.5	143	162	1	---	K.YSFFCNSGTESVEAALKLAK.A
2399.2446	2398.2373	2398.1331	43.5	259	279	1	---	R.KLCDEFGALMILDEVQTGMGR.T + Oxidation (M)

No match to: 758.4449, 761.4539, 773.4587, 807.4419, 842.5599, 870.5871, 931.5710, 965.5635, 1003.6035, 1046.6100, 1062.6412, 1064.6582, 1075.6517, 1076.6699, 1118.6055, 1133.6818, 1140.6556, 1149.6929, 1158.7030, 1165.6616, 1172.7483, 1179.6837, 1234.7577, 1259.7439, 1277.7544, 1285.7582, 1296.7598, 1308.7634, 1320.6892, 1333.7866, 1340.7881, 1373.7740, 1390.7958, 1434.8763, 1475.8714, 1493.8532, 1530.8044, 1537.8684, 1553.9584, 1561.9388, 1590.9204, 1598.9555, 1615.9930, 1638.9781, 1657.9450, 1683.0081, 1699.9587, 1707.9303, 1713.9658, 1729.9719, 1764.0939, 1786.0298, 1791.8986, 1804.0504, 1834.1144, 1839.0705, 1842.1348, 1858.1460, 1862.0788, 1930.0964, 1941.1169, 1955.1238, 1970.2165, 1972.1537, 1985.1441, 1998.1688, 2083.1868, 2105.2985, 2126.2672, 2141.2573, 2164.2353, 2203.3329, 2211.3104, 2215.3655, 2235.3499, 2274.3577, 2284.3628, 2296.2822, 2322.3983, 2343.2148, 2357.2678, 2384.1747, 2418.3632, 2440.5128, 2592.4853, 2641.5621, 2653.5041, 2705.3800, 2717.3219, 2888.5748, 3264.6235

14. RPOC2_PROMS **Mass:** 150243 **Score:** 59 **Expect:** 0.43 **Matches:** 18

DNA-directed RNA polymerase subunit beta' OS=Prochlorococcus marinus (strain AS9601) OX=146891 GN=rpoC2 PE=3 SV=1

Observed	Mr(expt)	Mr(calc)	ppm	Start	End	Miss	Ions	Peptide
773.4587	772.4514	772.4443	9.21	490	496	0	---	R.QGNITLK.A
807.4419	806.4346	806.3705	79.5	166	172	0	---	R.GNMSQVR.Q + Oxidation (M)
1046.6100	1045.6027	1045.5291	70.4	253	262	0	---	R.SIVVEAEDGK.F
1062.6412	1061.6339	1061.5757	54.8	404	412	0	---	K.QAEVDFILK.I
1179.6837	1178.6765	1178.6547	18.5	799	808	1	---	R.LTYKDGELIK.S
1340.7881	1339.7808	1339.7208	44.8	413	425	1	---	K.IVPQGNNSGKAQK.I
1373.7740	1372.7668	1372.7310	26.0	485	496	1	---	K.EVTDRQGNITLK.A
1475.8714	1474.8641	1474.7497	77.6	166	178	1	---	R.GNMSQVRQLVGMR.G
1530.8044	1529.7971	1529.7508	30.3	57	71	0	---	K.THGTAITAAMADNLK.D + Oxidation (M)

1537.8684	1536.8611	1536.9715	-71.83	849 -	861	1	---	R.LNLVILESILVRR.D
1545.9206	1544.9134	1544.7981	74.6	463 -	476	1	13	K.ATKDVICDLAGQVR.Y
1713.9658	1712.9585	1712.8655	54.3	557 -	571	0	---	R.EVQIVTTSMSLTNFK.L + Oxidation (M)
1729.9719	1728.9646	1728.8431	70.3	125 -	138	1	---	R.HTKVIDTWTETNER.L
1955.1238	1954.1166	1954.0259	46.4	1159 -	1176	0	---	R.IEDAGDTTLLPGELIELR.Q
2141.2573	2140.2500	2140.1052	67.7	72 -	91	1	---	K.DLGFKYATQAAVSISVDDLK.V
2235.3499	2234.3426	2234.1907	68.0	685 -	704	1	---	K.DIFSQTSGIVTVTQKNDILR.E
2343.2148	2342.2075	2342.1424	27.8	951 -	971	1	---	K.SIKSTFCGEIEEVSNSSVTLR.Y
2888.5748	2887.5675	2887.4637	35.9	1177 -	1204	0	---	R.QVEDTNQAMAITGGAPAEFTPVLLGITK.A + Oxidation (M)

No match to: 758.4449, 761.4539, 842.5599, 870.5871, 931.5710, 965.5635, 1003.6035, 1064.6582, 1075.6517, 1076.6699, 1118.6055, 1133.6818, 1140.6556, 1149.6929, 1158.7030, 1165.6616, 1172.7483, 1234.7577, 1259.7439, 1277.7544, 1285.7582, 1296.7598, 1308.7634, 1320.6892, 1333.7866, 1367.8244, 1390.7958, 1434.8763, 1457.8382, 1488.8863, 1493.8532, 1553.9584, 1561.9388, 1590.9204, 1598.9555, 1615.9930, 1638.9781, 1657.9450, 1683.0081, 1690.9261, 1699.9587, 1707.9303, 1764.0939, 1786.0298, 1791.8986, 1804.0504, 1822.0336, 1834.1144, 1839.0705, 1842.1348, 1858.1460, 1862.0788, 1930.0964, 1941.1169, 1970.2165, 1972.1537, 1985.1441, 1998.1688, 2076.2873, 2083.1868, 2105.2985, 2126.2672, 2164.2353, 2203.3329, 2211.3104, 2215.3655, 2222.2808, 2274.3577, 2284.3628, 2296.2822, 2322.3983, 2357.2678, 2384.1747, 2399.2446, 2418.3632, 2440.5128, 2592.4853, 2641.5621, 2653.5041, 2705.3800, 2717.3219, 3264.6235

15. RPOC2_PROMO **Mass:** 150258 **Score:** 57 **Expect:** 0.67 **Matches:** 18
DNA-directed RNA polymerase subunit beta' OS=Prochlorococcus marinus (strain MIT 9301) OX=167546 GN=rpoC2 PE=3 SV=1

Observed	Mr(expt)	Mr(calc)	ppm	Start	End	Miss	Ions	Peptide
761.4539	760.4466	760.3967	65.7	922 -	927	1	---	R.EEDKIK.I
773.4587	772.4514	772.4443	9.21	490 -	496	0	---	R.QGNITLK.A
807.4419	806.4346	806.3705	79.5	166 -	172	0	---	R.GNMSQVR.Q + Oxidation (M)
1046.6100	1045.6027	1045.5291	70.4	253 -	262	0	---	R.SIVVEAEDGK.F
1062.6412	1061.6339	1061.5757	54.8	404 -	412	0	---	K.QAEVDFILK.I
1140.6556	1139.6483	1139.6298	16.2	1107 -	1116	0	---	K.DQKPLIEAAR.E
1179.6837	1178.6765	1178.6547	18.5	799 -	808	1	---	R.LTYKDGELIK.S
1373.7740	1372.7668	1372.7310	26.0	485 -	496	1	---	K.EVTDRQGNITLK.A
1475.8714	1474.8641	1474.7497	77.6	166 -	178	1	---	R.GNMSQVRQLVGMR.G
1537.8684	1536.8611	1536.9715	-71.83	849 -	861	1	---	R.LNLVILESILVRR.D
1545.9206	1544.9134	1544.7981	74.6	463 -	476	1	13	K.ATKDVICDLAGQVR.Y
1713.9658	1712.9585	1712.8655	54.3	557 -	571	0	---	R.EVQIVTTSMSLTNFK.L + Oxidation (M)
1729.9719	1728.9646	1728.8431	70.3	125 -	138	1	---	R.HTKVIDTWTETNER.L
1955.1238	1954.1166	1954.0259	46.4	1159 -	1176	0	---	R.IEDAGDTTLLPGELIELR.Q
2105.2985	2104.2913	2104.1052	88.4	77 -	96	1	---	K.YATQAAVSISVDDLKVPEAK.Q
2141.2573	2140.2500	2140.1052	67.7	72 -	91	1	---	K.DLGFKYATQAAVSISVDDLK.V
2235.3499	2234.3426	2234.1907	68.0	685 -	704	1	---	K.DIFSQTSGIVTVTQKNDILR.E
2888.5748	2887.5675	2887.4637	35.9	1177 -	1204	0	---	R.QVEDTNQAMAITGGAPAEFTPVLLGITK.A + Oxidation (M)

No match to: 758.4449, 842.5599, 870.5871, 931.5710, 965.5635, 1003.6035, 1064.6582, 1075.6517, 1076.6699, 1118.6055, 1133.6818, 1149.6929, 1158.7030, 1165.6616, 1172.7483, 1234.7577, 1259.7439, 1277.7544, 1285.7582, 1296.7598, 1308.7634, 1320.6892, 1333.7866, 1340.7881, 1367.8244, 1390.7958, 1434.8763, 1457.8382, 1488.8863, 1493.8532, 1530.8044, 1553.9584, 1561.9388, 1590.9204, 1598.9555, 1615.9930, 1638.9781, 1657.9450, 1683.0081, 1690.9261, 1699.9587, 1707.9303, 1764.0939, 1786.0298, 1791.8986, 1804.0504, 1822.0336, 1834.1144, 1839.0705, 1842.1348, 1858.1460, 1862.0788, 1930.0964, 1941.1169, 1970.2165, 1972.1537, 1985.1441, 1998.1688, 2076.2873, 2083.1868, 2126.2672, 2164.2353, 2203.3329, 2211.3104, 2215.3655, 2222.2808, 2274.3577, 2284.3628, 2296.2822, 2322.3983, 2343.2148, 2357.2678, 2384.1747, 2399.2446, 2418.3632, 2440.5128, 2592.4853, 2641.5621, 2653.5041, 2705.3800, 2717.3219, 3264.6235

16. RL9_PARXL **Mass:** 16075 **Score:** 57 **Expect:** 0.68 **Matches:** 5
50S ribosomal protein L9 OS=Paraburkholderia xenovorans (strain LB400) OX=266265 GN=rplI PE=3 SV=1

Observed	Mr(expt)	Mr(calc)	ppm	Start	End	Miss	Ions	Peptide
1003.6035	1002.5962	1002.5783	17.9	1 -	8	0	---	-.MQIILLEK.V + Oxidation (M)
1158.7030	1157.6958	1157.6404	47.8	72 -	82	0	---	K.LAGSTVQINQK.A
1713.9658	1712.9585	1712.9169	24.3	72 -	88	1	24	K.LAGSTVQINQKAGVDGR.L
2126.2672	2125.2599	2125.1491	52.2	62 -	82	1	---	K.LAAAQAQGEKLAGSTVQINQK.A
2203.3329	2202.3256	2202.1644	73.2	83 -	104	1	---	K.AGVDGRLFGSVTNADIAEALVK.Q

No match to: 758.4449, 761.4539, 773.4587, 807.4419, 842.5599, 870.5871, 931.5710, 965.5635, 1046.6100, 1062.6412, 1064.6582, 1075.6517, 1076.6699, 1118.6055, 1133.6818, 1140.6556, 1149.6929, 1165.6616, 1172.7483, 1179.6837, 1234.7577, 1259.7439, 1277.7544, 1285.7582, 1296.7598, 1308.7634, 1320.6892, 1333.7866, 1340.7881, 1367.8244, 1373.7740, 1390.7958, 1434.8763, 1457.8382, 1475.8714, 1488.8863, 1493.8532, 1530.8044, 1537.8684, 1545.9206, 1553.9584, 1561.9388, 1590.9204, 1598.9555, 1615.9930, 1638.9781, 1657.9450, 1683.0081, 1690.9261, 1699.9587, 1707.9303, 1729.9719, 1764.0939, 1786.0298, 1791.8986, 1804.0504, 1822.0336, 1834.1144, 1839.0705, 1842.1348, 1858.1460, 1862.0788, 1930.0964, 1941.1169, 1955.1238, 1970.2165, 1972.1537, 1985.1441, 1998.1688, 2076.2873, 2083.1868, 2105.2985, 2141.2573, 2164.2353, 2211.3104, 2215.3655, 2222.2808, 2235.3499, 2274.3577, 2284.3628, 2296.2822, 2322.3983, 2343.2148, 2357.2678, 2384.1747, 2399.2446, 2418.3632, 2440.5128, 2592.4853, 2641.5621, 2653.5041, 2705.3800, 2717.3219, 2888.5748, 3264.6235

17. RL10_THIDA **Mass:** 18640 **Score:** 56 **Expect:** 0.92 **Matches:** 7
50S ribosomal protein L10 OS=Thiobacillus denitrificans (strain ATCC 25259) OX=292415 GN=rplJ PE=3 SV=1

Observed	Mr(expt)	Mr(calc)	ppm	Start	End	Miss	Ions	Peptide
758.4449	757.4377	757.4559	-24.01	57 -	62	1	---	K.NTLVRR.A
1076.6699	1075.6627	1075.6390	22.0	48 -	56	1	---	K.EGVYLRVLK.N
1475.8714	1474.8641	1474.7814	56.1	32 -	44	1	---	R.GIAVEDMTQLRVK.A + Oxidation (M)
1545.9206	1544.9134	1544.7657	95.6	87 -	100	1	14	K.DPVAAAKVMHEFSK.T + Oxidation (M)
1862.0788	1861.0715	1860.9251	78.7	121 -	137	1	---	K.DVGNLASMPSREELLSK.L + Oxidation (M)
2215.3655	2214.3582	2214.2008	71.1	10 -	31	0	---	K.AVVAEVAAQVAAAQTVVVAEYR.G
2343.2148	2342.2075	2342.2958	-37.68	9 -	31	1	---	K.KAVVAEVAAQVAAAQTVVVAEYR.G

No match to: 761.4539, 773.4587, 807.4419, 842.5599, 870.5871, 931.5710, 965.5635, 1003.6035, 1046.6100, 1062.6412, 1064.6582, 1075.6517, 1118.6055, 1133.6818, 1140.6556, 1149.6929, 1158.7030, 1165.6616, 1172.7483, 1179.6837, 1234.7577, 1259.7439, 1277.7544, 1285.7582, 1296.7598, 1308.7634, 1320.6892, 1333.7866, 1340.7881, 1367.8244, 1373.7740, 1390.7958, 1434.8763, 1457.8382, 1488.8863, 1493.8532, 1530.8044, 1537.8684, 1553.9584, 1561.9388, 1590.9204, 1598.9555, 1615.9930, 1638.9781, 1657.9450, 1683.0081, 1690.9261, 1699.9587, 1707.9303, 1713.9658, 1729.9719, 1764.0939, 1786.0298, 1791.8986, 1804.0504, 1822.0336, 1834.1144, 1839.0705, 1842.1348, 1858.1460, 1930.0964, 1941.1169, 1955.1238, 1970.2165, 1972.1537, 1985.1441, 1998.1688, 2076.2873, 2083.1868, 2105.2985, 2126.2672, 2141.2573, 2164.2353, 2203.3329, 2211.3104, 2222.2808, 2235.3499, 2274.3577, 2284.3628, 2296.2822, 2322.3983, 2357.2678, 2384.1747, 2399.2446, 2418.3632, 2440.5128, 2592.4853, 2641.5621, 2653.5041, 2705.3800, 2717.3219, 2888.5748, 3264.6235

18. EPMA_SALAR **Mass:** 37284 **Score:** 55 **Expect:** 0.96 **Matches:** 3
Elongation factor P--(R)-beta-lysine ligase OS=Salmonella arizonae (strain ATCC BAA-731 / CDC346-86 / RSK2980) OX=41514 GN=epmA

Observed	Mr(expt)	Mr(calc)	ppm	Start	End	Miss	Ions	Peptide
1076.6699	1075.6627	1075.5808	76.1	20 -	28	1	---	R.AAIMTEIRR.F + Oxidation (M)
1561.9388	1560.9315	1560.7905	90.4	84 -	97	0	47	R.LLAAGCGPVFQLCR.S
1985.1441	1984.1368	1984.0378	49.9	2 -	19	1	---	M.SETATWQPSASVPNLLKR.A

No match to: 758.4449, 761.4539, 773.4587, 807.4419, 842.5599, 870.5871, 931.5710, 965.5635, 1003.6035, 1046.6100, 1062.6412, 1064.6582, 1075.6517, 1118.6055, 1133.6818, 1140.6556, 1149.6929, 1158.7030, 1165.6616, 1172.7483, 1179.6837, 1234.7577,

1259.7439, 1277.7544, 1285.7582, 1296.7598, 1308.7634, 1320.6892, 1333.7866, 1340.7881, 1367.8244, 1373.7740, 1390.7958, 1434.8763, 1457.8382, 1475.8714, 1488.8863, 1493.8532, 1530.8044, 1537.8684, 1545.9206, 1553.9584, 1590.9204, 1598.9555, 1615.9930, 1638.9781, 1657.9450, 1683.0081, 1690.9261, 1699.9587, 1707.9303, 1713.9658, 1729.9719, 1764.0939, 1786.0298, 1791.8986, 1804.0504, 1822.0336, 1834.1144, 1839.0705, 1842.1348, 1858.1460, 1862.0788, 1930.0964, 1941.1169, 1955.1238, 1970.2165, 1972.1537, 1998.1688, 2076.2873, 2083.1868, 2105.2985, 2126.2672, 2141.2573, 2164.2353, 2203.3329, 2211.3104, 2215.3655, 2222.2808, 2235.3499, 2274.3577, 2284.3628, 2296.2822, 2322.3983, 2343.2148, 2357.2678, 2384.1747, 2399.2446, 2418.3632, 2440.5128, 2592.4853, 2641.5621, 2653.5041, 2705.3800, 2717.3219, 2888.5748, 3264.6235

19. IF2_BACFN **Mass:** 112675 **Score:** 55 **Expect:** 1.2 **Matches:** 13

Translation initiation factor IF-2 OS=Bacteroides fragilis (strain ATCC 25285 / DSM 2151 / JCM 11019 / NCTC 9343) OX=272559 GN=i

Observed	Mr(expt)	Mr(calc)	ppm	Start	End	Miss	Ions	Peptide
1062.6412	1061.6339	1061.5618	67.9	63	70	1	---	R.FIQERQNK.D
1076.6699	1075.6627	1075.6026	55.8	235	243	0	---	K.IRPTEFVSK.I
1165.6616	1164.6543	1164.5458	93.2	734	742	1	---	K.AMFNERNQR.V
1259.7439	1258.7367	1258.7285	6.47	108	118	1	---	K.FKPVGKIDLDK.L
1296.7598	1295.7525	1295.6357	90.1	73	84	1	---	R.NKASVSIDGYDK.K
1340.7881	1339.7808	1339.6693	83.2	38	48	0	---	K.ITEEQYAMLVK.E + Oxidation (M)
1488.8863	1487.8790	1487.7467	88.9	381	393	1	---	K.QEVSEEDVAKQVK.E
1561.9388	1560.9315	1560.8107	77.4	690	704	1	20	R.NATGSIIESTLDKGR.G
1713.9658	1712.9585	1712.9937	-20.56	806	820	1	8	R.RIALGNFQELNVIVK.G
1842.1348	1841.1275	1840.9716	84.7	674	689	1	---	K.VLLEAEMLDLKANPNR.N + Oxidation (M)
2343.2148	2342.2075	2342.2477	-17.14	664	684	1	---	K.GLGVPELMEKVLLEAEMLDLK.A + Oxidation (M)
2384.1747	2383.1674	2383.2669	-41.73	584	606	1	---	R.GAKVTDIAIIIVAADDDVMPQTK.E
2888.5748	2887.5675	2887.3698	68.5	638	662	1	---	K.ETLAQMNYLVEEWGGKYQSQDISAK.K

No match to: 758.4449, 761.4539, 773.4587, 807.4419, 842.5599, 870.5871, 931.5710, 965.5635, 1003.6035, 1046.6100, 1064.6582, 1075.6517, 1118.6055, 1133.6818, 1140.6556, 1149.6929, 1158.7030, 1172.7483, 1179.6837, 1234.7577, 1277.7544, 1285.7582, 1308.7634, 1320.6892, 1333.7866, 1367.8244, 1373.7740, 1390.7958, 1434.8763, 1457.8382, 1475.8714, 1493.8532, 1530.8044, 1537.8684, 1545.9206, 1553.9584, 1590.9204, 1598.9555, 1615.9930, 1638.9781, 1657.9450, 1683.0081, 1690.9261, 1699.9587, 1707.9303, 1729.9719, 1764.0939, 1786.0298, 1791.8986, 1804.0504, 1822.0336, 1834.1144, 1839.0705, 1858.1460, 1862.0788, 1930.0964, 1941.1169, 1955.1238, 1970.2165, 1972.1537, 1985.1441, 1998.1688, 2076.2873, 2083.1868, 2105.2985, 2126.2672, 2141.2573, 2164.2353, 2203.3329, 2211.3104, 2215.3655, 2222.2808, 2235.3499, 2274.3577, 2284.3628, 2296.2822, 2322.3983, 2357.2678, 2399.2446, 2418.3632, 2440.5128, 2592.4853, 2641.5621, 2653.5041, 2705.3800, 2717.3219, 3264.6235

20. IF2_BACFR **Mass:** 112675 **Score:** 55 **Expect:** 1.2 **Matches:** 13

Translation initiation factor IF-2 OS=Bacteroides fragilis (strain YCH46) OX=295405 GN=infB PE=3 SV=1

Observed	Mr(expt)	Mr(calc)	ppm	Start	End	Miss	Ions	Peptide
1062.6412	1061.6339	1061.5618	67.9	63	70	1	---	R.FIQERQNK.D
1076.6699	1075.6627	1075.6026	55.8	235	243	0	---	K.IRPTEFVSK.I
1165.6616	1164.6543	1164.5458	93.2	734	742	1	---	K.AMFNERNQR.V
1259.7439	1258.7367	1258.7285	6.47	108	118	1	---	K.FKPVGKIDLDK.L
1296.7598	1295.7525	1295.6357	90.1	73	84	1	---	R.NKASVSIDGYDK.K
1340.7881	1339.7808	1339.6693	83.2	38	48	0	---	K.ITEEQYAMLVK.E + Oxidation (M)
1488.8863	1487.8790	1487.7467	88.9	381	393	1	---	K.QEVSEEDVAKQVK.E
1561.9388	1560.9315	1560.8107	77.4	690	704	1	20	R.NATGSIIESTLDKGR.G
1713.9658	1712.9585	1712.9937	-20.56	806	820	1	8	R.RIALGNFQELNVIVK.G
1842.1348	1841.1275	1840.9716	84.7	674	689	1	---	K.VLLEAEMLDLKANPNR.N + Oxidation (M)
2343.2148	2342.2075	2342.2477	-17.14	664	684	1	---	K.GLGVPELMEKVLLEAEMLDLK.A + Oxidation (M)
2384.1747	2383.1674	2383.2669	-41.73	584	606	1	---	R.GAKVTDIAIIIVAADDDVMPQTK.E
2888.5748	2887.5675	2887.3698	68.5	638	662	1	---	K.ETLAQMNYLVEEWGGKYQSQDISAK.K

No match to: 758.4449, 761.4539, 773.4587, 807.4419, 842.5599, 870.5871, 931.5710, 965.5635, 1003.6035, 1046.6100, 1064.6582, 1075.6517, 1118.6055, 1133.6818, 1140.6556, 1149.6929, 1158.7030, 1172.7483, 1179.6837, 1234.7577, 1277.7544, 1285.7582, 1308.7634, 1320.6892, 1333.7866, 1367.8244, 1373.7740, 1390.7958, 1434.8763, 1457.8382, 1475.8714, 1493.8532, 1530.8044, 1537.8684, 1545.9206, 1553.9584, 1590.9204, 1598.9555, 1615.9930, 1638.9781, 1657.9450, 1683.0081, 1690.9261, 1699.9587, 1707.9303, 1729.9719, 1764.0939, 1786.0298, 1791.8986, 1804.0504, 1822.0336, 1834.1144, 1839.0705, 1858.1460, 1862.0788, 1930.0964, 1941.1169, 1955.1238, 1970.2165, 1972.1537, 1985.1441, 1998.1688, 2076.2873, 2083.1868, 2105.2985, 2126.2672, 2141.2573, 2164.2353, 2203.3329, 2211.3104, 2215.3655, 2222.2808, 2235.3499, 2274.3577, 2284.3628, 2296.2822, 2322.3983, 2357.2678, 2399.2446, 2418.3632, 2440.5128, 2592.4853, 2641.5621, 2653.5041, 2705.3800, 2717.3219, 3264.6235

Search Parameters

Type of search : MS/MS Ion Search
Enzyme : Trypsin
Fixed modifications : Carbamidomethyl (C)
Variable modifications : Oxidation (M)
Mass values : Monoisotopic
Protein Mass : Unrestricted
Peptide Mass Tolerance : ± 100 ppm
Fragment Mass Tolerance: ± 2 Da
Max Missed Cleavages : 1
Instrument type : MALDI-TOF-TOF
Query1 (758.4449,1+): <no title>
Query2 (761.4539,1+): <no title>
Query3 (773.4587,1+): <no title>
Query4 (807.4419,1+): <no title>
Query5 (842.5599,1+): <no title>
Query6 (870.5871,1+): <no title>
Query7 (931.5710,1+): <no title>
Query8 (965.5635,1+): <no title>
Query9 (1003.6035,1+): <no title>
Query10 (1046.6100,1+): <no title>
Query11 (1062.6412,1+): <no title>
Query12 (1064.6582,1+): <no title>
Query13 (1075.6517,1+): <no title>
Query14 (1076.6699,1+): <no title>
Query15 (1118.6055,1+): <no title>
Query16 (1133.6818,1+): <no title>
Query17 (1140.6556,1+): <no title>
Query18 (1149.6929,1+): <no title>
Query19 (1158.7030,1+): <no title>
Query20 (1165.6616,1+): <no title>
Query21 (1172.7483,1+): <no title>
Query22 (1179.6837,1+): <no title>
Query23 (1234.7577,1+): <no title>
Query24 (1259.7439,1+): <no title>
Query25 (1277.7544,1+): <no title>
Query26 (1285.7582,1+): <no title>
Query27 (1296.7598,1+): <no title>
Query28 (1308.7634,1+): <no title>

```
 Query29 (1320.6892,1+): <no title>
 Query30 (1333.7866,1+): <no title>
 Query31 (1340.7881,1+): <no title>
 Query32 (1367.8244,1+): <no title>
 Query33 (1373.7740,1+): <no title>
 Query34 (1390.7958,1+): <no title>
 Query35 (1434.8763,1+): <no title>
 Query36 (1457.8382,1+): <no title>
 Query37 (1475.8714,1+): <no title>
 Query38 (1488.8863,1+): <no title>
 Query39 (1493.8532,1+): <no title>
 Query40 (1530.8044,1+): <no title>
 Query41 (1537.8684,1+): <no title>
 Query42 (1545.9206,1+): <no title>
 Query43 (1553.9584,1+): <no title>
 Query44 (1561.9388,1+): <no title>
 Query45 (1590.9204,1+): <no title>
 Query46 (1598.9555,1+): <no title>
 Query47 (1615.9930,1+): <no title>
 Query48 (1638.9781,1+): <no title>
 Query49 (1657.9450,1+): <no title>
 Query50 (1683.0081,1+): <no title>
 Query51 (1690.9261,1+): <no title>
 Query52 (1699.9587,1+): <no title>
 Query53 (1707.9303,1+): <no title>
 Query54 (1713.9658,1+): <no title>
 Query55 (1729.9719,1+): <no title>
 Query56 (1764.0939,1+): <no title>
 Query57 (1786.0298,1+): <no title>
 Query58 (1791.8986,1+): <no title>
 Query59 (1804.0504,1+): <no title>
 Query60 (1822.0336,1+): <no title>
 Query61 (1834.1144,1+): <no title>
 Query62 (1839.0705,1+): <no title>
 Query63 (1842.1348,1+): <no title>
 Query64 (1858.1460,1+): <no title>
 Query65 (1862.0788,1+): <no title>
 Query66 (1930.0964,1+): <no title>
 Query67 (1941.1169,1+): <no title>
 Query68 (1955.1238,1+): <no title>
 Query69 (1970.2165,1+): <no title>
 Query70 (1972.1537,1+): <no title>
 Query71 (1985.1441,1+): <no title>
 Query72 (1998.1688,1+): <no title>
 Query73 (2076.2873,1+): <no title>
 Query74 (2083.1868,1+): <no title>
 Query75 (2105.2985,1+): <no title>
 Query76 (2126.2672,1+): <no title>
 Query77 (2141.2573,1+): <no title>
 Query78 (2164.2353,1+): <no title>
 Query79 (2203.3329,1+): <no title>
 Query80 (2211.3104,1+): <no title>
 Query81 (2215.3655,1+): <no title>
 Query82 (2222.2808,1+): <no title>
 Query83 (2235.3499,1+): <no title>
 Query84 (2274.3577,1+): <no title>
 Query85 (2284.3628,1+): <no title>
 Query86 (2296.2822,1+): <no title>
 Query87 (2322.3983,1+): <no title>
 Query88 (2343.2148,1+): <no title>
 Query89 (2357.2678,1+): <no title>
 Query90 (2384.1747,1+): <no title>
 Query91 (2399.2446,1+): <no title>
 Query92 (2418.3632,1+): <no title>
 Query93 (2440.5128,1+): <no title>
 Query94 (2592.4853,1+): <no title>
 Query95 (2641.5621,1+): <no title>
 Query96 (2653.5041,1+): <no title>
 Query97 (2705.3800,1+): <no title>
 Query98 (2717.3219,1+): <no title>
 Query99 (2888.5748,1+): <no title>
Query100 (3264.6235,1+): <no title>
```

Mascot: http://www.matrixscience.com/

MALDI MS MS Data Sheet of Pus Isolate

C:\Users\madhurekha\Desktop\MALDI_Data\16th batch data 070718\BHU\3\0_B3\1\1233.7044.LIFT\1SRef

Comment 1

Comment 2

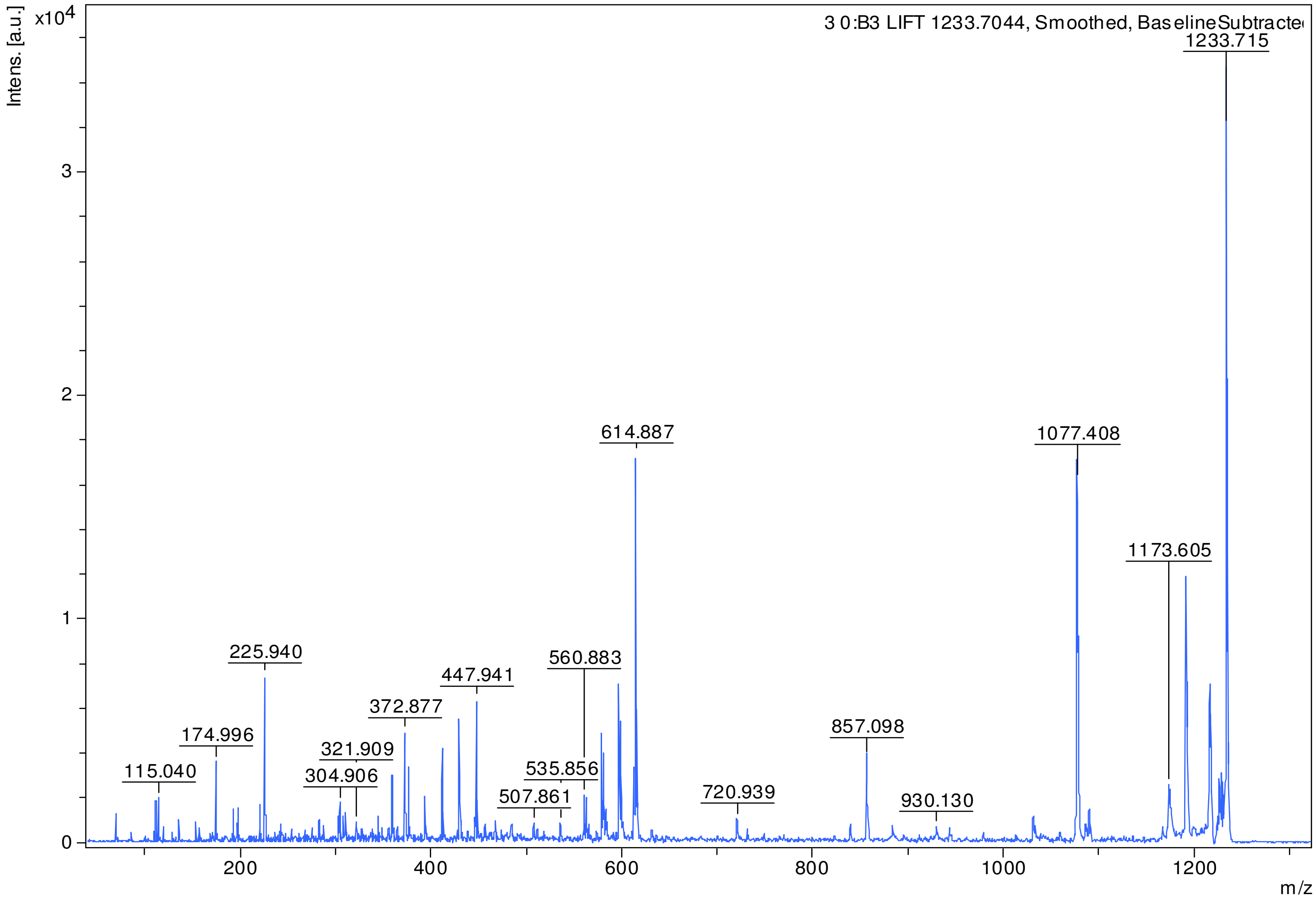

m/z	S/N	Quality Fac.	Res.	Intens.	Area
70.056	19.8	7453	449	1272.74	218
86.056	7.1	901	668	462.80	66
101.034	4.5	65	737	291.90	45
112.041	29.2	7109	874	1860.72	268
115.040	31.6	18831	946	2023.37	276
120.025	10.9	7789	957	688.41	97
129.030	6.7	381	892	421.87	69
133.024	3.8	180	939	240.42	39
136.010	15.9	11725	1013	1015.46	155
153.978	14.4	4200	1181	931.78	139
157.985	10.5	762	1158	679.85	106
168.994	6.0	539	1253	395.17	62
170.990	7.3	110	1193	480.45	80
174.996	52.3	17925	1202	3667.60	625
192.955	20.6	5685	1246	1519.87	278
197.968	21.5	3525	1337	1586.60	278
214.944	4.0	79	1237	313.07	65
216.960	3.6	44	1323	284.35	56
220.920	21.7	7880	1316	1726.37	348
225.940	92.2	28352	1432	7501.79	1425
229.922	4.8	74	1467	385.38	73
236.917	5.6	54	1209	471.35	112
242.939	10.0	137	1334	864.16	191
247.940	4.8	51	1552	420.63	82
253.970	6.7	109	1308	617.59	147
261.938	4.7	55	1219	445.65	118
267.929	5.7	212	1357	567.92	138
275.908	6.8	105	1349	719.93	182
282.919	9.7	557	1423	1057.84	262
287.931	6.7	632	1182	725.40	220
302.893	10.3	842	1597	1233.34	294
304.906	15.2	2442	1523	1837.05	463
308.026	9.6	792	1496	1189.94	309
310.883	11.2	1388	1435	1393.36	381
321.909	7.2	230	1397	965.82	283
336.906	3.2	34	1282	464.89	157
344.905	8.1	313	1588	1245.16	348
348.896	4.3	86	1256	669.86	240

m/z	S/N	Quality Fac.	Res.	Intens.	Area
359.861	18.9	9758	1560	3078.07	922
364.889	4.4	86	1376	730.03	252
372.877	28.9	21197	1578	4994.09	1545
376.878	19.4	12806	1517	3385.31	1103
393.963	12.0	1331	1769	2176.36	642
411.945	22.9	7575	1846	4351.83	1299
429.943	28.6	4831	1775	5669.09	1855
447.941	31.3	26338	1922	6483.52	2062
456.856	4.3	43	1574	901.11	359
467.843	4.5	116	1405	978.95	450
507.861	4.2	176	1296	935.40	517
535.856	3.9	274	1419	905.12	489
560.883	9.2	2664	1895	2159.73	928
563.196	8.1	609	1767	1875.93	869
565.578	3.1	57	1561	717.20	378
578.895	21.1	9556	1833	4883.03	2261
581.148	16.3	2571	1687	3754.75	1898
583.480	6.0	81	1348	1378.67	877
596.907	31.0	11345	1749	7044.03	3584
599.107	22.6	4224	1738	5131.18	2641
601.925	3.4	74	1306	771.74	532
614.887	77.9	89722	2004	17470.26	8075
720.939	5.6	490	1311	1093.32	963
731.945	3.3	328	1592	643.56	477
840.092	4.2	535	1634	779.30	687
857.098	20.7	16675	1573	3844.99	3624
884.075	4.0	131	1054	737.39	1087
930.130	3.9	221	1557	743.49	801
943.897	3.1	156	1370	600.96	753
1031.346	5.9	135	1528	1192.27	1537
1077.408	79.6	100182	1757	16226.36	19610
1086.595	4.1	195	1208	831.37	1482
1090.075	7.0	1485	2070	1450.67	1517
1167.312	4.0	159	1947	640.91	798
1173.605	16.9	1212	1363	2638.34	4734
1176.616	7.5	233	1307	1141.23	2145
1182.920	3.4	75	1312	510.06	968
1191.633	75.5	25712	1988	10926.44	13790
1198.910	5.9	58	1398	825.32	1509
1201.833	3.3	88	1335	458.06	879
1204.542	3.0	33	1769	417.86	604

C:\Users\madhurekha\Desktop\MALDI_Data\16th batch data 070718\BHU\3\0_B3\1\1233.7044.LIFT\1SRef

m/z	S/N	Quality Fac.	Res.	Intens.	Area
1206.553	3.7	42	1770	507.85	738
1208.612	4.6	37	1520	630.43	1066
1216.643	48.1	5324	1352	6397.60	12299
1224.541	8.7	227	1805	1138.60	1659
1226.837	19.6	1831	2467	2560.14	2736
1229.158	21.1	775	2791	2731.50	2588
1231.384	17.6	43	2885	2267.64	2087
1233.715	251.6	39482	2795	32094.42	30569

C:\Users\madhurekha\Desktop\MALDI_Data\16th batch data 070718\BHU\3\0_B3\1\1631.9377.LIFT\1SRef

Comment 1

Comment 2

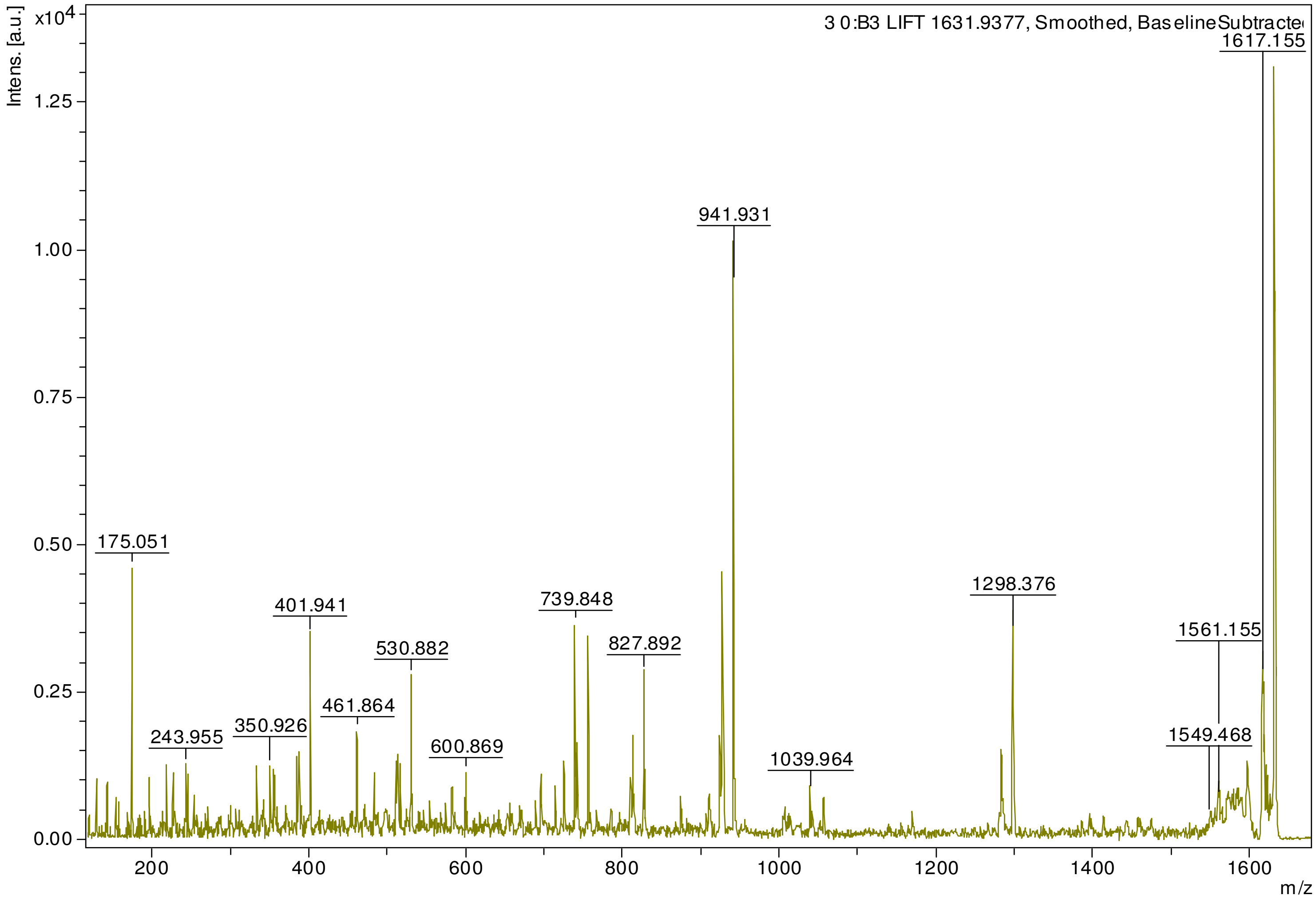

m/z	S/N	Quality Fac.	Res.	Intens.	Area
70.081	5.5	306	292	391.20	103
84.096	4.5	395	684	322.61	44
86.111	6.8	401	558	496.74	85
98.106	8.2	962	658	601.58	100
101.075	5.4	153	741	397.03	60
112.074	16.0	3352	1023	1146.22	141
120.075	5.8	1987	918	409.81	60
129.096	14.6	8466	1081	1035.14	140
136.038	3.4	110	1131	238.72	33
143.095	13.9	18129	1244	977.68	128
154.050	10.5	1035	1228	726.87	104
158.041	9.2	2380	1281	646.56	92
169.044	6.5	4186	1177	451.00	76
171.058	4.5	55	1203	312.25	52
175.051	66.9	26654	1286	4636.53	738
181.015	3.1	39	1361	216.42	34
183.039	5.1	93	1195	362.59	65
185.043	6.4	68	1215	453.24	81
191.030	6.9	152	1339	487.11	82
197.036	14.8	8338	1390	1050.60	176
201.024	3.3	34	1360	237.46	42
211.009	4.1	41	1188	299.77	64
214.044	6.3	149	1166	460.22	101
218.994	16.9	12832	1280	1275.68	262
226.983	15.4	1351	1283	1196.77	255
229.963	6.0	396	1200	473.76	110
232.030	3.7	39	1336	290.51	61
243.955	15.6	1768	1261	1281.53	302
246.009	13.4	3268	1194	1110.05	279
254.008	9.2	288	1290	792.01	191
257.993	4.8	92	1299	416.42	101
262.943	3.4	54	1331	305.85	74
271.988	6.2	169	1113	571.80	173
285.976	4.1	62	1254	394.64	112
288.017	4.3	50	1022	411.87	145
300.927	5.6	78	1310	576.95	167
308.040	4.7	86	1260	501.06	155
311.947	4.7	103	1395	504.63	143

m/z	S/N	Quality Fac.	Res.	Intens.	Area
333.897	10.7	2453	1354	1267.78	400
342.964	5.9	88	1354	732.17	239
350.926	9.8	1369	1400	1263.72	409
355.925	10.4	242	1253	1367.98	504
359.958	4.2	76	1266	562.06	208
370.983	4.3	87	1103	606.03	267
384.937	9.7	2415	1587	1423.28	455
388.048	10.2	1945	1265	1498.54	607
390.883	4.1	127	1268	609.85	249
401.941	22.7	133935	1667	3485.68	1118
461.864	10.5	4257	1499	1886.50	799
471.892	3.6	30	1348	653.72	316
483.910	6.2	221	1687	1150.25	459
511.898	7.2	593	1724	1400.40	588
513.896	7.2	843	1789	1409.68	573
516.998	6.6	844	1639	1300.45	581
530.882	14.0	71048	1945	2798.35	1090
554.882	3.3	64	1588	677.93	343
574.874	3.1	101	1564	647.74	348
582.854	4.7	145	1696	979.99	495
600.869	5.5	458	2103	1164.30	497
689.830	3.2	97	1916	687.82	389
696.865	5.4	357	1979	1162.53	646
714.862	4.5	300	2019	958.37	542
725.923	6.4	675	1780	1353.33	886
739.848	17.6	23048	2165	3690.41	2041
742.941	7.8	1610	2130	1635.81	925
756.858	16.2	16238	2176	3361.28	1911
810.848	5.8	240	1938	1168.31	824
813.883	8.8	2795	2244	1768.32	1083
827.892	14.6	28802	2205	2848.40	1820
874.808	4.2	228	1990	789.58	607
910.860	4.6	95	2060	855.68	675
924.891	10.3	862	2115	1890.84	1487
927.849	24.7	7827	1926	4556.99	3955
941.931	52.1	39009	2097	9462.53	7716
1007.816	3.0	43	2471	511.91	393
1039.964	5.0	727	2153	838.13	777
1057.026	4.1	1120	1669	687.27	843
1283.774	9.8	1104	1495	1547.23	2950
1298.376	21.9	37476	2020	3549.82	5111

C:\Users\madhurekha\Desktop\MALDI_Data\16th batch data 070718\BHU\3\0_B3\1\1631.9377.LIFT\1SRef

m/z	S/N	Quality Fac.	Res.	Intens.	Area
1549.468	3.6	49	1722	438.38	1023
1558.739	3.3	57	1733	393.17	921
1561.155	6.1	207	2025	725.33	1458
1564.765	4.4	190	1736	507.72	1192
1571.892	5.6	88	1744	637.08	1508
1574.110	5.8	255	1982	671.54	1403
1578.020	5.8	369	2052	656.94	1342
1580.233	4.0	184	1974	451.43	958
1582.181	4.8	126	2378	547.18	965
1584.441	6.6	350	1977	735.45	1565
1586.796	4.5	112	1761	505.56	1208
1589.317	5.9	206	1765	631.46	1513
1597.663	12.7	663	1774	1385.64	3336
1600.560	3.4	72	2065	374.23	775
1617.155	27.8	3399	1796	2815.07	6851
1620.658	6.9	220	3013	700.10	1018
1622.919	8.0	1073	3950	808.21	903
1631.919	110.6	23519	2965	10795.23	16204

C:\Users\madhurekha\Desktop\MALDI_Data\16th batch data 070718\BHU\3\0_B3\1\2229.3184.LIFT\1SRef

Comment 1

Comment 2

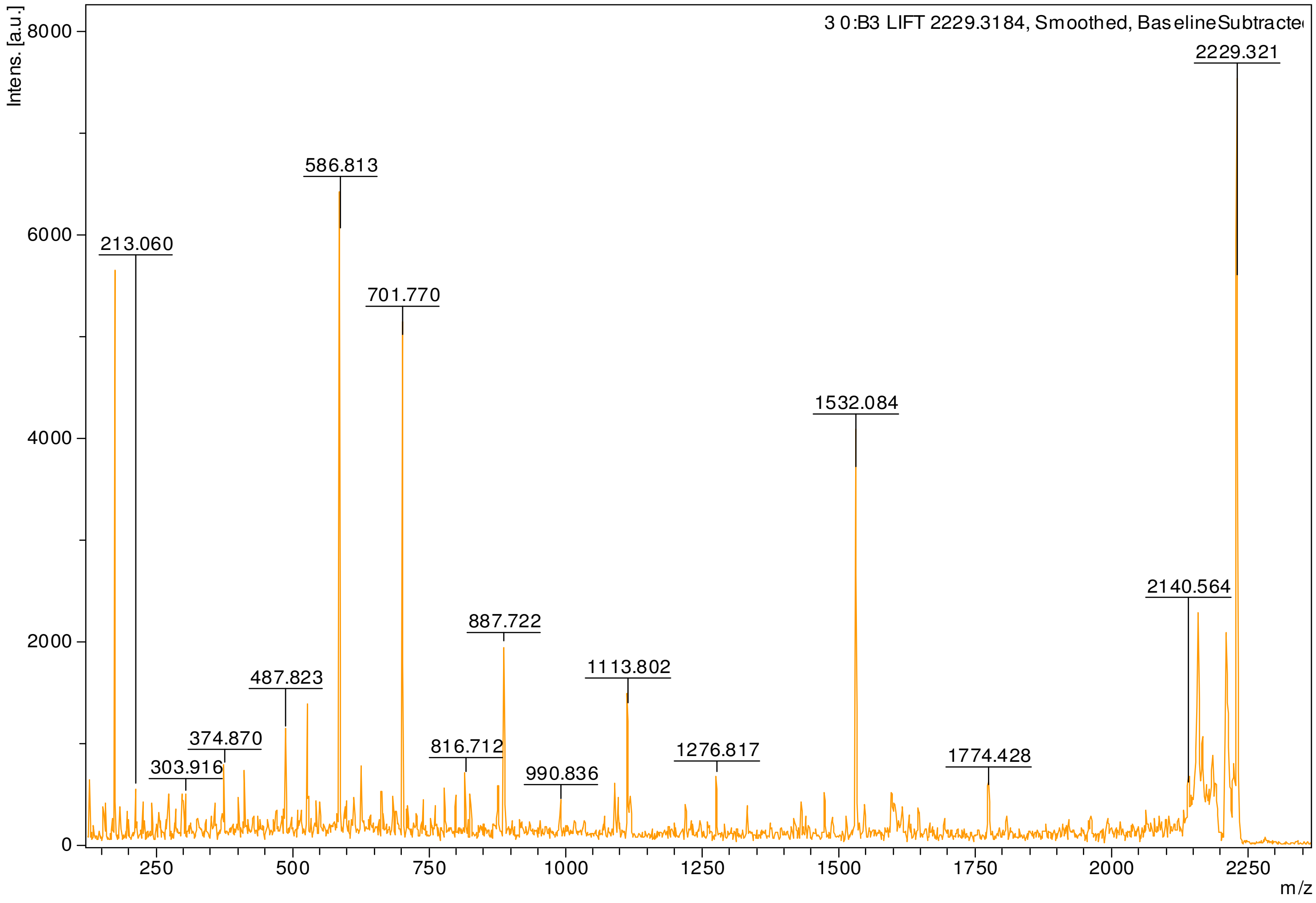

m/z	S/N	Quality Fac.	Res.	Intens.	Area
70.042	4.9	460	378	325.90	66
86.091	7.5	2602	724	493.45	65
102.071	3.6	520	1202	231.45	22
112.086	22.7	23550	1211	1500.50	156
115.092	3.5	33	1122	231.62	27
120.069	3.1	519	1351	206.46	21
129.103	9.6	12552	1234	631.99	75
154.065	5.7	220	1169	376.91	57
158.065	6.3	733	1282	415.02	59
171.064	4.2	86	1200	276.81	46
175.073	83.8	14652	1164	5622.16	989
185.084	5.6	408	1138	384.29	74
197.069	5.0	809	1059	349.62	77
199.071	3.8	155	930	262.82	67
201.018	3.4	214	867	242.80	67
213.060	7.7	1300	1078	563.13	133
227.004	5.1	367	1036	385.51	102
243.010	4.9	134	1181	394.79	99
255.971	3.9	254	1201	324.30	85
271.988	3.3	52	1035	281.95	92
274.048	6.2	1532	1042	544.22	177
286.969	4.0	71	945	367.00	139
297.922	5.0	241	1330	478.42	134
299.950	4.4	44	1282	419.88	123
303.916	5.0	732	1281	487.37	146
374.870	6.4	1250	1259	769.30	300
400.847	3.7	32	1221	468.29	204
412.832	5.3	386	1212	693.92	316
487.823	7.6	1367	1644	1118.86	463
527.729	8.9	1460	1522	1358.75	671
586.813	37.9	17113	1731	6017.36	3003
612.840	3.5	80	1360	548.39	372
626.721	4.7	368	1734	745.21	408
663.886	3.2	111	1361	512.00	387
684.763	3.3	37	2251	521.89	249
701.770	31.6	38146	1884	4976.98	2936
778.892	3.9	149	1739	586.07	434
799.739	3.2	243	1805	473.54	351

C:\Users\madhurekha\Desktop\MALDI_Data\16th batch data 070718\BHU\3\0_B3\1\2229.3184.LIFT\1SRef

m/z	S/N	Quality Fac.	Res.	Intens.	Area
816.712	4.5	454	1913	672.37	485
825.875	3.6	68	2084	541.57	365
877.840	4.1	244	1565	593.96	583
887.722	13.7	24233	2335	1969.24	1318
990.836	3.1	117	1971	416.33	391
1090.832	5.2	142	2467	693.49	609
1096.795	3.5	138	2435	474.00	426
1113.802	10.2	4859	2298	1357.47	1324
1219.743	3.1	113	2856	416.05	380
1276.817	5.1	1395	2289	674.79	832
1332.776	3.1	194	2497	409.74	500
1431.684	3.2	123	2909	444.57	529
1474.992	3.7	476	2224	517.13	851
1532.084	26.5	20840	2108	3673.87	6844
1546.821	3.2	38	2620	441.91	675
1597.726	3.8	80	2558	539.44	901
1774.428	4.1	478	1972	553.84	1478
2140.564	6.1	209	2379	568.07	1637
2145.668	3.6	198	2382	331.28	956
2148.640	3.4	72	2613	318.59	838
2151.806	4.9	99	2464	451.37	1263
2155.250	8.2	1082	3706	772.99	1439
2158.361	20.3	15675	2871	1889.69	4551
2165.553	6.7	216	2405	582.54	1682
2168.392	5.7	433	2688	508.40	1315
2170.956	3.1	92	2820	282.84	699
2174.102	4.6	181	2415	403.23	1165
2178.610	4.7	197	2421	411.88	1190
2182.088	3.9	105	2424	335.89	971
2185.266	8.8	364	2425	748.40	2167
2190.292	6.0	170	2432	520.19	1506
2207.882	6.1	130	2454	492.14	1432
2210.288	19.4	8137	2726	1619.31	4246
2213.753	10.2	1696	3812	860.39	1616
2218.143	4.4	157	6434	363.98	406
2222.442	6.4	644	4927	527.90	772
2224.719	5.4	35	4640	447.36	695
2229.321	68.0	50838	3615	5564.42	11129

C:\Users\madhurekha\Desktop\MALDI_Data\16th batch data 070718\BHU\3\0_B3\1\1SRef

Comment 1

Comment 2

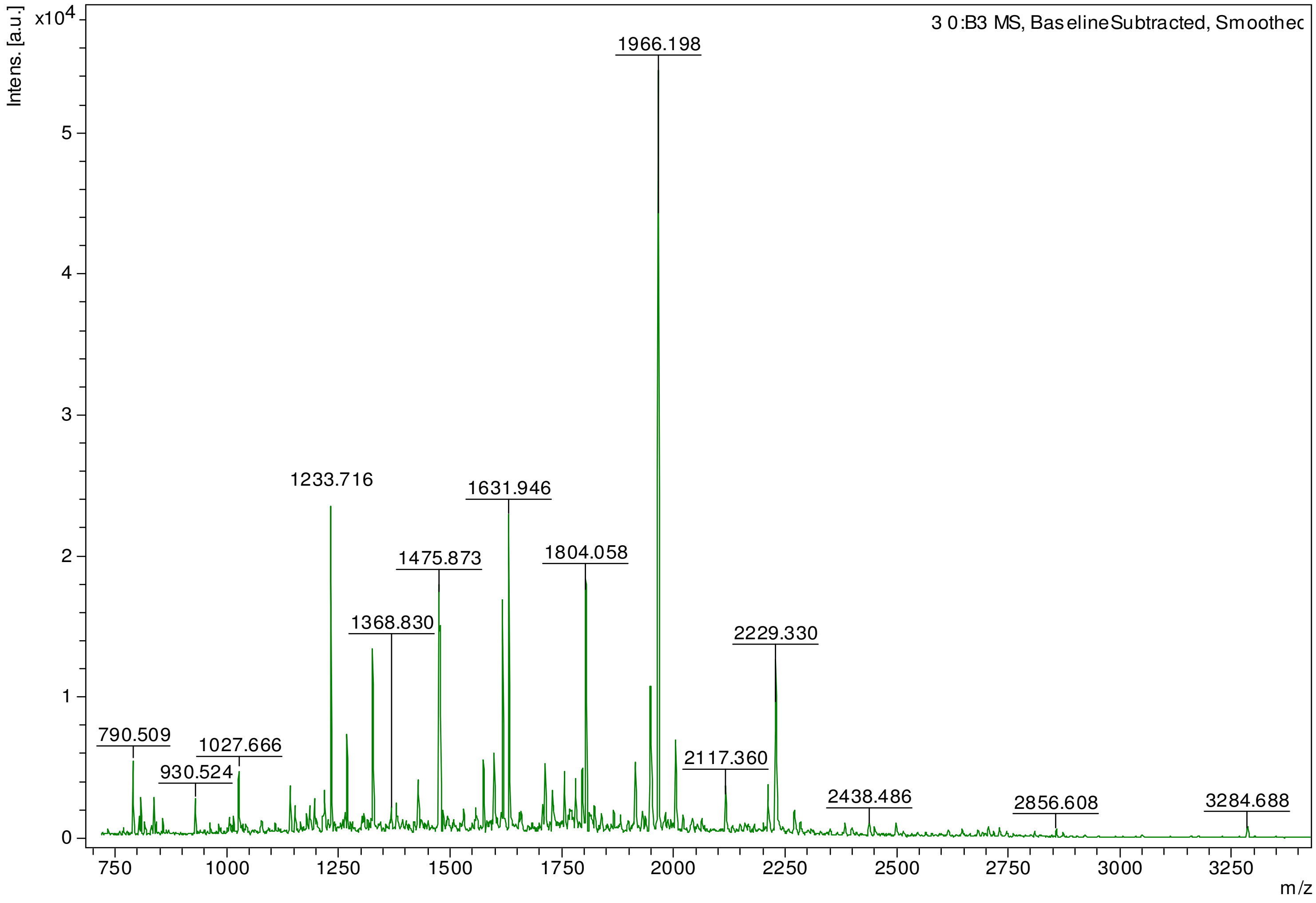

m/z	S/N	Quality Fac.	Res.	Intens.	Area
790.509	31.7	63576	5626	5388.92	1331
804.332	7.3	628	4936	1246.93	349
808.445	16.0	11428	5560	2753.22	699
837.559	15.6	14589	6098	2755.55	681
857.505	6.6	1667	5225	1179.25	345
930.524	13.7	6221	5839	2665.18	797
1006.645	6.1	488	4904	1261.25	502
1015.633	6.9	2270	7095	1451.76	418
1027.666	22.7	7210	7008	4778.47	1424
1142.674	14.3	6028	6169	3465.96	1354
1153.655	8.0	1498	5782	1960.22	830
1186.730	7.5	732	6058	1897.16	805
1197.658	8.0	365	4650	2052.49	1141
1219.644	10.2	1037	4037	2680.12	1782
1233.716	91.1	47471	6626	24270.98	10072
1269.724	25.1	15834	6787	6950.25	2966
1326.788	46.9	20428	6885	13464.13	6106
1368.830	6.5	1074	6642	1927.21	949
1380.867	6.6	544	5868	1965.26	1112
1428.921	11.7	3694	6251	3575.18	2029
1475.873	55.4	26434	7013	17148.72	9244
1477.849	24.6	10362	6550	7606.92	4367
1575.015	16.3	13125	7540	5133.41	2871
1598.984	19.1	10641	7281	5995.02	3642
1617.928	52.3	45964	7184	16354.19	10267
1631.946	72.9	62393	7498	22703.40	13937
1712.968	16.1	15133	7709	4863.80	3209
1729.003	10.2	5440	8430	3078.01	1867
1756.087	14.0	4531	7242	4146.67	3028
1781.094	9.6	810	5375	2838.58	2853
1797.115	11.7	7277	7642	3420.05	2497
1804.058	59.3	69867	7902	17300.11	12317
1915.149	16.0	7863	7463	4232.01	3479
1947.142	12.1	581	6974	3069.99	2721
1949.132	32.9	16438	7609	8314.25	6919
1952.152	8.2	901	4978	2071.61	2588
1966.198	177.6	30339	7372	44013.54	38141
2005.247	23.9	21912	7703	5639.58	4778

m/z	S/N	Quality Fac.	Res.	Intens.	Area
2117.360	14.3	4351	7444	2809.44	2628
2211.316	16.0	1493	7705	2721.93	2600
2229.330	56.5	7475	7739	9375.13	9076
2271.365	8.9	3328	7604	1386.29	1393
2438.486	6.1	925	6541	699.07	896
2856.608	6.5	4103	8169	361.51	476
3284.688	16.3	4338	8332	411.68	680

MATRIX SCIENCE Mascot Search Results

```
User          : Madhurarekha
Email         : sanovar@sandor.co.in
Search title  :
MS data file  : DATA.TXT
Database      : SwissProt 2018_06 (557713 sequences; 200130199 residues)
Taxonomy      : Bacteria (Eubacteria) (333552 sequences)
Timestamp     : 11 Jul 2018 at 11:45:45 GMT
Warning       : A Peptide summary report will usually give a much clearer picture of MS/MS search results.
Top Score     : 123 for EFTU_KLEP7, Elongation factor Tu OS=Klebsiella pneumoniae subsp. pneumoniae (strain ATCC 700721 / MGH 78578)
```

Mascot Score Histogram

Protein score is -10*Log(P), where P is the probability that the observed match is a random event.
Protein scores greater than 68 are significant (p<0.05).
Protein scores are derived from ions scores as a non-probabilistic basis for ranking protein hits.

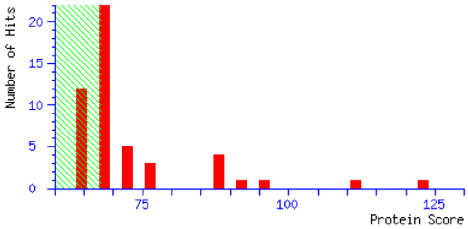

Protein Summary Report

Format As | Protein Summary (deprecated) | Help

Significance threshold p< 0.05 Max. number of hits 20

Preferred taxonomy All entries

Overview Table

Click on column header to jump to entry in results list.
Move mouse over any indicator to highlight identical peptides.
Click on an indicator to see details of individual match.
Use check boxes to select sub-set of queries for new search.

Mouse over: -Query-
-Accession- -Sequence-

Hit:	1	2	3	4	5	6	7	8	9	10	11	12	13	14	15	16	17	18	19	20
☑ 790.5086 (1+)																				
☑ 804.3324 (1+)																				
☑ 808.4445 (1+)																				
☑ 817.4935 (1+)																				
☑ 832.4057 (1+)																				
☑ 837.5595 (1+)																				
☑ 842.5625 (1+)																				
☑ 857.5055 (1+)																				
☑ 930.5245 (1+)																				
☑ 964.5936 (1+)																				
☑ 982.6070 (1+)																				
☑ 986.6133 (1+)																				
☑ 1006.6445 (1+)																				
☑ 1015.6326 (1+)																				
☑ 1027.6664 (1+)																				
☑ 1042.6300 (1+)																				
☑ 1078.6445 (1+)																				
☑ 1107.8344 (1+)																				
☑ 1142.6742 (1+)																				
☑ 1153.6546 (1+)																				
☑ 1165.6966 (1+)																				
☑ 1179.7073 (1+)																				
☑ 1186.7303 (1+)																				
☑ 1197.6582 (1+)																				
☑ 1214.7097 (1+)																				
☑ 1219.6443 (1+)																				
☑ 1233.7159 (1+)	●	●		●				●												
☑ 1240.6944 (1+)																				
☑ 1265.7392 (1+)																				
☑ 1269.7243 (1+)																				

☑ 1326.7883 (1+)			●																	
☑ 1368.8298 (1+)																				
☑ 1380.8667 (1+)																				
☑ 1428.9212 (1+)																				
☑ 1450.8531 (1+)																				
☑ 1475.8727 (1+)					●	●	●				●		●		●	●	●			
☑ 1477.8489 (1+)																				
☑ 1484.8980 (1+)																				
☑ 1530.8700 (1+)																				
☑ 1557.9283 (1+)																				
☑ 1575.0154 (1+)																				
☑ 1598.9839 (1+)																				
☑ 1617.9280 (1+)																				
☑ 1631.9464 (1+)					●	●	●	●		●	●			●	●	●		●	●	●
☑ 1654.9735 (1+)																				
☑ 1658.9825 (1+)																				
☑ 1708.0185 (1+)																				
☑ 1712.9684 (1+)																				
☑ 1724.0543 (1+)																				
☑ 1729.0035 (1+)																				
☑ 1733.0874 (1+)																				
☑ 1756.0868 (1+)																				
☑ 1781.0936 (1+)																				
☑ 1797.1145 (1+)																				
☑ 1804.0575 (1+)																				
☑ 1813.1035 (1+)																				
☑ 1824.0974 (1+)																				
☑ 1839.0932 (1+)																				
☑ 1850.0717 (1+)																				
☑ 1882.1314 (1+)																				
☑ 1900.1334 (1+)																				
☑ 1915.1486 (1+)																				
☑ 1931.1352 (1+)																				
☑ 1938.1476 (1+)																				
☑ 1947.1417 (1+)																				
☑ 1949.1320 (1+)																				
☑ 1952.1522 (1+)																				
☑ 1966.1980 (1+)	●	●		●					●				●	●			●			
☑ 1981.1803 (1+)																				
☑ 1994.1847 (1+)																				
☑ 2005.2465 (1+)																				
☑ 2021.1854 (1+)																				
☑ 2042.2539 (1+)																				
☑ 2044.2373 (1+)																				
☑ 2064.3065 (1+)																				
☑ 2117.3601 (1+)																				
☑ 2149.2508 (1+)																				
☑ 2181.2406 (1+)																				
☑ 2211.3162 (1+)																				
☑ 2215.2391 (1+)																				
☑ 2229.3298 (1+)									●			●								
☑ 2271.3649 (1+)																				
☑ 2284.3753 (1+)																				
☑ 2384.2549 (1+)																				
☑ 2399.3191 (1+)																				
☑ 2438.4857 (1+)																				
☑ 2450.5041 (1+)																				
☑ 2498.5023 (1+)																				
☑ 2615.5335 (1+)																				
☑ 2645.5873 (1+)																				
☑ 2681.5950 (1+)																				
☑ 2705.4362 (1+)																				
☑ 2717.3922 (1+)																				
☑ 2729.6061 (1+)																				
☑ 2745.6012 (1+)																				
☑ 2808.6023 (1+)																				
☑ 2856.6076 (1+)																				
☑ 2872.6380 (1+)																				
☑ 3048.6449 (1+)																				
☑ 3284.6878 (1+)																				

Select All | Select None | Search Selected

Index

	Accession	Mass	Score	Description
1.	EFTU_KLEP7	43390	123	Elongation factor Tu OS=Klebsiella pneumoniae subsp. pneumoniae (strain ATCC 700721 / MGH 78578) OX=27
2.	EFTU1_HAEI8	43384	112	Elongation factor Tu 1 OS=Haemophilus influenzae (strain 86-028NP) OX=281310 GN=tuf1 PE=3 SV=1
3.	CISY_SALTY	48474	96	Citrate synthase OS=Salmonella typhimurium (strain LT2 / SGSC1412 / ATCC 700720) OX=99287 GN=gltA PE=3
4.	EFTU_AERHH	43525	94	Elongation factor Tu OS=Aeromonas hydrophila subsp. hydrophila (strain ATCC 7966 / DSM 30187 / JCM 102
5.	EFTU_COXBN	43613	88	Elongation factor Tu OS=Coxiella burnetii (strain Dugway 5J108-111) OX=434922 GN=tuf1 PE=3 SV=1
6.	EFTU_COXBR	43613	88	Elongation factor Tu OS=Coxiella burnetii (strain RSA 331 / Henzerling II) OX=360115 GN=tuf1 PE=3 SV=1
7.	EFTU_COXBU	43613	88	Elongation factor Tu OS=Coxiella burnetii (strain RSA 493 / Nine Mile phase I) OX=227377 GN=tufA PE=1
8.	EFTU_MARHV	44034	87	Elongation factor Tu OS=Marinobacter hydrocarbonoclasticus (strain ATCC 700491 / DSM 11845 / VT8) OX=3
9.	DNAK_BRADU	68364	76	Chaperone protein DnaK OS=Bradyrhizobium diazoefficiens (strain JCM 10833 / IAM 13628 / NBRC 14792 / U
10.	ADH_THEBR	37851	75	NADP-dependent isopropanol dehydrogenase OS=Thermoanaerobacter brockii OX=29323 GN=adh PE=1 SV=1
11.	TOP1_STAS1	79764	75	DNA topoisomerase 1 OS=Staphylococcus saprophyticus subsp. saprophyticus (strain ATCC 15305 / DSM 2022
12.	HSCA_AROAE	66854	74	Chaperone protein HscA homolog OS=Aromatoleum aromaticum (strain EbN1) OX=76114 GN=hscA PE=3 SV=1
13.	LEU1_BUCUN	56434	72	2-isopropylmalate synthase (Fragment) OS=Buchnera aphidicola subsp. Uroleucon sonchi OX=118118 GN=leuA
14.	SECA_PROM4	107505	71	Protein translocase subunit SecA OS=Prochlorococcus marinus (strain MIT 9211) OX=93059 GN=secA PE=3 SV
15.	RPOA_PROM1	34244	71	DNA-directed RNA polymerase subunit alpha OS=Prochlorococcus marinus (strain NATL1A) OX=167555 GN=rpoA
16.	RPOA_PROMT	34244	71	DNA-directed RNA polymerase subunit alpha OS=Prochlorococcus marinus (strain NATL2A) OX=59920 GN=rpoA
17.	PROA_BRUSU	44300	69	Gamma-glutamyl phosphate reductase OS=Brucella suis biovar 1 (strain 1330) OX=204722 GN=proA PE=3 SV=1
18.	EFTU_HERAU	43768	69	Elongation factor Tu OS=Herpetosiphon aurantiacus OX=65 GN=tuf PE=3 SV=1
19.	EFTU1_HALHL	43283	69	Elongation factor Tu 1 OS=Halorhodospira halophila (strain DSM 244 / SL1) OX=349124 GN=tuf1 PE=3 SV=1
20.	EFTU2_HALHL	43269	69	Elongation factor Tu 2 OS=Halorhodospira halophila (strain DSM 244 / SL1) OX=349124 GN=tuf2 PE=3 SV=1

Results List

1. EFTU_KLEP7 **Mass:** 43390 **Score:** 123 **Expect:** 1.7e-07 **Matches:** 14

Elongation factor Tu OS=Klebsiella pneumoniae subsp. pneumoniae (strain ATCC 700721 / MGH 78578) OX=272620 GN=tufA PE=3 SV=1

Observed	Mr(expt)	Mr(calc)	ppm	Start - End	Miss	Ions	Peptide
837.5595	836.5522	836.4868	78.2	118 - 124	0	---	R.EHILLGR.Q
1027.6664	1026.6591	1026.5822	74.9	271 - 280	0	---	R.AGENVGVLLR.G
1214.7097	1213.7024	1213.6230	65.4	305 - 314	0	---	K.FESEVYILSK.D
1233.7159	1232.7086	1232.6091	80.8	326 - 334	0	10	K.GYRPQFYFR.T
1729.0035	1727.9962	1727.8366	92.4	305 - 319	1	---	K.FESEVYILSKDEGGR.H
1781.0936	1780.0863	1779.9375	83.6	359 - 374	0	---	K.MVVTLIHPIAMDDGLR.F
1797.1145	1796.1072	1795.9325	97.3	359 - 374	0	---	K.MVVTLIHPIAMDDGLR.F + Oxidation (M)
1804.0575	1803.0502	1802.8799	94.5	60 - 75	0	---	R.GITINTSHVEYDTPTR.H
1813.1035	1812.0963	1811.9274	93.2	359 - 374	0	---	K.MVVTLIHPIAMDDGLR.F + 2 Oxidation (M)
1966.1980	1965.1907	1965.0207	86.5	189 - 205	0	64	K.IIELAGHLDTYIPEPER.A
2117.3601	2116.3529	2116.1568	92.6	206 - 224	0	---	R.AIDKPFLLPIEDVFSISGR.G
2284.3753	2283.3680	2283.2232	63.4	359 - 378	1	---	K.MVVTLIHPIAMDDGLRFAIR.E + Oxidation (M)
2729.6061	2728.5988	2728.3346	96.8	91 - 117	0	---	K.NMITGAAQMDGAILVVAATDGPMPQTR.E
2745.6012	2744.5939	2744.3296	96.3	91 - 117	0	---	K.NMITGAAQMDGAILVVAATDGPMPQTR.E + Oxidation (M)

No match to: 790.5086, 804.3324, 808.4445, 817.4935, 832.4057, 842.5625, 857.5055, 930.5245, 964.5936, 982.6070, 986.6133, 1006.6445, 1015.6326, 1042.6300, 1078.6445, 1107.8344, 1142.6742, 1153.6546, 1165.6966, 1179.7073, 1186.7303, 1197.6582, 1219.6443, 1240.6944, 1265.7392, 1269.7243, 1326.7883, 1368.8298, 1380.8667, 1428.9212, 1450.8531, 1475.8727, 1477.8489, 1484.8980, 1530.8700, 1557.9283, 1575.0154, 1598.9839, 1617.9280, 1631.9464, 1654.9735, 1658.9825, 1708.0185, 1712.9684, 1724.0543, 1733.0874, 1756.0868, 1824.0974, 1839.0932, 1850.0717, 1882.1314, 1900.1334, 1915.1486, 1931.1352, 1938.1476, 1947.1417, 1949.1320, 1952.1522, 1981.1803, 1994.1847, 2005.2465, 2021.1854, 2042.2539, 2044.2373, 2064.3065, 2149.2508, 2181.2406, 2211.3162, 2215.2391, 2229.3298, 2271.3649, 2384.2549, 2399.3191, 2438.4857, 2450.5041, 2498.5023, 2615.5335, 2645.5873, 2681.5950, 2705.4362, 2717.3922, 2808.6023, 2856.6076, 2872.6380, 3048.6449, 3284.6878

2. EFTU1_HAEI8 **Mass:** 43384 **Score:** 112 **Expect:** 2.1e-06 **Matches:** 10

Elongation factor Tu 1 OS=Haemophilus influenzae (strain 86-028NP) OX=281310 GN=tuf1 PE=3 SV=1

Observed	Mr(expt)	Mr(calc)	ppm	Start - End	Miss	Ions	Peptide
837.5595	836.5522	836.4868	78.2	118 - 124	0	---	R.EHILLGR.Q
842.5625	841.5553	841.5134	49.8	232 - 238	1	---	R.VERGIIR.T
1233.7159	1232.7086	1232.6091	80.8	326 - 334	0	10	K.GYRPQFYFR.T
1575.0154	1574.0081	1573.8199	120	239 - 253	1	---	R.TGDEVEIVGIKDTAK.T
1598.9839	1597.9766	1597.9039	45.5	235 - 249	1	---	R.GIIRTGDEVEIVGIK.D
1804.0575	1803.0502	1802.8799	94.5	60 - 75	0	---	R.GITINTSHVEYDTPTR.H
1966.1980	1965.1907	1965.0207	86.5	189 - 205	0	64	K.ILELAGHLDTYIPEPER.A
2117.3601	2116.3529	2116.1205	110	206 - 224	0	---	R.AIDQPFLLPIEDVFSISGR.G
2729.6061	2728.5988	2728.3346	96.8	91 - 117	0	---	K.NMITGAAQMDGAILVVAATDGPMPQTR.E
2745.6012	2744.5939	2744.3296	96.3	91 - 117	0	---	K.NMITGAAQMDGAILVVAATDGPMPQTR.E + Oxidation (M)

No match to: 790.5086, 804.3324, 808.4445, 817.4935, 832.4057, 857.5055, 930.5245, 964.5936, 982.6070, 986.6133, 1006.6445, 1015.6326, 1027.6664, 1042.6300, 1078.6445, 1107.8344, 1142.6742, 1153.6546, 1165.6966, 1179.7073, 1186.7303, 1197.6582, 1214.7097, 1219.6443, 1240.6944, 1265.7392, 1269.7243, 1326.7883, 1368.8298, 1380.8667, 1428.9212, 1450.8531, 1475.8727, 1477.8489, 1484.8980, 1530.8700, 1557.9283, 1617.9280, 1631.9464, 1654.9735, 1658.9825, 1708.0185, 1712.9684, 1724.0543, 1729.0035, 1733.0874, 1756.0868, 1781.0936, 1797.1145, 1813.1035, 1824.0974, 1839.0932, 1850.0717, 1882.1314, 1900.1334, 1915.1486, 1931.1352, 1938.1476, 1947.1417, 1949.1320, 1952.1522, 1981.1803, 1994.1847, 2005.2465, 2021.1854, 2042.2539, 2044.2373, 2064.3065, 2149.2508, 2181.2406, 2211.3162, 2215.2391, 2229.3298, 2271.3649, 2284.3753, 2384.2549, 2399.3191, 2438.4857, 2450.5041, 2498.5023, 2615.5335, 2645.5873, 2681.5950, 2705.4362, 2717.3922, 2808.6023, 2856.6076, 2872.6380, 3048.6449, 3284.6878

3. CISY_SALTY **Mass:** 48474 **Score:** 96 **Expect:** 9.2e-05 **Matches:** 15

Citrate synthase OS=Salmonella typhimurium (strain LT2 / SGSC1412 / ATCC 700720) OX=99287 GN=gltA PE=3 SV=1

Observed	Mr(expt)	Mr(calc)	ppm	Start - End	Miss	Ions	Peptide
790.5086	789.5013	789.4286	92.1	121 - 126	0	---	R.LFHAFR.R
808.4445	807.4373	807.4490	-14.59	420 - 426	1	---	R.DFKSALK.R
817.4935	816.4862	816.4065	97.6	301 - 307	0	---	R.LMGFGHR.V
1015.6326	1014.6253	1014.4804	143	321 - 328	0	---	R.ETCHEVLK.E
1186.7303	1185.7230	1185.6354	73.9	23 - 33	0	---	K.GTLGQDVIDIR.S
1219.6443	1218.6371	1218.4905	120	169 - 178	0	---	K.MPTMAAMCYK.Y + Oxidation (M)
1265.7392	1264.7319	1264.6346	76.9	111 - 120	0	---	R.HTMIHEQITR.L
1326.7883	1325.7810	1325.6768	78.6	179 - 189	0	18	K.YSIGQPFVYPR.N
1598.9839	1597.9766	1597.8464	81.5	57 - 70	0	---	K.ITFIDGDEGILLHR.G
1631.9464	1630.9392	1630.9029	22.2	8 - 22	0	---	K.ITLTGDTTIELDVLK.G
1658.9825	1657.9752	1657.8999	45.4	23 - 38	1	---	K.GTLGQDVIDIRSLGSK.G
1824.0974	1823.0901	1822.9472	78.4	106 - 120	1	---	R.TTVTRHTMIHEQITR.L
1938.1476	1937.1403	1936.8513	149	39 - 56	0	---	K.GVFTFDPGFTSTASCESK.I
2005.2465	2004.2393	2004.0388	100	223 - 240	0	---	R.ILILHADHEQNASTSTVR.T

2717.3922	2716.3849	2716.3305	20.0	334 - 356	0	---	K.DDLLEVAMELEHIALNDPYFIEK.K

No match to: 804.3324, 832.4057, 837.5595, 842.5625, 857.5055, 930.5245, 964.5936, 982.6070, 986.6133, 1006.6445, 1027.6664, 1042.6300, 1078.6445, 1107.8344, 1142.6742, 1153.6546, 1165.6966, 1179.7073, 1197.6582, 1214.7097, 1233.7159, 1240.6944, 1269.7243, 1368.8298, 1380.8667, 1428.9212, 1450.8531, 1475.8727, 1477.8489, 1484.8980, 1530.8700, 1557.9283, 1575.0154, 1617.9280, 1654.9735, 1708.0185, 1712.9684, 1724.0543, 1729.0035, 1733.0874, 1756.0868, 1781.0936, 1797.1145, 1804.0575, 1813.1035, 1839.0932, 1850.0717, 1882.1314, 1900.1334, 1915.1486, 1931.1352, 1947.1417, 1949.1320, 1952.1522, 1966.1980, 1981.1803, 1994.1847, 2021.1854, 2042.2539, 2044.2373, 2064.3065, 2117.3601, 2149.2508, 2181.2406, 2211.3162, 2215.2391, 2229.3298, 2271.3649, 2284.3753, 2384.2549, 2399.3191, 2438.4857, 2450.5041, 2498.5023, 2615.5335, 2645.5873, 2681.5950, 2705.4362, 2729.6061, 2745.6012, 2808.6023, 2856.6076, 2872.6380, 3048.6449, 3284.6878

4. EFTU_AERHH **Mass:** 43525 **Score:** 94 **Expect:** 0.00015 **Matches:** 7

Elongation factor Tu OS=Aeromonas hydrophila subsp. hydrophila (strain ATCC 7966 / DSM 30187 / JCM 1027 / KCTC 2358 / NCIMB 9240

Observed	Mr(expt)	Mr(calc)	ppm	Start - End	Miss	Ions	Peptide
837.5595	836.5522	836.4868	78.2	118 - 124	0	---	R.EHILLGR.Q
1233.7159	1232.7086	1232.6091	80.8	326 - 334	0	10	K.GYRPQFYFR.T
1575.0154	1574.0081	1573.9304	49.4	290 - 304	0	---	R.GQVLAKPGTIKPHTK.F
1729.0035	1727.9962	1727.8366	92.4	305 - 319	1	---	K.FESEVYVLSKEEGGR.H
1966.1980	1965.1907	1965.0207	86.5	189 - 205	0	64	K.ILELAGHLDTYIPEPER.A
2729.6061	2728.5988	2728.3346	96.8	91 - 117	0	---	K.NMITGAAQMDGAILVVAATDGPMPQTR.E
2745.6012	2744.5939	2744.3296	96.3	91 - 117	0	---	K.NMITGAAQMDGAILVVAATDGPMPQTR.E + Oxidation (M)

No match to: 790.5086, 804.3324, 808.4445, 817.4935, 832.4057, 842.5625, 857.5055, 930.5245, 964.5936, 982.6070, 986.6133, 1006.6445, 1015.6326, 1027.6664, 1042.6300, 1078.6445, 1107.8344, 1142.6742, 1153.6546, 1165.6966, 1179.7073, 1186.7303, 1197.6582, 1214.7097, 1219.6443, 1240.6944, 1265.7392, 1269.7243, 1326.7883, 1368.8298, 1380.8667, 1428.9212, 1450.8531, 1475.8727, 1477.8489, 1484.8980, 1530.8700, 1557.9283, 1598.9839, 1617.9280, 1631.9464, 1654.9735, 1658.9825, 1708.0185, 1712.9684, 1724.0543, 1733.0874, 1756.0868, 1781.0936, 1797.1145, 1804.0575, 1813.1035, 1824.0974, 1839.0932, 1850.0717, 1882.1314, 1900.1334, 1915.1486, 1931.1352, 1938.1476, 1947.1417, 1949.1320, 1952.1522, 1981.1803, 1994.1847, 2005.2465, 2021.1854, 2042.2539, 2044.2373, 2064.3065, 2117.3601, 2149.2508, 2181.2406, 2211.3162, 2215.2391, 2229.3298, 2271.3649, 2284.3753, 2384.2549, 2399.3191, 2438.4857, 2450.5041, 2498.5023, 2615.5335, 2645.5873, 2681.5950, 2705.4362, 2717.3922, 2808.6023, 2856.6076, 2872.6380, 3048.6449, 3284.6878

5. EFTU_COXBN **Mass:** 43613 **Score:** 88 **Expect:** 0.00058 **Matches:** 8

Elongation factor Tu OS=Coxiella burnetii (strain Dugway 5J108-111) OX=434922 GN=tuf1 PE=3 SV=1

Observed	Mr(expt)	Mr(calc)	ppm	Start - End	Miss	Ions	Peptide
817.4935	816.4862	816.4090	94.6	286 - 291	1	---	K.REEVER.G
1186.7303	1185.7230	1185.6969	22.0	386 - 397	1	---	R.TVGAGVVTKIIE.-
1326.7883	1325.7810	1325.7231	43.7	306 - 316	1	---	K.KFEAEIYVLSK.E
1475.8727	1474.8655	1474.8606	3.26	26 - 39	1	5	K.TTLTAALTKVLSEK.Y
1631.9464	1630.9392	1630.7699	104	46 - 59	1	63	K.AFDQIDNAPEERAR.G
1712.9684	1711.9611	1711.9178	25.3	362 - 377	0	---	K.VTVELIAPVAMDEGLR.F
1729.0035	1727.9962	1727.9128	48.3	362 - 377	0	---	K.VTVELIAPVAMDEGLR.F + Oxidation (M)
1824.0974	1823.0901	1822.9438	80.2	9 - 25	0	---	R.EKPHVNVGTIGHVDHGK.T

No match to: 790.5086, 804.3324, 808.4445, 832.4057, 837.5595, 842.5625, 857.5055, 930.5245, 964.5936, 982.6070, 986.6133, 1006.6445, 1015.6326, 1027.6664, 1042.6300, 1078.6445, 1107.8344, 1142.6742, 1153.6546, 1165.6966, 1179.7073, 1197.6582, 1214.7097, 1219.6443, 1233.7159, 1240.6944, 1265.7392, 1269.7243, 1368.8298, 1380.8667, 1428.9212, 1450.8531, 1477.8489, 1484.8980, 1530.8700, 1557.9283, 1575.0154, 1598.9839, 1617.9280, 1654.9735, 1658.9825, 1708.0185, 1724.0543, 1733.0874, 1756.0868, 1781.0936, 1797.1145, 1804.0575, 1813.1035, 1839.0932, 1850.0717, 1882.1314, 1900.1334, 1915.1486, 1931.1352, 1938.1476, 1947.1417, 1949.1320, 1952.1522, 1966.1980, 1981.1803, 1994.1847, 2005.2465, 2021.1854, 2042.2539, 2044.2373, 2064.3065, 2117.3601, 2149.2508, 2181.2406, 2211.3162, 2215.2391, 2229.3298, 2271.3649, 2284.3753, 2384.2549, 2399.3191, 2438.4857, 2450.5041, 2498.5023, 2615.5335, 2645.5873, 2681.5950, 2705.4362, 2717.3922, 2729.6061, 2745.6012, 2808.6023, 2856.6076, 2872.6380, 3048.6449, 3284.6878

6. EFTU_COXBR **Mass:** 43613 **Score:** 88 **Expect:** 0.00058 **Matches:** 8

Elongation factor Tu OS=Coxiella burnetii (strain RSA 331 / Henzerling II) OX=360115 GN=tuf1 PE=3 SV=1

Observed	Mr(expt)	Mr(calc)	ppm	Start - End	Miss	Ions	Peptide
817.4935	816.4862	816.4090	94.6	286 - 291	1	---	K.REEVER.G
1186.7303	1185.7230	1185.6969	22.0	386 - 397	1	---	R.TVGAGVVTKIIE.-
1326.7883	1325.7810	1325.7231	43.7	306 - 316	1	---	K.KFEAEIYVLSK.E
1475.8727	1474.8655	1474.8606	3.26	26 - 39	1	5	K.TTLTAALTKVLSEK.Y
1631.9464	1630.9392	1630.7699	104	46 - 59	1	63	K.AFDQIDNAPEERAR.G
1712.9684	1711.9611	1711.9178	25.3	362 - 377	0	---	K.VTVELIAPVAMDEGLR.F
1729.0035	1727.9962	1727.9128	48.3	362 - 377	0	---	K.VTVELIAPVAMDEGLR.F + Oxidation (M)
1824.0974	1823.0901	1822.9438	80.2	9 - 25	0	---	R.EKPHVNVGTIGHVDHGK.T

No match to: 790.5086, 804.3324, 808.4445, 832.4057, 837.5595, 842.5625, 857.5055, 930.5245, 964.5936, 982.6070, 986.6133, 1006.6445, 1015.6326, 1027.6664, 1042.6300, 1078.6445, 1107.8344, 1142.6742, 1153.6546, 1165.6966, 1179.7073, 1197.6582, 1214.7097, 1219.6443, 1233.7159, 1240.6944, 1265.7392, 1269.7243, 1368.8298, 1380.8667, 1428.9212, 1450.8531, 1477.8489, 1484.8980, 1530.8700, 1557.9283, 1575.0154, 1598.9839, 1617.9280, 1654.9735, 1658.9825, 1708.0185, 1724.0543, 1733.0874, 1756.0868, 1781.0936, 1797.1145, 1804.0575, 1813.1035, 1839.0932, 1850.0717, 1882.1314, 1900.1334, 1915.1486, 1931.1352, 1938.1476, 1947.1417, 1949.1320, 1952.1522, 1966.1980, 1981.1803, 1994.1847, 2005.2465, 2021.1854, 2042.2539, 2044.2373, 2064.3065, 2117.3601, 2149.2508, 2181.2406, 2211.3162, 2215.2391, 2229.3298, 2271.3649, 2284.3753, 2384.2549, 2399.3191, 2438.4857, 2450.5041, 2498.5023, 2615.5335, 2645.5873, 2681.5950, 2705.4362, 2717.3922, 2729.6061, 2745.6012, 2808.6023, 2856.6076, 2872.6380, 3048.6449, 3284.6878

7. EFTU_COXBU **Mass:** 43613 **Score:** 88 **Expect:** 0.00058 **Matches:** 8

Elongation factor Tu OS=Coxiella burnetii (strain RSA 493 / Nine Mile phase I) OX=227377 GN=tufA PE=1 SV=1

Observed	Mr(expt)	Mr(calc)	ppm	Start - End	Miss	Ions	Peptide
817.4935	816.4862	816.4090	94.6	286 - 291	1	---	K.REEVER.G
1186.7303	1185.7230	1185.6969	22.0	386 - 397	1	---	R.TVGAGVVTKIIE.-
1326.7883	1325.7810	1325.7231	43.7	306 - 316	1	---	K.KFEAEIYVLSK.E
1475.8727	1474.8655	1474.8606	3.26	26 - 39	1	5	K.TTLTAALTKVLSEK.Y
1631.9464	1630.9392	1630.7699	104	46 - 59	1	63	K.AFDQIDNAPEERAR.G
1712.9684	1711.9611	1711.9178	25.3	362 - 377	0	---	K.VTVELIAPVAMDEGLR.F
1729.0035	1727.9962	1727.9128	48.3	362 - 377	0	---	K.VTVELIAPVAMDEGLR.F + Oxidation (M)
1824.0974	1823.0901	1822.9438	80.2	9 - 25	0	---	R.EKPHVNVGTIGHVDHGK.T

No match to: 790.5086, 804.3324, 808.4445, 832.4057, 837.5595, 842.5625, 857.5055, 930.5245, 964.5936, 982.6070, 986.6133, 1006.6445, 1015.6326, 1027.6664, 1042.6300, 1078.6445, 1107.8344, 1142.6742, 1153.6546, 1165.6966, 1179.7073, 1197.6582, 1214.7097, 1219.6443, 1233.7159, 1240.6944, 1265.7392, 1269.7243, 1368.8298, 1380.8667, 1428.9212, 1450.8531, 1477.8489, 1484.8980, 1530.8700, 1557.9283, 1575.0154, 1598.9839, 1617.9280, 1654.9735, 1658.9825, 1708.0185, 1724.0543, 1733.0874, 1756.0868, 1781.0936, 1797.1145, 1804.0575, 1813.1035, 1839.0932, 1850.0717, 1882.1314, 1900.1334, 1915.1486, 1931.1352, 1938.1476, 1947.1417, 1949.1320, 1952.1522, 1966.1980, 1981.1803, 1994.1847, 2005.2465, 2021.1854, 2042.2539, 2044.2373, 2064.3065, 2117.3601, 2149.2508, 2181.2406, 2211.3162, 2215.2391, 2229.3298, 2271.3649, 2284.3753, 2384.2549, 2399.3191, 2438.4857, 2450.5041, 2498.5023, 2615.5335, 2645.5873, 2681.5950, 2705.4362, 2717.3922, 2729.6061, 2745.6012, 2808.6023, 2856.6076, 2872.6380, 3048.6449, 3284.6878

8. EFTU_MARHV **Mass:** 44034 **Score:** 87 **Expect:** 0.00071 **Matches:** 5

Elongation factor Tu OS=Marinobacter hydrocarbonoclasticus (strain ATCC 700491 / DSM 11845 / VT8) OX=351348 GN=tuf PE=3 SV=1

Observed	Mr(expt)	Mr(calc)	ppm	Start - End	Miss	Ions	Peptide

1027.6664	1026.6591	1026.5822	74.9	275	284	0	---	R.AGENVGVLLR.G
1233.7159	1232.7086	1232.6091	80.8	330	338	0	10	K.GYRPQFYFR.T
1631.9464	1630.9392	1630.7699	104	47	60	1	63	R.AFDQIDNAPEERAR.G
1658.9825	1657.9752	1657.8199	93.7	196	209	0	---	K.LVEALDDYIPEPER.A
2808.6023	2807.5950	2807.2711	115	92	118	0	---	K.NMITGAAQMDGAILVCSAADGPMPQTR.E + 2 Oxidation (M)

No match to: 790.5086, 804.3324, 808.4445, 817.4935, 832.4057, 837.5595, 842.5625, 857.5055, 930.5245, 964.5936, 982.6070, 986.6133, 1006.6445, 1015.6326, 1042.6300, 1078.6445, 1107.8344, 1142.6742, 1153.6546, 1165.6966, 1179.7073, 1186.7303, 1197.6582, 1214.7097, 1219.6443, 1240.6944, 1265.7392, 1269.7243, 1326.7883, 1368.8298, 1380.8667, 1428.9212, 1450.8531, 1475.8727, 1477.8489, 1484.8980, 1530.8700, 1557.9283, 1575.0154, 1598.9839, 1617.9280, 1654.9735, 1708.0185, 1712.9684, 1724.0543, 1729.0035, 1733.0874, 1756.0868, 1781.0936, 1797.1145, 1804.0575, 1813.1035, 1824.0974, 1839.0932, 1850.0717, 1882.1314, 1900.1334, 1915.1486, 1931.1352, 1938.1476, 1947.1417, 1949.1320, 1952.1522, 1966.1980, 1981.1803, 1994.1847, 2005.2465, 2021.1854, 2042.2539, 2044.2373, 2064.3065, 2117.3601, 2149.2508, 2181.2406, 2211.3162, 2215.2391, 2229.3298, 2271.3649, 2284.3753, 2384.2549, 2399.3191, 2438.4857, 2450.5041, 2498.5023, 2615.5335, 2645.5873, 2681.5950, 2705.4362, 2717.3922, 2729.6061, 2745.6012, 2856.6076, 2872.6380, 3048.6449, 3284.6878

9. DNAK_BRADU **Mass:** 68364 **Score:** 76 **Expect:** 0.0076 **Matches:** 7

Chaperone protein DnaK OS=Bradyrhizobium diazoefficiens (strain JCM 10833 / IAM 13628 / NBRC 14792 / USDA 110) OX=224911 GN=dnaK

Observed	Mr(expt)	Mr(calc)	ppm	Start	End	Miss	Ions	Peptide
1015.6326	1014.6253	1014.5644	60.0	290	297	1	---	K.HLTMKLTR.A + Oxidation (M)
1078.6445	1077.6372	1077.5488	82.0	583	592	0	---	K.TQTLAQASMK.L
1186.7303	1185.7230	1185.6102	95.2	242	251	1	---	K.EQGINLRNDK.L
1658.9825	1657.9752	1657.8320	86.4	448	462	0	---	K.MLGQFDLMGIPPAPR.G + Oxidation (M)
1966.1980	1965.1907	1964.9547	120	4	22	0	36	K.VIGIDLGTTNSCVAVMDGK.N + Oxidation (M)
2229.3298	2228.3225	2228.1835	62.4	318	340	1	25	K.ALKDAGVTAGEIGEVVLVGGMSR.M
2450.5041	2449.4968	2449.1440	144	441	462	1	---	R.EMAADNKMLGQFDLMGIPPAPR.G + 3 Oxidation (M)

No match to: 790.5086, 804.3324, 808.4445, 817.4935, 832.4057, 837.5595, 842.5625, 857.5055, 930.5245, 964.5936, 982.6070, 986.6133, 1006.6445, 1027.6664, 1042.6300, 1107.8344, 1142.6742, 1153.6546, 1165.6966, 1179.7073, 1197.6582, 1214.7097, 1219.6443, 1233.7159, 1240.6944, 1265.7392, 1269.7243, 1326.7883, 1368.8298, 1380.8667, 1428.9212, 1450.8531, 1475.8727, 1477.8489, 1484.8980, 1530.8700, 1557.9283, 1575.0154, 1598.9839, 1617.9280, 1631.9464, 1654.9735, 1708.0185, 1712.9684, 1724.0543, 1729.0035, 1733.0874, 1756.0868, 1781.0936, 1797.1145, 1804.0575, 1813.1035, 1824.0974, 1839.0932, 1850.0717, 1882.1314, 1900.1334, 1915.1486, 1931.1352, 1938.1476, 1947.1417, 1949.1320, 1952.1522, 1981.1803, 1994.1847, 2005.2465, 2021.1854, 2042.2539, 2044.2373, 2064.3065, 2117.3601, 2149.2508, 2181.2406, 2211.3162, 2215.2391, 2271.3649, 2284.3753, 2384.2549, 2399.3191, 2438.4857, 2498.5023, 2615.5335, 2645.5873, 2681.5950, 2705.4362, 2717.3922, 2729.6061, 2745.6012, 2808.6023, 2856.6076, 2872.6380, 3048.6449, 3284.6878

10. ADH_THEBR **Mass:** 37851 **Score:** 75 **Expect:** 0.0096 **Matches:** 7

NADP-dependent isopropanol dehydrogenase OS=Thermoanaerobacter brockii OX=29323 GN=adh PE=1 SV=1

Observed	Mr(expt)	Mr(calc)	ppm	Start	End	Miss	Ions	Peptide
1042.6300	1041.6227	1041.5502	69.7	292	301	1	---	K.GGLCPGGRLR.M
1214.7097	1213.7024	1213.6199	68.0	1	11	1	---	-.MKGFAMLSIGK.V + 2 Oxidation (M)
1240.6944	1239.6871	1239.6355	41.6	333	342	1	---	K.AFMLMKDKPK.D + 2 Oxidation (M)
1530.8700	1529.8628	1529.7483	74.8	279	291	1	---	R.LEWGCGMAHKTIK.G
1631.9464	1630.9392	1630.7872	93.2	220	234	0	48	K.DGPIESQIMNLTEGK.G
2229.3298	2228.3225	2228.0545	120	92	111	1	---	R.TSEVQRGYHQHSGGMLAGWK.F
3048.6449	3047.6376	3047.5135	40.7	53	80	1	---	R.HNMILGHEAVGEVVEVGSEVKDFKPGDR.V

No match to: 790.5086, 804.3324, 808.4445, 817.4935, 832.4057, 837.5595, 842.5625, 857.5055, 930.5245, 964.5936, 982.6070, 986.6133, 1006.6445, 1015.6326, 1027.6664, 1078.6445, 1107.8344, 1142.6742, 1153.6546, 1165.6966, 1179.7073, 1186.7303, 1197.6582, 1219.6443, 1233.7159, 1265.7392, 1269.7243, 1326.7883, 1368.8298, 1380.8667, 1428.9212, 1450.8531, 1475.8727, 1477.8489, 1484.8980, 1557.9283, 1575.0154, 1598.9839, 1617.9280, 1654.9735, 1658.9825, 1708.0185, 1712.9684, 1724.0543, 1729.0035, 1733.0874, 1756.0868, 1781.0936, 1797.1145, 1804.0575, 1813.1035, 1824.0974, 1839.0932, 1850.0717, 1882.1314, 1900.1334, 1915.1486, 1931.1352, 1938.1476, 1947.1417, 1949.1320, 1952.1522, 1966.1980, 1981.1803, 1994.1847, 2005.2465, 2021.1854, 2042.2539, 2044.2373, 2064.3065, 2117.3601, 2149.2508, 2181.2406, 2211.3162, 2215.2391, 2271.3649, 2284.3753, 2384.2549, 2399.3191, 2438.4857, 2450.5041, 2498.5023, 2615.5335, 2645.5873, 2681.5950, 2705.4362, 2717.3922, 2729.6061, 2745.6012, 2808.6023, 2856.6076, 2872.6380, 3284.6878

11. TOP1_STAS1 **Mass:** 79764 **Score:** 75 **Expect:** 0.01 **Matches:** 12

DNA topoisomerase 1 OS=Staphylococcus saprophyticus subsp. saprophyticus (strain ATCC 15305 / DSM 20229) OX=342451 GN=topA PE=3

Observed	Mr(expt)	Mr(calc)	ppm	Start	End	Miss	Ions	Peptide
1006.6445	1005.6372	1005.5607	76.1	401	409	1	---	R.ANGQTIKFK.G
1186.7303	1185.7230	1185.6003	104	358	366	1	---	K.NFLTRDQHR.L
1240.6944	1239.6871	1239.5924	76.4	184	192	0	---	R.NFKPEEYWK.I
1269.7243	1268.7171	1268.6976	15.4	2	13	0	---	M.AENLVIVESPAK.A
1368.8298	1367.8225	1367.8249	-1.72	167	178	1	---	R.VQSVALRLVIDR.E
1450.8531	1449.8459	1449.7874	40.3	27	39	1	---	K.VIASMGHVRDLPR.S
1475.8727	1474.8655	1474.7086	106	127	139	0	26	R.GIEMELVDAQQAR.R + Oxidation (M)
1631.9464	1630.9392	1630.8097	79.4	127	140	1	29	R.GIEMELVDAQQARR.I + Oxidation (M)
1850.0717	1849.0644	1848.9264	74.6	124	139	1	---	K.HPRGIEMELVDAQQAR.R
1882.1314	1881.1241	1880.9740	79.8	274	289	1	---	K.TMMLAQQLYEGIDLKK.Q
1938.1476	1937.1403	1936.9351	106	334	351	0	---	K.GQGDQDAHEAIRPSSTLR.T
2229.3298	2228.3225	2228.0419	126	40	58	1	---	R.SQMGVDAENDYEPKYITIR.G

No match to: 790.5086, 804.3324, 808.4445, 817.4935, 832.4057, 837.5595, 842.5625, 857.5055, 930.5245, 964.5936, 982.6070, 986.6133, 1015.6326, 1027.6664, 1042.6300, 1078.6445, 1107.8344, 1142.6742, 1153.6546, 1165.6966, 1179.7073, 1197.6582, 1214.7097, 1219.6443, 1233.7159, 1265.7392, 1326.7883, 1380.8667, 1428.9212, 1477.8489, 1484.8980, 1530.8700, 1557.9283, 1575.0154, 1598.9839, 1617.9280, 1654.9735, 1658.9825, 1708.0185, 1712.9684, 1724.0543, 1729.0035, 1733.0874, 1756.0868, 1781.0936, 1797.1145, 1804.0575, 1813.1035, 1824.0974, 1839.0932, 1900.1334, 1915.1486, 1931.1352, 1947.1417, 1949.1320, 1952.1522, 1966.1980, 1981.1803, 1994.1847, 2005.2465, 2021.1854, 2042.2539, 2044.2373, 2064.3065, 2117.3601, 2149.2508, 2181.2406, 2211.3162, 2215.2391, 2271.3649, 2284.3753, 2384.2549, 2399.3191, 2438.4857, 2450.5041, 2498.5023, 2615.5335, 2645.5873, 2681.5950, 2705.4362, 2717.3922, 2729.6061, 2745.6012, 2808.6023, 2856.6076, 2872.6380, 3048.6449, 3284.6878

12. HSCA_AROAE **Mass:** 66854 **Score:** 74 **Expect:** 0.014 **Matches:** 12

Chaperone protein HscA homolog OS=Aromatoleum aromaticum (strain EbN1) OX=76114 GN=hscA PE=3 SV=1

Observed	Mr(expt)	Mr(calc)	ppm	Start	End	Miss	Ions	Peptide
808.4445	807.4373	807.4425	-6.52	89	95	1	---	R.FMGRGLK.D
832.4057	831.3984	831.4450	-56.00	165	172	1	---	R.QATKDAAK.L
857.5055	856.4982	856.5130	-17.28	587	594	1	---	K.KGIEALAR.A
964.5936	963.5863	963.5171	71.8	81	88	1	---	K.NTIMSVKR.F + Oxidation (M)
1240.6944	1239.6871	1239.7299	-34.52	169	180	1	---	K.DAAKLAGLNVLR.L
1368.8298	1367.8225	1367.7409	59.7	321	333	1	---	K.VLRDAGLGPEDVK.G
1617.9280	1616.9207	1616.8093	68.9	51	64	1	---	R.SMLPSIVRYHADGR.I + Oxidation (M)
1824.0974	1823.0901	1822.9577	72.6	416	431	1	---	R.NSTLPIARAQEFTTFK.D
2149.2508	2148.2435	2148.0092	109	432	450	1	---	K.DGQTAMAFHVVQGEREMVK.D + Oxidation (M)
2211.3162	2210.3090	2209.9950	142	96	115	0	---	K.DVSHVESTPYDFIDAGGMVR.L + Oxidation (M)
2229.3298	2228.3225	2228.1405	81.7	38	58	1	33	R.NGIAVCLADEAGRSMLPSIVR.Y
2399.3191	2398.3118	2398.2162	39.9	65	87	1	---	R.IEVGQTAAAAHTTDPKNTIMSVK.R + Oxidation (M)

No match to: 790.5086, 804.3324, 817.4935, 837.5595, 842.5625, 930.5245, 982.6070, 986.6133, 1006.6445, 1015.6326, 1027.6664, 1042.6300, 1078.6445, 1107.8344, 1142.6742, 1153.6546, 1165.6966, 1179.7073, 1186.7303, 1197.6582, 1214.7097, 1219.6443, 1233.7159, 1265.7392, 1269.7243, 1326.7883, 1380.8667, 1428.9212, 1450.8531, 1475.8727, 1477.8489, 1484.8980, 1530.8700, 1557.9283, 1575.0154, 1598.9839, 1631.9464, 1654.9735, 1658.9825, 1708.0185, 1712.9684, 1724.0543, 1729.0035, 1733.0874, 1756.0868, 1781.0936, 1797.1145, 1804.0575, 1813.1035, 1839.0932, 1850.0717, 1882.1314, 1900.1334, 1915.1486, 1931.1352, 1938.1476, 1947.1417, 1949.1320, 1952.1522, 1966.1980, 1981.1803, 1994.1847, 2005.2465, 2021.1854, 2042.2539, 2044.2373, 2064.3065, 2117.3601, 2181.2406, 2215.2391, 2271.3649, 2284.3753, 2384.2549, 2438.4857, 2450.5041, 2498.5023, 2615.5335, 2645.5873, 2681.5950, 2705.4362, 2717.3922, 2729.6061, 2745.6012, 2808.6023, 2856.6076, 2872.6380, 3048.6449, 3284.6878

13. LEU1_BUCUN **Mass:** 56434 **Score:** 72 **Expect:** 0.022 **Matches:** 6

2-isopropylmalate synthase (Fragment) OS=Buchnera aphidicola subsp. Uroleucon sonchi OX=118118 GN=leuA PE=3 SV=1

Observed	Mr(expt)	Mr(calc)	ppm	Start	End	Miss	Ions	Peptide
790.5086	789.5013	789.4345	84.7	256	262	0	---	R.TSQVISR.I
930.5245	929.5172	929.5658	-52.29	311	318	1	---	K.VKLNLTSR.S
1475.8727	1474.8655	1474.7701	64.7	223	236	0	39	R.AGNTALEEVIMAIK.V + Oxidation (M)
1724.0543	1723.0470	1722.8498	114	296	310	1	---	R.ENYEIMDPSSIGLKK.V
1882.1314	1881.1241	1880.8938	122	294	309	1	---	K.NRENYEIMDPSSIGLK.K + Oxidation (M)
1966.1980	1965.1907	1964.9554	120	25	43	0	19	K.AGVDIMEVGFPISSPGDFK.S

No match to: 804.3324, 808.4445, 817.4935, 832.4057, 837.5595, 842.5625, 857.5055, 964.5936, 982.6070, 986.6133, 1006.6445, 1015.6326, 1027.6664, 1042.6300, 1078.6445, 1107.8344, 1142.6742, 1153.6546, 1165.6966, 1179.7073, 1186.7303, 1197.6582, 1214.7097, 1219.6443, 1233.7159, 1240.6944, 1265.7392, 1269.7243, 1326.7883, 1368.8298, 1380.8667, 1428.9212, 1450.8531, 1477.8489, 1484.8980, 1530.8700, 1557.9283, 1575.0154, 1598.9839, 1617.9280, 1631.9464, 1654.9735, 1658.9825, 1708.0185, 1712.9684, 1729.0035, 1733.0874, 1756.0868, 1781.0936, 1797.1145, 1804.0575, 1813.1035, 1824.0974, 1839.0932, 1850.0717, 1900.1334, 1915.1486, 1931.1352, 1938.1476, 1947.1417, 1949.1320, 1952.1522, 1981.1803, 1994.1847, 2005.2465, 2021.1854, 2042.2539, 2044.2373, 2064.3065, 2117.3601, 2149.2508, 2181.2406, 2211.3162, 2215.2391, 2229.3298, 2271.3649, 2284.3753, 2384.2549, 2399.3191, 2438.4857, 2450.5041, 2498.5023, 2615.5335, 2645.5873, 2681.5950, 2705.4362, 2717.3922, 2729.6061, 2745.6012, 2808.6023, 2856.6076, 2872.6380, 3048.6449, 3284.6878

14. SECA_PROM4 **Mass:** 107505 **Score:** 71 **Expect:** 0.025 **Matches:** 12

Protein translocase subunit SecA OS=Prochlorococcus marinus (strain MIT 9211) OX=93059 GN=secA PE=3 SV=1

Observed	Mr(expt)	Mr(calc)	ppm	Start	End	Miss	Ions	Peptide
808.4445	807.4373	807.4239	16.6	41	47	1	---	R.AKTADFR.E
857.5055	856.4982	856.5018	-4.18	589	595	0	---	K.VLIDLER.K
1428.9212	1427.9139	1427.7197	136	299	310	0	---	K.DPWAHYITNALK.A
1598.9839	1597.9766	1597.8689	67.4	128	141	1	---	R.GVHVVTVNDYLARR.D
1631.9464	1630.9392	1630.8712	41.7	242	257	1	17	K.AAEVVASLIRAAEMGK.D + Oxidation (M)
1824.0974	1823.0901	1822.9247	90.7	507	523	1	---	R.GTDIILGGNSDYMARLK.L
1947.1417	1946.1345	1945.9680	85.5	325	342	1	---	R.NGEAVIVDEFTGRVMPGR.R
1966.1980	1965.1907	1965.1272	32.3	532	548	1	31	K.LVKPEDGHKPPVPLQRR.S
2042.2539	2041.2467	2041.1208	61.7	59	75	1	---	K.QRELLDEILPEVFSVVR.E
2149.2508	2148.2435	2147.9544	135	928	945	1	---	R.NVIYSMFMFQPAPESDKE.- + Oxidation (M)
2384.2549	2383.2476	2383.1664	34.1	86	106	0	---	R.HFDVQLIGGMVLHEGQIAEMK.T + 2 Oxidation (M)
2856.6076	2855.6003	2855.3946	72.0	86	111	1	---	R.HFDVQLIGGMVLHEGQIAEMKTGEGK.T + 2 Oxidation (M)

No match to: 790.5086, 804.3324, 817.4935, 832.4057, 837.5595, 842.5625, 930.5245, 964.5936, 982.6070, 986.6133, 1006.6445, 1015.6326, 1027.6664, 1042.6300, 1078.6445, 1107.8344, 1142.6742, 1153.6546, 1165.6966, 1179.7073, 1186.7303, 1197.6582, 1214.7097, 1219.6443, 1233.7159, 1240.6944, 1265.7392, 1269.7243, 1326.7883, 1368.8298, 1380.8667, 1450.8531, 1475.8727, 1477.8489, 1484.8980, 1530.8700, 1557.9283, 1575.0154, 1617.9280, 1654.9735, 1658.9825, 1708.0185, 1712.9684, 1724.0543, 1729.0035, 1733.0874, 1756.0868, 1781.0936, 1797.1145, 1804.0575, 1813.1035, 1839.0932, 1850.0717, 1882.1314, 1900.1334, 1915.1486, 1931.1352, 1938.1476, 1949.1320, 1952.1522, 1981.1803, 1994.1847, 2005.2465, 2021.1854, 2044.2373, 2064.3065, 2117.3601, 2181.2406, 2211.3162, 2215.2391, 2229.3298, 2271.3649, 2284.3753, 2399.3191, 2438.4857, 2450.5041, 2498.5023, 2615.5335, 2645.5873, 2681.5950, 2705.4362, 2717.3922, 2729.6061, 2745.6012, 2808.6023, 2872.6380, 3048.6449, 3284.6878

15. RPOA_PROM1 **Mass:** 34244 **Score:** 71 **Expect:** 0.026 **Matches:** 5

DNA-directed RNA polymerase subunit alpha OS=Prochlorococcus marinus (strain NATL1A) OX=167555 GN=rpoA PE=3 SV=1

Observed	Mr(expt)	Mr(calc)	ppm	Start	End	Miss	Ions	Peptide
804.3324	803.3251	803.4137	-110.29	84	90	0	---	K.QISVDSR.S
1197.6582	1196.6509	1196.7129	-51.78	99	110	1	---	R.LVVTGPADVKAK.D
1475.8727	1474.8655	1474.7814	57.0	43	57	0	28	R.VLMGGLEGSAVTAVR.I + Oxidation (M)
1631.9464	1630.9392	1630.8825	34.8	42	57	1	27	R.RVLMGGLEGSAVTAVR.I + Oxidation (M)
2117.3601	2116.3529	2116.0834	127	73	90	1	---	R.EDVLDILLNCKQISVDSR.S

No match to: 790.5086, 808.4445, 817.4935, 832.4057, 837.5595, 842.5625, 857.5055, 930.5245, 964.5936, 982.6070, 986.6133, 1006.6445, 1015.6326, 1027.6664, 1042.6300, 1078.6445, 1107.8344, 1142.6742, 1153.6546, 1165.6966, 1179.7073, 1186.7303, 1214.7097, 1219.6443, 1233.7159, 1240.6944, 1265.7392, 1269.7243, 1326.7883, 1368.8298, 1380.8667, 1428.9212, 1450.8531, 1477.8489, 1484.8980, 1530.8700, 1557.9283, 1575.0154, 1598.9839, 1617.9280, 1654.9735, 1658.9825, 1708.0185, 1712.9684, 1724.0543, 1729.0035, 1733.0874, 1756.0868, 1781.0936, 1797.1145, 1804.0575, 1813.1035, 1824.0974, 1839.0932, 1850.0717, 1882.1314, 1900.1334, 1915.1486, 1931.1352, 1938.1476, 1947.1417, 1949.1320, 1952.1522, 1966.1980, 1981.1803, 1994.1847, 2005.2465, 2021.1854, 2042.2539, 2044.2373, 2064.3065, 2149.2508, 2181.2406, 2211.3162, 2215.2391, 2229.3298, 2271.3649, 2284.3753, 2384.2549, 2399.3191, 2438.4857, 2450.5041, 2498.5023, 2615.5335, 2645.5873, 2681.5950, 2705.4362, 2717.3922, 2729.6061, 2745.6012, 2808.6023, 2856.6076, 2872.6380, 3048.6449, 3284.6878

16. RPOA_PROMT **Mass:** 34244 **Score:** 71 **Expect:** 0.026 **Matches:** 5

DNA-directed RNA polymerase subunit alpha OS=Prochlorococcus marinus (strain NATL2A) OX=59920 GN=rpoA PE=3 SV=1

Observed	Mr(expt)	Mr(calc)	ppm	Start	End	Miss	Ions	Peptide
804.3324	803.3251	803.4137	-110.29	84	90	0	---	K.QISVDSR.S
1197.6582	1196.6509	1196.7129	-51.78	99	110	1	---	R.LVVTGPADVKAK.D
1475.8727	1474.8655	1474.7814	57.0	43	57	0	28	R.VLMGGLEGSAVTAVR.I + Oxidation (M)
1631.9464	1630.9392	1630.8825	34.8	42	57	1	27	R.RVLMGGLEGSAVTAVR.I + Oxidation (M)
2117.3601	2116.3529	2116.0834	127	73	90	1	---	R.EDVLDILLNCKQISVDSR.S

No match to: 790.5086, 808.4445, 817.4935, 832.4057, 837.5595, 842.5625, 857.5055, 930.5245, 964.5936, 982.6070, 986.6133, 1006.6445, 1015.6326, 1027.6664, 1042.6300, 1078.6445, 1107.8344, 1142.6742, 1153.6546, 1165.6966, 1179.7073, 1186.7303, 1214.7097, 1219.6443, 1233.7159, 1240.6944, 1265.7392, 1269.7243, 1326.7883, 1368.8298, 1380.8667, 1428.9212, 1450.8531, 1477.8489, 1484.8980, 1530.8700, 1557.9283, 1575.0154, 1598.9839, 1617.9280, 1654.9735, 1658.9825, 1708.0185, 1712.9684, 1724.0543, 1729.0035, 1733.0874, 1756.0868, 1781.0936, 1797.1145, 1804.0575, 1813.1035, 1824.0974, 1839.0932, 1850.0717, 1882.1314, 1900.1334, 1915.1486, 1931.1352, 1938.1476, 1947.1417, 1949.1320, 1952.1522, 1966.1980, 1981.1803, 1994.1847, 2005.2465, 2021.1854, 2042.2539, 2044.2373, 2064.3065, 2149.2508, 2181.2406, 2211.3162, 2215.2391, 2229.3298, 2271.3649, 2284.3753, 2384.2549, 2399.3191, 2438.4857, 2450.5041, 2498.5023, 2615.5335, 2645.5873, 2681.5950, 2705.4362, 2717.3922, 2729.6061, 2745.6012, 2808.6023, 2856.6076, 2872.6380, 3048.6449, 3284.6878

17. PROA_BRUSU **Mass:** 44300 **Score:** 69 **Expect:** 0.039 **Matches:** 9

Gamma-glutamyl phosphate reductase OS=Brucella suis biovar 1 (strain 1330) OX=204722 GN=proA PE=3 SV=1

Observed	Mr(expt)	Mr(calc)	ppm	Start	End	Miss	Ions	Peptide
1015.6326	1014.6253	1014.5345	89.5	80	88	0	---	R.IDAIAEDIR.A
1475.8727	1474.8655	1474.7450	81.7	258	271	0	21	R.TGICGAAETLLVDR.A
1631.9464	1630.9392	1630.8461	57.1	257	271	1	---	R.RTGICGAAETLLVDR.A
1756.0868	1755.0795	1754.9162	93.0	47	62	1	---	R.ADILEANRLDLANAEK.N
1813.1035	1812.0963	1811.9741	67.4	73	88	1	---	R.LTLNEARIDAIAEDIR.A

1938.1476	1937.1403	1936.9312	108	55 -	72	1	---	R.LDLANAEKNGMAASFVDR.L + Oxidation (M)
1966.1980	1965.1907	1965.1081	42.0	189 -	208	1	12	R.AAVGEMLKGLGGAIDVIVPR.G
2181.2406	2180.2333	2180.1549	36.0	34 -	54	1	---	K.ALNAAADAILEARADILEANR.L
2681.5950	2680.5877	2680.3795	77.7	217 -	239	1	---	R.VQSEARVPVFAHLEGICHLYIDK.S

No match to: 790.5086, 804.3324, 808.4445, 817.4935, 832.4057, 837.5595, 842.5625, 857.5055, 930.5245, 964.5936, 982.6070, 986.6133, 1006.6445, 1027.6664, 1042.6300, 1078.6445, 1107.8344, 1142.6742, 1153.6546, 1165.6966, 1179.7073, 1186.7303, 1197.6582, 1214.7097, 1219.6443, 1233.7159, 1240.6944, 1265.7392, 1269.7243, 1326.7883, 1368.8298, 1380.8667, 1428.9212, 1450.8531, 1477.8489, 1484.8980, 1530.8700, 1557.9283, 1575.0154, 1598.9839, 1617.9280, 1654.9735, 1658.9825, 1708.0185, 1712.9684, 1724.0543, 1729.0035, 1733.0874, 1781.0936, 1797.1145, 1804.0575, 1824.0974, 1839.0932, 1850.0717, 1882.1314, 1900.1334, 1915.1486, 1931.1352, 1947.1417, 1949.1320, 1952.1522, 1981.1803, 1994.1847, 2005.2465, 2021.1854, 2042.2539, 2044.2373, 2064.3065, 2117.3601, 2149.2508, 2211.3162, 2215.2391, 2229.3298, 2271.3649, 2284.3753, 2384.2549, 2399.3191, 2438.4857, 2450.5041, 2498.5023, 2615.5335, 2645.5873, 2705.4362, 2717.3922, 2729.6061, 2745.6012, 2808.6023, 2856.6076, 2872.6380, 3048.6449, 3284.6878

18. EFTU_HERAU **Mass:** 43768 **Score:** 69 **Expect:** 0.04 **Matches:** 3

Elongation factor Tu OS=Herpetosiphon aurantiacus OX=65 GN=tuf PE=3 SV=1

Observed	Mr(expt)	Mr(calc)	ppm	Start	End	Miss	Ions	Peptide
804.3324	803.3251	803.4436	-147.44	35 -	41	1	---	K.TMALRGR.A
1631.9464	1630.9392	1630.7699	104	46 -	59	1	63	R.AFDQIDNAPEERAR.G
1947.1417	1946.1345	1946.0513	42.7	156 -	172	1	---	R.ELLTKYGFPGDEIPIVR.G

No match to: 790.5086, 808.4445, 817.4935, 832.4057, 837.5595, 842.5625, 857.5055, 930.5245, 964.5936, 982.6070, 986.6133, 1006.6445, 1015.6326, 1027.6664, 1042.6300, 1078.6445, 1107.8344, 1142.6742, 1153.6546, 1165.6966, 1179.7073, 1186.7303, 1197.6582, 1214.7097, 1219.6443, 1233.7159, 1240.6944, 1265.7392, 1269.7243, 1326.7883, 1368.8298, 1380.8667, 1428.9212, 1450.8531, 1475.8727, 1477.8489, 1484.8980, 1530.8700, 1557.9283, 1575.0154, 1598.9839, 1617.9280, 1654.9735, 1658.9825, 1708.0185, 1712.9684, 1724.0543, 1729.0035, 1733.0874, 1756.0868, 1781.0936, 1797.1145, 1804.0575, 1813.1035, 1824.0974, 1839.0932, 1850.0717, 1882.1314, 1900.1334, 1915.1486, 1931.1352, 1938.1476, 1949.1320, 1952.1522, 1966.1980, 1981.1803, 1994.1847, 2005.2465, 2021.1854, 2042.2539, 2044.2373, 2064.3065, 2117.3601, 2149.2508, 2181.2406, 2211.3162, 2215.2391, 2229.3298, 2271.3649, 2284.3753, 2384.2549, 2399.3191, 2438.4857, 2450.5041, 2498.5023, 2615.5335, 2645.5873, 2681.5950, 2705.4362, 2717.3922, 2729.6061, 2745.6012, 2808.6023, 2856.6076, 2872.6380, 3048.6449, 3284.6878

19. EFTU1_HALHL **Mass:** 43283 **Score:** 69 **Expect:** 0.04 **Matches:** 3

Elongation factor Tu 1 OS=Halorhodospira halophila (strain DSM 244 / SL1) OX=349124 GN=tuf1 PE=3 SV=1

Observed	Mr(expt)	Mr(calc)	ppm	Start	End	Miss	Ions	Peptide
817.4935	816.4862	816.4705	19.2	385 -	393	0	---	R.TVGAGVVSK.I
1631.9464	1630.9392	1630.7699	104	46 -	59	1	63	R.AFDQIDNAPEERAR.G
1654.9735	1653.9662	1653.8686	59.0	267 -	282	0	---	K.LLDQGEAGDNIGALLR.G

No match to: 790.5086, 804.3324, 808.4445, 832.4057, 837.5595, 842.5625, 857.5055, 930.5245, 964.5936, 982.6070, 986.6133, 1006.6445, 1015.6326, 1027.6664, 1042.6300, 1078.6445, 1107.8344, 1142.6742, 1153.6546, 1165.6966, 1179.7073, 1186.7303, 1197.6582, 1214.7097, 1219.6443, 1233.7159, 1240.6944, 1265.7392, 1269.7243, 1326.7883, 1368.8298, 1380.8667, 1428.9212, 1450.8531, 1475.8727, 1477.8489, 1484.8980, 1530.8700, 1557.9283, 1575.0154, 1598.9839, 1617.9280, 1658.9825, 1708.0185, 1712.9684, 1724.0543, 1729.0035, 1733.0874, 1756.0868, 1781.0936, 1797.1145, 1804.0575, 1813.1035, 1824.0974, 1839.0932, 1850.0717, 1882.1314, 1900.1334, 1915.1486, 1931.1352, 1938.1476, 1947.1417, 1949.1320, 1952.1522, 1966.1980, 1981.1803, 1994.1847, 2005.2465, 2021.1854, 2042.2539, 2044.2373, 2064.3065, 2117.3601, 2149.2508, 2181.2406, 2211.3162, 2215.2391, 2229.3298, 2271.3649, 2284.3753, 2384.2549, 2399.3191, 2438.4857, 2450.5041, 2498.5023, 2615.5335, 2645.5873, 2681.5950, 2705.4362, 2717.3922, 2729.6061, 2745.6012, 2808.6023, 2856.6076, 2872.6380, 3048.6449, 3284.6878

20. EFTU2_HALHL **Mass:** 43269 **Score:** 69 **Expect:** 0.04 **Matches:** 3

Elongation factor Tu 2 OS=Halorhodospira halophila (strain DSM 244 / SL1) OX=349124 GN=tuf2 PE=3 SV=1

Observed	Mr(expt)	Mr(calc)	ppm	Start	End	Miss	Ions	Peptide
817.4935	816.4862	816.4705	19.2	385 -	393	0	---	R.TVGAGVVSK.I
1631.9464	1630.9392	1630.7699	104	46 -	59	1	63	R.AFDQIDNAPEERAR.G
1654.9735	1653.9662	1653.8686	59.0	267 -	282	0	---	K.LLDQGEAGDNIGALLR.G

No match to: 790.5086, 804.3324, 808.4445, 832.4057, 837.5595, 842.5625, 857.5055, 930.5245, 964.5936, 982.6070, 986.6133, 1006.6445, 1015.6326, 1027.6664, 1042.6300, 1078.6445, 1107.8344, 1142.6742, 1153.6546, 1165.6966, 1179.7073, 1186.7303, 1197.6582, 1214.7097, 1219.6443, 1233.7159, 1240.6944, 1265.7392, 1269.7243, 1326.7883, 1368.8298, 1380.8667, 1428.9212, 1450.8531, 1475.8727, 1477.8489, 1484.8980, 1530.8700, 1557.9283, 1575.0154, 1598.9839, 1617.9280, 1658.9825, 1708.0185, 1712.9684, 1724.0543, 1729.0035, 1733.0874, 1756.0868, 1781.0936, 1797.1145, 1804.0575, 1813.1035, 1824.0974, 1839.0932, 1850.0717, 1882.1314, 1900.1334, 1915.1486, 1931.1352, 1938.1476, 1947.1417, 1949.1320, 1952.1522, 1966.1980, 1981.1803, 1994.1847, 2005.2465, 2021.1854, 2042.2539, 2044.2373, 2064.3065, 2117.3601, 2149.2508, 2181.2406, 2211.3162, 2215.2391, 2229.3298, 2271.3649, 2284.3753, 2384.2549, 2399.3191, 2438.4857, 2450.5041, 2498.5023, 2615.5335, 2645.5873, 2681.5950, 2705.4362, 2717.3922, 2729.6061, 2745.6012, 2808.6023, 2856.6076, 2872.6380, 3048.6449, 3284.6878

Search Parameters

Type of search : MS/MS Ion Search
Enzyme : Trypsin
Fixed modifications : Carbamidomethyl (C)
Variable modifications : Oxidation (M)
Mass values : Monoisotopic
Protein Mass : Unrestricted
Peptide Mass Tolerance : ± 150 ppm
Fragment Mass Tolerance: ± 2 Da
Max Missed Cleavages : 1
Instrument type : MALDI-TOF-TOF
Query1 (790.5086,1+): <no title>
Query2 (804.3324,1+): <no title>
Query3 (808.4445,1+): <no title>
Query4 (817.4935,1+): <no title>
Query5 (832.4057,1+): <no title>
Query6 (837.5595,1+): <no title>
Query7 (842.5625,1+): <no title>
Query8 (857.5055,1+): <no title>
Query9 (930.5245,1+): <no title>
Query10 (964.5936,1+): <no title>
Query11 (982.6070,1+): <no title>
Query12 (986.6133,1+): <no title>
Query13 (1006.6445,1+): <no title>
Query14 (1015.6326,1+): <no title>
Query15 (1027.6664,1+): <no title>
Query16 (1042.6300,1+): <no title>
Query17 (1078.6445,1+): <no title>
Query18 (1107.8344,1+): <no title>
Query19 (1142.6742,1+): <no title>
Query20 (1153.6546,1+): <no title>
Query21 (1165.6966,1+): <no title>
Query22 (1179.7073,1+): <no title>
Query23 (1186.7303,1+): <no title>
Query24 (1197.6582,1+): <no title>
Query25 (1214.7097,1+): <no title>
Query26 (1219.6443,1+): <no title>

```
 Query27 (1233.7159,1+): <no title>
 Query28 (1240.6944,1+): <no title>
 Query29 (1265.7392,1+): <no title>
 Query30 (1269.7243,1+): <no title>
 Query31 (1326.7883,1+): <no title>
 Query32 (1368.8298,1+): <no title>
 Query33 (1380.8667,1+): <no title>
 Query34 (1428.9212,1+): <no title>
 Query35 (1450.8531,1+): <no title>
 Query36 (1475.8727,1+): <no title>
 Query37 (1477.8489,1+): <no title>
 Query38 (1484.8980,1+): <no title>
 Query39 (1530.8700,1+): <no title>
 Query40 (1557.9283,1+): <no title>
 Query41 (1575.0154,1+): <no title>
 Query42 (1598.9839,1+): <no title>
 Query43 (1617.9280,1+): <no title>
 Query44 (1631.9464,1+): <no title>
 Query45 (1654.9735,1+): <no title>
 Query46 (1658.9825,1+): <no title>
 Query47 (1708.0185,1+): <no title>
 Query48 (1712.9684,1+): <no title>
 Query49 (1724.0543,1+): <no title>
 Query50 (1729.0035,1+): <no title>
 Query51 (1733.0874,1+): <no title>
 Query52 (1756.0868,1+): <no title>
 Query53 (1781.0936,1+): <no title>
 Query54 (1797.1145,1+): <no title>
 Query55 (1804.0575,1+): <no title>
 Query56 (1813.1035,1+): <no title>
 Query57 (1824.0974,1+): <no title>
 Query58 (1839.0932,1+): <no title>
 Query59 (1850.0717,1+): <no title>
 Query60 (1882.1314,1+): <no title>
 Query61 (1900.1334,1+): <no title>
 Query62 (1915.1486,1+): <no title>
 Query63 (1931.1352,1+): <no title>
 Query64 (1938.1476,1+): <no title>
 Query65 (1947.1417,1+): <no title>
 Query66 (1949.1320,1+): <no title>
 Query67 (1952.1522,1+): <no title>
 Query68 (1966.1980,1+): <no title>
 Query69 (1981.1803,1+): <no title>
 Query70 (1994.1847,1+): <no title>
 Query71 (2005.2465,1+): <no title>
 Query72 (2021.1854,1+): <no title>
 Query73 (2042.2539,1+): <no title>
 Query74 (2044.2373,1+): <no title>
 Query75 (2064.3065,1+): <no title>
 Query76 (2117.3601,1+): <no title>
 Query77 (2149.2508,1+): <no title>
 Query78 (2181.2406,1+): <no title>
 Query79 (2211.3162,1+): <no title>
 Query80 (2215.2391,1+): <no title>
 Query81 (2229.3298,1+): <no title>
 Query82 (2271.3649,1+): <no title>
 Query83 (2284.3753,1+): <no title>
 Query84 (2384.2549,1+): <no title>
 Query85 (2399.3191,1+): <no title>
 Query86 (2438.4857,1+): <no title>
 Query87 (2450.5041,1+): <no title>
 Query88 (2498.5023,1+): <no title>
 Query89 (2615.5335,1+): <no title>
 Query90 (2645.5873,1+): <no title>
 Query91 (2681.5950,1+): <no title>
 Query92 (2705.4362,1+): <no title>
 Query93 (2717.3922,1+): <no title>
 Query94 (2729.6061,1+): <no title>
 Query95 (2745.6012,1+): <no title>
 Query96 (2808.6023,1+): <no title>
 Query97 (2856.6076,1+): <no title>
 Query98 (2872.6380,1+): <no title>
 Query99 (3048.6449,1+): <no title>
Query100 (3284.6878,1+): <no title>
```

Mascot: http://www.matrixscience.com/

MALDI MS MS Data Sheet of Urine Isolate

C:\Users\madhurekha\Desktop\MALDI_Data\16th batch data 070718\BHU\4\0_B4\1\1549.8852.LIFT\1SRef

Comment 1

Comment 2

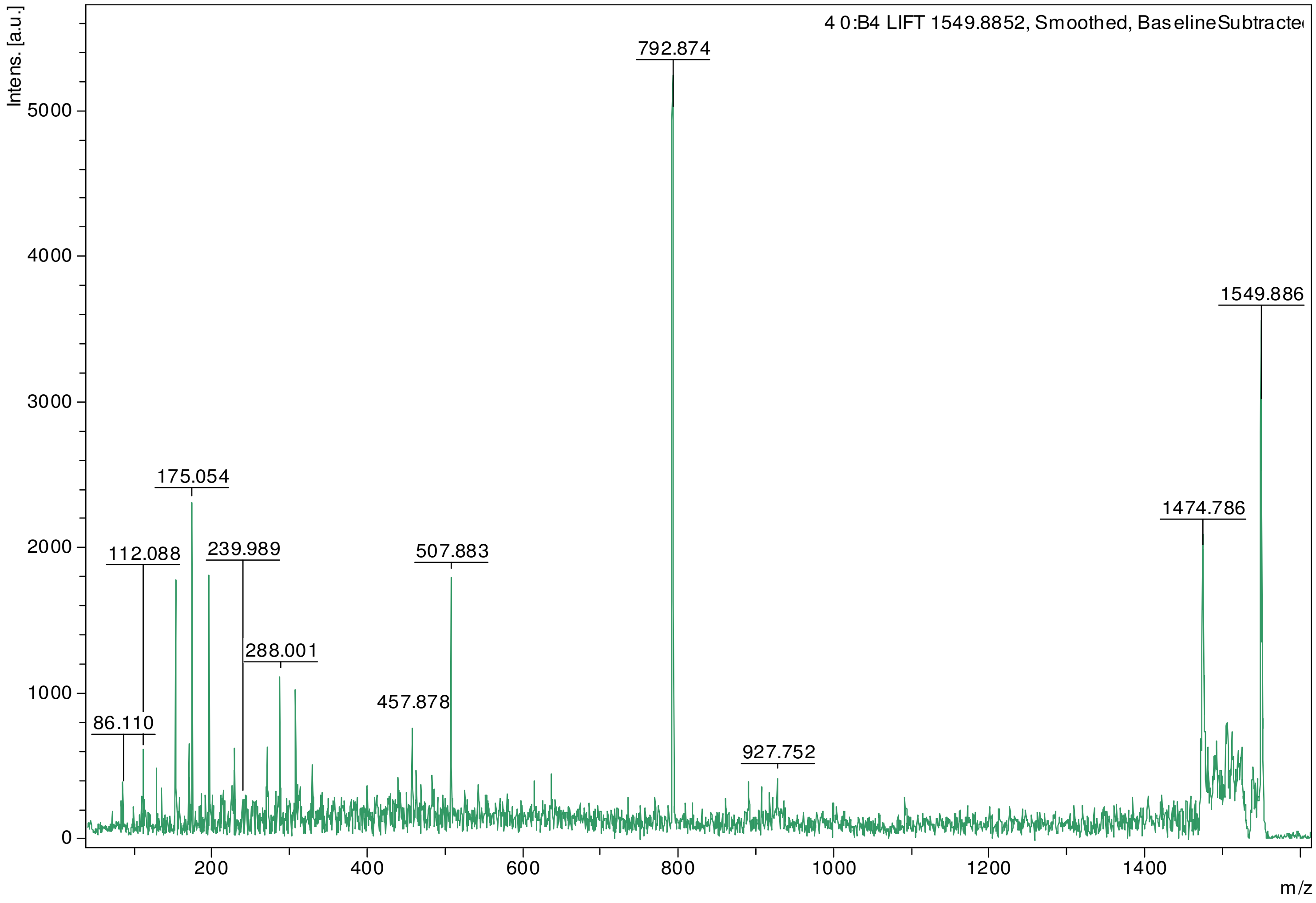

m/z	S/N	Quality Fac.	Res.	Intens.	Area
84.068	3.3	50	756	242.18	30
86.110	5.0	159	584	362.70	59
100.086	3.3	47	969	237.91	27
112.088	8.4	902	927	611.99	83
129.090	6.9	553	1113	498.01	65
136.056	5.0	375	1236	354.06	44
154.042	25.7	7002	1274	1802.78	250
171.046	9.8	232	1235	685.35	111
175.054	33.4	134020	1263	2326.66	377
187.055	4.6	63	1413	317.18	49
192.051	4.6	150	1230	317.69	59
197.043	26.2	14133	1353	1832.65	316
215.022	4.8	54	1311	342.32	67
217.033	3.6	63	1178	255.60	56
239.989	4.1	34	1363	299.93	64
254.003	3.8	34	1181	285.44	75
288.001	14.0	3247	1392	1140.48	294
308.098	11.9	1759	1506	1026.27	265
313.985	4.3	36	1287	374.91	116
328.951	6.3	35	1530	582.30	160
342.941	3.6	36	1204	340.02	125
457.878	6.3	173	1864	822.18	277
463.897	3.7	42	1734	482.20	178
507.883	12.8	14729	1942	1822.50	672
792.874	31.5	26310	2026	4995.12	3261
927.752	3.1	48	2494	450.85	302
1472.429	5.1	73	1637	557.19	1242
1474.786	18.0	4905	2006	1989.73	3628
1477.907	3.6	70	1640	389.04	869
1481.154	4.7	235	1938	514.35	979
1491.241	7.0	48	2756	737.59	1000
1494.643	4.0	159	1661	413.17	939
1497.937	3.5	100	2729	363.21	501
1500.212	3.9	36	2628	402.09	579
1504.691	8.6	138	2632	875.96	1264
1506.579	5.6	88	2728	572.35	799
1510.589	4.7	78	2731	468.12	656
1512.524	6.1	156	1811	604.22	1288

C:\Users\madhurekha\Desktop\MALDI_Data\16th batch data 070718\BHU\4\0_B4\1\1549.8852.LIFT\1SRef

m/z	S/N	Quality Fac.	Res.	Intens.	Area
1517.668	4.9	115	1684	473.46	1089
1520.891	5.5	189	1689	527.18	1212
1524.039	5.9	275	1693	563.19	1294
1538.552	5.7	95	2245	538.33	950
1543.084	3.7	235	3365	346.10	410
1549.886	32.7	18955	3045	2986.80	3937

C:\Users\madhurekha\Desktop\MALDI_Data\16th batch data 070718\BHU\4\0_B4\1\1989.1187.LIFT\1SRef

Comment 1

Comment 2

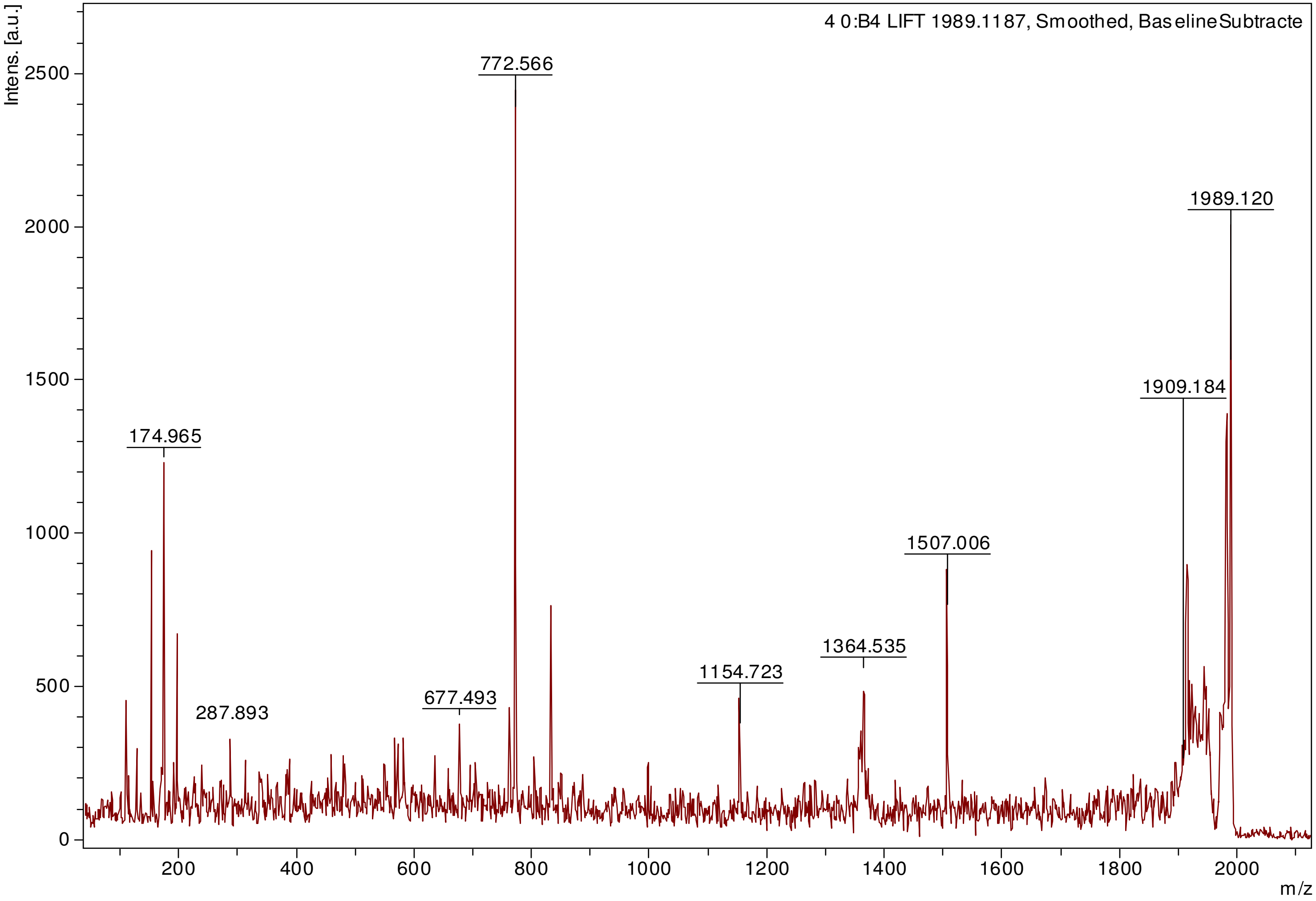

m/z	S/N	Quality Fac.	Res.	Intens.	Area
110.008	6.9	11791	1096	452.00	51
112.017	6.6	746	1105	432.46	49
115.006	3.2	569	1208	209.33	22
129.017	4.8	356	1267	303.09	35
153.970	15.4	23766	1430	959.92	118
171.951	3.9	40	1150	244.45	43
174.965	20.0	29380	1367	1232.30	184
191.939	4.3	153	1068	264.17	56
196.941	11.3	4058	1240	695.09	131
239.885	3.9	42	1370	247.64	53
287.893	5.3	190	1040	365.12	126
313.817	3.6	31	1222	255.23	83
388.741	3.1	121	1571	261.04	85
582.549	3.1	51	1742	331.58	163
677.493	3.5	94	2172	392.44	191
762.515	4.1	279	2409	448.94	233
772.566	21.9	45367	2257	2374.99	1341
833.469	6.5	1019	2339	696.85	424
1154.723	3.5	171	2826	364.71	307
1364.535	4.9	175	2763	544.67	626
1507.006	6.6	1520	1891	753.36	1517
1909.184	3.0	31	4067	250.10	368
1914.977	10.0	720	2747	813.11	1775
1920.499	4.4	128	2206	336.87	919
1923.700	5.6	190	2489	446.19	1081
1928.734	4.9	120	2492	389.35	944
1935.686	4.3	109	2150	315.11	890
1939.152	4.2	65	2154	319.44	902
1944.389	7.4	244	2157	527.89	1493
1947.635	3.8	168	2389	283.20	724
1950.867	4.5	154	2166	334.99	947
1971.236	5.2	222	2401	361.47	933
1974.294	4.2	191	2192	290.77	824
1977.810	4.7	210	3621	334.64	575
1980.041	4.6	118	4427	320.73	451
1982.323	17.2	5856	4284	1198.11	1745
1985.696	5.7	86	3259	393.13	754
1989.120	22.5	18551	3049	1549.41	3182

C:\Users\madhurekha\Desktop\MALDI_Data\16th batch data 070718\BHU\4\0_B4\1\2705.3299.LIFT\1SRef

Comment 1

Comment 2

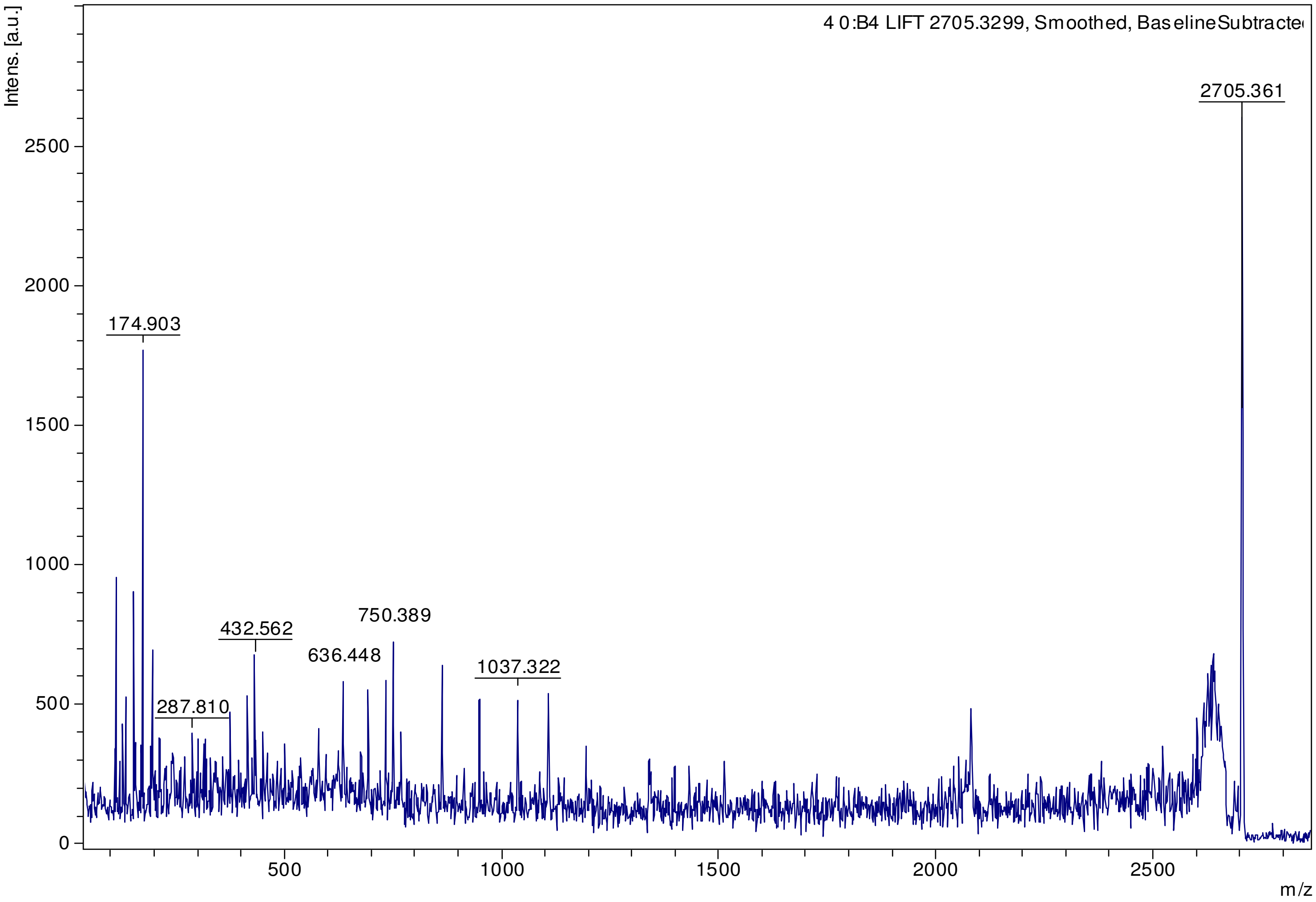

m/z	S/N	Quality Fac.	Res.	Intens.	Area
111.949	7.7	3570	701	879.65	158
128.947	3.5	300	686	395.68	84
135.910	4.6	1225	1452	518.75	55
153.901	7.9	7500	1378	884.01	113
157.907	3.5	47	1112	391.59	64
170.892	3.5	52	1386	386.79	56
174.903	16.2	22161	1567	1780.25	232
191.865	3.0	380	1306	332.14	58
196.880	6.4	1273	1517	696.09	107
213.864	3.8	109	1495	422.51	72
246.813	3.0	72	1460	337.90	70
271.797	3.1	41	1586	360.25	76
287.810	3.4	113	1152	401.88	125
301.711	3.2	95	2092	385.45	70
318.707	3.0	37	1465	377.66	104
375.642	3.2	94	1262	442.48	173
415.600	4.0	116	1447	578.26	223
432.562	4.6	134	1687	672.08	233
636.448	3.8	143	1605	612.30	370
693.370	3.6	175	2165	569.49	287
733.351	3.8	114	2028	594.00	346
750.389	5.0	777	1955	773.36	483
863.339	4.5	825	2222	665.55	449
950.391	3.7	368	2107	518.67	427
1037.322	4.0	266	2701	553.28	407
1108.270	3.2	84	2122	447.44	469
2624.440	5.5	178	3925	489.81	1138
2629.634	4.1	58	2923	356.91	1117
2634.529	3.6	63	2925	313.11	981
2638.928	5.2	103	2933	416.48	1305
2643.993	4.5	95	2936	365.00	1145
2649.931	3.9	66	2943	316.70	996
2705.361	19.7	14471	4251	1547.18	3477

C:\Users\madhurekha\Desktop\MALDI_Data\16th batch data 070718\BHU\4\0_B4\1\1SRef

Comment 1

Comment 2

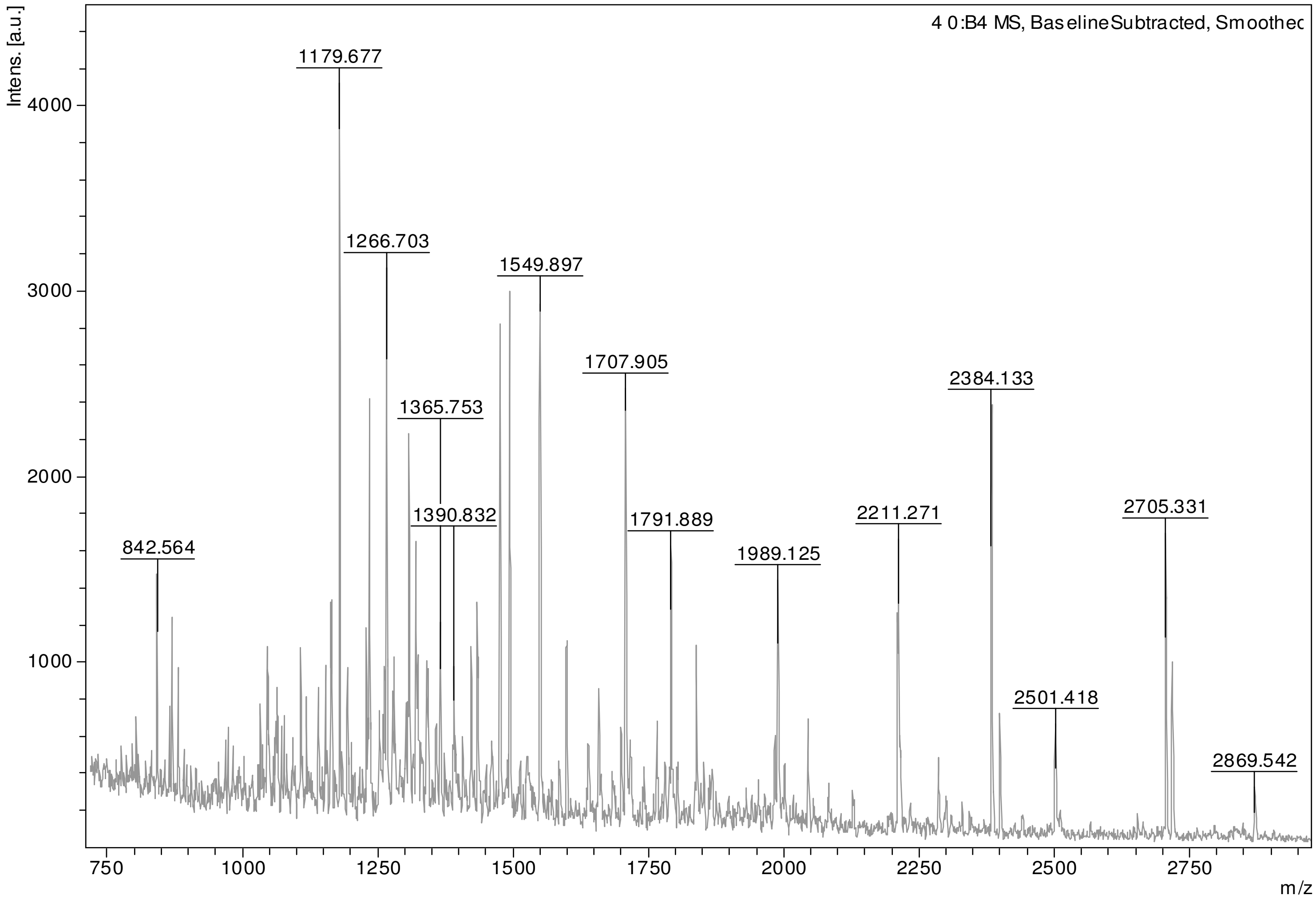

m/z	S/N	Quality Fac.	Res.	Intens.	Area
842.564	8.5	1479	4124	1141.12	406
870.594	6.3	810	3626	843.69	360
1107.621	7.0	368	4361	920.00	480
1153.695	6.6	435	7420	864.70	296
1162.697	6.4	79	5812	840.57	356
1164.675	7.2	202	5022	945.43	466
1179.677	29.4	13665	5948	3848.40	1651
1193.698	6.3	216	6222	828.88	347
1229.897	8.1	861	6044	1057.70	470
1234.747	19.4	1998	5959	2515.46	1162
1266.703	20.2	7660	5763	2607.41	1294
1280.752	6.7	596	5593	860.29	449
1308.753	11.9	2233	5063	1524.58	903
1320.680	8.4	366	4093	1070.68	798
1340.777	6.2	847	5061	785.04	486
1365.753	7.5	1696	5083	940.11	599
1390.832	6.2	571	4547	770.56	566
1422.803	7.2	1515	5520	886.07	563
1434.864	9.2	2839	6133	1119.94	654
1475.856	22.1	8771	6540	2640.49	1515
1493.845	22.8	12552	6061	2706.52	1720
1549.897	25.0	18485	6858	2870.36	1737
1598.938	9.2	6046	6343	1023.43	712
1657.932	6.1	633	4757	649.91	642
1707.905	22.7	25644	6707	2328.44	1744
1791.889	13.1	2963	5653	1260.03	1223
1839.063	8.8	5216	6673	816.14	712
1989.125	14.0	8035	6866	1079.87	1006
2045.190	6.4	1553	5619	454.15	531
2211.271	22.9	11216	9070	1293.53	1038
2384.133	35.3	15290	8029	1597.91	1625
2399.201	10.1	6130	8689	449.30	425
2501.418	10.3	1439	6624	403.84	528
2705.331	37.2	60552	9753	1110.08	1115
2717.253	19.7	3369	7836	580.75	734
2869.542	6.6	1077	6568	160.65	262

MATRIX SCIENCE Mascot Search Results

```
User          : sanovar
Email         : sanovar@sandor.co.in
Search title  :
MS data file  : DATA.TXT
Database      : SwissProt 2018_06 (557713 sequences; 200130199 residues)
Taxonomy      : Bacteria (Eubacteria) (333552 sequences)
Timestamp     : 11 Jul 2018 at 12:08:07 GMT
Warning       : A Peptide summary report will usually give a much clearer picture of MS/MS search results.
Top Score     : 75 for TKT1_ECOLI, Transketolase 1 OS=Escherichia coli (strain K12) OX=83333 GN=tktA PE=1 SV=5
```

Mascot Score Histogram

Protein score is -10*Log(P), where P is the probability that the observed match is a random event.
Protein scores greater than 68 are significant (p<0.05).
Protein scores are derived from ions scores as a non-probabilistic basis for ranking protein hits.

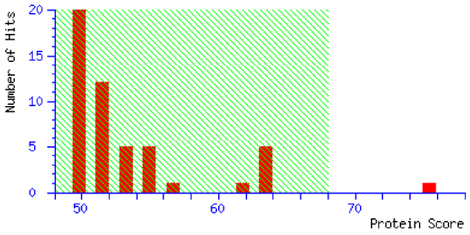

Protein Summary Report

Format As | Protein Summary (deprecated) | Help

Significance threshold p< 0.05 Max. number of hits 20

Preferred taxonomy All entries

Re-Search All | Search Unmatched

Index

	Accession	Mass	Score	Description
1.	TKT1_ECOLI	72451	75	Transketolase 1 OS=Escherichia coli (strain K12) OX=83333 GN=tktA PE=1 SV=5
2.	RS1_DICD3	61334	63	30S ribosomal protein S1 OS=Dickeya dadantii (strain 3937) OX=198628 GN=rpsA PE=3 SV=2
3.	RS1_ECO57	61235	63	30S ribosomal protein S1 OS=Escherichia coli O157:H7 OX=83334 GN=rpsA PE=3 SV=1
4.	RS1_ECOL6	61235	63	30S ribosomal protein S1 OS=Escherichia coli O6:H1 (strain CFT073 / ATCC 700928 / UPEC) OX=199310 GN=rp
5.	RS1_ECOLI	61235	63	30S ribosomal protein S1 OS=Escherichia coli (strain K12) OX=83333 GN=rpsA PE=1 SV=1
6.	RS1_SHIFL	61235	63	30S ribosomal protein S1 OS=Shigella flexneri OX=623 GN=rpsA PE=3 SV=1
7.	ATPG_BACCA	32323	61	ATP synthase gamma chain OS=Bacillus caldotenax OX=1395 GN=atpG PE=3 SV=1
8.	DXR_CLOBM	43041	57	1-deoxy-D-xylulose 5-phosphate reductoisomerase OS=Clostridium botulinum (strain Loch Maree / Type A3)
9.	DXR_CLOB6	43168	56	1-deoxy-D-xylulose 5-phosphate reductoisomerase OS=Clostridium botulinum (strain 657 / Type Ba4) OX=515
10.	DXR_CLOBJ	43182	56	1-deoxy-D-xylulose 5-phosphate reductoisomerase OS=Clostridium botulinum (strain Kyoto / Type A2) OX=53
11.	DXR_CLOBL	43212	56	1-deoxy-D-xylulose 5-phosphate reductoisomerase OS=Clostridium botulinum (strain Langeland / NCTC 10281
12.	DXR_CLOBK	43169	55	1-deoxy-D-xylulose 5-phosphate reductoisomerase OS=Clostridium botulinum (strain Okra / Type B1) OX=498
13.	ERA_LACH4	34027	54	GTPase Era OS=Lactobacillus helveticus (strain DPC 4571) OX=405566 GN=era PE=3 SV=1
14.	ASSY_SOLUE	50458	54	Argininosuccinate synthase OS=Solibacter usitatus (strain Ellin6076) OX=234267 GN=argG PE=3 SV=1
15.	IOLG_RUBXD	37446	54	Inositol 2-dehydrogenase OS=Rubrobacter xylanophilus (strain DSM 9941 / NBRC 16129) OX=266117 GN=iolG P
16.	UREG_RHOPB	22253	53	Urease accessory protein UreG OS=Rhodopseudomonas palustris (strain BisB18) OX=316056 GN=ureG PE=3 SV=1
17.	CCPA_STAEQ	36500	53	Catabolite control protein A OS=Staphylococcus epidermidis (strain ATCC 35984 / RP62A) OX=176279 GN=ccp
18.	CCPA_STAES	36500	53	Catabolite control protein A OS=Staphylococcus epidermidis (strain ATCC 12228) OX=176280 GN=ccpA PE=3 S
19.	ACKA_PORG3	43586	52	Acetate kinase OS=Porphyromonas gingivalis (strain ATCC 33277 / DSM 20709 / CIP 103683 / JCM 12257 / NC
20.	RPOC_PSYCK	155806	52	DNA-directed RNA polymerase subunit beta' OS=Psychrobacter cryohalolentis (strain K5) OX=335284 GN=rpoC

Results List

1. TKT1_ECOLI **Mass:** 72451 **Score:** 75 **Expect:** 0.0096 **Matches:** 6
Transketolase 1 OS=Escherichia coli (strain K12) OX=83333 GN=tktA PE=1 SV=5

Observed	Mr(expt)	Mr(calc)	ppm	Start	End	Miss	Ions	Peptide
1033.5958	1032.5885	1032.5604	27.2	245 -	254	0	---	K.TIIGFGSPNK.A
1277.7589	1276.7516	1276.6353	91.1	205 -	214	0	---	R.FEAYGWHVIR.D
1329.7326	1328.7254	1328.7452	-14.93	343 -	354	1	---	K.EFIAKLQANPAK.I
1983.1414	1982.1341	1982.0261	54.5	604 -	621	1	---	K.AVTARVAVEAGIADYWYK.Y
1989.1250	1988.1177	1987.9712	73.7	255 -	274	0	63	K.AGTHDSHGAPLGDAEIALTR.E
2001.0988	2000.0915	1999.9309	80.3	580 -	597	1	---	R.VVSMPSTDAFDKQDAAYR.E

No match to: 720.3201, 721.3315, 724.3214, 804.3108, 842.5642, 865.5456, 870.5940, 881.3242, 969.5548, 973.5948, 982.5138, 1037.6072, 1045.6282, 1047.6804, 1064.6488, 1072.6447, 1077.2496, 1082.6362, 1093.6181, 1107.6212, 1109.5910, 1118.5838, 1140.6515, 1153.6950, 1163.6855, 1164.6754, 1165.6684, 1179.6771, 1184.6858, 1193.6977, 1195.6893, 1201.7343, 1229.8974, 1232.6901, 1253.7005, 1259.2801, 1259.7540, 1263.2247, 1263.7024, 1266.7035, 1280.7521, 1283.7721, 1302.8009, 1306.7653, 1308.7528, 1310.3027, 1320.6798, 1323.2817, 1323.7637, 1324.7890, 1326.3207, 1332.7618, 1340.7775, 1343.7777, 1357.7890, 1365.7525, 1373.7532, 1390.8324, 1393.8069, 1407.7954, 1422.8033, 1434.8643, 1460.8550, 1475.8557, 1490.8367, 1493.8451, 1549.8969, 1598.9377, 1638.9642, 1657.9317, 1684.0126, 1699.9639, 1707.9051, 1716.9715, 1740.9356, 1781.0033, 1789.0003, 1791.8887, 1804.0297, 1839.0630, 1847.0491, 1852.0436, 2083.1533, 2128.1603, 2211.2706, 2215.3315, 2286.2807, 2299.3189, 2329.2726, 2343.2471, 2384.1325, 2399.2011, 2441.2881, 2501.4182, 2510.3224, 2705.3310, 2717.2530, 2848.5165, 2869.5420, 3223.3745, 3264.5742, 3312.3559

2. RS1_DICD3 **Mass:** 61334 **Score:** 63 **Expect:** 0.18 **Matches:** 2
30S ribosomal protein S1 OS=Dickeya dadantii (strain 3937) OX=198628 GN=rpsA PE=3 SV=2

Observed	Mr(expt)	Mr(calc)	ppm	Start	End	Miss	Ions	Peptide
1329.7326	1328.7254	1328.7664	-30.87	451 -	464	1	---	K.GAIVTGKVTAVDAK.G
1549.8969	1548.8896	1548.7784	71.8	465 -	479	0	63	K.GATVELADGVEGYLR.A

No match to: 720.3201, 721.3315, 724.3214, 804.3108, 842.5642, 865.5456, 870.5940, 881.3242, 969.5548, 973.5948, 982.5138, 1033.5958, 1037.6072, 1045.6282, 1047.6804, 1064.6488, 1072.6447, 1077.2496, 1082.6362, 1093.6181, 1107.6212, 1109.5910,

1118.5838, 1140.6515, 1153.6950, 1163.6855, 1164.6754, 1165.6684, 1179.6771, 1184.6858, 1193.6977, 1195.6893, 1201.7343, 1229.8974, 1232.6901, 1253.7005, 1259.2801, 1259.7540, 1263.2247, 1263.7024, 1266.7035, 1277.7589, 1280.7521, 1283.7721, 1302.8009, 1306.7653, 1308.7528, 1310.3027, 1320.6798, 1323.2817, 1323.7637, 1324.7890, 1326.3207, 1332.7618, 1340.7775, 1343.7777, 1357.7890, 1365.7525, 1373.7532, 1390.8324, 1393.8069, 1407.7954, 1422.8033, 1434.8643, 1460.8550, 1475.8557, 1490.8367, 1493.8451, 1598.9377, 1638.9642, 1657.9317, 1684.0126, 1699.9639, 1707.9051, 1716.9715, 1740.9356, 1781.0033, 1789.0003, 1791.8887, 1804.0297, 1839.0630, 1847.0491, 1852.0436, 1983.1414, 1989.1250, 2001.0988, 2083.1533, 2128.1603, 2211.2706, 2215.3315, 2286.2807, 2299.3189, 2329.2726, 2343.2471, 2384.1325, 2399.2011, 2441.2881, 2501.4182, 2510.3224, 2705.3310, 2717.2530, 2848.5165, 2869.5420, 3223.3745, 3264.5742, 3312.3559

3. RS1_ECO57 **Mass:** 61235 **Score:** 63 **Expect:** 0.18 **Matches:** 2
30S ribosomal protein S1 OS=Escherichia coli O157:H7 OX=83334 GN=rpsA PE=3 SV=1

Observed	Mr(expt)	Mr(calc)	ppm	Start	End	Miss	Ions	Peptide
1329.7326	1328.7254	1328.7664	-30.87	451	464	1	---	K.GAIVTGKVTAVDAK.G
1549.8969	1548.8896	1548.7784	71.8	465	479	0	63	K.GATVELADGVEGYLR.A

No match to: 720.3201, 721.3315, 724.3214, 804.3108, 842.5642, 865.5456, 870.5940, 881.3242, 969.5548, 973.5948, 982.5138, 1033.5958, 1037.6072, 1045.6282, 1047.6804, 1064.6488, 1072.6447, 1077.2496, 1082.6362, 1093.6181, 1107.6212, 1109.5910, 1118.5838, 1140.6515, 1153.6950, 1163.6855, 1164.6754, 1165.6684, 1179.6771, 1184.6858, 1193.6977, 1195.6893, 1201.7343, 1229.8974, 1232.6901, 1253.7005, 1259.2801, 1259.7540, 1263.2247, 1263.7024, 1266.7035, 1277.7589, 1280.7521, 1283.7721, 1302.8009, 1306.7653, 1308.7528, 1310.3027, 1320.6798, 1323.2817, 1323.7637, 1324.7890, 1326.3207, 1332.7618, 1340.7775, 1343.7777, 1357.7890, 1365.7525, 1373.7532, 1390.8324, 1393.8069, 1407.7954, 1422.8033, 1434.8643, 1460.8550, 1475.8557, 1490.8367, 1493.8451, 1598.9377, 1638.9642, 1657.9317, 1684.0126, 1699.9639, 1707.9051, 1716.9715, 1740.9356, 1781.0033, 1789.0003, 1791.8887, 1804.0297, 1839.0630, 1847.0491, 1852.0436, 1983.1414, 1989.1250, 2001.0988, 2083.1533, 2128.1603, 2211.2706, 2215.3315, 2286.2807, 2299.3189, 2329.2726, 2343.2471, 2384.1325, 2399.2011, 2441.2881, 2501.4182, 2510.3224, 2705.3310, 2717.2530, 2848.5165, 2869.5420, 3223.3745, 3264.5742, 3312.3559

4. RS1_ECOL6 **Mass:** 61235 **Score:** 63 **Expect:** 0.18 **Matches:** 2
30S ribosomal protein S1 OS=Escherichia coli O6:H1 (strain CFT073 / ATCC 700928 / UPEC) OX=199310 GN=rpsA PE=3 SV=1

Observed	Mr(expt)	Mr(calc)	ppm	Start	End	Miss	Ions	Peptide
1329.7326	1328.7254	1328.7664	-30.87	451	464	1	---	K.GAIVTGKVTAVDAK.G
1549.8969	1548.8896	1548.7784	71.8	465	479	0	63	K.GATVELADGVEGYLR.A

No match to: 720.3201, 721.3315, 724.3214, 804.3108, 842.5642, 865.5456, 870.5940, 881.3242, 969.5548, 973.5948, 982.5138, 1033.5958, 1037.6072, 1045.6282, 1047.6804, 1064.6488, 1072.6447, 1077.2496, 1082.6362, 1093.6181, 1107.6212, 1109.5910, 1118.5838, 1140.6515, 1153.6950, 1163.6855, 1164.6754, 1165.6684, 1179.6771, 1184.6858, 1193.6977, 1195.6893, 1201.7343, 1229.8974, 1232.6901, 1253.7005, 1259.2801, 1259.7540, 1263.2247, 1263.7024, 1266.7035, 1277.7589, 1280.7521, 1283.7721, 1302.8009, 1306.7653, 1308.7528, 1310.3027, 1320.6798, 1323.2817, 1323.7637, 1324.7890, 1326.3207, 1332.7618, 1340.7775, 1343.7777, 1357.7890, 1365.7525, 1373.7532, 1390.8324, 1393.8069, 1407.7954, 1422.8033, 1434.8643, 1460.8550, 1475.8557, 1490.8367, 1493.8451, 1598.9377, 1638.9642, 1657.9317, 1684.0126, 1699.9639, 1707.9051, 1716.9715, 1740.9356, 1781.0033, 1789.0003, 1791.8887, 1804.0297, 1839.0630, 1847.0491, 1852.0436, 1983.1414, 1989.1250, 2001.0988, 2083.1533, 2128.1603, 2211.2706, 2215.3315, 2286.2807, 2299.3189, 2329.2726, 2343.2471, 2384.1325, 2399.2011, 2441.2881, 2501.4182, 2510.3224, 2705.3310, 2717.2530, 2848.5165, 2869.5420, 3223.3745, 3264.5742, 3312.3559

5. RS1_ECOLI **Mass:** 61235 **Score:** 63 **Expect:** 0.18 **Matches:** 2
30S ribosomal protein S1 OS=Escherichia coli (strain K12) OX=83333 GN=rpsA PE=1 SV=1

Observed	Mr(expt)	Mr(calc)	ppm	Start	End	Miss	Ions	Peptide
1329.7326	1328.7254	1328.7664	-30.87	451	464	1	---	K.GAIVTGKVTAVDAK.G
1549.8969	1548.8896	1548.7784	71.8	465	479	0	63	K.GATVELADGVEGYLR.A

No match to: 720.3201, 721.3315, 724.3214, 804.3108, 842.5642, 865.5456, 870.5940, 881.3242, 969.5548, 973.5948, 982.5138, 1033.5958, 1037.6072, 1045.6282, 1047.6804, 1064.6488, 1072.6447, 1077.2496, 1082.6362, 1093.6181, 1107.6212, 1109.5910, 1118.5838, 1140.6515, 1153.6950, 1163.6855, 1164.6754, 1165.6684, 1179.6771, 1184.6858, 1193.6977, 1195.6893, 1201.7343, 1229.8974, 1232.6901, 1253.7005, 1259.2801, 1259.7540, 1263.2247, 1263.7024, 1266.7035, 1277.7589, 1280.7521, 1283.7721, 1302.8009, 1306.7653, 1308.7528, 1310.3027, 1320.6798, 1323.2817, 1323.7637, 1324.7890, 1326.3207, 1332.7618, 1340.7775, 1343.7777, 1357.7890, 1365.7525, 1373.7532, 1390.8324, 1393.8069, 1407.7954, 1422.8033, 1434.8643, 1460.8550, 1475.8557, 1490.8367, 1493.8451, 1598.9377, 1638.9642, 1657.9317, 1684.0126, 1699.9639, 1707.9051, 1716.9715, 1740.9356, 1781.0033, 1789.0003, 1791.8887, 1804.0297, 1839.0630, 1847.0491, 1852.0436, 1983.1414, 1989.1250, 2001.0988, 2083.1533, 2128.1603, 2211.2706, 2215.3315, 2286.2807, 2299.3189, 2329.2726, 2343.2471, 2384.1325, 2399.2011, 2441.2881, 2501.4182, 2510.3224, 2705.3310, 2717.2530, 2848.5165, 2869.5420, 3223.3745, 3264.5742, 3312.3559

6. RS1_SHIFL **Mass:** 61235 **Score:** 63 **Expect:** 0.18 **Matches:** 2
30S ribosomal protein S1 OS=Shigella flexneri OX=623 GN=rpsA PE=3 SV=1

Observed	Mr(expt)	Mr(calc)	ppm	Start	End	Miss	Ions	Peptide
1329.7326	1328.7254	1328.7664	-30.87	451	464	1	---	K.GAIVTGKVTAVDAK.G
1549.8969	1548.8896	1548.7784	71.8	465	479	0	63	K.GATVELADGVEGYLR.A

No match to: 720.3201, 721.3315, 724.3214, 804.3108, 842.5642, 865.5456, 870.5940, 881.3242, 969.5548, 973.5948, 982.5138, 1033.5958, 1037.6072, 1045.6282, 1047.6804, 1064.6488, 1072.6447, 1077.2496, 1082.6362, 1093.6181, 1107.6212, 1109.5910, 1118.5838, 1140.6515, 1153.6950, 1163.6855, 1164.6754, 1165.6684, 1179.6771, 1184.6858, 1193.6977, 1195.6893, 1201.7343, 1229.8974, 1232.6901, 1253.7005, 1259.2801, 1259.7540, 1263.2247, 1263.7024, 1266.7035, 1277.7589, 1280.7521, 1283.7721, 1302.8009, 1306.7653, 1308.7528, 1310.3027, 1320.6798, 1323.2817, 1323.7637, 1324.7890, 1326.3207, 1332.7618, 1340.7775, 1343.7777, 1357.7890, 1365.7525, 1373.7532, 1390.8324, 1393.8069, 1407.7954, 1422.8033, 1434.8643, 1460.8550, 1475.8557, 1490.8367, 1493.8451, 1598.9377, 1638.9642, 1657.9317, 1684.0126, 1699.9639, 1707.9051, 1716.9715, 1740.9356, 1781.0033, 1789.0003, 1791.8887, 1804.0297, 1839.0630, 1847.0491, 1852.0436, 1983.1414, 1989.1250, 2001.0988, 2083.1533, 2128.1603, 2211.2706, 2215.3315, 2286.2807, 2299.3189, 2329.2726, 2343.2471, 2384.1325, 2399.2011, 2441.2881, 2501.4182, 2510.3224, 2705.3310, 2717.2530, 2848.5165, 2869.5420, 3223.3745, 3264.5742, 3312.3559

7. ATPG_BACCA **Mass:** 32323 **Score:** 61 **Expect:** 0.27 **Matches:** 12
ATP synthase gamma chain OS=Bacillus caldotenax OX=1395 GN=atpG PE=3 SV=1

Observed	Mr(expt)	Mr(calc)	ppm	Start	End	Miss	Ions	Peptide
1140.6515	1139.6442	1139.7026	-51.22	2	11	1	---	M.KPLASLRDIK.T
1164.6754	1163.6681	1163.6008	57.8	42	50	0	---	R.EIVRPYMEK.I
1266.7035	1265.6962	1265.6914	3.77	64	74	0	---	R.SHPMLVSRPVK.K + Oxidation (M)
1320.6798	1319.6725	1319.7019	-22.31	41	50	1	---	K.REIVRPYMEK.I
1332.7618	1331.7545	1331.6867	50.9	51	63	0	---	K.IQEVVAMSASAAR.S
1343.7777	1342.7704	1342.7391	23.3	130	140	1	---	K.RNMPVILDITR.L + Oxidation (M)
1373.7532	1372.7459	1372.7602	-10.43	189	200	0	---	K.LLPLTDFLAENK.Q
1657.9317	1656.9244	1656.9199	2.75	189	202	1	---	K.LLPLTDFLAENKQR.T
1699.9639	1698.9566	1698.8941	36.8	141	155	1	---	R.LPDQPSFADIKEIAR.K
1781.0033	1779.9960	1779.8243	96.5	243	258	1	---	R.MTAMKNATDNANDVIR.T + Oxidation (M)
2399.2011	2398.1938	2398.2566	-26.20	131	151	1	---	R.NMPVILDITRLPDQPSFADIK.E + Oxidation (M)
2510.3224	2509.3151	2509.2668	19.2	42	63	1	---	R.EIVRPYMEKIQEVVAMSASAAR.S + 2 Oxidation (M)

No match to: 720.3201, 721.3315, 724.3214, 804.3108, 842.5642, 865.5456, 870.5940, 881.3242, 969.5548, 973.5948, 982.5138, 1033.5958, 1037.6072, 1045.6282, 1047.6804, 1064.6488, 1072.6447, 1077.2496, 1082.6362, 1093.6181, 1107.6212, 1109.5910, 1118.5838, 1153.6950, 1163.6855, 1165.6684, 1179.6771, 1184.6858, 1193.6977, 1195.6893, 1201.7343, 1229.8974, 1232.6901, 1253.7005, 1259.2801, 1259.7540, 1263.2247, 1263.7024, 1277.7589, 1280.7521, 1283.7721, 1302.8009, 1306.7653, 1308.7528, 1310.3027, 1323.2817, 1323.7637, 1324.7890, 1326.3207, 1329.7326, 1340.7775, 1357.7890, 1365.7525, 1390.8324, 1393.8069, 1407.7954, 1422.8033, 1434.8643, 1460.8550, 1475.8557, 1490.8367, 1493.8451, 1549.8969, 1598.9377, 1638.9642, 1684.0126, 1707.9051, 1716.9715, 1740.9356, 1789.0003, 1791.8887, 1804.0297, 1839.0630, 1847.0491, 1852.0436, 1983.1414, 1989.1250, 2001.0988, 2083.1533, 2128.1603, 2211.2706, 2215.3315, 2286.2807, 2299.3189, 2329.2726, 2343.2471, 2384.1325, 2441.2881, 2501.4182, 2705.3310, 2717.2530, 2848.5165, 2869.5420, 3223.3745, 3264.5742, 3312.3559

8. DXR_CLOBM **Mass:** 43041 **Score:** 57 **Expect:** 0.73 **Matches:** 12

1-deoxy-D-xylulose 5-phosphate reductoisomerase OS=Clostridium botulinum (strain Loch Maree / Type A3) OX=498214 GN=dxr PE=3 SV=

Observed	Mr(expt)	Mr(calc)	ppm	Start	End	Miss	Ions	Peptide
842.5642	841.5569	841.5637	-8.05	41	47	1	---	K.KVIEIIK.E
1193.6977	1192.6904	1192.6815	7.46	30	40	1	---	K.LVAISANKSYK.K
1320.6798	1319.6725	1319.7119	-29.82	207	218	1	---	R.KISIDSATLMNK.G
1329.7326	1328.7254	1328.7299	-3.44	113	125	1	---	K.AIESGKDIALANK.E
1343.7777	1342.7704	1342.8224	-38.72	42	52	1	---	K.VIEIIKEFKPK.Y
1357.7890	1356.7817	1356.7864	-3.46	126	138	0	---	K.ETLVVAGELVISK.A
1716.9715	1715.9642	1715.9206	25.4	166	181	1	---	K.EEVKNIIVTASGGPFR.G
1852.0436	1851.0364	1850.9713	35.2	238	253	0	---	K.VVVHPQSIVHSMVEYK.D
2083.1533	2082.1461	2082.1936	-22.83	119	138	1	---	K.DIALANKETLVVAGELVISK.A
2128.1603	2127.1531	2127.1398	6.23	318	338	0	---	K.GGVMPAILNGANEVAVDLFLK.G
2329.2726	2328.2653	2328.2511	6.10	318	340	1	---	K.GGVMPAILNGANEVAVDLFLKGK.I + Oxidation (M)
3312.3559	3311.3486	3311.6353	-86.54	238	267	1	---	K.VVVHPQSIVHSMVEYKDGSVIAQMATPDMK.L + Oxidation (M)

No match to: 720.3201, 721.3315, 724.3214, 804.3108, 865.5456, 870.5940, 881.3242, 969.5548, 973.5948, 982.5138, 1033.5958, 1037.6072, 1045.6282, 1047.6804, 1064.6488, 1072.6447, 1077.2496, 1082.6362, 1093.6181, 1107.6212, 1109.5910, 1118.5838, 1140.6515, 1153.6950, 1163.6855, 1164.6754, 1165.6684, 1179.6771, 1184.6858, 1195.6893, 1201.7343, 1229.8974, 1232.6901, 1253.7005, 1259.2801, 1259.7540, 1263.2247, 1263.7024, 1266.7035, 1277.7589, 1280.7521, 1283.7721, 1302.8009, 1306.7653, 1308.7528, 1310.3027, 1323.2817, 1323.7637, 1324.7890, 1326.3207, 1332.7618, 1340.7775, 1365.7525, 1373.7532, 1390.8324, 1393.8069, 1407.7954, 1422.8033, 1434.8643, 1460.8550, 1475.8557, 1490.8367, 1493.8451, 1549.8969, 1598.9377, 1638.9642, 1657.9317, 1684.0126, 1699.9639, 1707.9051, 1740.9356, 1781.0033, 1789.0003, 1791.8887, 1804.0297, 1839.0630, 1847.0491, 1983.1414, 1989.1250, 2001.0988, 2211.2706, 2215.3315, 2286.2807, 2299.3189, 2343.2471, 2384.1325, 2399.2011, 2441.2881, 2501.4182, 2510.3224, 2705.3310, 2717.2530, 2848.5165, 2869.5420, 3223.3745, 3264.5742

9. DXR_CLOB6 **Mass:** 43168 **Score:** 56 **Expect:** 0.94 **Matches:** 12

1-deoxy-D-xylulose 5-phosphate reductoisomerase OS=Clostridium botulinum (strain 657 / Type Ba4) OX=515621 GN=dxr PE=3 SV=1

Observed	Mr(expt)	Mr(calc)	ppm	Start	End	Miss	Ions	Peptide
842.5642	841.5569	841.5637	-8.05	41	47	1	---	K.KVIEIIK.E
1193.6977	1192.6904	1192.6815	7.46	30	40	1	---	K.LVAISANKSYK.K
1320.6798	1319.6725	1319.7119	-29.82	207	218	1	---	R.KISIDSATLMNK.G
1329.7326	1328.7254	1328.7299	-3.44	113	125	1	---	K.AIESGKDIALANK.E
1343.7777	1342.7704	1342.8224	-38.72	42	52	1	---	K.VIEIIKEFKPK.Y
1357.7890	1356.7817	1356.7864	-3.46	126	138	0	---	K.ETLVVAGELVISK.A
1716.9715	1715.9642	1715.9206	25.4	166	181	1	---	K.EEVKNIIVTASGGPFR.G
1852.0436	1851.0364	1850.9713	35.2	238	253	0	---	K.VVVHPQSIVHSMVEYK.D
2083.1533	2082.1461	2082.1936	-22.83	119	138	1	---	K.DIALANKETLVVAGELVISK.A
2128.1603	2127.1531	2127.1398	6.23	318	338	0	---	K.GGVMPAILNGANEVAVDLFLK.G
2329.2726	2328.2653	2328.2511	6.10	318	340	1	---	K.GGVMPAILNGANEVAVDLFLKGK.I + Oxidation (M)
3312.3559	3311.3486	3311.6353	-86.54	238	267	1	---	K.VVVHPQSIVHSMVEYKDGSVIAQMATPDMK.L + Oxidation (M)

No match to: 720.3201, 721.3315, 724.3214, 804.3108, 865.5456, 870.5940, 881.3242, 969.5548, 973.5948, 982.5138, 1033.5958, 1037.6072, 1045.6282, 1047.6804, 1064.6488, 1072.6447, 1077.2496, 1082.6362, 1093.6181, 1107.6212, 1109.5910, 1118.5838, 1140.6515, 1153.6950, 1163.6855, 1164.6754, 1165.6684, 1179.6771, 1184.6858, 1195.6893, 1201.7343, 1229.8974, 1232.6901, 1253.7005, 1259.2801, 1259.7540, 1263.2247, 1263.7024, 1266.7035, 1277.7589, 1280.7521, 1283.7721, 1302.8009, 1306.7653, 1308.7528, 1310.3027, 1323.2817, 1323.7637, 1324.7890, 1326.3207, 1332.7618, 1340.7775, 1365.7525, 1373.7532, 1390.8324, 1393.8069, 1407.7954, 1422.8033, 1434.8643, 1460.8550, 1475.8557, 1490.8367, 1493.8451, 1549.8969, 1598.9377, 1638.9642, 1657.9317, 1684.0126, 1699.9639, 1707.9051, 1740.9356, 1781.0033, 1789.0003, 1791.8887, 1804.0297, 1839.0630, 1847.0491, 1983.1414, 1989.1250, 2001.0988, 2211.2706, 2215.3315, 2286.2807, 2299.3189, 2343.2471, 2384.1325, 2399.2011, 2441.2881, 2501.4182, 2510.3224, 2705.3310, 2717.2530, 2848.5165, 2869.5420, 3223.3745, 3264.5742

10. DXR_CLOBJ **Mass:** 43182 **Score:** 56 **Expect:** 0.94 **Matches:** 12

1-deoxy-D-xylulose 5-phosphate reductoisomerase OS=Clostridium botulinum (strain Kyoto / Type A2) OX=536232 GN=dxr PE=3 SV=1

Observed	Mr(expt)	Mr(calc)	ppm	Start	End	Miss	Ions	Peptide
842.5642	841.5569	841.5637	-8.05	41	47	1	---	K.KVIEIIK.E
1193.6977	1192.6904	1192.6815	7.46	30	40	1	---	K.LVAISANKSYK.K
1320.6798	1319.6725	1319.7119	-29.82	207	218	1	---	R.KISIDSATLMNK.G
1329.7326	1328.7254	1328.7299	-3.44	113	125	1	---	K.AIESGKDIALANK.E
1343.7777	1342.7704	1342.8224	-38.72	42	52	1	---	K.VIEIIKEFKPK.Y
1357.7890	1356.7817	1356.7864	-3.46	126	138	0	---	K.ETLVVAGELVISK.A
1716.9715	1715.9642	1715.9206	25.4	166	181	1	---	K.EEVKNIIVTASGGPFR.G
1852.0436	1851.0364	1850.9713	35.2	238	253	0	---	K.VVVHPQSIVHSMVEYK.D
2083.1533	2082.1461	2082.1936	-22.83	119	138	1	---	K.DIALANKETLVVAGELVISK.A
2128.1603	2127.1531	2127.1398	6.23	318	338	0	---	K.GGVMPAILNGANEVAVDLFLK.G
2329.2726	2328.2653	2328.2511	6.10	318	340	1	---	K.GGVMPAILNGANEVAVDLFLKGK.I + Oxidation (M)
3312.3559	3311.3486	3311.6353	-86.54	238	267	1	---	K.VVVHPQSIVHSMVEYKDGSVIAQMATPDMK.L + Oxidation (M)

No match to: 720.3201, 721.3315, 724.3214, 804.3108, 865.5456, 870.5940, 881.3242, 969.5548, 973.5948, 982.5138, 1033.5958, 1037.6072, 1045.6282, 1047.6804, 1064.6488, 1072.6447, 1077.2496, 1082.6362, 1093.6181, 1107.6212, 1109.5910, 1118.5838, 1140.6515, 1153.6950, 1163.6855, 1164.6754, 1165.6684, 1179.6771, 1184.6858, 1195.6893, 1201.7343, 1229.8974, 1232.6901, 1253.7005, 1259.2801, 1259.7540, 1263.2247, 1263.7024, 1266.7035, 1277.7589, 1280.7521, 1283.7721, 1302.8009, 1306.7653, 1308.7528, 1310.3027, 1323.2817, 1323.7637, 1324.7890, 1326.3207, 1332.7618, 1340.7775, 1365.7525, 1373.7532, 1390.8324, 1393.8069, 1407.7954, 1422.8033, 1434.8643, 1460.8550, 1475.8557, 1490.8367, 1493.8451, 1549.8969, 1598.9377, 1638.9642, 1657.9317, 1684.0126, 1699.9639, 1707.9051, 1740.9356, 1781.0033, 1789.0003, 1791.8887, 1804.0297, 1839.0630, 1847.0491, 1983.1414, 1989.1250, 2001.0988, 2211.2706, 2215.3315, 2286.2807, 2299.3189, 2343.2471, 2384.1325, 2399.2011, 2441.2881, 2501.4182, 2510.3224, 2705.3310, 2717.2530, 2848.5165, 2869.5420, 3223.3745, 3264.5742

11. DXR_CLOBL **Mass:** 43212 **Score:** 56 **Expect:** 0.94 **Matches:** 12

1-deoxy-D-xylulose 5-phosphate reductoisomerase OS=Clostridium botulinum (strain Langeland / NCTC 10281 / Type F) OX=441772 GN=d

Observed	Mr(expt)	Mr(calc)	ppm	Start	End	Miss	Ions	Peptide
842.5642	841.5569	841.5637	-8.05	41	47	1	---	K.KVIEIIK.E
1193.6977	1192.6904	1192.6815	7.46	30	40	1	---	K.LVAISANKSYK.K
1320.6798	1319.6725	1319.7119	-29.82	207	218	1	---	R.KISIDSATLMNK.G
1329.7326	1328.7254	1328.7299	-3.44	113	125	1	---	K.AIESGKDIALANK.E
1343.7777	1342.7704	1342.8224	-38.72	42	52	1	---	K.VIEIIKEFKPK.Y
1357.7890	1356.7817	1356.7864	-3.46	126	138	0	---	K.ETLVVAGELVISK.A
1716.9715	1715.9642	1715.9206	25.4	166	181	1	---	K.EEVKNIIVTASGGPFR.G
1852.0436	1851.0364	1850.9713	35.2	238	253	0	---	K.VVVHPQSIVHSMVEYK.D
2083.1533	2082.1461	2082.1936	-22.83	119	138	1	---	K.DIALANKETLVVAGELVISK.A
2128.1603	2127.1531	2127.1398	6.23	318	338	0	---	K.GGVMPAILNGANEVAVDLFLK.G
2329.2726	2328.2653	2328.2511	6.10	318	340	1	---	K.GGVMPAILNGANEVAVDLFLKGK.I + Oxidation (M)
3312.3559	3311.3486	3311.6353	-86.54	238	267	1	---	K.VVVHPQSIVHSMVEYKDGSVIAQMATPDMK.L + Oxidation (M)

No match to: 720.3201, 721.3315, 724.3214, 804.3108, 865.5456, 870.5940, 881.3242, 969.5548, 973.5948, 982.5138, 1033.5958, 1037.6072, 1045.6282, 1047.6804, 1064.6488, 1072.6447, 1077.2496, 1082.6362, 1093.6181, 1107.6212, 1109.5910, 1118.5838, 1140.6515, 1153.6950, 1163.6855, 1164.6754, 1165.6684, 1179.6771, 1184.6858, 1195.6893, 1201.7343, 1229.8974, 1232.6901, 1253.7005, 1259.2801, 1259.7540, 1263.2247, 1263.7024, 1266.7035, 1277.7589, 1280.7521, 1283.7721, 1302.8009, 1306.7653, 1308.7528, 1310.3027, 1323.2817, 1323.7637, 1324.7890, 1326.3207, 1332.7618, 1340.7775, 1365.7525, 1373.7532, 1390.8324,

1393.8069, 1407.7954, 1422.8033, 1434.8643, 1460.8550, 1475.8557, 1490.8367, 1493.8451, 1549.8969, 1598.9377, 1638.9642, 1657.9317, 1684.0126, 1699.9639, 1707.9051, 1740.9356, 1781.0033, 1789.0003, 1791.8887, 1804.0297, 1839.0630, 1847.0491, 1983.1414, 1989.1250, 2001.0988, 2211.2706, 2215.3315, 2286.2807, 2299.3189, 2343.2471, 2384.1325, 2399.2011, 2441.2881, 2501.4182, 2510.3224, 2705.3310, 2717.2530, 2848.5165, 2869.5420, 3223.3745, 3264.5742

12. DXR_CLOBK **Mass:** 43169 **Score:** 55 **Expect:** 1 **Matches:** 12

1-deoxy-D-xylulose 5-phosphate reductoisomerase OS=Clostridium botulinum (strain Okra / Type B1) OX=498213 GN=dxr PE=3 SV=1

Observed	Mr(expt)	Mr(calc)	ppm	Start	End	Miss	Ions	Peptide
842.5642	841.5569	841.5637	-8.05	41	47	1	---	K.KVIEIIK.E
1193.6977	1192.6904	1192.6815	7.46	30	40	1	---	K.LVAISANKSYK.K
1320.6798	1319.6725	1319.7119	-29.82	207	218	1	---	R.KISIDSATLMNK.G
1329.7326	1328.7254	1328.7299	-3.44	113	125	1	---	K.AIESGKDIALANK.E
1343.7777	1342.7704	1342.8224	-38.72	42	52	1	---	K.VIEIIKEFKPK.Y
1357.7890	1356.7817	1356.7864	-3.46	126	138	0	---	K.ETLVVAGELVISK.A
1716.9715	1715.9642	1715.9206	25.4	166	181	1	---	K.EEVKNIIVTASGGPFR.G
1852.0436	1851.0364	1850.9713	35.2	238	253	0	---	K.VVVHPQSIVHSMVEYK.D
2083.1533	2082.1461	2082.1936	-22.83	119	138	1	---	K.DIALANKETLVVAGELVISK.A
2128.1603	2127.1531	2127.1398	6.23	318	338	0	---	K.GGVMPAILNGANEVAVDLFLK.G
2329.2726	2328.2653	2328.2511	6.10	318	340	1	---	K.GGVMPAILNGANEVAVDLFLKGK.I + Oxidation (M)
3312.3559	3311.3486	3311.6353	-86.54	238	267	1	---	K.VVVHPQSIVHSMVEYKDGSVIAQMATPDMK.L + Oxidation (M)

No match to: 720.3201, 721.3315, 724.3214, 804.3108, 865.5456, 870.5940, 881.3242, 969.5548, 973.5948, 982.5138, 1033.5958, 1037.6072, 1045.6282, 1047.6804, 1064.6488, 1072.6447, 1077.2496, 1082.6362, 1093.6181, 1107.6212, 1109.5910, 1118.5838, 1140.6515, 1153.6950, 1163.6855, 1164.6754, 1165.6684, 1179.6771, 1184.6858, 1195.6893, 1201.7343, 1229.8974, 1232.6901, 1253.7005, 1259.2801, 1259.7540, 1263.2247, 1263.7024, 1266.7035, 1277.7589, 1280.7521, 1283.7721, 1302.8009, 1306.7653, 1308.7528, 1310.3027, 1323.2817, 1323.7637, 1324.7890, 1326.3207, 1332.7618, 1340.7775, 1365.7525, 1373.7532, 1390.8324, 1393.8069, 1407.7954, 1422.8033, 1434.8643, 1460.8550, 1475.8557, 1490.8367, 1493.8451, 1549.8969, 1598.9377, 1638.9642, 1657.9317, 1684.0126, 1699.9639, 1707.9051, 1740.9356, 1781.0033, 1789.0003, 1791.8887, 1804.0297, 1839.0630, 1847.0491, 1983.1414, 1989.1250, 2001.0988, 2211.2706, 2215.3315, 2286.2807, 2299.3189, 2343.2471, 2384.1325, 2399.2011, 2441.2881, 2501.4182, 2510.3224, 2705.3310, 2717.2530, 2848.5165, 2869.5420, 3223.3745, 3264.5742

13. ERA_LACH4 **Mass:** 34027 **Score:** 54 **Expect:** 1.2 **Matches:** 7

GTPase Era OS=Lactobacillus helveticus (strain DPC 4571) OX=405566 GN=era PE=3 SV=1

Observed	Mr(expt)	Mr(calc)	ppm	Start	End	Miss	Ions	Peptide
842.5642	841.5569	841.5385	21.8	242	250	1	---	K.GIIIGKGGK.M
1195.6893	1194.6821	1194.6608	17.8	261	270	1	---	R.KEIEHLLGEK.V
1232.6901	1231.6828	1231.6635	15.7	133	142	0	---	K.LLLIMDSYHK.L
1253.7005	1252.6932	1252.6485	35.7	22	32	0	---	K.STLMNYLVGQK.V
1365.7525	1364.7453	1364.7466	-0.95	275	284	1	---	R.LWVKVQHNWR.S
1490.8367	1489.8294	1489.8140	10.3	103	115	1	---	K.GDQYIANLLKEVK.V
1549.8969	1548.8896	1548.8624	17.6	262	274	1	28	K.EIEHLLGEKVNLR.L

No match to: 720.3201, 721.3315, 724.3214, 804.3108, 865.5456, 870.5940, 881.3242, 969.5548, 973.5948, 982.5138, 1033.5958, 1037.6072, 1045.6282, 1047.6804, 1064.6488, 1072.6447, 1077.2496, 1082.6362, 1093.6181, 1107.6212, 1109.5910, 1118.5838, 1140.6515, 1153.6950, 1163.6855, 1164.6754, 1165.6684, 1179.6771, 1184.6858, 1193.6977, 1201.7343, 1229.8974, 1259.2801, 1259.7540, 1263.2247, 1263.7024, 1266.7035, 1277.7589, 1280.7521, 1283.7721, 1302.8009, 1306.7653, 1308.7528, 1310.3027, 1320.6798, 1323.2817, 1323.7637, 1324.7890, 1326.3207, 1329.7326, 1332.7618, 1340.7775, 1343.7777, 1357.7890, 1373.7532, 1390.8324, 1393.8069, 1407.7954, 1422.8033, 1434.8643, 1460.8550, 1475.8557, 1493.8451, 1598.9377, 1638.9642, 1657.9317, 1684.0126, 1699.9639, 1707.9051, 1716.9715, 1740.9356, 1781.0033, 1789.0003, 1791.8887, 1804.0297, 1839.0630, 1847.0491, 1852.0436, 1983.1414, 1989.1250, 2001.0988, 2083.1533, 2128.1603, 2211.2706, 2215.3315, 2286.2807, 2299.3189, 2329.2726, 2343.2471, 2384.1325, 2399.2011, 2441.2881, 2501.4182, 2510.3224, 2705.3310, 2717.2530, 2848.5165, 2869.5420, 3223.3745, 3264.5742, 3312.3559

14. ASSY_SOLUE **Mass:** 50458 **Score:** 54 **Expect:** 1.4 **Matches:** 11

Argininosuccinate synthase OS=Solibacter usitatus (strain Ellin6076) OX=234267 GN=argG PE=3 SV=1

Observed	Mr(expt)	Mr(calc)	ppm	Start	End	Miss	Ions	Peptide
1153.6950	1152.6877	1152.6139	64.0	206	215	1	---	K.DLEHLDKGVK.I
1253.7005	1252.6932	1252.6775	12.5	2	13	0	---	M.GNILQNLPAGEK.V
1266.7035	1265.6962	1265.6723	18.9	108	119	0	---	R.AVTGTMLVIAMK.E + 2 Oxidation (M)
1324.7890	1323.7817	1323.6531	97.2	271	282	0	---	R.HGLGVSDQIENR.I
1343.7777	1342.7704	1342.7569	10.1	355	366	1	---	R.AITGEVTVELRR.G
1357.7890	1356.7817	1356.7224	43.7	216	227	0	---	K.IVEPIMGVASWR.E
1373.7532	1372.7459	1372.7173	20.8	216	227	0	---	K.IVEPIMGVASWR.E + Oxidation (M)
1657.9317	1656.9244	1656.9021	13.4	213	227	1	---	K.GVKIVEPIMGVASWR.E + Oxidation (M)
1699.9639	1698.9566	1698.9417	8.76	351	365	1	---	R.WVARAITGEVTVELR.R
1989.1250	1988.1177	1987.9938	62.3	14	32	0	10	K.VGLAFSGGLDTSAAIHWMR.A
3223.3745	3222.3672	3222.6608	-91.10	2	32	1	---	M.GNILQNLPAGEKVGLAFSGGLDTSAAIHWMR.A

No match to: 720.3201, 721.3315, 724.3214, 804.3108, 842.5642, 865.5456, 870.5940, 881.3242, 969.5548, 973.5948, 982.5138, 1033.5958, 1037.6072, 1045.6282, 1047.6804, 1064.6488, 1072.6447, 1077.2496, 1082.6362, 1093.6181, 1107.6212, 1109.5910, 1118.5838, 1140.6515, 1163.6855, 1164.6754, 1165.6684, 1179.6771, 1184.6858, 1193.6977, 1195.6893, 1201.7343, 1229.8974, 1232.6901, 1259.2801, 1259.7540, 1263.2247, 1263.7024, 1277.7589, 1280.7521, 1283.7721, 1302.8009, 1306.7653, 1308.7528, 1310.3027, 1320.6798, 1323.2817, 1323.7637, 1326.3207, 1329.7326, 1332.7618, 1340.7775, 1365.7525, 1390.8324, 1393.8069, 1407.7954, 1422.8033, 1434.8643, 1460.8550, 1475.8557, 1490.8367, 1493.8451, 1549.8969, 1598.9377, 1638.9642, 1684.0126, 1707.9051, 1716.9715, 1740.9356, 1781.0033, 1789.0003, 1791.8887, 1804.0297, 1839.0630, 1847.0491, 1852.0436, 1983.1414, 2001.0988, 2083.1533, 2128.1603, 2211.2706, 2215.3315, 2286.2807, 2299.3189, 2329.2726, 2343.2471, 2384.1325, 2399.2011, 2441.2881, 2501.4182, 2510.3224, 2705.3310, 2717.2530, 2848.5165, 2869.5420, 3264.5742, 3312.3559

15. IOLG_RUBXD **Mass:** 37446 **Score:** 54 **Expect:** 1.5 **Matches:** 5

Inositol 2-dehydrogenase OS=Rubrobacter xylanophilus (strain DSM 9941 / NBRC 16129) OX=266117 GN=iolG PE=3 SV=1

Observed	Mr(expt)	Mr(calc)	ppm	Start	End	Miss	Ions	Peptide
1072.6447	1071.6374	1071.5924	42.0	35	44	0	---	R.LVAVADLDTR.R
1093.6181	1092.6108	1092.6114	-0.50	122	130	1	---	R.KLVQVGFMR.R + Oxidation (M)
1393.8069	1392.7996	1392.8089	-6.69	249	262	0	---	R.GTVQVPPLSGAIVR.R
1549.8969	1548.8896	1548.9100	-13.16	249	263	1	38	R.GTVQVPPLSGAIVRR.G
1740.9356	1739.9283	1739.8406	50.4	186	198	0	---	R.WFIEEEIEEVYVR.G

No match to: 720.3201, 721.3315, 724.3214, 804.3108, 842.5642, 865.5456, 870.5940, 881.3242, 969.5548, 973.5948, 982.5138, 1033.5958, 1037.6072, 1045.6282, 1047.6804, 1064.6488, 1077.2496, 1082.6362, 1107.6212, 1109.5910, 1118.5838, 1140.6515, 1153.6950, 1163.6855, 1164.6754, 1165.6684, 1179.6771, 1184.6858, 1193.6977, 1195.6893, 1201.7343, 1229.8974, 1232.6901, 1253.7005, 1259.2801, 1259.7540, 1263.2247, 1263.7024, 1266.7035, 1277.7589, 1280.7521, 1283.7721, 1302.8009, 1306.7653, 1308.7528, 1310.3027, 1320.6798, 1323.2817, 1323.7637, 1324.7890, 1326.3207, 1329.7326, 1332.7618, 1340.7775, 1343.7777, 1357.7890, 1365.7525, 1373.7532, 1390.8324, 1407.7954, 1422.8033, 1434.8643, 1460.8550, 1475.8557, 1490.8367, 1493.8451, 1598.9377, 1638.9642, 1657.9317, 1684.0126, 1699.9639, 1707.9051, 1716.9715, 1781.0033, 1789.0003, 1791.8887, 1804.0297, 1839.0630, 1847.0491, 1852.0436, 1983.1414, 1989.1250, 2001.0988, 2083.1533, 2128.1603, 2211.2706, 2215.3315, 2286.2807, 2299.3189, 2329.2726, 2343.2471, 2384.1325, 2399.2011, 2441.2881, 2501.4182, 2510.3224, 2705.3310, 2717.2530, 2848.5165, 2869.5420, 3223.3745, 3264.5742, 3312.3559

16. UREG_RHOPB **Mass:** 22253 **Score:** 53 **Expect:** 1.5 **Matches:** 6

Urease accessory protein UreG OS=Rhodopseudomonas palustris (strain BisB18) OX=316056 GN=ureG PE=3 SV=1

Observed	Mr(expt)	Mr(calc)	ppm	Start	End	Miss	Ions	Peptide
721.3315	720.3242	720.3476	-32.50	166	171	0	---	K.MQIDAK.R + Oxidation (M)
1064.6488	1063.6415	1063.5406	94.9	21	29	0	---	K.TALMDLLCK.T
1082.6362	1081.6289	1081.6244	4.19	134	144	1	---	K.IPSKGGPGITR.S
1259.7540	1258.7467	1258.7397	5.52	193	204	1	---	R.IVGFIEAKGGLR.A
1434.8643	1433.8570	1433.7734	58.3	21	32	1	---	K.TALMDLLCKTLR.D
1989.1250	1988.1177	1988.0435	37.3	10	29	1	23	R.VGIGGPVGSGKTALMDLLCK.T + Oxidation (M)

No match to: 720.3201, 724.3214, 804.3108, 842.5642, 865.5456, 870.5940, 881.3242, 969.5548, 973.5948, 982.5138, 1033.5958, 1037.6072, 1045.6282, 1047.6804, 1072.6447, 1077.2496, 1093.6181, 1107.6212, 1109.5910, 1118.5838, 1140.6515, 1153.6950, 1163.6855, 1164.6754, 1165.6684, 1179.6771, 1184.6858, 1193.6977, 1195.6893, 1201.7343, 1229.8974, 1232.6901, 1253.7005, 1259.2801, 1263.2247, 1263.7024, 1266.7035, 1277.7589, 1280.7521, 1283.7721, 1302.8009, 1306.7653, 1308.7528, 1310.3027, 1320.6798, 1323.2817, 1323.7637, 1324.7890, 1326.3207, 1329.7326, 1332.7618, 1340.7775, 1343.7777, 1357.7890, 1365.7525, 1373.7532, 1390.8324, 1393.8069, 1407.7954, 1422.8033, 1460.8550, 1475.8557, 1490.8367, 1493.8451, 1549.8969, 1598.9377, 1638.9642, 1657.9317, 1684.0126, 1699.9639, 1707.9051, 1716.9715, 1740.9356, 1781.0033, 1789.0003, 1791.8887, 1804.0297, 1839.0630, 1847.0491, 1852.0436, 1983.1414, 2001.0988, 2083.1533, 2128.1603, 2211.2706, 2215.3315, 2286.2807, 2299.3189, 2329.2726, 2343.2471, 2384.1325, 2399.2011, 2441.2881, 2501.4182, 2510.3224, 2705.3310, 2717.2530, 2848.5165, 2869.5420, 3223.3745, 3264.5742, 3312.3559

17. CCPA_STAEQ **Mass:** 36500 **Score:** 53 **Expect:** 1.8 **Matches:** 10

Catabolite control protein A OS=Staphylococcus epidermidis (strain ATCC 35984 / RP62A) OX=176279 GN=ccpA PE=3 SV=1

Observed	Mr(expt)	Mr(calc)	ppm	Start	End	Miss	Ions	Peptide
1037.6072	1036.5999	1036.5553	43.0	2	10	0	---	M.TVTIYDVAR.E
1140.6515	1139.6442	1139.5532	79.9	81	90	0	---	R.GLEDIATMYK.Y
1184.6858	1183.6785	1183.5907	74.2	1	10	0	---	-.MTVTIYDVAR.E + Oxidation (M)
1329.7326	1328.7254	1328.7425	-12.91	43	53	1	---	K.RLNYRPNAVAR.G
1373.7532	1372.7459	1372.6623	60.9	151	162	0	---	K.DDHIASVNIDFK.Q
1393.8069	1392.7996	1392.7361	45.6	2	13	1	---	M.TVTIYDVAREAR.V
1493.8451	1492.8378	1492.7409	64.9	163	175	0	---	K.QAAEEATQYLIEK.G
2083.1533	2082.1461	2081.9840	77.8	305	321	0	---	K.YMNDEEIENPNVILPHR.I
2286.2807	2285.2734	2285.1910	36.1	14	34	1	---	R.VSMATVSRVVNGNQNVKPETR.N
2848.5165	2847.5092	2847.3926	40.9	151	175	1	---	K.DDHIASVNIDFKQAAEEATQYLIEK.G

No match to: 720.3201, 721.3315, 724.3214, 804.3108, 842.5642, 865.5456, 870.5940, 881.3242, 969.5548, 973.5948, 982.5138, 1033.5958, 1045.6282, 1047.6804, 1064.6488, 1072.6447, 1077.2496, 1082.6362, 1093.6181, 1107.6212, 1109.5910, 1118.5838, 1153.6950, 1163.6855, 1164.6754, 1165.6684, 1179.6771, 1193.6977, 1195.6893, 1201.7343, 1229.8974, 1232.6901, 1253.7005, 1259.2801, 1259.7540, 1263.2247, 1263.7024, 1266.7035, 1277.7589, 1280.7521, 1283.7721, 1302.8009, 1306.7653, 1308.7528, 1310.3027, 1320.6798, 1323.2817, 1323.7637, 1324.7890, 1326.3207, 1332.7618, 1340.7775, 1343.7777, 1357.7890, 1365.7525, 1390.8324, 1407.7954, 1422.8033, 1434.8643, 1460.8550, 1475.8557, 1490.8367, 1549.8969, 1598.9377, 1638.9642, 1657.9317, 1684.0126, 1699.9639, 1707.9051, 1716.9715, 1740.9356, 1781.0033, 1789.0003, 1791.8887, 1804.0297, 1839.0630, 1847.0491, 1852.0436, 1983.1414, 1989.1250, 2001.0988, 2128.1603, 2211.2706, 2215.3315, 2299.3189, 2329.2726, 2343.2471, 2384.1325, 2399.2011, 2441.2881, 2501.4182, 2510.3224, 2705.3310, 2717.2530, 2869.5420, 3223.3745, 3264.5742, 3312.3559

18. CCPA_STAES **Mass:** 36500 **Score:** 53 **Expect:** 1.8 **Matches:** 10

Catabolite control protein A OS=Staphylococcus epidermidis (strain ATCC 12228) OX=176280 GN=ccpA PE=3 SV=2

Observed	Mr(expt)	Mr(calc)	ppm	Start	End	Miss	Ions	Peptide
1037.6072	1036.5999	1036.5553	43.0	2	10	0	---	M.TVTIYDVAR.E
1140.6515	1139.6442	1139.5532	79.9	81	90	0	---	R.GLEDIATMYK.Y
1184.6858	1183.6785	1183.5907	74.2	1	10	0	---	-.MTVTIYDVAR.E + Oxidation (M)
1329.7326	1328.7254	1328.7425	-12.91	43	53	1	---	K.RLNYRPNAVAR.G
1373.7532	1372.7459	1372.6623	60.9	151	162	0	---	K.DDHIASVNIDFK.Q
1393.8069	1392.7996	1392.7361	45.6	2	13	1	---	M.TVTIYDVAREAR.V
1493.8451	1492.8378	1492.7409	64.9	163	175	0	---	K.QAAEEATQYLIEK.G
2083.1533	2082.1461	2081.9840	77.8	305	321	0	---	K.YMNDEEIENPNVILPHR.I
2286.2807	2285.2734	2285.1910	36.1	14	34	1	---	R.VSMATVSRVVNGNQNVKPETR.N
2848.5165	2847.5092	2847.3926	40.9	151	175	1	---	K.DDHIASVNIDFKQAAEEATQYLIEK.G

No match to: 720.3201, 721.3315, 724.3214, 804.3108, 842.5642, 865.5456, 870.5940, 881.3242, 969.5548, 973.5948, 982.5138, 1033.5958, 1045.6282, 1047.6804, 1064.6488, 1072.6447, 1077.2496, 1082.6362, 1093.6181, 1107.6212, 1109.5910, 1118.5838, 1153.6950, 1163.6855, 1164.6754, 1165.6684, 1179.6771, 1193.6977, 1195.6893, 1201.7343, 1229.8974, 1232.6901, 1253.7005, 1259.2801, 1259.7540, 1263.2247, 1263.7024, 1266.7035, 1277.7589, 1280.7521, 1283.7721, 1302.8009, 1306.7653, 1308.7528, 1310.3027, 1320.6798, 1323.2817, 1323.7637, 1324.7890, 1326.3207, 1332.7618, 1340.7775, 1343.7777, 1357.7890, 1365.7525, 1390.8324, 1407.7954, 1422.8033, 1434.8643, 1460.8550, 1475.8557, 1490.8367, 1549.8969, 1598.9377, 1638.9642, 1657.9317, 1684.0126, 1699.9639, 1707.9051, 1716.9715, 1740.9356, 1781.0033, 1789.0003, 1791.8887, 1804.0297, 1839.0630, 1847.0491, 1852.0436, 1983.1414, 1989.1250, 2001.0988, 2128.1603, 2211.2706, 2215.3315, 2299.3189, 2329.2726, 2343.2471, 2384.1325, 2399.2011, 2441.2881, 2501.4182, 2510.3224, 2705.3310, 2717.2530, 2869.5420, 3223.3745, 3264.5742, 3312.3559

19. ACKA_PORG3 **Mass:** 43586 **Score:** 52 **Expect:** 2 **Matches:** 10

Acetate kinase OS=Porphyromonas gingivalis (strain ATCC 33277 / DSM 20709 / CIP 103683 / JCM 12257 / NCTC 11834 / 2561) OX=43194

Observed	Mr(expt)	Mr(calc)	ppm	Start	End	Miss	Ions	Peptide
721.3315	720.3242	720.3225	2.41	358	363	0	---	K.VNEGMR.G + Oxidation (M)
1037.6072	1036.5999	1036.5627	35.9	15	22	1	---	K.YKLLEMPK.G + Oxidation (M)
1072.6447	1071.6374	1071.5560	75.9	23	33	0	---	K.GDVLAQGGVEK.L
1163.6855	1162.6783	1162.5903	75.6	366	375	0	---	K.EMVISKPESK.V + Oxidation (M)
1332.7618	1331.7545	1331.7119	32.0	364	375	1	---	R.GKEMVISKPESK.V
1390.8324	1389.8251	1389.7650	43.3	2	14	1	---	M.KVLVLNCGSSSVK.Y
1460.8550	1459.8477	1459.7995	33.0	271	285	1	---	K.KSGVLGVSGVSSDLR.E
1549.8969	1548.8896	1548.8987	-5.88	204	219	0	14	R.IITAHIGNGASIAAIK.N
2510.3224	2509.3151	2509.3866	-28.49	51	72	1	---	K.VVLEKDMPEHTIAVEFILSVLK.D
3223.3745	3222.3672	3222.5656	-61.56	312	342	1	---	K.YVGAYAAAMGGVDVLVFTGGVGENQYTTREK.V

No match to: 720.3201, 724.3214, 804.3108, 842.5642, 865.5456, 870.5940, 881.3242, 969.5548, 973.5948, 982.5138, 1033.5958, 1045.6282, 1047.6804, 1064.6488, 1077.2496, 1082.6362, 1093.6181, 1107.6212, 1109.5910, 1118.5838, 1140.6515, 1153.6950, 1164.6754, 1165.6684, 1179.6771, 1184.6858, 1193.6977, 1195.6893, 1201.7343, 1229.8974, 1232.6901, 1253.7005, 1259.2801, 1259.7540, 1263.2247, 1263.7024, 1266.7035, 1277.7589, 1280.7521, 1283.7721, 1302.8009, 1306.7653, 1308.7528, 1310.3027, 1320.6798, 1323.2817, 1323.7637, 1324.7890, 1326.3207, 1329.7326, 1340.7775, 1343.7777, 1357.7890, 1365.7525, 1373.7532, 1393.8069, 1407.7954, 1422.8033, 1434.8643, 1475.8557, 1490.8367, 1493.8451, 1598.9377, 1638.9642, 1657.9317, 1684.0126, 1699.9639, 1707.9051, 1716.9715, 1740.9356, 1781.0033, 1789.0003, 1791.8887, 1804.0297, 1839.0630, 1847.0491, 1852.0436, 1983.1414, 1989.1250, 2001.0988, 2083.1533, 2128.1603, 2211.2706, 2215.3315, 2286.2807, 2299.3189, 2329.2726, 2343.2471, 2384.1325, 2399.2011, 2441.2881, 2501.4182, 2705.3310, 2717.2530, 2848.5165, 2869.5420, 3264.5742, 3312.3559

20. RPOC_PSYCK **Mass:** 155806 **Score:** 52 **Expect:** 2.1 **Matches:** 15

DNA-directed RNA polymerase subunit beta' OS=Psychrobacter cryohalolentis (strain K5) OX=335284 GN=rpoC PE=3 SV=1

Observed	Mr(expt)	Mr(calc)	ppm	Start	End	Miss	Ions	Peptide
982.5138	981.5065	981.4879	18.9	785	793	0	---	K.TANSGYLTR.R
1109.5910	1108.5837	1108.4893	85.2	691	700	0	---	K.AMMDNLATDK.I
1153.6950	1152.6877	1152.6866	0.89	380	390	0	---	K.LLSHGLATTIK.A
1184.6858	1183.6785	1183.7037	-21.28	310	320	1	---	R.AITGSNKRPLK.S
1201.7343	1200.7270	1200.6350	76.7	1273	1283	1	---	K.ALNAKLEAEDK.F

1283.7721	1282.7648	1282.7721	-5.71	830 - 841	1	---	K.LGELVLGRVTAR.D
1332.7618	1331.7545	1331.6834	53.4	677 - 687	1	---	K.VVDIWSRTNDK.V
1407.7954	1406.7882	1406.8180	-21.21	1238 - 1248	1	---	K.HIEVIIRQMLR.K
1549.8969	1548.8896	1548.8552	22.3	1083 - 1095	0	16	K.EVVYFLPAETIIR.V
1657.9317	1656.9244	1656.9232	0.71	119 - 132	1	---	R.IGLLLDMTLRDIER.V
1684.0126	1683.0054	1682.8952	65.5	1111 - 1127	1	---	R.VPQASSGTKDITGGLPR.V
1716.9715	1715.9642	1715.8624	59.3	293 - 307	1	---	R.MLQESVDALLDNGRR.G
1791.8887	1790.8814	1790.9315	-28.00	760 - 775	0	15	R.EGLTVLQYFISTHGAR.K
2083.1533	2082.1461	2081.9762	81.6	1139 - 1157	1	---	K.DHAIMAEMTGVVSFGKETK.G + 2 Oxidation (M)
2705.3310	2704.3238	2704.1893	49.7	572 - 593	0	---	R.LLIWNIMPVGMSFDECNEEMTK.K + 3 Oxidation (M)

No match to: 720.3201, 721.3315, 724.3214, 804.3108, 842.5642, 865.5456, 870.5940, 881.3242, 969.5548, 973.5948, 1033.5958, 1037.6072, 1045.6282, 1047.6804, 1064.6488, 1072.6447, 1077.2496, 1082.6362, 1093.6181, 1107.6212, 1118.5838, 1140.6515, 1163.6855, 1164.6754, 1165.6684, 1179.6771, 1193.6977, 1195.6893, 1229.8974, 1232.6901, 1253.7005, 1259.2801, 1259.7540, 1263.2247, 1263.7024, 1266.7035, 1277.7589, 1280.7521, 1302.8009, 1306.7653, 1308.7528, 1310.3027, 1320.6798, 1323.2817, 1323.7637, 1324.7890, 1326.3207, 1329.7326, 1340.7775, 1343.7777, 1357.7890, 1365.7525, 1373.7532, 1390.8324, 1393.8069, 1422.8033, 1434.8643, 1460.8550, 1475.8557, 1490.8367, 1493.8451, 1598.9377, 1638.9642, 1699.9639, 1707.9051, 1740.9356, 1781.0033, 1789.0003, 1804.0297, 1839.0630, 1847.0491, 1852.0436, 1983.1414, 1989.1250, 2001.0988, 2128.1603, 2211.2706, 2215.3315, 2286.2807, 2299.3189, 2329.2726, 2343.2471, 2384.1325, 2399.2011, 2441.2881, 2501.4182, 2510.3224, 2717.2530, 2848.5165, 2869.5420, 3223.3745, 3264.5742, 3312.3559

Search Parameters

Type of search : MS/MS Ion Search
Enzyme : Trypsin
Fixed modifications : Carbamidomethyl (C)
Variable modifications : Oxidation (M)
Mass values : Monoisotopic
Protein Mass : Unrestricted
Peptide Mass Tolerance : ± 100 ppm
Fragment Mass Tolerance: ± 2 Da
Max Missed Cleavages : 1
Instrument type : MALDI-TOF-TOF
Query1 (720.3201,1+): <no title>
Query2 (721.3315,1+): <no title>
Query3 (724.3214,1+): <no title>
Query4 (804.3108,1+): <no title>
Query5 (842.5642,1+): <no title>
Query6 (865.5456,1+): <no title>
Query7 (870.5940,1+): <no title>
Query8 (881.3242,1+): <no title>
Query9 (969.5548,1+): <no title>
Query10 (973.5948,1+): <no title>
Query11 (982.5138,1+): <no title>
Query12 (1033.5958,1+): <no title>
Query13 (1037.6072,1+): <no title>
Query14 (1045.6282,1+): <no title>
Query15 (1047.6804,1+): <no title>
Query16 (1064.6488,1+): <no title>
Query17 (1072.6447,1+): <no title>
Query18 (1077.2496,1+): <no title>
Query19 (1082.6362,1+): <no title>
Query20 (1093.6181,1+): <no title>
Query21 (1107.6212,1+): <no title>
Query22 (1109.5910,1+): <no title>
Query23 (1118.5838,1+): <no title>
Query24 (1140.6515,1+): <no title>
Query25 (1153.6950,1+): <no title>
Query26 (1163.6855,1+): <no title>
Query27 (1164.6754,1+): <no title>
Query28 (1165.6684,1+): <no title>
Query29 (1179.6771,1+): <no title>
Query30 (1184.6858,1+): <no title>
Query31 (1193.6977,1+): <no title>
Query32 (1195.6893,1+): <no title>
Query33 (1201.7343,1+): <no title>
Query34 (1229.8974,1+): <no title>
Query35 (1232.6901,1+): <no title>
Query36 (1253.7005,1+): <no title>
Query37 (1259.2801,1+): <no title>
Query38 (1259.7540,1+): <no title>
Query39 (1263.2247,1+): <no title>
Query40 (1263.7024,1+): <no title>
Query41 (1266.7035,1+): <no title>
Query42 (1277.7589,1+): <no title>
Query43 (1280.7521,1+): <no title>
Query44 (1283.7721,1+): <no title>
Query45 (1302.8009,1+): <no title>
Query46 (1306.7653,1+): <no title>
Query47 (1308.7528,1+): <no title>
Query48 (1310.3027,1+): <no title>
Query49 (1320.6798,1+): <no title>
Query50 (1323.2817,1+): <no title>
Query51 (1323.7637,1+): <no title>
Query52 (1324.7890,1+): <no title>
Query53 (1326.3207,1+): <no title>
Query54 (1329.7326,1+): <no title>
Query55 (1332.7618,1+): <no title>
Query56 (1340.7775,1+): <no title>
Query57 (1343.7777,1+): <no title>
Query58 (1357.7890,1+): <no title>
Query59 (1365.7525,1+): <no title>
Query60 (1373.7532,1+): <no title>
Query61 (1390.8324,1+): <no title>
Query62 (1393.8069,1+): <no title>
Query63 (1407.7954,1+): <no title>
Query64 (1422.8033,1+): <no title>
Query65 (1434.8643,1+): <no title>
Query66 (1460.8550,1+): <no title>
Query67 (1475.8557,1+): <no title>
Query68 (1490.8367,1+): <no title>
Query69 (1493.8451,1+): <no title>
Query70 (1549.8969,1+): <no title>
Query71 (1598.9377,1+): <no title>

```
 Query72 (1638.9642,1+): <no title>
 Query73 (1657.9317,1+): <no title>
 Query74 (1684.0126,1+): <no title>
 Query75 (1699.9639,1+): <no title>
 Query76 (1707.9051,1+): <no title>
 Query77 (1716.9715,1+): <no title>
 Query78 (1740.9356,1+): <no title>
 Query79 (1781.0033,1+): <no title>
 Query80 (1789.0003,1+): <no title>
 Query81 (1791.8887,1+): <no title>
 Query82 (1804.0297,1+): <no title>
 Query83 (1839.0630,1+): <no title>
 Query84 (1847.0491,1+): <no title>
 Query85 (1852.0436,1+): <no title>
 Query86 (1983.1414,1+): <no title>
 Query87 (1989.1250,1+): <no title>
 Query88 (2001.0988,1+): <no title>
 Query89 (2083.1533,1+): <no title>
 Query90 (2128.1603,1+): <no title>
 Query91 (2211.2706,1+): <no title>
 Query92 (2215.3315,1+): <no title>
 Query93 (2286.2807,1+): <no title>
 Query94 (2299.3189,1+): <no title>
 Query95 (2329.2726,1+): <no title>
 Query96 (2343.2471,1+): <no title>
 Query97 (2384.1325,1+): <no title>
 Query98 (2399.2011,1+): <no title>
 Query99 (2441.2881,1+): <no title>
Query100 (2501.4182,1+): <no title>
Query101 (2510.3224,1+): <no title>
Query102 (2705.3310,1+): <no title>
Query103 (2717.2530,1+): <no title>
Query104 (2848.5165,1+): <no title>
Query105 (2869.5420,1+): <no title>
Query106 (3223.3745,1+): <no title>
Query107 (3264.5742,1+): <no title>
Query108 (3312.3559,1+): <no title>
```

Mascot: http://www.matrixscience.com/
